# *Ortho*-Selective amination of arene carboxylic acids *via* rearrangement of acyl *O*-hydroxylamines†

**DOI:** 10.1039/d3sc03293k

**Published:** 2023-08-18

**Authors:** James E. Gillespie, Nelson Y. S. Lam, Robert J. Phipps

**Affiliations:** a Yusuf Hamied Department of Chemistry, University of Cambridge Lensfield Road Cambridge CB2 1EW UK rjp71@cam.ac.uk

## Abstract

Direct amination of arene C–H bonds is an attractive disconnection to form aniline-derived building blocks. This transformation presents significant practical challenges; classical methods for *ortho*-selective amination require strongly acidic or forcing conditions, while contemporary catalytic processes often require bespoke directing groups and/or precious metal catalysis. We report a mild and procedurally straightforward *ortho*-selective amination of arene carboxylic acids, arising from a facile rearrangement of acyl *O*-hydroxylamines without requiring precious metal catalysts. A broad scope of benzoic acid substrates are compatible and the reaction can be applied to longer chain arene carboxylic acids. Mechanistic studies probe the specific requirement for trifluoroacetic acid in generating the active aminating agent, and suggest that two separate mechanisms may be operating in parallel in the presence of an iron catalyst: (i) an iron-nitrenoid intermediate and (ii) a radical chain pathway. Regardless of which mechanism is followed, high *ortho* selectivity is obtained, proposed to arise from the directivity (first) or attractive interactions (second) arising with the carboxylic acid motif.

## Abstract 2

Aromatic acyl *O*-hydroxylamines, synthesised in one straightforward step, undergo rearrangement under simple conditions to give *ortho*-aminated arenes. For benzoic acid-derived substrates, this leads directly to unprotected anthranilic acids.

## Graphical abstract

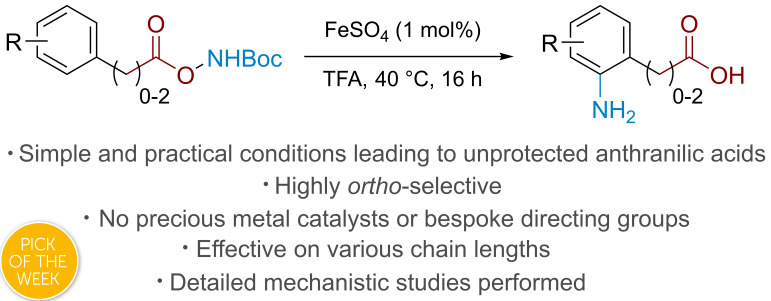

## Introduction

**Fig. 1:**
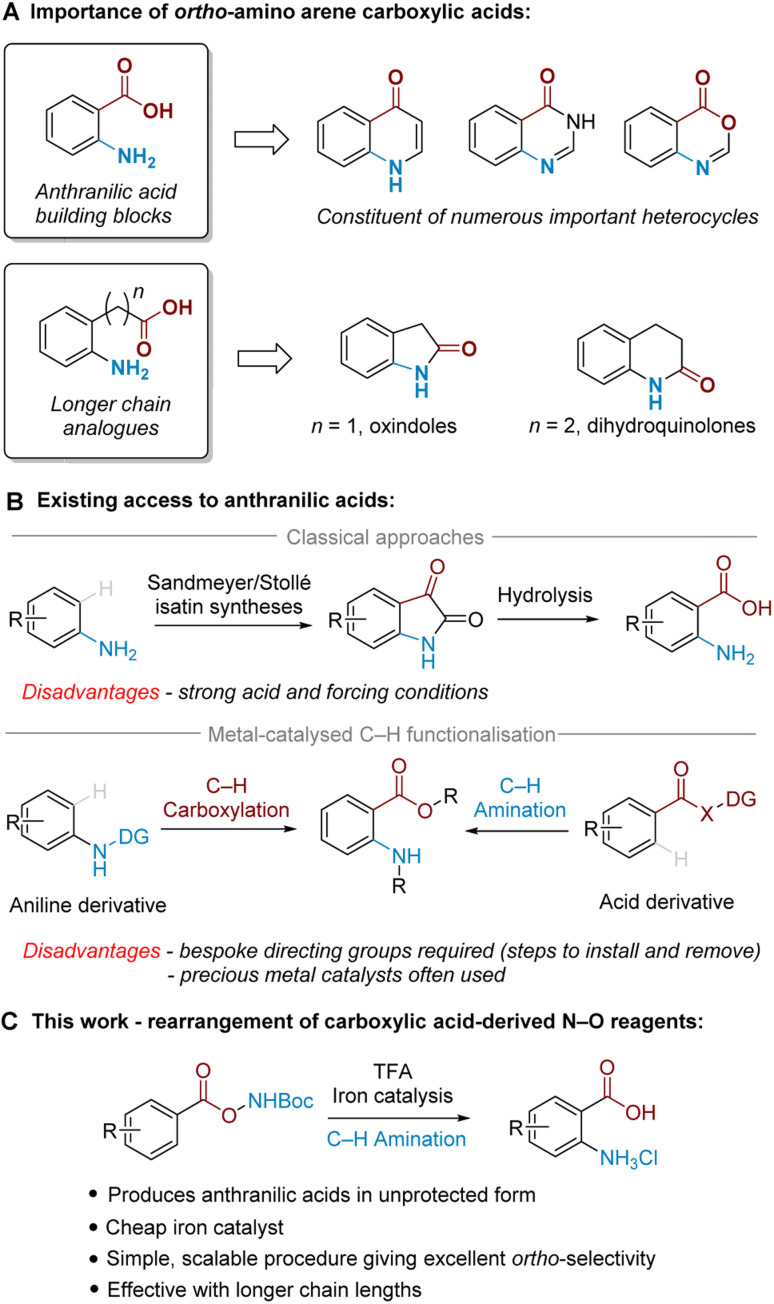
Background and summary of the present work.

*Ortho*-Amino arene carboxylic acids are prevalent motifs in bioactive natural products and medicinal agents, present either explicitly as a discrete unit, or implicitly embedded within the structure of heterocycles. *Ortho*-Aminobenzoic acids (*i.e.* anthranilic acids) are a particularly versatile building block present in a wide range of heterocycles, and can be identified within the core of a number of prominent medicinal compounds (Fig. 1A).^1^ By the same retrosynthetic logic, 2-oxindoles^2^ and dihydroquinolin-2(1*H*)-ones,^3^ among other heterocycles, can be analogously synthesised from their corresponding higher order *ortho*-aminoalkyl benzoic acid derivatives.

While many methods for *ortho*-amino arene carboxylic acid synthesis are known, few exist that can access them *via* direct C–H amination from the widely available parent arene carboxylic acid. For anthranilic acids, these are classically accessed by oxidative cleavage of isatins, which can be made by Sandmeyer^4^ or Stollé^5^ methodology *via* multistep synthesis from aniline (Fig. 1B, upper).^6^ Alternatively, nitration of benzoic acid followed by reduction is also known;^7^ this typically requires harsh conditions and only provides the required *ortho* relationship under specific substrate-imposed circumstances.^8^ More modern synthetic approaches utilise transition metal-catalysed *ortho*-selective C–H amination of benzoic acid derivatives^9^ or C–H carboxylation of aniline derivatives^10^ (Fig. 1B, lower). These methods, however, are often poorly generalisable to higher order arene carboxylic acids, and typically require precious metals and/or bespoke directing groups (DGs), meaning that separate DG installation/removal steps are needed to access the desired product and accessing unprotected anthranilic acids in many cases can be lengthy.^11^

We have been particularly interested in overcoming the regioselectivity challenges associated with arene amination using hydroxylamine-derived aminating agents.^12–16^ To this end, we recently developed an *ortho*-selective amination of aniline-derived sulfamate salts,^17^ and further discovered that sulfonyl *O*-hydroxylamines could undergo an aminative rearrangement to access *ortho*-sulfonyl anilines.^18^ In those reactions we believe that the *ortho* selectivity is dictated by attractive non-covalent interactions (NCIs) between the arenesulfonate anion and a putative cationic ammonium radical generated upon cleavage of the weak N–O bond.^19,20^

In the present work, we disclose the finding that arene carboxylic acid-derived N–O reagents can undergo a highly *ortho*-selective aminative rearrangement upon simple treatment with trifluoroacetic acid, with reactivity enhanced by the addition of only 1 mol% of an iron catalyst (Fig. 1C). This is surprising because benzoic acid-derived N–O reagents have been used with increasing frequency in recent years as sources of electrophilic nitrogen, and their ability to ‘self-aminate’ under mild conditions with high regioselectivity has not yet been reported. Complementing the toolkit of contemporary amination methods, we believe that this mild and straightforward *ortho*-amination protocol will be of significant practical utility for the following reasons: it produces valuable *ortho*-aminated acids in unprotected form, is scalable with precursors accessed in a single step from readily available materials, is generalisable to longer chain lengths and does not require precious metal catalysts. This study also probes the mechanism of the process, including the role of the iron catalyst as well as the unique role of TFA.

## Results and discussion

### Optimisation and scope investigation

We commenced our investigation with Boc-protected benzoyl *O*-hydroxylamine (1a), readily accessed in a single step from the corresponding benzoic acid. Treatment of 1a with five equivalents of TFA in CH_2_Cl_2_ at 40 °C resulted in product 2a, albeit in only 16% NMR yield (Table 1, entry 1). A solvent survey (see ESI† for full details) indicated that TFE was optimal in terms of yield and reproducibility (entry 2). The addition of 1 mol% of FeSO_4_·7H_2_O gave a further increase in the reaction yield (72%, entry 3), in line with our previous observations.^18^ A brief exploration of alternative acids indicated that TFA was singularly effective (entries 4–6; *vide infra* for discussion), and we note that the *ortho*-aminated product was the only product isomer observed.

**Table 1:** Reaction optimisation^a^

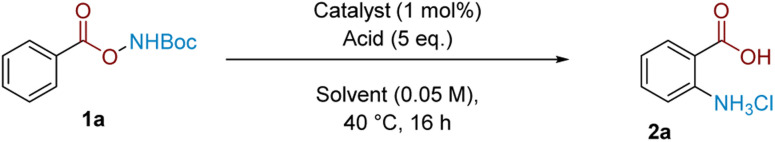				
Entry	Solvent	Acid	Catalyst	Yield (%)
1	CH_2_Cl_2_	TFA	—	16
2	TFE	TFA	—	68
**3**	**TFE**	**TFA**	**FeSO**_**4**_ **·7H**_**2**_ **O**	**72**
4	TFE	AcOH	FeSO_4_·7H_2_O	0
5	TFE	*p*TsOH	FeSO_4_·7H_2_O	0
6	TFE	TfOH	FeSO_4_·7H_2_O	2

a Yields determined by ^1^H NMR using 1,2-dimethoxyethane (DME) as an internal standard.

We proceeded to evaluate the substrate scope of the aminative rearrangement of benzoic acid derivatives to give substituted anthranilic acids. Evaluation of a small selection of substrates with and without an iron catalyst gave mixed results in which some substrates benefitted significantly from the presence of iron whilst others did not (see the ESI†). For this reason, we elected to perform the substrate scope in the presence of the iron catalyst to give the best consistency. A practical benefit of our process is that the majority of products could be easily purified through simple precipitation as the corresponding hydrochloride salt, and no special precautions were needed to exclude air or moisture from the reaction.

**Scheme 1:**
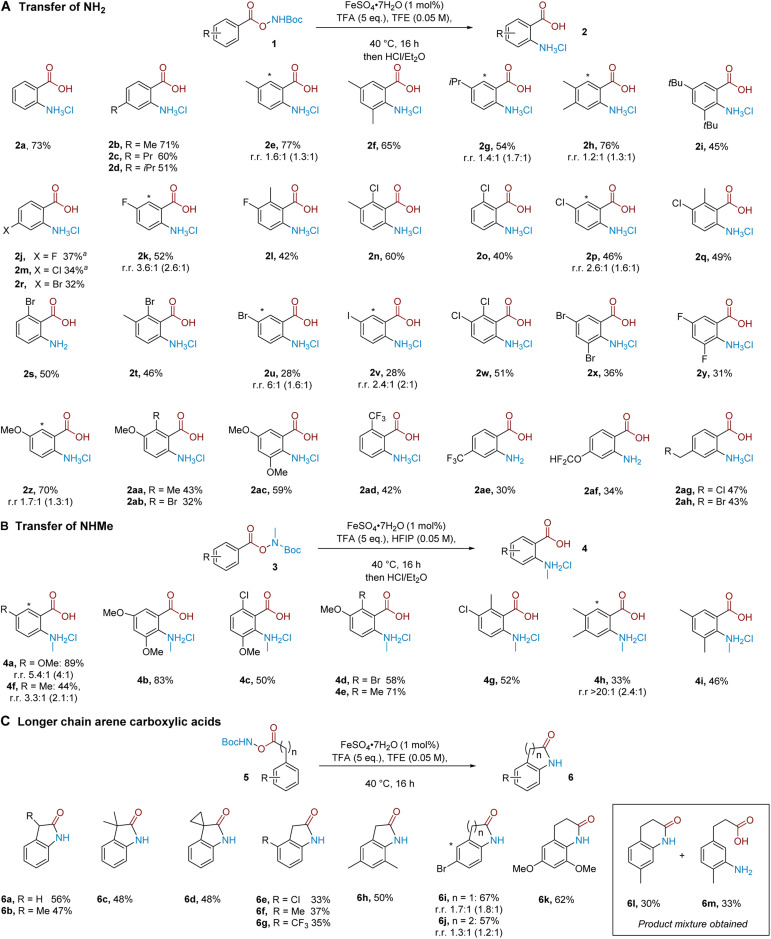
Substrate scope. Yields are isolated. All reactions conducted with no exclusion of air or moisture. Ratio (r.r.) refers to regioisomeric ratio of aminated product, where (*) denotes minor regioisomer. Ratio in parenthesis refers to r.r from crude material prior to purification.^*a*^ Isolated as free amine.

A series of alkyl-substituted benzoic acid-derived N–O reagents smoothly generated the desired products (Scheme 1A, 2a–2i). Similarly, halogen-substituted variants encompassing fluoride (2j–2l), also chloride (2m–2q), bromide (2r–2u) and iodide (2v) substituents worked in moderate to good yields, and poly-halogenated substrates (2w–2y) were also competent. These results showcase the orthogonality of our method to C–N bond formation through cross-coupling approaches, while preserving useful reactive handles for subsequent diversification. Electronically diverse aryl substituents were similarly competent; the reaction tolerates electron donating alkoxy groups (2z–2ac), as well as electron withdrawing trifluoromethyl (2ad, 2ae) and difluoromethoxy groups (2af). Pleasingly, alkyl substituents bearing a potentially labile benzylic chloride (2ag) or bromide (2ah) also undergo the rearrangement and could act as handles for further functionalisation. In cases where the substrate has two inequivalent *ortho* positions, the product was obtained as a mixture of regioisomers and by precipitation it was unfortunately not possible to separate these regioisomers. In these cases, there seems to be little sensitivity to sterics. For example, good reactivity was seen with 2g and 2i, which could be due to the small size of the putative aminium electrophile. In cases where yields were low, the remaining mass balance typically consists of the corresponding benzoic acid, where the N–O bond has been cleaved but productive arene amination has not occurred, rather than the presence of other regioisomers.

Next, we questioned whether mono-alkylated amines could be transferred to give *N*-alkyl anthranilic acids.^12*f*^ Pleasingly, NHMe transfer proved to be viable when the reaction was performed using HFIP as the solvent (Scheme 1B). In comparison with NH_2_ transfer, NHMe transfer typically required more electron rich substrates to obtain productive reactivity, the exact reason for which remains to be determined.^21^ Substrates bearing one (4a) or two (4b) methoxy groups on the aromatic ring were well tolerated, giving the desired products in excellent yields. Methoxy groups in combination with chlorine (4c), bromine (4d) and methyl (4e) substituents also proceeded smoothly. Monoalkylated substrates (4f) as well as substrates bearing an alkyl group alongside a chlorine atom (4g) worked in good isolated yield and two alkyl groups (4h–4i) were also tolerated.

We considered whether the reaction would tolerate the addition of a methylene unit between the carboxylate group and aromatic ring, which, after *in situ* cyclisation, should allow direct access to 2-oxindole products. We were pleased to find that a number of substrates proved amenable; the unsubstituted substrate could undergo the rearrangement followed by cyclisation to give the oxindole in 56% isolated yield (6a). α-Mono- and di-alkylated substrates were also amenable (6b–6d). Chloride (6e), methyl (6f) and trifluoromethyl (6g) substituents were tolerated at the arene *ortho*-position. A 3,5-dimethyl substrate (6h) as well as a substrate bearing a bromine atom in the *meta* position (6i) also gave the desired products. Extending the chain between the arene and the carboxylic acid further to include two methylene units, we found that the reaction still proceeds, now affording the corresponding 3,4-1*H*-dihydroquinolinone after *in situ* cyclisation (6j–6k). Interestingly, in the case of a *para*-methyl substituted substrate, an approximately 1 : 1 ratio of *ortho* : *meta* selectivity in the amination was observed, evidenced by isolation of dihydroquinolinone 6l and *meta*-aminated product 6m (Scheme 1C, inset box, see later for discussion).

**Scheme 2:**
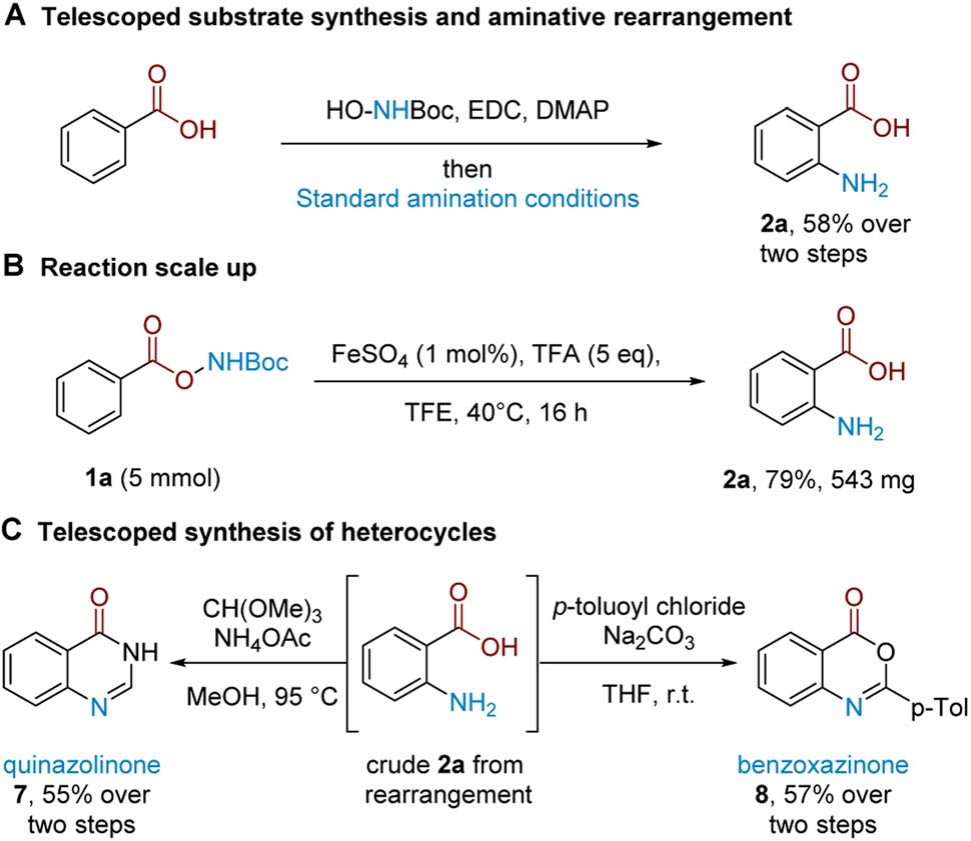
Synthetic applications.

A strength of this method lies in its procedural ease and that it leads directly to unprotected amines.^22^ We further demonstrate that substrate synthesis from benzoic acid can be successfully telescoped with the rearrangement protocol: coupling of *N*-Boc hydroxylamine followed by rearrangement gave anthranilic acid 2a in 58% isolated yield (Scheme 2A). The rearrangement protocol performs well on a 5 mmol scale and 2a could be isolated in 79% yield (Scheme 2B). The crude reaction mixture following amination could be telescoped with electrophilic reagents to generate related benzo-fused heterocycles (Scheme 2C); treatment of crude 2a with CH(OMe)_3_ and NH_4_OAc cleanly afforded quinazolinone 7 (see the ESI† for more quinazolinone derivatisations), while the corresponding treatment with *p*-toluoyl chloride under basic conditions afforded substituted benzoxazinone 8.

### Probing inter-*vs.* intramolecularity of the rearrangement

Having established the synthetic viability and scope, we sought to gain insight into its mechanism. Together with our previously-reported aminative rearrangement of sulfonyl N–O reagents, several questions remain outstanding relating to (i) the seemingly unique ability of TFA to promote the reaction amongst other Brønsted acids, (ii) the role of the iron catalyst, and (iii) whether the rearrangement proceeds *via* an intramolecular or intermolecular mechanism.

**Scheme 3:**
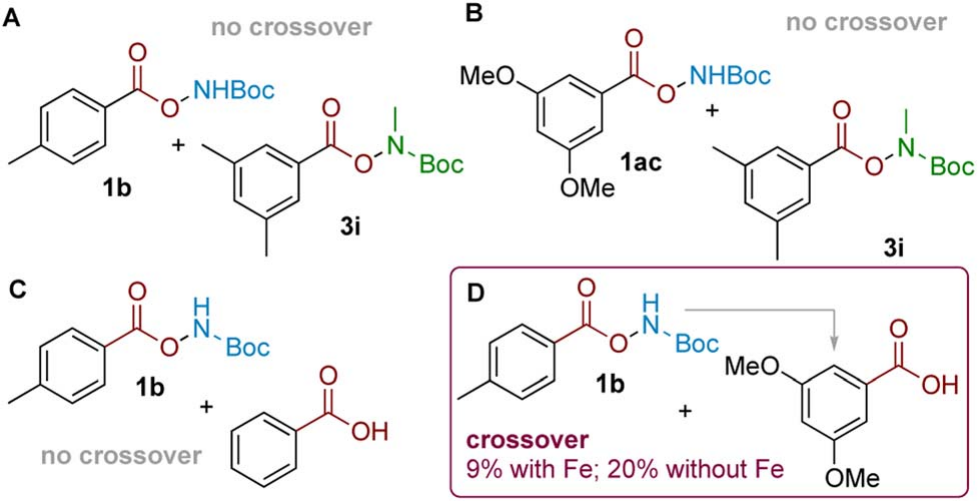
(A and B): Two pairs of crossover experiments. (C and D): Same-substrate class competition experiments indicating crossover only for arenes with different electronics.

In our previous work, we established that the related aminative rearrangement of sulfonyl *O*-hydroxylamines proceeded *via* an intermolecular mechanism, shown by extensive scrambling in crossover experiments.^18,23^ In contrast, analogous experiments in the present system—*i.e.* subjecting carboxyl substrates 1b (delivering NH_2_) and 3i (delivering NHMe) afforded no product crossover, which suggested that the reaction might proceed *via* an intramolecular mechanism (Scheme 3A). This was further confirmed with an alternative electronically-differentiated substrate pair (1ac, 3i; Scheme 3B). A further competition experiment was next conducted where an equimolar amount of an electronically-similar (Scheme 3C) or electronically-different (Scheme 3D) benzoic acid was exogenously added to the rearrangement reaction of 1b.^24^ This revealed that crossover only occurred when arenes of different electronics were used to give 2ac as the crossover product (Scheme 3D; 9% with Fe, 20% without; see the ESI† for details). This suggests that an intermolecular amination mechanism can occur, but only for electronically-activated substrates that can out-compete against a more favourable proximity-driven intramolecular/self-amination process. This outcome reconciles observations seen during our scope exploration, where we noted that the *para*-methyl substituted substrate 5l with a longer tether gave a 1 : 1 mixture of *ortho* and *meta* aminated products (Scheme 1C inset box; see the ESI† for further examples and discussion).

### Probing the role of trifluoroacetic acid

A full survey of Brønsted acids in the rearrangement indicated that reactivity was decoupled from acid p*K*_a_ values and that carboxylic acids bearing inductively withdrawing groups were uniquely enabling (Table 2, entries 4–7). Weaker carboxylic acids were evidently inferior at Boc deprotection giving little or no conversion to 2a (entries 8 and 9). Meanwhile, stronger acids (MsOH, *p*TsOH, TfOH) are presumed to cleave the Boc group, but this did not result in product formation, with only benzoic acid observed in crude reaction mixtures (entries 1–3). This very particular requirement mirrors the prominence of TFA in related aminations that utilise N–O reagents though the reasons for this remain unclear.^15*b*,18,25^

**Table 2:** Acid dependence study for 1a^a^

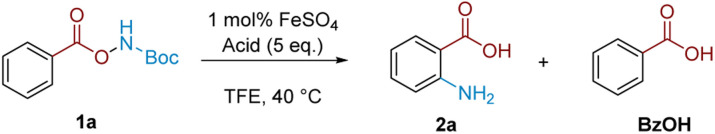				
Entry	Acid	Yield 2a	Yield 1a	Yield BzOH
1	TfOH	2	0	63
2	MsOH	0	0	100
3	*p*TsOH	0	0	60
**4**	**TFA**	**64**	**0**	**22**
**5**	**TCA**	**56**	**0**	**29**
**6**	**CF**_**2**_ **HCO**_**2**_ **H**	**34**	**17**	**34**
**7**	**C**_**2**_ **F**_**5**_ **CO**_**2**_ **H**	**56**	**0**	**19**
8	CF_3_CH_2_CO_2_H	4	70	14
9	AcOH	0	33	12

a Yields determined by ^1^H NMR using 1,2-DME as internal standard.

**Scheme 4:**
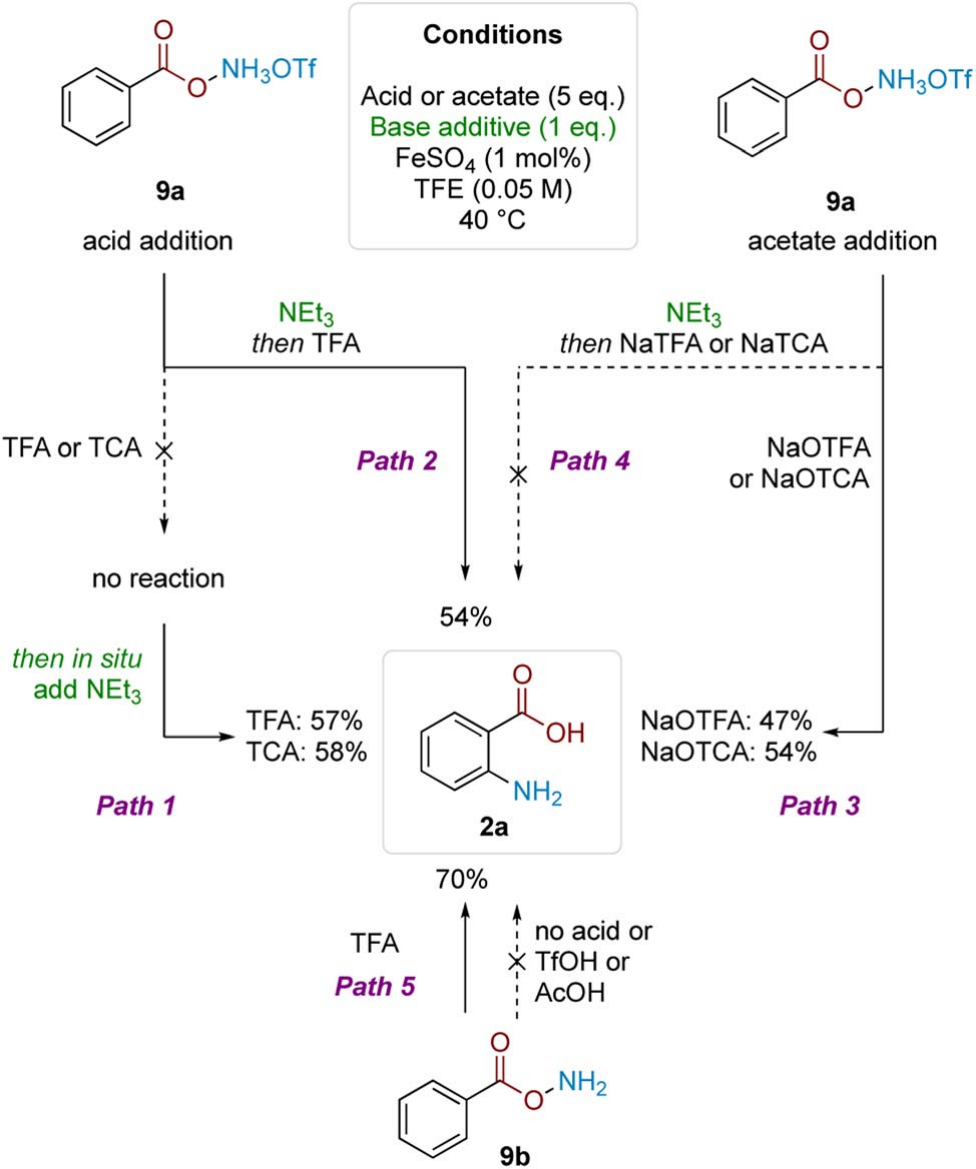
Studies to probe role of acid.

To gain insight into the reasons for this strict acid dependence, we next investigated the role of hydroxylamine protonation on reactivity. Anticipating that deprotection of the Boc group might complicate investigation of the role of acid in the subsequent mechanistic steps, we commenced the investigation using benzoyl *O*-hydroxylammonium triflate (9a) (Scheme 4). Subjecting 9a to TFA or TCA (5 eq.) did not give product formation. However, subsequent *in situ* treatment of the above reaction mixtures with Et_3_N (1 eq.) led to product formation in good yield (Path 1). Analogously, product formation was observed in similarly good yield when the sequence was reversed—1 eq. Et_3_N addition to 9a to form the free base, followed by addition of 5 eq. of TFA (Path 2). Hypothesising that formation of the trifluoroacetate anion was crucial for reactivity, we performed the reciprocal reaction by treating triflate salt 9a with 5 eq. of either sodium trifluoroacetate (NaTFA) or sodium trichloroacetate (NaTCA). In both cases, product formation was observed in good yields (Path 3), whereas none was observed when the reaction mixture was pretreated with Et_3_N to deprotonate the starting material prior to addition of NaTFA/NaTCA (Path 4). These results were corroborated using neutral benzoyl *O*-hydroxylamine (9b), where reactivity was only observed using TFA; acids that were more acidic but lacked carboxyl motifs (TfOH) or not acidic enough but contained carboxyl motifs (AcOH) did not react (Path 5). The result also showed that the free base by itself was not competent in the reaction. These observations pointed to the importance of acyl *O*-hydroxylamine protonation, as well as the carboxylate motif for productive reaction.

**Fig. 2:**
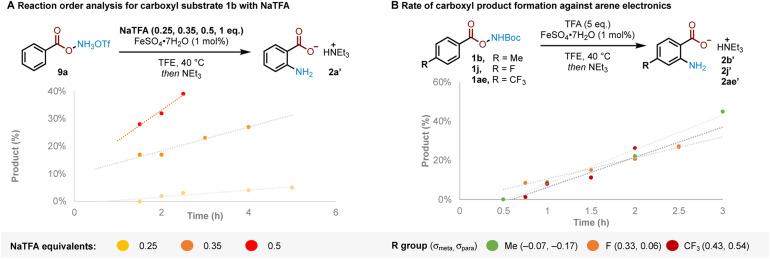
(A) Reaction order analysis in NaTFA for triflate salt 9a. (B) Rate-determining step (RDS) inference from rate of product formation with varying arene electronics. Yields determined by ^1^H NMR using 1,2-DME as an internal standard after quenching with Et_3_N and filtering through silica.

The results previously outlined (Scheme 4) indicate that the crucial role of TFA must occur after Boc deprotection. We sought to identify the point in the reaction pathway downstream of Boc deprotection (*i.e.* N–O bond cleavage, arene amination or deprotonation/rearomatisation) where these features in TFA were crucial for reactivity. Reaction order analysis indicated a positive order in NaTFA when starting from ammonium triflate salt 9a, leading us to conclude that TFA is likely involved in the RDS (Fig. 2A, see ESI† for detailed discussion). Supporting experiments were next conducted to position the RDS in the reaction pathway. In brief, no kinetic isotope effect was observed,^12*c*,*d*,16^ suggesting against deprotonation-rearomatisation as rate determining (see ESI† for details).^26^ Additionally, modulation of arene electronics did not meaningfully alter the rate of product formation, suggesting against arene amination (Fig. 2B). Altogether, these experiments position the RDS—where TFA plays a vital role—at the N–O bond cleavage step.^27^

### Probing the role of the iron catalyst

**Fig. 3:**
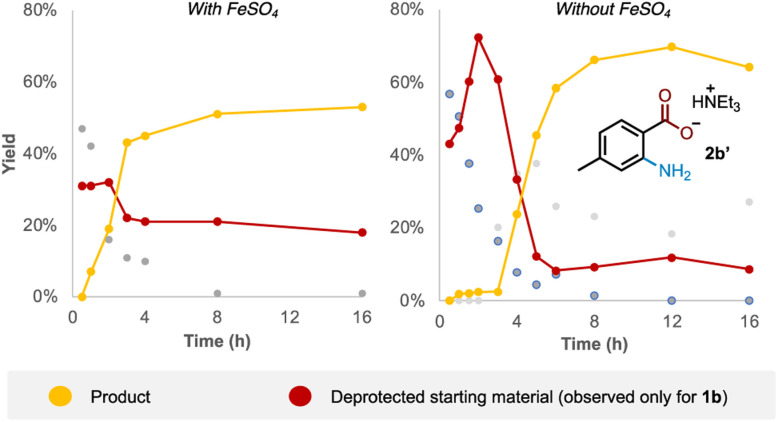
Time course reaction profile for 1b with and without iron catalysis.

Examining the reaction profile for 1b with and without iron catalysis revealed two quite different time course profiles (Fig. 3). Appreciable and consistent product formation was seen with Fe catalysis, whereas a clear induction period was observed in its absence, notably with an accumulation of the deprotected starting material during this period.

We next probed whether a radical mechanism was operative in the rearrangement of acyl *O*-hydroxylamines, which we had presumed to be the case for the corresponding sulfonyl substrates in our previous work. We subjected carboxyl substrate 1b to our standard Fe-catalysed and Fe-free aminative conditions with TEMPO as a stoichiometric additive, and compared the outcome by subjecting sulfonyl substrate 10 under the same conditions as a control. The addition of one equivalent of TEMPO heavily suppressed reactivity for the sulfonyl substrate 10 (Table 3; entries 1 and 2), as well as for the carboxyl substrate 1b without Fe catalysis (entry 3), strongly suggesting that a radical pathway is operating for these processes. However, reactivity was only modestly suppressed in the presence of Fe catalysis for 1b (entry 4). Furthermore there was no further reduction in product yield at superstoichiometric TEMPO loading (entry 5; 2 eq.). The latter experiment provides evidence against Fe solely serving as a radical initiator for a radical chain mechanism, although this possibility cannot be rigorously excluded at this stage.

**Table 3:** TEMPO inhibition study

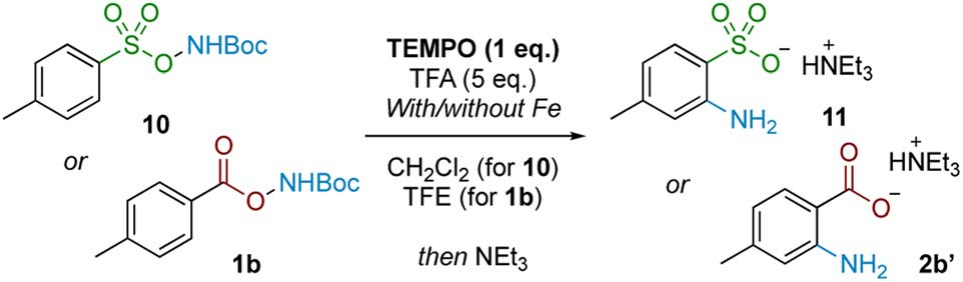			
Entry	Substrate	Catalyst (1 mol%)	Yield^a^ (%)
1	10	—	0% (82% without TEMPO)
2	10	FeSO_4_·7H_2_O	6% (70% without TEMPO)
3	1b	—	6% (57% without TEMPO)
4	1b	FeSO_4_·7H_2_O	26% (54% without TEMPO)
5^b^	1b	FeSO_4_·7H_2_O	25% (54% without TEMPO)

a Yields determined by ^1^H NMR using 1,2-dimethoxyethane as an internal standard after quenching with Et_3_N and filtration over silica.

b Two eq. TEMPO added.

**Scheme 5:**
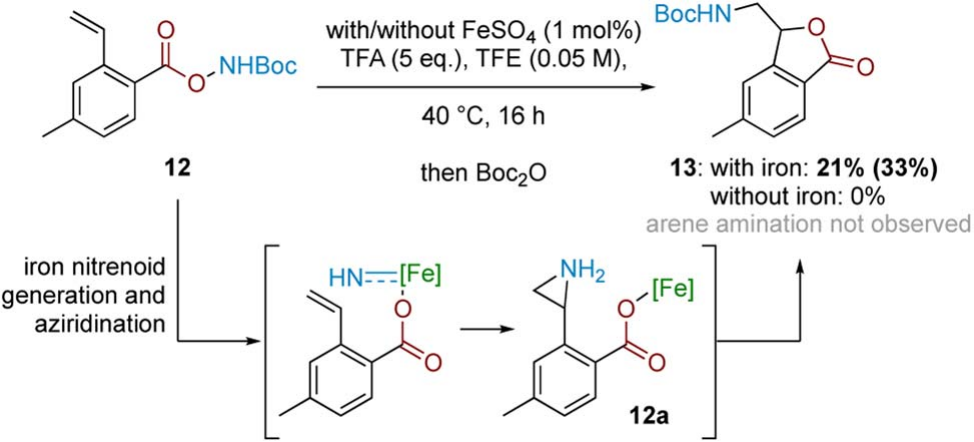
Mechanistic probe with styrenyl substrate 12. Bracketed yield denotes NMR yield.

In conjunction with the divergent reaction profile, this observation raises the possibility that a radical mechanism may not fully account for reactivity under an Fe-catalysed regimen, and that a parallel mechanism may also be operating. Further evidence supporting an alternative, iron-catalysed pathway was seen from divergent reaction outcomes after subjecting styrenyl substrate 12 under Fe and Fe-free conditions. While no reaction was observed under iron-free conditions, under iron-catalysed conditions aminolactone 13 was isolated, potentially arising from nucleophilic attack from the neighbouring carboxylate motif into an arizidinyl intermediate 12a (Scheme 5). This correlates with the established reactivity of iron nitrenoid-mediated alkene aziridination, although an alternative mechanism involving a carbocationic intermediate cannot be rigorously excluded at this stage.^27,28^

### Summary of mechanistic findings

**Scheme 6:**
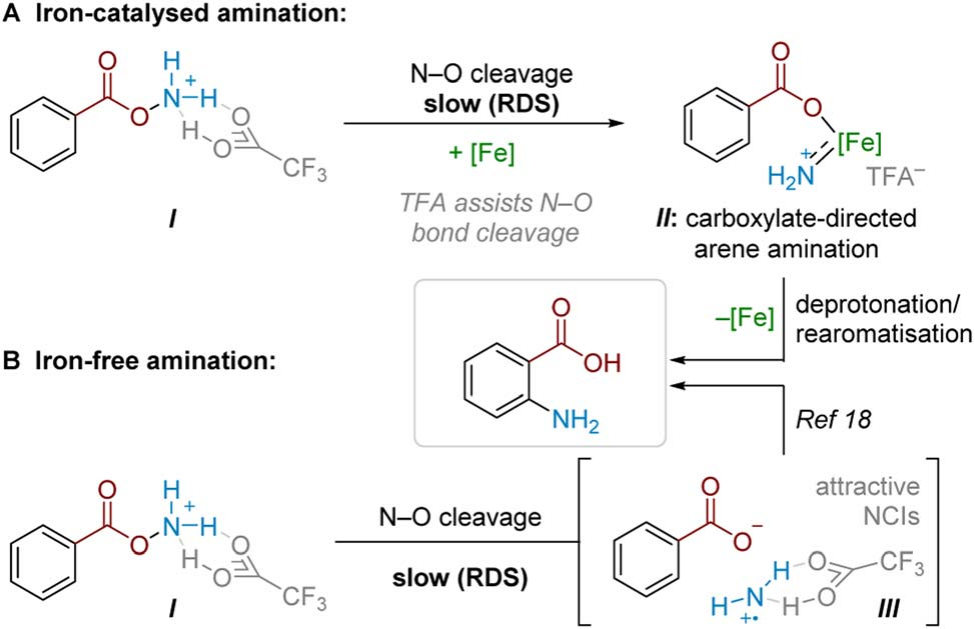
Proposed amination mechanisms.

Combining findings from our above mechanistic experiments, we believe that, following Boc deprotection, the N–O reagent is protonated by TFA to give I. Experiments suggest that TFA is a crucial component of the rate-determining N–O cleavage step, potentially rendering the N–O bond more susceptible to cleavage and/or assisting in stabilising the formed intermediate (Scheme 6A).^25,29,30^ In an Fe-catalysed pathway, we speculate that the active aminating agent may involve an electrophilic iron nitrenoid species, based on known reactivity (see Scheme 5) and literature precedents,^27,28,31^ where Fe-assisted N–O bond reduction could generate a carboxylate-bound iron nitrenoid complex II*in situ*.^27^ Several potential pathways (*e.g.* electrophilic amination, radical amination pathways *etc.*) could occur to deliver the product.^28*a*^ Taking our crossover studies into account,^32^*ortho* selectivity is likely driven by the directing effect by the carboxylate group acting as a ligand for iron, where intramolecular arene amination occurs at the most proximal position. Accounting for the modest reduction in yield through TEMPO addition, as well as the fact that some substrates undergo conversion in the absence of a catalyst, a radical chain pathway likely occurs in parallel. This pathway would then become the sole productive pathway in the absence of Fe catalysis. For some substrates, this radical-chain pathway delivers high yields, though variability in initiation, aminative capture or unaccounted side-reactivity of the aminium radical could lead to poorer generalisability in this pathway (see ESI† for a comparison of various substrates with and without iron catalyst). From I, reductive N–O cleavage, most likely as part of a radical chain process,^33^ would generate a putative TFA-stabilised planar aminium radical cation (III, Scheme 6B). The observed selectivity most likely results from attractive NCIs (ion-pairing and hydrogen bonding) with the associated carboxyl motif. This would place the aminating agent proximal to the *ortho* position, in analogy to previously proposed related systems.^17,18^

## Conclusions

We have developed a practical method for the *ortho*-amination of benzoic and higher order arene carboxylic acid derivatives that proceeds *via* a facile rearrangement of acyl *O*-hydroxylamine derivatives. Notable for its efficacy on electron-deficient benzoic acids, this mild and procedurally straightforward method directly generates valuable anthranilic acids. The protocol is also applicable for higher order arene carboxylic acids, which undergo *in situ* cyclisation to generate heterocyclic products. Mechanistic studies suggest that the two mechanisms may be occurring in parallel, and we suggest that an iron catalyst may permit an iron nitrenoid mechanism to take place in concurrence with a radical-based amination pathway. The generally excellent *ortho*-selectivity is imagined to be dictated by the directing effect of the pendant carboxyl group for the former and attractive NCIs for the latter. Further investigations revealed the crucial role of TFA in this class of rearrangement, indicating that substrate protonation and activation *via* the non-innocent trifluoroacetate motif likely assists in the generation of the active aminating agent.

This method complements the existing toolkit of site-selective C–N bond forming reaction, particularly on electron-deficient arenes, and represents a practical method to generate these ubiquitous motifs valuable in organic synthesis. Moreover, it has the potential be applied as a retrosynthetic strategy to expedite the synthesis of functionalised heterocyclic scaffolds from simple precursors. In addition, we hope that the mechanistic insights may be more broadly applicable to future reaction development of N–O reagents for electrophilic aminative processes.

## Data availability

All data associated with this work can be found in the ESI.†

## Author contributions

The manuscript was written through the contributions of all authors and all authors have given approval to the final version.

## Conflicts of interest

There are no conflicts to declare.

## Supplementary Material

SC-014-D3SC03293K-s001
