# Supplementary material for: Ortho-Selective amination of arene carboxylic acids via rearrangement of acyl O-hydroxylamines
Source: Chem Sci. 2023 Aug 18;14(37):10103–11. doi: 10.1039/d3sc03293k (PMC10530477; doi:10.1039/d3sc03293k)

Electronic Supplementary Material (ESI) for Chemical Science.
This journal is © The Royal Society of Chemistry 2023

Ortho-Selective Amination of Arene Carboxylic Acids *via* Rearrangement of Acyl *O*-Hydroxylamines

James E. Gillespie, Nelson Y. S. Lam and Robert J. Phipps*

Yusuf Hamied Department of Chemistry, University of Cambridge

Lensfield Road, Cambridge, United Kingdom, CB2 1EW

E-mail: rjp71@cam.ac.uk

Supporting Information

Contents

1 General Information **3**

2 General Procedures **4**

3 Optimisation **5**

4 Comparison of Fe and Fe-free Protocol **9**

5 Unsuccessful Substrates **10**

6 Substrate Synthesis **11**

7 Amination Products **35**

8 Reaction Utility **65**

9 Mechanistic Experiments **68**

9.1 Crossover Experiments 68

9.2 Competition Experiments 79

9.3 Chain Length Study 93

9.4 Probing the Effect of Acid 97

9.5 Stoichiometric Experiments with Protonated Triflate Salt **9a** 99

9.6 Kinetic Isotope Effect Studies 101

9.7 Reaction Order Determination 104

9.8 Time Course Investigation 115

9.9 Mechanistic Probes 124

10 References **131**

11 NMR Spectra **132**

1 General Information

NMR spectra: ^{1}H NMR spectra were recorded on a 700 MHz TXO Cryoprobe spectrometer, a 500 MHz Avance III Smart probe spectrometer, a 400 MHz Avance III HD Smart probe spectrometer, and a 400 MHz QNP Cryoprobe spectrometer. Chemical shifts are reported in parts per million (ppm) and the spectra are calibrated to the resonance resulting from incomplete deuteration of the solvent ($CDCl_3$: 7.26 ppm, CD_3OD: 3.31 ppm, $(CD_3)_2SO$: 2.50 ppm). ^{13}C NMR spectra were recorded with the same spectrometers with complete proton decoupling. Chemical shifts are reported in ppm with the solvent resonance as the internal standard ($^{13}CDCl_3$: 77.16 ppm, t; $^{13}CD_3OD$: 49.00, sept; $^{13}(CD_3)_2SO$: 39.52, sept). Data are reported as follows: chemical shift δ/ppm, integration (^{1}H only), multiplicity (s = singlet, d = doublet, t = triplet, q = quartet, p = pentet, sept = septet, h = heptet, br = broad, m = multiplet or combinations thereof; ^{13}C signals are singlets unless stated otherwise), coupling constants *J* in Hz. ^{1}H-COSY, DEPT-135, HSQC and HMBC were used where appropriate to facilitate structural determination of regioisomers. ^{19}F NMR spectra were recorded on a 400 MHz Avance III HD Smart probe spectrometer and a 500 MHz Avance III Smart probe spectrometer and were proton decoupled.

High Resolution Mass Spectrometry (HRMS): Samples were recorded on a Waters Micromass LCT Premier or a Waters Xevo G2-S or a Waters Vion QTof spectrometer using a positive electrospray ionisation (ESI+). The measured values are reported to 4 decimal places and are within ±5 ppm of the calculated value. The calculated values are based on the most abundant isotope.

Chromatography: Analytical thin layer chromatography was performed using precoated Merck glass backed silica gel plates (Silicagel 60 F254). Visualisation was by ultraviolet fluorescence (λ = 254 nm) and/or staining potassium permanganate ($KMnO_4$). Silica gel chromatography was performed using silica gel 60 (0.040-0.063 µm) from Material Harvest.

Reagents: Unless stated otherwise were used as supplied from commercial sources without further purification. CH_2Cl_2, THF, Et_2O, CH_3CN and MeOH were purified by distillation on site under an inert atmosphere via the following processes: THF and Et_2O were pre-dried over sodium wire then distilled from calcium hydride and lithium aluminium hydride; CH_3CN, MeOH and CH_2Cl_2 were distilled from calcium hydride.

Compound labelling: Throughout the manuscript, the ʹ notation (*e.g.* **2a** *vs.* **2a'**) specifically refers to the anionic carboxylate form of a given aminoarene carboxylic acid product as reported in the reaction scope table (Scheme 1 in the manuscript). Otherwise, the same compound number is used to refer to both the neutral as well as the cationic/protonated form for a given aminoarene carboxylic acid product.

2 General Procedures

General procedure A: Synthesis of benzoyloxycarbamates from the corresponding benzoic acid

DCC (1.1 eq.) was added to a stirred solution of the relevant benzoic acid (1 eq.) and *tert*-butyl *N*-hydroxycarbamate (1 eq.) in CH_2Cl_2 (0.2 M) at 0 ℃. The reaction was stirred at the same temperature for 1 h. The resulting suspension was filtered and the filtrate concentrated *in vacuo*. The resulting residue was purified by silica gel chromatography to give the desired product.

General procedure B: *N*-Methylation of benzoyloxycarbamates

Under an atmosphere of nitrogen, sodium hydride (1.2 eq.) was added portion-wise to a stirred solution of benzoyloxycarbamate substrate (1 eq.) in dry DMF (0.5 M) at 0 ℃. The reaction was stirred at 0 ℃ for 30 min. Methyl iodide (1.2 eq.) was then added and the reaction was allowed to warm to room temperature and stirred for 16 h. The reaction was then quenched with H_2O and the aqueous layer was extracted with EtOAc (3 x 50 mL). The combined organics were washed with brine (3 x 50 mL) and H_2O (2 x 50 mL), dried ($MgSO_4$), filtered and concentrated *in vacuo*. The crude residue was then purified by silica gel chromatography to give the desired product.

General procedure C: Synthesis of 2-aminobenzoic acid hydrochloride salts and 2-oxindoles

TFA (5 eq.) was added to a solution of $FeSO_4 \cdot 7H_2O$ (0.01 eq.) and the corresponding benzoyloxycarbamate or benzyloxycarbamate substrate (1 eq.) in TFE (0.05 M). The reaction was stirred at 40 ℃ for 16 h. The solvent was then removed under a stream of air. The resulting residue was passed through a short silica plug (*ca*. 5 cm), first eluting with a non-polar solvent system (typically 5-10% EtOAc in Pet. Ether) and then a more polar solvent system (typically 30-70% EtOAc in Pet. Ether). The filtrate corresponding to the more polar solvent system was retained and concentrated *in vacuo*. HCl in ether (2 M, 10 equiv) was added to the resulting residue and the resulting suspension was stirred at room temperature for 1 h. The precipitate was then collected by filtration to give the corresponding 2-aminobenzoic acid hydrochloride salt. In the case of the 2-oxindole products, purification was done by silica gel chromatography. In some cases, the 2-aminobenzoic acid product was also isolated as the free base by silica gel chromatography.

3 Optimisation

Typical procedure for optimisation reactions: To a 4 mL crimp top vial, TFA (57.0 mg, 0.5 mmol, 5 eq.) was added to a solution of $FeSO_4 \cdot 7H_2O$ (0.28 mg, 0.001 mmol, 0.01 eq.) and the corresponding benzoyloxycarbamate substrate (0.1 mmol, 1 eq.) in TFE (2 mL, 0.05 M). The reaction was stirred at 40 ℃ for 16 h. The solvent was then removed under a stream of air. The reaction was then filtered through a plug of silica (eluent:EtOAc) and the filtrate concentrated under a stream of air. The resulting residue was then analysed by ^1H NMR spectroscopy, using 1,2-dimethoxyethane as an internal standard.

Note: In some cases, the reaction was quenched with NEt_3 (200 µL). In these cases, CH_2Cl_2:MeOH (90:10) was used as the silica plug eluent.

*Table S1. Initial results for the rearrangement of **1a**.*

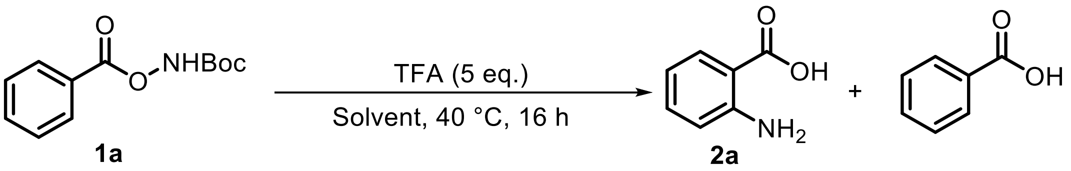

Entry[a]	Solvent	Conc (M)	Yield (2a)[b]	Yield (Benzoic Acid)
1	CH_2Cl_2	0.05	16	86
2	HFIP	0.2	69*	20

[a] Reactions were performed on a 0.1 mmol scale. [b] Yields for each product were assessed by ^1H NMR using 1,2-dimethoxyethane as an internal standard.

*Table S2. The effect of acid on the rearrangement of **1a**.*

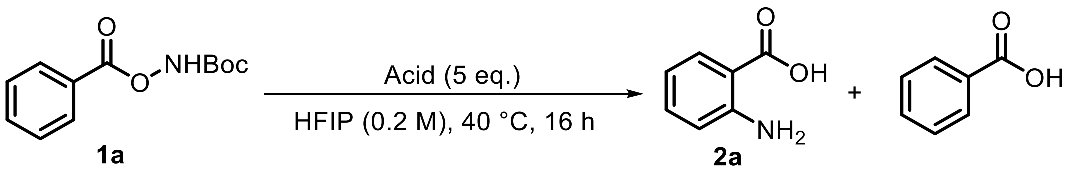

Entry[a]	Acid	Yield (2a)[b]	Yield (Benzoic Acid)	Yield (1a)
1	TFA	11*	27	50
2	*p*TsOH	0	57	0
3	TfOH	0	104	0
4	Conc. HCl	0	96	0

[a] Reactions were performed on a 0.1 mmol scale. [b] Yields for each product were assessed by ^1H NMR using 1,2-dimethoxyethane as an internal standard.

*Table S3. The effect of solvent on the rearrangement of **1a**.*

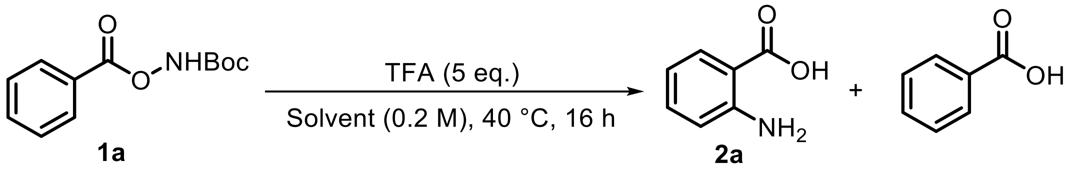

Entry[a]	Solvent	Yield (2a)[b]	Yield (Benzoic Acid)	Yield (1a)
1	HFIP	42*	0	43
2	TFE	55	27	0
3	CH_3CN	0	0	136
4	MeOH	0	0	96
5	$CHCl_3$	ND	ND	ND
6	EtOH	0	0	99

[a] Reactions were performed on a 0.1 mmol scale. [b] NMR yields for each product were assessed by ^{1}H NMR using 1,2-dimethoxyethane as an internal standard

*Table S4. The effect of TFA equivalents on the rearrangement of **1a**.*

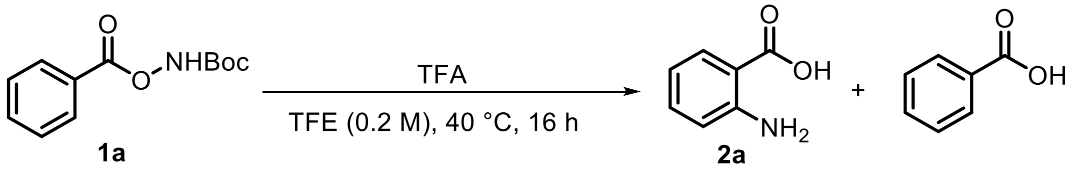

Entry[a]	TFA Eq.	Yield (2a)[b]	Yield (Benzoic Acid)
1	2	47	30
2	5	55	27
3	10	50	18
4	20	39	18

[a] Reactions were performed on a 0.1 mmol scale. [b] NMR yields for each product were assessed by ^{1}H NMR using 1,2-dimethoxyethane as an internal standard.

*Note: We observed poor reproducibility in yield when HFIP was used as the solvent. While the reaction was effective as shown in Table S1 (entry 2: 69%), the reaction was less effective as seen in Table S2 (entry 1: 11%) and Table S3 (entry 1: 42%). The use of TFE mitigates these reproducibility issues (Table S3, entry 2).

*Table S5. The effect of temperature on the rearrangement of **1a**.*

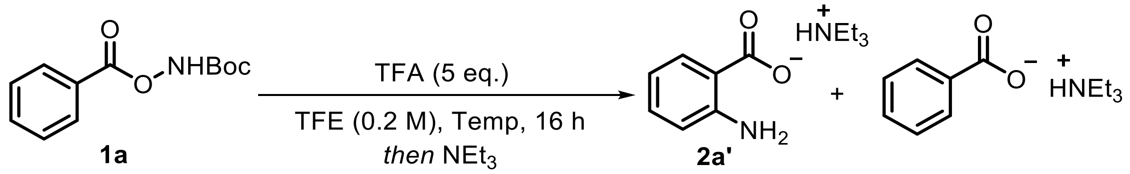

Entry[a]	Temp (°C)	Yield (2a')[b]	Yield (Benzoic acid)
1	rt	0	64
2	40	55	27
3	60	36	28
4	80	18	40

[a] Reactions were performed on a 0.1 mmol scale. [b] NMR yields for each product were assessed by ^{1}H NMR using 1,2-dimethoxyethane as an internal standard.

*Table S6. The effect of reaction concentration on the rearrangement of **1a**.*

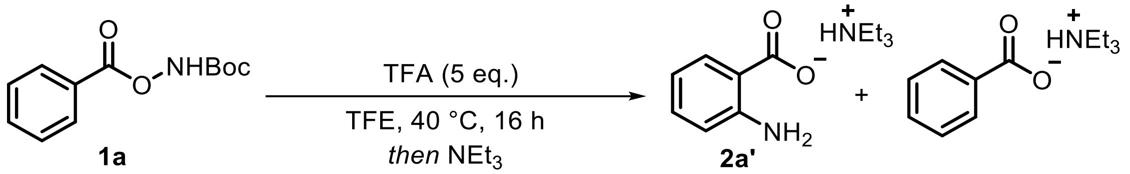

Entry[a]	Concentration (M)	Yield (2a')[b]	Yield (Benzoic acid)
1	1	39	30
2	0.5	40	22
3	0.2	55	27
4	0.1	59	22
5	0.05	68	19

[a] Reactions were performed on a 0.1 mmol scale. [b] NMR yields for each product were assessed by ^{1}H NMR using 1,2-dimethoxyethane as an internal standard.

*Table S7. The effect of reaction time on the rearrangement of **1a**.*

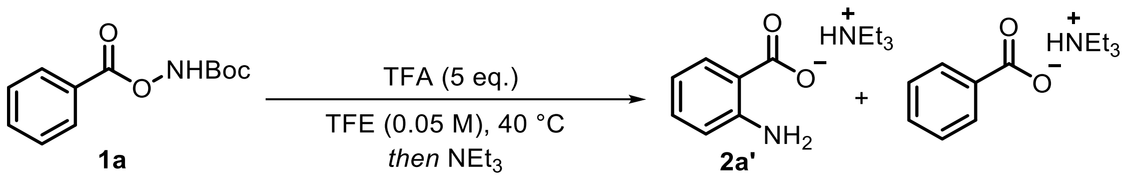

Entry[a]	Time (h)	Yield (2a')[b]	Yield (Benzoic acid)
1	24	64	21
2	16	68	19
3	8	57	20
4	4	19	42

[a] Reactions were performed on a 0.1 mmol scale. [b] NMR yields for each product were assessed by ^{1}H NMR using 1,2-dimethoxyethane as an internal standard.

*Table S8. The effect of catalytic Fe(II) on the rearrangement of **1a**.*

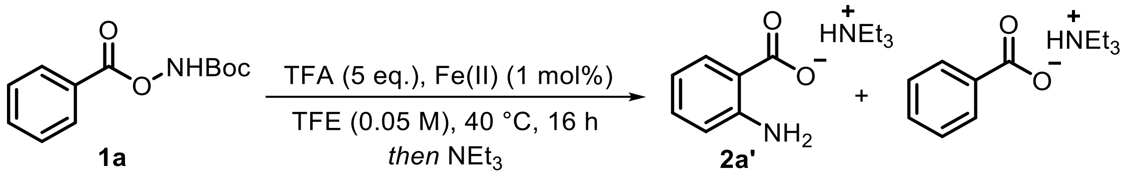

Entry[a]	Fe(II) source	Yield (2a')[b]	Yield (Benzoic acid)
1	$FeBr_2$	72	14
2	$FeCl_2$	72	16
3	$FeSO_4$	72	12
4	$Fe(OAc)_2$	72	12
5	$Fe(acac)_2$	72	17
6	Ferrocene	50	22

[a] Reactions were performed on a 0.1 mmol scale. [b] NMR yields for each product were assessed by ^{1}H NMR using 1,2-dimethoxyethane as an internal standard.

4 Comparison of Fe and Fe-free Protocol

As part of the scope, we evaluated a number of substrates with and without iron catalyst to evaluate the effect more broadly. In some cases, the yields were similar without iron but in other cases they were much lower and the iron-catalysed reactions were found to be more reproducible. A representative comparison is shown below; all reactions were analysed by crude ^{1}H NMR using 1,2-DME as an internal standard. For reproducibility and for broader applicability, we conducted all our scope entries with the inclusion of the iron catalyst.

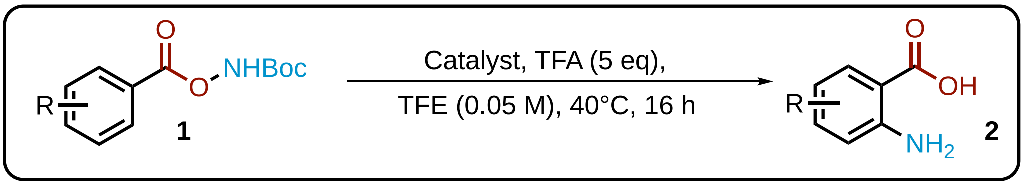

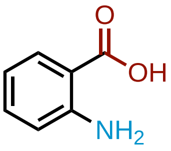

2a

No Catalyst: 68%
1 mol% $FeSO_4$: 72%

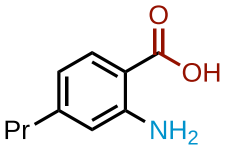

2c

No Catalyst: Trace
1 mol% $FeSO_4$: 62%

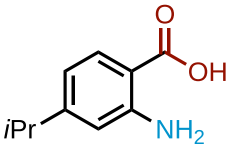

2d

No Catalyst: Trace
1 mol% $FeSO_4$: 50%

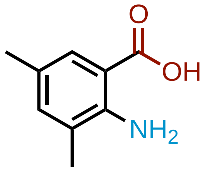

2f

No Catalyst: Complex mixture
1 mol% $FeSO_4$: 72%

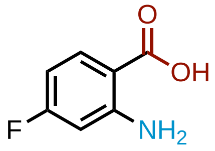

2j

No Catalyst: 33%
1 mol% $FeSO_4$: 44%

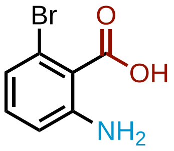

2s

No Catalyst: 56%
1 mol% $FeSO_4$: 60%

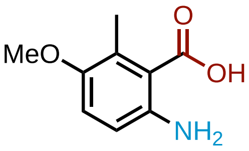

2aa

No Catalyst: 35%
1 mol% $FeSO_4$: 53%

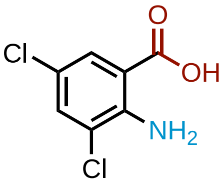

2am

No Catalyst: 20%
1 mol% $FeSO_4$: 39%

5 Unsuccessful Substrates

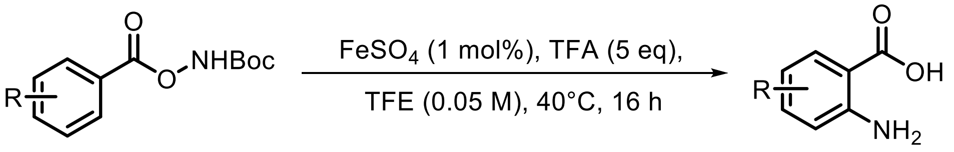

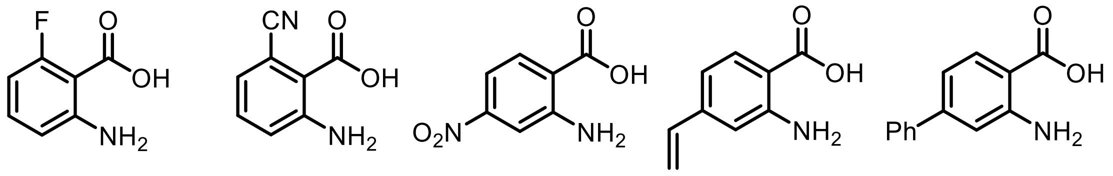

complex mixture   complex mixture   Trace   0%   complex mixture

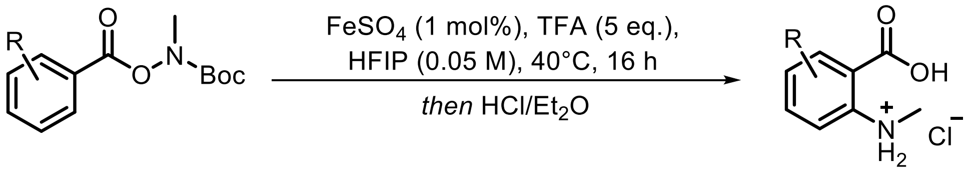

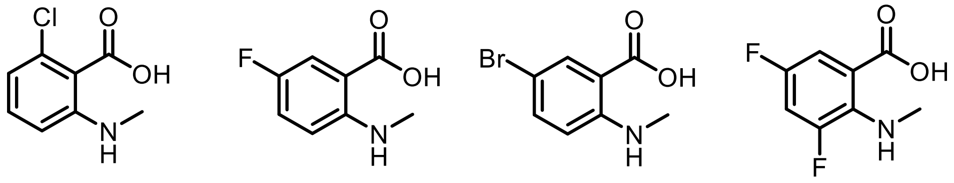

0%   Trace   Trace   0%

6 Substrate Synthesis

***tert*-Butyl (benzoyloxy)carbamate 1a**

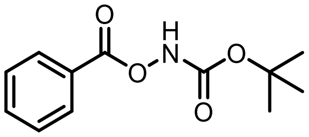

Under an atmosphere of nitrogen, triethylamine (5.57 g, 55.0 mmol, 1.1 eq.) was added to a solution of benzoyl chloride (7.03 g, 50.0 mmol, 1 eq.) and *tert*-butyl *N*-hydroxycarbamate (7.32 g, 55.0 mmol, 1 eq.) in CH_2Cl_2 (100 mL) at 0 ℃. The reaction was allowed to warm to room temperature and was stirred for 16 h. The reaction was then quenched with H_2O (100 mL) and the aqueous layer was then extracted with CH_2Cl_2 (3 x 100 mL). The combined organics were then dried ($MgSO_4$), filtered and concentrated *in vacuo.* The crude residue was then purified by silica gel chromatography (eluent: Pet Ether: EtOAc 100:0 – 90:10) to give the title compound (9.16 g, 38.6 mmol, 77%) as a white solid.

^{1}H NMR (400 MHz, $CDCl_3$) δ 8.29 (br s, 1H), 8.08 (d, *J* = 7.8 Hz, 2H), 7.59 (t, *J* = 7.4 Hz, 1H), 7.45 (t, *J* = 7.7 Hz, 2H), 1.50 (s, 9H); ^{13}C NMR (101 MHz, $CDCl_3$) δ 166.1, 155.6, 134.1, 129.9, 128.6, 127.0, 83.3, 28.0.

Data matches literature values.[1]

***tert*-Butyl ((4-methylbenzoyl)oxy)carbamate 1b**

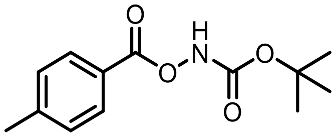

Under an atmosphere of nitrogen, triethylamine (1.11 g, 11.0 mmol, 1.1 eq.) was added to a solution of 4-methylbenzoyl chloride (1.54 g, 10.0 mmol, 1 eq.) and *tert*-butyl *N*-hydroxycarbamate (1.33 g, 10.0 mmol, 1 eq.) in CH_2Cl_2 (20 mL) at 0 ℃. The reaction was allowed to warm to room temperature and was stirred for 16 h. The reaction was quenched with H_2O (100 mL) and the aqueous layer was then extracted with CH_2Cl_2 (3 x 100 mL). The combined organics were then dried ($MgSO_4$), filtered and concentrated *in vacuo.* The crude residue was then purified by silica gel chromatography (eluent: Pet Ether: EtOAc 98:2 – 94:6) to give the title compound (2.39 g, 9.50 mmol, 95%) as a white solid.

^{1}H NMR (400 MHz, $CDCl_3$) δ 8.29 (br s, 1H), 7.99 (d, *J* = 8.1 Hz, 2H), 7.28 (d, *J* = 8.1 Hz, 2H), 2.43 (s, 3H), 1.52 (s, 9H); ^{13}C NMR (101 MHz, $CDCl_3$) δ 166.1, 155.7, 145.0, 129.9, 129.4, 124.1, 83.2, 28.1, 21.8; HRMS (ESI) calcd for $[C_{13}H_{17}NO_4 + Na]^+$: 274.1050. Found: 274.1046.

tert-Butyl ((4-propylbenzoyl)oxy)carbamate 1c

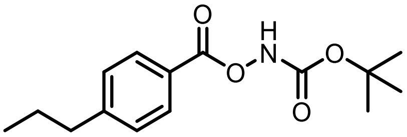

4-Propylbenzoic acid (822 mg, 4.99 mmol, 1 eq.), *tert*-butyl *N*-hydroxycarbamate (665 mg, 5.00 mmol, 1 eq.) and DCC (1.14 g, 5.53 mmol, 1.1 eq.) were subject to general procedure A. The crude residue was purified by silica gel chromatography (eluent: Pet Ether:EtOAc 95:5 – 92:8) to give the title compound (905 mg, 3.24 mmol, 65%) as a white solid.

^{1}H NMR (400 MHz, $CDCl_3$) δ 8.26 (br s, 1H), 8.02 (d, *J* = 7.9 Hz, 2H), 7.29 (d, *J* = 7.9 Hz, 2H), 2.67 (t, *J* = 7.6 Hz, 2H), 1.68 (h, *J* = 7.4 Hz, 2H), 1.53 (s, 9H), 0.95 (t, *J* = 7.3 Hz, 3H); ^{13}C NMR (101 MHz, $CDCl_3$) δ 166.2, 155.7, 149.7, 130.0, 128.8, 124.3, 83.2, 38.1, 28.1, 24.2, 13.7; HRMS (ESI) calcd for $[C_{15}H_{21}NO_4 + Na]^+$: 302.1363. Found: 302.1361.

Alternative approach: Under an atmosphere of nitrogen, triethylamine (304 mg, 3.01 mmol, 1 eq.) was added to a solution of 4-propylbenzoyl chloride (549 mg, 3.00 mmol, 1 eq.) and *tert*-butyl *N*-hydroxycarbamate (399 mg, 3.00 mmol, 1 eq.) in CH_2Cl_2 (6 mL) at 0 ℃. The reaction was allowed to warm to room temperature and was stirred for 16 h. The reaction was quenched with H_2O (100 mL) and the aqueous layer was then extracted with CH_2Cl_2 (3 x 100 mL). The combined organics were then dried ($MgSO_4$), filtered and concentrated *in vacuo.* The crude residue was then purified by silica gel chromatography (eluent: Pet Ether: EtOAc (98:2 – 94:6) to give the title compound (459 mg, 1.65 mmol, 55%) as a white solid.

Data consistent with the title compound being synthesised via general procedure A.

tert-Butyl ((4-isopropylbenzoyl)oxy)carbamate 1d

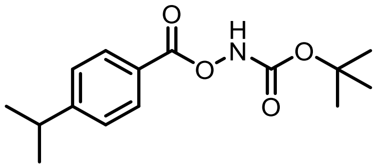

4-Isopropylbenzoic acid (1.64 g, 9.99 mmol, 1 eq.), *tert*-butyl *N*-hydroxycarbamate (1.33 g, 10.0 mmol, 1 eq.) and DCC (2.17 g, 10.5 mmol, 1 eq.) were subject to general procedure A. The crude residue was purified by silica gel chromatography (eluent: Pet Ether:EtOAc 95:5 – 90:10) to give the title compound (2.16 g, 7.72 mmol, 77%) as a white solid.

^{1}H NMR (400 MHz, $CDCl_3$) δ 8.31 (s, 1H), 8.03 (d, *J* = 8.4 Hz, 2H), 7.33 (d, *J* = 8.4 Hz, 2H), 2.98 (hept, *J* = 6.9 Hz, 1H), 1.52 (s, 9H), 1.27 (d, *J* = 7.0 Hz, 6H); ^{13}C NMR (101 MHz, $CDCl_3$) δ 166.1, 155.7, 130.1, 126.8, 126.8, 124.5, 83.2, 34.4, 28.0, 23.6; HRMS (ESI) calcd for $[C_{15}H_{21}NO_4 + Na]^+$: 302.1363. Found: 302.1366.

***tert*-Butyl ((3-methylbenzoyl)oxy)carbamate 1e**

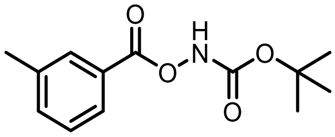

3-Methylbenzoic acid (1.36 g, 10.0 mmol, 1 eq.), *tert*-butyl *N*-hydroxycarbamate (1.33 g, 10.0 mmol, 1 eq.) and DCC (2.17 g, 10.5 mmol, 1.05 eq.) were subject to general procedure A. The crude residue was purified by silica gel chromatography (eluent: Pet Ether:EtOAc 93:7) to give the title compound (1.76 g, 6.91 mmol, 69%) as a white solid.

^{1}H NMR (400 MHz, $CDCl_3$) δ 8.22 (br s, 1H), 7.94 – 7.82 (m, 2H), 7.42 (d, *J* = 7.7 Hz, 1H), 7.35 (t, *J* = 7.6 Hz, 1H), 2.40 (s, 3H), 1.51 (s, 9H); ^{13}C NMR (101 MHz, $CDCl_3$) δ 166.3, 155.6, 138.6, 134.9, 130.4, 128.5, 127.1, 126.8, 83.3, 28.1, 21.2; HRMS (ESI) calcd for $[C_{13}H_{17}NO_4 + Na]^+$: 274.1050. Found: 274.1052.

***tert*-Butyl ((3,5-dimethylbenzoyl)oxy)carbamate 1f**

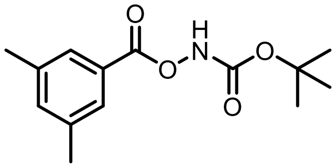

3,5-Dimethylbenzoic acid (1.50 g, 10.0 mmol, 1 eq.), *tert*-butyl *N*-hydroxycarbamate (1.33 g, 10.0 mmol, 1 eq.) and DCC (2.27 g, 10.5 mmol, 1.05 eq.) were subject to general procedure A. The crude residue was purified by silica gel chromatography (eluent: Pet Ether:EtOAc 95:5) to give the title compound (2.07 g, 7.80 mmol, 78%) as a white solid.

^{1}H NMR (400 MHz, $CDCl_3$) δ 8.34 (s, 1H), 7.73 (s, 2H), 2.38 (s, 6H), 1.54 (s, 9H); ^{13}C NMR (101 MHz, $CDCl_3$) δ 166.4, 155.7, 138.4, 135.8, 127.6, 126.7, 83.2, 28.0, 21.1; HRMS (ESI) calcd for $[C_{14}H_{19}NO_4 + Na]^+$: 288.1206. Found: 288.1199.

tert-Butyl ((3-isopropylbenzoyl)oxy)carbamate 1g

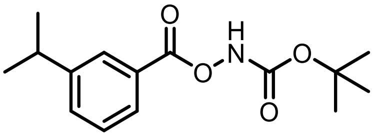

3-Isopropylbenzoic acid (821 mg, 5.00 mmol, 1 eq.), *tert*-butyl *N*-hydroxycarbamate (665 mg, 5.00 mmol) and DCC (1.09 g, 5.28 mmol, 1.05 eq.) were subject to general procedure A. The crude residue was purified by silica gel chromatography (eluent: Pet Ether:EtOAc 97:3 – 92:8) to give the title compound (748 mg, 2.68 mmol, 54%) as a colourless oil.

^{1}H NMR (400 MHz, $CDCl_3$) δ 8.20 (br s, 1H), 7.95 (s, 1H), 7.91 (dt, *J* = 7.7, 1.5 Hz, 1H), 7.48 (dt, *J* = 7.8, 1.6 Hz, 1H), 7.39 (t, *J* = 7.7 Hz, 1H), 2.97 (h, *J* = 6.9 Hz, 1H), 1.51 (s, 9H), 1.27 (d, *J* = 6.9 Hz, 6H); ^{13}C NMR (101 MHz, $CDCl_3$) δ 166.4, 155.6, 149.5, 132.4, 128.7, 127.9, 127.5, 126.9, 83.3, 34.0, 28.1, 23.8; HRMS (ESI) calcd for $[C_{15}H_{21}NO_4 + Na]^+$: 302.1363. Found: 302.1360.

tert-Butyl ((3,4-dimethylbenzoyl)oxy)carbamate 1h

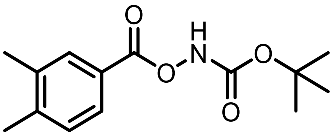

3,4-Dimethylbenzoic acid (1.50 g, 10.0 mmol, 1 eq.), *tert*-butyl *N*-hydroxycarbamate (1.33 g, 10.0 , 1 eq.) and DCC (2.17 g, 10.5 mmol, 1.05 eq.) were subject to general procedure A. The crude residue was purified by silica gel chromatography (eluent: Pet Ether:EtOAc 95:5 – 92:8) to give the title compound (1.57 g, 5.92 mmol, 59%) as a yellow oil.

^{1}H NMR (400 MHz, $CDCl_3$) δ 8.23 (br s, 1H), 7.88 (s, 1H), 7.84 (d, *J* = 7.9 Hz, 1H), 7.24 (d, *J* = 7.9 Hz, 1H), 2.34 (s, 3H), 2.32 (s, 3H), 1.53 (s, 9H); ^{13}C NMR (101 MHz, $CDCl_3$) δ 166.3, 155.7, 143.8, 137.1, 130.8, 129.9, 127.5, 124.3, 83.2, 28.1, 20.1, 19.6; HRMS (ESI) calcd for $[C_{14}H_{19}NO_4 + Na]^+$: 288.1206. Found: 288.1209.

tert-Butyl ((3,5-di-tert-butylbenzoyl)oxy)carbamate 1i

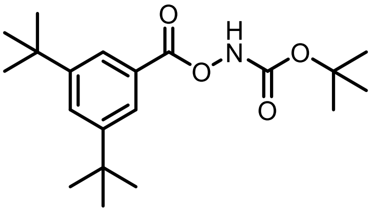

3,5-Di-*tert*-butylbenzoic acid (937 mg, 4.00 mmol, 1 eq.), *tert*-butyl *N*-hydroxycarbamate (532 mg, 4.00 mmol, 1 eq.) and DCC (868 mg, 4.21 mmol, 1.05 eq.) were subject to general procedure A. The crude residue was

purified by silica gel chromatography (eluent: Pet Ether:EtOAc 100:0 – 95:5) to give the title compound (1.25 g, 3.58 mmol, 89%) as a yellow oil.

^{1}H NMR (400 MHz, $CDCl_3$) δ 8.20 (br s, 1H), 7.94 (d, *J* = 1.8 Hz, 2H), 7.69 (t, *J* = 1.9 Hz, 1H), 1.52 (s, 9H), 1.35 (s, 18H); ^{13}C NMR (101 MHz, $CDCl_3$) δ 166.9, 155.7, 151.4, 128.4, 126.3, 124.1, 83.2, 35.0, 31.3, 28.1; HRMS (ESI) calcd for $[C_{20}H_{31}NO_4 + Na]^+$: 372.2145. Found: 372.2146.

***tert*-Butyl ((4-fluorobenzoyl)oxy)carbamate 1j**

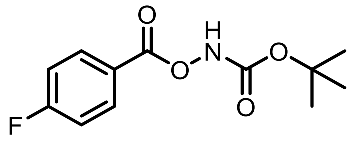

4-Fluorobenzoic acid (1.40 g, 10.0 mmol, 1 eq.), *tert*-butyl *N*-hydroxycarbamate (1.33 g, 10.0 mmol, 1 eq.) and DCC (2.17 g, 10.5 mmol, 1 eq.) were subject to general procedure A. The crude residue was purified by silica gel chromatography (eluent: Pet Ether:EtOAc 92:8) to give the title compound (2.00 g, 7.82 mmol, 78%) as a white solid.

^{1}H NMR (500 MHz, $CDCl_3$) δ 8.21 (br s, 1H), 8.16 – 8.05 (m, 2H), 7.18 – 7.10 (m, 2H), 1.50 (s, 9H); ^{13}C NMR (126 MHz, $CDCl_3$) δ 166.4 (d, *J* = 256.1 Hz), 165.1, 155.5, 132.6 (d, *J* = 9.5 Hz), 123.2 (d, *J* = 3.0 Hz), 116.0 (d, *J* = 22.2 Hz), 83.5, 28.0; ^{19}F NMR (471 MHz, $CDCl_3$) δ -103.05; HRMS (ESI) calcd for $[C_{12}H_{14}FNO_4 + Na]^+$: 278.0799. Found: 278.0790.

***tert*-Butyl ((4-chlorobenzoyl)oxy)carbamate 1m**

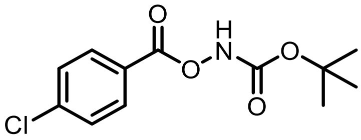

4-Chlorobenzoic acid (1.57 g, 10.0 mmol, 1 eq.), *tert*-butyl *N*-hydroxycarbamate (1.33 g, 10.0 mmol, 1 eq.) and DCC (2.17 g, 10.5 mmol, 1 eq.) were subject to general procedure A. The crude residue was purified by silica gel chromatography (eluent: Pet Ether:EtOAc 95:5 – 90:10) to give the title compound (2.21 g, 8.15 mmol, 81%) as a white solid.

^{1}H NMR (400 MHz, $CDCl_3$) δ 8.33 (br s, 1H), 8.02 (d, *J* = 8.6 Hz, 2H), 7.45 (d, *J* = 8.6 Hz, 2H), 1.51 (s, 9H); ^{13}C NMR (101 MHz, $CDCl_3$) δ 165.2, 155.5, 140.7, 131.2, 129.1, 125.4, 83.5, 28.0; HRMS (ESI) calcd for $[C_{12}H_{14}ClNO_4 + Na]^+$: 294.0504. Found: 294.0490.

tert-Butyl ((3-fluorobenzoyl)oxy)carbamate 1k

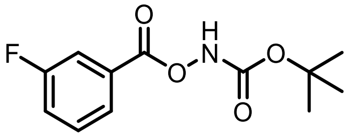

3-Fluorobenzoic acid (1.40 g, 10.0 mmol, 1 eq.), *tert*-butyl *N*-hydroxycarbamate (1.33 g, 10.0 mmol, 1 eq.) and DCC (2.27 g, 10.5 mmol, 1.05 eq.) were subject to general procedure A. The crude residue was purified by silica gel chromatography (eluent: Pet Ether:EtOAc 93:7) to give the title compound (1.37 g, 5.36 mmol, 54%) as a white solid.

^{1}H NMR (500 MHz, $CDCl_3$) δ 8.21 (br s, 1H), 7.92 (dt, *J* = 7.8, 1.3 Hz, 1H), 7.83 – 7.76 (m, 1H), 7.49 (td, *J* = 8.1, 5.5 Hz, 1H), 7.35 (tdd, *J* = 8.3, 2.7, 1.0 Hz, 1H), 1.54 (s, 9H); ^{13}C NMR (126 MHz, $CDCl_3$) δ 165.0 (d, *J* = 3.1 Hz), 162.5 (d, *J* = 248.3 Hz), 155.4, 130.4 (d, *J* = 7.7 Hz), 129.0 (d, *J* = 7.8 Hz), 125.7 (d, *J* = 3.2 Hz), 121.3 (d, *J* = 21.3 Hz), 116.8 (d, *J* = 23.5 Hz), 83.6, 28.0; ^{19}F NMR (471 MHz, $CDCl_3$) δ -111.38; HRMS (ESI) calcd for $[C_{12}H_{14}FNO_4 + Na]^+$: 278.0799. Found: 278.0809.

tert-Butyl ((3-fluoro-2-methylbenzoyl)oxy)carbamate 1l

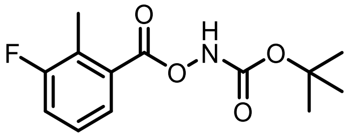

3-Fluoro-2-methylbenzoic acid (1.54 g, 10.0 mmol, 1 eq.), *tert*-butyl *N*-hydroxycarbamate (1.33 g, 10.0 mmol, 1 eq.) and DCC (2.17 g, 10.5 mmol, 1.05 eq.) were subject to general procedure A. The crude residue was purified by silica gel chromatography (eluent: Pet Ether:EtOAc 95:5 – 92:8) to give the title compound (1.92 g, 7.15 mmol, 72%) as a white solid.

^{1}H NMR (400 MHz, $CDCl_3$) δ 8.29 (br s, 1H), 7.85 – 7.67 (m, 1H), 7.24 – 7.14 (m, 2H), 2.48 (d, *J* = 2.4 Hz, 3H), 1.50 (s, 9H);^{13}C NMR (101 MHz, $CDCl_3$) δ 166.3 (d, *J* = 3.7 Hz), 161.3 (d, *J* = 244.9 Hz), 155.6, 128.7 (d, *J* = 4.6 Hz), 127.8 (d, *J* = 18.3 Hz), 126.9 (d, *J* = 8.7 Hz), 126.4 (d, *J* = 3.6 Hz), 119.8 (d, *J* = 23.6 Hz), 83.4, 28.0, 11.8 (d, *J* = 6.0 Hz); ^{19}F NMR (376 MHz, $CDCl_3$) δ -115.63; HRMS (ESI) calcd for $[C_{13}H_{16}FNO_4 + Na]^+$: 292.0956. Found: 292.0955.

tert-Butyl ((2-chloro-3-methylbenzoyl)oxy)carbamate 1n

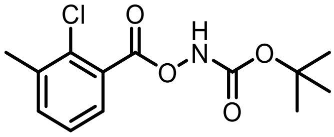

2-Chloro-3-methylbenzoic acid (1.71 g, 10.0 mmol, 1 eq.), *tert*-butyl *N*-hydroxycarbamate (1.33 g, 10.0 mmol, 1 eq.) and DCC (2.27 g, 10.5 mmol, 1.05 eq.) were subject to general procedure A. The crude residue was purified by silica gel chromatography (eluent: Pet Ether:EtOAc 95:5 – 92:8) to give the title compound (1.53 g, 5.36 mmol, 54%) as a white solid.

^{1}H NMR (400 MHz, $CDCl_3$) δ 8.15 (br s, 1H), 7.77 (d, *J* = 7.0 Hz, 1H), 7.46 (d, *J* = 6.8 Hz, 1H), 7.27 (t, *J* = 7.7 Hz, 1H), 2.46 (s, 3H), 1.55 (s, 9H); ^{13}C NMR (101 MHz, $CDCl_3$) δ 165.7, 155.5, 138.3, 134.9, 133.8, 129.2, 127.6, 126.3, 83.5, 28.1, 20.6; HRMS (ESI) calcd for $[C_{13}H_{16}ClNO_4 + Na]^+$: 308.0660. Found: 308.0660.

tert-Butyl ((2-chlorobenzoyl)oxy)carbamate 1o

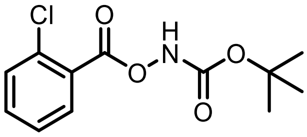

2-Chlorobenzoic acid (1.57 g, 10.0 mmol, 1 eq.), *tert*-butyl *N*-hydroxycarbamate (1.33 g, 10.0 mmol, 1 eq.) and DCC (2.27 g, 10.5 mmol, 1.05 eq.) were subject to general procedure A**.** The crude residue was purified by silica gel chromatography (eluent: Pet Ether:EtOAc 94:6 – 92:8) to give the title compound (2.15 g, 7.91 mmol, 79%) as a white solid.

^{1}H NMR (400 MHz, $CDCl_3$) δ 8.18 (br s, 1H), 7.99 (d, *J* = 7.8 Hz, 1H), 7.57 – 7.46 (m, 2H), 7.44 – 7.32 (m, 1H), 1.54 (s, 9H); ^{13}C NMR (101 MHz, $CDCl_3$) δ 165.0, 155.5, 134.5, 133.7, 131.9, 131.2, 126.8, 126.8, 83.6, 28.1; HRMS (ESI) calcd for $[C_{12}H_{14}ClNO_4 + Na]^+$: 294.0504. Found: 294.0503.

tert-Butyl ((3-chlorobenzoyl)oxy)carbamate 1p

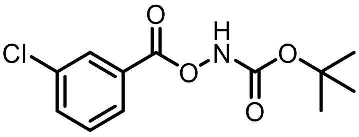

3-Chlorobenzoic acid (1.57 g, 10.0 mmol, 1 eq.), *tert*-butyl *N*-hydroxycarbamate (1.33 g, 10.0 mmol, 1 eq.) and DCC (2.27 g, 10.5 mmol, 1.05 eq.) were subject to general procedure A. The crude residue was purified by silica gel chromatography (eluent: Pet Ether:EtOAc 93:7) to give the title compound (1.68 g, 6.18 mmol, 62%) as a white solid.

^{1}H NMR (400 MHz, $CDCl_3$) δ 8.32 (br s, 1H), 8.07 (t, *J* = 1.9 Hz, 1H), 7.98 (dt, *J* = 7.7, 1.3 Hz, 1H), 7.60 (ddd, *J* = 8.1, 2.2, 1.1 Hz, 1H), 7.43 (t, *J* = 7.9 Hz, 1H), 1.52 (s, 9H); ^{13}C NMR (101 MHz, $CDCl_3$) δ 164.9, 155.4, 134.9, 134.1, 130.0, 129.9, 128.7, 128.0, 83.6, 28.0; HRMS (ESI) calcd for $[C_{12}H_{14}ClNO_4 + Na]^+$: 294.0504. Found: 294.0515.

***tert*-Butyl ((3-chloro-2-methylbenzoyl)oxy)carbamate 1q**

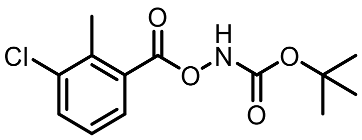

3-Chloro-2-methylbenzoic acid (1.71 g, 10.0 mmol, 1 eq.), *tert*-butyl *N*-hydroxycarbamate (1.33 g, 10.0 mmol, 1 eq.) and DCC (2.17 g, 10.5 mmol, 1.05 eq.) were subject to general procedure A. The crude residue was purified by silica gel chromatography (eluent: Pet Ether:EtOAc 95:5 – 90:10) to give the title compound (2.15 g, 7.52 mmol, 75%) as a white solid.

^{1}H NMR (400 MHz, $CDCl_3$) δ 8.17 (br s, 1H), 7.84 (dd, *J* = 7.8, 1.3 Hz, 1H), 7.58 (dd, *J* = 8.0, 1.3 Hz, 1H), 7.23 (t, *J* = 7.9 Hz, 1H), 2.64 (s, 3H), 1.54 (s, 9H); ^{13}C NMR (101 MHz, $CDCl_3$) δ 166.9, 155.6, 137.9, 136.2, 133.6, 129.1, 129.0, 126.7, 83.5, 28.1, 17.4; HRMS (ESI) calcd for $[C_{13}H_{16}ClNO_4 + Na]^+$: 308.0660. Found: 308.0653.

***tert*-Butyl ((4-bromobenzoyl)oxy)carbamate 1r**

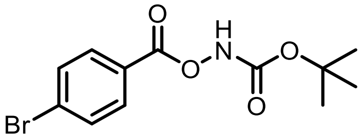

4-Bromobenzoic acid (2.01 g, 10.0 mmol, 1 eq.), *tert*-butyl *N*-hydroxycarbamate (1.33 g, 10.0 mmol, 1 eq.) and DCC (2.17 g, 10.5 mmol, 1 eq.) were subject to general procedure A. The crude residue was purified by silica gel chromatography (eluent: Pet Ether:EtOAc 95:5 – 90:10) to give the title compound (1.89 g, 5.99 mmol, 60%) as a white solid.

^{1}H NMR (400 MHz, $CDCl_3$) δ 8.23 (br s, 1H), 7.93 (d, *J* = 8.5 Hz, 2H), 7.61 (d, *J* = 8.5 Hz, 2H), 1.50 (s, 9H); ^{13}C NMR (101 MHz, $CDCl_3$) δ 165.4, 155.4, 132.1, 131.3, 129.5, 125.8, 83.5, 28.0; HRMS (ESI) calcd for $[C_{12}H_{14}BrNO_4 + Na]^+$: 337.9998. Found: 338.0000.

tert-Butyl ((2-bromobenzoyl)oxy)carbamate 1s

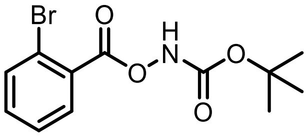

2-Bromobenzoic acid (2.01 g, 10.0 mmol, 1 eq.), *tert*-butyl *N*-hydroxycarbamate (1.33 g, 10.0 mmol, 1 eq.) and DCC (2.27 g, 10.5 mmol, 1.05 eq.) were subject to general procedure A. The crude residue was purified by silica gel chromatography (eluent: Pet Ether:EtOAc 95:5) to give the title compound (2.19 g, 6.93 mmol, 69%) as a white solid.

^{1}H NMR (400 MHz, $CDCl_3$) δ 8.22 (br s, 1H), 7.98 – 7.87 (m, 1H), 7.73 – 7.62 (m, 1H), 7.45 – 7.34 (m, 2H), 1.50 (s, 9H); ^{13}C NMR (101 MHz, $CDCl_3$) δ 165.5, 155.5, 134.5, 133.7, 131.9, 128.8, 127.3, 122.3, 83.6, 28.1; HRMS (ESI) calcd for $[C_{12}H_{14}BrNO_4 + Na]^+$: 337.9998. Found: 338.0006.

Alternative approach: Under an atmosphere of nitrogen, triethylamine (508 mg, 5.00 mmol, 1 eq.) was added to a solution of 2-bromobenzoyl chloride (1.10 g, 5.00 mmol, 1 eq.) and *tert*-butyl *N*-hydroxycarbamate (665 mg, 5.00 mmol, 1 eq.) in CH_2Cl_2 (10 mL) at 0 ℃. The reaction was allowed to warm to room temperature and was stirred for 16 h. The reaction was then quenched with H_2O (100 mL) and the aqueous layer was then extracted with CH_2Cl_2 (3 x 100 mL). The combined organics were then dried ($MgSO_4$), filtered and concentrated *in vacuo.* The crude residue was then purified by silica gel chromatography (eluent: Pet Ether: EtOAc (98:2 – 94:6) to give the title compound (1.30 g, 4.12 mmol, 82%) as a white solid.

Data consistent with the title compound being synthesised via general procedure A.

tert-Butyl ((2-bromo-3-methylbenzoyl)oxy)carbamate 1t

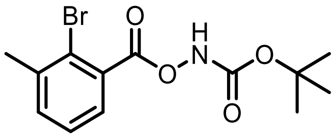

2-Bromo-3-methylbenzoic acid (2.15 g, 10.0 mmol, 1 eq.), *tert*-butyl *N*-hydroxycarbamate (1.33 g, 10.0 mmol, 1 eq.) and DCC (2.27 g, 10.5 mmol, 1.05 eq.) were subject to general procedure A. The crude residue was purified by silica gel chromatography (eluent: Pet Ether:EtOAc 94:6) to give the title compound (2.59 g, 7.86 mmol, 79%) as a white solid.

^{1}H NMR (500 MHz, $CDCl_3$) δ 8.13 (br s, 1H), 7.69 (dd, *J* = 7.7, 1.3 Hz, 1H), 7.44 (dd, *J* = 7.7, 0.9 Hz, 1H), 7.31 (t, *J* = 7.7 Hz, 1H), 2.49 (s, 3H), 1.54 (s, 9H); ^{13}C NMR (126 MHz, $CDCl_3$) δ 166.4, 155.5, 140.2, 134.4, 130.2, 129.0, 127.0, 123.9, 83.6, 28.1, 23.8; HRMS (ESI) calcd for $[C_{13}H_{16}BrNO_4 + Na]^+$: 352.0155. Found: 352.0162.

tert-Butyl ((3-bromobenzoyl)oxy)carbamate 1u

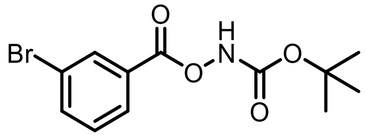

3-Bromobenzoic acid (2.01 g, 10.0 mmol, 1 eq.), *tert*-butyl *N*-hydroxycarbamate (1.33 g, 10.0 mmol, 1 eq.) and DCC (2.27 g, 10.5 mmol, 1.05 eq.) were subject to general procedure A. The crude residue was purified by silica gel chromatography (eluent: Pet Ether:EtOAc 93:7) to give the title compound (1.71 g, 5.41 mmol, 54%) as a white solid.

^{1}H NMR (400 MHz, $CDCl_3$) δ 8.34 (br s, 1H), 8.21 (t, *J* = 1.7 Hz, 1H), 8.01 (d, *J* = 7.9 Hz, 1H), 7.74 (d, *J* = 8.1 Hz, 1H), 7.35 (t, *J* = 7.9 Hz, 1H), 1.51 (s, 9H); ^{13}C NMR (101 MHz, $CDCl_3$) δ 164.8, 155.4, 137.0, 132.8, 130.2, 128.8, 128.4, 122.7, 83.6, 28.0; HRMS (ESI) calcd for $[C_{12}H_{14}BrNO_4 + Na]^+$: 337.9998. Found: 338.0007.

tert-Butyl ((3-iodobenzoyl)oxy)carbamate 1v

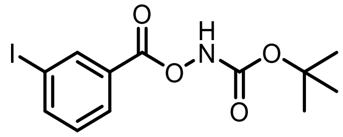

3-Iodobenzoic acid (2.48 g, 10.0 mmol, 1 eq.), *tert*-butyl *N*-hydroxycarbamate (1.33 g, 10.0 mmol, 1 eq.) and DCC (2.17 g, 10.5 mmol, 1 eq.) were subject to general procedure A. The crude residue was purified by silica gel chromatography (eluent: Pet Ether:EtOAc 95:5 – 90:10) to give the title compound (2.91 g, 8.01 mmol, 80%) as a yellow solid.

^{1}H NMR (400 MHz, $CDCl_3$) δ 8.41 (t, *J* = 1.7 Hz, 1H), 8.19 (br s, 1H), 8.05 (dt, *J* = 7.9, 1.3 Hz, 1H), 7.98 – 7.89 (m, 1H), 7.21 (t, *J* = 7.9 Hz, 1H), 1.51 (s, 9H); ^{13}C NMR (101 MHz, $CDCl_3$) δ 164.7, 155.4, 142.9, 138.6, 130.3, 129.0, 128.8, 93.9, 83.6, 28.01. HRMS (ESI) calcd for $[C_{12}H_{14}INO_4 + Na]^+$: 385.9860. Found: 385.9865.

tert-Butyl ((2,3-dichlorobenzoyl)oxy)carbamate 1w

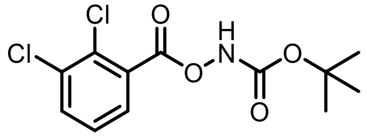

2,3-Dichlorobenzoic acid (1.91 g, 10.0 mmol, 1 eq.), *tert*-butyl *N*-hydroxycarbamate (1.33 g, 10.0 mmol, 1 eq.) and DCC (2.27 g, 10.5 mmol, 1.05 eq.) were subject to general procedure A. The crude residue was purified by silica gel chromatography (eluent: Pet Ether:EtOAc 93:7 – 90:10) to give the title compound (1.78 g, 5.82 mmol, 58%) as a white solid.

^{1}H NMR (400 MHz, $CDCl_3$) δ 8.21 (br s, 1H), 7.83 (d, *J* = 7.7 Hz, 1H), 7.67 (d, *J* = 8.0 Hz, 1H), 7.32 (t, *J* = 7.9 Hz, 1H), 1.54 (s, 9H); ^{13}C NMR (101 MHz, $CDCl_3$) δ 164.6, 155.4, 134.9, 134.3, 132.4, 129.7, 129.4, 127.3, 83.8, 28.0; HRMS (ESI) calcd for $[C_{12}H_{13}Cl_2NO_4 + Na]^+$: 328.0114. Found: 328.0099.

***tert*-Butyl ((3,5-dibromobenzoyl)oxy)carbamate 1x**

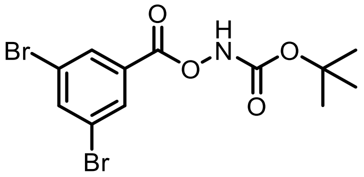

3,5-Dibromobenzoic acid (2.80 g, 10.0 mmol, 1 eq.), *tert*-butyl *N*-hydroxycarbamate (1.33 g, 10.0 mmol, 1 eq.) and DCC (2.17 g, 10.5 mmol, 1.05 eq.) were subject to general procedure A. The crude residue was purified by silica gel chromatography (eluent: Pet Ether:EtOAc 95:5 – 92:8) to give the title compound (3.37 g, 8.54 mmol, 85%) as a white solid.

^{1}H NMR (400 MHz, $CDCl_3$) δ 8.25 (br s, 1H), 8.13 (d, *J* = 1.8 Hz, 2H), 7.89 (t, *J* = 1.8 Hz, 1H), 1.50 (s, 9H); ^{13}C NMR (101 MHz, $CDCl_3$) δ 163.7, 155.2, 139.4, 131.5, 130.1, 123.3, 83.9, 28.0; HRMS (ESI) calcd for $[C_{12}H_{13}Br_2NO_4 + Na]^+$: 415.9104. Found: 415.9105.

***tert*-Butyl ((3,5-difluorobenzoyl)oxy)carbamate 1y**

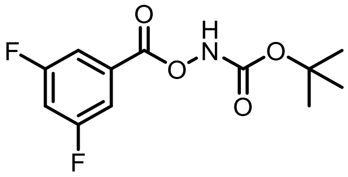

3,5-Difluorobenzoic acid (1.58 g, 10.0 mmol, 1 eq.), *tert*-butyl *N*-hydroxycarbamate (1.33 g, 10.0 mmol, 1 eq.) and DCC (2.27 g, 10.5 mmol, 1.05 mmol) were subject to general procedure A. The crude residue was purified by silica gel chromatography (eluent: Pet Ether:EtOAc 94:6) to give the title compound (2.16 g, 7.92 mmol, 79%) as a white solid.

^{1}H NMR (500 MHz, $CDCl_3$) δ 8.26 (br s, 1H), 7.73 – 7.49 (m, 2H), 7.07 (tt, *J* = 8.5, 2.4 Hz, 1H), 1.50 (s, 9H);^{13}C NMR (126 MHz, $CDCl_3$) δ 164.0 (t, *J* = 3.5 Hz), 162.8 (dd, *J* = 251.3, 11.9 Hz), 155.3, 130.0 (t, *J* = 9.5 Hz), 113.4 – 112.6 (m), 109.6 (t, *J* = 25.1 Hz), 83.8, 28.0; ^{19}F NMR (376 MHz, $CDCl_3$) δ -107.42. HRMS (ESI) calcd for $[C_{12}H_{13}F_2NO_4 + Na]^+$: 296.0705. Found: 296.0714.

tert-Butyl ((3-methoxybenzoyl)oxy)carbamate 1z

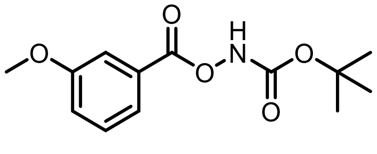

3-Methoxybenzoic acid (1.52 g, 10.0 mmol, 1 eq.), *tert*-butyl *N*-hydroxycarbamate (1.33 g, 10.0 mmol, 1 eq.) and DCC (2.27 g, 10.5 mmol, 1 eq.) were subject to general procedure A. The crude residue was purified by silica gel chromatography (eluent: Pet Ether:EtOAc 93:7) to give the title compound (1.82 g, 6.81 mmol, 68%) as a white solid.

^{1}H NMR (700 MHz, $CDCl_3$) δ 8.21 (br s, 1H), 7.72 (dt, *J* = 7.7, 1.3 Hz, 1H), 7.61 (dd, *J* = 2.7, 1.5 Hz, 1H), 7.40 (t, *J* = 8.0 Hz, 1H), 7.18 (ddd, *J* = 8.3, 2.7, 1.0 Hz, 1H), 3.87 (s, 3H), 1.54 (s, 9H); ^{13}C NMR (176 MHz, $CDCl_3$) δ 166.0, 159.7, 155.6, 129.7, 128.1, 122.4, 120.9, 114.0, 83.4, 55.5, 28.1; HRMS (ESI) calcd for $[C_{13}H_{17}NO_5 + Na]^+$: 290.0999. Found: 290.0992.

tert-Butyl ((3-methoxy-2-methylbenzoyl)oxy)carbamate 1aa

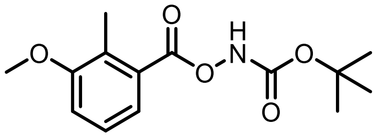

3-Methoxy-2-methylbenzoic acid (8.31 g, 50.0 mmol, 1 eq.), *tert*-butyl *N*-hydroxycarbamate (6.65 g, 50.0 mmol, 1 eq.) and DCC (11.4 g, 55.3 mmol, 1.1 eq.) were subject to general procedure A. The crude residue was purified by silica gel chromatography (eluent: Pet Ether:EtOAc 95:5 – 90:10) to give the title compound (12.3 g, 43.7 mmol, 87%) as a white solid.

^{1}H NMR (400 MHz, $CDCl_3$) δ 8.13 (br s, 1H), 7.51 (d, *J* = 7.8 Hz, 1H), 7.22 (t, *J* = 8.0 Hz, 1H), 7.03 (d, *J* = 8.2 Hz, 1H), 3.85 (s, 3H), 2.44 (s, 3H), 1.52 (s, 9H); ^{13}C NMR (101 MHz, $CDCl_3$) δ 167.5, 158.0, 155.7, 129.3, 128.1, 126.4, 122.3, 114.4, 83.3, 55.8, 28.1, 12.7; HRMS (ESI) calcd for $[C_{14}H_{19}NO_5 + Na]^+$: 304.1155. Found: 304.1149.

tert-Butyl ((2-bromo-3-methoxybenzoyl)oxy)carbamate 1ab

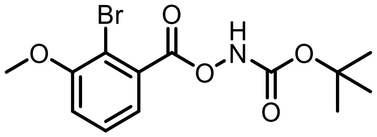

2-Bromo-3-methoxybenzoic acid (924 mg, 4.00 mmol, 1 eq.), *tert*-butyl *N*-hydroxycarbamate (532 mg, 4.00 mmol, 1 eq.) and DCC (868 mg, 4.21 mmol, 1.05 eq.) were subject to general procedure A. The crude residue was purified by silica gel chromatography (eluent: Pet Ether:EtOAc 95:5 – 88:12) to give the title compound (1.16 g, 3.35 mmol, 84%) as a white solid.

^{1}H NMR (400 MHz, $CDCl_3$) δ 8.09 (br s, 1H), 7.46 (dd, *J* = 7.8, 1.5 Hz, 1H), 7.36 (t, *J* = 8.0 Hz, 1H), 7.08 (dd, *J* = 8.2, 1.5 Hz, 1H), 3.93 (s, 3H), 1.52 (s, 9H); ^{13}C NMR (101 MHz, $CDCl_3$) δ 165.9, 156.7, 155.5, 131.1, 128.3, 123.2, 115.4, 111.9, 83.6, 56.7, 28.1; HRMS (ESI) calcd for $[C_{13}H_{16}BrNO_5 + Na]^+$: 368.0104. Found: 368.0101.

tert-Butyl ((3,5-dimethoxybenzoyl)oxy)carbamate 1ac

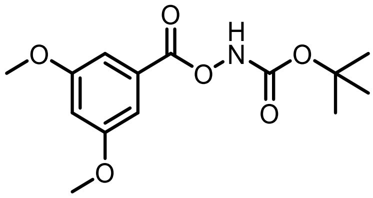

3,5-Dimethoxybenzoic acid (1.58 g, 10.0 mmol, 1 eq.), *tert*-butyl *N*-hydroxycarbamate (1.33 g, 10.0 mmol, 1 eq.) and DCC (2.27 g, 10.5 mmol, 1 eq.) were subject to general procedure A. The crude residue was purified by silica gel chromatography (eluent: Pet Ether:EtOAc 94:6) to give the title compound (2.16 g, 7.92 mmol, 79%) as a white solid.

^{1}H NMR (500 MHz, $CDCl_3$) δ 8.20 (br s, 1H), 7.24 (d, *J* = 2.4 Hz, 2H), 6.72 (t, *J* = 2.4 Hz, 1H), 3.85 (s, 6H), 1.53 (s, 9H); ^{13}C NMR (126 MHz, $CDCl_3$) δ 166.0, 160.8, 155.5, 128.6, 107.4, 107.1, 83.4, 55.7, 28.1; HRMS (ESI) calcd for $[C_{14}H_{19}NO_6 + Na]^+$: 320.1105. Found: 320.1106.

tert-Butyl ((2-trifluoromethylbenzoyl)oxy)carbamate 1ad

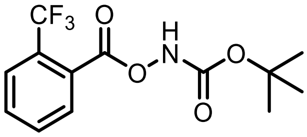

2-Trifluoromethylbenzoic acid (1.90 g, 10.0 mmol, 1 eq.), *tert*-butyl *N*-hydroxycarbamate (1.33 g, 10.0 mmol, 1 eq.) and DCC (2.27 g, 10.5 mmol, 1.05 eq.) were subject to general procedure A. The crude residue was purified by silica gel chromatography (eluent: Pet Ether:EtOAc 95:5 – 92:8) to give the title compound (2.55 g, 8.35 mmol, 84%) as a white solid.

^{1}H NMR (400 MHz, $CDCl_3$) δ 8.18 (br s, 1H), 8.04 – 7.95 (m, 1H), 7.85 – 7.79 (m, 1H), 7.76 – 7.61 (m, 2H), 1.54 (s, 9H); ^{13}C NMR (101 MHz, $CDCl_3$) δ 165.8, 155.4, 132.4, 131.9, 130.9, 129.4 (q, *J* = 33.0 Hz), 127.5 – 127.2 (m), 126.9 (q, *J* = 5.3 Hz), 123.0 (d, *J* = 273.6 Hz), 83.7, 28.0; ^{19}F NMR (376 MHz, $CDCl_3$) δ -60.55; HRMS (ESI) calcd for $[C_{13}H_{14}F_3NO_4 + Na]^+$: 328.0767. Found: 328.0762.

***tert*-Butyl ((4-(trifluoromethyl)benzoyl)oxy)carbamate 1ae**

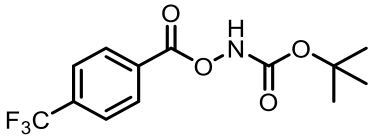

(4-Trifluoromethyl)benzoic acid (1.90 g, 10.0 mmol, 1 eq.), *tert*-butyl *N*-hydroxycarbamate (1.33 g, 10.0 mmol, 1 eq.) and DCC (2.17 g, 10.5 mmol, 1 eq.) were subject to general procedure A. The crude residue was purified by silica gel chromatography (eluent: Pet Ether:EtOAc 95:5 – 90:10) to give the title compound (2.07 g, 6.78 mmol, 68%) as a white solid.

^{1}H NMR (500 MHz, $CDCl_3$) δ 8.30 (s, 1H), 8.22 (d, *J* = 7.9 Hz, 2H), 7.76 (d, *J* = 8.2 Hz, 2H), 1.53 (s, 9H); ^{13}C NMR (126 MHz, $CDCl_3$) δ 164.9, 155.3, 135.5 (q, *J* = 32.9 Hz), 130.3, 130.3 (q, *J* = 1.3 Hz), 125.7 (q, *J* = 3.8 Hz), 123.4 (q, *J* = 272.9 Hz), 83.7, 28.0; ^{19}F NMR (471 MHz, $CDCl_3$) δ -63.36. HRMS (ESI) calcd for $[C_{13}H_{14}F_3NO_4 + Na]^+$: 328.0767. Found: 328.0763.

***tert*-Butyl ((4-(difluoromethoxy)benzoyl)oxy)carbamate 1af**

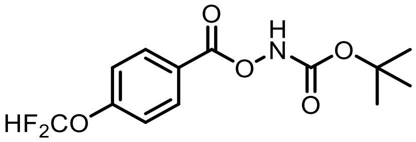

4-(Difluoromethoxy)benzoic acid (940 mg, 5.00 mmol, 1.1 eq.), *tert*-butyl *N*-hydroxycarbamate (665 mg, 5.00 mmol, 1.1 eq.) and DCC (1.09 g, 5.28 mmol, 1.05 eq.) were subject to general procedure A. The crude residue was purified by silica gel chromatography (eluent: Pet Ether:EtOAc 95:5 – 90:10) to give the title compound (1.04 g, 3.42 mmol, 68%) as a white solid.

^{1}H NMR (400 MHz, $CDCl_3$) δ 8.20 (br s, 1H), 8.11 (d, *J* = 8.7 Hz, 2H), 7.18 (d, *J* = 8.6 Hz, 2H), 6.61 (t, *J* = 72.8 Hz, 1H), 1.51 (s, 9H); ^{13}C NMR (126 MHz, $CDCl_3$) δ 165.1, 155.6, 155.5, 132.1, 123.7, 118.7, 115.3 (t, *J* = 262.1 Hz), 83.4, 28.0; ^{19}F NMR (376 MHz, $CDCl_3$) δ -83.04. HRMS (ESI) calcd for $[C_{13}H_{15}F_2NO_5 + Na]^+$: 326.0811. Found: 326.0816.

***tert*-Butyl ((4-(chloromethyl)benzoyl)oxy)carbamate 1ag**

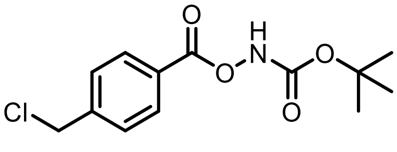

4-(Chloromethyl)benzoic acid (1.71 g, 10.0 mmol, 1 eq.), *tert*-butyl *N*-hydroxycarbamate (1.33 g, 10.0 mmol, 1 eq.) and DCC (2.27 g, 10.5 mmol, 1.05 eq.) were subject to general procedure A. The crude residue was purified

by silica gel chromatography (eluent: Pet Ether:EtOAc 93:7 – 90:10) to give the title compound (2.18 g, 7.64 mmol, 76%) as a white solid.

^{1}H NMR (400 MHz, $CDCl_3$) δ 8.38 (br s, 1H), 8.05 (d, *J* = 8.0 Hz, 2H), 7.48 (d, *J* = 8.0 Hz, 2H), 4.60 (s, 2H), 1.51 (s, 9H); ^{13}C NMR (101 MHz, $CDCl_3$) δ 165.5, 155.6, 143.5, 130.3, 128.7, 126.8, 83.4, 45.2, 28.0; HRMS (ESI) calcd for $[C_{13}H_{16}ClNO_4 + Na]^+$: 308.0660. Found: 308.0663.

***tert*-Butyl ((4-(bromomethyl)benzoyl)oxy)carbamate 1ah**

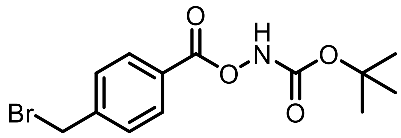

4-(Bromomethyl)benzoic acid (2.15 g, 10.0 mmol, 1 eq.), *tert*-butyl *N*-hydroxycarbamate (1.33 g, 10.0 mmol, 1 eq.) and DCC (2.17 g, 10.5 mmol, 1.05 eq.) were subject to general procedure A. The crude residue was purified by silica gel chromatography (eluent: Pet Ether:EtOAc 95:5 – 90:10) to give the title compound (2.01 g, 6.09 mmol, 61%) as a white solid.

^{1}H NMR (400 MHz, $CDCl_3$) δ 8.21 (br s, 1H), 8.06 (d, *J* = 8.4 Hz, 2H), 7.49 (d, *J* = 8.3 Hz, 2H), 4.49 (s, 2H), 1.51 (s, 9H); ^{13}C NMR (101 MHz, $CDCl_3$) δ 165.5, 155.5, 143.9, 130.4, 129.3, 126.8, 83.5, 31.9, 28.1; HRMS (ESI) calcd for $[C_{13}H_{16}BrNO_4 + Na]^+$: 352.0155. Found: 352.0159.

***tert*-Butyl ((3-methoxybenzoyl)oxy)(methyl)carbamate 3a**

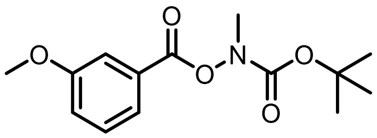

tert-Butyl ((3-methoxybenzoyl)oxy)carbamate (1.12 g, 4.19 mmol, 1 eq.), sodium hydride (60% in mineral oil, 184 mg, 4.61 mmol, 1.1 eq.) and methyl iodide (654 mg, 4.61 mmol, 1.1 eq.) were reacted according to general procedure B. The crude residue was purified by silica gel chromatography (eluent: Pet Ether:EtOAc 100:0 - 95:5) to give the title compound (750 mg, 2.67 mmol, 64%) as a colourless oil.

^{1}H NMR (400 MHz, $CDCl_3$) δ 7.65 (d, *J* = 7.7 Hz, 1H), 7.56 (s, 1H), 7.36 (t, *J* = 7.9 Hz, 1H), 7.15 (dd, *J* = 8.3, 2.8 Hz, 1H), 3.85 (s, 3H), 3.33 (s, 3H), 1.46 (s, 9H); ^{13}C NMR (101 MHz, $CDCl_3$) δ 164.6, 159.6, 155.3, 129.6, 128.8, 122.3, 120.5, 114.1, 82.4, 55.5, 38.0, 28.1; HRMS (ESI) calcd for $[C_{14}H_{19}NO_5 + Na]^+$: 304.1155. Found: 304.1153.

tert-Butyl ((3,5-dimethoxybenzoyl)oxy)(methyl)carbamate 3b

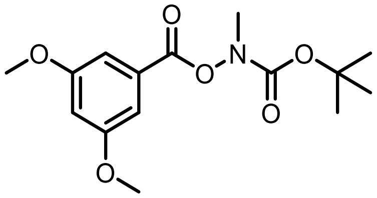

tert-Butyl ((3,5-dimethoxybenzoyl)oxy)carbamate (1.02 g, 3.43 mmol, 1 eq.), sodium hydride (60% in mineral oil, 165 mg, 4.13 mmol, 1.2 eq.) and methyl iodide (538 mg, 3.79 mmol, 1.1 eq.) were reacted according to general procedure B. The crude residue was purified by silica gel chromatography (eluent: Pet Ether:EtOAc 100:0 – 94:6) to give the title compound (749 mg, 2.41 mmol, 70%) as a colourless oil.

^{1}H NMR (400 MHz, $CDCl_3$) δ 7.22 (s, 2H), 6.71 (s, 1H), 3.86 (s, 6H), 3.35 (s, 3H), 1.49 (s, 9H); ^{13}C NMR (101 MHz, $CDCl_3$) δ 164.6, 160.7, 155.3, 129.3, 107.4, 106.7, 82.4, 55.6, 37.9, 28.1; HRMS (ESI) calcd for $[C_{15}H_{21}NO_6 + Na]^+$: 334.1261. Found: 334.1265.

tert-Butyl ((2-chloro-5-methoxybenzoyl)oxy)carbamate S3c

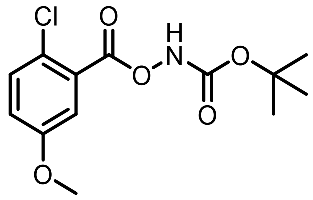

2-Chloro-5-methoxybenzoic acid (933 mg, 5.00 mmol, 1 eq.), *tert*-butyl *N*-hydroxycarbamate (665 mg, 5.00 mmol, 1 eq.) and DCC (1.09 g, 5.28 mmol, 1.05 eq.) were subject to general procedure A. The crude residue was purified by silica gel chromatography (eluent: Pet Ether:EtOAc 95:5 – 88:12) to give the title compound (1.18 g, 3.91 mmol, 78%) as a white solid.

^{1}H NMR (400 MHz, $CDCl_3$) δ 8.22 (s, 1H), 7.45 (d, *J* = 3.1 Hz, 1H), 7.37 (d, *J* = 8.9 Hz, 1H), 7.03 (dd, *J* = 8.9, 3.1 Hz, 1H), 3.84 (s, 3H), 1.53 (s, 9H); ^{13}C NMR (101 MHz, $CDCl_3$) δ 164.9, 158.0, 155.5, 132.0, 127.2, 125.6, 120.3, 116.1, 83.5, 55.8, 28.0; HRMS (ESI) calcd for $[C_{13}H_{16}ClNO_5 + Na]^+$: 324.0609. Found: 324.0612.

tert-Butyl ((2-chloro-5-methoxybenzoyl)oxy)(methyl)carbamate 3c

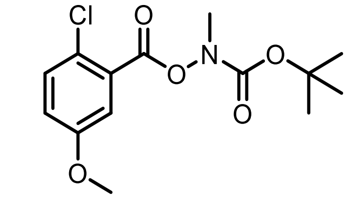

tert-Butyl ((2-chloro-5-methoxybenzoyl)oxy)carbamate (905 mg, 3.00 mmol, 1 eq.), sodium hydride (60% in mineral oil, 144 mg, 3.60 mmol, 1.1 eq.) and methyl iodide (511 mg, 3.62 mmol, 1.1 eq.) were reacted according to general procedure B. The crude residue was purified by silica gel chromatography (eluent: Pet Ether:EtOAc 100:0 - 92:8) to give the title compound (842 mg, 2.67 mmol, 89%) as a colourless oil.

^{1}H NMR (500 MHz, $CDCl_3$) δ 7.36 (dd, *J* = 8.9, 2.1 Hz, 1H), 7.34 – 7.31 (m, 1H), 7.04 – 6.98 (m, 1H), 3.83 (s, 3H), 3.35 (s, 3H), 1.50 (s, 9H); ^{13}C NMR (126 MHz, $CDCl_3$) δ 163.6, 158.0, 155.2, 131.8, 128.3, 125.1, 119.7, 115.9, 82.6, 55.8, 37.9, 28.1; HRMS (ESI) calcd for $[C_{14}H_{18}ClNO_5 + Na]^+$: 338.0766. Found: 338.0766.

tert-Butyl ((2-bromo-3-methoxybenzoyl)oxy)(methyl)carbamate 3d

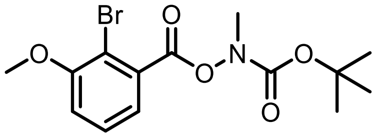

tert-Butyl ((2-bromo-3-methoxybenzoyl)oxy)carbamate (692 mg, 2.00 mmol, 1 eq.), sodium hydride (60% in mineral oil, 96 mg, 2.40 mmol, 1.2 eq.) and methyl iodide (340 mg, 2.41 mmol, 1.2 eq.) were reacted according to general procedure B. The crude residue was purified by silica gel chromatography (eluent: Pet Ether:EtOAc 100:0 - 88:12) to give the title compound (517 mg, 1.44 mmol, 72%) as a colourless oil.

^{1}H NMR (500 MHz, $CDCl_3$) δ 7.36 (t, *J* = 7.8 Hz, 1H), 7.32 (dd, *J* = 7.7, 1.7 Hz, 1H), 7.06 (dd, *J* = 8.0, 1.7 Hz, 1H), 3.93 (s, 3H), 3.36 (s, 3H), 1.50 (s, 9H);^{13}C NMR (126 MHz, $CDCl_3$) δ 164.6, 156.6, 155.3, 132.3, 128.3, 122.5, 114.9, 111.3, 82.6, 56.7, 37.8, 28.2; HRMS (ESI) calcd for $[C_{14}H_{18}BrNO_5 + Na]^+$: 382.0261. Found: 382.0272.

tert-Butyl ((3-methoxy-2-methylbenzoyl)oxy)(methyl)carbamate 3e

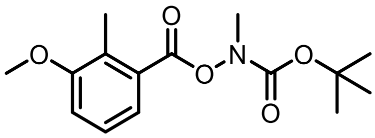

tert-Butyl ((3-methoxy-2-methylbenzoyl)oxy)carbamate (1.41 g, 5.00 mmol, 1 eq.), sodium hydride (60% in mineral oil, 220 mg, 5.50 mmol, 1.1 eq.) and methyl iodide (780 mg, 5.50 mmol, 1.1 eq.) were reacted

according to general procedure B. The crude residue was purified by silica gel chromatography (eluent: Pet Ether:EtOAc 95:5) to give the title compound (1.27 g, 4.30 mmol, 86%) as a white solid.

^{1}H NMR (400 MHz, CDCl$_3$) δ 7.43 (d, *J* = 7.8 Hz, 1H), 7.24 (t, *J* = 8.0 Hz, 1H), 7.04 (d, *J* = 8.2 Hz, 1H), 3.87 (s, 3H), 3.35 (s, 1H), 2.45 (s, 3H), 1.51 (s, 9H); ^{13}C NMR (101 MHz, CDCl$_3$) δ 166.0, 158.1, 155.3, 129.1, 128.8, 126.3, 121.8, 114.0, 82.3, 55.8, 37.8, 28.2, 12.7; HRMS (ESI) calcd for $[C_{15}H_{21}NO_5 + Na]^+$: 318.1312. Found: 318.1310.

tert-Butyl methyl((3-methylbenzoyl)oxy)carbamate 3f

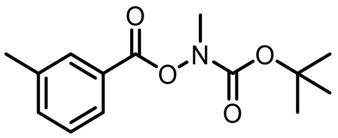

tert-Butyl ((3-methylbenzoyl)oxy)carbamate (1.26 g, 5.00 mmol, 1 eq.), sodium hydride (60% in mineral oil, 240 mg, 6.00 mmol, 1.2 eq.) and methyl iodide (853 mg, 6.01 mmol, 1.2 eq.) were reacted according to general procedure B. The crude residue was purified by silica gel chromatography (eluent: Pet Ether:EtOAc 100:0 - 95:4) to give the title compound (1.08 g, 4.07 mmol, 81%) as a white solid.

^{1}H NMR (400 MHz, CDCl$_3$) δ 7.92 – 7.80 (m, 2H), 7.43 (d, *J* = 7.8 Hz, 1H), 7.36 (t, *J* = 7.6 Hz, 1H), 3.35 (s, 3H), 2.42 (s, 3H), 1.48 (s, 9H); ^{13}C NMR (101 MHz, CDCl$_3$) δ 164.9, 155.3, 138.5, 134.60, 130.3, 128.5, 127.5, 127.0, 82.3, 37.9, 28.1, 21.2; HRMS (ESI) calcd for $[C_{14}H_{19}NO_4 + Na]^+$: 288.1206. Found: 288.1194.

tert-Butyl ((3-chloro-2-methylbenzoyl)oxy)(methyl)carbamate 3g

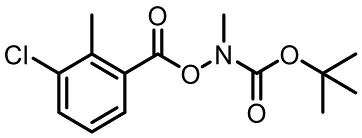

tert-Butyl ((3-chloro-2-methylbenzoyl)oxy)carbamate (1.43 g, 5.01 mmol, 1 eq.), sodium hydride (60% in mineral oil, 220 mg, 5.50 mmol, 1.1 eq.) and methyl iodide (780 mg, 5.50 mmol, 1.1 eq.) were reacted according to general procedure B. The crude residue was purified by silica gel chromatography (eluent: Pet Ether:EtOAc 100:0 - 95:5) to give the title compound (1.24 g, 4.14 mmol, 83%) as a white solid.

^{1}H NMR (400 MHz, CDCl$_3$) δ 7.70 (d, *J* = 7.8 Hz, 1H), 7.49 (d, *J* = 1.3 Hz, 1H), 7.17 (t, *J* = 7.9 Hz, 1H), 3.31 (s, 3H), 2.57 (s, 3H), 1.46 (s, 9H); ^{13}C NMR (101 MHz, CDCl$_3$) δ 165.4, 155.2, 137.4, 136.1, 133.2, 130.0, 128.5, 126.6, 82.5, 37.9, 28.1, 17.3; HRMS (ESI) calcd for $[C_{14}H_{18}ClNO_4 + Na]^+$: 322.0817. Found: 322.0802.

tert-Butyl ((3,4-dimethylbenzoyl)oxy)(methyl)carbamate 3h

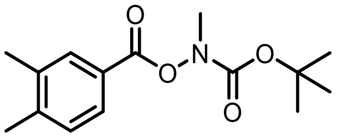

tert-Butyl ((3,4-dimethylbenzoyl)oxy)carbamate (929 mg, 3.50 mmol, 1 eq.), sodium hydride (60% in mineral oil, 168 mg, 4.20 mmol, 1.2 eq.) and methyl iodide (262 µL, 4.24 mmol, 1.2 eq.) were reacted according to general procedure B. The crude residue was purified by silica gel chromatography (eluent: Pet Ether:EtOAc 100:0 - 95:4) to give the title compound (783 mg, 2.80 mmol, 80%) as a colourless oil.

^{1}H NMR (400 MHz, $CDCl_3$) δ 7.82 (d, *J* = 1.8 Hz, 1H), 7.78 (dd, *J* = 7.9, 1.8 Hz, 1H), 7.20 (d, *J* = 7.9 Hz, 1H), 3.31 (s, 3H), 2.31 (s, 3H), 2.29 (s, 3H), 1.45 (s, 9H); ^{13}C NMR (101 MHz, $CDCl_3$) δ 164.9, 155.4, 143.4, 137.0, 130.8, 129.9, 127.5, 125.1, 82.2, 37.9, 28.1, 20.1, 19.6; HRMS (ESI) calcd for $[C_{15}H_{21}NO_4 + Na]^+$: 302.1363. Found: 302.1362.

***tert*-Butyl ((3,5-dimethylbenzoyl)oxy)(methyl)carbamate 3i**

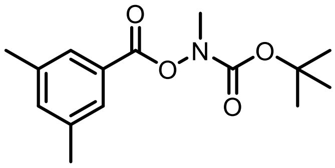

tert-Butyl ((3,5-dimethylbenzoyl)oxy)carbamate (1.33 g, 5.00 mmol, 1 eq.), sodium hydride (60% in mineral oil, 220 mg, 5.50 mmol, 1.1 eq.) and methyl iodide (780 mg, 5.50 mmol, 1.1 eq.) were reacted according to general procedure B. The crude residue was purified by silica gel chromatography (eluent: Pet Ether:EtOAc 100:0 - 95:5) to give the title compound (1.26 g, 4.51 mmol, 90%) as a white solid.

^{1}H NMR (400 MHz, $CDCl_3$) δ 7.67 (s, 2H), 7.21 (s, 1H), 3.31 (s, 3H), 2.34 (s, 6H), 1.45 (s, 9H); ^{13}C NMR (101 MHz, $CDCl_3$) δ 165.0, 155.3, 138.3, 135.5, 127.5, 127.4, 82.2, 37.9, 28.1, 21.1; HRMS (ESI) calcd for $[C_{15}H_{21}NO_4 + Na]^+$: 302.1363. Found: 302.1359.

***tert*-Butyl (2-phenylacetoxy)carbamate 5a**

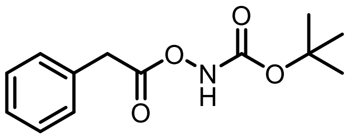

Under an atmosphere of nitrogen, triethylamine (1.11 g, 11.0 mmol, 1.1 eq.) was added to a solution of phenylacetyl chloride (1.55 g, 10.0 mmol, 1 eq.) and *tert*-butyl *N*-hydroxycarbamate (133 g, 10.0 mmol, 1 eq.) in CH_2Cl_2 (20 mL) at 0 °C. The reaction was allowed to warm to room temperature and was stirred for 20 h. The reaction was then quenched with H_2O (100 mL) and the aqueous layer was then extracted with CH_2Cl_2 (3 x 100 mL). The combined organics were then dried ($MgSO_4$), filtered and concentrated *in vacuo.* The crude residue was then purified by silica gel chromatography (eluent: Pet Ether: EtOAc (95:5 – 85:15) to give the title compound (1.96 g, 7.82 mmol, 78%) as a colourless oil.

^{1}H NMR (400 MHz, $CDCl_3$) δ 7.88 (br s, 1H), 7.45 – 7.26 (m, 5H), 3.77 (s, 2H), 1.46 (s, 9H); ^{13}C NMR (101 MHz, $CDCl_3$) δ 170.8, 155.4, 132.2, 129.3, 128.8, 127.6, 83.4, 38.7, 28.0; HRMS (ESI) calcd for $[C_{13}H_{17}NO_4 + Na]^+$: 274.1050. Found: 274.1037.

***tert*-Butyl ((2-phenylpropanoyl)oxy)carbamate 5b**

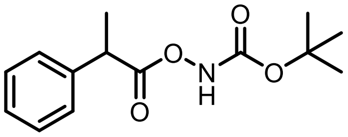

2-Phenylpropanoic acid (1.50 g, 10.0 mmol, 1 eq.), *tert*-butyl *N*-hydroxycarbamate (1.33 g, 10.0 mmol, 1 eq.) and DCC (2.17 g, 10.5 mmol, 1 eq.) were subject to general procedure A. The crude residue was purified by silica gel chromatography (eluent: Pet Ether:EtOAc 95:5 – 90:10) to give the title compound (2.10 g, 7.91 mmol, 79%) as a white solid.

^{1}H NMR (400 MHz, $CDCl_3$) δ 7.86 (br s, 1H), 7.42 – 7.25 (m, 5H), 3.91 (q, *J* = 7.2 Hz, 1H), 1.61 (d, *J* = 7.2 Hz, 3H), 1.46 (s, 9H); ^{13}C NMR (101 MHz, $CDCl_3$) δ 173.8, 155.5, 138.8, 128.8, 127.6, 127.6, 83.2, 43.4, 28.0, 18.5; HRMS (ESI) calcd for $[C_{14}H_{19}NO_4 + Na]^+$: 288.1206. Found: 288.1209.

***tert*-Butyl ((2-methyl-2-phenylpropanoyl)oxy)carbamate 5c**

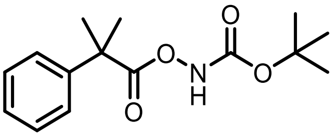

2-Methyl-2-phenylpropanoic acid (1.64 g, 10.0 mmol, 1 eq.), *tert*-butyl *N*-hydroxycarbamate (1.33 gg, 10.0 mmol, 1 eq.) and DCC (2.17 g, 10.5 mmol, 1.05 eq.) were subject to general procedure A. The crude residue was purified by silica gel chromatography (eluent: Pet Ether:EtOAc 95:5 – 90:10) to give the title compound (2.36 g, 8.45 mmol, 84%) as a colourless oil.

^{1}H NMR (400 MHz, $CDCl_3$) δ 7.73 (br s, 1H), 7.45 – 7.34 (m, 4H), 7.34 – 7.24 (m, 1H), 1.71 (s, 6H), 1.49 (s, 9H); ^{13}C NMR (101 MHz, $CDCl_3$) δ 176.2, 155.5, 143.0, 128.5, 127.2, 125.8, 83.2, 45.8, 28.0, 26.5; HRMS (ESI) calcd for $[C_{15}H_{21}NO_4 + Na]^+$: 302.1363. Found: 302.1357.

tert-Butyl ((1-phenylcyclopropane-1-carbonyl)oxy)carbamate 5d

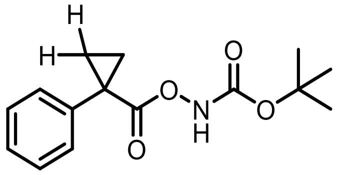

1-Phenyl-1-cyclopropanecarboyxlic acid (1.62 g, 10.0 mmol, 1 eq.), *tert*-butyl *N*-hydroxycarbamate (1.33 g, 10.0 mmol, 1 eq.) and DCC (2.17 g, 10.5 mmol, 1.0 eq.) were subject to general procedure A. The crude residue was purified by silica gel chromatography (eluent: Pet Ether:EtOAc 100:0 – 90:10) to give the title compound (1.72 g, 6.20 mmol, 62%) as a white solid.

^{1}H NMR (400 MHz, $CDCl_3$) δ 7.86 (br s, 1H), 7.47 – 7.40 (m, 2H), 7.39 – 7.25 (m, 3H), 1.80 (q, *J* = 4.1 Hz, 2H), 1.50 (s, 9H), 1.37 (q, *J* = 4.2 Hz, 2H); ^{13}C NMR (101 MHz, $CDCl_3$) δ 174.2, 155.5, 137.7, 130.6, 128.4, 127.7, 83.0, 28.1, 27.9, 17.5; HRMS (ESI) calcd for $[C_{15}H_{19}NO_4 + Na]^+$: 300.1206. Found: 300.1213.

tert-Butyl (2-(2-chlorophenyl)acetoxy)carbamate 5e

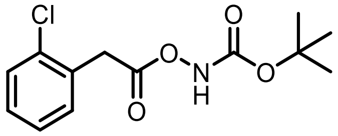

2-Chlorophenylacetic acid (1.71 g, 10.0 mmol, 1 eq.), *tert*-butyl *N*-hydroxycarbamate (1.33 g, 10.0 mmol, 1 eq.) and DCC (2.17 g, 10.5 mmol, 1.05 eq.) were subject to general procedure A. The crude residue was purified by silica gel chromatography (eluent: Pet Ether:EtOAc 95:5 – 85:15) to give the title compound (2.19 g, 7.67 mmol, 77%) as a colourless oil.

^{1}H NMR (400 MHz, $CDCl_3$) δ 8.03 (br s, 1H), 7.36 – 7.28 (m, 1H), 7.28 – 7.23 (m, 1H), 7.19 – 7.11 (m, 2H), 3.84 (s, 2H), 1.40 (s, 9H); ^{13}C NMR (101 MHz, $CDCl_3$) δ 169.9, 155.5, 134.5, 131.6, 130.8, 129.6, 129.2, 127.1, 83.3, 36.5, 28.0; HRMS (ESI) calcd for $[C_{13}H_{16}ClNO_4 + Na]^+$: 308.0660. Found: 308.0658.

tert-Butyl (2-(*o*-tolyl)acetoxy)carbamate 5f

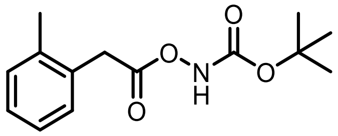

2-Methylphenylacetic acid (1.50 g, 10.0 mmol, 1 eq.), *tert*-butyl *N*-hydroxycarbamate (1.33 g, 10.0 mmol, 1 eq.) and DCC (2.17 g, 10.5 mmol, 1 eq.) were subject to general procedure A. The crude residue was purified by silica gel chromatography (eluent: Pet Ether:EtOAc 95:5 – 85:15) to give the title compound (2.18 g, 8.23 mmol, 82%) as a colourless oil.

^{1}H NMR (400 MHz, $CDCl_3$) 7.88 (br s, 1H), 7.26 – 7.12 (m, 4H), 3.78 (s, 2H), 2.35 (s, 3H), 1.46 (s, 9H); ^{13}C NMR (101 MHz, $CDCl_3$) δ 170.8, 155.4, 137.0, 131.0, 130.5, 130.3, 127.9, 126.3, 83.3, 36.6, 28.0, 19.6; HRMS (ESI) calcd for $[C_{14}H_{19}NO_4 + Na]^+$: 288.1206. Found: 288.1210.

***tert*-Butyl (2-(2-(trifluoromethyl)phenyl)acetoxy)carbamate 5g**

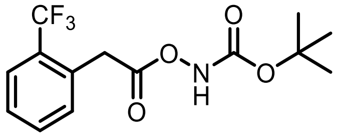

2-(Trifluoromethyl)phenylacetic acid (2.04 g, 10.0 mmol, 1 eq.), *tert*-butyl *N*-hydroxycarbamate (1.33 g, 10.0 mmol, 1 eq.) and DCC (2.17 g, 10.5 mmol, 1 eq.) were subject to general procedure A. The crude residue was purified by silica gel chromatography (eluent: Pet Ether:EtOAc 95:5 – 85:15) to give the title compound (2.05 g, 6.42 mmol, 64%) as a colourless oil.

^{1}H NMR (500 MHz, $CDCl_3$) δ 7.67 (d, *J* = 7.8 Hz, 1H), 7.54 (t, *J* = 7.6 Hz, 1H), 7.45 (d, *J* = 7.8 Hz, 1H), 7.42 (t, *J* = 7.7 Hz, 1H), 3.97 (s, 2H), 1.47 (s, 9H); ^{13}C NMR (126 MHz, $CDCl_3$) δ 170.1, 155.4, 132.5, 132.2 (q, *J* = 1.2 Hz), 130.6 (t, *J* = 1.8 Hz), 129.1 (q, *J* = 30.2 Hz), 127.9, 126.2 (q, *J* = 5.5 Hz), 124.1 (q, *J* = 273.7 Hz), 83.4, 35.7 (q, *J* = 1.9 Hz), 28.0; ^{19}F NMR (376 MHz, $CDCl_3$) δ -59.92; HRMS (ESI) calcd for $[C_{14}H_{16}F_3NO_4 + Na]^+$: 342.0924. Found: 342.0927.

***tert*-Butyl (2-(3,5-dimethylphenyl)acetoxy)carbamate 5h**

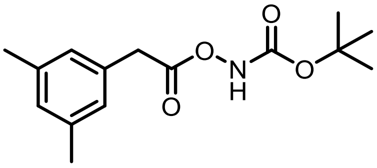

3,5-Dimethylphenylacetic acid (656 mg, 4.00 mmol, 1 eq.), *tert*-butyl *N*-hydroxycarbamate (532 mg, 4.00 mmol, 1 eq.) and DCC (908 mg, 4.40 mmol, 1.1 eq.) were subject to general procedure A. The crude residue was purified by silica gel chromatography (eluent: Pet Ether:EtOAc 97:3 – 90:10) to give the title compound (956 mg, 3.42 mmol, 86%) as a colourless oil.

^{1}H NMR (400 MHz, $CDCl_3$) δ 7.89 (br s, 1H), 6.95 (s, 3H), 3.71 (s, 2H), 2.32 (s, 6H), 1.49 (s, 9H); ^{13}C NMR (101 MHz, $CDCl_3$) δ 171.0, 155.4, 138.3, 132.0, 129.2, 127.1, 83.3, 38.5, 28.0, 21.2; HRMS (ESI) calcd for $[C_{15}H_{21}NO_4 + Na]^+$: 302.1363 Found: 302.1366.

tert-Butyl (2-(3-bromophenyl)acetoxy)carbamate 5i

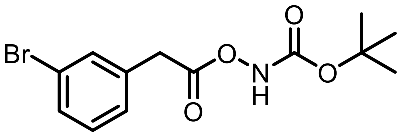

3-Bromophenylacetic acid (2.15 g, 10.0 mmol, 1 eq.), *tert*-butyl *N*-hydroxycarbamate (1.33 g, 10.0 mmol, 1 eq.) and DCC (2.17 g, 10.5 mmol, 1.05 eq.) were subject to general procedure A. The crude residue was purified by silica gel chromatography (eluent: Pet Ether:EtOAc 95:5 – 85:15) to give the title compound (2.19 g, 6.63 mmol, 66%) as a colourless oil.

^{1}H NMR (400 MHz, $CDCl_3$) δ 7.94 (br s, 1H), 7.49 (t, *J* = 1.8 Hz, 1H), 7.45 (dt, *J* = 7.5, 1.7 Hz, 1H), 7.30 – 7.20 (m, 2H), 3.76 (s, 2H), 1.48 (s, 9H); ^{13}C NMR (101 MHz, $CDCl_3$) δ 170.2, 155.3, 134.3, 132.4, 130.8, 130.3, 128.0, 122.7, 83.5, 38.2, 28.0; HRMS (ESI) calcd for $[C_{13}H_{16}BrNO_4 + Na]^+$: 352.0155. Found: 352.0144.

tert-Butyl ((3-(3-bromophenyl)propanoyl)oxy)carbamate 5j

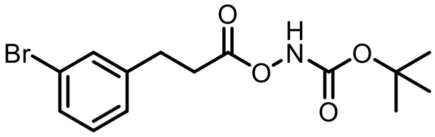

3-(3-Bromophenyl)propionic acid (1.15 g, 5.02 mmol, 1 eq.), *tert*-butyl *N*-hydroxycarbamate (665 mg, 5.00 mmol, 1 eq.) and DCC (1.09 g, 5.28 mmol, 1 eq.) were subject to general procedure A. The crude residue was purified by silica gel chromatography (eluent: Pet Ether:EtOAc 95:5 – 85:15) to give the title compound (1.22 g, 3.54 mmol, 71%) as a colourless oil.

^{1}H NMR (400 MHz, $CDCl_3$) δ 7.88 (br s, 1H), 7.42 – 7.31 (m, 2H), 7.24 – 7.12 (m, 2H), 3.01 (t, *J* = 7.7 Hz, 2H), 2.79 (t, *J* = 7.7 Hz, 2H), 1.51 (s, 9H); ^{13}C NMR (101 MHz, $CDCl_3$) δ 171.8, 155.4, 141.9, 131.4, 130.2, 129.8, 127.0, 122.6, 83.4, 33.2, 30.1, 28.0; HRMS (ESI) calcd for $[C_{14}H_{18}BrNO_4 + Na]^+$: 366.0311. Found: 366.0297.

tert-Butyl ((3-(3,5-dimethoxyphenyl)propanoyl)oxy)carbamate 5k

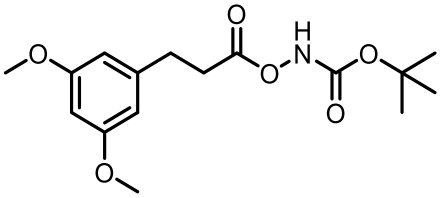

3-(3,5-Dimethoxyphenyl)propionic acid (1.05 g, 5.00 mmol, 1 eq.), *tert*-butyl *N*-hydroxycarbamate (665 mg, 5.00 mmol, 1 eq.) and DCC (1.09 g, 5.28 mmol, 1.05 eq.) were subject to general procedure A. The crude

residue was purified by silica gel chromatography (eluent: Pet Ether:EtOAc 95:5 – 80:20) to give the title compound (1.04 g, 3.20 mmol, 64%) as a colourless oil.

^{1}H NMR (400 MHz, $CDCl_3$) δ 7.91 (br s, 1H), 6.44 – 6.32 (m, 3H), 3.80 (s, 6H), 3.02 – 2.92 (m, 2H), 2.79 (t, *J* = 7.6 Hz, 2H), 1.51 (s, 9H); ^{13}C NMR (101 MHz, $CDCl_3$) δ 172.1, 160.9, 155.4, 142.0, 106.3, 98.5, 83.3, 55.3, 33.4, 30.8, 28.0; HRMS (ESI) calcd for $[C_{16}H_{23}NO_6 + Na]^+$: 348.1418. Found: 348.1410.

***tert*-Butyl ((3-(*p*-tolyl)propanoyl)oxy)carbamate 5l**

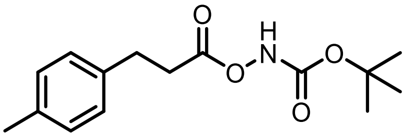

3-(*p*-tolyl)propionic acid (821 mg, 5.00 mmol, 1 eq.), *tert*-butyl *N*-hydroxycarbamate (665 mg, 5.00 mmol, 1 eq.) and DCC (1.09 g, 5.28 mmol, 1.05 eq.) were subject to general procedure A. The crude residue was purified by silica gel chromatography (eluent: Pet Ether:EtOAc 95:5 – 85:15) to give the title compound (1.11 g, 3.96 mmol, 79%) as a colourless oil.

^{1}H NMR (400 MHz, $CDCl_3$) δ 7.88 (br s, 1H), 7.10 (s, 4H), 2.98 (t, *J* = 7.9 Hz, 2H), 2.76 (t, *J* = 7.9 Hz, 2H), 2.32 (s, 3H), 1.49 (s, 9H); ^{13}C NMR (101 MHz, $CDCl_3$) δ 172.2, 155.5, 136.5, 136.1, 129.3, 128.1, 83.3, 33.6, 30.1, 28.0, 21.0; HRMS (ESI) calcd for $[C_{15}H_{21}NO_4 + Na]^+$: 302.1363. Found: 302.1361.

7 Amination Products

2-Aminobenzoic acid hydrochloride 2a

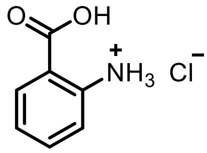

tert-Butyl (benzoyloxy)carbamate (119 mg, 0.50 mmol, 1 eq.), TFA (285 mg, 2.50 mmol, 5 equiv) and $FeSO_4 \cdot 7H_2O$ (1.4 mg, 5.04 µmol, 0.01 eq.) were reacted according to general procedure C. The crude residue was filtered through a short silica plug eluting first with Pet.Ether:EtOAc (90:10) then Pet.Ether:EtOAc (50:50). The title compound (63.6 mg, 0.37 mmol, 73%) was isolated as a yellow solid.

^{1}H NMR (400 MHz, MeOD-*d*$_4$) δ 8.23 (dd, *J* = 7.9, 1.6 Hz, 1H), 7.78 (td, *J* = 7.7, 1.6 Hz, 1H), 7.61 (td, *J* = 7.7, 1.2 Hz, 1H), 7.57 (dd, *J* = 7.9, 1.2 Hz, 1H); ^{13}C NMR (101 MHz, MeOD-*d*$_4$) δ 167.1, 134.2, 132.8, 132.0, 128.6, 124.2, 123.3; HRMS (ESI) calcd for $[C_7H_7NO_2 + H]^+$: 138.0550. Found: 138.0545.

2-Amino-4-methylbenzoic acid hydrochloride 2b

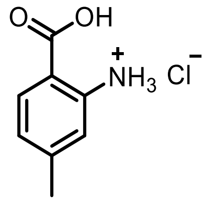

tert-Butyl (4-methylbenzoyloxy)carbamate (126 mg, 0.50 mmol, 1 eq.), TFA (285 mg, 2.50 mmol, 5 eq.) and $FeSO_4 \cdot 7H_2O$ (1.4 mg, 5.04 µmol, 0.01 eq.) were reacted according to general procedure C. The crude residue was filtered through a short silica plug eluting first with Pet.Ether:EtOAc (92:8) then Pet.Ether:EtOAc (60:40). The title compound (66.9 mg, 0.36 mmol, 71%) was isolated as a yellow solid.

^{1}H NMR (400 MHz, MeOD-*d*$_4$) δ 8.10 (d, *J* = 8.6 Hz, 1H), 7.43 (d, *J* = 6.9 Hz, 1H), 7.42 (s, 1H), 2.49 (s, 3H); ^{13}C NMR (101 MHz, MeOD-*d*$_4$) δ 167.2, 146.0, 132.4, 132.0, 129.5, 124.9, 120.6, 20.1; HRMS (ESI) calcd for $[C_8H_9NO_2 + H]^+$: 152.0706. Found: 152.0699.

2-Amino-4-propylbenzoic acid hydrochloride 2c

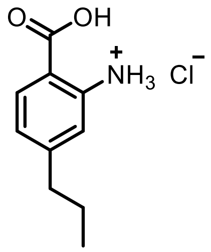

tert-Butyl (4-propylbenzoyloxy)carbamate (158 mg, 0.50 mmol, 1 eq.), TFA (285 mg, 2.50 mmol, 5 eq.) and $FeSO_4{\cdot}7H_2O$ (1.4 mg, 5.04 µmol, 0.01 eq.) were reacted according to general procedure C. The crude residue was filtered through a short silica plug eluting first with Pet.Ether:EtOAc (93:7) then Pet.Ether:EtOAc (60:40). The title compound (64.6 mg, 0.30 mmol, 60%) was isolated as a yellow solid.

^{1}H NMR (400 MHz, MeOD-d_4) δ 8.14 (d, *J* = 8.0 Hz, 1H), 7.45 (dd, *J* = 8.1, 1.6 Hz, 1H), 7.40 (d, *J* = 1.5 Hz, 1H), 2.75 (t, *J* = 7.3 Hz 2H), 1.73 (h, *J* = 7.3 Hz, 2H), 0.99 (t, *J* = 7.4 Hz, 3H); ^{13}C NMR (101 MHz, MeOD-d_4) δ 167.1, 150.5, 132.5, 132.0, 128.9, 124.2, 120.8, 37.2, 23.7, 12.6; HRMS (ESI) calcd for $[C_{10}H_{13}NO_2 + H]^+$: 180.1019. Found: 180.1012.

2-Amino-4-isopropylbenzoic acid hydrochloride 2d

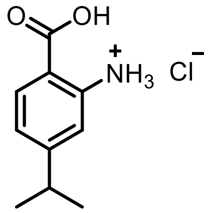

tert-Butyl (4-isopropylbenzoyl)oxy)carbamate (140 mg, 0.50 mmol, 1 eq.), TFA (285 mg, 2.50 mmol, 5 eq.) and $FeSO_4{\cdot}7H_2O$ (1.4 mg, 5.04 µmol, 0.01 eq.) were reacted according to general procedure C. The crude residue was filtered through a short silica plug eluting first with Pet.Ether:EtOAc (95:5) then Pet.Ether:EtOAc (60:40). The title compound (55.0 mg, 0.26 mmol, 51%) was isolated as a pink solid.

^{1}H NMR (400 MHz, MeOD-d_4) δ 8.15 (d, *J* = 8.1 Hz, 1H), 7.49 (dd, *J* = 8.1, 1.7 Hz, 1H), 7.46 (d, *J* = 1.7 Hz, 1H), 3.07 (h, *J* = 6.8 Hz, 1H), 1.32 (d, *J* = 6.9 Hz, 6H); ^{13}C NMR (101 MHz, MeOD-d_4) δ 167.2, 156.5, 132.8, 132.2, 126.8, 122.3, 120.8, 33.9, 22.4; HRMS (ESI) calcd for $[C_{10}H_{13}NO_2 + H]^+$: 180.1019. Found: 180.1012.

2-Amino-5-methylbenzoic acid hydrochloride 2e and 2-amino-3-methylbenzoic acid hydrochloride *iso*-2e

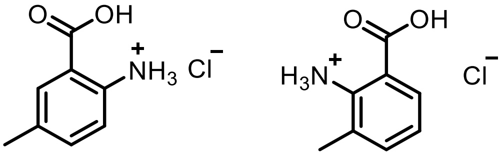

2e   ***iso*-2e**

tert-Butyl (3-methylbenzoyloxy)carbamate (126 mg, 0.50 mmol, 1 eq.), TFA (285 mg, 2.50 mmol, 5 eq.) and $FeSO_4 \cdot 7H_2O$ (1.4 mg, 5.04 µmol, 0.01 eq.) were reacted according to general procedure C. The crude residue was filtered through a short silica plug eluting first with Pet.Ether:EtOAc (92:8) then Pet.Ether:EtOAc (60:40). The title compounds (72.6 mg, 0.39 mmol, 77%) were isolated as a brown solid in a 1.6:1 mixture of regioisomers (crude ratio 1.3:1).

2-Amino-5-methylbenzoic acid hydrochloride 2e

^{1}H NMR (400 MHz, MeOD-d_4) δ 8.05 (d, *J* = 2.0 Hz, 1H), 7.60 (dd, *J* = 8.2, 2.1 Hz, 1H), 7.48 (d, *J* = 8.1 Hz, 1H), 2.46 (s, 3H); ^{13}C NMR (101 MHz, MeOD-d_4) δ 167.1, 139.8, 134.6, 132.4, 129.4, 124.3, 123.4, 19.6.

2-Amino-3-methylbenzoic acid hydrochloride *iso*-2e

^{1}H NMR (400 MHz, MeOD-d_4) δ 8.08 (dd, *J* = 8.0, 1.5 Hz, 1H), 7.66 – 7.62 (m, 1H), 7.44 (t, *J* = 7.8 Hz, 1H), 2.49 (s, 3H); ^{13}C NMR (101 MHz, MeOD-d_4) δ 169.2, 136.3, 133.2, 132.2, 129.7, 127.7, 121.8, 15.9.

HRMS (ESI) calcd for $[C_8H_9NO_2 + H]^+$: 152.0706. Found: 152.0709.

2-Amino-3,5-dimethylbenzoic acid hydrochloride 2f

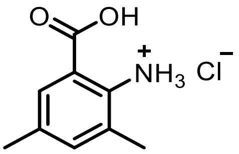

tert-Butyl (3,5-dimethylbenzoyloxy)carbamate (133 mg, 0.50 mmol, 1 eq.), TFA (285 mg, 2.50 mmol, 5 eq.) and $FeSO_4 \cdot 7H_2O$ (1.4 mg, 5.04 µmol, 0.01 eq.) were reacted according to general procedure C. The crude residue was filtered through a short silica plug eluting first with Pet.Ether:EtOAc (92:8) then Pet.Ether:EtOAc (60:40). The title compound (65.1 mg, 0.32 mmol, 65%) was isolated as a yellow solid.

^{1}H NMR (400 MHz, MeOD-d_4) δ 7.92 (s, 1H), 7.50 (s, 1H), 2.47 (s, 3H), 2.42 (s, 3H); ^{13}C NMR (101 MHz, MeOD-d_4) δ 169.2, 139.0, 136.9, 133.4, 130.0, 128.6, 122.1, 19.4, 15.7; HRMS (ESI) calcd for $[C_9H_{11}NO_2 + H]^+$: 166.0863. Found: 166.0855.

2-Amino-5-isopropylbenzoic acid hydrochloride 2g and 2-amino-3-isopropylbenzoic acid hydrochloride *iso*-2g

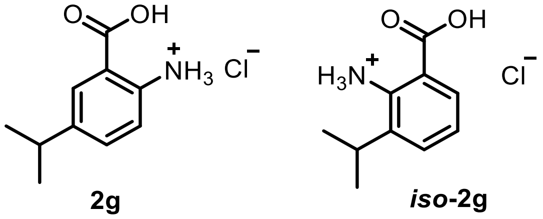

tert-Butyl (3-isopropylbenzoyl)oxy)carbamate (140 mg, 0.50 mmol, 1 eq.), TFA (285 mg, 2.50 mmol, 5 eq.) and $FeSO_4 \cdot 7H_2O$ (1.4 mg, 5.04 µmol, 0.01 eq.) were reacted according to general procedure C. The crude residue was filtered through a short silica plug eluting first with Pet.Ether:EtOAc (92:8) then Pet.Ether:EtOAc (50:50). The title compounds (58.3 mg, 0.27 mmol, 54%) were isolated as a yellow solid as a 1.4:1 mixture of regioisomers (crude ratio 1.7:1).

2-Amino-5-isopropylbenzoic acid hydrochloride 2g

^{1}H NMR (400 MHz, MeOD-d_4) δ 8.14 – 8.03 (m, 1H), 7.67 (dd, *J* = 8.2, 2.2 Hz, 1H), 7.50 (d, *J* = 8.2 Hz, 1H), 3.06 (h, *J* = 7.0 Hz, 1H), 1.31 (d, *J* = 6.9 Hz, 6H); ^{13}C NMR (101 MHz, MeOD-d_4) δ 169.5, 142.7, 132.0, 131.9, 129.7, 127.1, 120.8, 27.1, 22.3.

2-Amino-3-isopropylbenzoic acid hydrochloride *iso*-2g

^{1}H NMR (400 MHz, MeOD-d_4) δ 8.14 – 8.03 (m, 1H), 7.76 (dd, *J* = 8.0, 1.6 Hz, 1H), 7.47 (t, *J* = 7.8 Hz, 1H), 3.20 (h, *J* = 6.7 Hz, 1H), 1.34 (d, *J* = 6.8 Hz, 6H); ^{13}C NMR (101 MHz, MeOD-d_4) δ 167.1, 150.2, 132.2, 130.0, 129.9, 124.4, 123.4, 33.5, 22.7.

HRMS (ESI) calcd for $[C_{10}H_{13}NO_2 + H]^+$: 180.1019. Found: 180.1016.

2-Amino-4,5-dimethylbenzoic acid hydrochloride 2h and 2-amino-3,4-dimethylbenzoic acid hydrochloride *iso*-2h

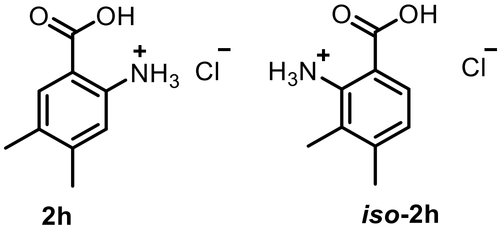

tert-Butyl (3,4-dimethylbenzoyl)oxy)carbamate (133 mg, 0.50 mmol, 1 eq.), TFA (285 mg, 2.50 mmol, 5 eq.) and $FeSO_4 \cdot 7H_2O$ (1.4 mg, 5.04 µmol, 0.01 eq.) were reacted according to general procedure C. The crude residue was filtered through a short silica plug eluting first with Pet.Ether:EtOAc (92:8) then Pet.Ether:EtOAc

(50:50). The title compounds (76.3 mg, 0.38 mmol, 76%) were isolated as a yellow solid as a 1.2:1 mixture of regioisomers (crude ratio 1.3:1).

2-Amino-4,5-dimethylbenzoic acid hydrochloride 2h

^{1}H NMR (400 MHz, MeOD-*d*$_4$) δ 7.99 (s, 1H), 7.34 (s, 1H), 2.41 (s, 3H), 2.37 (s, 3H); ^{13}C NMR (101 MHz, MeOD-*d*$_4$) δ 167.2, 144.4, 138.2, 132.7, 129.7, 125.2, 120.8, 18.6, 17.9.

2-Amino-3,4-dimethylbenzoic acid hydrochloride *iso*-2h

^{1}H NMR (400 MHz, MeOD-*d*$_4$) δ 8.00 (d, *J* = 8.2 Hz, 1H), 7.40 (d, *J* = 8.1 Hz, 1H), 2.45 (s, 3H), 2.37 (s, 3H); ^{13}C NMR (101 MHz, MeOD-*d*$_4$) δ 169.6, 145.4, 131.8, 131.8, 129.3, 129.2, 119.4, 19.4, 12.2.

HRMS (ESI) calcd for $[C_9H_{11}NO_2 + H]^+$: 166.0863. Found: 166.0860.

2-Amino-3,5-di-*tert*-butylbenzoic acid hydrochloride 2i

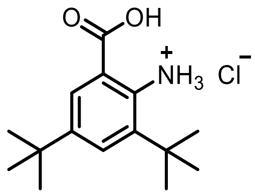

tert-Butyl (3,5-di-*tert*-butylbenzoyl)oxy)carbamate (175 mg, 0.50 mmol, 1 eq.), TFA (285 mg, 2.50 mmol, 5 eq.) and $FeSO_4{\cdot}7H_2O$ (1.4 mg, 5.04 µmol, 0.01 eq.) were reacted according to general procedure C. The crude residue was filtered through a short silica plug eluting first with Pet.Ether:EtOAc (92:8) then Pet.Ether:EtOAc (50:50). The title compound (63.7 mg, 0.22 mmol, 45%) was isolated as a white solid.

^{1}H NMR (400 MHz, MeOD-*d*$_4$) δ 8.18 (d, *J* = 2.4 Hz, 1H), 7.87 (d, *J* = 2.4 Hz, 1H), 1.54 (s, 9H), 1.38 (s, 9H); ^{13}C NMR (101 MHz, MeOD-*d*$_4$) δ 169.6, 150.1, 142.9, 130.1, 130.0, 127.2, 122.1, 35.0, 34.5, 30.1, 30.0; HRMS (ESI) calcd for $[C_{15}H_{23}NO_2 + H]^+$: 250.1802. Found: 250.1797.

2-Amino-4-fluorobenzoic acid 2j

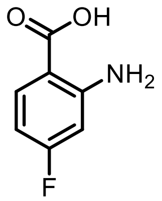

tert-Butyl (4-fluorobenzoyl)oxy)carbamate (76.5 mg, 0.30 mmol, 1 eq.), TFA (170 mg, 1.49 mmol, 5 eq.) and $FeSO_4{\cdot}7H_2O$ (0.8 mg, 2.79 µmol, 0.01 eq.) were reacted according to a modified version of general procedure C. The solvent was then removed under a stream of air and the resulting residue purified by silica gel

chromatography (eluent: Pet.Ether:Et_2O:AcOH 95:4.5:0.5 – 60:39.5:0.5) to give the title compound (17.2 mg, 0.11 mmol, 37%) as a white solid.

^{1}H NMR (400 MHz, MeOD-d_4) δ 7.86 (dd, J = 9.0, 6.8 Hz, 1H), 6.43 (dd, J = 11.6, 2.6 Hz, 1H), 6.29 (td, J = 8.7, 2.5 Hz, 1H); ^{13}C NMR (101 MHz, MeOD-d_4) δ 169.4, 166.6 (d, J = 248.9 Hz), 153.9 (d, J = 13.0 Hz), 134.1 (d, J = 11.6 Hz), 106.8, 102.5 (d, J = 23.0 Hz), 101.1 (d, J = 24.6 Hz); ^{19}F NMR (471 MHz, MeOD-d_4) δ -108.57.

Data matches commercial authentic sample.

2-Amino-5-fluorobenzoic acid hydrochloride 2k and 2-amino-3-fluorobenzoic acid hydrochloride *iso*-2k

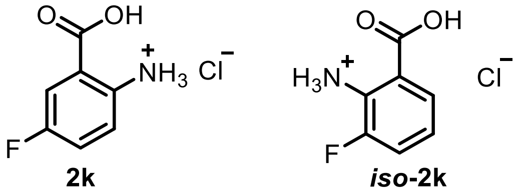

tert-Butyl (3-fluorobenzoyl)oxy)carbamate (128 mg, 0.50 mmol, 1 eq.), TFA (285 mg, 2.50 mmol, 5 eq.) and $FeSO_4 \cdot 7H_2O$ (1.4 mg, 5.04 µmol, 0.01 eq.) were reacted according to general procedure C. The crude residue was filtered through a short silica plug eluting first with Pet.Ether:EtOAc (93:7) then Pet.Ether:EtOAc (50:50). The title compounds (50.0 mg, 0.26 mmol, 52%) were isolated as a pale pink solid as a 3.6:1 mixture of regioisomers (crude ratio 2.6:1).

2-Amino-5-fluorobenzoic acid hydrochloride 2k

^{1}H NMR (500 MHz, MeOD-d_4) δ 7.97 – 7.86 (m, 1H), 7.70 – 7.61 (m, 1H), 7.59 – 7.45 (m, 1H); ^{13}C NMR (126 MHz, MeOD-d_4) δ 165.8 (d, J = 2.2 Hz), 161.7 (d, J = 249.0 Hz), 128.6 (d, J = 3.3 Hz), 126.6 (d, J = 8.5 Hz), 125.8 (d, J = 7.6 Hz), 121.0 (d, J = 23.4 Hz) 118.5 (d, J = 25.0 Hz); ^{19}F NMR (376 MHz, MeOD-d_4) δ -114.09.

2-Amino-3-fluorobenzoic acid hydrochloride *iso*-2k

^{1}H NMR (500 MHz, MeOD-d_4) δ 7.81 – 7.72 (m, 1H), 7.35 – 7.24 (m, 1H), 7.00 – 6.83 (m, 1H); ^{13}C NMR (126 MHz, MeOD-d_4) δ 168.5 (d, J = 3.6 Hz), 153.0 (d, J = 241.8 Hz), 133.9 (d, J = 14.5 Hz), 126.8 (d, J = 3.4 Hz), 119.0 (d, J = 7.6 Hz), 118.8 (d, J = 18.8 Hz), 116.6 (d, J = 3.2 Hz); ^{19}F NMR (376 MHz, MeOD-d_4) δ -135.05.

HRMS (ESI) calcd for $[C_7H_6FNO_2 + H]^+$: 156.0455. Found: 156.0451.

6-Amino-3-fluoro-2-methylbenzoic acid hydrochloride 2l

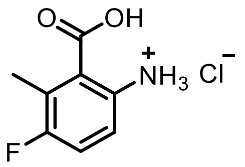

tert-Butyl (3-fluoro-2-methylbenzoyloxy)carbamate (135 mg, 0.50 mmol, 1 eq.), TFA (285 mg, 2.50 mmol, 5 eq.) and $FeSO_4{\cdot}7H_2O$ (1.4 mg, 5.04 µmol, 0.01 eq.) were reacted according to general procedure C. The crude residue was filtered through a short silica plug eluting first with Pet.Ether:EtOAc (92:8) then Pet.Ether:EtOAc (60:40). The title compound (43.1 mg, 0.21 mmol, 42%) was isolated as a yellow solid.

^{1}H NMR (500 MHz, MeOD-d_4) δ 7.45 (dd, *J* = 8.9, 4.6 Hz, 1H), 7.41 (t, *J* = 8.7 Hz, 1H), 2.48 (d, *J* = 2.5 Hz, 3H); ^{13}C NMR (126 MHz, MeOD-d_4) δ 166.1 (d, *J* = 2.6 Hz), 160.9 (d, *J* = 246.4 Hz), 128.8 (d, *J* = 3.9 Hz), 127.8 (d, *J* = 18.9 Hz), 126.2 (d, *J* = 3.5 Hz), 123.1 (d, *J* = 9.4 Hz), 118.2 (d, *J* = 25.9 Hz), 11.4 (d, *J* = 5.8 Hz); ^{19}F NMR (471 MHz, MeOD-d_4) δ -115.50. HRMS (ESI) calcd for $[C_8H_8FNO_2 + H]^+$: 170.0612. Found: 170.0612.

2-Amino-4-chlorobenzoic acid 2m

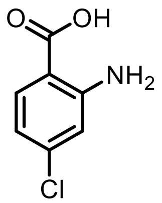

tert-Butyl (4-chlorobenzoyl)oxy)carbamate (81.5 mg, 0.30 mmol, 1 eq.), TFA (170 mg, 1.49 mmol, 5 eq.) and $FeSO_4{\cdot}7H_2O$ (0.8 mg, 2.79 µmol, 0.01 eq.) were reacted according to a modified version of general procedure C. The solvent was then removed under a stream of air and the resulting residue purified by silica gel column chromatography (eluent: Pet.Ether:Et_2O:AcOH 95:4.5:0.5 – 40:49.5:0.5) to give the title compound (17.3 mg, 0.10 mmol, 34%) as a white solid.

^{1}H NMR (400 MHz, MeOD-d_4) δ 7.78 (d, *J* = 8.6 Hz, 1H), 6.77 (d, *J* = 2.1 Hz, 1H), 6.53 (dd, *J* = 8.6, 2.1 Hz, 1H); ^{13}C NMR (101 MHz, MeOD-d_4) δ 169.5, 152.5, 139.3, 132.8, 115.2, 115.0, 108.8.

Data matches commercial authentic sample.

6-Amino-2-chloro-3-methylbenzoic acid hydrochloride 2n

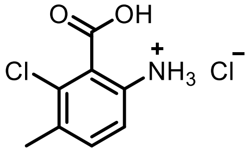

tert-Butyl (2-chloro-3-methylbenzoyloxy)carbamate (143 mg, 0.50 mmol, 1 eq.), TFA (285 mg, 2.50 mmol, 5 eq.) and $FeSO_4 \cdot 7H_2O$ (1.4 mg, 5.04 µmol, 0.01 eq.) were reacted according to general procedure C. The crude residue was filtered through a short silica plug eluting first with Pet.Ether:EtOAc (92:8) then Pet.Ether:EtOAc (70:30). The title compound (67.0 mg, 0.30 mmol, 60%) was isolated as a pink solid.

^{1}H NMR (500 MHz, MeOD-d_4) δ 7.58 (dd, *J* = 8.2, 0.9 Hz, 1H), 7.41 (d, *J* = 8.2 Hz, 1H), 2.47 (s, 3H); ^{13}C NMR (126 MHz, MeOD-d_4) δ 165.2, 138.5, 133.1, 133.0, 128.3, 128.0, 121.9, 19.1; HRMS (ESI) calcd for $[C_8H_8ClNO_2 + H]^+$: 186.0316. Found: 186.0315.

2-Amino-6-chlorobenzoic acid hydrochloride 2o

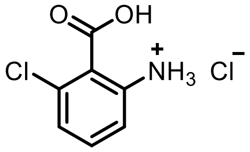

tert-Butyl (2-chlorobenzoyloxy)carbamate (136 mg, 0.50 mmol, 1 eq.), TFA (285 mg, 2.50 mmol, 5 eq.) and $FeSO_4 \cdot 7H_2O$ (1.4 mg, 5.04 µmol, 0.01 equiv) were reacted according to general procedure C. The crude residue was filtered through a short silica plug eluting first with Pet.Ether:EtOAc (90:10) then Pet.Ether:EtOAc (50:50). The title compound (41.4 mg, 0.20 mmol, 40%) was isolated as a yellow solid.

^{1}H NMR (400 MHz, MeOD-d_4) δ 7.63 – 7.55 (m, 2H), 7.50 – 7.39 (m, 1H); ^{13}C NMR (101 MHz, MeOD-d_4) δ 165.0, 133.9, 132.4, 132.2, 130.0, 126.5, 121.9; HRMS (ESI) calcd for $[C_7H_6ClNO_2 + H]^+$: 172.0160. Found: 172.0153.

2-Amino-5-chlorobenzoic acid hydrochloride 2p and 2-amino-3-chlorobenzoic acid hydrochloride *iso*-2p

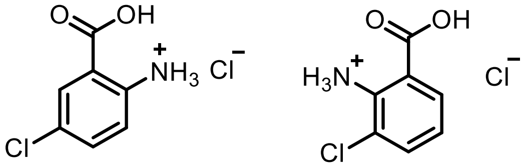

2p ***iso*-2p**

tert-Butyl (3-chlorobenzoyloxy)carbamate (136 mg, 0.50 mmol, 1 eq.), TFA (285 mg, 2.50 mmol, 5 eq.) and $FeSO_4 \cdot 7H_2O$ (1.4 mg, 5.04 µmol, 0.01 eq.) were reacted according to general procedure C. The crude residue was filtered through a short silica plug eluting first with Pet.Ether:EtOAc (90:10) then Pet.Ether:EtOAc (50:50).

The title compounds (47.8 mg, 0.23 mmol, 46%) were isolated as a yellow solid in a 2.6:1 mixture of regioisomers, crude ratio (1.6:1).

2-Amino-5-chlorobenzoic acid hydrochloride 2p

^{1}H NMR (400 MHz, MeOD-d_4) δ 8.12 (d, *J* = 2.5 Hz, 1H), 7.72 (dd, *J* = 8.6, 2.5 Hz, 1H), 7.50 (d, *J* = 8.6 Hz, 1H); ^{13}C NMR (101 MHz, MeOD-d_4) δ 165.9, 133.9, 133.2, 132.6, 131.5, 125.4, 124.2.

2-Amino-3-chlorobenzoic acid hydrochloride *iso*-2p

^{1}H NMR (400 MHz, MeOD-d_4) δ 7.87 (dd, *J* = 8.0, 1.5 Hz, 1H), 7.47 (dd, *J* = 7.9, 1.6 Hz, 1H), 6.73 (t, *J* = 7.9 Hz, 1H); ^{13}C NMR (101 MHz, MeOD-d_4) δ 169.1, 144.3, 133.6, 130.3, 121.1, 117.3, 113.0.

HRMS (ESI) calcd for $[C_7H_6ClNO_2 + H]^+$: 172.0160. Found: 172.0153.

6-Amino-3-chloro-2-methylbenzoic acid hydrochloride 2q

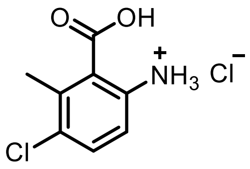

tert-Butyl (3-chloro-2-methylbenzoyloxy)carbamate (143 mg, 0.50 mmol, 1 eq.), TFA (285 mg, 2.50 mmol, 5 eq.) and $FeSO_4 \cdot 7H_2O$ (1.4 mg, 5.04 µmol, 0.01 eq.) were reacted according to general procedure C. The crude residue was filtered through a short silica plug eluting first with Pet.Ether:EtOAc (92:8) then Pet.Ether:EtOAc (60:40). The title compound (54.6 mg, 0.25 mmol, 49%) was isolated as a yellow solid.

^{1}H NMR (400 MHz, MeOD-d_4) δ 7.69 (d, *J* = 8.5 Hz, 1H), 7.41 (d, *J* = 8.5 Hz, 1H), 2.59 (s, 3H); ^{13}C NMR (101 MHz, MeOD-d_4) δ 166.4, 137.4, 135.8, 132.0, 129.2, 128.7, 122.5, 17.2; HRMS (ESI) calcd for $[C_8H_8ClNO + H]^+$: 186.0316. Found: 186.0309.

To prove the regiochemical outcome of the reaction, 6-amino-3-chloro-2-methylbenzoic acid **2q** was converted to the corresponding quinazolin-4(3*H*)-one **S2q**.

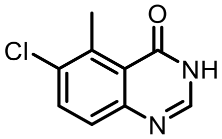

tert-Butyl (3-chloro-2-methylbenzoyloxy)carbamate (143 mg, 0.50 mmol, 1 eq.), TFA (285 mg, 2.50 mmol, 5 eq.) and $FeSO_4 \cdot 7H_2O$ (1.4 mg, 5.04 µmol, 0.01 eq.) were reacted according to general procedure C. The crude residue was filtered through a silica plug (eluent: EtOAc) and the solvent removed under a stream of air. The resulting residue was then dissolved in dry MeOH (1.5 mL). NH_4OAc (193 mg, 2.50 mmol, 5 eq.) and

trimethylorthoformate (185 mg, 1.75 mmol, 3.5 eq.) were added and the reaction was stirred at 95 °C for 14 h. The solvent was removed under a stream of air and then saturated aqueous Na_2CO_3 (20 mL) was added. The aqueous layer was extracted with CH_2Cl_2 (3 x 50 mL) and the combined organics were dried ($MgSO_4$), filtered and concentrated *in vacuo.* The resulting residue was then purified by silica gel chromatography (eluent: Pet.Ether:EtOAc 70:30 – 0:100) to give the title compound (25.2 mg, 0.13 mmol, 26 %) as a white solid.

^{1}H NMR (500 MHz, MeOD-d_4) δ 8.01 (s, 1H), 7.78 (d, *J* = 8.7 Hz, 1H), 7.50 (d, *J* = 8.7 Hz, 1H), 2.94 (s, 3H); ^{13}C NMR (126 MHz, MeOD-d_4) δ 161.6*, 148.9*, 145.2, 137.8, 134.6, 133.2, 125.7, 122.2, 17.0; HRMS (ESI) calcd for $[C_9H_7ClN_2O + H]^+$: 195.0320. Found: 195.0319.

*visible by HMBC only.

2-Amino-4-bromobenzoic acid hydrochloride 2r

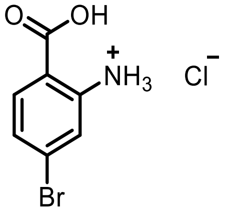

tert-Butyl (4-bromobenzoyl)oxy)carbamate (136 mg, 0.50 mmol, 1 eq.), TFA (285 mg, 2.50 mmol, 5 eq.) and $FeSO_4{\cdot}7H_2O$ (1.4 mg, 5.04 µmol, 0.01 eq.) were reacted according to general procedure C. The crude residue was filtered through a short silica plug eluting first with Pet.Ether:EtOAc (90:10) then Pet.Ether:EtOAc (50:50). The title compound (40.0 mg, 0.16 mmol, 32%) was isolated as a yellow solid.

^{1}H NMR (400 MHz, MeOD-d_4) δ 8.02 (d, J = 8.5 Hz, 1H), 7.59 (d, J = 1.9 Hz, 1H), 7.54 (dd, J = 8.5, 1.9 Hz, 1H); ^{13}C NMR (101 MHz, MeOD-d_4) δ 167.0, 138.1, 133.3, 128.8, 127.8, 125.4, 119.6; HRMS (ESI) calcd for $[C_7H_6BrNO_2 + H]^+$: 215.9655. Found: 215.9653.

2-Amino-6-bromobenzoic acid 2s

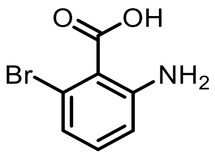

tert-Butyl (2-bromobenzoyl)oxy)carbamate (94.9 mg, 0.30 mmol, 1 eq.), TFA (171 mg, 1.50 mmol, 5 eq.) and $FeSO_4{\cdot}7H_2O$ (0.8 mg, 2.79 µmol, 0.01 eq.) were reacted according to a modified version of general procedure C. The solvent was removed under a stream of air and the resulting residue purified by silica gel chromatography (eluent: Pet.Ether:Et_2O:AcOH 95:4.5:0.5 – 60:39.5:0.5) to give the title compound (32.3 mg, 0.15 mmol, 50%) as a pink solid.

^{1}H NMR (400 MHz, MeOD-d_4) δ 7.01 (t, *J* = 8.0 Hz, 1H), 6.88 (dd, *J* = 7.9, 1.1 Hz, 1H), 6.74 (dd, *J* = 8.2, 1.0 Hz, 1H); ^{13}C NMR (101 MHz, MeOD-d_4) δ 169.1, 148.5, 131.3, 121.3, 120.6, 118.4, 115.0; HRMS (ESI) calcd for $[C_7H_6BrNO_2 + H]^+$: 215.9655. Found: 215.9656.

6-Amino-2-bromo-3-methylbenzoic acid hydrochloride 2t

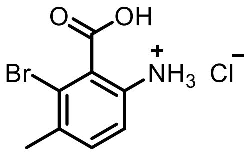

tert-Butyl (2-bromo-3-methylbenzoyloxy)carbamate (165 mg, 0.50 mmol, 1 eq.), TFA (285 mg, 2.50 mmol, 5 eq.) and $FeSO_4 \cdot 7H_2O$ (1.4 mg, 5.04 µmol, 0.01 eq.) were reacted according to general procedure C. The crude residue was filtered through a short silica plug eluting first with Pet.Ether:EtOAc (90:10) then Pet.Ether:EtOAc (60:40). The title compound (61.6 mg, 0.23 mmol, 46%) was isolated as a pink solid.

^{1}H NMR (500 MHz, MeOD-d_4) δ 7.56 (dd, *J* = 8.2, 0.8 Hz, 1H), 7.42 (d, *J* = 8.2 Hz, 1H), 2.50 (s, 3H); ^{13}C NMR (126 MHz, MeOD-d_4) δ 165.9, 140.3, 132.5, 130.7, 127.9, 123.1, 122.3, 22.2; HRMS (ESI) calcd for $[C_8H_8BrNO_2 + H]^+$: 229.9811. Found: 229.9802.

2-Amino-5-bromobenzoic acid hydrochloride 2u and 2-amino-3-bromobenzoic acid hydrochloride *iso*-2u

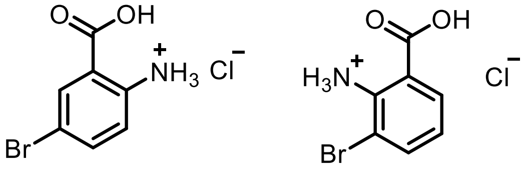

2u **_iso_-2u**

tert-Butyl (3-bromobenzoyloxy)carbamate (158 mg, 0.50 mmol, 1 eq.), TFA (285 mg, 2.50 mmol, 5 eq.) and $FeSO_4 \cdot 7H_2O$ (1.4 mg, 5.04 µmol, 0.01 eq.) were reacted according to general procedure C. The crude residue was filtered through a short silica plug eluting first with Pet.Ether:EtOAc (90:10) then Pet.Ether:EtOAc (50:50). The title compounds (35.7 mg, 0.14 mmol, 28%) were isolated as a brown solid in a 6:1 mixture of regioisomers (crude ratio 1.6:1).

2-Amino-5-bromobenzoic acid hydrochloride 2u

^{1}H NMR (400 MHz, MeOD-d_4) δ 8.24 (d, *J* = 2.4 Hz, 1H), 7.82 (dd, *J* = 8.5, 2.4 Hz, 1H), 7.36 (d, *J* = 8.6 Hz, 1H); ^{13}C NMR (101 MHz, MeOD-d_4) δ, 166.1, 136.8, 134.5, 134.4, 125.0, 123.3, 119.5.

2-Amino-3-bromobenzoic acid hydrochloride *iso*-2u

^{1}H NMR (400 MHz, MeOD-d_4) δ 7.89 (dd, *J* = 8.0, 1.5 Hz, 1H), 7.61 (dd, *J* = 7.8, 1.6 Hz, 1H), 6.62 (t, *J* = 7.9 Hz, 1H); ^{13}C NMR (101 MHz, MeOD-d_4) δ 169.1, 146.2, 137.0, 131.0, 117.2, 113.3, 110.7.

HRMS (ESI) calcd for $[C_7H_6BrNO_2 + H]^+$: 215.9655. Found: 215.9649.

2-Amino-5-iodobenzoic acid hydrochloride 2v and 2-amino-3-iodobenzoic acid hydrochloride *iso*-2v

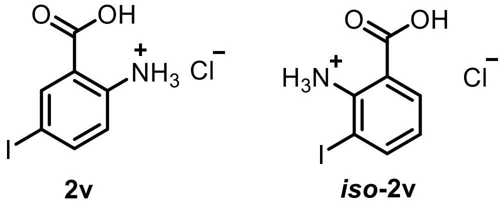

tert-Butyl (3-iodobenzoyl)oxy)carbamate (182 mg, 0.50 mmol, 1 eq.), TFA (285 mg, 2.50 mmol, 5 eq.) and $FeSO_4·7H_2O$ (1.4 mg, 5.04 µmol, 0.01 eq.) were reacted according to modified version of general procedure C. The crude residue was filtered through a short silica plug eluting with Pet.Ether/EtOAc (40:60). HCl/E_2O (2 M, 10 eq.) was added to the resulting residue and the resulting suspension was stirred at 0 ℃ for 1 h. The title compounds (42.6 mg, 0.14 mmol, 28%) were isolated as a brown solid as a 2.4:1 mixture of regioisomers (crude ratio 2:1).

2-Amino-5-iodobenzoic acid hydrochloride 2v

^{1}H NMR (400 MHz, MeOD-d_4) δ 8.43 (d, *J* = 2.0 Hz, 1H), 8.00 (dd, *J* = 8.4, 2.1 Hz, 1H), 7.20 (d, *J* = 8.5 Hz, 1H); ^{13}C NMR (101 MHz, MeOD-d_4) δ 166.1, 142.8, 140.5, 135.6, 124.7, 122.9, 89.5.

2-Amino-3-iodobenzoic acid hydrochloride *iso*-2v

^{1}H NMR (400 MHz, MeOD-d_4) δ 7.95 (dd, *J* = 7.9, 1.6 Hz, 1H), 7.88 (dd, *J* = 7.7, 1.5 Hz, 1H), 6.54 (t, *J* = 7.8 Hz, 1H); ^{13}C NMR (101 MHz, MeOD-d_4) δ 169.1, 144.1, 132.0, 128.5, 118.6, 112.8, 85.9.

HRMS (ESI) calcd for $[C_7H_6INO_2 + H]^+$: 263.9516. Found: 263.9510.

6-Amino-2,3-dichlorobenzoic acid hydrochloride 2w

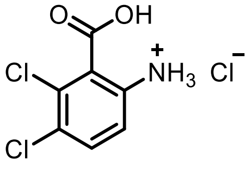

tert-Butyl (2,3-dichlorobenzoyloxy)carbamate (153 mg, 0.50 mmol, 1 eq.), TFA (285 mg, 2.50 mmol, 5 eq.) and $FeSO_4·7H_2O$ (1.4 mg, 5.04 µmol, 0.01 eq.) were reacted according to general procedure C. The crude residue was filtered through a short silica plug eluting first with Pet.Ether:EtOAc (90:10) then Pet.Ether:EtOAc (60:40). The title compound (61.7 mg, 0.25 mmol, 51%) was isolated as a brown solid.

^{1}H NMR (400 MHz, MeOD-d_4) δ 7.77 (d, *J* = 8.7 Hz, 1H), 7.45 (d, *J* = 8.7 Hz, 1H); ^{13}C NMR (101 MHz, MeOD-d_4) δ 164.6, 132.5, 132.4, 131.5, 131.4, 128.4, 122.6; HRMS (ESI) calcd for $[C_7H_6Cl_2NO_2 + H]^+$: 205.9770. Found: 205.9771.

To prove the regiochemical outcome of the reaction, 6-amino-2,3-dichlorobenzoic acid **2w** was converted to the corresponding quinazolin-4(3*H*)-one **S2w**.

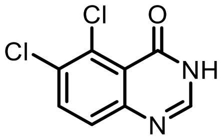

tert-Butyl (2,3-dichlorobenzoyloxy)carbamate (153 mg, 0.50 mmol, 1 eq.), TFA (285 mg, 2.50 mmol, 5 eq.) and $FeSO_4{\cdot}7H_2O$ (1.4 mg, 5.04 µmol, 0.01 eq.) were reacted according to general procedure C. The crude residue was filtered through a silica plug (eluent: EtOAc) and the solvent removed under a stream of air. The resulting residue was then dissolved in dry MeOH (1.5 mL). NH_4OAc (190 mg, 2.50 mmol, 5 eq.) and trimethylorthoformate (185 mg, 1.75 mmol, 3.5 eq.) were added and the reaction was stirred at 95 °C for 14 h. The solvent was removed under a stream of air and then saturated aqueous Na_2CO_3 (20 mL) was added. The aqueous layer was extracted with CH_2Cl_2 (3 x 50 mL) and the combined organics were dried ($MgSO_4$), filtered and concentrated *in vacuo.* The resulting residue was then purified by silica gel chromatography (eluent: Pet.Ether:EtOAc 70:30 – 0:100) to give the title compound (8.2 mg, 0.038 mmol, 8%) as a white solid.

^{1}H NMR (500 MHz, MeOD-d_4) δ 8.06 (s, 1H), 7.91 (d, *J* = 8.8 Hz, 1H), 7.61 (d, *J* = 8.8 Hz, 1H); ^{13}C NMR (126 MHz, MeOD-d_4) δ 159.2, 149.6, 146.1, 134.9, 132.5, 131.2, 126.8, 121.1; HRMS (ESI) calcd for $[C_8H_4Cl_2N_2O + H]^+$: 214.9973. Found: 214.9973.

2-Amino-3,5-dibromobenzoic acid hydrochloride 2x

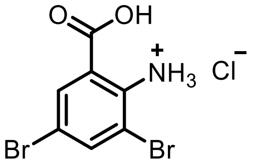

tert-Butyl (3,5-dibromobenzoyl)oxy)carbamate (198 mg, 0.50 mmol, 1 eq.), TFA (285 mg, 2.50 mmol, 5 eq.) and $FeSO_4{\cdot}7H_2O$ (1.4 mg, 5.04 µmol, 0.01 eq.) were reacted according to a modified version of general procedure C. The crude residue was filtered through a short silica plug eluting with Pet.Ether:EtOAc (40:60). HCl/Et_2O (2 M, 10 eq.) was added to the resulting residue and the resulting suspension was stirred at 0 °C for 1 h. The title compound (59.4 mg, 0.18 mmol, 36%) was isolated as a purple solid.

^{1}H NMR (400 MHz, MeOD-d_4) δ 7.96 (d, *J* = 2.4 Hz, 1H), 7.71 (d, *J* = 2.4 Hz, 1H); ^{13}C NMR (101 MHz, MeOD-d_4) δ 168.1, 147.2, 138.5, 133.1, 112.7, 110.2, 105.2; HRMS (ESI) calcd for $[C_7H_5Br_2NO_2 + H]^+$: 293.8760. Found: 293.8752.

2-Amino-3,5-difluorobenzoic acid hydrochloride 2y

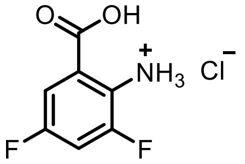

tert-Butyl (3,5-difluorobenzoyl)oxy)carbamate (138 mg, 0.50 mmol, 1 eq.), TFA (285 mg, 2.50 mmol, 5 eq.) and $FeSO_4 \cdot 7H_2O$ (1.4 mg, 5.04 µmol, 0.01 eq.) were reacted according to general procedure C. The crude residue was filtered through a short silica plug eluting first with Pet.Ether:EtOAc (95:5) then Pet.Ether:EtOAc (50:50). The title compound (32.0 mg, 0.15 mmol, 31%) was isolated as a pale orange solid.

^{1}H NMR (500 MHz, MeOD-d_4) δ 7.52 (ddd, *J* = 9.2, 3.0, 1.8 Hz, 1H), 7.27 (ddd, *J* = 11.0, 8.1, 3.0 Hz, 1H); ^{13}C NMR (126 MHz, MeOD-d_4) δ 167.4 – 167.2 (m), 156.1 – 154.0 (m), 154.0 – 151.8 (m), 130.3 (d, *J* = 13.5 Hz), 116.9 (dd, *J* = 8.2, 4.4 Hz), 112.3 (dd, *J* = 23.5, 3.7 Hz), 108.1 (dd, *J* = 27.7, 23.2 Hz); ^{19}F NMR (376 MHz, MeOD-d_4) δ -123.45, -129.87; HRMS (ESI) calcd for $[C_7H_5F_2NO_2 + H]^+$: 174.0361. Found: 174.0355.

2-Amino-5-methoxybenzoic acid hydrochloride 2z and 2-amino-3-methoxybenzoic acid hydrochloride *iso*-2z

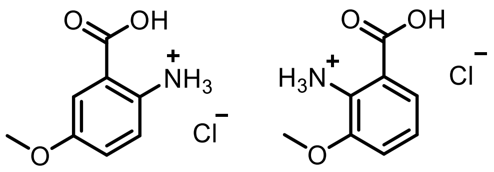

2z ***iso*-2z**

tert-Butyl (3-methoxybenzoyloxy)carbamate (158 mg, 0.50 mmol, 1 eq.), TFA (285 mg, 2.50 mmol, 5 eq.) and $FeSO_4 \cdot 7H_2O$ (1.4 mg, 5.04 µmol, 0.01 eq.) were reacted according to general procedure C. The crude residue was filtered through a short silica plug eluting first with Pet.Ether:EtOAc (90:10) then Pet.Ether:EtOAc (50:50). The title compounds (71.6 mg, 0.35 mmol, 70%) were isolated as a purple solid in a 1.7:1 mixture of regioisomers (crude ratio 1.3:1).

2-Amino-5-methoxybenzoic acid hydrochloride 2z

^{1}H NMR (400 MHz, DMSO-d_6) δ 7.56 (d, *J* = 8.8 Hz, 1H), 7.45 (d, *J* = 3.0 Hz, 1H), 7.25 (dd, *J* = 8.8, 3.0 Hz, 1H), 3.79 (s, 3H); ^{13}C NMR (101 MHz, DMSO-d_6) δ 166.8, 158.2, 126.9, 126.2, 124.7, 120.0, 116.2, 56.2.

2-Amino-3-methoxybenzoic acid hydrochloride *iso*-2z

^{1}H NMR (400 MHz, DMSO-d_6) δ 7.32 (dd, *J* = 8.2, 1.3 Hz, 1H), 6.98 (dd, *J* = 7.8, 1.3 Hz, 1H), 6.57 (t, *J* = 8.0 Hz, 1H), 3.79 (s, 3H); ^{13}C NMR (101 MHz, DMSO-d_6) δ 169.8, 147.7, 140.1, 122.9, 115.7, 114.1, 111.3, 56.2.

HRMS (ESI) calcd for $[C_8H_9NO_3 + H]^+$: 168.0655. Found: 168.0648.

6-Amino-3-methoxy-2-methylbenzoic acid hydrochloride 2aa

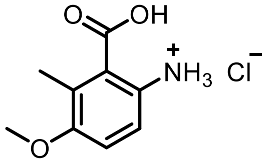

tert-Butyl (3-methoxy-2-methylbenzoyloxy)carbamate (142 mg, 0.50 mmol, 1 eq.), TFA (285 mg, 2.50 mmol, 5 eq.) and $FeSO_4 \cdot 7H_2O$ (1.4 mg, 5.04 µmol, 0.01 eq.) were reacted according to general procedure C. The crude residue was filtered through a short silica plug eluting first with Pet.Ether:EtOAc (90:10) then Pet.Ether:EtOAc (30:70). The title compound (46.4 mg, 0.21 mmol, 43%) was isolated as a brown solid.

^{1}H NMR (400 MHz, MeOD-d_4) δ 7.39 (d, J = 8.7 Hz, 1H), 7.19 (d, J = 8.8 Hz, 1H), 3.92 (s, 3H), 2.39 (s, 3H); ^{13}C NMR (101 MHz, MeOD-d_4) δ 167.2, 158.3, 128.7, 128.1, 122.2, 121.3, 112.4, 55.3, 12.4; HRMS (ESI) calcd for $[C_9H_{11}NO_3 + H]^+$: 182.0812. Found: 182.0803.

To prove the regiochemical outcome of the reaction, 6-amino-3-methoxy-2-methylbenzoic acid **2aa** was converted to the corresponding quinazolin-4(3*H*)-one **S2aa**.

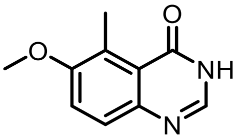

tert-Butyl (3-methoxy-2-methylbenzoyloxy)carbamate (142 mg, 0.50 mmol, 1 eq.), TFA (285 mg, 2.50 mmol, 5 eq.) and $FeSO_4 \cdot 7H_2O$ (1.4 mg, 5.04 µmol, 0.01 eq.) were reacted according to general procedure C. The crude residue was filtered through a silica plug (eluent: EtOAc) and the solvent removed under a stream of air. The resulting residue was then dissolved in dry MeOH (1.5 mL). NH_4OAc (193 mg, 2.50 mmol, 5 eq.) and trimethylorthoformate (185 mg, 1.75 mmol, 3.5 equiv) were added and the reaction was stirred at 95 °C for 14 h. The solvent was removed under a stream of air and then saturated aqueous Na_2CO_3 (20 mL) was added. The aqueous layer was extracted with CH_2Cl_2 (3 x 50 mL) and the combined organics were dried ($MgSO_4$), filtered and concentrated *in vacuo.* The resulting residue was then purified by silica gel chromatography (eluent: Pet.Ether:EtOAc 50:50 – 0:100) to give the title compound (29.2 mg, 0.15 mmol, 31 %) as a white solid.

^{1}H NMR (500 MHz, MeOD-d_4) δ 7.86 (s, 1H), 7.53 (d, J = 9.0 Hz, 1H), 7.49 (d, J = 8.9 Hz, 1H), 3.92 (s, 3H), 2.73 (s, 3H); ^{13}C NMR (126 MHz, MeOD-d_4) δ 162.6, 156.1, 143.2, 142.5, 127.3, 125.0, 121.5, 117.7, 55.4, 12.0; HRMS (ESI) calcd for $[C_{10}H_{10}N_2O_2 + H]^+$: 191.0815. Found: 191.0816.

6-Amino-2-bromo-3-methoxybenzoic acid hydrochloride 2ab

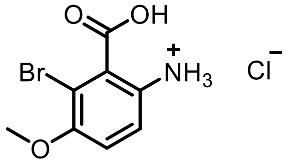

tert-Butyl (2-bromo-3-methoxybenzoyl)oxy)carbamate (173 mg, 0.50 mmol, 1 eq.), TFA (285 mg, 2.50 mmol, 5 eq.) and $FeSO_4 \cdot 7H_2O$ (1.4 mg, 5.04 µmol, 0.01 eq.) were reacted according to general procedure C. The crude residue was filtered through a short silica plug eluting first with Pet.Ether:EtOAc (90:10) then Pet.Ether:EtOAc (50:50). The title compound (45.9 mg, 0.16 mmol, 32%) was isolated as a purple solid.

^1H NMR (400 MHz, MeOD-d_4) δ 7.53 (d, J = 8.8 Hz, 1H), 7.29 (d, J = 8.9 Hz, 1H), 3.98 (s, 3H); ^{13}C NMR (101 MHz, MeOD-d_4) δ 165.5, 156.8, 131.7, 123.9, 121.6, 113.3, 111.0, 56.1; HRMS (ESI) calcd for $[C_8H_8BrNO_3 + H]^+$: 245.9760. Found: 245.9757.

To prove the regiochemical outcome of the reaction, 6-amino-2-bromo-3-methoxybenzoic acid **2ab** was converted to the corresponding quinazolin-4(3*H*)-one **S2ab**.

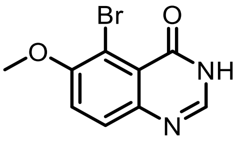

tert-Butyl (2-bromo-3-methoxybenzoyloxy)carbamate (242 mg, 0.70 mmol, 1 eq.), TFA (396 mg, 3.48 mmol, 5 eq.) and $FeSO_4 \cdot 7H_2O$ (2.0 mg, 7.19 µmol, 0.01 eq.) were reacted according to general procedure C. The crude residue was filtered through a silica plug (eluent: EtOAc) and the solvent removed under a stream of air. The resulting residue was then dissolved in dry MeOH (1.5 mL). NH_4OAc (189 mg, 2.45 mmol, 3.5 eq.) and trimethylorthoformate (262 mg, 2.45 mmol, 3.5 eq.) were added and the reaction was stirred at 95 °C for 14 h. The solvent was removed under a stream of air and then saturated aqueous Na_2CO_3 (20 mL) was added. The aqueous layer was extracted with CH_2Cl_2 (3 x 50 mL) and the combined organics were dried ($MgSO_4$), filtered and concentrated *in vacuo.* The resulting residue was then purified by silica gel chromatography (eluent: Pet.Ether:EtOAc 50:50 – 0:100) to give the title compound (35.7 mg, 0.14 mmol, 20%) as a yellow solid.

^1H NMR (500 MHz, DMSO-d_6) δ 12.15 (br s, 1H), 7.96 (s, 1H), 7.68 (d, J = 9.0 Hz, 1H), 7.63 (d, J = 9.0 Hz, 1H), 3.94 (s, 3H); ^{13}C NMR (126 MHz, DMSO-d_6) δ 159.6, 155.0, 144.9, 144.1, 128.6, 121.7, 119.4, 108.3, 57.4; HRMS (ESI) calcd for $[C_9H_7BrN_2O_2 + H]^+$: 254.9764. Found: 254.9769.

2-Amino-3,5-dimethoxybenzoic acid hydrochloride 2ac

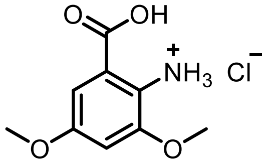

tert-Butyl (3,5-dimethoxybenzoyloxy)carbamate (149 mg, 0.50 mmol, 1 eq.), TFA (285 mg, 2.50 mmol, 5 eq.) and $FeSO_4 \cdot 7H_2O$ (1.4 mg, 5.04 µmol, 0.01 eq.) were reacted according to general procedure C. The crude residue was filtered through a short silica plug eluting first with Pet.Ether:EtOAc (90:10) then Pet.Ether:EtOAc (50:50). The title compound (68.9 mg, 0.29 mmol, 59%) was isolated as a yellow solid.

^{1}H NMR (400 MHz, MeOD-d_4) δ 7.28 (d, J = 2.7 Hz, 1H), 7.04 (d, J = 2.7 Hz, 1H), 4.03 (s, 3H), 3.91 (s, 3H); ^{13}C NMR (101 MHz, MeOD-d_4) δ 167.5, 160.3, 154.2, 124.5, 113.9, 106.9, 103.4, 56.1, 55.1; HRMS (ESI) calcd for $[C_9H_{11}NO_4 + H]^+$: 198.0761. Found: 198.0755.

2-Amino-6-(trifluoromethyl)benzoic acid hydrochloride 2ad

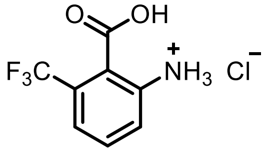

tert-Butyl 2-((trifluoromethyl)benzoyloxy)carbamate (158 mg, 0.50 mmol, 1 eq.), TFA (285 mg, 2.50 mmol, 5 eq.) and $FeSO_4 \cdot 7H_2O$ (1.4 mg, 5.04 µmol, 0.01 eq.) were reacted according to general procedure C. The crude residue was filtered through a short silica plug eluting first with Pet.Ether:EtOAc (90:10) then Pet.Ether:EtOAc (50:50). The title compound (50.4 mg, 0.21 mmol, 42%) was isolated as a yellow solid.

^{1}H NMR (500 MHz, MeOD-d_4) δ 7.92 – 7.85 (m, 1H), 7.84 – 7.79 (m, 2H); ^{13}C NMR (126 MHz, MeOD-d_4) δ 165.5, 131.9, 131.7, 129.7 (q, J = 32.7 Hz), 127.2, 126.3 (d, J = 2.0 Hz), 126.0 (q, J = 5.3 Hz), 123.0 (q, J = 273.1 Hz); ^{19}F NMR (471 MHz, MeOD-d_4) δ -61.51; HRMS (ESI) calcd for $[C_8H_6F_3NO + H]^+$: 206.0423. Found: 206.0421.

2-Amino-4-(trifluoromethyl)benzoic acid 2ae

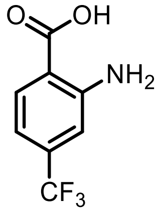

tert-Butyl (4-(trifluoromethyl)benzoyl)oxy)carbamate (91.6 mg, 0.30 mmol, 1 eq.), TFA (170 mg, 1.49 mmol, 5 eq.) and $FeSO_4 \cdot 7H_2O$ (0.8 mg, 2.78 µmol, 0.01 eq.) were reacted according to a modified version of general

procedure C. The solvent was then removed under a stream of air and the resulting residue purified by silica gel chromatography (eluent: Pet.Ether:Et_2O:AcOH 95:4.5:0.5– 60:39.5:0.5) to give the title compound (18.7 mg, 0.091 mmol, 30%) as a white solid.

^{1}H NMR (500 MHz, MeOD-d_4) δ 7.95 (d, J = 8.4 Hz, 1H), 7.02 (d, J = 1.7 Hz, 1H), 6.75 (dd, J = 8.4, 1.8 Hz, 1H); ^{13}C NMR (126 MHz, MeOD-d_4) δ 169.1, 151.5, 134.7 (q, J = 31.8 Hz), 132.3, 123.9 (q, J = 271.9 Hz), 112.7 (q, J = 4.2 Hz), 112.5, 110.3 (q, J = 3.6 Hz); ^{19}F NMR (376 MHz, MeOD-d_4) δ -65.41.

Data matches commercial authentic sample.

2-Amino-4-(difluoromethoxy)benzoic acid 2af

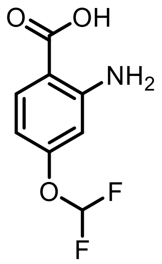

tert-Butyl (4-(difluoromethoxyl)benzoyl)oxy)carbamate (90.9 mg, 0.30 mmol, 1 eq.), TFA (170 mg, 1.49 mmol, 5 eq.) and $FeSO_4 \cdot 7H_2O$ (0.8 mg, 2.88 µmol, 0.01 eq.) were reacted according to a modified version of general procedure C. The solvent was then removed under a stream of air and the resulting residue purified by silica gel chromatography (eluent: Pet.Ether:Et_2O:AcOH 95:4.5:0.5 – 60:39.5:0.5) to give the title compound (20.7 mg, 0.10 mmol, 34%) as a white solid.

^{1}H NMR (500 MHz, MeOD-d_4) δ 7.85 (d, J = 8.8 Hz, 1H), 6.85 (t, J = 74.0 Hz, 1H), 6.46 (d, J = 2.4 Hz, 1H), 6.32 (dd, J = 8.8, 2.4 Hz, 1H); ^{13}C NMR (126 MHz, MeOD-d_4) δ 169.5, 156.2 (t, J = 3.1 Hz), 153.3, 133.6, 116.0 (t, J = 256.9 Hz), 107.2, 105.0, 103.9; ^{19}F NMR (471 MHz, MeOD-d_4) δ -83.81; HRMS (ESI) calcd for $[C_8H_7F_2NO_3 + H]^+$: 204.0467. Found: 204.0465.

To prove the regiochemical outcome of the reaction, 2-amino-4-(difluoromethoxy)benzoic acid **2af** was converted to the corresponding quinazolin-4(3*H*)-one **S2af**.

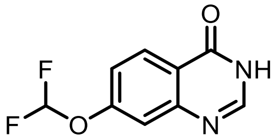

2-Amino-4-(difluoromethoxy)benzoic acid (20.7 mg, 0.10 mmol, 1 eq.), NH_4OAc (27 mg, 0.35 mmol, 3.5 eq.) and trimethylorthoformate (38.8 mg, 0.37 mmol, 3.5 equiv) were dissolved in dry MeOH (1 mL) and the reaction was stirred at 95 °C for 14 h. The solvent was removed under a stream of air and then saturated aqueous Na_2CO_3 (20 mL) was added. The aqueous layer was extracted with CH_2Cl_2 (3 x 50 mL) and the

combined organics were dried ($MgSO_4$), filtered and concentrated *in vacuo.* The resulting residue was then purified by silica gel chromatography (eluent: Pet.Ether:EtOAc 50:50 – 0:100) to give the title compound (16.7 mg, 0.079 mmol, 77%) as a white solid.

^{1}H NMR (500 MHz, MeOD-d_4) δ 8.25 (d, *J* = 8.8 Hz, 1H), 8.12 (s, 1H), 7.38 (d, *J* = 2.4 Hz, 1H), 7.32 (dd, *J* = 8.8, 2.4 Hz, 1H), 7.27 – 6.87 (m, 1H); ^{13}C NMR (126 MHz, MeOD-d_4) δ 161.1, 156.3, 150.2, 146.2, 128.3, 119.2, 118.1, 118.0 – 113.7 (m), 114.0; ^{19}F NMR (376 MHz, MeOD-d_4) δ -85.01; HRMS (ESI) calcd for $[C_9H_6F_2N_2O_2 + H]^+$: 213.0470. Found: 213.0472.

2-Amino-4-(chloromethyl)benzoic acid hydrochloride 2ag

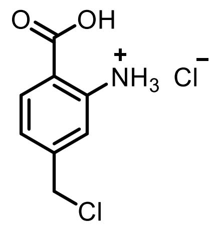

tert-Butyl ((4-(chloromethyl)benzoyl)oxy)carbamate (143 mg, 0.50 mmol, 1 eq.), TFA (285 mg, 2.50 mmol, 5 eq.) and $FeSO_4 \cdot 7H_2O$ (1.4 mg, 5.04 µmol, 0.01 eq.) were reacted according to general procedure C. The crude residue was filtered through a short silica plug eluting first with Pet.Ether:EtOAc (90:10) then Pet.Ether:EtOAc (50:50). The title compound (51.7 mg, 0.23 mmol, 47%) was isolated as a brown solid.

^{1}H NMR (400 MHz, MeOD-d_4) δ 8.20 (d, *J* = 8.0 Hz, 1H), 7.66 (d, *J* = 1.6 Hz, 1H), 7.63 (dd, *J* = 8.1, 1.8 Hz, 1H), 4.78 (s, 2H); ^{13}C NMR (101 MHz, MeOD-d_4) δ 166.7, 144.8, 133.4, 132.4, 128.3, 124.1, 122.7, 43.7; HRMS (ESI) calcd for $[C_8H_8ClNO_2 + H]^+$: 186.0316. Found: 186.0315.

2-Amino-4-(bromomethyl)benzoic acid hydrochloride 2ah

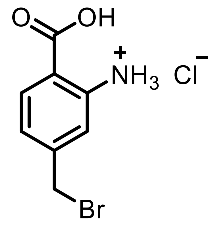

tert-Butyl ((4-(bromomethyl)benzoyl)oxy)carbamate (165 mg, 0.50 mmol, 1 eq.), TFA (285 mg, 2.50 mmol, 5 eq.) and $FeSO_4 \cdot 7H_2O$ (1.4 mg, 5.04 µmol, 0.01 eq.) were reacted according to general procedure C. The crude residue was filtered through a short silica plug eluting first with Pet.Ether:EtOAc (90:10) then Pet.Ether:EtOAc (50:50). The title compound (57.3 mg, 0.22 mmol, 43%) was isolated as a brown solid.

^1H NMR (400 MHz, MeOD-*d*$_4$) δ 8.19 (d, *J* = 8.5 Hz, 1H), 7.67 – 7.59 (m, 2H), 4.67 (s, 2H); ^{13}C NMR (101 MHz, MeOD-*d*$_4$) δ 166.7, 145.3, 133.2, 132.5, 128.9, 124.7, 122.8, 30.1; HRMS (ESI) calcd for $[C_8H_8BrNO_2 + H]^+$: 229.9811. Found: 229.9806.

5-Methoxy-2-(methylamino)benzoic acid hydrochloride 4a and 3-methoxy-2-(methylamino)benzoic acid hydrochloride *iso*-4a

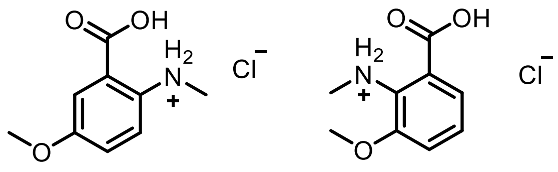

4a ***iso*-4a**

tert-Butyl ((3-methoxybenzoyl)oxy)(methyl)carbamate (141 mg, 0.50 mmol, 1 eq.), TFA (285 mg, 2.50 mmol, 5 eq.) and $FeSO_4 \cdot 7H_2O$ (1.4 mg, 5.04 µmol, 0.01 eq.) were reacted in HFIP (10 mL) according to a modified version of general procedure C. The crude residue was filtered through a short silica plug eluting with EtOAc. The title compounds (96.9 mg, 0.45 mmol, 89%) were isolated as a white solid, in a 5.4:1 mixture of regioisomers (crude ratio 4:1).

5-Methoxy-2-(methylamino)benzoic acid hydrochloride 4a

^1H NMR (500 MHz, MeOD-*d*$_4$) δ 7.73 (d, *J* = 3.0 Hz, 1H), 7.63 (d, *J* = 8.9 Hz, 1H), 7.37 (dd, *J* = 8.9, 3.0 Hz, 1H), 3.92 (s, 3H), 3.11 (s, 3H); ^{13}C NMR (126 MHz, MeOD-*d*$_4$) δ 167.1, 160.1, 130.7, 124.8, 123.6, 119.7, 117.0, 55.2, 37.0.

3-Methoxy-2-(methylamino)benzoic acid hydrochloride *iso*-4a

^1H NMR (500 MHz, MeOD-*d*$_4$) δ 7.82 (dd, *J* = 7.8, 1.3 Hz, 1H), 7.66 (t, *J* = 8.3 Hz, 1H), 7.59 (dd, *J* = 8.4, 1.3 Hz, 1H), 4.09 (s, 3H), 3.11 (s, 3H); ^{13}C NMR (126 MHz, MeOD-*d*$_4$) δ 167.5, 152.9, 130.7, 126.1, 123.6, 123.2, 117.5, 56.2, 35.7.

HRMS (ESI) calcd for $[C_9H_{11}NO_3 + H]^+$: 182.0812. Found: 182.0804.

3,5-Dimethoxy-2-(methylamino)benzoic acid hydrochloride 4b

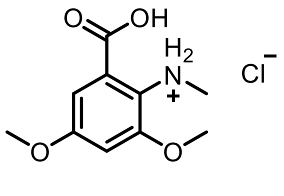

tert-Butyl ((3,5-dimethoxybenzoyl)oxy)(methyl)carbamate (117 mg, 0.37 mmol, 1 eq.), TFA (209 mg, 1.83 mmol, 5 eq.) and $FeSO_4 \cdot 7H_2O$ (1.4 mg, 5.04 µmol, 0.01 eq.) were reacted in HFIP (8 mL) according to a modified

version of general procedure C. The crude residue was filtered through a short silica plug eluting with CH_2Cl_2/MeOH (90:10). The title compound (76.3 mg, 0.31 mmol, 83%) was isolated as a purple solid.

^{1}H NMR (400 MHz, MeOD-*d*$_4$) δ 7.30 (d, *J* = 2.6 Hz, 1H), 7.04 (d, *J* = 2.7 Hz, 1H), 4.06 (s, 3H), 3.92 (s, 3H), 3.05 (s, 3H); ^{13}C NMR (101 MHz, MeOD-*d*$_4$) δ 167.9, 161.1, 154.0, 125.3, 119.2, 107.4, 103.5, 56.1, 55.2, 35.6; HRMS (ESI) calcd for $[C_{10}H_{13}NO_4 + H]^+$: 212.0917. Found: 212.0918.

6-Chloro-3-methoxy-2-(methylamino)benzoic acid hydrochloride 4c

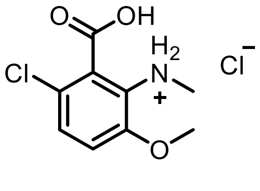

tert-Butyl ((2-chloro-5-methoxybenzoyl)oxy)(methyl)carbamate (158 mg, 0.50 mmol, 1 eq.), TFA (285 mg, 2.50 mmol, 5 eq.) and $FeSO_4·7H_2O$ (1.4 mg, 5.04 µmol, 0.01 eq.) were reacted in HFIP (10 mL) according to a modified version of general procedure C. The crude residue was filtered through a short silica plug eluting with EtOAc. The title compound (62.5 mg, 0.25 mmol, 50%) was isolated as an orange solid.

^{1}H NMR (400 MHz, MeOD-*d*$_4$) δ 7.63 (d, *J* = 9.0 Hz, 1H), 7.41 (d, *J* = 9.1 Hz, 1H), 4.07 (s, 3H), 3.11 (s, 3H); ^{13}C NMR (101 MHz, MeOD-*d*$_4$) δ 165.1, 151.2, 131.9, 128.1, 124.3, 123.6, 115.2, 56.4, 36.4; HRMS (ESI) calcd for $[C_9H_{10}ClNO_3 + H]^+$: 216.0422. Found: 216.0415.

2-Bromo-3-methoxy-6-(methylamino)benzoic acid hydrochloride 4d

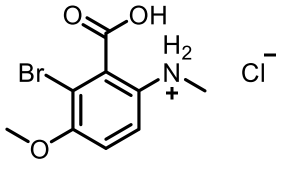

tert-Butyl ((2-bromo-3-methoxybenzoyl)oxy)(methyl)carbamate (158 mg, 0.50 mmol, 1 eq.), TFA (285 mg, 2.50 mmol, 5 eq.) and $FeSO_4·7H_2O$ (1.4 mg, 5.04 µmol, 0.01 eq.) were reacted in HFIP (10 mL) according to a modified version of general procedure C. The crude residue was filtered through a short silica plug eluting with EtOAc. The title compound (86.6 mg, 0.29 mmol, 58%) was isolated as a red solid.

^{1}H NMR (400 MHz, MeOD-*d*$_4$) δ 7.63 (d, *J* = 9.0 Hz, 1H), 7.33 (d, *J* = 9.0 Hz, 1H), 3.99 (s, 3H), 3.11 (s, 3H); ^{13}C NMR (101 MHz, MeOD-*d*$_4$) δ 165.7, 157.1, 131.7, 127.3, 123.2, 113.5, 110.7, 56.2, 37.5; HRMS (ESI) calcd for $[C_9H_{10}BrNO_3 + H]^+$: 259.9917. Found: 259.9908.

3-Methoxy-2-methyl-6-(methylamino)benzoic acid hydrochloride 4e

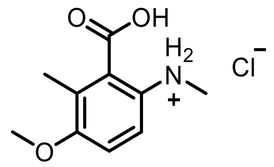

tert-Butyl ((3-methoxy-2-methylbenzoyl)oxy)(methyl)carbamate (148 mg, 0.50 mmol, 1 eq.), TFA (285 mg, 2.50 mmol, 5 eq.) and $FeSO_4 \cdot 7H_2O$ (1.4 mg, 5.04 µmol, 0.01 eq.) were reacted in HFIP (10 mL) according to a modified version of general procedure C. The crude residue was filtered through a short silica plug eluting with CH_2Cl_2:MeOH (90:10). The title compound (82.3 mg, 0.36 mmol, 71%) was isolated as an orange solid.

^{1}H NMR (400 MHz, MeOD-d_4) δ 7.47 (d, J = 8.9 Hz, 1H), 7.25 (d, J = 8.9 Hz, 1H), 3.94 (s, 3H), 3.09 (s, 3H), 2.39 (s, 3H); ^{13}C NMR (101 MHz, MeOD-d_4) δ 167.6, 158.6, 128.7, 127.6, 127.5, 121.3, 112.7, 55.3, 37.4, 12.5; HRMS (ESI) calcd for $[C_{10}H_{13}NO_3]^+$: 195.0890. Found: 195.0889.

5-Methyl-2-(methylamino)benzoic acid hydrochloride 4f and 3-methyl-2-(methylamino)benzoic acid hydrochloride *iso*-4f

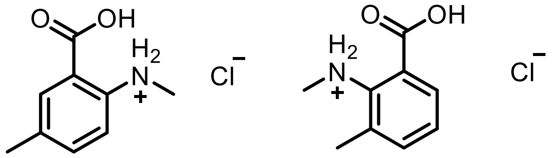

4f ***iso*-4f**

tert-Butyl ((3-methylbenzoyl)oxy)(methyl)carbamate (133 mg, 0.50 mmol, 1 eq.), TFA (285 mg, 2.50 mmol, 5 eq.) and $FeSO_4 \cdot 7H_2O$ (1.4 mg, 5.04 µmol, 0.01 eq.) were reacted in HFIP (10 mL) according to a modified version of general procedure C. The crude residue was filtered through a short silica plug eluting with EtOAc. The title compounds (44.2 mg, 0.22 mmol, 44%) were isolated as a red solid in a 3.3:1 mixture of regioisomers (crude ratio 2.1:1).

5-Methyl-2-(methylamino)benzoic acid hydrochloride 4f

^{1}H NMR (400 MHz, MeOD-d_4) δ 8.06 (d, J = 1.8 Hz, 1H), 7.64 (dd, J = 8.2, 1.9 Hz, 1H), 7.54 (d, J = 8.1 Hz, 1H), 3.11 (s, 3H), 2.46 (s, 3H); ^{13}C NMR (101 MHz, MeOD-d_4) δ 167.6, 139.6, 136.5, 135.2, 132.5, 122.7, 121.5, 36.5, 19.5.

3-Methyl-2-(methylamino)benzoic acid hydrochloride *iso*-4f

^{1}H NMR (400 MHz, MeOD-d_4) δ 8.15 (dd, *J* = 7.8, 1.3 Hz, 1H), 7.73 (d, *J* = 7.6 Hz, 1H), 7.59 (t, *J* = 7.8 Hz, 1H), 3.15 (s, 3H), 2.60 (s, 3H); ^{13}C NMR (101 MHz, MeOD-d_4) δ 168.5, 137.6, 133.3, 133.2, 130.3, 129.6, 122.4, 36.0, 15.7.

HRMS (ESI) calcd for $[C_9H_{11}NO_2 + H]^+$: 166.0863. Found: 166.0863.

3-Chloro-2-methyl-6-(methylamino)benzoic acid hydrochloride 4g

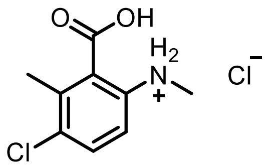

tert-Butyl ((3-chloro-2-methylbenzoyl)oxy)(methyl)carbamate (150 mg, 0.50 mmol, 1 eq.), TFA (285 mg, 2.50 mmol, 5 eq.) and $FeSO_4 \cdot 7H_2O$ (1.4 mg, 5.04 µmol, 0.01 eq.) were reacted in HFIP (10 mL) according to general procedure C. The crude residue was filtered through a short silica plug eluting first with Pet.Ether:EtOAc (90:10) then EtOAc. The title compound (62.9 mg, 0.26 mmol, 52%) was isolated as a grey solid.

^{1}H NMR (400 MHz, MeOD-d_4) δ 7.71 (d, *J* = 8.7 Hz, 1H), 7.47 (d, *J* = 8.7 Hz, 1H), 3.12 (s, 3H), 2.57 (s, 3H); ^{13}C NMR (101 MHz, MeOD-d_4) δ 166.9, 137.2, 135.6, 135.0, 132.3, 128.22, 121.0, 36.7, 17.3; HRMS (ESI) calcd for $[C_9H_{10}ClNO_2 + H]^+$: 200.0473. Found: 200.0468.

4,5-Dimethyl-2-(methylamino)benzoic acid hydrochloride 4h

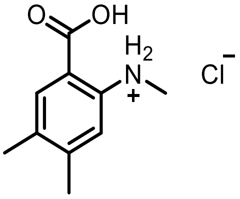

tert-Butyl ((3,4-dimethylbenzoyl)oxy)(methyl)carbamate (140 mg, 0.50 mmol, 1 eq.), TFA (285 mg, 2.50 mmol, 5 eq.) and $FeSO_4 \cdot 7H_2O$ (1.4 mg, 5.04 µmol, 0.01 eq.) were reacted in HFIP (10 mL) according to general procedure C. The crude residue was filtered through a short silica plug eluting first with Pet.Ether:EtOAc (90:10) then EtOAc. The title compound (35.8 mg, 0.17 mmol, 33%) was isolated as an orange solid.

Note: Crude ^{1}H NMR data suggested the formation of the other *ortho* functionalised regioisomer, 3,4-dimethyl-2-(methylamino)benzoic acid. The crude r.r was 2.4:1 with the reported regioisomer being the major regioisomer.

^{1}H NMR (500 MHz, MeOD-d_4) δ 7.97 (s, 1H), 7.38 (s, 1H), 3.09 (s, 3H), 2.41 (s, 3H), 2.36 (s, 3H); ^{13}C NMR (126 MHz, MeOD-d_4) δ 167.8, 145.1, 137.4, 137.3, 132.7, 123.0, 118.4, 36.1, 18.8, 17.9; HRMS (ESI) calcd for $[C_{10}H_{13}NO_2 + H]^+$: 180.1019. Found: 180.1012.

3,5-Dimethyl-2-(methylamino)benzoic acid hydrochloride salt 4i

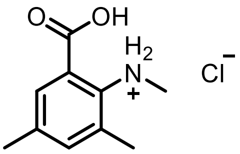

tert-Butyl ((3,5-dimethylbenzoyl)oxy)(methyl)carbamate (140 mg, 0.50 mmol, 1 eq.), TFA (285 mg, 2.50 mmol, 5 eq.) and $FeSO_4{\cdot}7H_2O$ (1.4 mg, 5.04 µmol, 0.01 eq.) were reacted in HFIP (10 mL) according to a modified version of general procedure C. The crude residue was filtered through a short silica plug eluting with EtOAc. The title compound (49.4 mg, 0.23 mmol, 46%) was isolated as a white solid.

^{1}H NMR (400 MHz, MeOD-d_4) δ 7.96 (d, J = 2.0 Hz, 1H), 7.55 (d, J = 2.1 Hz, 1H), 3.12 (s, 3H), 2.56 (s, 3H), 2.43 (s, 3H); ^{13}C NMR (101 MHz, MeOD-d_4) δ 168.7, 140.2, 138.0, 133.97, 132.86, 130.6, 122.1, 36.0, 19.5, 15.7; HRMS (ESI) calcd for $[C_{10}H_{13}NO_2 + H]^+$: 180.1019. Found: 180.1018.

2-Oxindole 6a

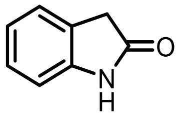

tert-Butyl (2-phenylacetoxy)carbamate (126 mg, 0.50 mmol, 1 eq.), TFA (285 mg, 2.50 mmol, 5 eq.) and $FeSO_4{\cdot}7H_2O$ (1.4 mg, 5.04 µmol, 0.01 eq.) were reacted according to a modified version of general procedure C. The crude residue was purified by silica gel chromatography (eluent: Pet. Ether:EtOAc:AcOH 95:4.5:0.5 – 60:39.5:0.5) to give the title compound (37.5 mg, 0.28 mmol, 56%) as a pale yellow solid.

^{1}H NMR (400 MHz, $CDCl_3$) δ 9.21 (br s, 1H), 7.24 – 7.17 (m, 2H), 7.02 (t, J = 7.5 Hz, 1H), 6.91 (d, J = 7.8 Hz, 1H), 3.55 (s, 2H); ^{13}C NMR (101 MHz, $CDCl_3$) δ 178.3, 142.5, 127.9, 125.3, 124.6, 122.4, 109.9, 36.4.

Data matches literature values.[3]

3-Methyl-2-oxindole 6b

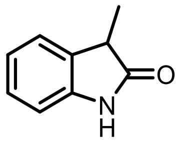

tert-Butyl ((2-phenylpropanoyl)oxy)carbamate (147 mg, 0.55 mmol, 1 eq.), TFA (285 mg, 2.50 mmol, 4.5 eq.) and $FeSO_4 \cdot 7H_2O$ (1.4 mg, 5.04 µmol, 0.01 eq.) were reacted according to a modified version of general procedure C. The crude residue was purified by silica gel chromatography (eluent: Pet. Ether:EtOAc:AcOH 90:9.5:0.5 – 60:39.5:0.5) to give the title compound (38.4 mg, 0.26 mmol, 47%) as a white solid.

^{1}H NMR (400 MHz, $CDCl_3$) δ 9.18 (br s, 1H), 7.23 – 7.15 (m, 2H), 7.03 (td, *J* = 7.4, 1.0 Hz, 1H), 6.93 (d, *J* = 7.5 Hz, 1H), 3.48 (q, *J* = 7.6 Hz, 1H), 1.51 (d, *J* = 7.6 Hz, 3H); ^{13}C NMR (101 MHz, $CDCl_3$) δ 181.8, 141.3, 131.3, 127.9, 123.8, 122.4, 109.8, 41.1, 15.2.

Data matches literature values.[4]

3,3-Dimethyl-2-oxindole 6c

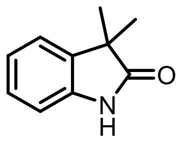

tert-Butyl ((2-methyl-2-phenylpropanoyl)oxy)carbamate (180 mg, 0.64 mmol, 1 eq.), TFA (362 mg, 3.18 mmol, 5 eq.) and $FeSO_4 \cdot 7H_2O$ (1.4 mg, 5.04 µmol, 0.01 eq.) were reacted according to a modified version of general procedure C. The crude residue was purified by silica gel chromatography (eluent: Pet. Ether:EtOAc:AcOH 99.5:0:0.5 – 70:29.5:0.5) to give the title compound (49.6 mg, 0.31 mmol, 48%) as a white solid.

^{1}H NMR (400 MHz, $CDCl_3$) δ 9.45 (br s, 1H), 7.22 – 7.13 (m, 2H), 7.10 – 6.99 (m, 1H), 6.97 (d, *J* = 7.4 Hz, 1H), 1.41 (s, 6H); ^{13}C NMR (101 MHz, $CDCl_3$) δ 184.7, 140.0, 136.3, 127.7, 122.5, 122.5 110.1, 44.8, 24.3.

Data matches literature values.[4]

Spiro[cyclopropane-1,3'-indolin]-2'-one 6d

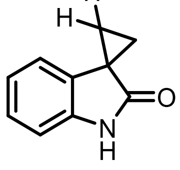

tert-Butyl ((1-phenylcyclopropane-1-carbonyl)oxy)carbamate (147 mg, 0.53 mmol, 1 eq.), TFA (285 mg, 2.50 mmol, 4.7 eq.) and $FeSO_4 \cdot 7H_2O$ (1.4 mg, 5.04 µmol, 0.01 eq.) were reacted according to a modified version of

general procedure C. The crude residue was purified by silica gel chromatography (eluent: Pet. Ether:EtOAc:AcOH 95:4.5:0.5 – 75:24.5:0.5) to give the title compound (40.8 mg, 0.26 mmol, 48%) as a white solid.

^{1}H NMR (400 MHz, $CDCl_3$) δ 9.47 (br s, 1H), 7.21 (td, *J* = 7.7, 1.2 Hz, 1H), 7.09 – 6.97 (m, 2H), 6.85 (dd, *J* = 7.8, 1.2 Hz, 1H), 1.80 (q, *J* = 4.1 Hz, 2H), 1.57 (q, *J* = 4.1 Hz, 2H); ^{13}C NMR (101 MHz, $CDCl_3$) δ 180.0, 140.9, 131.3, 126.7, 122.0, 118.5, 110.0, 27.6, 19.5.

Data matches literature values.[5]

4-Chloro-2-oxindole 6e

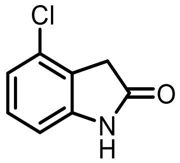

tert-Butyl (2-(2-chlorophenyl)acetoxy)carbamate (158 mg, 0.55 mmol, 1 eq.), TFA (285 mg, 2.50 mmol, 4.5 eq.) and $FeSO_4{\cdot}7H_2O$ (1.4 mg, 5.04 µmol, 0.01 eq.) were reacted according to a modified version of general procedure C. The crude residue was purified by silica gel chromatography (eluent: Pet. Ether:EtOAc:AcOH 95:4.5:0.5 – 75:24.5:0.5) to give the title compound (30.5 mg, 0.18 mmol, 33%) as a yellow solid.

^{1}H NMR (500 MHz, $CDCl_3$) δ 8.53 (br s, 1H), 7.24 – 7.17 (m, 1H), 7.04 (dd, *J* = 8.2, 0.7 Hz, 1H), 6.81 (d, *J* = 7.8 Hz, 1H), 3.58 (s, 2H); ^{13}C NMR (126 MHz, $CDCl_3$) δ 176.4, 143.3, 130.6, 129.3, 123.8, 122.6, 108.0, 35.7; HRMS (ESI) calcd for $[C_8H_6ClNO + H]^+$: 168.0211. Found: 168.0213.

4-Methyl-2-oxindole 6f

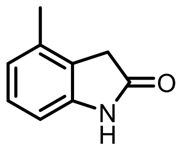

tert-Butyl (2-(*o*-tolyl)acetoxy)carbamate (160 mg, 0.60 mmol, 1 eq.), TFA (340 mg, 2.98 mmol, 5 eq.) and $FeSO_4{\cdot}7H_2O$ (1.4 mg, 5.04 µmol, 0.01 eq.) were reacted according to a modified version of general procedure C. The crude residue was purified by silica gel chromatography (eluent: Pet. Ether:EtOAc:AcOH 95:4.5:0.5 – 75:24.5:0.5) to give the title compound (32.8 mg, 0.22 mmol, 37%) as a white solid.

^{1}H NMR (400 MHz, $CDCl_3$) δ 8.74 (br s, 1H), 7.15 (t, *J* = 7.8 Hz, 1H), 6.87 (d, *J* = 7.7 Hz, 1H), 6.75 (d, *J* = 7.7 Hz, 1H), 3.45 (s, 2H), 2.28 (s, 3H); ^{13}C NMR (101 MHz, $CDCl_3$) δ 177.8, 142.1, 134.3, 127.9, 124.1, 123.5, 107.2, 35.2, 18.6.

Data matches literature values.[6]

4-(Trifluoromethyl)-2-oxindole 6g

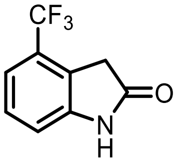

tert-Butyl (2-(2-(trifluoromethyl)phenyl)acetoxy)carbamate (182 mg, 0.57 mmol, 1 eq.), TFA (323 mg, 2.83 mmol, 5 eq.) and $FeSO_4{\cdot}7H_2O$ (1.4 mg, 5.04 µmol, 0.01 eq.) were reacted according to a modified version of general procedure C. The crude residue was purified by silica gel chromatography (eluent: Pet. Ether:EtOAc:AcOH 95:4.5:0.5 – 75:24.5:0.5) to give the title compound (40.4 mg, 0.20 mmol, 35%) as a yellow solid.

^{1}H NMR (400 MHz, MeOD-d_4) δ 7.40 (t, *J* = 7.9 Hz, 1H), 7.26 (d, *J* = 8.0 Hz, 1H), 7.13 (d, *J* = 7.8 Hz, 1H), 3.67 (s, 2H); ^{13}C NMR (101 MHz, MeOD-d_4) δ 177.3, 144.6, 128.4, 126.1, 124.0 (q, *J* = 271.8 Hz), 123.4, 118.5 – 117.5 (m), 112.8, 35.0; ^{19}F NMR (471 MHz, $CDCl_3$) δ -63.64; HRMS (ESI) calcd for $[C_9H_6F_3NO + H]^+$: 202.0474. Found: 202.0470.

5,7-Dimethyl-2-oxindole 6h

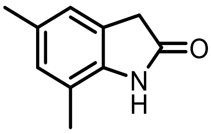

tert-Butyl (2-(3,5-dimethylphenyl)acetoxy)carbamate (140 mg, 0.50 mmol, 1 eq.), TFA (285 mg, 2.50 mmol, 5 eq.) and $FeSO_4{\cdot}7H_2O$ (1.4 mg, 5.04 µmol, 0.01 eq,) were reacted according to a modified version of general procedure C. The crude residue was purified by silica gel chromatography (eluent: Pet. Ether:EtOAc:AcOH 95:4.5:0.5 – 70:29.5:0.5) to give the title compound (40.2 mg, 0.25 mmol, 50%) as a white solid.

^{1}H NMR (400 MHz, $CDCl_3$) δ 9.14 (br s, 1H), 6.91 (s, 1H), 6.87 (s, 1H), 3.55 (s, 2H), 2.31 (s, 3H), 2.28 (s, 3H); ^{13}C NMR (101 MHz, $CDCl_3$) δ 178.3, 138.9, 131.7, 129.6, 125.0, 122.7, 118.9, 36.7, 21.0, 16.4. HRMS (ESI) calcd for $[C_{10}H_{11}NO + H]^+$: 162.0913. Found: 162.0914.

5-Bromo-2-oxindole 6i and 7-Bromo-2-oxindole *iso*-6i

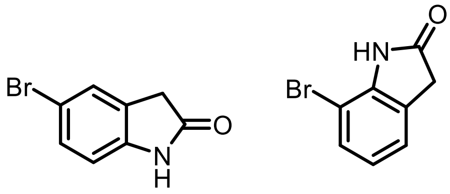

6i **iso-6i**

tert-Butyl (2-(3-bromophenyl)acetoxy)carbamate (165 mg, 0.50 mmol, 1 eq.), TFA (285 mg 2.50 mmol, 5 eq.) and $FeSO_4 \cdot 7H_2O$ (1.4 mg, 5.04 µmol, 0.01 eq.) were reacted according to a modified version of general procedure C The crude residue was purified by silica gel chromatography (eluent: Pet. Ether:EtOAc:AcOH 95:4.5:0.5 – 70:29.5:0.5) to give the title compound **6i** (45.2 mg, 0.21 mmol, 42%) and the title compound ***iso*-6i** (26.4 mg, 0.12 mmol, 25%) as yellow solids with a total yield of 67% and a regioisomer ratio of 1.7:1 (crude ratio 1.8:1).

5-Bromo-2-oxindole 6i

^{1}H NMR (400 MHz, DMSO-d_6) δ 10.48 (br s, 1H), 7.38 (d, *J* = 1.8 Hz, 1H), 7.34 (dd, *J* = 8.3, 2.0 Hz, 1H), 6.76 (d, *J* = 8.2 Hz, 1H), 3.51 (s, 2H); ^{13}C NMR (101 MHz, MeOD-d_4) δ 177.9, 142.4, 130.3, 128.1, 127.3, 114.1, 110.8, 35.6.

Data matches literature values.[7]

7-Bromo-2-oxindole *iso*-6i

^{1}H NMR (400 MHz, MeOD-d_4) δ 7.36 (dd, *J* = 8.2, 1.0 Hz, 1H), 7.22 (dd, *J* = 7.4, 1.2 Hz, 1H), 6.93 (t, *J* = 7.8 Hz, 1H), 3.64 (s, 2H); ^{13}C NMR (101 MHz, MeOD-d_4) δ 177.5, 142.6, 130.4, 127.3, 123.1, 123.1, 101.8, 36.5. HRMS (ESI) calcd for $[C_8H_6BrNO + H]^+$: 211.9706. Found 211.9711.

6-Bromo-3,4-dihydroquinolin-2(1*H*)-one 6j and 8-bromo-3,4-dihydroquinolin-2(1*H*)-one *iso*-6j

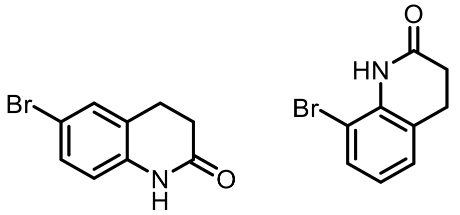

6j **iso-6j**

tert-Butyl ((3-(3-bromophenyl)propanoyl)oxy)carbamate (181 mg, 0.53 mmol, 1 eq.), TFA (285 mg, 2.50 mmol, 5 eq.) and $FeSO_4 \cdot 7H_2O$ (1.4 mg, 5.04 µmol, 0.01 eq.) were reacted according to a modified version of general procedure C. The crude residue was purified by silica gel chromatography (eluent: Pet. Ether:EtOAc:AcOH

95:4.5:0.5 – 60:39.5:0.5) to give the title compound **6j** (38.8 mg, 0.17 mmol, 33%) and the title compound ***iso*-6j** (28.9 mg, 0.13 mmol, 24%) as brown and white solids, respectively, with a total yield of 57% and a regioisomer ratio of 1.3:1 (crude ratio 1.2:1).

6-Bromo-3,4-dihydroquinolin-2(1*H*)-one 6j

^{1}H NMR (400 MHz, $CDCl_3$) δ 9.08 (br s, 1H), 7.32 – 7.26 (m, 2H), 6.71 (d, *J* = 8.1 Hz, 1H), 2.95 (dd, *J* = 8.5, 6.7 Hz, 2H), 2.63 (dd, *J* = 8.5, 6.6 Hz, 2H); ^{13}C NMR (101 MHz, $CDCl_3$) δ 171.8, 136.4, 130.8, 130.4, 125.7, 117.0, 115.5, 30.3, 25.1.

Data matches literature values.[4]

8-Bromo-3,4-dihydroquinolin-2(1*H*)-one *iso*-6j

^{1}H NMR (400 MHz, $CDCl_3$) δ 7.90 (br s, 1H), 7.42 (d, *J* = 8.0 Hz, 1H), 7.14 (d, *J* = 7.4 Hz, 1H), 6.89 (t, *J* = 7.8 Hz, 1H), 3.01 (t, *J* = 7.6 Hz, 2H), 2.66 (t, *J* = 7.6 Hz, 2H); ^{13}C NMR (101 MHz, $CDCl_3$) δ 170.7, 135.1, 130.9, 127.1, 125.6, 123.8, 109.7, 30.6, 26.0.

Data matches literature values.[8]

6,8-Dimethoxy-3,4-dihydroquinolin-2(1*H*)-one 6k

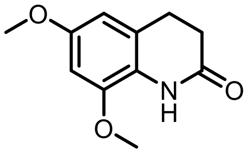

tert-Butyl ((3-(3,5-dimethoxyphenyl)propanoyl)oxy)carbamate (170 mg, 0.52 mmol, 1 eq.), TFA (285 mg, 2.50 mmol, 4.8 eq.) and $FeSO_4 \cdot 7H_2O$ (1.4 mg, 5.04 µmol, 0.01 eq.) were reacted according to a modified version of general procedure C. The crude residue was purified by silica gel chromatography (eluent: Pet. Ether:EtOAc:AcOH 95:4.5:0.5 – 60:39.5:0.5) to give the title compound (67.2 mg, 0.32 mmol, 62%) as a yellow solid.

^{1}H NMR (500 MHz, MeOD-d_4) δ 6.46 (d, *J* = 2.5 Hz, 1H), 6.38 (d, *J* = 2.5 Hz, 1H), 3.86 (s, 3H), 3.77 (s, 3H), 2.94 – 2.86 (m, 2H), 2.58 – 2.51 (m, 2H); ^{13}C NMR (126 MHz, MeOD-d_4) δ 171.5, 156.4, 147.4, 125.1, 119.5, 103.7, 96.9, 54.9, 54.6, 30.2, 25.2; HRMS (ESI) calcd for $[C_{11}H_{13}NO_3 + H]^+$: 208.0968. Found: 208.0970.

7-Methyl-3,4-dihydroquinolin-2(1*H*)-one 6l and 3-(3-amino-4-methylphenyl)propanoic acid 6m

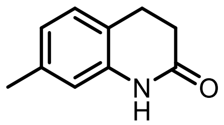

6l

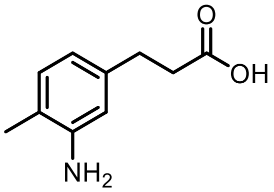

6m

tert-Butyl ((3-(*p*-tolyl)propanoyl)oxy)carbamate (140 mg, 0.50 mmol, 1 eq.), TFA (285 mg, 2.50 mmol, 5 eq.) and $FeSO_4 \cdot 7H_2O$ (1.4 mg, 5.04 µmol, 0.01 eq.) were reacted according to a modified version of general procedure C. The crude residue was purified by silica gel chromatography (eluent: Pet. Ether:EtOAc:AcOH 95:4.5:0.5 – 70:29.5:0.5) to give the title compound 6l (24.4 mg, 0.15 mmol, 30%) and the title compound 6m (29.3 mg, 0.16 mmol, 33%) as white and brown solids, respectively, with a total yield of 63% and an isolated regioisomer ratio of 1:1.1.

7-Methyl-3,4-dihydroquinolin-2(1*H*)-one 6l

^{1}H NMR (500 MHz, $CDCl_3$) δ 9.20 (br s, 1H), 7.03 (d, *J* = 7.6 Hz, 1H), 6.80 (dd, *J* = 7.6, 0.9 Hz, 1H), 6.66 (s, 1H), 2.92 (t, *J* = 7.6 Hz, 2H), 2.67 – 2.59 (m, 2H), 2.30 (s, 3H); ^{13}C NMR (126 MHz, $CDCl_3$) δ 172.6, 137.5, 137.1, 127.7, 123.8, 120.6, 116.2, 30.9, 24.9, 21.1.

Data matches literature values.[4]

3-(3-Amino-4-methylphenyl)propanoic acid 6m

^{1}H NMR (400 MHz, $CDCl_3$) δ 6.98 (d, *J* = 7.5 Hz, 1H), 6.57 (dd, *J* = 7.6, 1.7 Hz, 1H), 6.55 (d, *J* = 1.6 Hz, 1H), 6.22 (br s, 3H), 2.85 (t, *J* = 7.8 Hz, 2H), 2.64 (dd, *J* = 8.6, 7.1 Hz, 2H), 2.14 (s, 3H); ^{13}C NMR (101 MHz, $CDCl_3$) δ 179.0, 144.3, 139.1, 130.6, 120.6, 118.7, 115.1, 35.7, 30.3, 17.0. HRMS (ESI) calcd for $[C_{10}H_{13}NO_2 + H]^+$: 180.1019. Found: 180.1012.

8 Reaction Utility

Telescoped procedure – 2-Aminobenzoic acid 2a

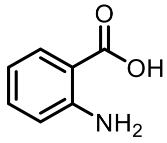

Under an atmosphere of nitrogen, EDC hydrochloride (42.2 mg, 0.22 mmol, 1.1 eq.) was added to a suspension of benzoic acid (24.4 mg, 0.20 mmol, 1 eq.), *tert*-butyl *N*-hydroxycarbamate (26.6 mg, 0.20 mmol, 1 eq.) and DMAP (2.5 mg, 0.02 mmol, 0.1 eq.) in dry CH_2Cl_2 (1 mL) at 0 ℃. The reaction was stirred at room temperature for 16 h. The reaction was then diluted with aqueous NH_4Cl (5 mL) and the aqueous layer was extracted with $CDCl_3$ (3 x 3 mL). The combined organics were then dried ($MgSO_4$) and concentrated *in vacuo*. TFE (4 mL), $FeSO_4{\cdot}7H_2O$ (0.6 mg, 2.16 µmol, 0.01 eq.) and TFA (113 mg, 0.99 mmol, 5 eq.) were added sequentially to the resulting crude residue. The resulting solution was stirred at 40 ℃ for 16 h and then concentrated under a stream of air. The crude residue was purified by silica gel chromatography (eluent: Pet. Ether:EtOAc:AcOH 99.5:0:0.5 – 80:19.5:0.5) to give the title compound (15.8 mg, 0.12 mmol, 58%) as a white solid.

^{1}H NMR (400 MHz, DMSO-*d*$_6$) δ 8.58 (br s, 3H), 7.69 (dd, *J* = 8.0, 1.6 Hz, 1H), 7.22 (ddd, *J* = 8.5, 6.8, 1.7 Hz, 1H), 6.73 (d, *J* = 8.2 Hz, 1H), 6.50 (t, *J* = 7.5 Hz, 1H); ^{13}C NMR (101 MHz, DMSO-*d*$_6$) δ 170.0, 151.9, 134.2, 131.6, 116.8, 115.0, 110.0.

Data matches that of a commercial sample.

Scaled up procedure – 2-Aminobenzoic acid 2a

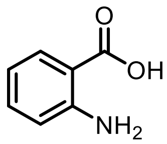

TFA (2.85 g, 25.0 mmol, 5 eq.) was added to a solution of *tert*-butyl (benzoyloxy)carbamate (1.19 g, 5.02 mmol, 1 eq.) and $FeSO_4{\cdot}7H_2O$ (13.9 mg, 0.050 mmol, 0.01 eq.) in TFE (100 mL). The reaction was stirred at 40 ℃ for 16 h. The reaction was then concentrated *in vacuo* and the crude residue purified by silica gel chromatography (eluent: Pet. Ether:EtOAc:AcOH 99.5:0:0.5 – 80:19.5:0.5) to give the title compound (543 mg, 3.96 mmol, 79%) as a pale pink solid.

^{1}H NMR (500 MHz, MeOD-d_4) δ 7.90 (dd, *J* = 8.0, 1.6 Hz, 1H), 7.34 (ddd, *J* = 8.5, 7.1, 1.7 Hz, 1H), 6.90 (dd, *J* = 8.3, 1.1 Hz, 1H), 6.78 (ddd, *J* = 8.2, 7.2, 1.2 Hz, 1H); ^{13}C NMR (126 MHz, MeOD-d_4) δ 169.6, 147.7, 133.7, 131.4, 117.9, 117.9, 112.9.

Data matches that of a commercial sample.

Quinazolin-4(3*H*)-one 7

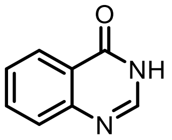

tert-Butyl (benzoyloxy)carbamate (47.4 mg, 0.20 mmol, 1 eq.), TFA (133 mg, 0.99 mmol, 5 eq.) and $FeSO_4 \cdot 7H_2O$ (0.6 mg, 2.16 µmol, 0.01 eq.) were reacted according to a modified version of general procedure C. The reaction was concentrated under a stream of air and then filtered through a silica plug (eluent: EtOAc). The filtrate was concentrated under a stream of air and, under an atmosphere of nitrogen, the resulting crude residue was dissolved in dry MeOH (1 mL). Ammonium acetate (77.0 mg, 1.00 mmol, 5 eq.) and trimethylorthoformate (106 mg, 1.00 mmol, 5 eq.) were added and the reaction was stirred at 95 ℃ for 14 h. The solvent was then removed under a stream of air. The resulting residue was then dissolved in CH_2Cl_2 (3 mL) and saturated aqueous Na_2CO_3 solution (5 mL) was added. The aqueous layer was extracted with CH_2Cl_2 (3 x 5 mL) and the combined organics were then dried ($MgSO_4$), filtered and concentrated *in vacuo*. The crude residue was purified by silica gel chromatography (eluent: Pet. Ether:EtOAc 50:50 – 0:100) to give the title compound (16.1 mg, 0.11 mmol, 55%) as a white solid.

^{1}H NMR (500 MHz, $CDCl_3$) δ 8.33 (d, *J* = 8.1 Hz, 1H), 8.17 (s, 1H), 7.88 – 7.75 (m, 2H), 7.56 (t, *J* = 7.4 Hz, 1H); ^{13}C NMR (126 MHz, $CDCl_3$) δ 163.2, 148.9, 143.6, 135.0, 127.8, 127.5, 126.4, 122.5.

Data matches literature values.[13]

2-(*p*-Tolyl)-4*H*-benzo[d][1,3]oxazin-4-one 8

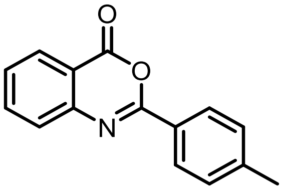

tert-Butyl (benzoyloxy)carbamate (47.4 mg, 0.20 mmol, 1 eq.), TFA (133 mg, 0.99 mmol, 5 eq.) and $FeSO_4 \cdot 7H_2O$ (0.6 mg, 0.002 mmol, 0.01 eq.) were reacted according to a modified version of general procedure C. The reaction was concentrated under a stream of air and then filtered through a silica plug (eluent: EtOAc). The filtrate was concentrated under a stream of air. Under an atmosphere of nitrogen, the crude residue was

dissolved in dry THF (0.4 mL). Na_2CO_3 (42.4 mg, 0.4 mmol, 2 eq.) and *p*-toluoyl chloride (61.8 mg, 0.40 mmol, 2 eq.) were added sequentially at 0 ℃. The reaction was stirred for 24 h at room temperature. The solvent was removed under a stream of air. The resulting residue was then dissolved in CH_2Cl_2 (3 mL) and saturated aqueous Na_2CO_3 (5 mL) was added. The aqueous layer was then extracted with CH_2Cl_2 (3 x 5 mL) and the combined organics were then dried ($MgSO_4$), filtered and concentrated *in vacuo*. The crude residue was purified by silica gel chromatography (eluent: Pet. Ether:EtOAc 100:0 – 95:5) to give the title compound (27.2 mg, 0.11 mmol, 57%) as a white solid.

^{1}H NMR (400 MHz, $CDCl_3$) δ 8.25 (dd, *J* = 8.0, 1.5 Hz, 1H), 8.21 (d, *J* = 8.3 Hz, 2H), 7.83 (ddd, *J* = 8.7, 7.3, 1.6 Hz, 1H), 7.69 (dd, *J* = 8.1, 1.1 Hz, 1H), 7.57 – 7.47 (m, 1H), 7.33 (d, *J* = 8.0 Hz, 2H), 2.46 (s, 3H); ^{13}C NMR (101 MHz, $CDCl_3$) δ 159.7, 157.3, 147.2, 143.4, 136.5, 129.5, 128.6, 128.3, 128.0, 127.5, 127.1, 116.9, 21.7.

Data matches literature values.[14]

9 Mechanistic Experiments

9.1 Crossover Experiments

Authentic Neutral Isomer Syntheses

3,5-Dimethyl-2-(methylamino)benzoic acid 4i

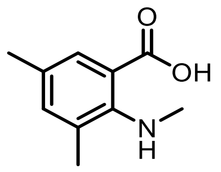

tert-Butyl ((3,5-dimethylbenzoyl)oxy)(methyl)carbamate (140 mg, 0.50 mmol, 1 eq.), TFA (285 mg, 2.50 mmol, 5 eq.) and $FeSO_4 \cdot 7H_2O$ (1.4 mg, 5.04 µmol, 0.01 eq.) were reacted in HFIP (10 mL) according to a modified version of general procedure C. The crude residue purified by silica gel chromatography (eluent: CH_2Cl_2:EtOAc:AcOH 80:19.5:0.5 then CH_2Cl_2:MeOH:AcOH 98:1.5:0.5 – 92:7.5:0.5). The title compound (71.2 mg, 0.40 mmol, 80%) was isolated as a white solid.

^{1}H NMR (400 MHz, MeOD-d_4) δ 7.85 (s, 1H), 7.33 (s, 1H), 2.93 (s, 3H), 2.46 (s, 3H), 2.37 (s, 3H); ^{13}C NMR (101 MHz, MeOD-d_4) δ 170.4*, 138.0, 136.8, 135.7, 132.0, 129.9, 126.0, 34.7, 19.5, 15.5; HRMS (ESI) calcd for $[C_{10}H_{13}NO_2 + H]^+$: 180.1019. Found: 180.1020. *Peak only visible by HMBC.

2-Amino-3,5-dimethylbenzoic acid 2f

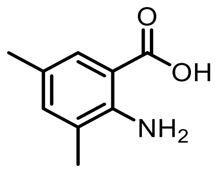

tert-Butyl ((3,5-dimethylbenzoyl)oxy)carbamate (133 mg, 0.50 mmol, 1 eq.), TFA (285 mg, 2.5 mmol, 5 eq.) and $FeSO_4 \cdot 7H_2O$ (1.4 mg, 5.04 µmol, 0.01 eq.) were reacted according to a modified version of general procedure C. The crude residue purified by silica gel chromatography (eluent: Pet.Ether:EtOAc:AcOH 95:4.5:0.5 – 75:24.5:0.5) The title compound (58.3 mg, 0.35 mmol, 71%) was isolated as a white solid.

^{1}H NMR (500 MHz, MeOD-d_4) δ 7.57 (d, *J* = 2.3 Hz, 1H), 7.04 (d, *J* = 2.2 Hz, 1H), 2.20 (s, 3H), 2.15 (s, 3H); ^{13}C NMR (126 MHz, MeOD-d_4) δ 170.6, 146.8, 135.6, 128.9, 124.4, 123.7, 110.5, 19.0, 16.2; HRMS (ESI) calcd for $[C_9H_{11}NO_2 + H]^+$: 166.0863. Found: 166.0862.

4-Methyl-2-(methylamino)benzoic acid 4j

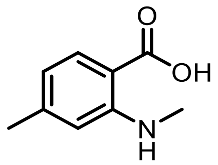

tert-Butyl methyl((4-methylbenzoyl)oxy)carbamate (133 mg, 0.50 mmol, 1 eq.), TFA (285 mg, 2.50 mmol, 5 eq.) and $FeSO_4{\cdot}7H_2O$ (1.4 mg, 5.04 µmol, 0.01 eq.) were reacted according to a modified version of general procedure C. The crude residue purified by silica gel chromatography (eluent: Pet.Ether:EtOAc:AcOH 99.5:0:0.5 – 80:19.5:0.5) The title compound (12.3 mg, 0.069 mmol, 14%) was isolated as a yellow solid.

^{1}H NMR (500 MHz, MeOD-d_4) δ 7.77 (d, *J* = 8.1 Hz, 1H), 6.52 (s, 1H), 6.41 (dd, *J* = 8.1, 1.6 Hz, 1H), 2.89 (s, 3H), 2.32 (s, 3H); ^{13}C NMR (126 MHz, MeOD-d_4) δ 170.6, 152.2, 145.2, 131.7, 115.3, 110.5, 107.6, 28.2, 20.8; HRMS (ESI) calcd for $[C_9H_{11}NO_2 + H]^+$: 166.0859. Found: 166.0862.

2-Amino-4-methylbenzoic acid 2b

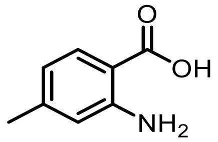

tert-Butyl ((4-methylbenzoyl)oxy)carbamate (126 mg, 0.50 mmol, 1 eq.), TFA (285 mg, 2.50 mmol, 5 eq.) and $FeSO_4{\cdot}7H_2O$ (1.4 mg, 5.04 µmol, 0.01 eq.) were reacted according to a modified version of general procedure C. The crude residue purified by silica gel chromatography (eluent: Pet.Ether:EtOAc:AcOH 95:4.5:0.5 – 75:24.5:0.5) The title compound (8.8 mg, 0.058 mmol, 12%) was isolated as a yellow solid.

^{1}H NMR (500 MHz, MeOD-d_4) δ 7.70 (d, *J* = 8.2 Hz, 1H), 6.57 (s, 1H), 6.43 (dd, *J* = 8.2, 1.0 Hz, 1H), 2.25 (s, 3H); ^{13}C NMR (126 MHz, MeOD-d_4) δ 170.2, 151.4, 144.4, 131.3, 116.7, 116.4, 108.0, 20.3; HRMS (ESI) calcd for $[C_8H_9NO_2 + H]^+$: 152.0706. Found: 152.0709.

3,5-Dimethoxy-2-(methylamino)benzoic acid 4b

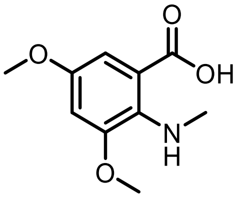

tert-Butyl ((3,5-dimethoxybenzoyl)oxy)(methyl)carbamate (43 mg, 0.15 mmol, 1 eq.), TFA (84.9 mg, 0.74 mmol, 5 eq.) and $FeSO_4{\cdot}7H_2O$ (0.4 mg, 1.44 µmol, 0.01 eq.) were reacted according to a modified version of general procedure C. The crude residue purified by silica gel chromatography (eluent: CH_2Cl_2:MeOH:AcOH 98:1.5:0.5 – 92:7.5:0.5) The title compound (25.9 mg, 0.12 mmol, 80%) was isolated as a yellow solid.

^{1}H NMR (500 MHz, MeOD-d_4) δ 7.28 (s, 1H), 6.86 (s, 1H), 3.98 (s, 3H), 3.86 (s, 3H), 2.94 (s, 3H); ^{13}C NMR (126 MHz, MeOD-d_4) δ 169.8, 160.5, 154.0, 129.1, 120.2, 106.4, 102.3, 55.7, 54.9, 34.6; HRMS (ESI) calcd for $[C_{10}H_{13}NO_4 + H]^+$: 212.0917. Found: 212.0913.

2-Amino-3,5-dimethoxybenzoic acid 2ac

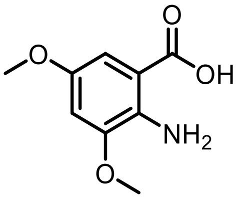

tert-Butyl ((3,5-dimethoxylbenzoyl)oxy)carbamate (149 mg, 0.50 mmol, 1 eq.), TFA (285 mg, 2.50 mmol, 5 eq.) and $FeSO_4{\cdot}7H_2O$ (1.4 mg, 5.04 µmol, 0.01 eq.) were reacted according to a modified version of general procedure C. The crude residue purified by silica gel chromatography (eluent: Pet.Ether:EtOAc:AcOH 95:4.5:0.5 – 70:29.5:0.5) The title compound (69.5 mg, 0.33 mmol, 66%) was isolated as a brown solid.

^{1}H NMR (500 MHz, MeOD-d_4) δ 7.10 (d, J = 2.7 Hz, 1H), 6.80 (d, J = 2.7 Hz, 1H), 3.93 (s, 3H), 3.81 (s, 3H); ^{13}C NMR (126 MHz, MeOD-d_4) δ 169.0, 154.3, 150.9, 126.9, 116.3, 104.7, 103.6, 55.4, 54.8; HRMS (ESI) calcd for $[C_9H_{11}NO_4 + H]^+$: 198.0761. Found: 198.0760.

Crossover Experiment 1

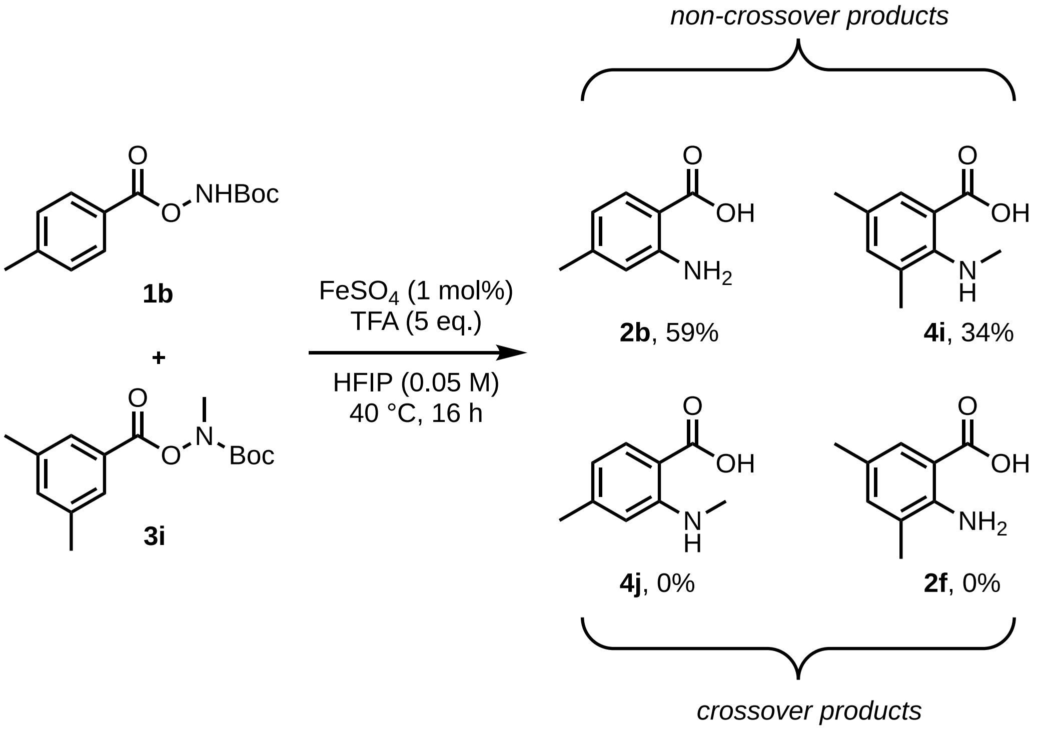

A solution of *tert*-Butyl ((4-methylbenzoyl)oxy)carbamate (**1b**, 25.1 mg, 0.10 mmol, 1 eq.), *tert*-butyl ((3,5-dimethylbenzoyl)oxy)(methyl)carbamate (**3i**, 27.9 mg, 0.10 mmol, 1 eq.), $FeSO_4 \cdot 7H_2O$ (0.6 mg, 2.16 µmol, 0.02 eq.) and TFA (113 mg, 0.99 mmol, 10 eq.) in HFIP (4 mL) was heated at 40 ℃ for 16 h. The reaction was concentrated under a stream of air and the resulting residue was filtered through a plug of silica (eluent: EtOAc). The crude residue was then analysed by ^{1}H NMR and the product distribution analysed. Non-cross over products **2b** and **4i** were observed in 59% and 34% NMR yield, respectively. Crossover products **4j** and **2f** were not observed. Product identity was confirmed by synthesis of all the authentic products (see above).

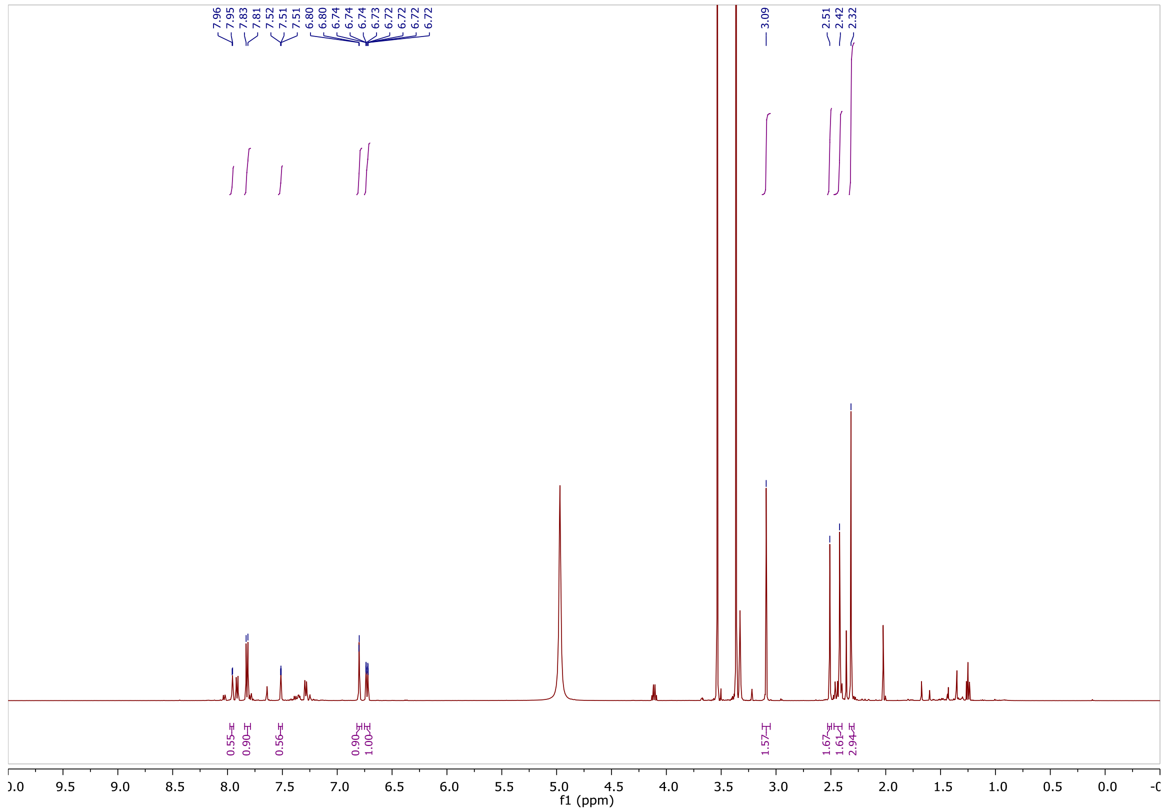
7.96
7.95
7.83
7.81
7.52
7.51
7.51
6.80
6.80
6.74
6.74
6.73
6.72
6.72
6.72
3.09
2.51
2.42
2.32
0.55
0.90
0.56
0.90
1.00
1.57
1.67
1.61
2.94
f1 (ppm)

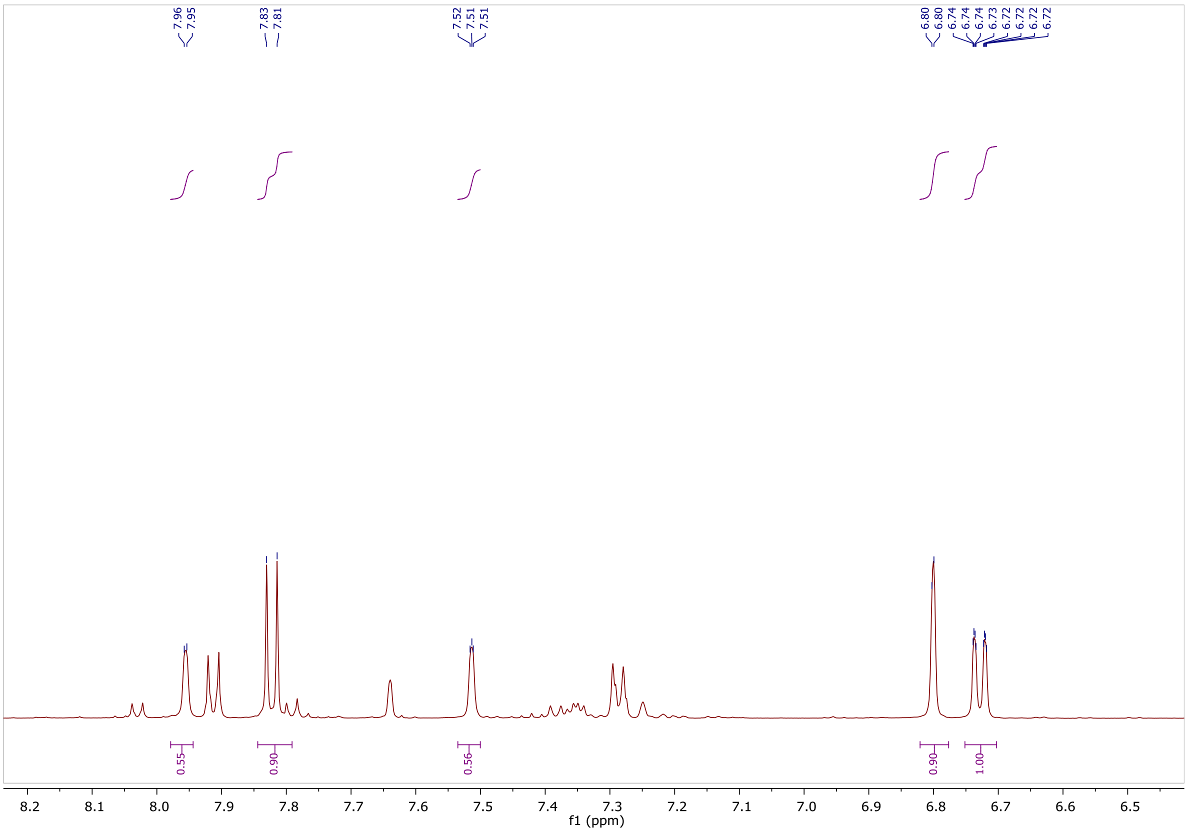
7.96
7.95
7.83
7.81
7.52
7.51
7.51
6.80
6.80
6.74
6.74
6.73
6.72
6.72
6.72
0.55
0.90
0.56
0.90
1.00
f1 (ppm)

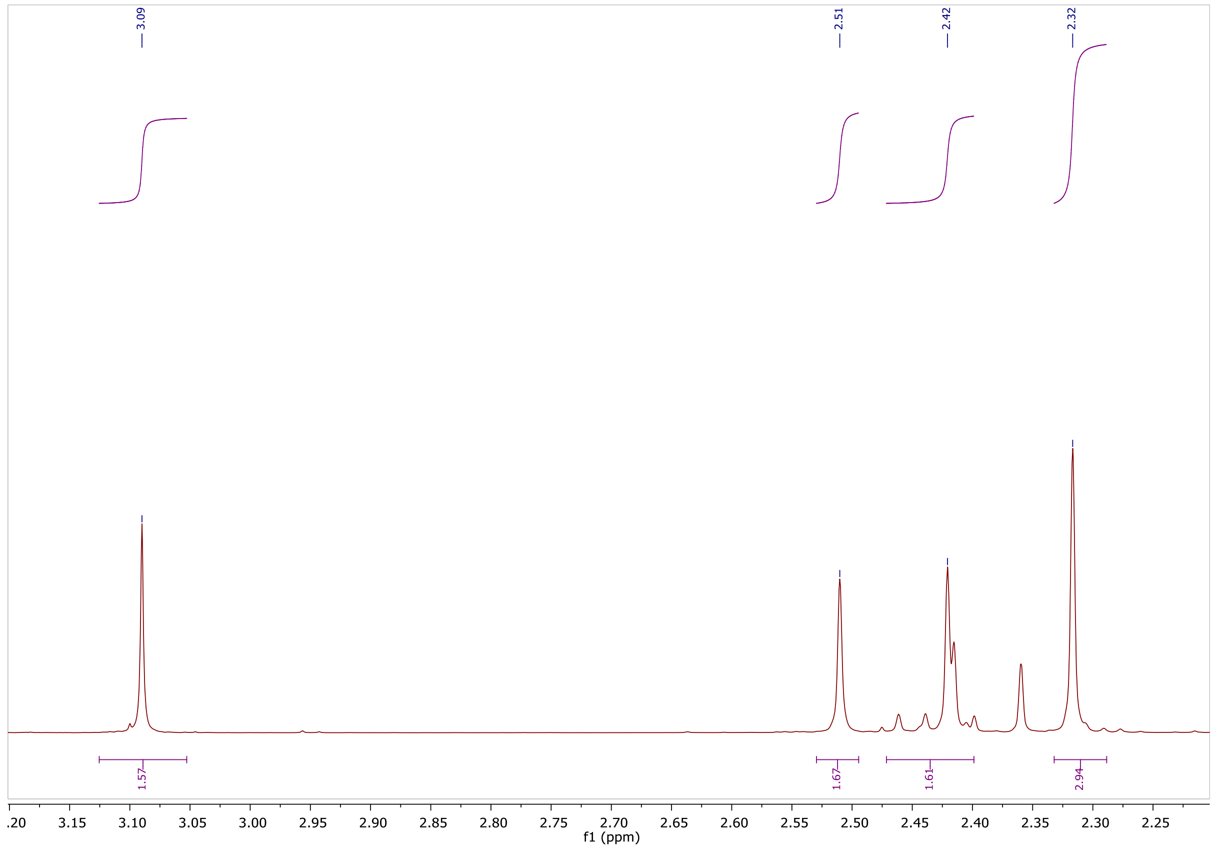
3.09
2.51
2.42
2.32
1.57
1.67
1.61
2.94
f1 (ppm)

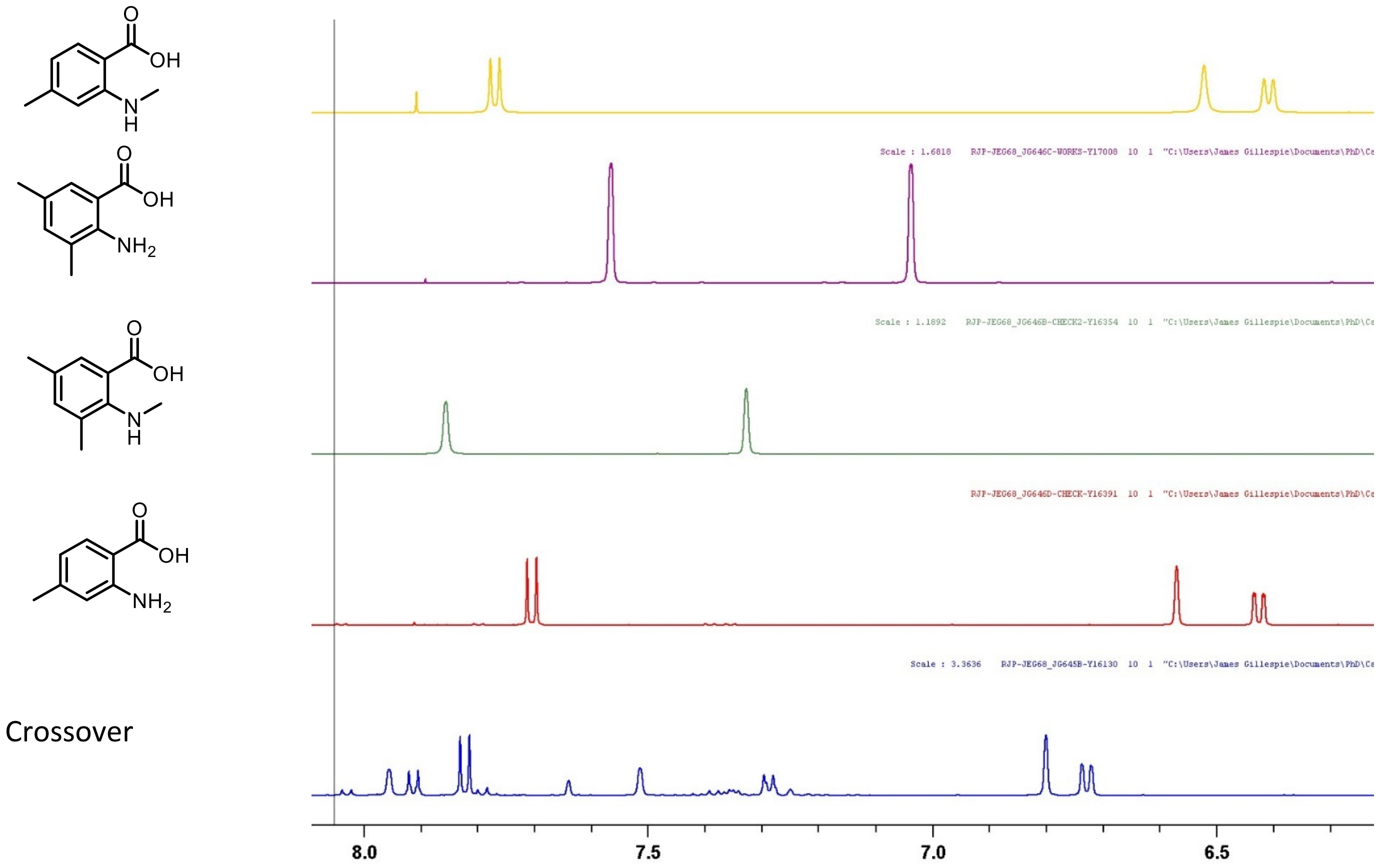
OH
NH2
Crossover
Scale : 1.6818 RJP-JEG68_JG646C-WORKS-Y17008 10 1 "C:\Users\James Gillespie\Documents\PhD\Cr
Scale : 1.1892 RJP-JEG68_JG646B-CHECK2-Y16354 10 1 "C:\Users\James Gillespie\Documents\PhD\Cr
RJP-JEG68_JG646D-CHECK-Y16391 10 1 "C:\Users\James Gillespie\Documents\PhD\Cr
Scale : 3.3636 RJP-JEG68_JG645B-Y16130 10 1 "C:\Users\James Gillespie\Documents\PhD\Cr
8.0
7.5
7.0
6.5

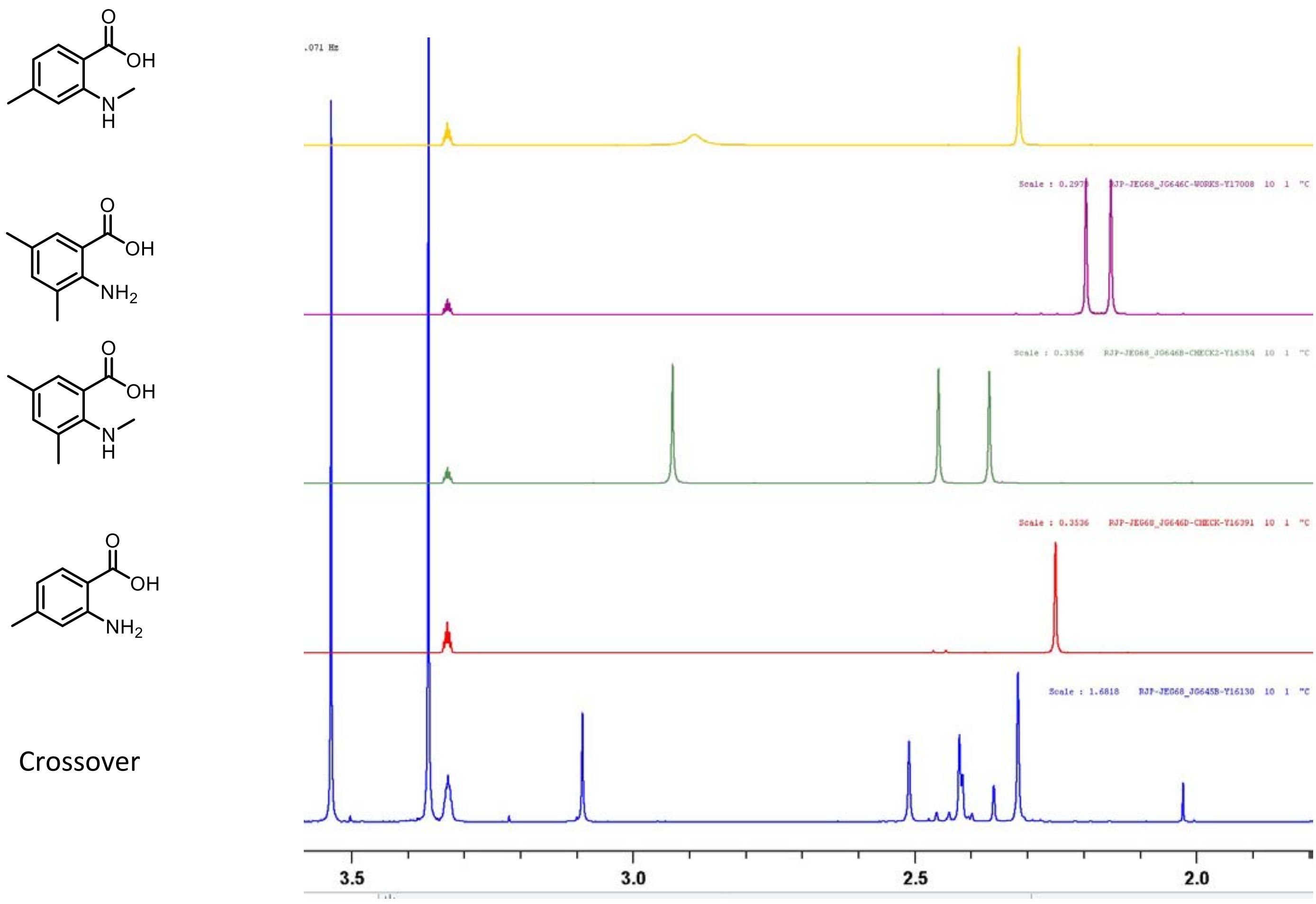
OH
N
H
NH2
OH
NH2
Crossover
3.5
3.0
2.5
2.0

Crossover Experiment 2

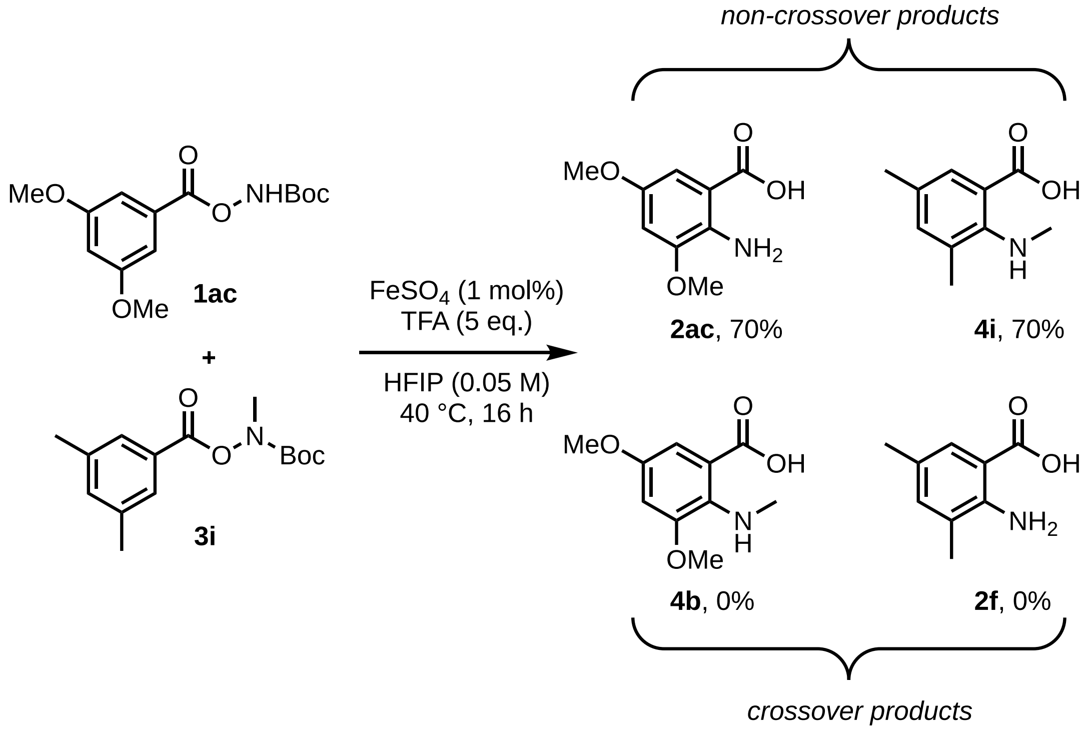

A solution of *tert*-Butyl ((3,5-dimethoxybenzoyl)oxy)carbamate (**1ac**, 29.7 mg, 0.10 mmol, 1 eq.), *tert*-butyl ((3,5-dimethylbenzoyl)oxy)(methyl)carbamate (**3i**, 27.9 mg, 0.10 mmol, 1 eq.), $FeSO_4 \cdot 7H_2O$ (0.6 mg, 2.16 µmol, 0.02 eq.) and TFA (113 mg, 0.99 mmol, 10 eq.) in HFIP (4 mL) was heated at 40 ℃ for 16 h. The reaction was concentrated under a stream of air and the resulting residue was filtered through a plug of silica (eluent: CH_2Cl_2:MeOH 90:10). The crude residue was then analysed by ^{1}H NMR and the product distribution analysed. Non-cross over products **2ac** and **4i** were both observed in 70% NMR yield. Crossover products **4b** and **2f** were not observed. Product identity was confirmed by synthesis of all the authentic products (see above).

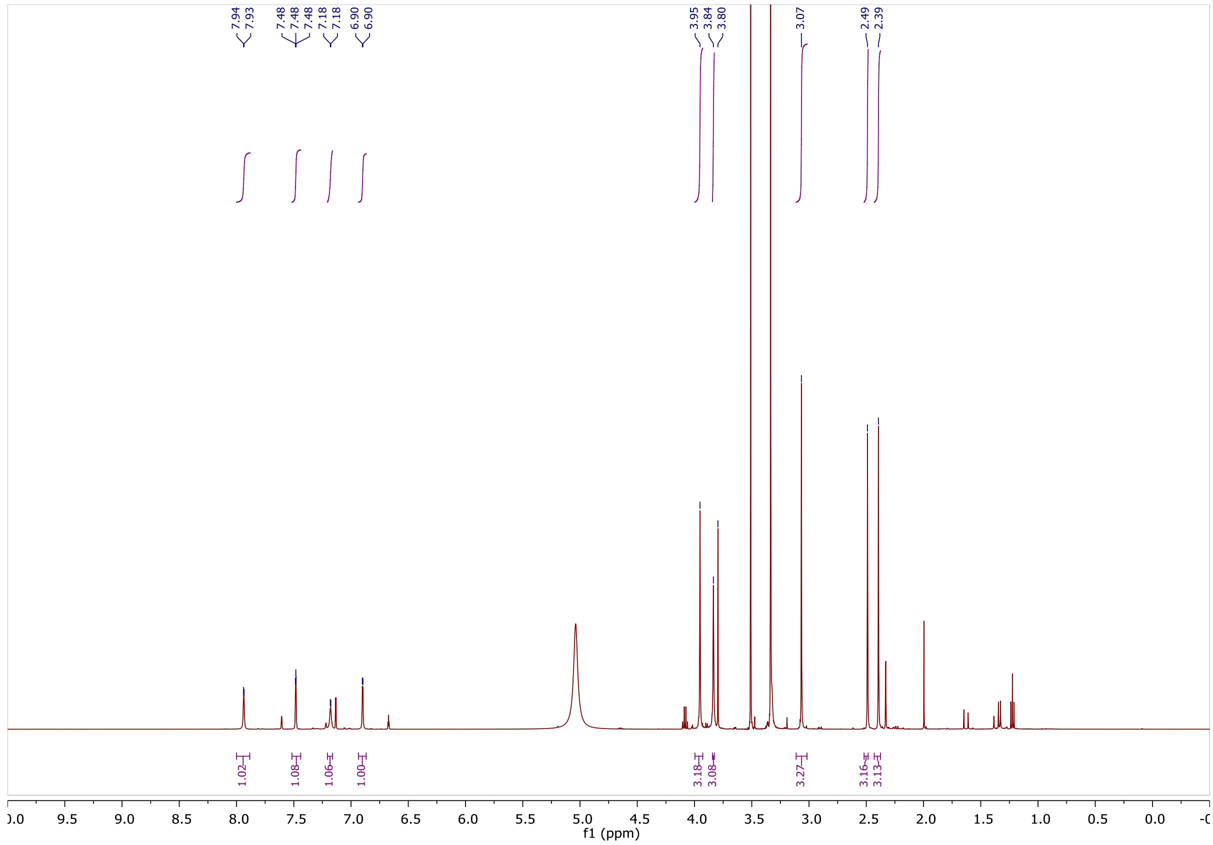
7.94
7.93
7.48
7.48
7.48
7.18
7.18
6.90
6.90
3.95
3.84
3.80
3.07
2.49
2.39
1.02
1.08
1.06
1.00
3.18
3.08
3.27
3.16
3.13
f1 (ppm)

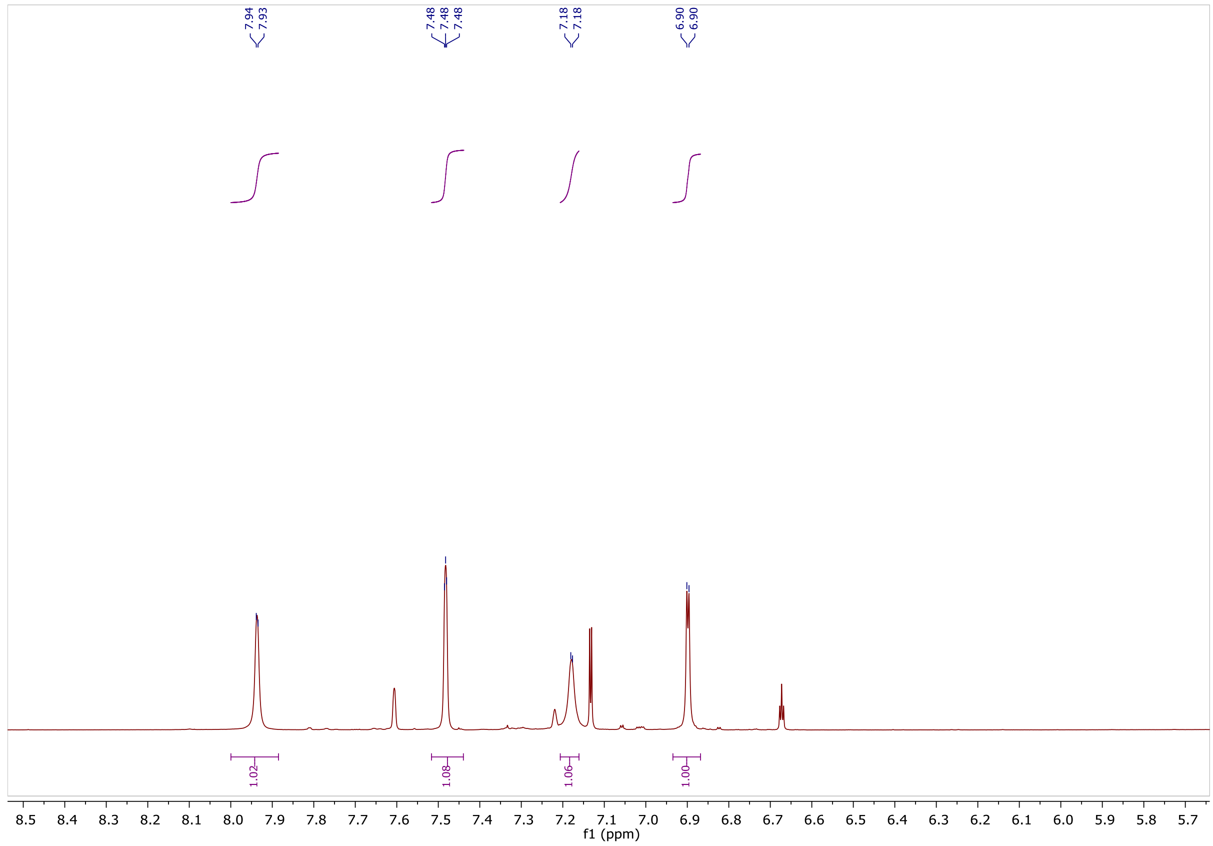
7.94
7.93
7.48
7.48
7.48
7.18
7.18
6.90
6.90
1.02
1.08
1.06
1.00
f1 (ppm)

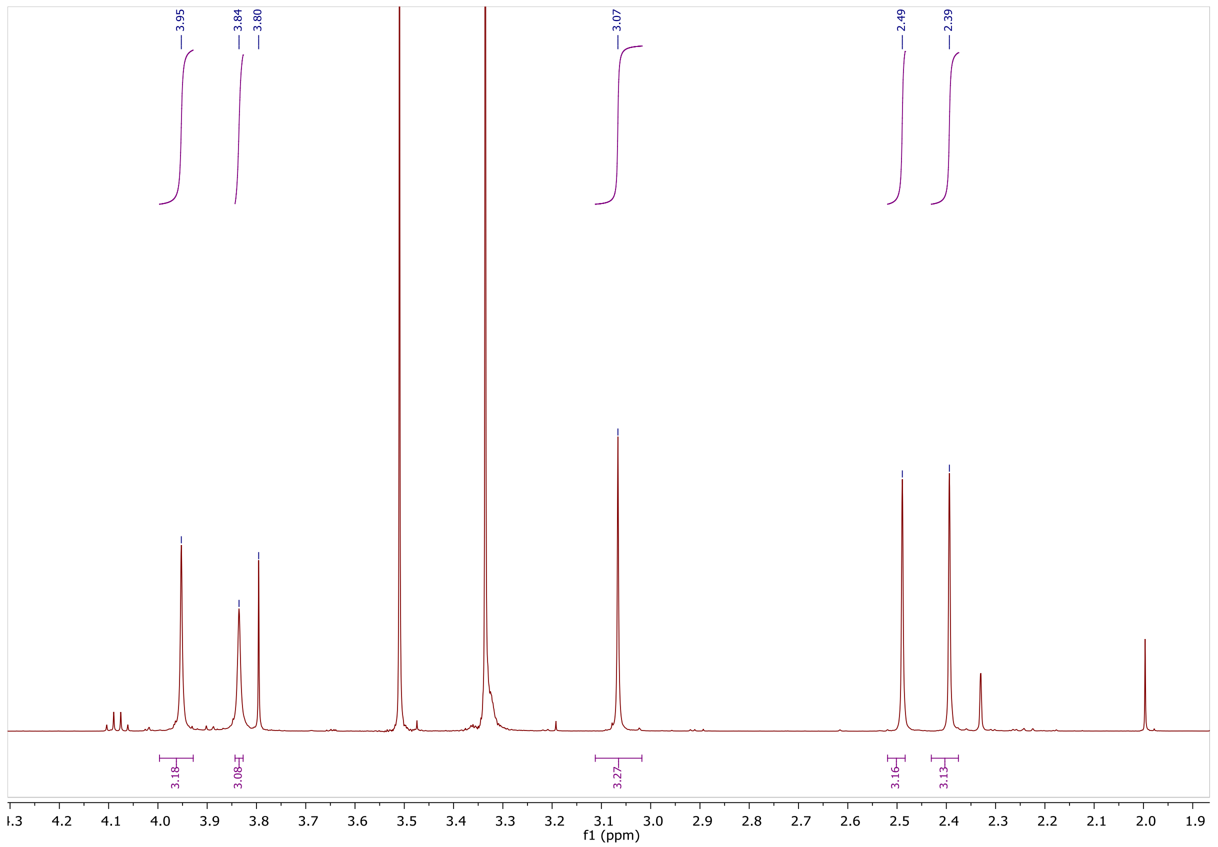
3.95
3.84
3.80
3.07
2.49
2.39
3.18
3.08
3.27
3.16
3.13
4.3 4.2 4.1 4.0 3.9 3.8 3.7 3.6 3.5 3.4 3.3 3.2 3.1 3.0 2.9 2.8 2.7 2.6 2.5 2.4 2.3 2.2 2.1 2.0 1.9
f1 (ppm)

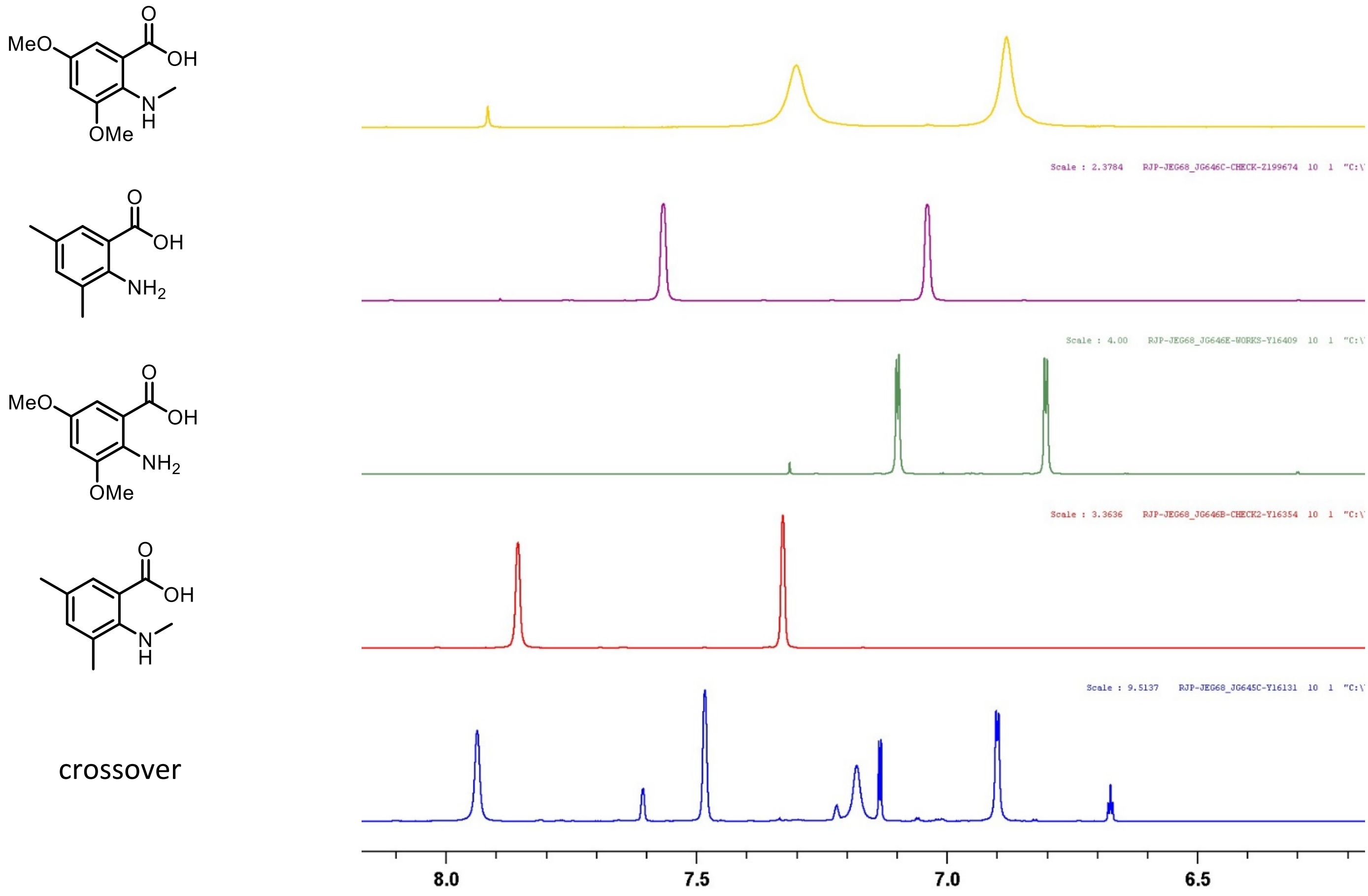
MeO
OH
OMe
NH2
crossover
8.0
7.5
7.0
6.5

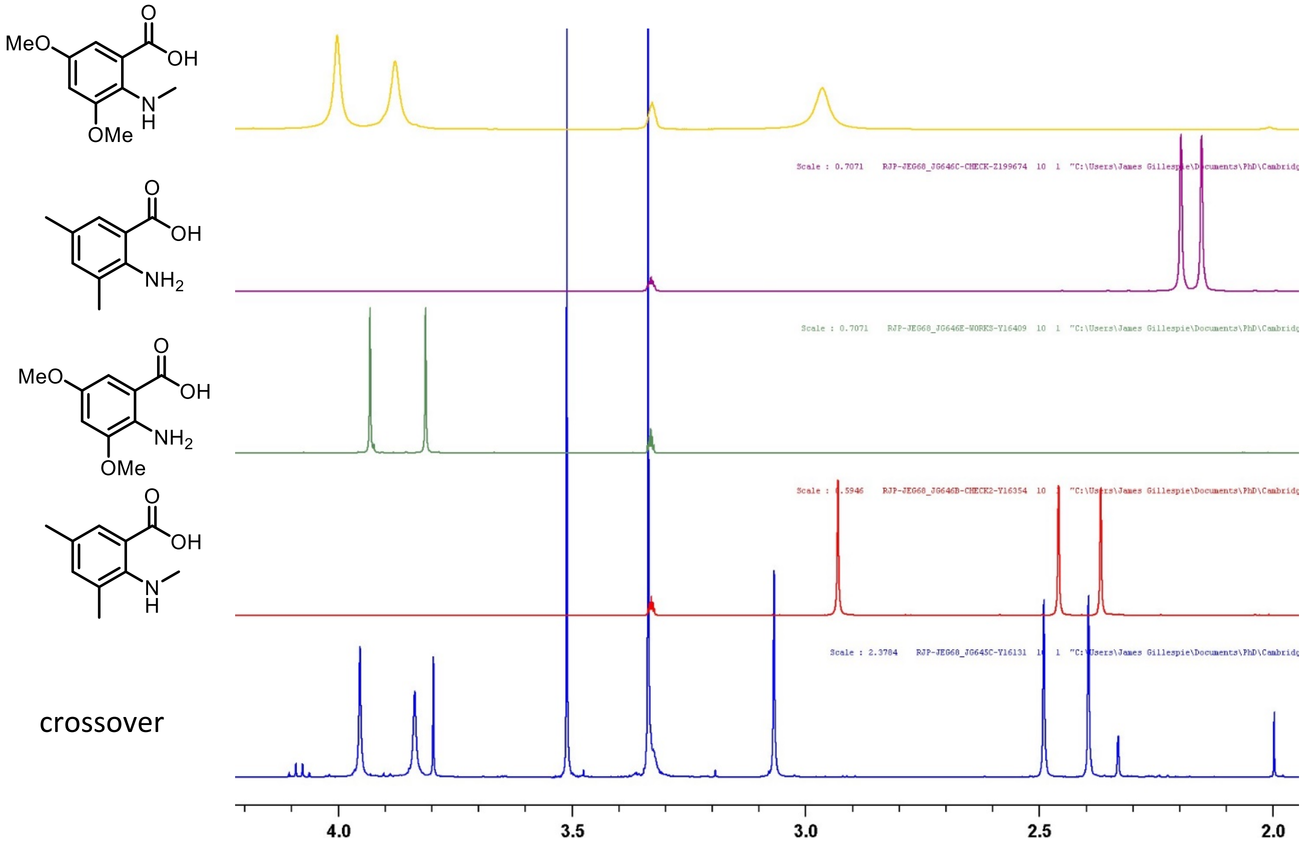

MeO
OMe
OH
NH2
crossover
4.0
3.5
3.0
2.5
2.0

Assessing relative rates of NH *vs*. NMe transfer

One scenario that could arise is that a lack of crossover could also arise from markedly different rate of NH *vs*. NMe transfer, such that the two reactions effectively take place in series rather than in parallel and giving the illusion of a lack of reaction crossover. To probe this scenario, we conducted two additional studies using the same crossover reagents (**1b** *vs*. **3i**; **1ac** vs. **3i**). By observing the extent of product formation at early time points (t = 2 and 3 h), this can be used as a readout on the relative rates of NH *vs* NMe transfer. In addition, by keeping **3i** (NMe transfer) constant we can then assess whether the rate of NH transfer as a whole might differ substantially from NMe transfer regardless of substrate substituents (*e.g.* **1b**: *p*-Me; **1ac**: *m,m'*-OMe). Standard reaction procedures were followed, and the reaction was was quenched with Et_3N (200 μL) and the mixture filtered through a pad of SiO_2 to remove iron. The pad was washed with 20% MeOH/CH_2Cl_2 and the combined filtrates were concentrated under a stream of air. ^{1}H NMR with 1,2-dimethoxyethane as an internal standard was used to determine the yield of reaction components.

*Crossover study with **1b** and **3i** – no crossover observed*

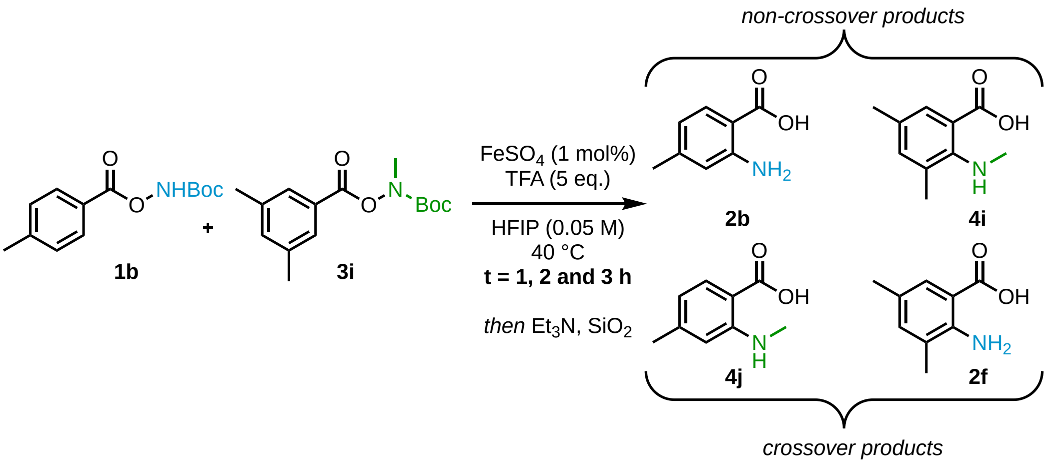

t = 1 h (yield in brackets denote a sum of deBoc starting material and benzoate recovered)

Substituents	NH aminated product (%)	NMe aminated product (%)
p-tolyl (**1b**)	17 (65)	-
3,5-di-methyl (**3i**)	-	62 (23)

t = 2 h

Substituents	NH aminated product (%)	NMe aminated product (%)
p-tolyl (**1b**)	32 (54)	-
3,5-di-methyl (**3i**)	-	88 (10)

t = 3 h

Substituents	NH aminated product (%)	NMe aminated product (%)
p-tolyl (**1b**)	56 (38)	-
3,5-di-methyl (**3i**)	-	90 (3)

*Crossover study with **1ac** and **3i** – no crossover observed*

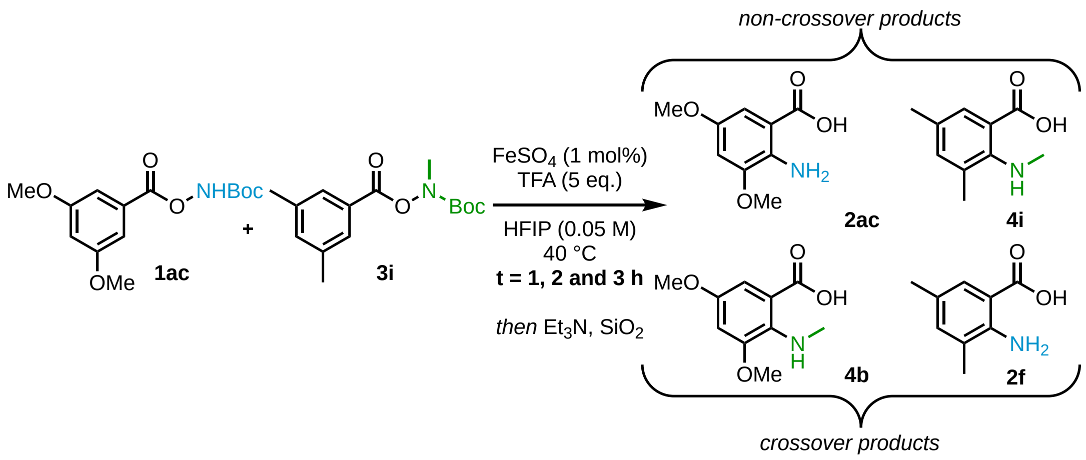

t = 1 h (yield in brackets denote a sum of deBoc starting material and benzoate recovered)

Substituents	NH aminated product (%)	NMe aminated product (%)
3,5-di-methoxy (**1ac**)	47 (31)	-
3,5-di-methyl (**3i**)	-	66 (14)

t = 2 h

Substituents	NH aminated product (%)	NMe aminated product (%)
3,5-di-methoxy (**1ac**)	66 (12)	-
3,5-di-methyl (**3i**)	-	82 (4)

t = 3 h

Substituents	NH aminated product (%)	NMe aminated product (%)
3,5-di-methoxy (**1ac**)	81 (12)	-
3,5-di-methyl (**3i**)	-	88 (0)

No crossover was observed in both cases. In addition, we find that there is an appreciable amount of product formation for both substrate classes. While the rate of NH amination (**1b**) appeared to be slower than NMe amination (**3i**), using substrate **1ac** afforded similar rates of product formation to **3i**. This provides evidence against one substrate class (*e.g.* NH vs. NMe) undergoes a markedly slower rearrangement, and therefore there is no evidence to confidently suggest that a lack of reaction crossover was due to this effect. Moreover, these experiments are still suggestive that an intramolecular amination mechanism is operative.

Feasibility of carboxylate exchange as a cross-over mechanism

A potential scenario to account for the crossover observed in **Scheme 3D** is an X,X-type carboxylate ligand exchange on the time scale of the amination step. To probe the feasibility of this step, we conducted two further competition-crossover experiments by subjecting *p*-toluic acid with aminating agents **A** (R = 2,6-difluorophenyl) and **B** (R = cyclohexyl), where there is no possibility for the aminating agent to 'self' aminate.

This experiment was chosen for three reasons. First, we know that the *p*-tolyl substrate is capable of amination to generate **2b**. Second, a lack of crossover observed in **Scheme 3C** indicates that an intermolecular amination is disfavoured compared to intramolecular amination under standard conditions. Third, by eliminating the entropically more favoured intramolecular amination pathway, any productive amination using **A** or **B** (to generate **2b**) must take place through an intermolecular mechanism, which might involve an X,X-carboxylate ligand exchange mechanism If X,X-ligand exchange was generally feasible, we would expect this to analogously take place with electronically-unbiased substrates (*e.g.* *p*-toluic acid) with structurally-similar aminating agents that are incapable of self-amination. Therefore, any crossover observed in this experiment would provide some evidence towards X,X-carboxylate ligand exchange taking place as a feasible mechanism.

Aminating agent on 2,6-difluorobenzoic acid; competition with p-toluic acid – no amination

This was chosen to minimise *ortho*-steric factors that may impact on potential ligand exchange kinetics

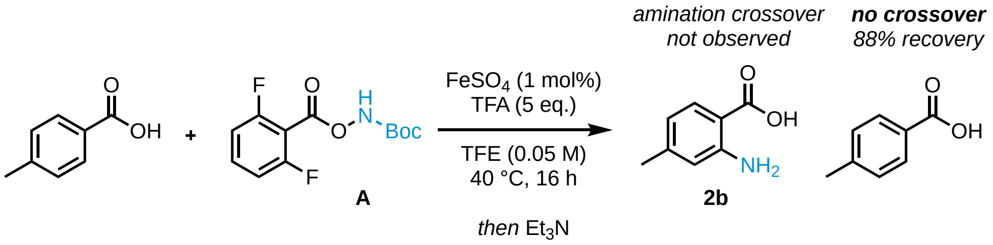

Aminating agent on cyclohexylbenzoic acid; competition with p-toluic acid – no amination

This was chosen as a saturated analogue of benzoic acid aminating agent

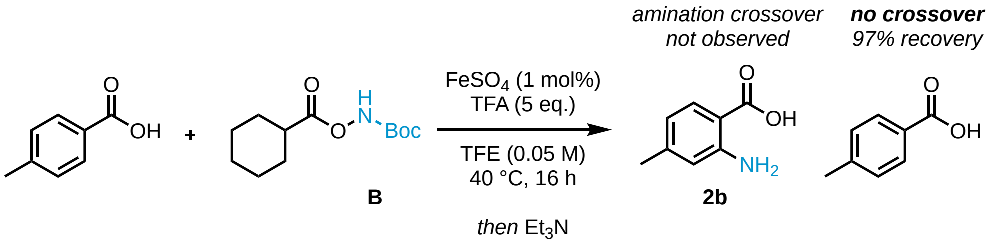

Subjecting **A** and **B** under standard aminating conditions with *p*-toluic acid gave did not give any aminated crossover product **2b**, with excellent recovery of *p*-toluic acid (along with the hydrolysed aminating agent; **A**: 72%, **B**: 91%). This provides evidence against ligand exchange as a feasible mechanism for the observed crossover in **Scheme 3D.**

Finally, we note that explicitly examining/analysing this hypothesis is not straightforward given that i) this process would occur after the rate determining step of the pathway and ii) *in situ* spectroscopic analysis is complicated by the difficulty of NMR analysis in the presence of paramagnetic Fe species. A positive outcome from this study would not conclusively implicate ligand exchange (*e.g.* potential intermolecular amination process could also give rise to the aminated product). However, a negative outcome would provide strong evidence against both process from occurring.

Characterisation data for novel aminating agents

***tert*-butyl ((2,6-difluorobenzoyl)oxy)carbamate (A)**

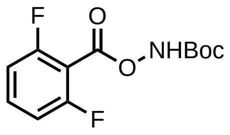

2,6-difluorobenzoic acid (320 mg, 2.02 mmol, 1.01 eq.), *tert*-butyl *N*-hydroxycarbamate (266 mg, 2.00 mmol, 1 eq.) and DCC (413 mg, 2.00 mmol, 1 eq.) were subjected to General Procedure A. The crude residue was purified by silica gel chromatography (eluent: Pet Ether:EtOAc 95:5 – 90:10) to give the title compound (450 mg, 1.65 mmol, 83%) as a white solid.

^{1}H NMR (400 MHz, $CDCl_3$) δ 8.04 (s, 1H), 7.51 (tt, *J* = 8.5, 6.1 Hz, 1H), 7.05 – 6.97 (m, 2H), 1.52 (s, 9H); ^{19}F NMR (376 MHz, $CDCl_3$) δ –107.7; ^{13}C NMR (101 MHz, $CDCl_3$) δ 162.3 (d, *J* = 5.6 Hz), 160.4, (d, *J* = 131.1 Hz), 155.1, 134.4 (t, *J* = 9.8 Hz), 112.3 (d, *J* = 22.0 Hz), 83.8, 28.0; HRMS (ESI) calcd for $[C_7H_5NO_2F_2+H]^+$ [M–Boc+H]: 174.0367. Found: 174.0370.

^{1}H NMR spectrum for aminating agent A

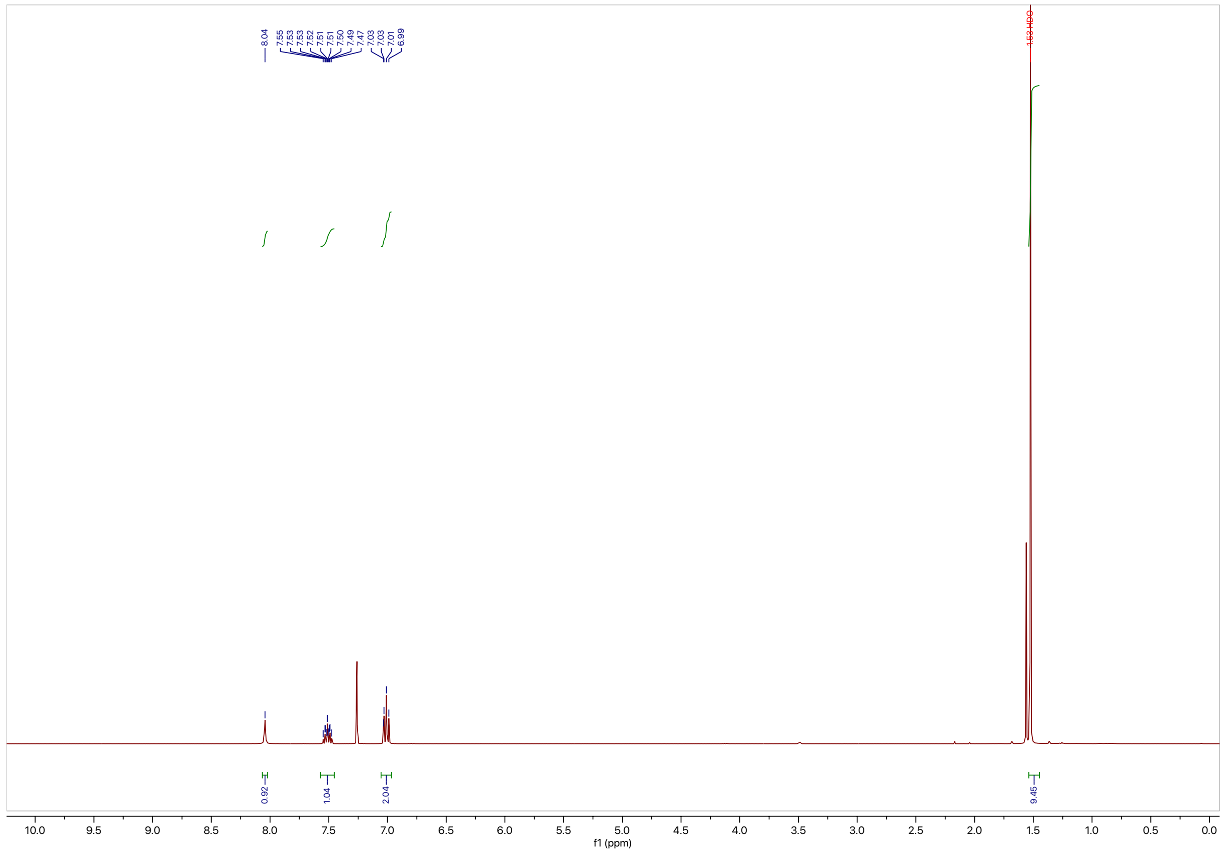

^{13}C NMR spectrum for aminating agent A

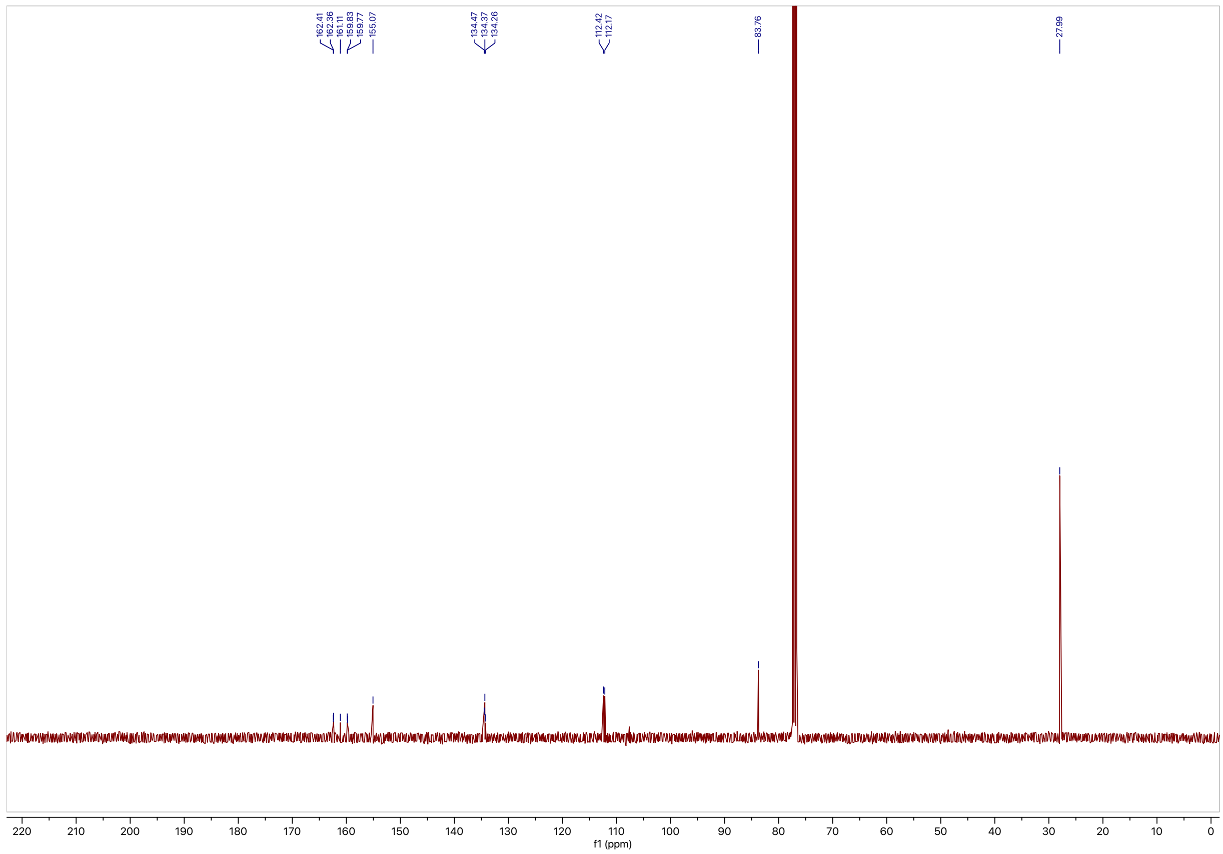

^{19}F NMR spectrum for aminating agent A

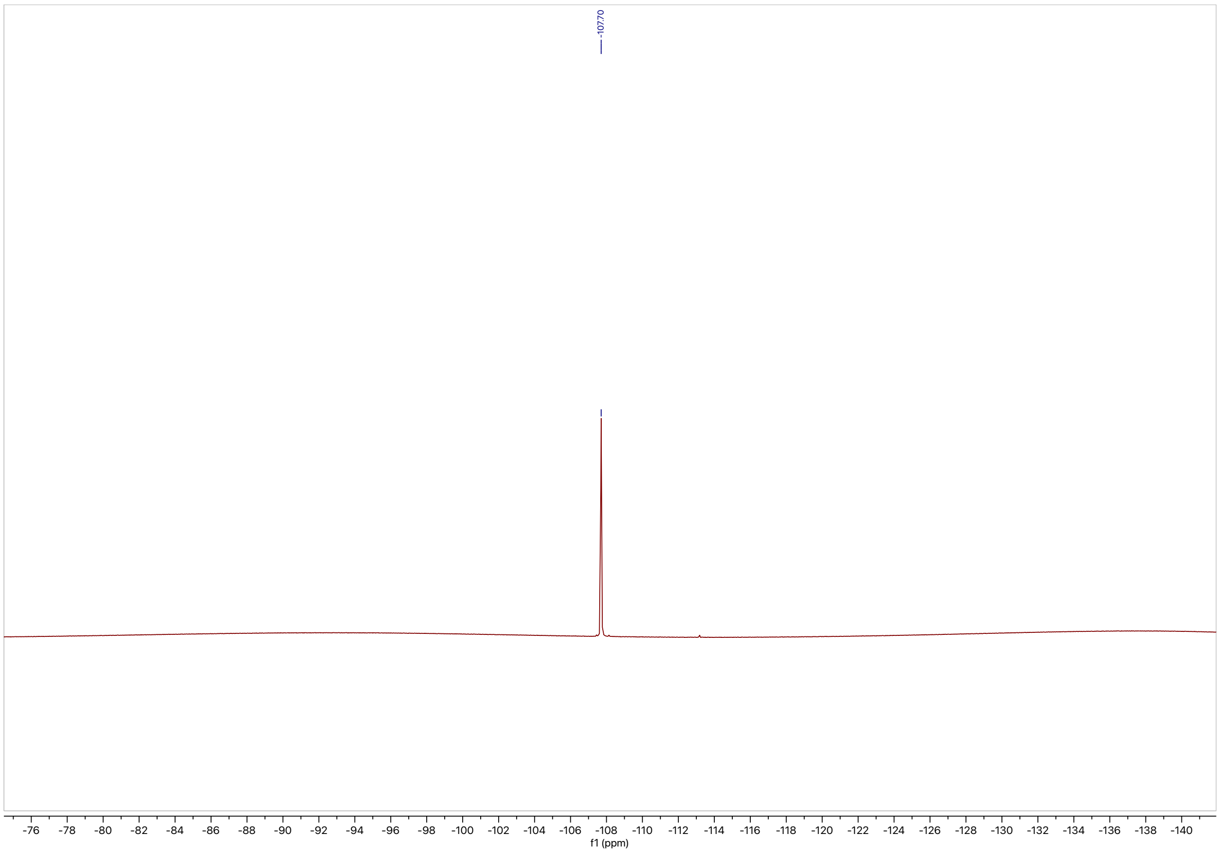

***tert*-butyl ((cyclohexanecarbonyl)oxy)carbamate (B)**

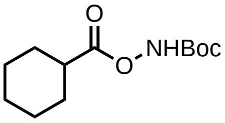

Cyclohexanecarboxylic acid (259 mg, 2.02 mmol, 1.01 eq.), *tert*-butyl *N*-hydroxycarbamate (266 mg, 2.00 mmol, 1 eq.) and DCC (413 mg, 2.00 mmol, 1 eq.) were subjected to General Procedure A. The crude residue was purified by silica gel chromatography (eluent: Pet Ether:EtOAc 95:5) to give the title compound (190 mg, 0.78 mmol, 39%) as a white solid.

^{1}H NMR (400 MHz, $CDCl_3$) δ 7.82 (s, 1H), 2.49 (tt, *J* = 11.3, 3.7 Hz, 1H), 2.01–1.93 (m, 2H), 1.83–1.70 (m, 2H), 1.65 (m, 1H), 1.53 (m, 1H), 1.48 (s, 9H), 1.38–1.17 (m, 3H).; ^{13}C NMR (101 MHz, $CDCl_3$) δ 175.3, 155.6, 83.1, 41.1, 28.7, 28.0, 25.5, 25.2; HRMS (ESI) calcd for $[C_7H_{14}NO_2+H]^+$ [M–Boc+H]: 144.1025. Found: 144.1019.

^{1}H NMR spectrum for aminating agent B

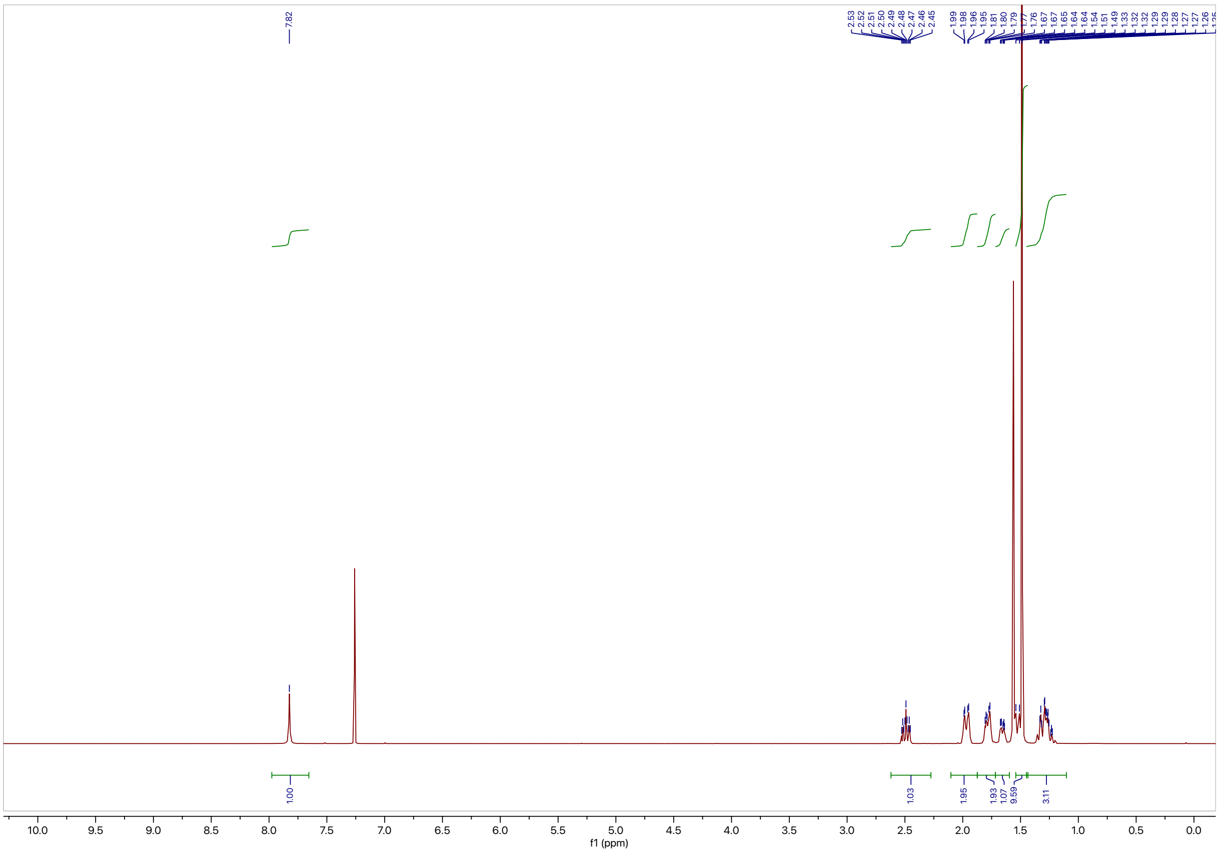

^{13}C NMR spectrum for aminating agent B

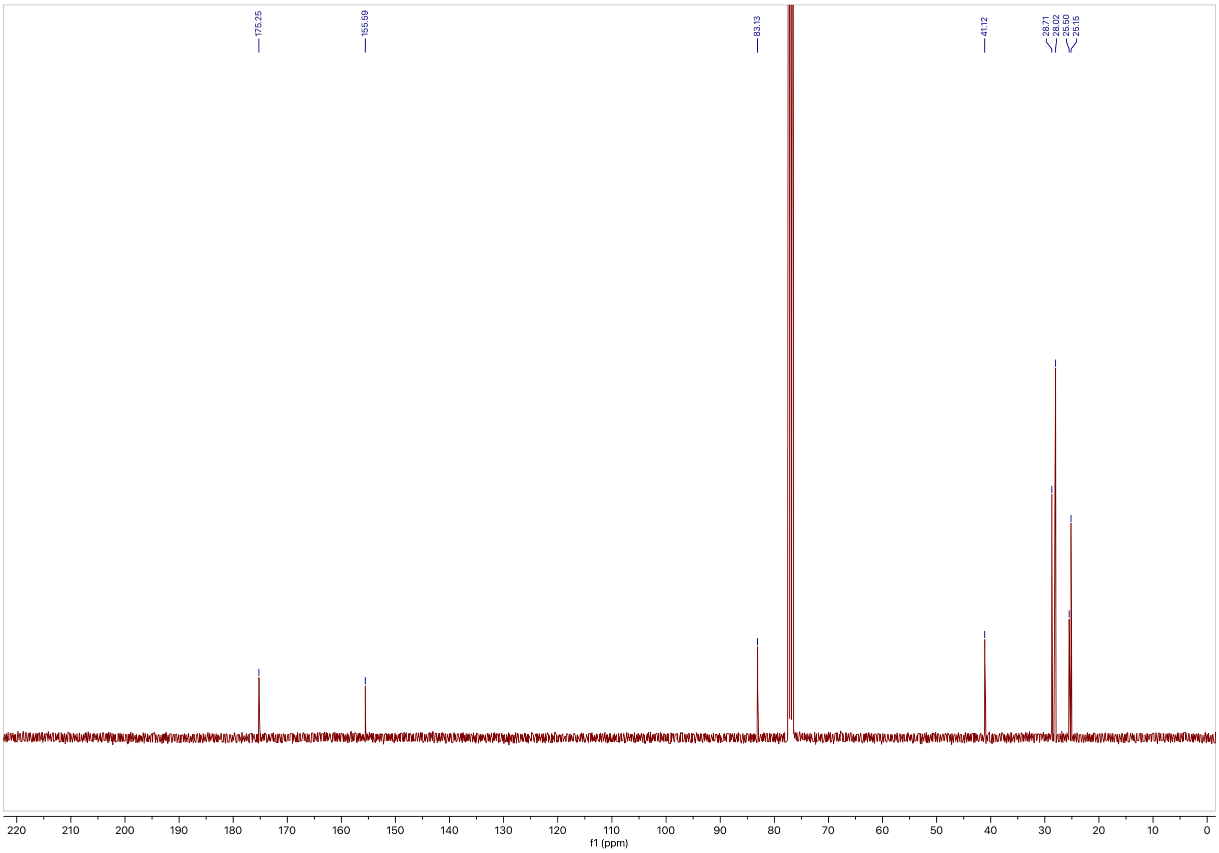

9.2 Competition Experiments

Cross-substrate class competition study

One potential explanation for the lack of crossover observed in the aminative rearrangement for carboxyl substrate classes may be because of an exceedingly fast arene amination, as compared with a slower arene aminative capture for the sulfonyl substrate class. The implication of this is such that the active aminating agent may not be able to diffuse out of the solvent cage prior to the carboxyl substrate re-capturing the aminating agent, leading to the apparent outcome of no substrate crossover. To probe this possibility, a series of cross-substrate class competition experiments were conducted to investigate the competitive rate of product formation, under iron-catalysed and iron-free conditions.

i. *Sulfonyl substrate **10** as the aminating agent*

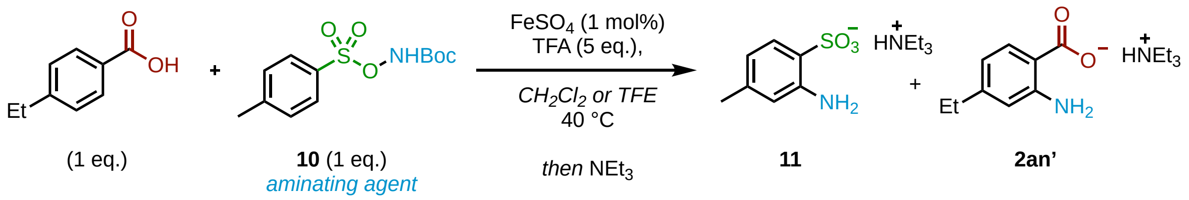

A solution of sulfonyl substrate **10** (72 mg, 0.25 mmol), *p*-ethylbenzoic acid (38 mg, 0.25 mmol) and $FeSO_4 \cdot 7H_2O$ (0.7 mg, 2.5 μmol, 1 mol%) was stirred in either CH_2Cl_2 or TFE (5 mL) at 40 °C. To this mixture was next added TFA (100 μL, 1.25 mmol, 5 eq.) to initiate the reaction, and the reaction was stirred at 40 °C for 16 h. The reaction was quenched with Et_3N (200 μL) and the mixture filtered through a pad of SiO_2 to remove iron. The pad was washed with 20% MeOH/CH_2Cl_2 and the combined filtrates were concentrated under a stream of air. ^{1}H NMR with 1,2-dimethoxyethane as an internal standard was used to determine the yield of reaction components.

No crossover was observed for this combination of substrates

	CH_2Cl_2	TFE
Sulfonyl Product **11**	63%	65%
Carboxyl Product **2an'**	0%	0%
p-toluenesulfonate	24%	20%
p-ethylbenzoate	100%	100%

ii. *Carboxyl substrate **1an** as the aminating agent*

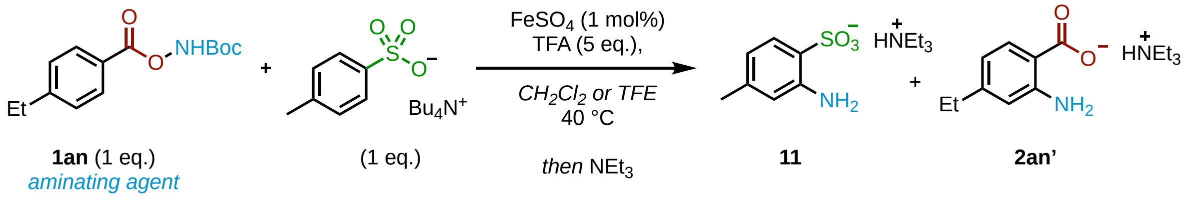

Carboxyl substrate **1an** was synthesised according to a known literature procedure (*Org. Lett.*, **2022**, *24*, 5651–5656)**.** A solution of carboxyl substrate **1an** (66 mg, 0.25 mmol), tetrabutylammonium *p*-toluenesulfonate (103 mg, 0.25 mmol) and $FeSO_4 \cdot 7H_2O$ (0.7 mg, 2.5 μmol, 1 mol%) was stirred in either CH_2Cl_2 or TFE (5 mL) at 40 °C. To this mixture was next added TFA (100 μL, 1.25 mmol, 5 eq.) to initiate the reaction, and the reaction was stirred at 40 °C for 16 h. The reaction was quenched with Et_3N (200 μL) and the mixture filtered through a pad of SiO_2 to remove iron. The pad was washed with 20% MeOH/CH_2Cl_2 and the combined filtrates were concentrated under a stream of air. ^1H NMR with 1,2-dimethoxyethane as an internal standard was used to determine the yield of reaction components. The reaction conducted without Fe catalysis afforded no product conversion (>85% recovered starting material)

Crossover was observed to the sulfonyl substrates denoted in orange

	CH_2Cl_2	TFE
Sulfonyl Product **11**	0%	11%
Carboxyl Product **2an'**	0%	4%
p-toluenesulfonate	73%	76%
p-ethylbenzoate	85%*	47% + 32%*

*Characterised as the putative acyl *O*-hydroxylamine free base. The red spectrum is from a crude aminative rearrangement reaction of **10**, highlighting the diagnostic Me signal confirming the presence of **11** in this crossover reaction (labelled orange). The green spectrum is a partially purified product (**2an**) from the above reaction, confirming its presence in the crude NMR (diagnostic benzylic methylene protons in green).

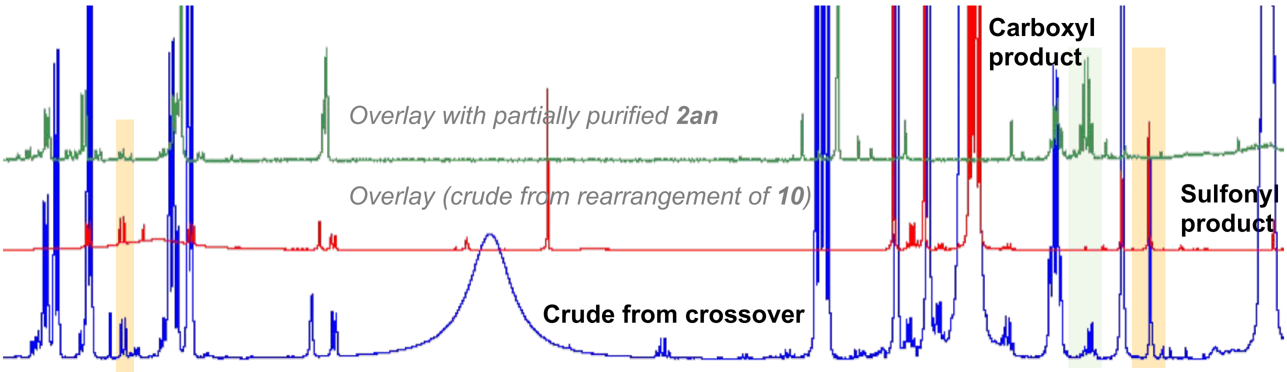

iii. O-Mesyl-N-Boc-hydroxylamine as the external aminating agent

To decouple aminating agent generation step from arene capture, a separate experiment using an external aminating agent was conducted.

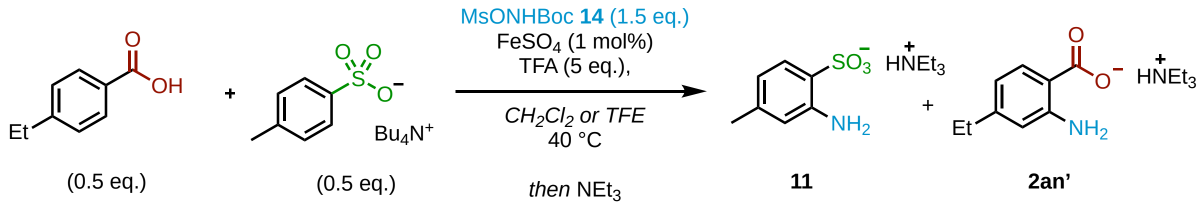

O-Mesyl-*N*-Boc-hydroxylamine **14** was synthesised according to a known literature procedure (*Angew. Chem. Int. Ed.*, **2022**, *61*, e202204025). A solution of *O*-Mesyl-*N*-Boc-hydroxylamine **14** (79 mg, 375 μmol), tetrabutylammonium *p*-toluenesulfonate (52 mg, 125 μmol, synthesis from *Angew. Chem. Int. Ed.*, **2022**, *61*, e202204025), *p*-ethylbenzoic acid (19 mg, 125 μmol) and $FeSO_4{\cdot}7H_2O$ (0.7 mg, 2.5 μmol, 1 mol%) was stirred in either CH_2Cl_2 or TFE (5 mL) at 40 °C. To this mixture was next added TFA (100 μL, 1.25 mmol, 5 eq.) to initiate the reaction, and the reaction was stirred at 40 °C for 16 h. The reaction was quenched with Et_3N (200 μL) and the mixture filtered through a pad of SiO_2 to remove iron. The pad was washed with 20% MeOH/CH_2Cl_2 and the combined filtrates were concentrated under a stream of air. ^{1}H NMR with 1,2-dimethoxyethane as an internal standard was used to determine the yield of reaction components. The reaction conducted without Fe catalyst afforded no product conversion (>85% recovered starting material)

Preferential amination occurred for the sulfonyl substrate denoted in orange

	CH_2Cl_2	TFE
Sulfonyl Product **11**	8%	22%
Carboxyl Product **2an'**	0%	0%
p-toluenesulfonate	42%	52%
p-ethylbenzoate	64%	70%

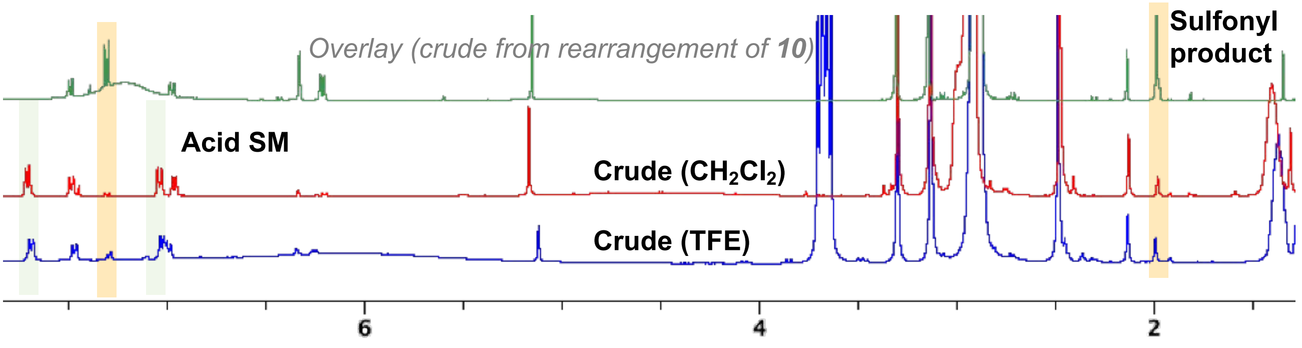

Same Substrate Class Competition Experiments

Noting that a radical amination mechanism is fundamentally bimolecular in nature, we opted to verify if crossover can be observed with our representative substrate classes under Fe-free conditions, outlined below.

i. *Iron free conditions with two different aminating agents: same electronics*

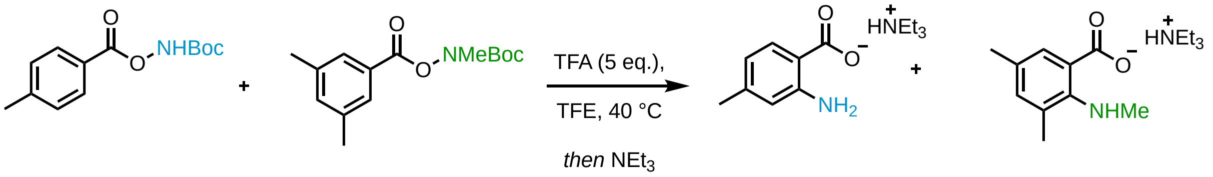

1b (0.5 eq.) **3i** (0.5 eq.) **2b'** **4i'**

A solution of *p*-Me carboxyl substrate **1b** (31 mg, 0.125 mmol), 3,5-di-Me substrate **3i** (35 mg, 0.125 mmol) was stirred in TFE (5 mL) at 40 °C. To this mixture was next added TFA (100 μL, 1.25 mmol, 5 eq.) to initiate the reaction, and the reaction was stirred at 40 °C for 16 h. The reaction was quenched with Et_3N (200 μL) and the mixture were concentrated under a stream of air. ^{1}H NMR with 1,2-dimethoxyethane as an internal standard was used to determine the yield of reaction components.

No crossover was observed for this combination of reactants

	**NH_2**	**NHMe**
p-Me Aminated Product	**2b': 20%**	-
3,5-diMe Aminated Product	-	**4i': 50%**
p-Me Benzoate	36%	-
3,5-diMe Benzoate	-	20%

ii. *Iron free conditions with two different aminating agents: different arene electronics*

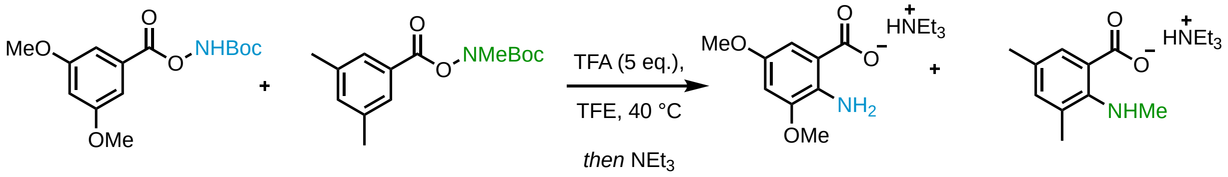

1ac (0.5 eq.) **3i** (0.5 eq.) **2ac'** **4i'**

A solution of 3,5-di-OMe substrate **1ac** (35 mg, 0.125 mmol), 3,5-di-Me substrate **3i** (37 mg, 0.125 mmol) was stirred in TFE (5 mL) at 40 °C. To this mixture was next added TFA (100 μL, 1.25 mmol, 5 eq.) to initiate the reaction, and the reaction was stirred at 40 °C for 16 h. The reaction was quenched with Et_3N (200 μL) and the

mixture were concentrated under a stream of air. ^{1}H NMR with 1,2-dimethoxyethane as an internal standard was used to determine the yield of reaction components.

No crossover was observed for this combination of reactants

	**NH_2**	**NHMe**
3,5-diOMe Aminated Product	**2ac': 28%**	-
3,5-diMe Aminated Product	-	**4i': 42%**
3,5-diOMe Benzoate	24%	-
3,5-diMe Benzoate	-	21%

iii. Iron free conditions with carboxyl aminating agent ***1b*** *doped with benzoic acid*

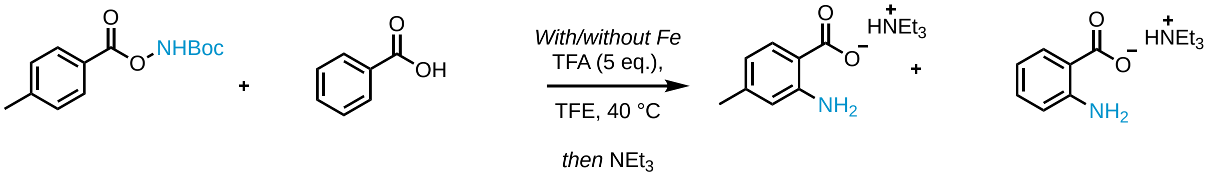

1b (0.5 eq.) (0.5 eq.) **2b'** **2a'**

A solution of *p*-Me carboxyl substrate **1b** (31 mg, 0.125 mmol) and benzoic acid (15 mg, 0.125 mmol) was stirred in TFE (5 mL) at 40 °C. For reactions conducted with Fe, $FeSO_4{\cdot}7H_2O$ (0.7 mg, 2.5 μmol, 1 mol%) was also added. To this mixture was next added TFA (100 μL, 1.25 mmol, 5 eq.) to initiate the reaction, and the reaction was stirred at 40 °C for 16 h. The reaction was quenched with Et_3N (200 μL) and the mixture were concentrated under a stream of air. For reactions with iron, the crude reaction mixture was filtered through a pad of SiO_2 to remove iron. The pad was washed with 20% MeOH/CH_2Cl_2 and the combined filtrates were concentrated under a stream of air. ^{1}H NMR with 1,2-dimethoxyethane as an internal standard was used to determine the yield of reaction components.

Reaction without Fe—no crossover was observed for this combination of reactants

	***p*-Me (2b')**	***p*-H (2a')**
Aminated Product	**67%**	-
Benzoate	26%	>99%

Reaction with Fe—no crossover was observed for this combination of reactants

	***p*-Me (2b')**	***p*-H (2a')**
Aminated Product	**62%**	-
Benzoate	26%	>99%

iv. *Iron and free conditions with carboxyl aminating agent **1b** doped with 3,5-dimethoxybenzoic acid*

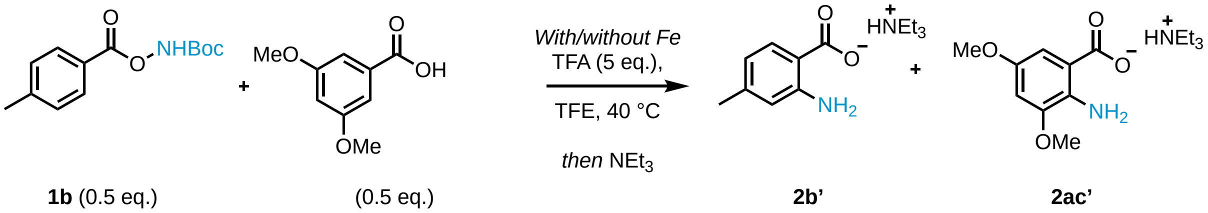

A solution of *p*-Me carboxyl substrate **1b** (31 mg, 0.125 mmol) and 3,5-dimethoxybenzoic acid (15 mg, 0.125 mmol) was stirred in TFE (5 mL) at 40 °C. For reactions conducted with Fe, $FeSO_4 \cdot 7H_2O$ (0.7 mg, 2.5 µmol, 1 mol%) was also added. To this mixture was next added TFA (100 µL, 1.25 mmol, 5 eq.) to initiate the reaction, and the reaction was stirred at 40 °C for 16 h. The reaction was quenched with Et_3N (200 µL) and the mixture were concentrated under a stream of air. For reactions with iron, the crude reaction mixture was filtered through a pad of SiO_2 to remove iron. The pad was washed with 20% MeOH/CH_2Cl_2 and the combined filtrates were concentrated under a stream of air. ^{1}H NMR with 1,2-dimethoxyethane as an internal standard was used to determine the yield of reaction components. The diagnostic OMe signals in **2ac'** were used to determine the NMR yield of the crossover product.

Reaction without Fe – crossover observed denoted in orange

	***p*-Me (2b')**	**3,5-di-OMe (2ac')**
Aminated Product	**51%**	**20%**
Benzoate	49%	80%

Reaction with Fe – crossover observed denoted in orange

	***p*-Me (2b')**	**3,5-di-OMe (2ac')**
Aminated Product	**37%**	**9%**
Benzoate	38%	61%

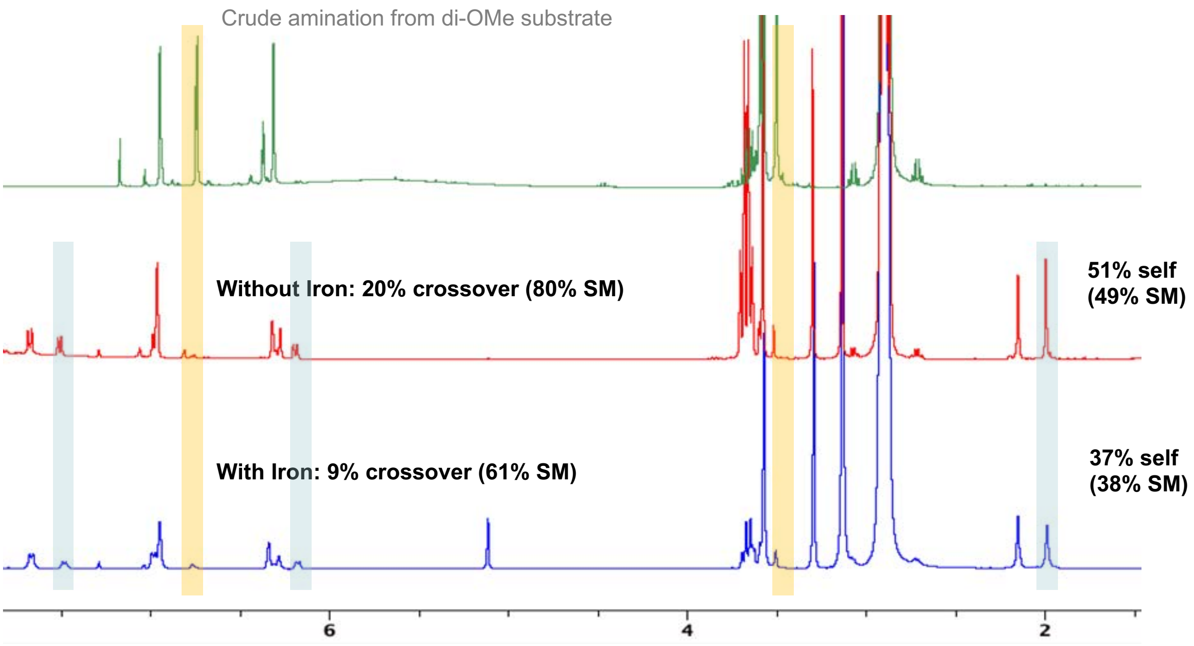

Collectively, all three cross-substrate class competition experiments indicate that the sulfonyl substrate is the preferred arene that undergoes amination. These experiments give an indication that the product determining step (arene amination) is kinetically faster for the sulfonyl substrate over the carboxyl substrate, refuting the hypothesis that a lack of reaction cross over may indicate a rapid intermolecular aminative capture from the carboxyl substrate. These experiments provide additional validation of the intermolecular mode of amination (particularly when the aminating agent is located on the carboxyl group) that was suggestive in the cross over experiments conducted in *Angew. Chem. Int. Ed.*, **2022**, *61, e202204025*.

These results indicate that an intermolecular amination mechanism is feasible if the arene electronics differ sufficiently. It also implies that under standard conditions, intermolecular amination is outcompeted by an entropically more accessible intramolecular/self-amination reaction for carboxyl substrate (in the case of iron catalysis), as well as implying that even though a bimolecular mechanism is implicated for radical amination, self-amination is kinetically more favourable than the corresponding intermolecular cross-amination. Both of these results further imply that self-amination is entropically more favoured than the corresponding intermolecular cross amination pathway, which reconciles results between the lack of cross-over observed (*self-amination is more favourable when given the opportunity)*, chain length study/observation of amination regioisomer (*substrate-guided intermolecular amination is possible if electronics differ sufficiently or when chain length becomes long enough that intermolecular amination becomes competitive)*.

9.3 Chain Length Study

***tert*-Butyl ((4-methoxybenzoyl)oxy)carbamate 1ai**

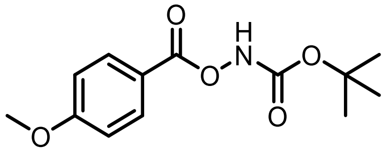

Synthesised according to our previous publication.[2]

***tert*-Butyl (2-(4-methoxyphenyl)acetoxy)carbamate 1aj**

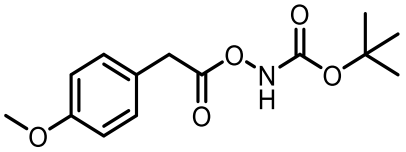

4-Methoxyphenylacetic acid (833 mg, 5.00 mmol, 1 eq.), *tert*-butyl *N*-hydroxycarbamate (665 mg, 5.00 mmol, 1 eq.) and DCC (1.09 g, 5.28 mmol, 1.05 eq.) were subject to general procedure C. The crude residue was purified by silica gel chromatography (eluent: Pet Ether:EtOAc 80:20) to give the title compound (660 mg, 2.35 mmol, 47%) as a colourless oil.

^{1}H NMR (400 MHz, $CDCl_3$) δ 8.08 (br s, 1H), 7.20 (d, *J* = 8.7 Hz, 2H), 6.85 (d, *J* = 8.7 Hz, 2H), 3.76 (s, 3H), 3.67 (s, 2H), 1.44 (s, 9H); ^{13}C NMR (101 MHz, $CDCl_3$) δ 171.1, 159.0, 155.6, 130.4, 124.4, 114.2, 83.1, 55.2, 37.7, 28.0; HRMS (ESI) calcd for $[C_{14}H_{19}NO_5 + Na]^+$: 304.1155. Found: 304.1153.

***tert*-Butyl ((3-(4-methoxyphenyl)propanoyl)oxy)carbamate 1ak**

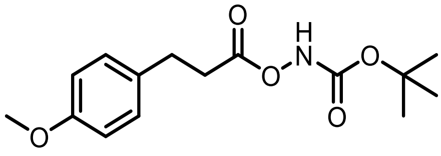

3-(4-Methoxyphenyl)propionic acid (900 mg, 5.00 mmol, 1 eq.), *tert*-butyl *N*-hydroxycarbamate (665 mg, 5.00 mmol, 1 eq.) and DCC (1.09 g, 5.28 mmol, 1.05 eq.) were subject to general procedure C. The crude residue was purified by silica gel chromatography (eluent: Pet Ether:EtOAc 95:5 - 80:20) to give the title compound (824 mg, 2.79 mmol, 56%) as a colourless oil.

^{1}H NMR (400 MHz, $CDCl_3$) δ 7.93 (br s, 1H), 7.12 (d, *J* = 8.6 Hz, 2H), 6.83 (d, *J* = 8.6 Hz, 2H), 3.78 (s, 3H), 2.95 (t, *J* = 7.8 Hz, 2H), 2.73 (t, *J* = 7.8 Hz, 2H), 1.48 (s, 9H); ^{13}C NMR (101 MHz, $CDCl_3$) δ 172.2, 158.3, 155.5, 131.7, 129.3, 114.0, 83.2, 55.3, 33.8, 29.7, 28.0; HRMS (ESI) calcd for $[C_{15}H_{21}NO_5 + Na]^+$: 318.1312. Found: 318.1306.

tert-Butyl ((4-(4-methoxyphenyl)butanoyl)oxy)carbamate 1al

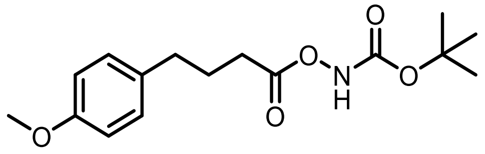

4-(4-Methoxyphenyl)butyric acid (970 mg, 5.00 mmol, 1 eq.), *tert*-butyl *N*-hydroxycarbamate (665 mg, 5.00 mmol, 1 eq.) and DCC (1.09 g, 5.28 mmol, 1.05 eq.) were subject to general procedure C. The crude residue was purified by silica gel chromatography (eluent: Pet Ether:EtOAc 95:5 - 80:20) to give the title compound (1.04 g, 3.36 mmol, 67%) as a colourless oil.

^{1}H NMR (400 MHz, $CDCl_3$) δ 8.01 (br s, 1H), 7.09 (d, *J* = 8.6 Hz, 2H), 6.82 (d, *J* = 8.6 Hz, 2H), 3.77 (s, 3H), 2.62 (t, *J* = 7.5 Hz, 2H), 2.43 (t, *J* = 7.5 Hz, 2H), 1.98 (p, *J* = 7.5 Hz, 2H), 1.48 (s, 9H); ^{13}C NMR (101 MHz, $CDCl_3$) δ 172.8, 158.0, 155.6, 132.9, 129.4, 113.9, 83.1, 55.2, 34.0, 31.0, 28.0, 26.5; HRMS (ESI) calcd for $[C_{16}H_{23}NO_5 + Na]^+$: 332.1468. Found: 332.1472

2-Amino-4-methoxybenzoic acid 2ai and 3-amino-4-methoxybenzoic acid *iso*-2ai

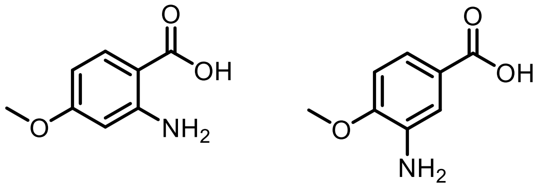

tert-Butyl ((4-methoxybenzoyl)oxy)carbamate (134 mg, 0.50 mmol, 1 eq.), TFA (285 mg, 2.50 mmol, 5 eq.) and $FeSO_4 \cdot 7H_2O$ (1.4 mg, 5.04 µmol, 0.01 eq.) were reacted according to a modified version of general procedure C. The crude residue was purified by silica gel chromatography (eluent: Pet. Ether:EtOAc:AcOH 95:4.5:0.5 – 70:29.5:0.5) to give the title compound **2ai** (9.7 mg, 0.058 mmol, 12%) and the title compound ***iso*-2ai** (7.0 mg, 0.041 mmol, 8%) as white solids, with as total yield of 20% and a regioisomer ratio of 1.4:1.

2-Amino-4-methoxybenzoic acid 2ai

^{1}H NMR (500 MHz, MeOD-*d*$_4$) δ 7.72 (d, *J* = 8.9 Hz, 1H), 6.23 (d, *J* = 2.5 Hz, 1H), 6.16 (dd, *J* = 8.9, 2.5 Hz, 1H), 3.76 (s, 3H); ^{13}C NMR (126 MHz, MeOD-*d*$_4$) δ 170.0, 164.4, 153.5, 133.0, 103.6, 103.6, 98.6, 54.1; HRMS (ESI) calcd for $[C_8H_9NO_3 + H]^+$: 168.0655. Found: 168.0650.

3-Amino-4-methoxybenzoic acid *iso*-2ai

^{1}H NMR (500 MHz, MeOD-*d*$_4$) δ 7.46 (dd, *J* = 8.3, 2.1 Hz, 1H), 7.43 (d, *J* = 2.1 Hz, 1H), 6.91 (d, *J* = 8.3 Hz, 1H), 3.93 (s, 3H); ^{13}C NMR (126 MHz, MeOD-*d*$_4$) δ 169.0, 151.5, 136.2, 122.8, 120.9, 115.8, 109.1, 54.8; HRMS (ESI) calcd for $[C_{18}H_9NO_3 + H]^+$: 168.0655. Found: 168.0654.

7-Methoxy-3,4-dihydroquinolin-2(1*H*)-one 2ak and 3-(3-amino-4-methoxyphenyl)propanoic acid S2ak

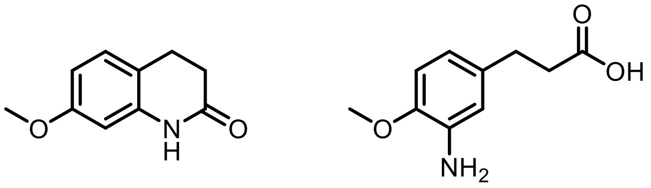

tert-Butyl ((3-(4-methoxyphenyl)propanoyl)oxy)carbamate (148 mg, 0.50 mmol, 1 eq.), TFA (285 mg, 2.50 mmol, 5 eq.) and $FeSO_4 \cdot 7H_2O$ (1.4 mg, 5.04 µmol, 0.01 eq.) were reacted according to a modified version of general procedure C. The crude residue was purified by silica gel chromatography (eluent: Pet. Ether:EtOAc:AcOH 99.5:0:0.5 – 40:59.5:0.5) to give the title compound **2ak** (4.2 mg, 0.024 mmol, 5%) and the title compound ***iso*-2ak** (27.0 mg, 0.14 mmol, 28%) as white solid and brown oil, respectively, with as total yield of 33% and a product ratio of 1:5.8.

7-Methoxy-3,4-dihydroquinolin-2(1*H*)-one 2ak

^{1}H NMR (500 MHz, MeOD-d_4) δ 7.06 (d, *J* = 8.3 Hz, 1H), 6.54 (dd, *J* = 8.3, 2.5 Hz, 1H), 6.45 (d, *J* = 2.5 Hz, 1H), 3.75 (s, 3H), 2.87 (t, *J* = 7.6 Hz, 2H), 2.56 – 2.50 (m, 2H); ^{13}C NMR (126 MHz, MeOD-d_4) δ 172.7, 159.3, 138.3, 128.2, 115.8, 107.8, 101.2, 54.3, 30.5, 24.0. HRMS (ESI) calcd for $[C_{10}H_{11}NO_2 + H]^+$: 178.0863. Found: 178.0868.

3-(3-Amino-4-methoxyphenyl)propanoic acid S2ak

^{1}H NMR (500 MHz, MeOD-d_4) δ 6.83 (d, *J* = 8.2 Hz, 1H), 6.77 (s, 1H), 6.72 (d, *J* = 7.9 Hz, 1H), 3.85 (s, 3H), 2.80 (t, *J* = 7.7 Hz, 2H), 2.56 (dd, *J* = 8.1, 7.2 Hz, 2H); ^{13}C NMR (126 MHz, MeOD-d_4) δ 175.5, 147.3, 133.5, 132.5, 120.4, 117.0, 110.5, 54.8, 35.6, 30.0; HRMS (ESI) calcd for $[C_{10}H_{13}NO_3 + H]^+$: 196.0968. Found: 196.0968.

4-(3-Amino-4-methoxyphenyl)butanoic acid 2al

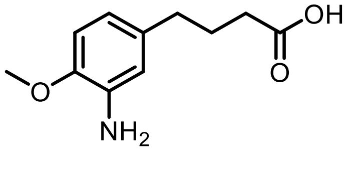

tert-Butyl ((4-(4-methoxyphenyl)butanoyl)oxy)carbamate (155 mg, 0.50 mmol, 1 eq.), TFA (285 mg, 2.50 mmol, 5 eq.) and $FeSO_4 \cdot 7H_2O$ (1.4 mg, 5.04 µmol, 0.01 eq.) were reacted according to a modified version of general procedure C. The crude was purified by silica gel chromatography (eluent: Pet. Ether:EtOAc:AcOH 95:4.5:0.5 – 60:39.5:0.5) to give the title compound (31.1 mg, 0.15 mmol, 31%) as a pale brown solid.

^{1}H NMR (500 MHz, DMSO-d_6) δ 6.90 (d, *J* = 8.2 Hz, 1H), 6.82 (d, *J* = 2.1 Hz, 1H), 6.76 (dd, *J* = 8.3, 2.1 Hz, 1H), 3.79 (s, 3H), 2.50 – 2.45 (m, 2H), 2.20 (t, *J* = 7.4 Hz, 2H), 1.74 (h, *J* = 7.5 Hz, 2H); ^{13}C NMR (126 MHz, DMSO-d_6)

δ 174.8, 148.0, 134.5, 129.3, 122.8, 119.1, 111.9, 56.1, 34.1, 33.4, 26.9; HRMS (ESI) calcd for $[C_{11}H_{15}NO_3 + H]^+$: 210.1125. Found: 210.1119.

Chain length study

Our crossover studies suggest that this occurs only for electronically-activated substrates that can out-compete against a more favorable intramolecular/self-amination process which is the most typical scenario. This outcome reconciles observations seen during our scope exploration, where we saw eroded *ortho*-selectivity in the presence of a *para*-methoxy substituent. Additionally, we observed that the *para*-methyl substituted substrate **5l** with a longer tether gave a 1:1 mixture of ortho and meta aminated products (see **Scheme 1C** in main text, **6l** and **6m** respectively). This observation suggests that proximity factors may favour *ortho*-selective 'self-amination' with short chain lengths, while at longer chain lengths intermolecular 'non-directed' reactions become competitive. To gain further support, we investigated this effect more deeply with a series of para-methoxy-substituted substrates of varying chain length (**1ai**-**1al** n = 0–3). With the strongly electron-donating methoxy substituent even the benzoic acid derivative (**1ai**, n = 0) gave a mixture of ortho and meta isomers. The ratio for n = 1 substrate 1aj could not be determined but for n = 2 (**1ak**) the *ortho*:*meta* ratio decreased to 1:5.8 and for n = 3 (**1al**) the *meta* isomer was produced almost exclusively. These results provide further evidence for a preferential proximity-induced self-amination which is only overturned when intrinsic substrate reactivity renders an intermolecular process more accessible.

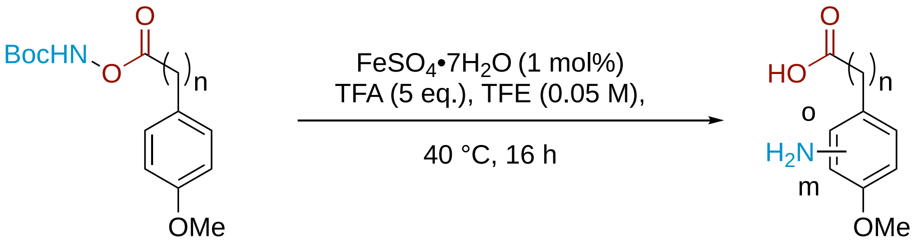

Substrate	Isolated yield (%)	r.r. (o:m)
**1ai**: n = 0	20	1.4:1
**1aj**: n = 1	n.d.	complex mixture
**1ak**: n = 2*	33*	1:5.8
**1al**: n = 3	31	<1:20

9.4 Probing the Effect of Acid

Table S9. The effect of acid on the rearrangement of benzoyl O-hydroxylamines.

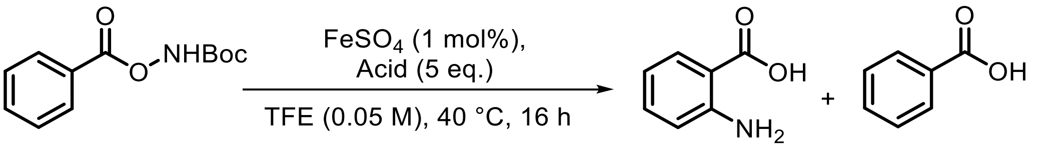

1a **2a**

Entry[a]	Acid	Yield (2a)[b]	Yield (Benzoic acid)	Yield (SM)
1	AcOH	0	12	33
2	CF_3CH_2COOH	4	14	70
3	CF_3CF_2COOH	56	19	0
4	CF_2HCOOH	34	34	17
5	CCl_3COOH	56	29	0
6	TFA	66	22	0
7	MsOH	0	107	0
8	*p*TsOH	0	60	0
9	TfOH	2	63	0

[a] Reactions were performed on a 0.1 mmol scale. [b] Yields for each product were assessed by ^{1}H NMR using 1,2-dimethoxyethane as an internal standard.

Benzoyl *O*-hydroxylammonium trifluoromethanesulfonate 9a

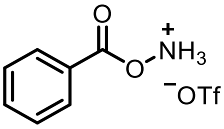

Synthesised according to our previous publication.[2]

Benzoyl *O*-hydroxylamine 9b

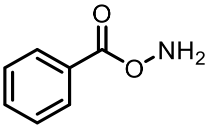

Benzoyl *O*-hydroxylammonium trifluoromethanesulfonate (**9a**, 287 mg, 1.00 mmol) was suspended in chloroform (5 mL) and saturated aqueous sodium carbonate (5 mL) was added. The aqueous layer was

extracted with chloroform (3 x 5 mL). The combined organics were dried ($MgSO_4$), filtered and concentrated under a stream of air to give the title compound which was used without further purification.

^1H NMR (400 MHz, $CDCl_3$) δ 8.09 – 7.89 (m, 2H), 7.63 – 7.52 (m, 1H), 7.45 (t, *J* = 7.7 Hz, 2H), 5.46 (s, 2H); ^{13}C NMR (101 MHz, $CDCl_3$) δ 167.6, 133.5, 129.4, 128.6, 128.0.

Data matches literature values.[9]

Experimental considerations for the rearrangement of benzoyl *O*-hydroxylamine 9b

It was found that benzoyl *O*-hydroxylamine **9b** degraded if allowed to solidify. Therefore, it was concentrated under a stream of air until such a stage as the compound resembled a colourless oil. This meant an accurate yield was impossible to quantify. **9b** was then weighed and a stock solution of **9b** in TFE was made such that 2 mL of TFE contained approximately 0.1 mmol of **9b**. TFA (5 eq.) and $FeSO_4·7H_2O$ (0.01 eq.) was then added to 2 mL of this stock solution and the reaction was conducted as described in general procedure C.

*Table S10. The rearrangement of free base **9b**.*

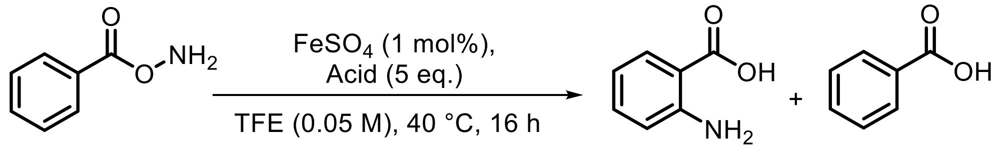

9b **2a**

Entry[a]	Acid	Yield (2a)[b]	Yield (Benzoic acid)
1	TFA	70	13
2[c]	TFA	38	32
3	AcOH	0	63
4	TfOH	0	83
5[d]	TFA	0	97
6	No acid	0	52

[a] Reactions were performed on a 0.1 mmol scale. [b] Yields for each product were assessed by ^1H NMR using 1,2-dimethoxyethane as an internal standard. [c] No $FeSO_4$ added. [d] Triflate salt **9a** used.

9.5 Stoichiometric Experiments with Protonated Triflate Salt 9a

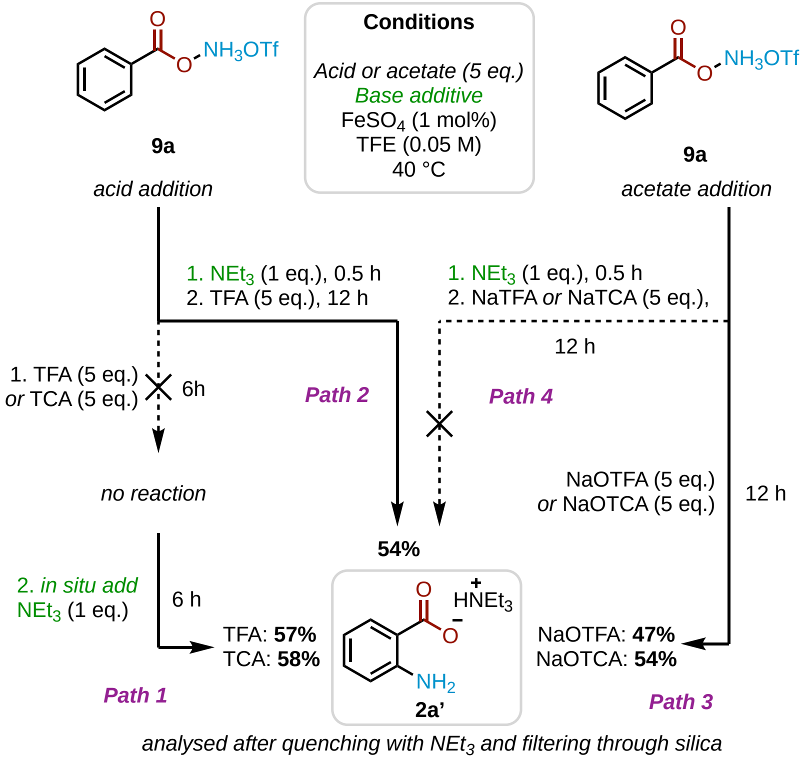

Role of acid with protonated triflate salt 9a

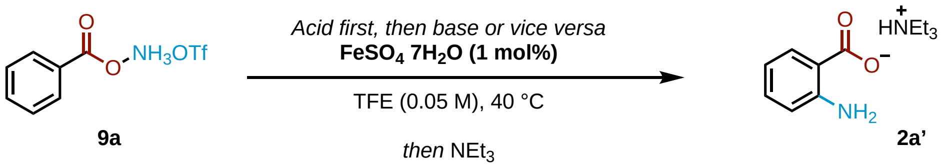

i. Acid addition only (Path 1)

A solution of protonated carboxyl substrate **9a** (62.0 mg, 0.25 mmol) and $FeSO_4·7H_2O$ (0.7 mg, 2.5 μmol) was stirred in TFE (5 mL) at 40 °C. The indicated acid (TFA or TCA, 5 eq.) was added and the reaction was stirred. After 6 h, a 2.5 mL aliquot of the reaction mixture was removed, quenched with Et_3N (100 μL) and filtered through a thin plug of SiO_2 to remove trace iron. The SiO_2 plug was next washed (20% MeOH/CH_2Cl_2) and the combined filtrates were concentrated under a stream of air.

^{1}H NMR with 1,2-dimethoxyethane as internal standard indicated no product formation with TFA or TCA.

ii. Acid addition followed by in situ base addition (Path 1)

To the remaining reaction mixture (2.5 mL) described above was added Et_3N (17 μL, 0.125 mmol, 1 eq.). The reaction was stirred for a further 6 h at 40 °C before quenching with Et_3N (100 μL). The solution was next filtered through a thin plug of SiO_2 to remove trace iron. The SiO_2 plug was next washed (20% MeOH/CH_2Cl_2) and the combined filtrates were concentrated under a stream of air.

^{1}H NMR with 1,2-dimethoxyethane as internal standard indicated that the product (**2a’**) was formed in 57% yield (with TFA), and 58% (with TCA)

iii. Base followed by acid addition (Path 2)

A solution of protonated carboxyl substrate **9a** (62.0 mg, 0.25 mmol), $FeSO_4{\cdot}7H_2O$ (0.7 mg, 2.5 μmol) and Et_3N (34 μL, 0.25 mmol, 1 eq.) was stirred in TFE (5 mL) at 40 °C. After stirring for 30 minutes, TFA (100 μL, 1.25 mmol, 5 eq.) was added and the reaction mixture was stirred for 12 h at 40 °C. The reaction mixture was quenched with Et_3N (200 μL). The solution was next filtered through a thin plug of SiO_2 to remove trace iron. The SiO_2 plug was next washed (20% $MeOH/CH_2Cl_2$) and the combined filtrates were concentrated under a stream of air.

^{1}H NMR with 1,2-dimethoxyethane as internal standard indicated that the product (**2a’**) formed in 54% yield.

Role of acetate with protonated triflate salt 9a

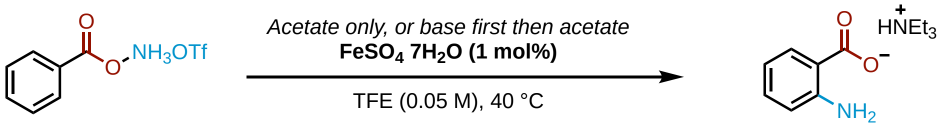

9a **2a’**

i) Acetate addition only (Path 3)

A solution of protonated carboxyl substrate **9a** (62.0 mg, 0.25 mmol), sodium trifluoroacetate or sodium trifluoroacetate (5 eq.) and $FeSO_4{\cdot}7H_2O$ (0.7 mg, 2.5 μmol) was stirred in TFE (5 mL). The reaction was stirred for 12 h at 40 °C before quenching with Et_3N (200 μL). The solution was next filtered through a thin plug of SiO_2 to remove trace iron. The SiO_2 plug was next washed (20% $MeOH/CH_2Cl_2$) and the combined filtrates were concentrated under a stream of air.

^{1}H NMR with 1,2-dimethoxyethane as internal standard indicated that the product (**2a’**) formed in 47% yield (with NaTFA) or 54% (with NaTCA).

ii) Base followed by acetate addition (Path 4)

A solution of protonated carboxyl substrate **9a** (62.0 mg, 0.25 mmol), $FeSO_4{\cdot}7H_2O$ (0.7 mg, 2.5 μmol) and Et_3N (34 μL, 0.25 mmol, 1 eq.) was stirred in TFE (5 mL) at 40 °C. After stirring for 30 minutes, NaTCA or NaTFA (5 eq.) was added and the reaction was stirred for 12 h at 40 °C. The reaction mixture was quenched with Et_3N (200 μL). The solution was next filtered through a thin plug of SiO_2 to remove trace iron. The SiO_2 plug was next washed (20% $MeOH/CH_2Cl_2$) and the combined filtrates were concentrated under a stream of air.

^{1}H NMR with 1,2-dimethoxyethane as internal standard indicated no product formation

9.6 Kinetic Isotope Effect Studies

(Phenyl-2-*d*)methanol 15

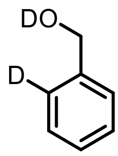

*n*BuLi (1.6 M in hexanes, 15.6 mL, 25.0 mmol, 2.5 eq.) was added dropwise to a solution of 2-bromobenzyl alcohol (1.87 g, 10.0 mmol, 1 eq.) in THF (40 mL) at -78 °C. The reaction was allowed to warm to room temperature and was stirred for 1 h. The reaction was then cooled to -78 °C and D_2O (7.2 mL, 400 mmol, 40 eq.) was added dropwise. The reaction was allowed to warm to room temperature and stirred for 2 h. The reaction was quenched with water and the aqueous layer was extracted with EtOAc (3 x 30 mL). The combined organics were then dried ($MgSO_4$), filtered and concentrated *in vacuo*. The resulting residue was purified by silica gel chromatography (eluent: Pet Ether:Et_2O 90:10 – 60:40) to give the title compound (868 mg, 8.04 mmol, 80%) as a colourless liquid with >95% deuterium incorporation.

^{1}H NMR (400 MHz, $CDCl_3$) δ 7.46 – 7.27 (m, 4H), 4.67 (s, 2H); ^{13}C NMR (101 MHz, $CDCl_3$) δ 140.8, 128.6, 128.5, 127.6, 127.0, 126.9 – 126.4 (m), 65.3.

Data matches literature values.[10]

2-*d*-Benzaldehyde 16

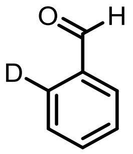

Pyridinium chlorochromate (1.81 g, 8.40 mmol, 1.2 eq.) was added to a stirred suspension of (phenyl-2-*d*)methanol-*d* (756 mg, 7.00 mmol, 1 eq.) and Celite (approx. 5 g) in dry CH_2Cl_2 (70 mL). The reaction was stirred at room temperature for 2 h. The suspension was then filtered and the resulting residue washed with CH_2Cl_2 (100 mL). The filtrate was concentrated *in vacuo*. The resulting residue was purified by silica gel chromatography (eluent: Pet Ether:Et_2O 100:0 – 95:5) to give the title compound (363 mg, 3.43 mmol, 49%) as a yellow liquid.

^{1}H NMR (400 MHz, $CDCl_3$) δ 10.03 (s, 1H), 7.89 (dd, *J* = 8.0, 1.3 Hz, 1H), 7.64 (td, *J* = 7.4, 1.3 Hz, 1H), 7.59 – 7.45 (m, 2H); ^{13}C NMR (126 MHz, $CDCl_3$) δ 192.4, 136.3, 134.5, 129.7, 129.7 – 129.1 (m), 129.0, 128.9.

Data matches literature values.[11]

tert-Butyl ((benzoyl-2-d)oxy)carbamate *d₁*-1a

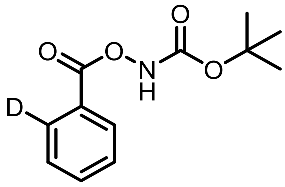

A solution of sodium chlorite (1.08 g, 12.0 mmol, 4.3 eq.) in water (15 mL) was added dropwise to a solution of benzaldehyde-2-*d* (295 mg, 2.78 mmol, 1 eq.), monosodium phosphate (234 mg, 1.50 mmol, 0.5 eq.) and hydrogen peroxide (30% aqueous solution, 1.22 mL, 12.0 mmol, 4.3 eq.) in CH_3CN:H_2O (5:1, 30 mL) at 0 °C. The reaction was stirred at room temperature for 24 h. The reaction was quenched with aqueous $Na_2S_2O_3$ and diluted with H_2O (30 mL). Aqueous HCl (2.5 M, 30 mL) was then added and the aqueous layer was extracted with EtOAc (3 x 50 mL). The combined organics were then dried ($MgSO_4$), filtered and concentrated in *vacuo*. The resulting residue, *tert*-butyl *N*-hydroxycarbamate (372 mg, 2.80 mmol, 1 eq.) and DCC (597 mg, 2.89 mmol, 1 eq.) were subject to general procedure A. The crude residue was purified by silica gel chromatography (eluent: Pet Ether:EtOAc 93:7 – 90:10) to give the title compound (459 mg, 1.93 mmol, 69% over two steps) as a white solid.

^{1}H NMR (500 MHz, $CDCl_3$) δ 8.39 (br s, 1H), 8.08 (dd, *J* = 8.2, 1.3 Hz, 1H), 7.61 (td, *J* = 7.5, 1.3 Hz, 1H), 7.51 – 7.40 (m, 2H), 1.51 (s, 9H); ^{13}C NMR (126 MHz, $CDCl_3$) δ 166.1, 155.7, 134.1, 129.9, 129.8 – 129.2 (m), 128.6, 128.5, 126.9, 83.3, 28.0; HRMS (ESI) calcd for $[C_{12}H_{14}DNO_4 + Na]^+$: 261.0956. Found: 261.0954.

Kinetic Isotope Effect Study

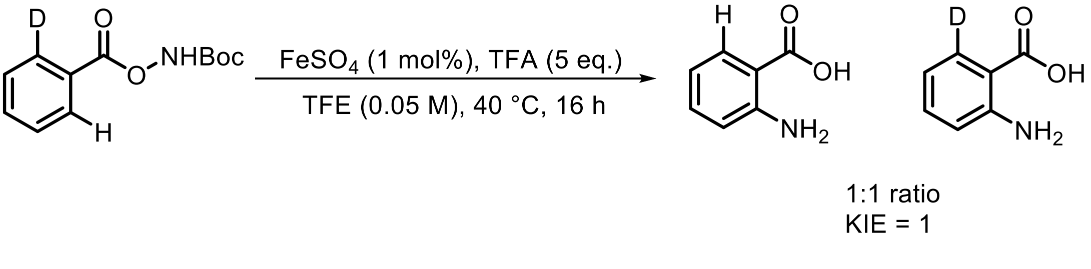

tert-Butyl ((benzoyl-2-d)oxy)carbamate (***d₁*-1a**, 47.6 mg, 0.20 mmol, 1 eq.), TFA (113 mg, 0.99 mmol, 5 eq.) and $FeSO_4 \cdot 7H_2O$ (0.6 mg, 2.16 µmol, 0.01 eq.) were subject to a modified version of general procedure C. The crude residue was purified by silica gel column chromatography (eluent: Pet.Ether:EtOAc:AcOH 95:4.5:0.5 – 75:24.5:0.5) to give the title compounds in a 1:1 ratio.

The ^{1}H NMR shows the signal at 7.82 ppm integrating to one, corresponding to the proton *ortho* to the carboxylic acid substituent in the product. This indicates a 1:1 ratio of the title compounds.

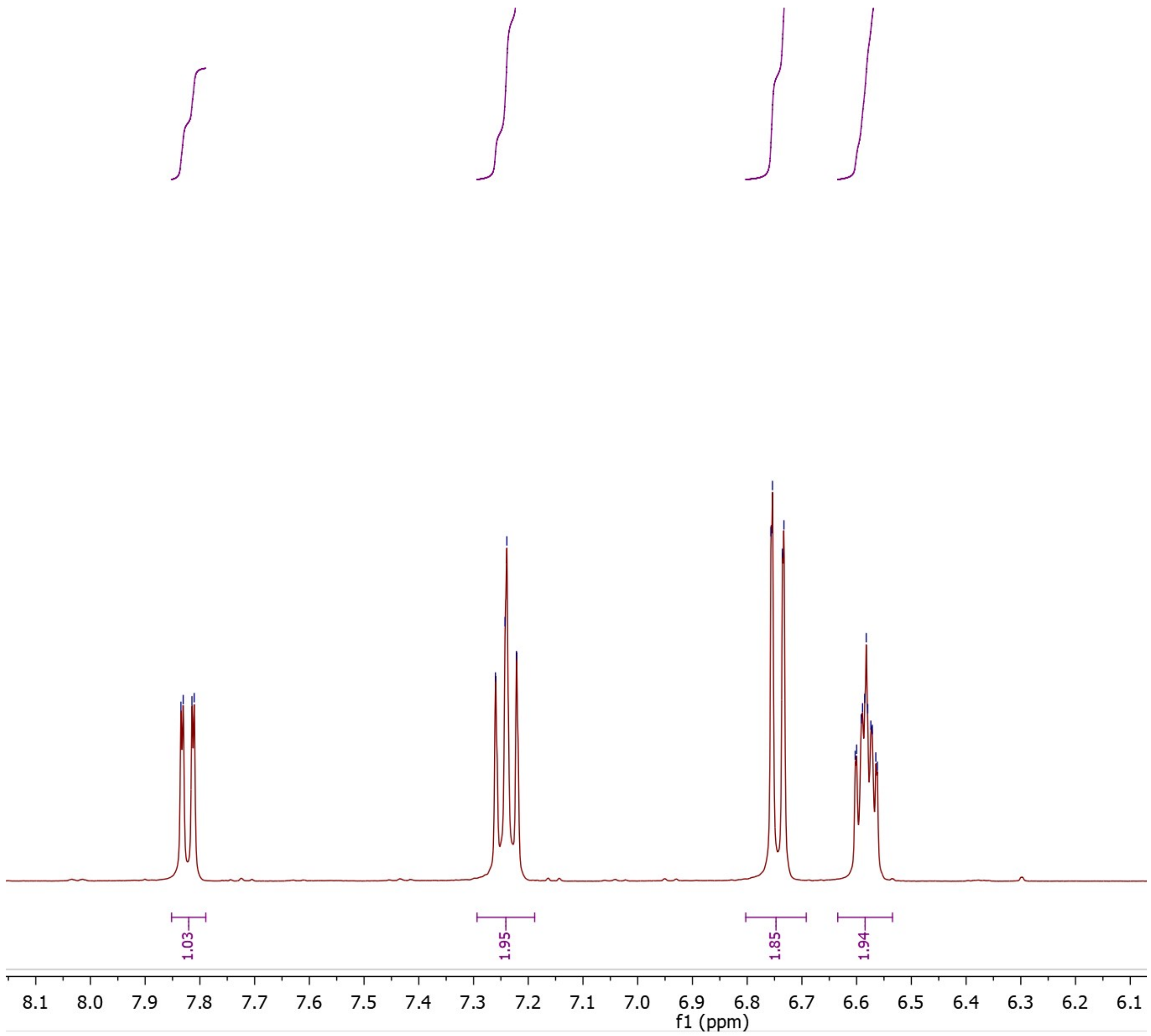

9.7 Reaction Order Determination

Given that the same specific acid dependence (TFA) was also observed for the aminative rearrangement of *O*-sulfonylhydroxylamines, we opted to evaluate them alongside our O-acylhydroxylamines in order to ascertain similarities and differences. Comparative experiments were conducted to identify reaction order in TFA to ascertain its involvement in the rate-determining step of the reaction, and ancillary studies were conducted to determine which step in the pathway was rate determining.

General Procedure

Reaction order experiments were conducted by using the method of initial rates and its change compared to the relative variance of a single reaction component. Points that deviated away from linearity were discarded from analysis and rates were approximated by using a linear regression. Individual reaction vessels were set up and subjecting to standard amination conditions (General Procedure C). The reaction was quenched with Et_3N at each designated time point. The reaction mixture was next filtered through a in plug of SiO_2 to remove trace iron. The SiO_2 plug was next washed (20% MeOH/CH_2Cl_2) and the combined filtrates were concentrated under a stream of air. ^{1}H NMR with 1,2-dimethoxyethane as an internal standard was then used to determine the % yield of each component. Where possible, several signals (for starting material related signals: benzylic Me, aromatic signals, for product-related signals: benzylic Me, aromatic signals) were cross validated on ^{1}H NMR to determine an accurate yield

To mitigate variability introduced by experimental errors, yields within the same experiment class were normalised against each other and plotted against time. A linear regression was fitted on Microsoft Excel® and initial rates was determined as the gradient from the line of best fit. We note that the rates determined from this method cannot be used as a comparison of absolute rates across substrate classes. Normalisation of initial rates and relative stoichiometry was conducted using a ln(x) transformation, and the resulting ln(rate):ln(relative stoichiometry) was plotted. A linear plot was then fitted and the reaction order is determined by the gradient of the resulting linear regression.

Finally, we also note a precise reaction order should not be concluded from this data (*i.e.* this analysis is not accurate enough to give a precise reaction order that feeds into a precise rate law) owing to the substantial experimental variability from weighing, reaction set up, isolation, NMR analysis and linear regression fitting, and so care should be exercised in interpreting exact reaction order/precise molecularity from this data. The conclusions obtained in the paper centre on whether a reaction is ordered in a particular component and its implication towards its role in the reaction pathway. In cases where the reaction is ordered, such as with TFA, it is very conceivable that one or more molecule (of TFA) may be explicitly participating at the RDTS. For completeness, linear regression analysis for normalised rate and relative concentration values are shown to show the approximate order of a particular component in the reaction.

Reaction order in TFA

For substrates **10** and **1b**, reaction order was conducted by varying TFA equivalents (5, 7.5, 10; relative equivalents: 1x, 1.5x, 2x) in the same total volume of solvent (5 mL). For *p*-CF_3 sulfonyl substrate, reaction order was conducted with 5, 10 and 15 eq. of TFA (relative equivalents: 1x, 2x, 3x).

*p-Me Sulfonyl substrate **10***

Time (h)	TFA eq. (relative eq.)	*p*-toluenesulfonate %*	Product %
0.25	5 (1)	99	1
0.50	5 (1)	86	14
1.0	5 (1)	73	27
1.5	5 (1)	32	68
0.25	7.5 (1.5)	83	15
0.35	7.5 (1.5)	74	23
0.50	7.5 (1.5)	56	39
0.75	7.5 (1.5)	33	52
0.25	10 (2)	81	19
0.35	10 (2)	56	44
0.50	10 (2)	38	62
0.6	10 (2)	20	80

Relative TFA eq.	Ln(relative TFA)	Initial Rate (%/time)	Ln(initial rate)
1.0	0.000	0.507	–0.679
1.5	0.405	1.002	–0.002
2.0	0.693	1.617	+0.481

*p-Me Sulfonyl substrate **10**: plot of product formation over time at varying TFA loading*

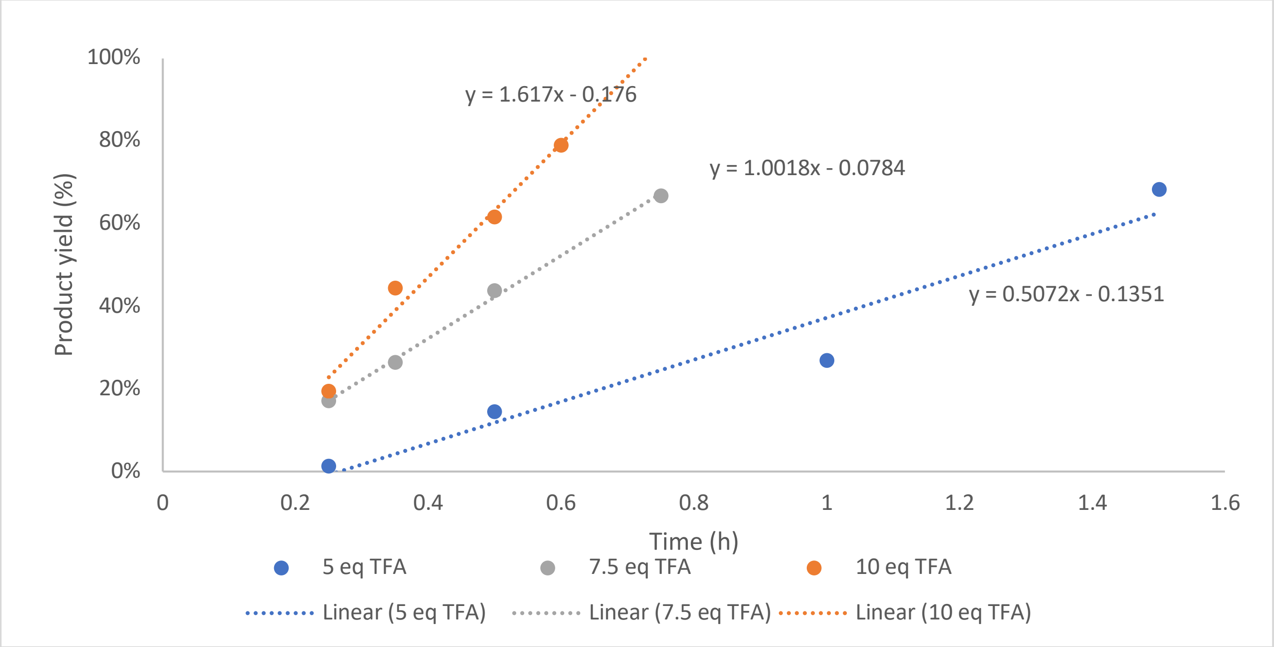

Graph of normalised rate of product formation against normalised relative TFA loading

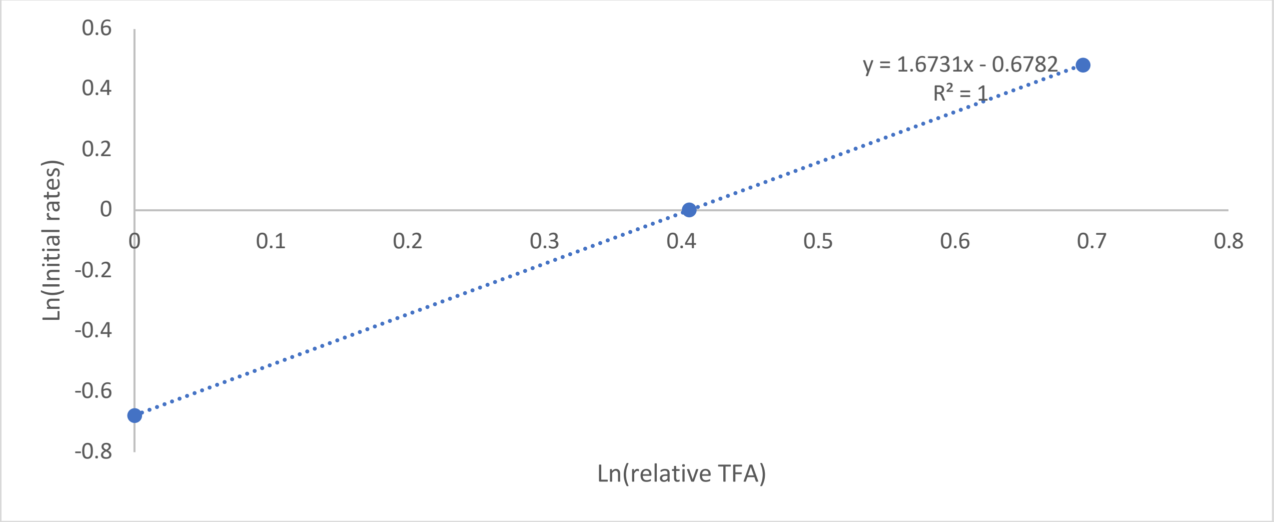

A clear positive reaction order in TFA can be observed, with an approximate TFA order of 1.6

*p-Me Carboxyl substrate **1b***

Time (h)	TFA eq. (rel eq.)	Boc SM %	de-Boc SM %	***p*-Toluate %**	Product %
0.5	5 (1)	60	40	0	0
1.0	5 (1)	53	39	0	9
2.0	5 (1)	28	36	14	22
3.0	5 (1)	11	23	21	45
1.0	7.5 (1.5)	39	49	*signals merged with SM*	7
1.5	7.5 (1.5)	31	33		30
2.0	7.5 (1.5)	27	29		37
2.5	7.5 (1.5)	20	28		44
1.0	10 (2)	36	31	*signals merged with SM*	11
1.5	10 (2)	33	32		23
2.0	10 (2)	23	37		30
2.5	10 (2)	20	27		40

Relative TFA eq.	Ln(relative TFA)	Initial Rate (%/time)	Ln(initial rate)
1.0	0.000	0.180	−1.72
1.5	0.405	0.272	−1.30
2.0	0.693	0.185	−1.69

*p-Me Carboxyl substrate **1b**: plot of product formation over time at varying TFA loading*

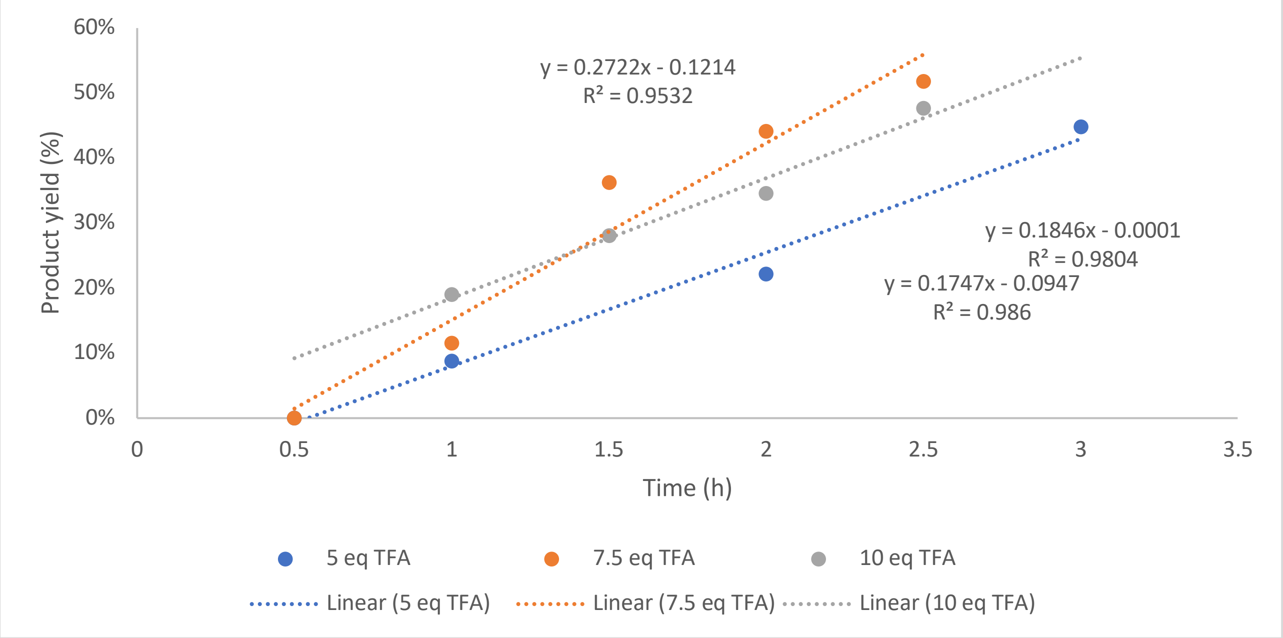

Graph of normalised rate of product formation against normalised relative TFA loading

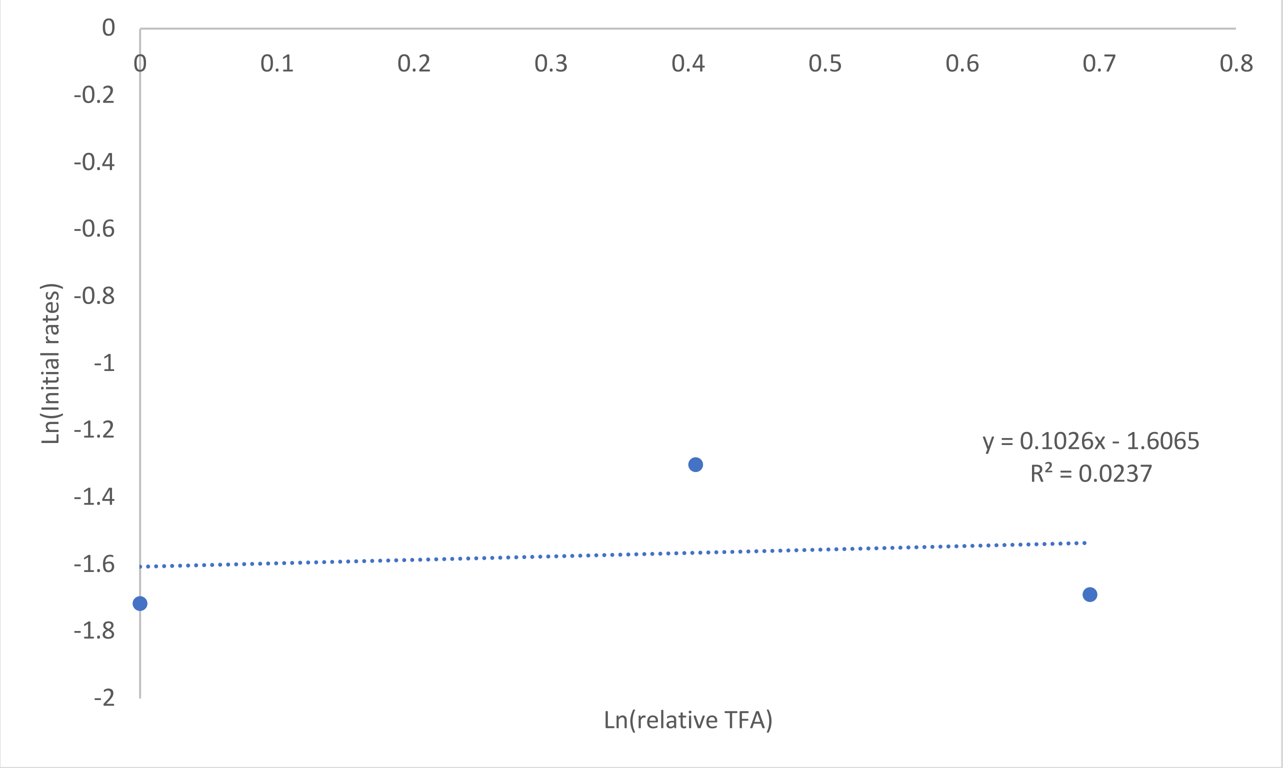

No clear reaction order in TFA at reaction relevant concentrations can be observed through a normalised relative TFA and initial rates plot – see below for further studies to probe this observation

Reaction order in NaTFA for ammonium triflate salt 9a

Two possibilities could account for a lack of observed order in TFA for carboxyl substrate **1b**; either TFA participation was indeed absent at the RDTS, or that the reaction was exhibiting saturation behaviour with respect to TFA at reaction-relevant stoichiometries. The latter case could be probed in principle by reducing TFA to sub-stoichiometric quantities. However, analysing this scenario using Boc-protected precursor **1b** would confound results as low TFA loadings would begin to impact the efficacy of the Boc deprotection that precedes N–O bond cleavage. As such, we elected to analyse its importance through the protonated triflate salt of the deprotected acyl *O*-hydroxylamine, where we see reactivity through addition of NaTFA.

Time (h)	NaTFA eq	Benzoate observed*	Product %
1.5	0.25	27	0
2.0	0.25	28	2
2.5	0.25	25	3
4.0	0.25	25	4
5.0	0.25	27	5
1.75	0.35	36	17
2.0	0.35	24	17
3.0	0.35	23	23
4.0	0.35	30	27
1.0	0.5	30	0
1.5	0.5	10	28
2.0	0.5	10	32
2.5	0.5	11	39
1.0	1.0	4	20
1.5	1.0	6	24
2.0	1.0	9	31
2.5	1.0	16	32

*No starting material was observed after quenching and filtration over SiO_2

Relative NaTFA eq.	Ln(relative NaTFA)	Initial Rate (%/time)	Ln(initial rate)
0.25	–1.386	0.012	–4.39
0.35	–1.050	0.473	–3.05
0.5	–0.691	0.110	–2.45
1.0*	0	0.086	–2.20

*Value at 1.0 NaTFA eq. excluded from analysis as it is clear that reaction has reached saturation with respects to NaTFA

Protonated triflate salt **9a**: *plot of product formation over time at varying NaTFA loading*

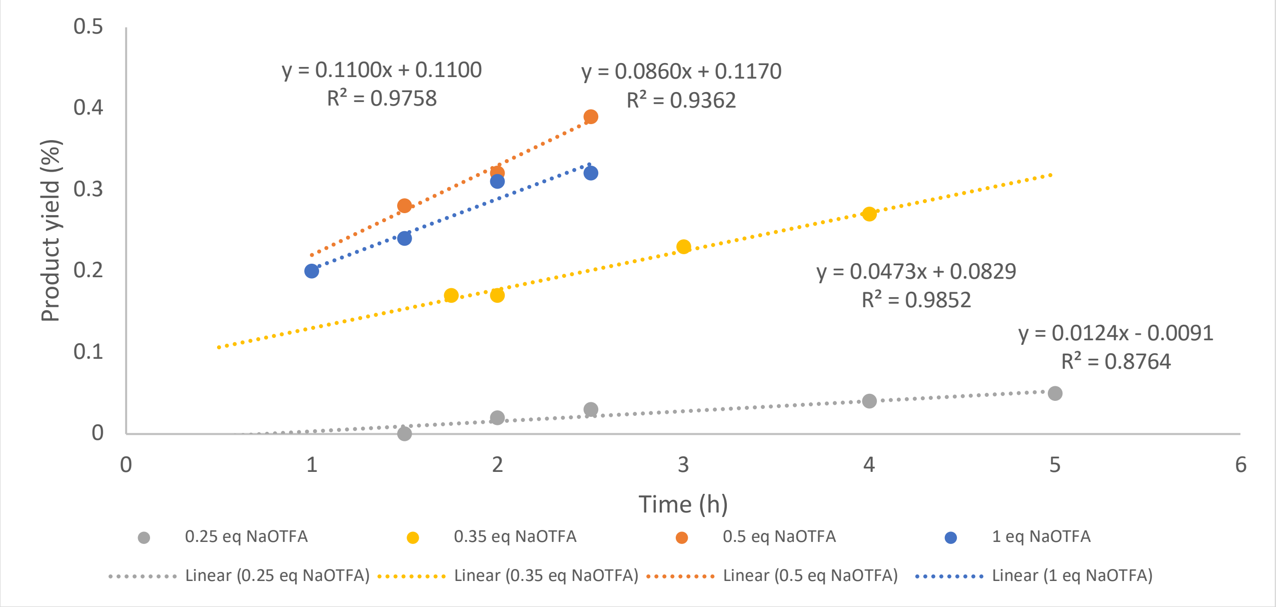

Graph of normalised rate of product formation against normalised relative TFA loading

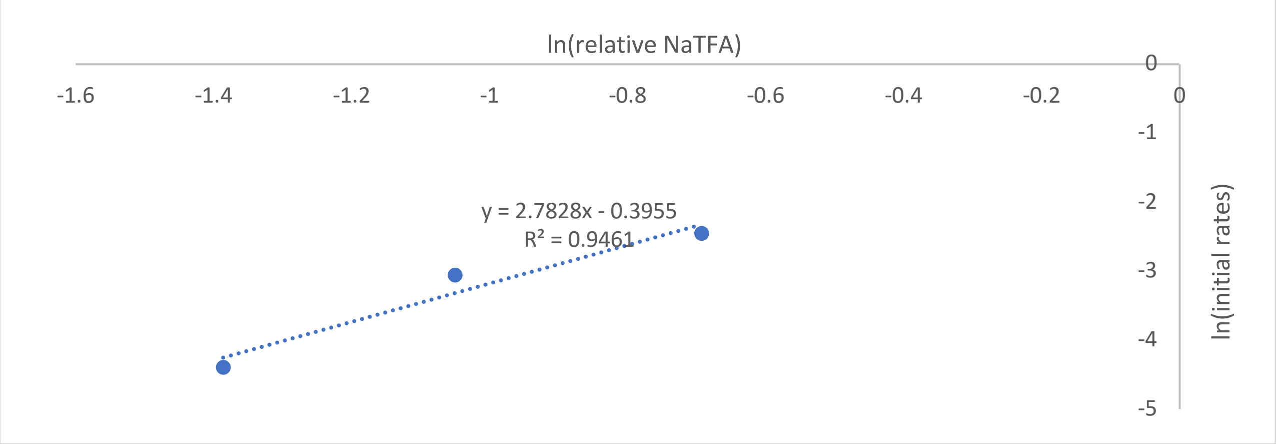

While no reaction order was seen with TFA with carboxyl substrates (at 5, 7.5 and 10 eq.), it is clear that at amounts less than 0.5 eq. the reaction becomes ordered in NaTFA. The presence of a reaction order with respects to TFA confirms its presence at the RDTS and aligns it in behaviour to sulfonyl substrates where a clear reaction order in TFA is observed at relevant reaction stoichiometries. This analysis also suggests there is approximately a reaction order of 2.7 in NaTFA (at < 0.5 eq.) for substrate **9a**.

The result also suggests that the reaction reaches saturation with respects to TFA at sub-stoichiometric quantities and implies that the resting state for the carboxyl substrate rests as the TFA-complex substrate, where a zero reaction order reflects no molecularity change between the resting state and the RDTS (rather than a lack of involvement at the RDTS). As TFA fundamentally alters the resting state of the reaction for carboxyl substrate, we therefore expect to see a similar zero order dependence in TFA at reaction relevant quantities for the Fe free reaction also.

RDTS determination: reaction order in *p*-toluenebenzene sulfonate

Our reaction order analysis operates under the assumption that the rate of product formation enables us to indirectly infer the effects at the rate determining step of the reaction (either i. N–O bond cleavage or ii. arene amination). By its nature, arene amination must be the product determining step of the reaction, however, this does not give an indication on which of these two steps are overall rate determining for the pathway, and so the experiments in this section were designed to probe where along the reaction pathway this may be. A caveat is that the two steps may have similar barriers, though our reaction order analysis and related mechanistic studies (*J. Am. Chem. Soc.* **2022***, 144,* 2637−2656) indicate that this is highly unlikely.

Given that arene amination is the only bimolecular step in the reaction pathway that involves the sulfonate anion, and we previously established that intermolecular amination is kinetically feasible in crossover experiments, we inferred the position of the RDTS by reaction order analysis with exogenously added [*p*-TsO][Et_4N],

Time (h)	[Et_4N][TsO] added (total TsO)	*p*-toluenesulfonate %*	Product %
0.25	0 (1)	100	0
0.50	0 (1)	85	14
1.0	0 (1)	38	57
1.5	0 (1)	27	66
0.50	0.25 (1.25)	70	30
0.75	0.25 (1.25)	50	50
1.0	0.25 (1.25)	39	61
1.25	0.25 (1.25)	38	62
0.50	0.5 (1.5)	85	15
0.75	0.5 (1.5)	56	44
1.0	0.5 (1.5)	53	47
1.25	0.5 (1.5)	32	67

Relative TFA eq.	Ln(relative TFA)	Initial Rate (%/time)	Ln(initial rate)
1.0	0.000	0.622	–0.475
1.25	0.223	0.434	–0.834
1.5	0.405	0.653	–0.425

**p*-toluenesulfonate remaining calculated by total integration subtracted by added TsO^-

RDTS determination: rate of product formation against arene electronics

Time (h)	R group	Boc SM %	de-Boc SM %	*p*-R acid %	Product %
0.5	*p*-Me	60	40	0	0
1.0	*p*-Me	53	39	0	9
2.0	*p*-Me	28	36	14	22
3.0	*p*-Me	11	23	21	45
0.75	*p*-F	50	28	14	8
1.5	*p*-F	15	38	32	15
2.0	*p*-F	0	38	42	21
2.5	*p*-F	0	40	33	27
0.75	*p*-CF_3	44	49	5	1
1.0	*p*-CF_3	10	46	36	8
1.5	*p*-CF_3	0	49	40	11
2.0	*p*-CF_3	0	18	55	26

Substituent	σ_{meta}	σ_{para}	Initial Rate (%/time)	Ln(initial rate)
p-Me	–0.07	–0.17	0.180	–1.72
p-F	0.33	0.06	0.107	–2.23
p-CF_3	0.43	0.54	0.153	–1.87

A corresponding investigation with arene electronics was not conducted with sulfonyl substrate given that electron withdrawing sulfonates result in a shift in the resting state (as well as the resultant mechanistic behaviour) favouring TFA displacement with the sulfonyl substrate. Similarly, a corresponding investigation of toluic acid loading was not conducted owing to a limited viability of intermolecular aminative capture, as well as the potential importance of the carboxylate anion (masked through a coordination complex with iron) in the reaction—the use of toluate anion in TFA would result in immediate protonation and would confound results.

Plot of product formation over time at different *p*-toluenebenzene sulfonate loading

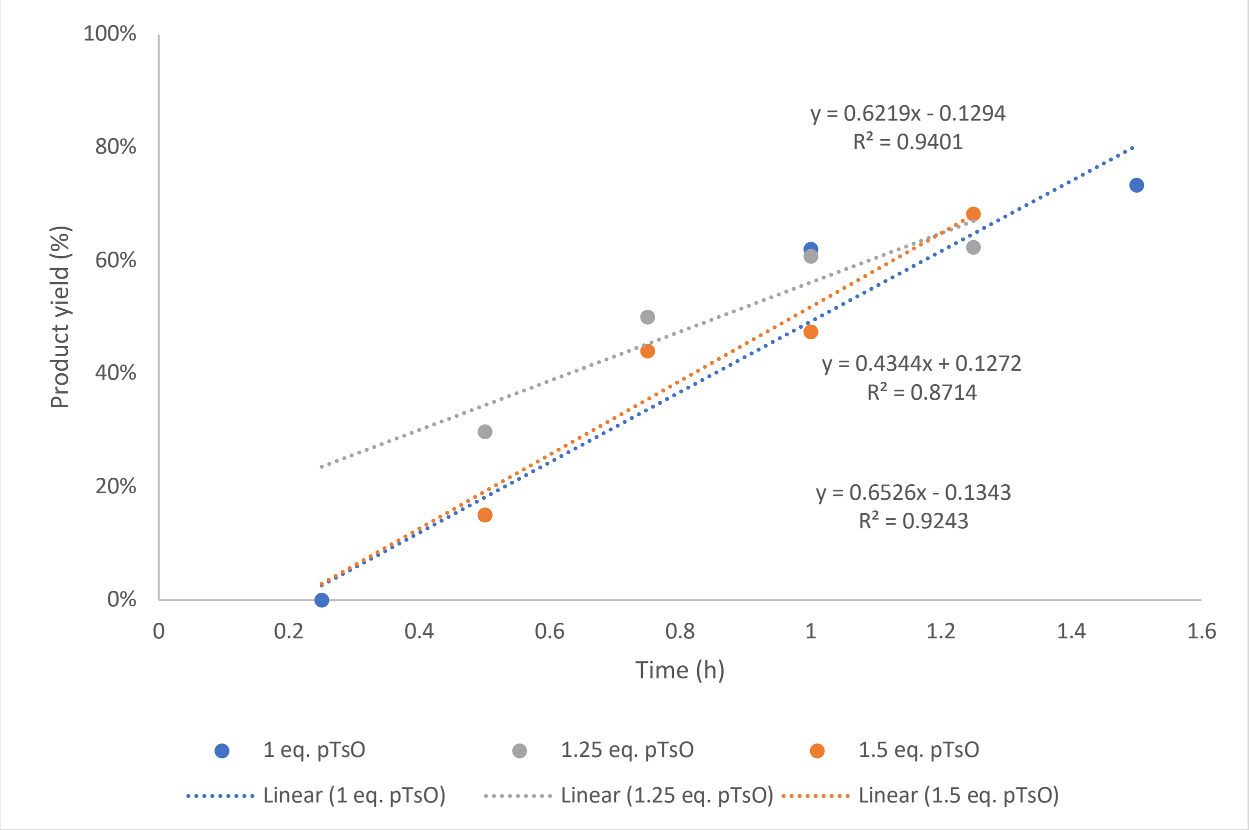

Graph of normalised rate of product formation against normalised relative [p-TsO] loading

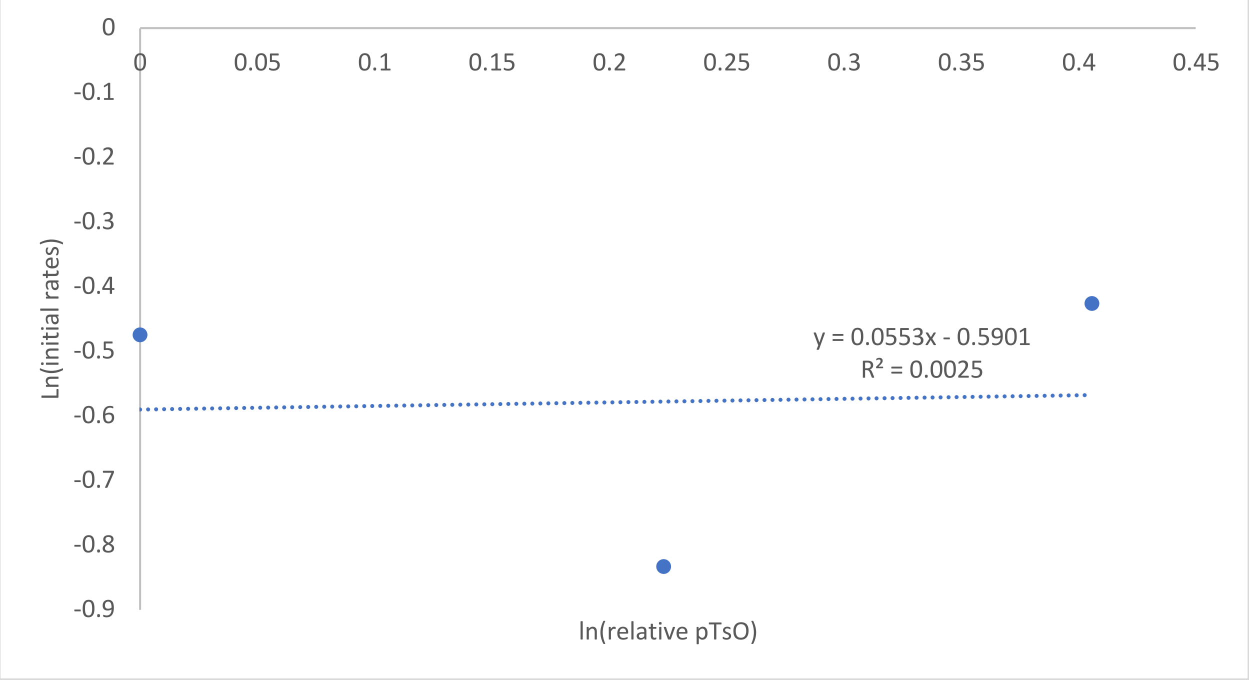

No clear reaction order in [pTsO] can be observed through a normalised relative pTsO and initial rates plot

Plot of product formation over time with different arene electronics

To probe whether arene amination may be rate determining in this case, we first considered an analogous investigation by exogenously doping in the corresponding substituted benzoate. In addition to a limited viability of an intermolecular amination process for these substrates, we were cognisant that the pKa difference (>4) between TFA and the carboxylate would likely result in rapid protonation of the latter; the same issue would not arise with the anionic sulfonate due to its lower basicity. As these effects could complicate interpretation, we elected to probe the RDTS position by altering the arene electronics to assess whether rate difference in product formation can be observed

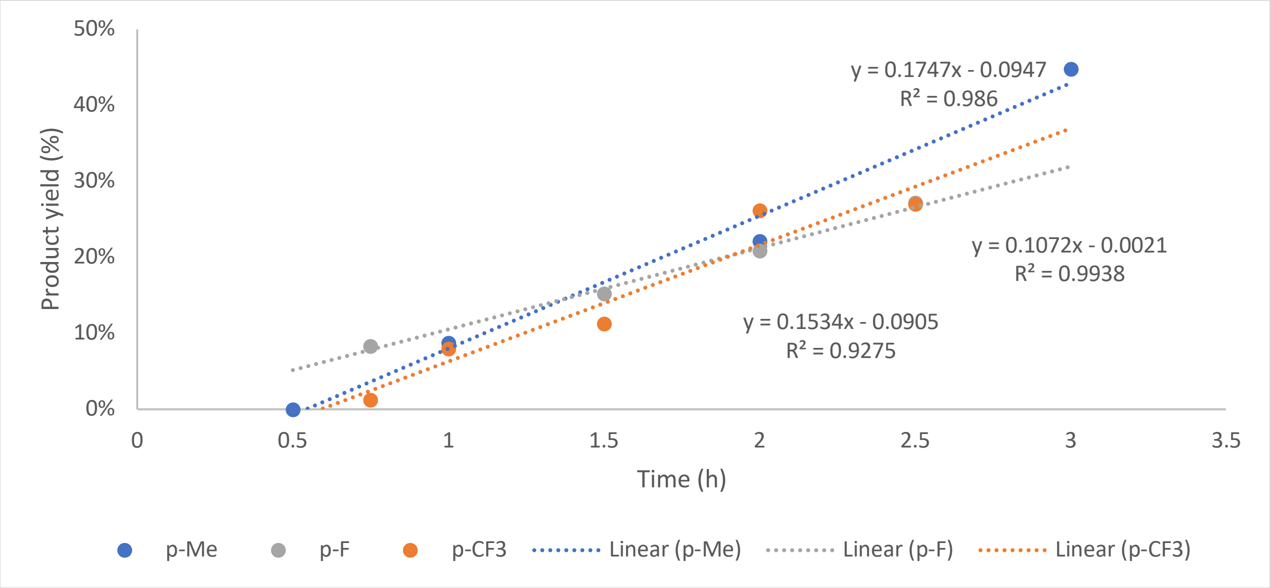

Graph of normalised rate of product formation against Hammett values

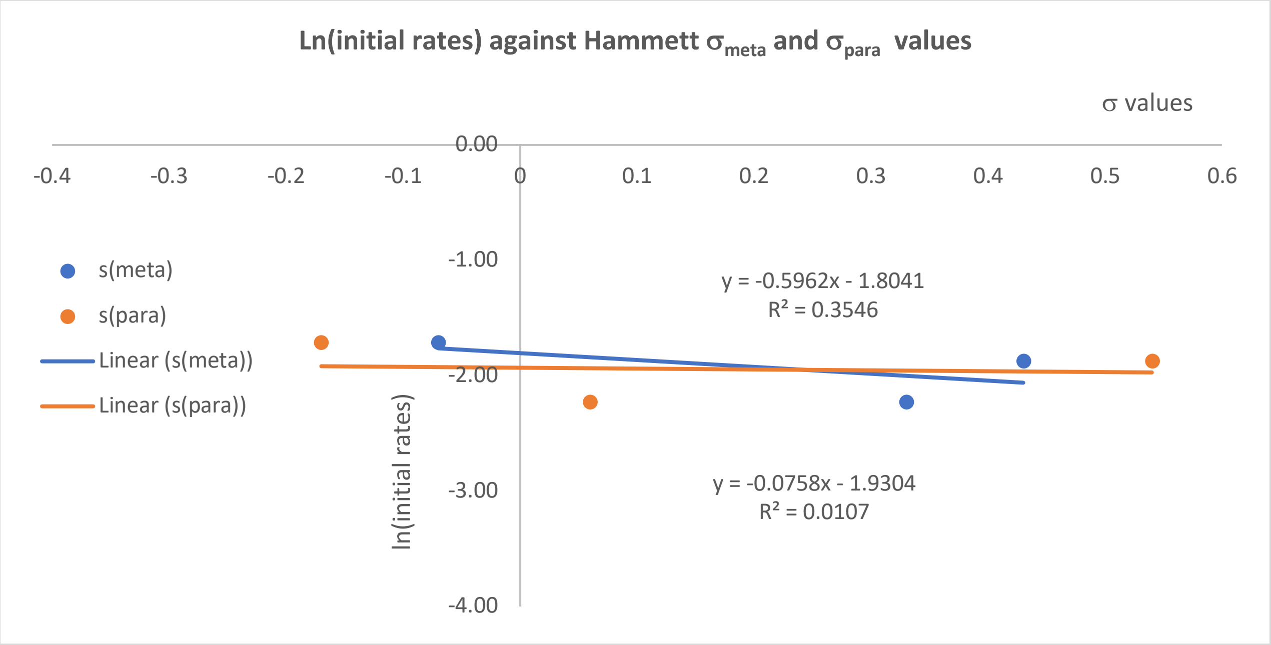

No clear rate differences observed with varying arene electronics for carboxyl substrates

Both results here show a clear lack of dependence on attributes associated with the arene amination elementary step. If the RDTS was positioned at this step, the sulfonyl substrate would exhibit a positive order with respects to the *p*-TsO substrate added. Similarly, rate deceleration for product formation would be expected for carboxyl substrate **1** with more electron withdrawing substrates.

Collectively, these observations led us to conclude that TFA is involved at the N–O bond cleavage RDTS for both sulfonyl and carboxyl substrates. For carboxyl substrates, a lack of TFA order under reaction-relevant conditions reflects a lack of molecularity change between the resting state and the RDTS.

9.8 Time Course Investigation

Sulfonyl substrate ***10***

A solution of sulfonyl substrate **10** (72.0 mg, 0.25 mmol) was stirred in CH_2Cl_2 (5 mL) at 40 °C (for reactions conducted with iron, $FeSO_4{\cdot}7H_2O$ (0.7 mg, 2.5 μmol) was added with the substrate at the beginning). TFA (100 μL, 1.25 mmol) was next added and the reaction was stirred for the indicated time before quenching with Et_3N (200 μL). For reactions conducted with iron catalyst, the resulting mixture was filtered through a thin plug of SiO_2 and was next washed (20% MeOH/CH_2Cl_2). In all cases, the combined organic solvents/filtrates were concentrated under a stream of air. ^1H NMR with 1,2-dimethoxyethane as an internal standard was used to determine the yield of reaction components, noting that upon Et_3N addition the intermediary sulfonyl hydroxylamine will be displaced by the tertiary amine to generate the corresponding sulfonate.

Carboxyl substrate ***1b***

A solution of carboxyl substrate **1b** (62.0 mg, 0.25 mmol) was stirred in TFE (5 mL) at 40 °C (for reactions conducted with iron, $FeSO_4{\cdot}7H_2O$ (0.7 mg, 2.5 μmol) was added with the substrate at the beginning). TFA (100 μL, 1.25 mmol) was next added and the reaction was stirred for the indicated time before quenching with Et_3N (200 μL). For reactions conducted with iron catalyst, the resulting mixture was filtered through a thin plug of SiO_2 and was next washed (20% MeOH/CH_2Cl_2). In all cases, the combined organic solvents/filtrates were concentrated under a stream of air. ^1H NMR with 1,2-dimethoxyethane as an internal standard was used to determine the yield of reaction components.

Normalised yields are used for analysis to mitigate experimental errors arising from setting up, purifying and analysing individual time points, which are conducted as individual runs.

Plot of reaction over time with sulfonyl substrate (10) with iron

Table of obtained raw reaction yields

Time (h)	Sulfonate %	Product (11) %
0.25	80	1
0.5	77	13
1.0	68	25
1.5	20	43
2.0	17	72
4.0	13	80
8.0	13	80

All reaction yields were normalised against each other to mitigate experimental variability. In the case of sulfonyl substrate, no other product or starting material related resonances were observed except at t = 0.25, where a minor amount of Boc-protected starting material was observed; this was represented as a summative yield (*) with sulfonate and Boc starting material.

Plot of raw reaction yields over time

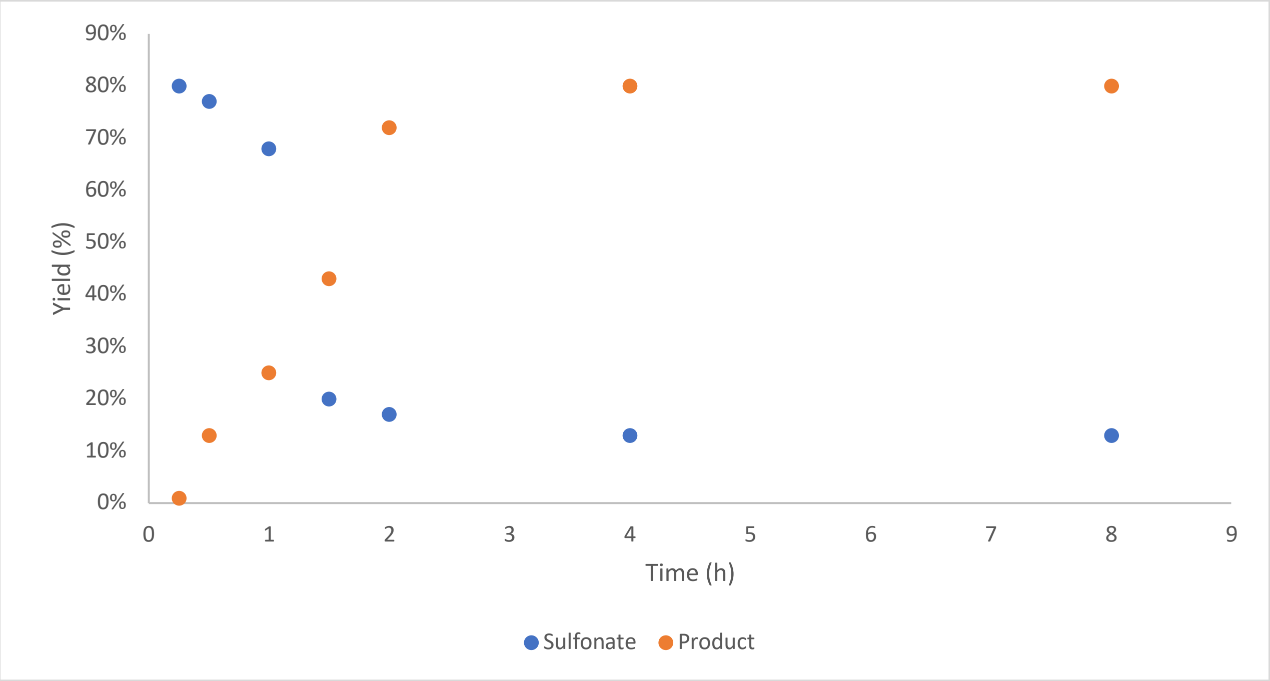

Plot of normalised reaction yields over time

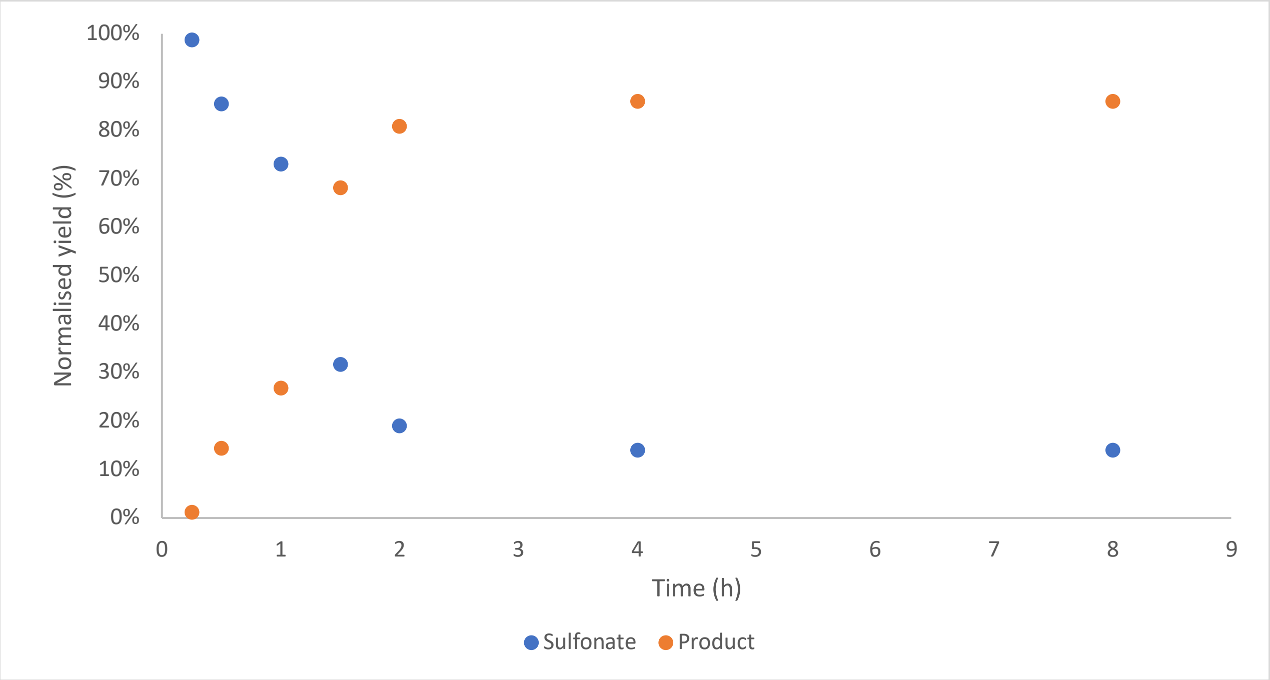

Plot of reaction over time with sulfonyl substrate (10) without iron

Table of obtained raw reaction yields

Time (h)	Sulfonate %	Product (11) %
0.25	99	0
0.5	79	14
1.0	35	57
1.5	24	66
2.0	21	73
3.0	17	82

Plot of raw reaction yields over time

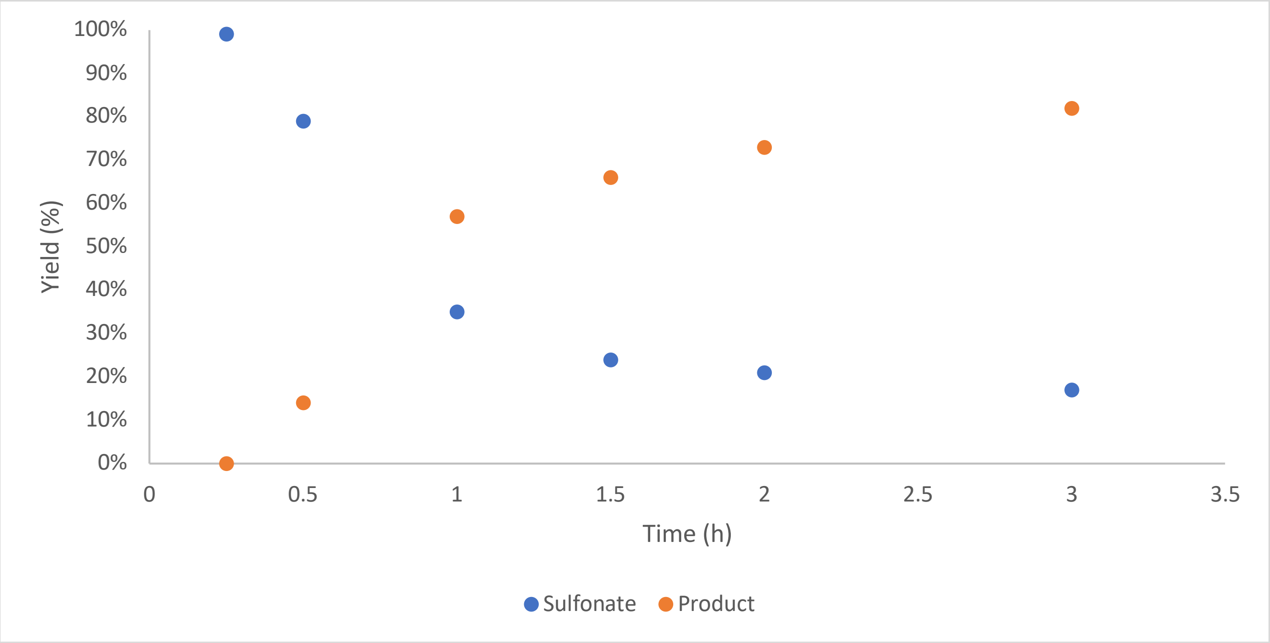

Plot of normalised reaction yields over time

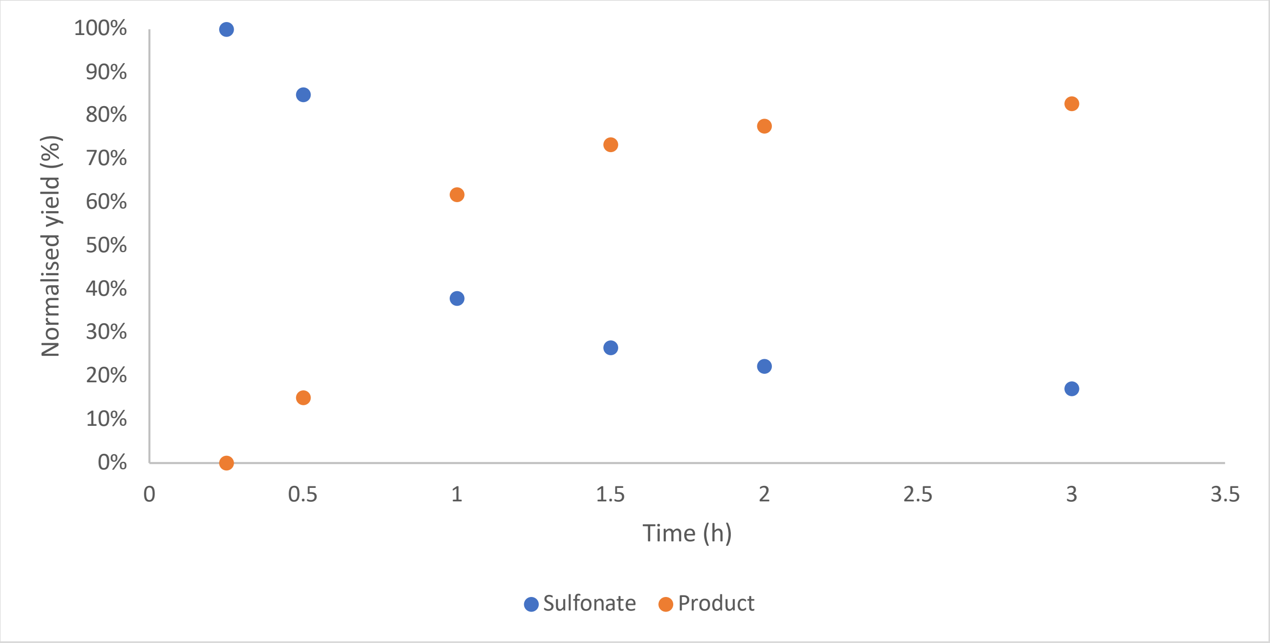

We note that the aminative rearrangement for sulfonyl substrate proceeds very cleanly, and after work up the two major products observed was either the product, or the sulfonate (arising from an S_N2 amination between NEt_3 with the active aminating agent or its intermediate. A comparable rate of amination was observed for the *p*-Me substrate with and without iron, suggesting that a rapid radical amination mechanism is the predominant driving force for reactivity in this process.

Plot of reaction over time with carboxyl substrate (1b) with iron

The reaction profile is considerably more complex for carboxyl substrate **1b** (see ^{1}H NMR spectra over time below). Putative assignments are given between three starting material related components (Boc SM, corroborated with integration between arene, Me and Boc signals, de Boc SM, corroborated by its time dependent appearance and disappearance, and the corresponding carboxylate). We note that it is often challenging to map conclusively the signals to an external structure given variability in H-bonding, protonation state and concentration dependence on chemical shift for acyl-containing substrates.

Table of obtained raw reaction yields

Time (h)	Boc SM 1b (%)	deBoc SM (%)	Carboxylate (%)	Product 2b' (%)
0.5	47	31	0	0
1.0	42	31	0	9
2.0	24	31	14	22
3.0	11	22	21	43
4.0	10	21	22	45
8.0	9	12	20	50
16	1	18	23	53

Plot of raw reaction yields over time

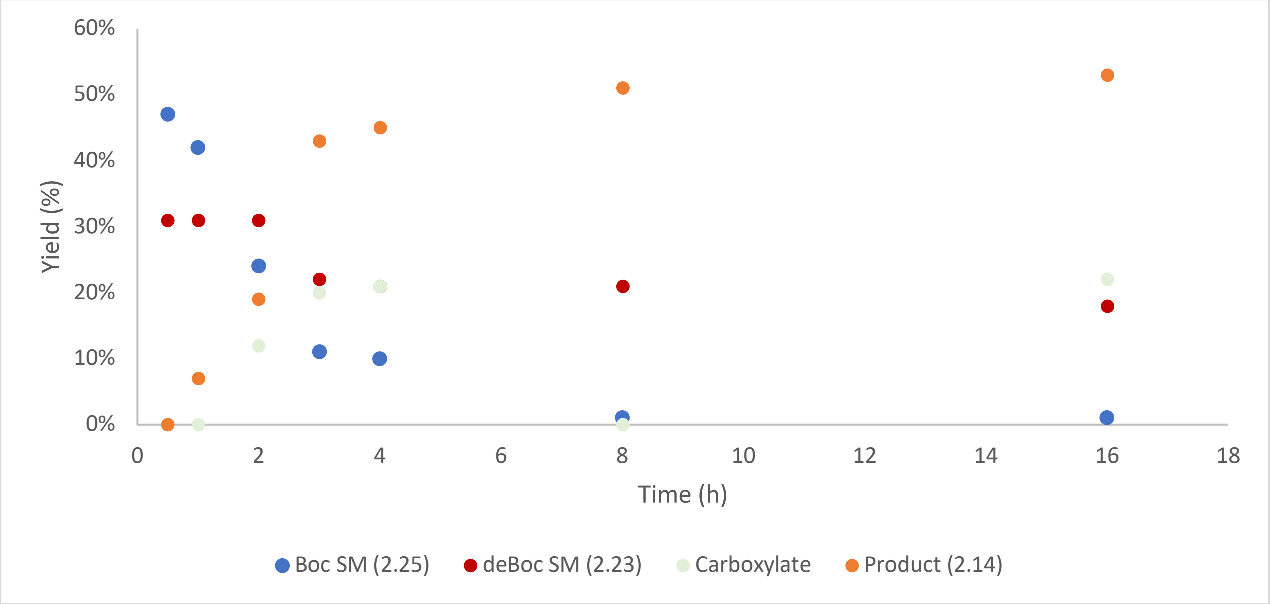

Plot of normalised reaction yields over time

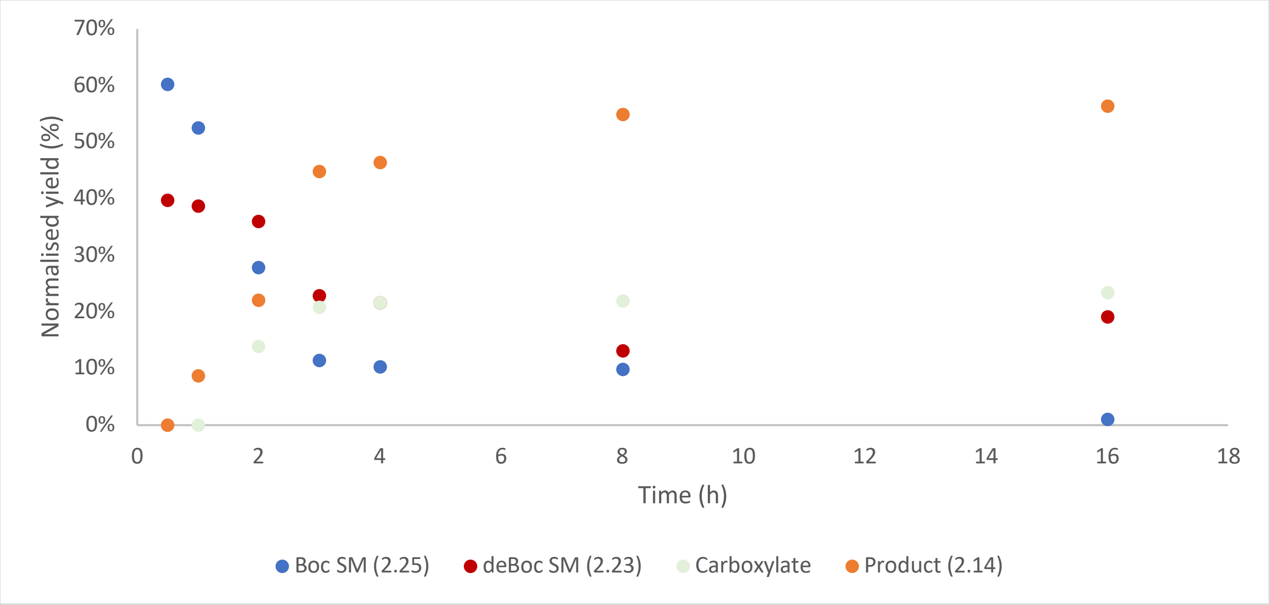

Plot of reaction over time with carboxyl substrate (1b) without iron

Table of obtained raw reaction yields

Time (h)	Boc SM 1b (%)	deBoc SM (%)	Carboxylate (%)	Product 2b' (%)
0.5	50	38	0	0
1.0	48	45	0	2
1.5	35	56	0	2
2.0	21	60	0	2
3.0	13	48	16	2
4.0	5	21	22	15
5.0	4	11	34	41
6.0	7	8	25	56
8.0	1	8	20	57
12	0	9	14	53
16	0	8	25	59

Plot of raw reaction yields over time

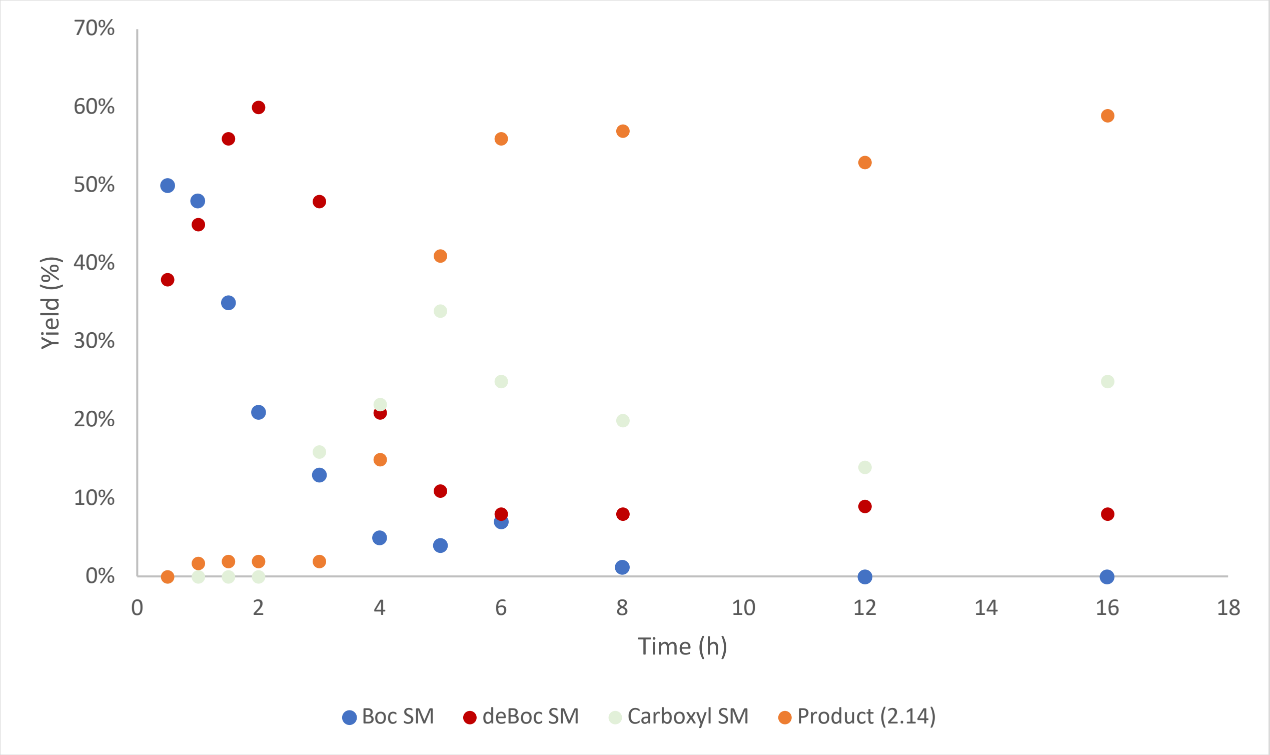

Plot of normalised reaction yields over time

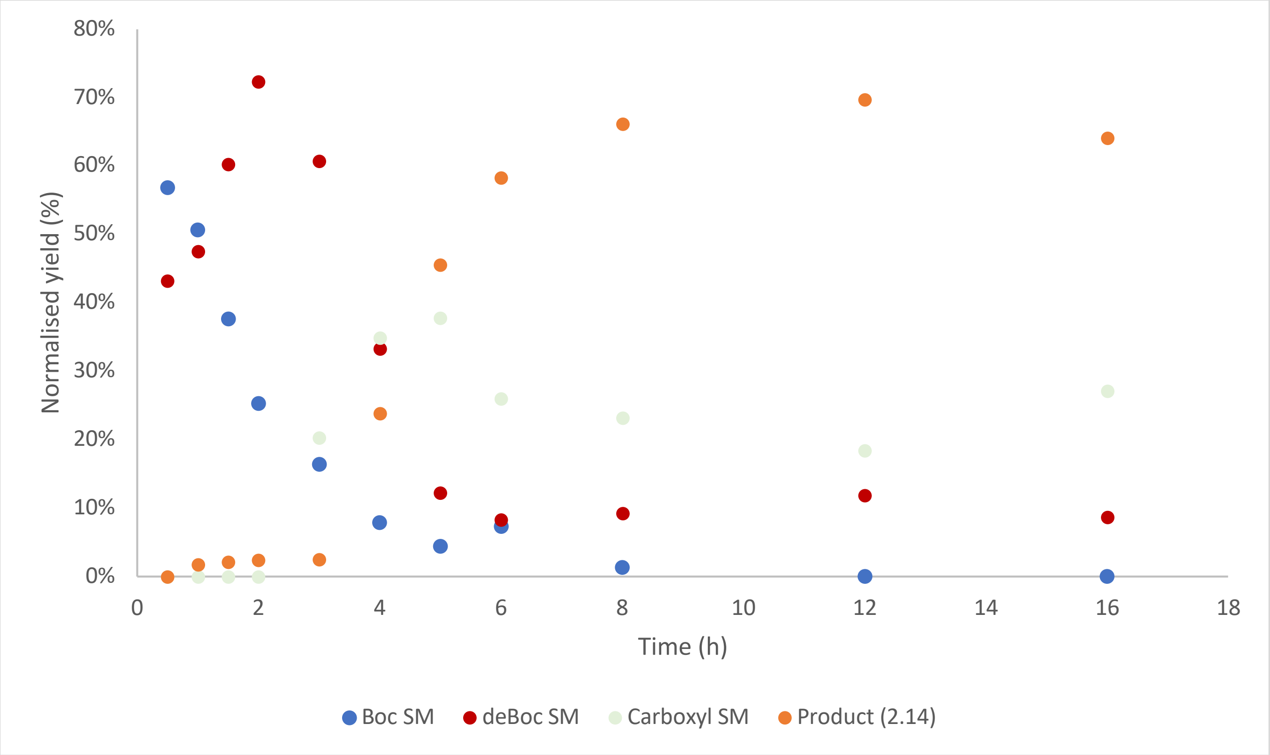

Illustrative ^{1}H NMR of reaction over time with sulfonyl substrate (10) without iron

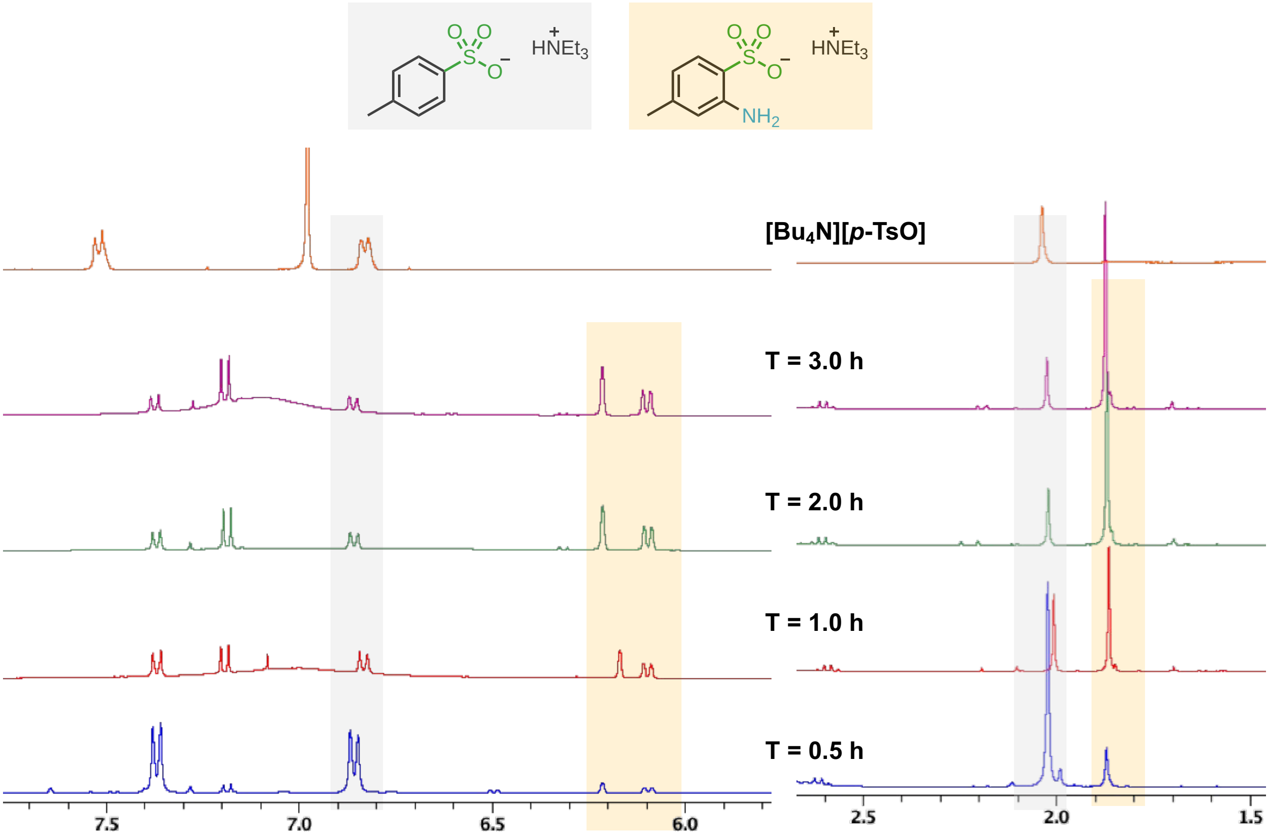

Illustrative ^{1}H NMR of reaction over time with sulfonyl substrate (10) with iron

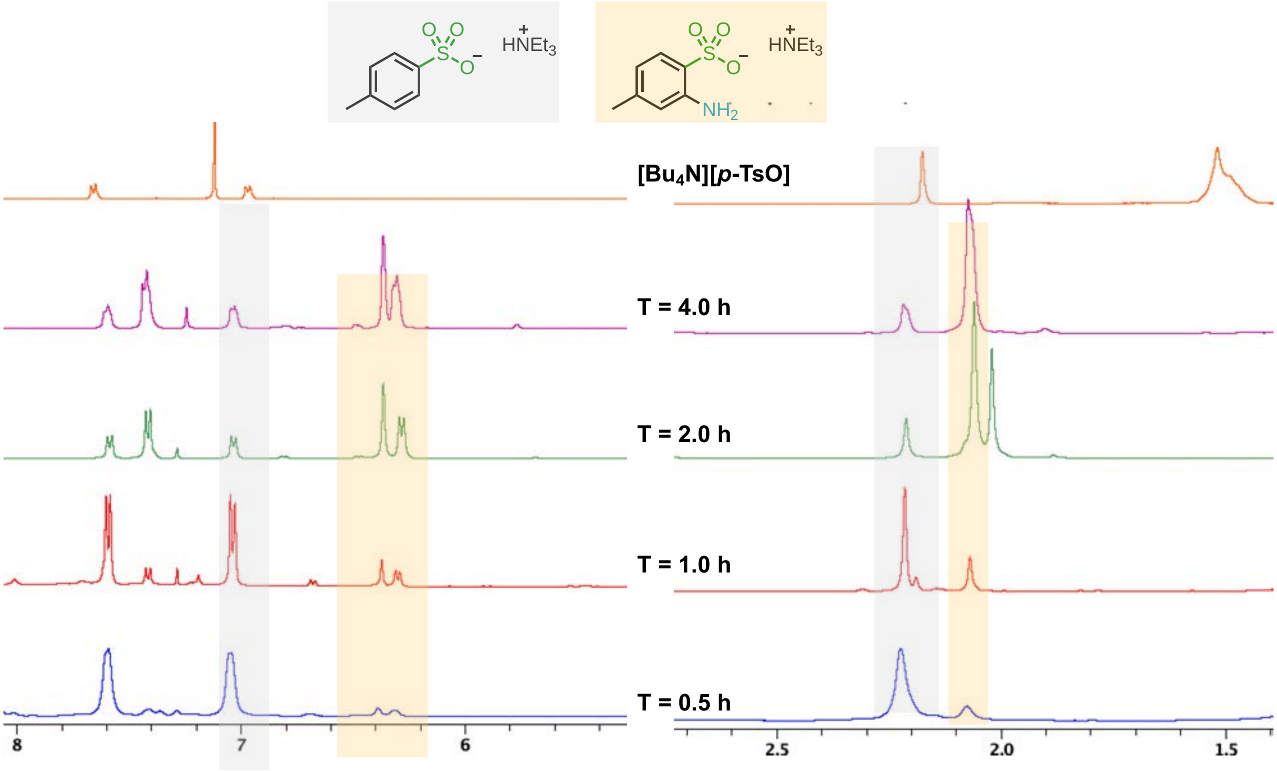

Illustrative ^{1}H NMR of reaction over time with carboxyl substrate (1b) without iron

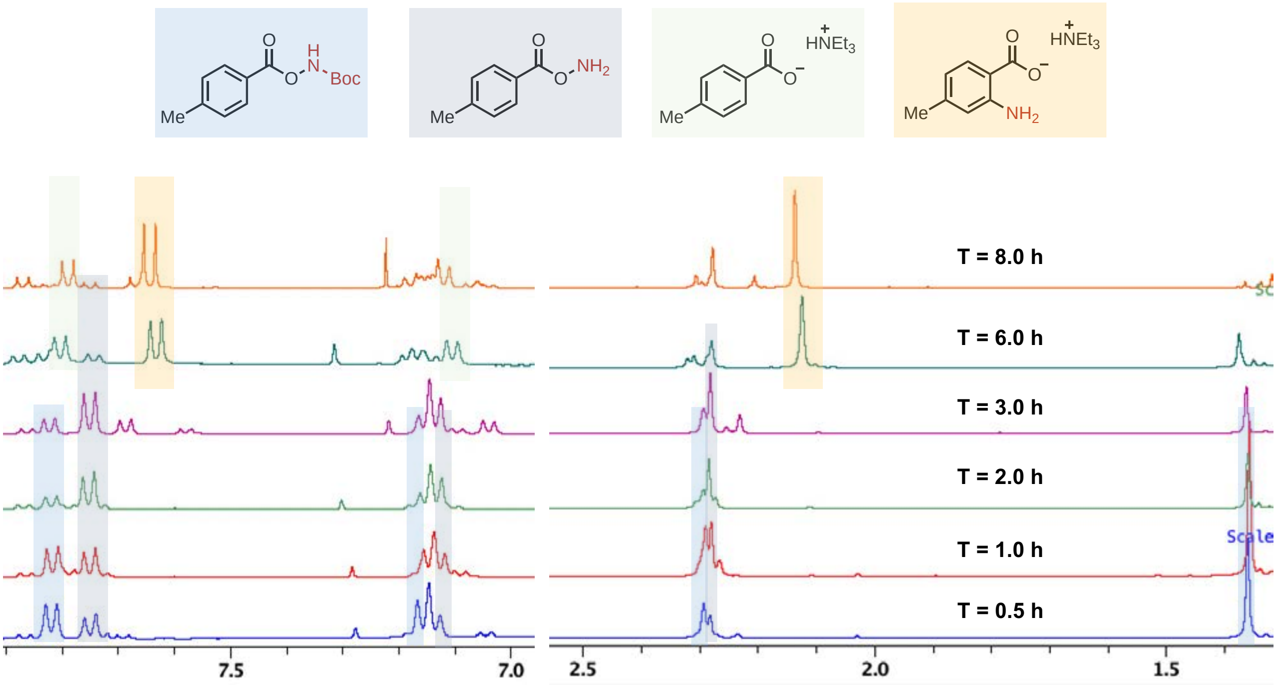

Illustrative ^{1}H NMR of reaction over time with carboxyl substrate (1b) with iron

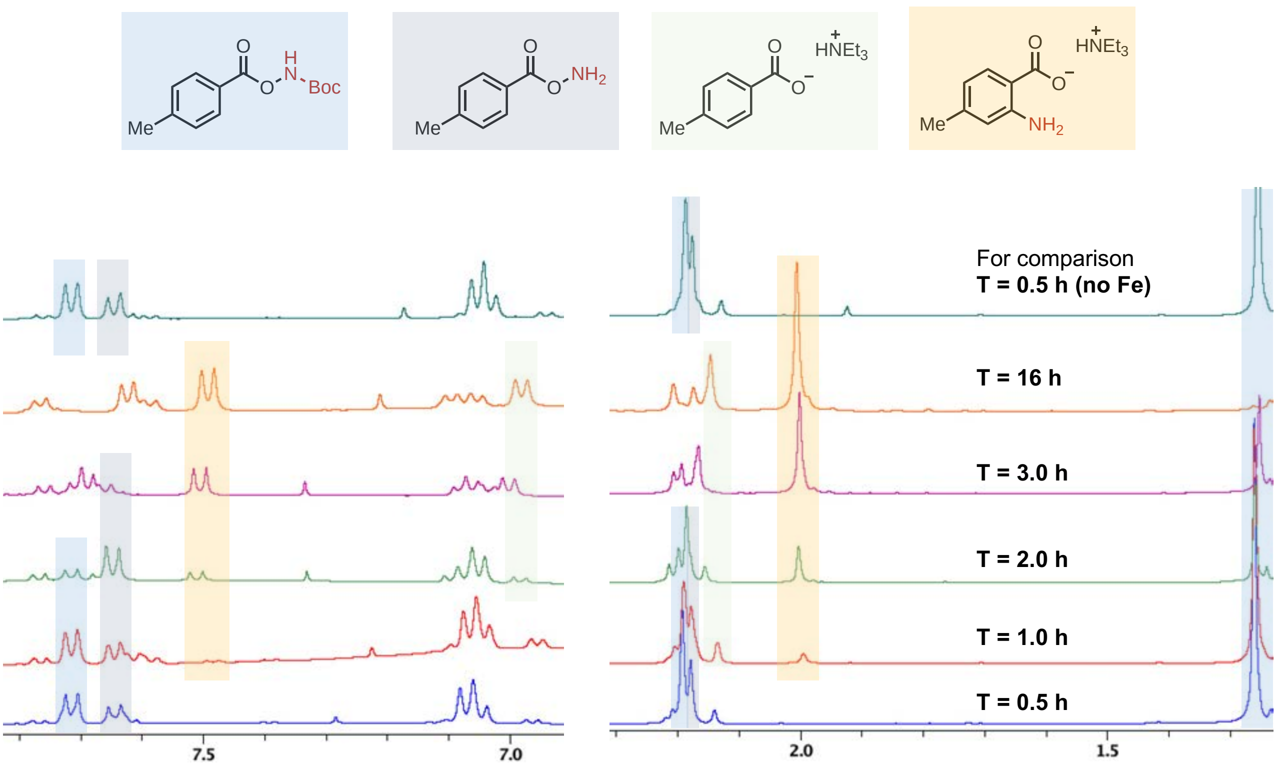

The reaction profile is more complex with carboxyl substrate **1b** particularly in comparison with sulfonyl substrate **10**, though all intermediates are starting material related noted by the clear *para*-substitution pattern and their appearance and disappearance over time. We note that a precise determination of intermediate yields is not straightforward based on our analytical methods, and so we have not used these to derive meaningful kinetic/reaction order data and focus instead on % product formation as our key indicator for all mechanistic studies. Finally, we note that conclusions reached from the kinetic studies outlined above requires the assumption that the product determining step is an accurate read-out of a *singular* RDS in the reaction pathway (downstream/upstream steps minimally impact the rate of product formation/no other steps with comparable barriers to RDS). Our studies, as well as literature precedents on related systems (*J. Am. Chem. Soc.* **2022**, *144*, 2637–2656) indicate that this assumption holds and that under standard reaction conditions, no other steps have a comparable barrier to the rate-determining N–O bond cleavage step.

9.9 Mechanistic Probes

TEMPO inhibition study

*Sulfonyl substrate **10***

A solution of sulfonyl substrate **10** (72.0 mg, 0.25 mmol) and TEMPO (0.2, 0.5 or 1.0 eq.) was stirred in CH_2Cl_2 (5 mL) at 40 °C (for reactions conducted with iron, $FeSO_4 \cdot 7H_2O$ (0.7 mg, 2.5 μmol) was added with the sulfonyl substrate at the beginning). TFA (100 μL, 1.25 mmol) was next added and the reaction was stirred for the indicated time before quenching with Et_3N (200 μL). For reactions conducted with iron catalyst, the resulting mixture was filtered through a thin plug of SiO_2 and was next washed (20% $MeOH/CH_2Cl_2$). In all cases, the combined organic solvents/filtrates were concentrated under a stream of air. ^1H NMR with 1,2-dimethoxyethane as an internal standard was used to determine the yield of reaction components.

*Carboxyl substrate **1b***

A solution of carboxyl substrate **1b** (62.0 mg, 0.25 mmol) and TEMPO (0.2, 0.5 or 1.0 eq.)) was stirred in TFE (5 mL) at 40 °C (for reactions conducted with iron, $FeSO_4 \cdot 7H_2O$ (0.7 mg, 2.5 μmol) was added with the sulfonyl substrate at the beginning). TFA (100 μL, 1.25 mmol) was next added and the reaction was stirred for the indicated time before quenching with Et_3N (200 μL). For reactions conducted with iron catalyst, the resulting mixture was filtered through a thin plug of SiO_2 and was next washed (20% $MeOH/CH_2Cl_2$). In all cases, the combined organic solvents/filtrates were concentrated under a stream of air. ^1H NMR with 1,2-dimethoxyethane as an internal standard was used to determine the yield of reaction components.

TEMPO inhibition study indicate that a radical chain process is in process for sulfonyl substrate **10** and for carboxyl substrate **1b** under iron free condition, which is broken and inhibited through its addition

	From sulfonyl substrate 10		From carboxyl substrate 1b	
TEMPO loading	SM*	Product (11)	SM**	Product (2b')
-	0	70	18	53
0.2	33	52	21	35
0.5	56	20	30	29
1.0	67	6	47	26
1.0 (no Fe)	67	0	86	6
2.0	-	-	48	25

* as corresponding sulfonate **as a sum of deprotected starting material and corresponding benzoate

Synthesis and reactivity of styrenyl substrate 12

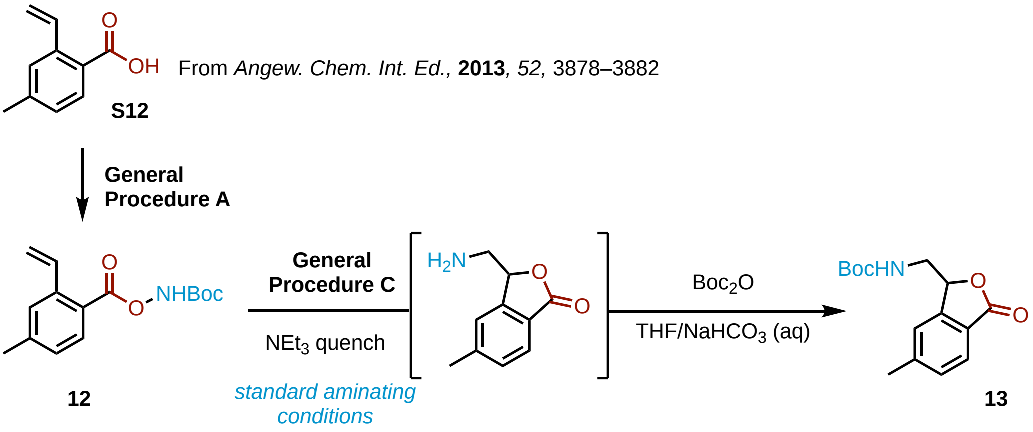

*Synthesis of styrenyl substrate **12***

The starting acid **S12** was synthesised according to known literature procedure published by Dydio and Reek (*Angew. Chem. Int. Ed.*, **2013**, *52*, 3878–3882). Following General Procedure A, the free acid **S12** (164 mg, 1.01 mmol, 1.01 eq.) was used to generate the product **12** as a yellow oil (270 mg, 0.975 mmol, 97%).

^{1}H NMR (400 MHz, $CDCl_3$) δ 8.10 (br s, 1H), 7.91 (d, *J* = 8.0 Hz, 1H), 7.48–7.41 (m, 2H), 7.17 (dd, *J* = 8.0, 1.0 Hz, 1H), 5.79 (dd, *J* = 17.4, 1.2. 1H), 5.38 (dd, *J* = 11.0, 1.2, 1H), 2.42 (s, 3H), 1.52 ppm (s, 9H); ^{13}C NMR (100 MHz, $CDCl_3$) δ 166.8, 155.7, 140.1, 140.3, 135.2, 130.8, 128.4, 127.9, 122.3, 117.1, 83.3, 28.0, 21.7 ppm; HRMS calcd. for $C_{15}H_{19}NO_4H$ $[M+H]^+$ 278.1392, found 278.1390.

*Mechanistic probe with styrenyl substrate **12***

Styrenyl substrate **12** (0.25 mmol) was next subjected to standard aminating conditions (General Procedure C). Following quenching with Et_3N (200 μL), filtration over SiO_2 and washing with 20% MeOH/CH_2Cl_2, the combined filtrates were concentrated under a stream of air. ^{1}H NMR with 1,2-dimethoxyethane as an internal standard gave a ^{1}H NMR yield of the crude mixture, which indicated exclusive formation of an aminolactone product. The corresponding reaction conducted without iron did not give any product.

Crude ^{1}H NMR in d_4-MeOD showing exclusive formation of a new aminolactone product

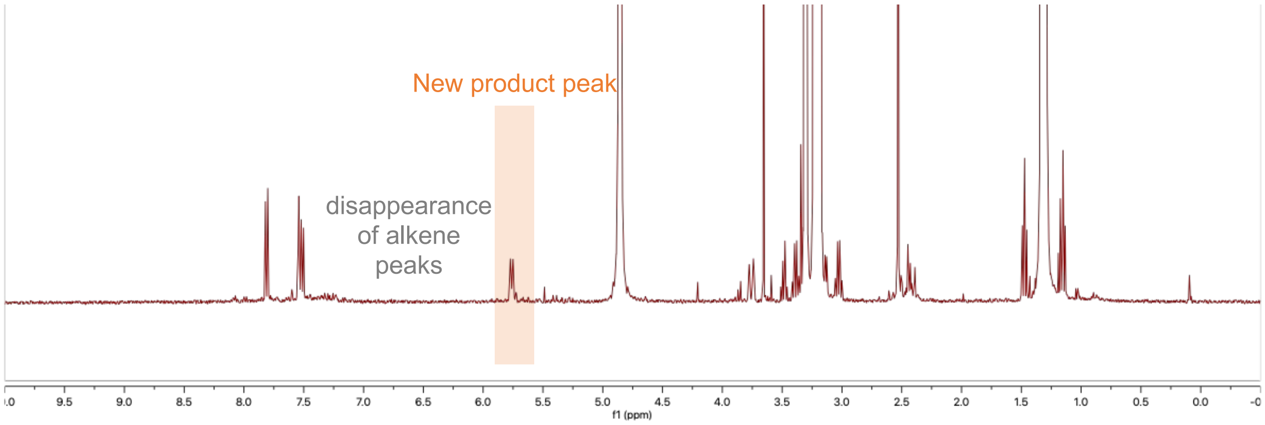

Boc protection was next conducted to i) simplify chromatographic purification if an amine functionality was present, and ii) provide orthogonal confirmation of the presence of the amine functionality in the aminolactone product if Boc reactivity took place. The above crude reaction mixture was next concentrated, resuspended in a mixture of THF (1 mL) and sat. $NaHCO_3$ (1 mL). Boc_2O (115 μL, 0.50 mmol, 2 eq.) was added and the reaction was vigorously stirred at rt for 2 h. The reaction mixture was next extracted with EtOAc (3 x 1 mL) and the combined organic extracts were dried over $MgSO_4$, filtered over SiO_2 and the combined filtrate was concentrated under a stream of air. Column chromatography of the resulting crude mixture (EtOAc/PE 10–70%) afforded the product **13** as a pale-yellow solid (14.5 mg, 52.3 μmol, 21% isolated yield over two steps).

^{1}H NMR (400 MHz, $CDCl_3$) δ 7.77 (d, J = 8.0 Hz, 1H), 7.35–7.32 (m, 2H), 5.50 (br d, *J* = 4.4 Hz, 1H), 4.93 (m, 1H), 3.86 (m, 1H), 3.36 (dt, *J* = 14.6, 6.0 Hz, 1H), 2.48 (s, 3H), 1.37 ppm (s, 9H); ^{13}C NMR (100 MHz, $CDCl_3$) δ 170.2, 155.7, 147.3, 145.5, 130.7, 125.4, 123.9, 122.9, 80.3, 79.9, 44.0, 28.2, 22.1 ppm; HRMS calcd. for $C_{15}H_{19}NO_4H$ $[M+H]^+$ 278.1392, found 278.1388.

The formation of the aminolactone implicates a preferential olefin aziridination, followed by ring opening with the adjacent benzoic acid motif. Given extensive literature precedent of iron-catalysed olefin aziridination (followed by aziridine opening to give formal amino-di-functionalisation of olefins) gives mechanistic confirmation that this is a viable pathway for the arene amination process.

^{1}H NMR spectra for styrenyl substrate 12

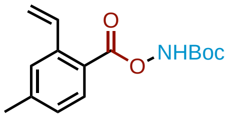

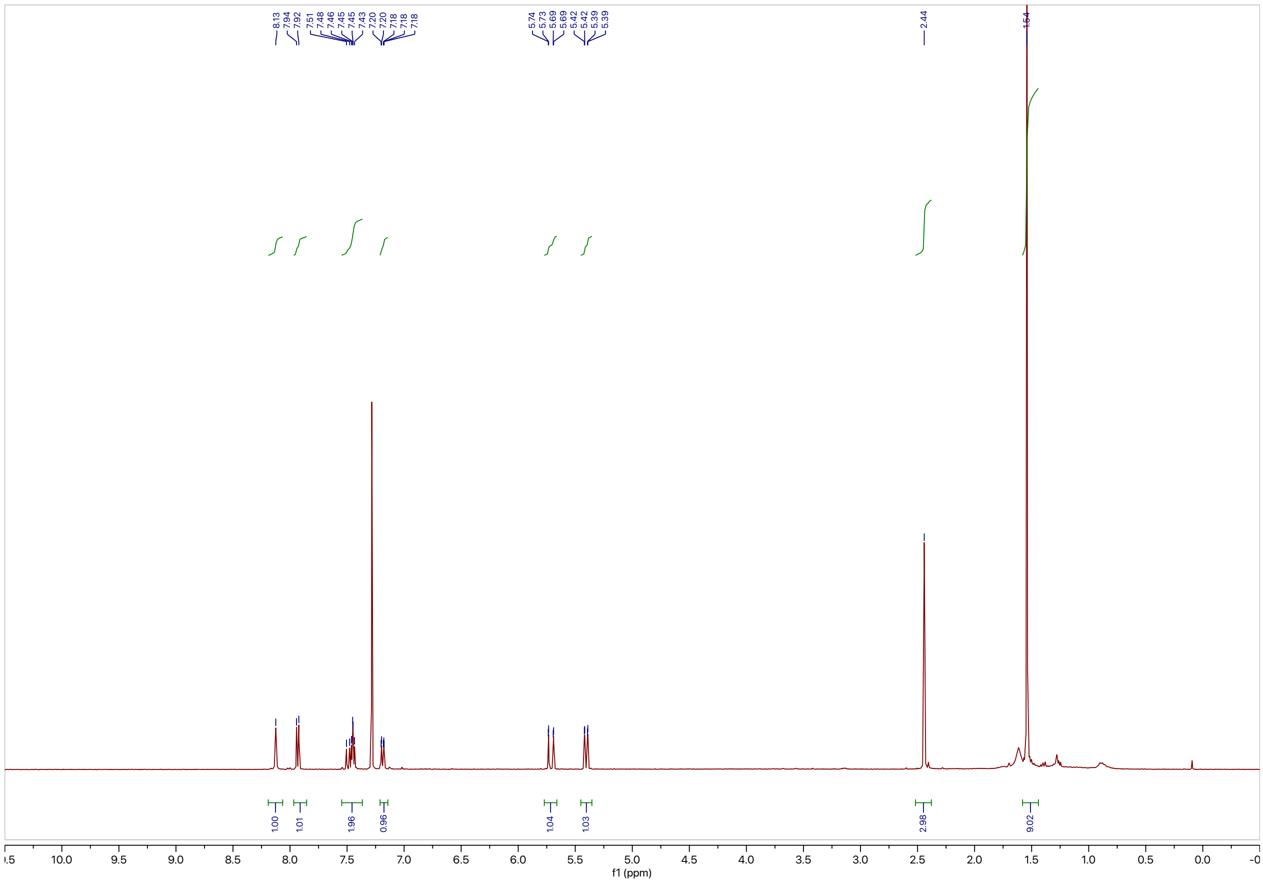

^{13}C NMR spectra for styrenyl substrate 12

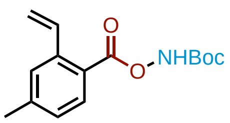

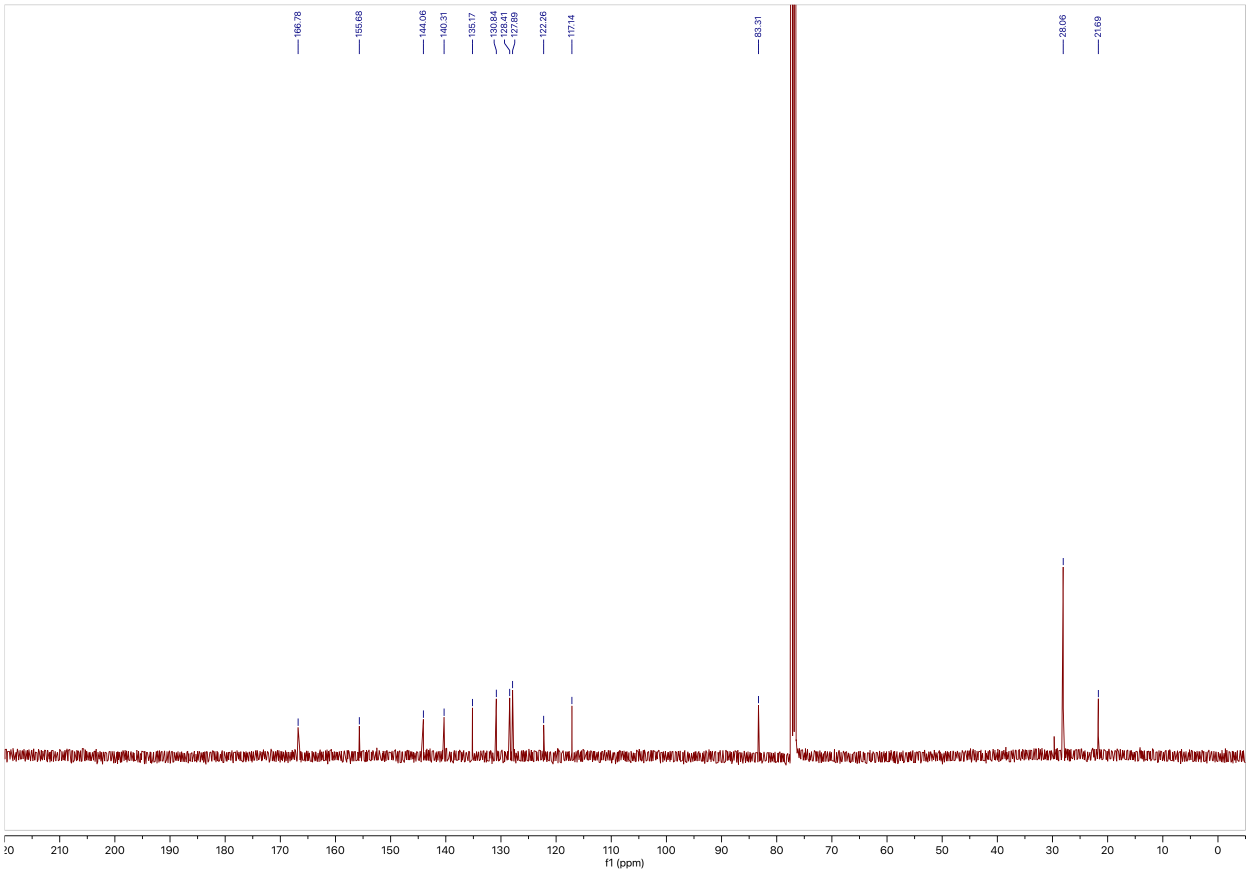

^{1}H NMR spectra for aminolactone 13

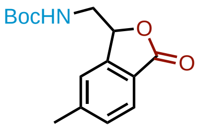

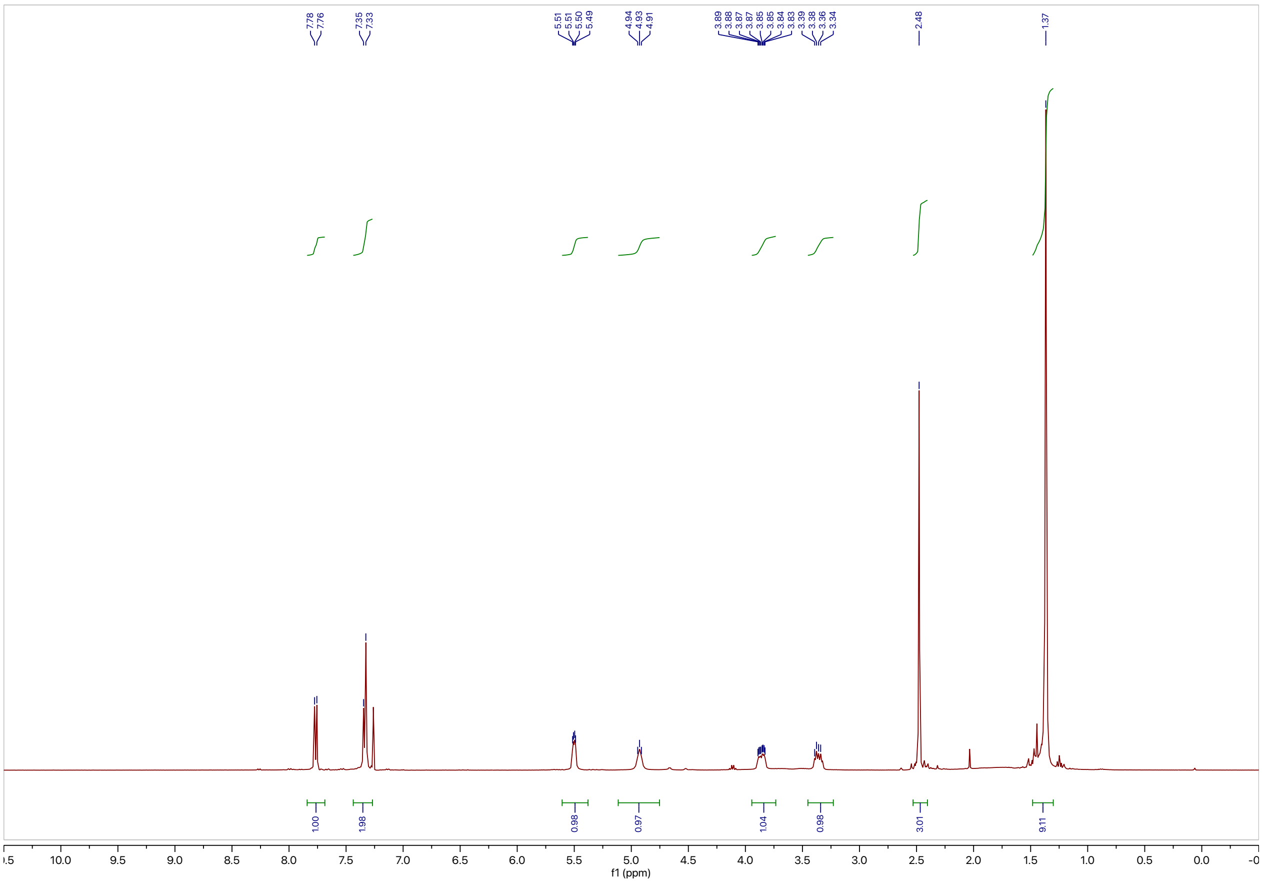

In addition to the crude NMR (*vide supra*), the diagnostic disappearance of alkene protons, in conjunction with no changes in the aromatic protons indicate that changes must have occurred on the alkene portion. Taken in conjunction with the appearance of diastereotopic protons (3.86, 3.36) indicate a new stereocentre formed. Accounting for a new further downfield NMR signal indicates a methine proton next to an ester allowed the gross assignment of the structure. The corresponding ^{13}C spectra below corroborates the disappearance of alkene protons, as well as indicating that there are two α-oxygen carbons (confirming lactonisation), and a new signal corresponding to a new methylene signal. These all spectroscopically confirm the formation of the aminolactone product.

^{13}C NMR spectra for aminolactone 13

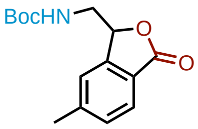

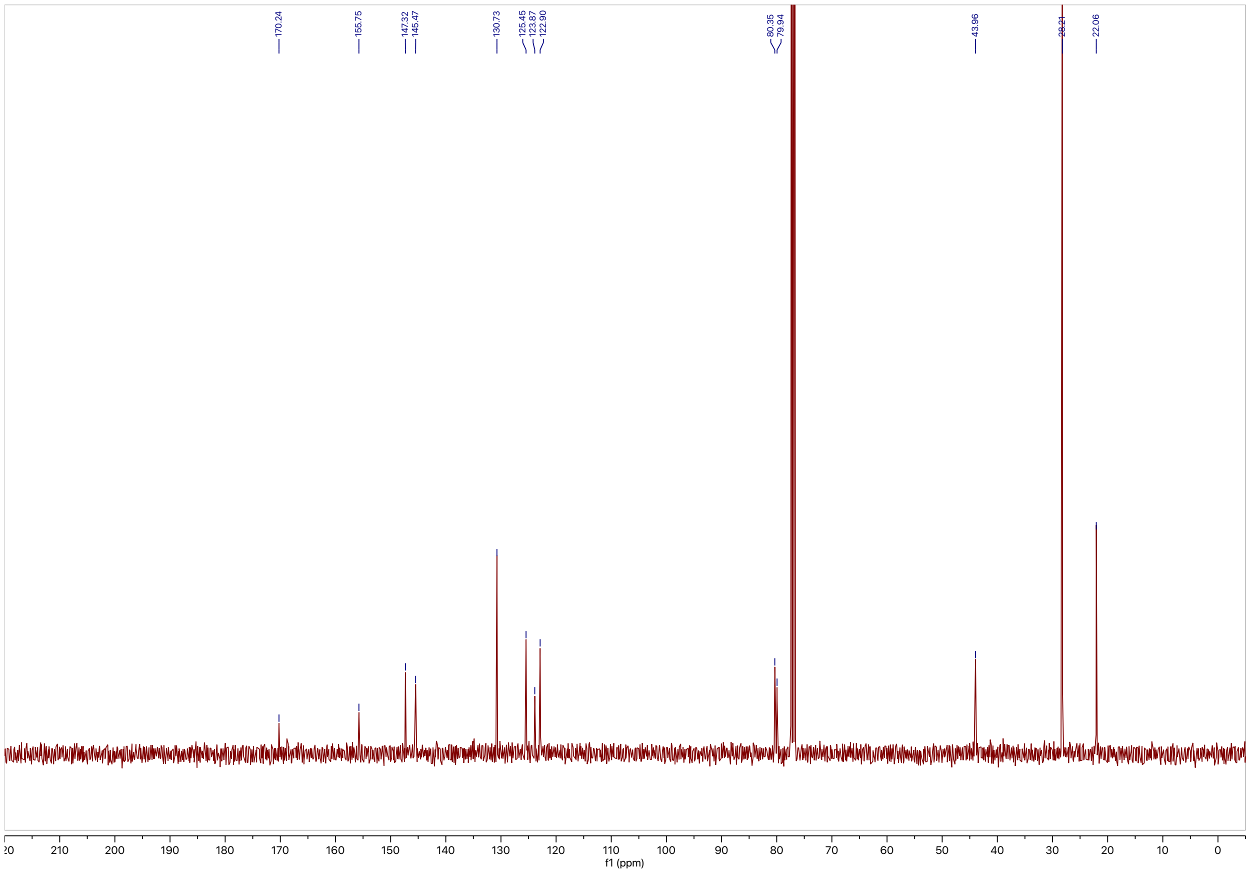

10 References

[1] P. Patel, S. Chang, *Org. Lett.* **2014**, *16*, 3328–3331.

[2] J. E. Gillespie, C. Morrill, R. J. Phipps, *J. Am. Chem. Soc.* **2021**, *143*, 9355–9360.

[3] P. Mondal, S. Rajapakse, G. B. Wijeratne, *J. Am. Chem. Soc.* **2022**, *144*, 3843–3854.

[4] B. Li, Y. Park, S. Chang, *J. Am. Chem. Soc.* **2014**, *136*, 1125–1131.

[5] F. Juliá, J. Yan, F. Paulus, T. Ritter, *J. Am. Chem. Soc.* **2021**, *143*, 12992–12998.

[6] G. Zhao, L. Liang, E. Wang, S. Lou, R. Qi, R. Tong, *Green Chem.* **2021**, *23*, 2300–2307.

[7] K. Holzschneider, F. Mohr, S. F. Kirsch, *Org. Lett.* **2018**, *20*, 7066–7070.

[8] W. Sun, C. H. Ling, C. M. Au, W. Y. Yu, *Org. Lett.* **2021**, *23*, 3310–3314.

[9] K. Michigami, H. Murakami, T. Nakamura, N. Hayama, Y. Takemoto, *Org. Biomol. Chem.* **2019**, *17*, 2331–2335.

[10] C. Liu, S. Han, M. Li, X. Chong, B. Zhang, *Angew. Chem. Int. Ed.* **2020**, *59*, 18527–18531.

[11] L. Jin, X. L. Zhang, R. L. Guo, M. Y. Wang, Y. R. Gao, Y. Q. Wang, *Org. Lett.* **2021**, *23*, 1921–1927.

[12] E. M. D’Amato, J. Börgel, T. Ritter, *Chem. Sci.* **2019**, *10*, 2424–2428.

[13] S. Lee, J. Sim, H. Jo, M. Viji, L. Srinu, K. Lee, H. Lee, V. Manjunatha, J. K. Jung, *Org. Biomol. Chem.* **2019**, *17*, 8067–8070.

[14] Z. Yin, Z. Wang, X. F. Wu, *Org. Lett.* **2017**, *19*, 6232–6235.

11 NMR Spectra

Substrates

^{1}H NMR spectrum of *tert*-butyl (benzoyloxy)carbamate **1a** in $CDCl_3$

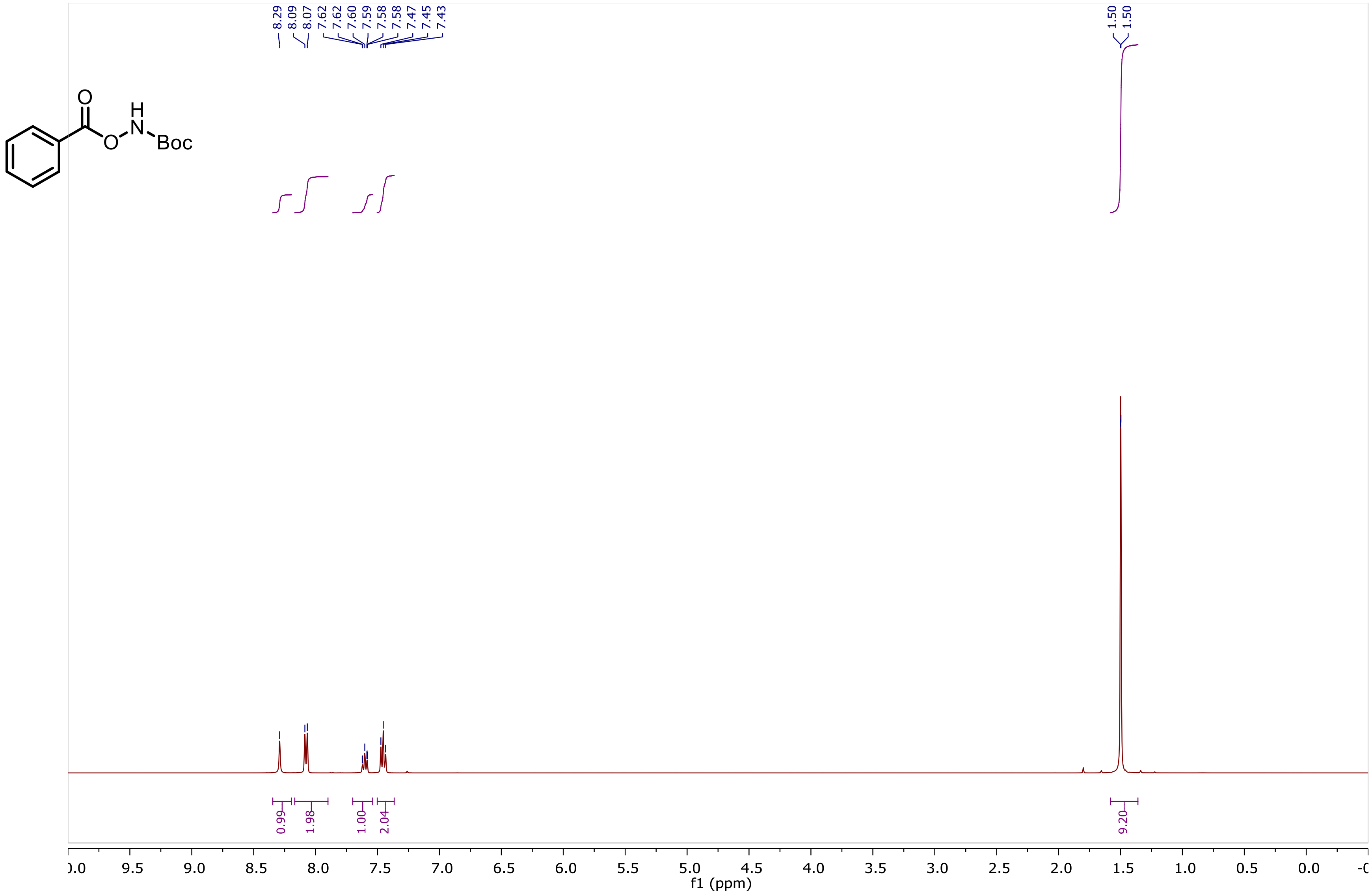

^{13}C NMR spectrum of *tert*-butyl (benzoyloxy)carbamate **1a** in $CDCl_3$

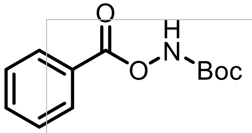

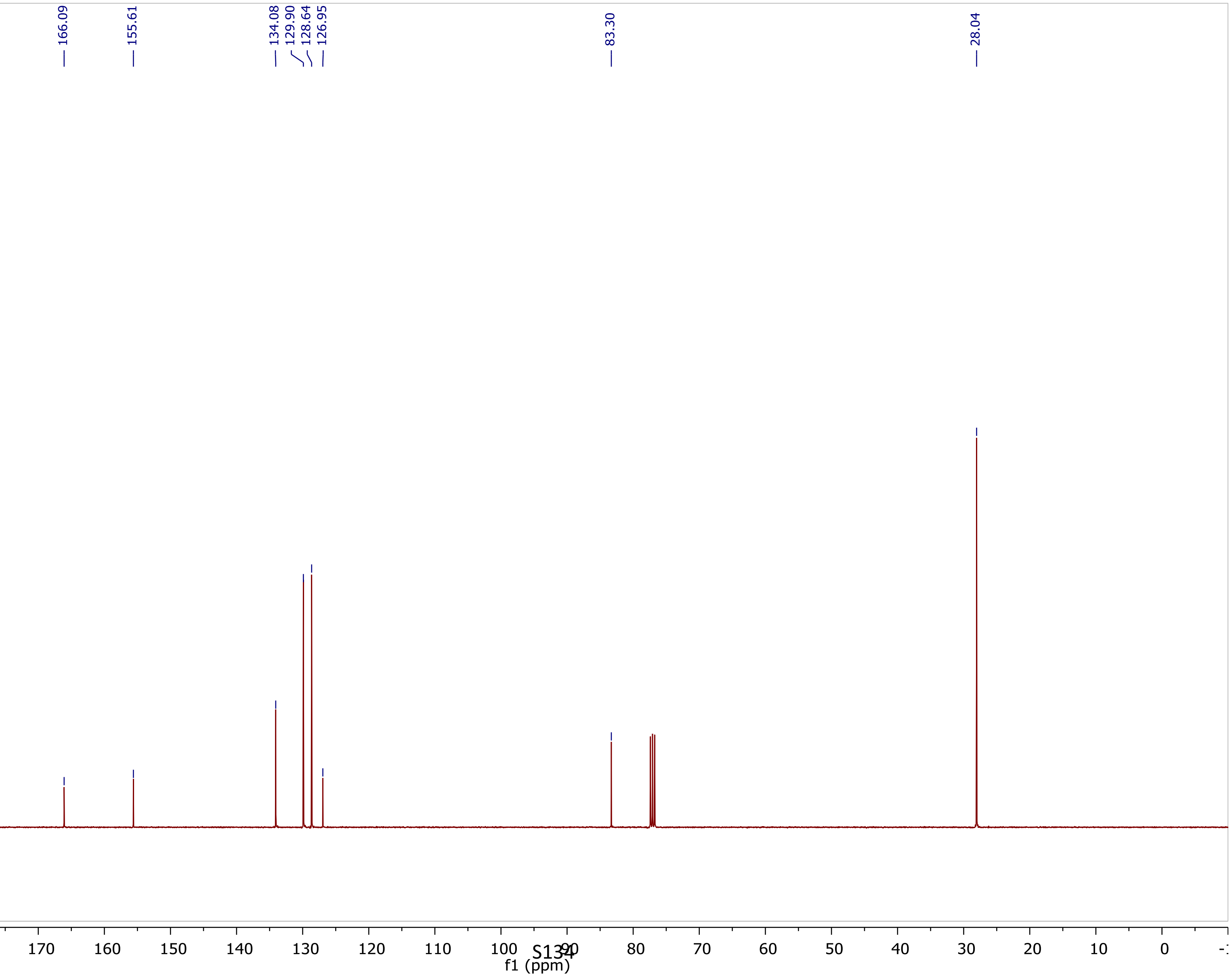

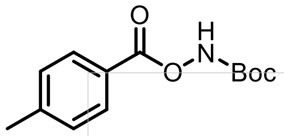

^{1}H NMR spectrum of *tert*-butyl (4-methylbenzoyloxy)carbamate **1b** in $CDCl_3$

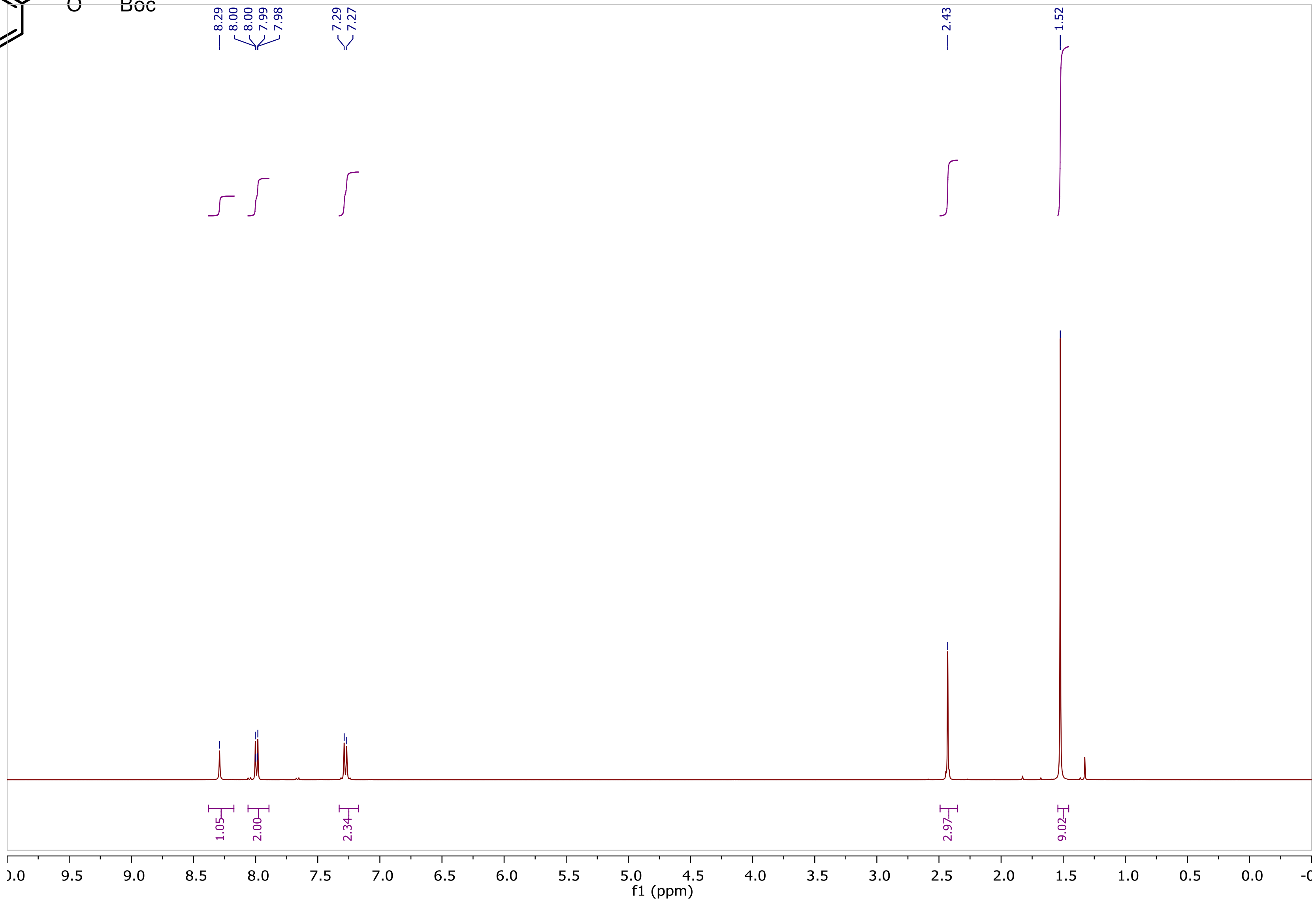

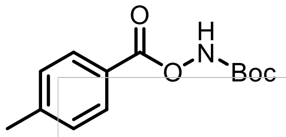

^{13}C NMR spectrum of *tert*-butyl (4-methylbenzoyloxy)carbamate **1b** in $CDCl_3$

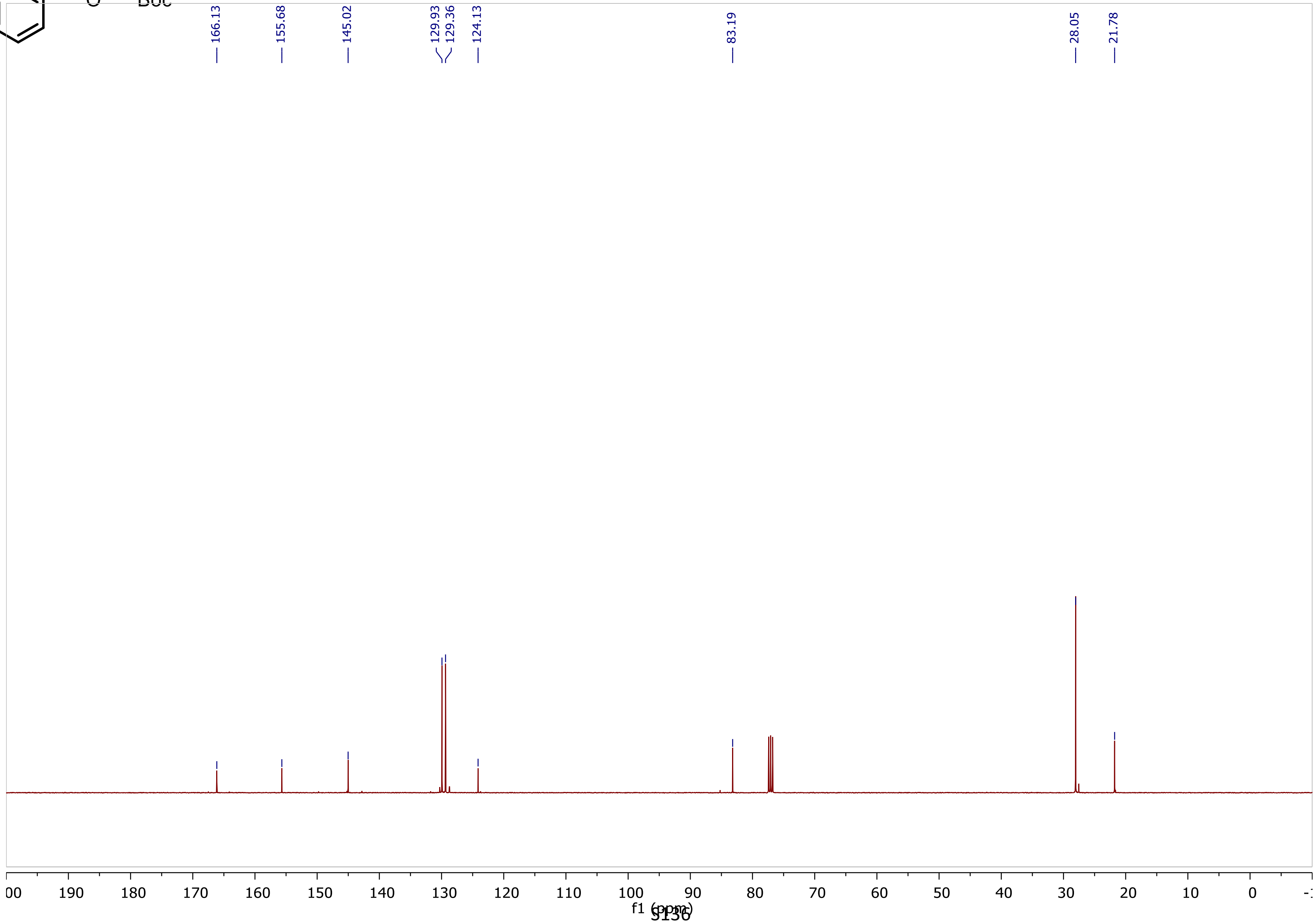

^{1}H NMR spectrum of *tert*-butyl (4-propylbenzoyloxy)carbamate **1c** in $CDCl_3$

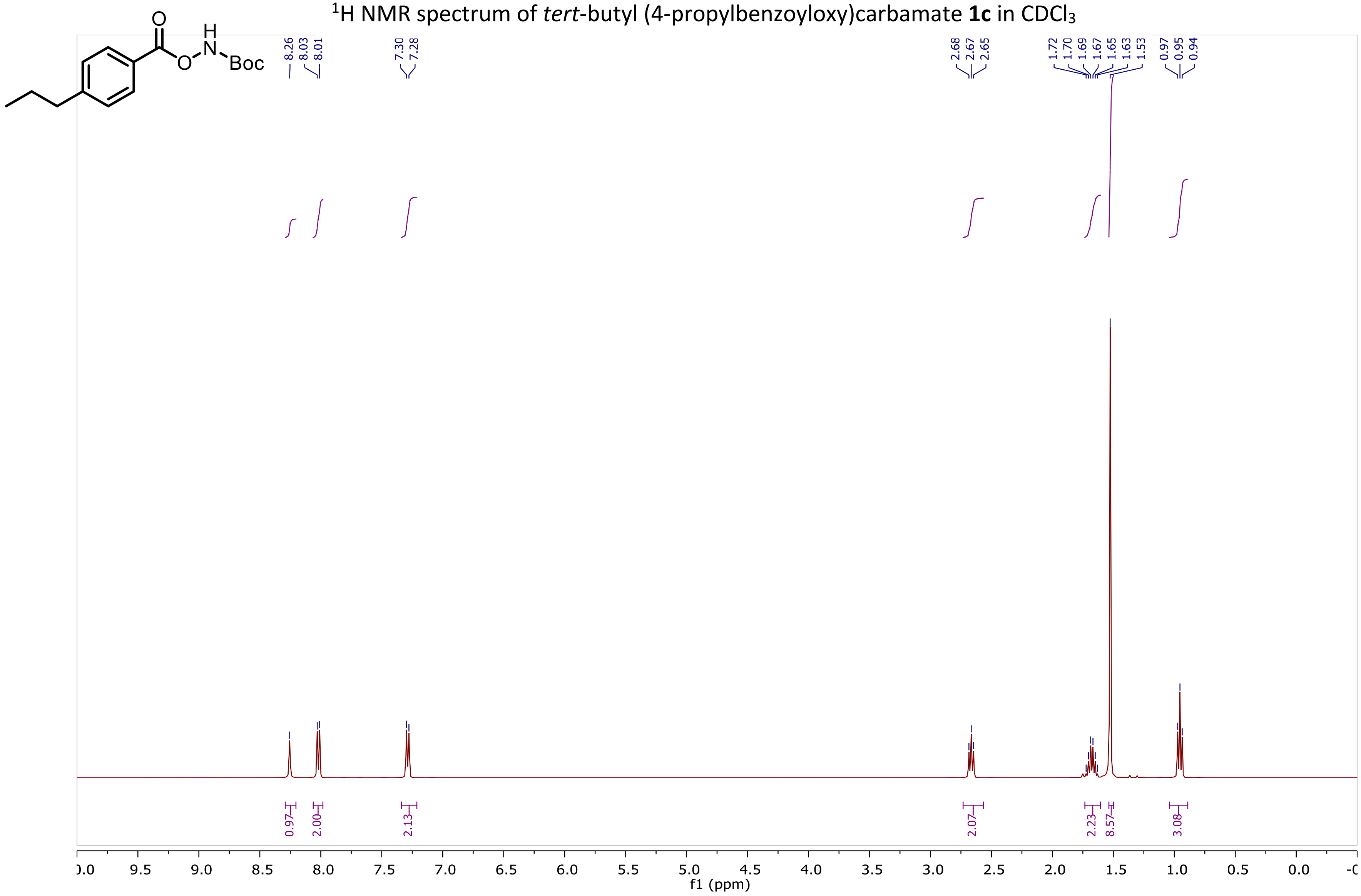

^{13}C NMR spectrum of *tert*-butyl (4-propylbenzoyloxy)carbamate **1c** in $CDCl_3$

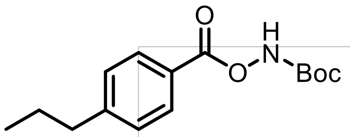

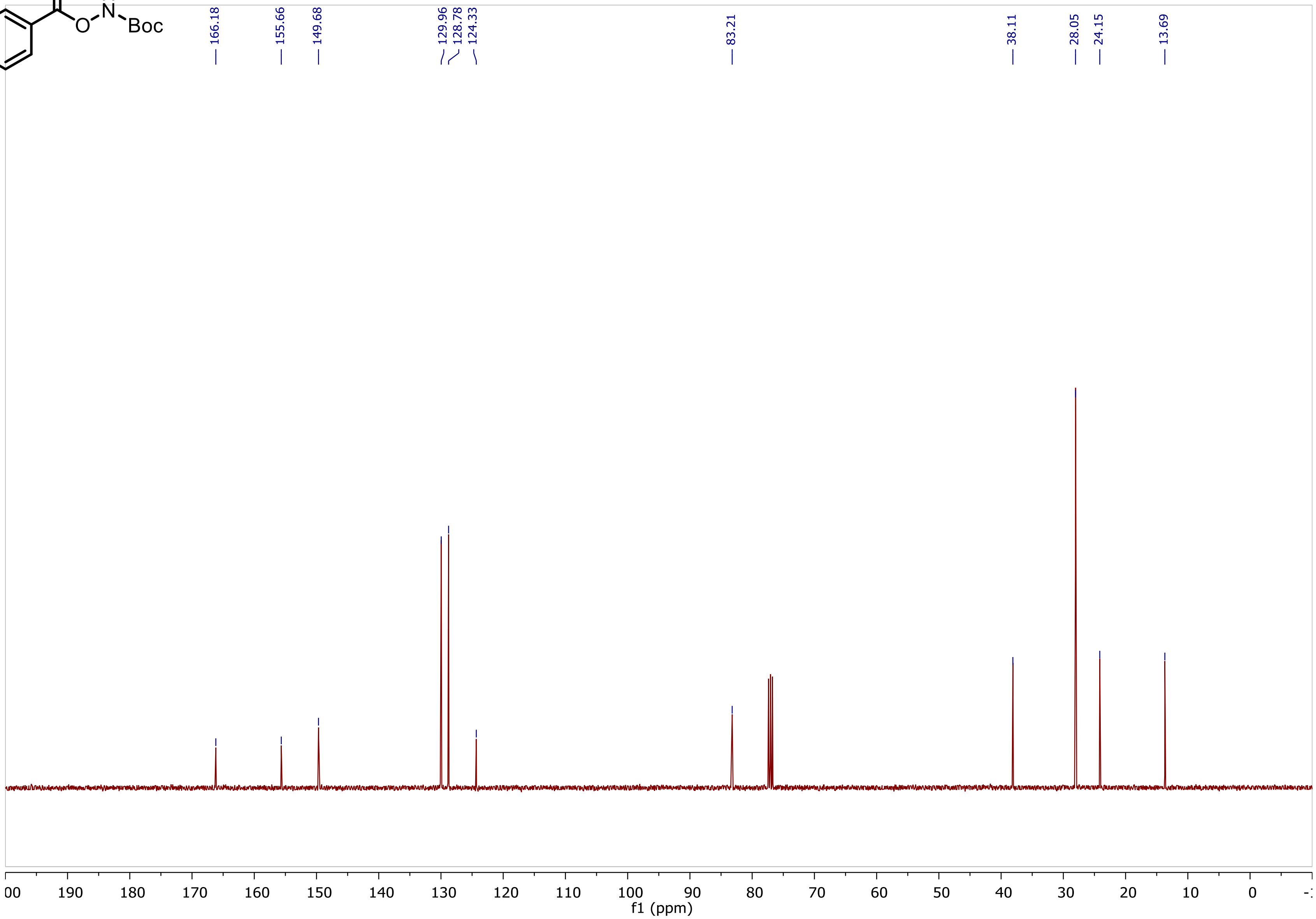

^{1}H NMR spectrum of *tert*-butyl (4-isopropylbenzoyloxy)carbamate **1d** in $CDCl_3$

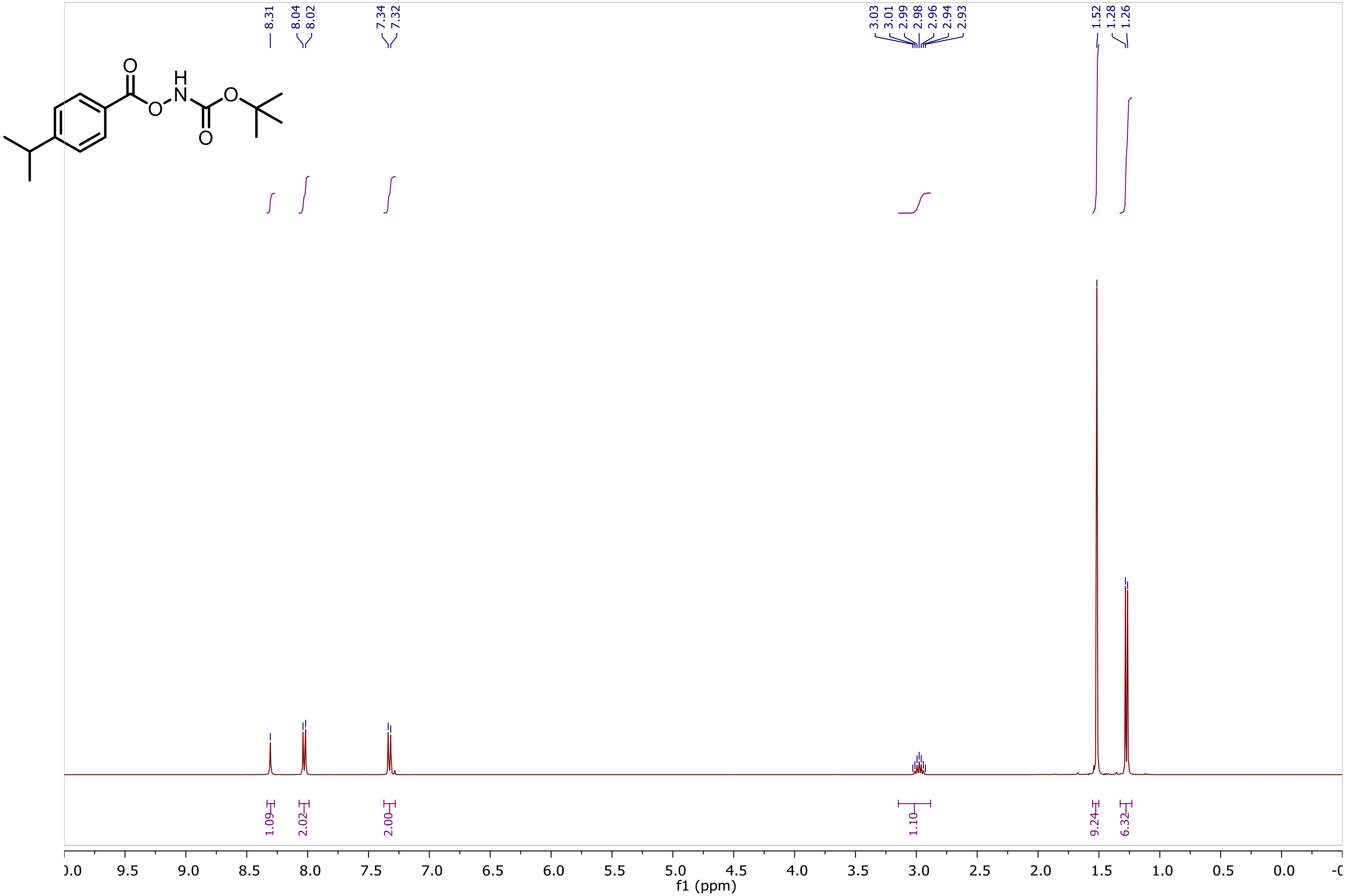

^{13}C NMR spectrum of *tert*-butyl (4-isopropylbenzoyloxy)carbamate **1d** in $CDCl_3$

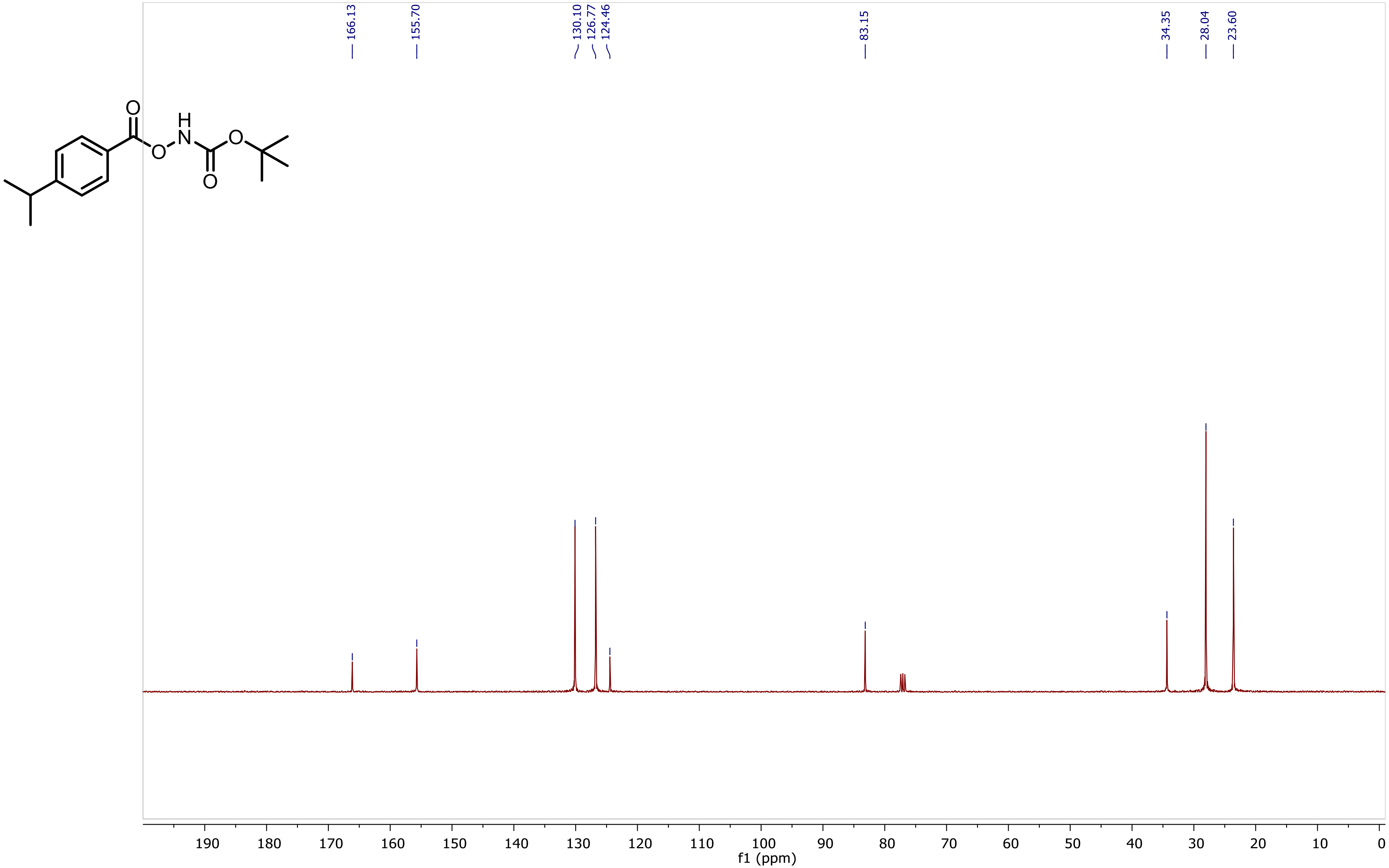

^{1}H NMR spectrum of *tert*-butyl (3-methylbenzoyloxy)carbamate **1e** in $CDCl_3$

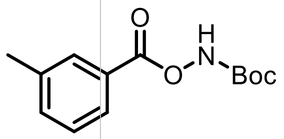

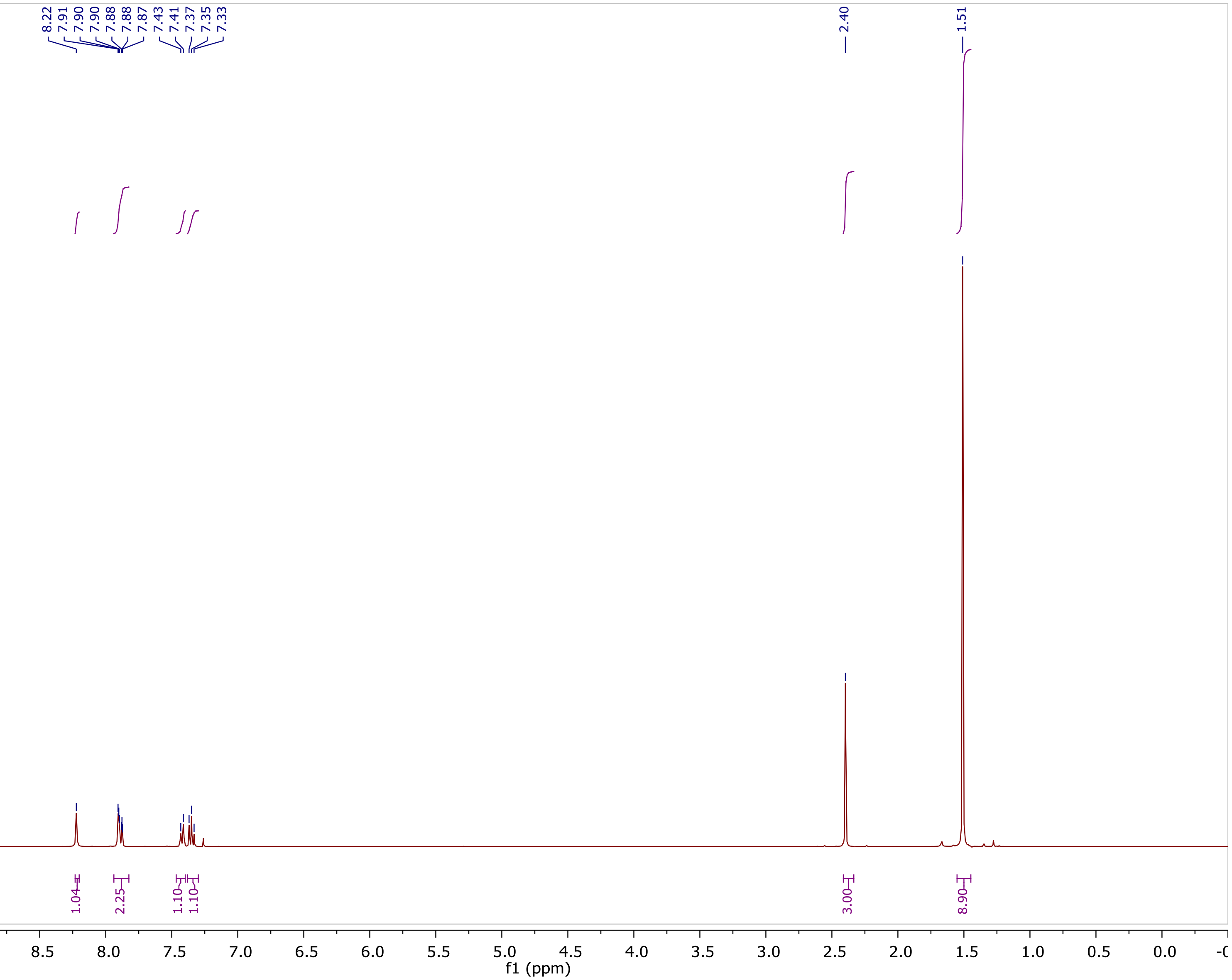

^{13}C NMR spectrum of *tert*-butyl (3-methylbenzoyloxy)carbamate **1e** in $CDCl_3$

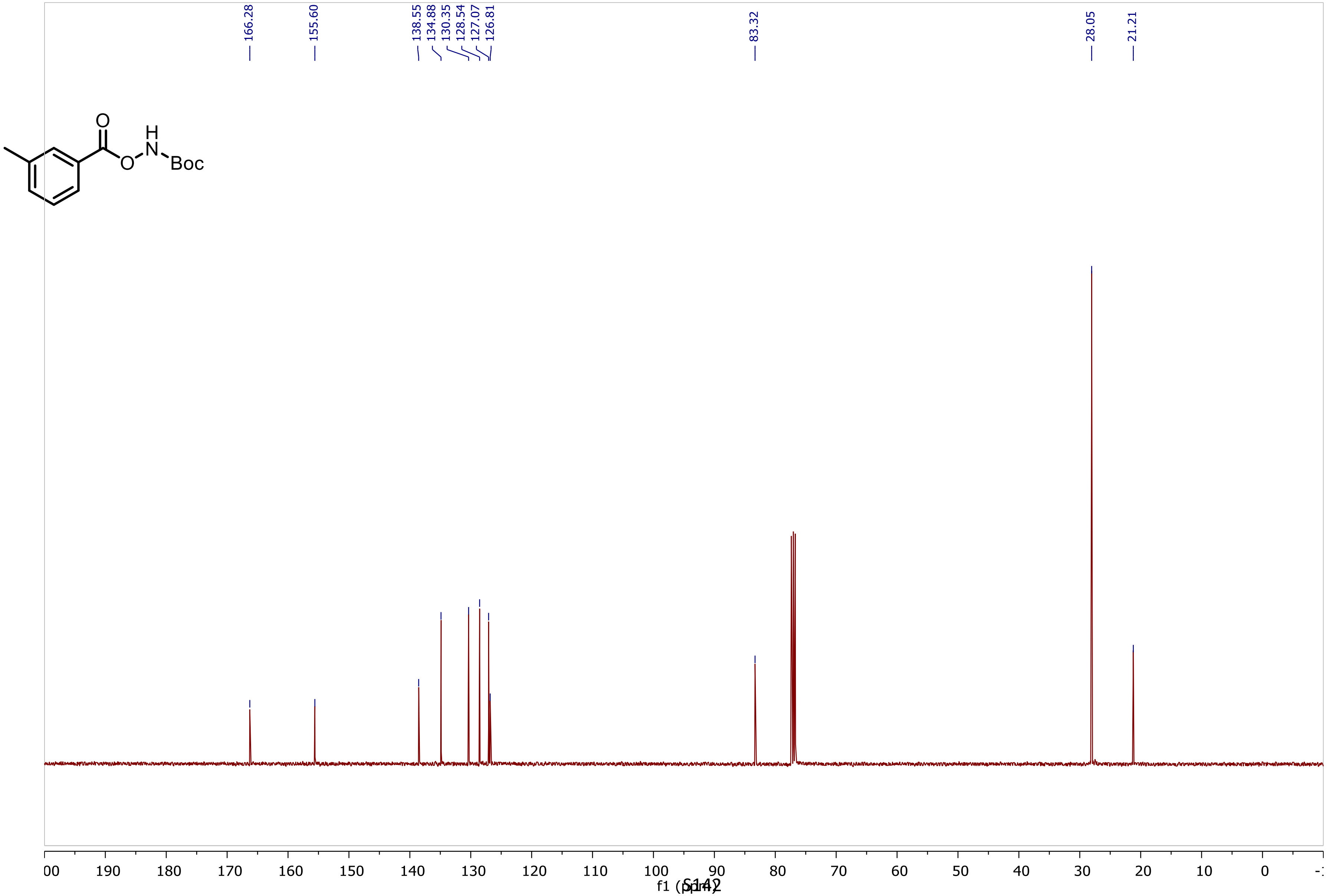

^{1}H NMR spectrum of *tert*-butyl (3,5-dimethylbenzoyloxy)carbamate **1f** in $CDCl_3$

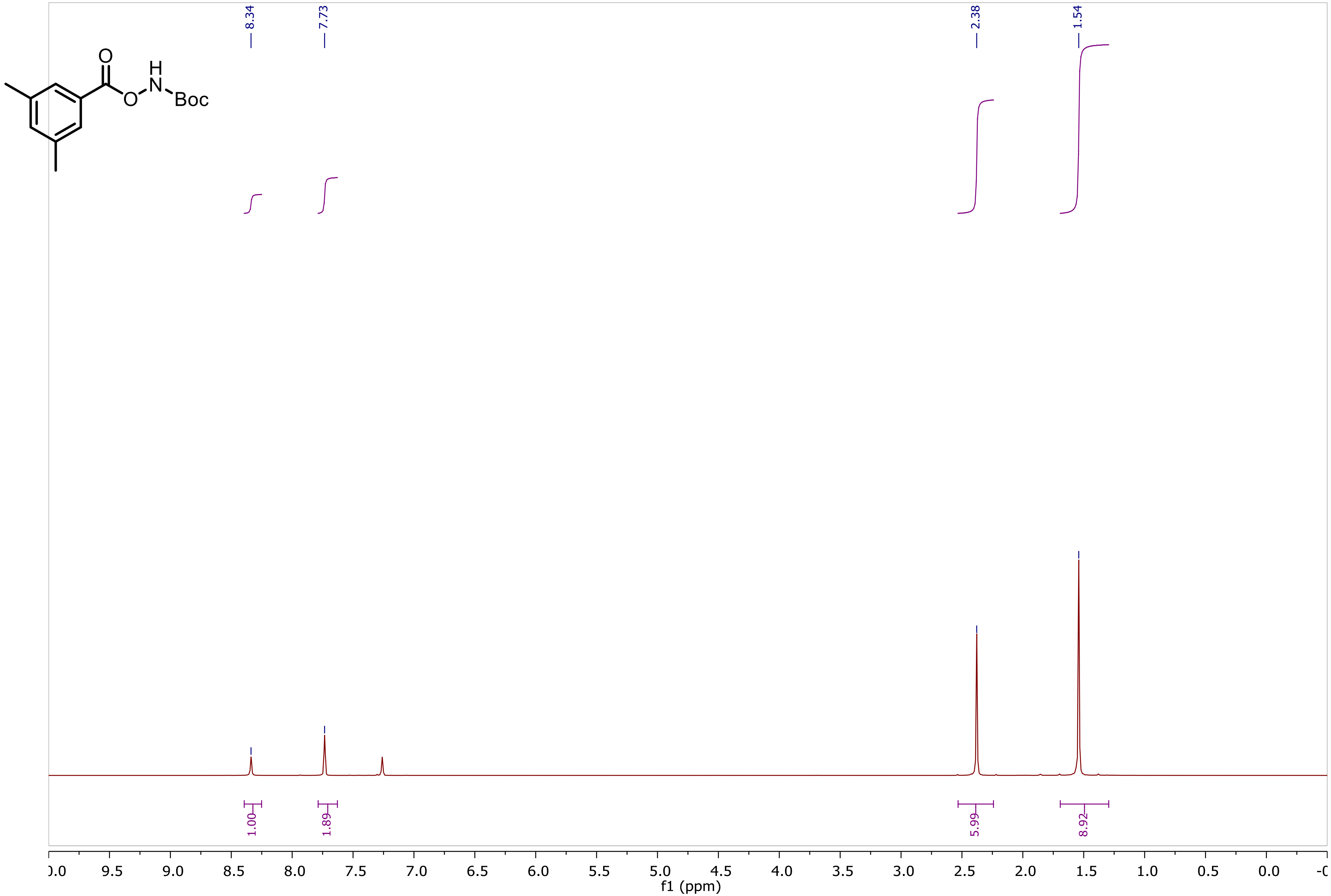

^{13}C NMR spectrum of *tert*-butyl (3,5-dimethylbenzoyloxy)carbamate **1f** in $CDCl_3$

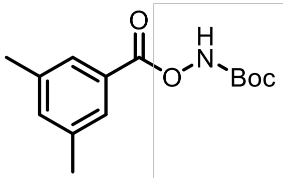

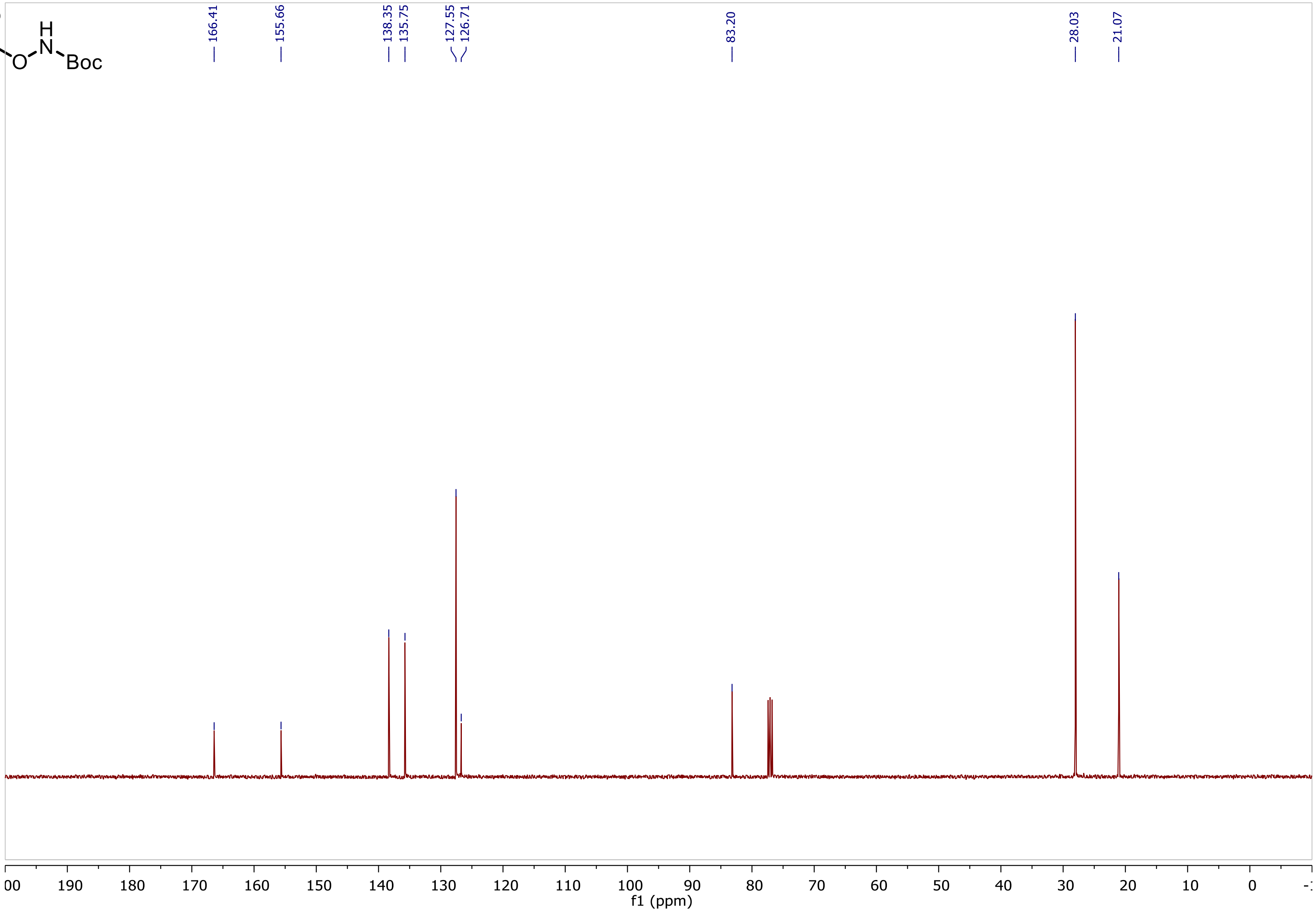

^{1}H NMR spectrum of *tert*-butyl (3-isopropylbenzoyloxy)carbamate **1g** in $CDCl_3$

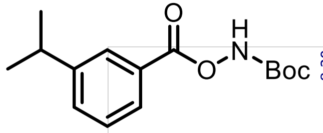

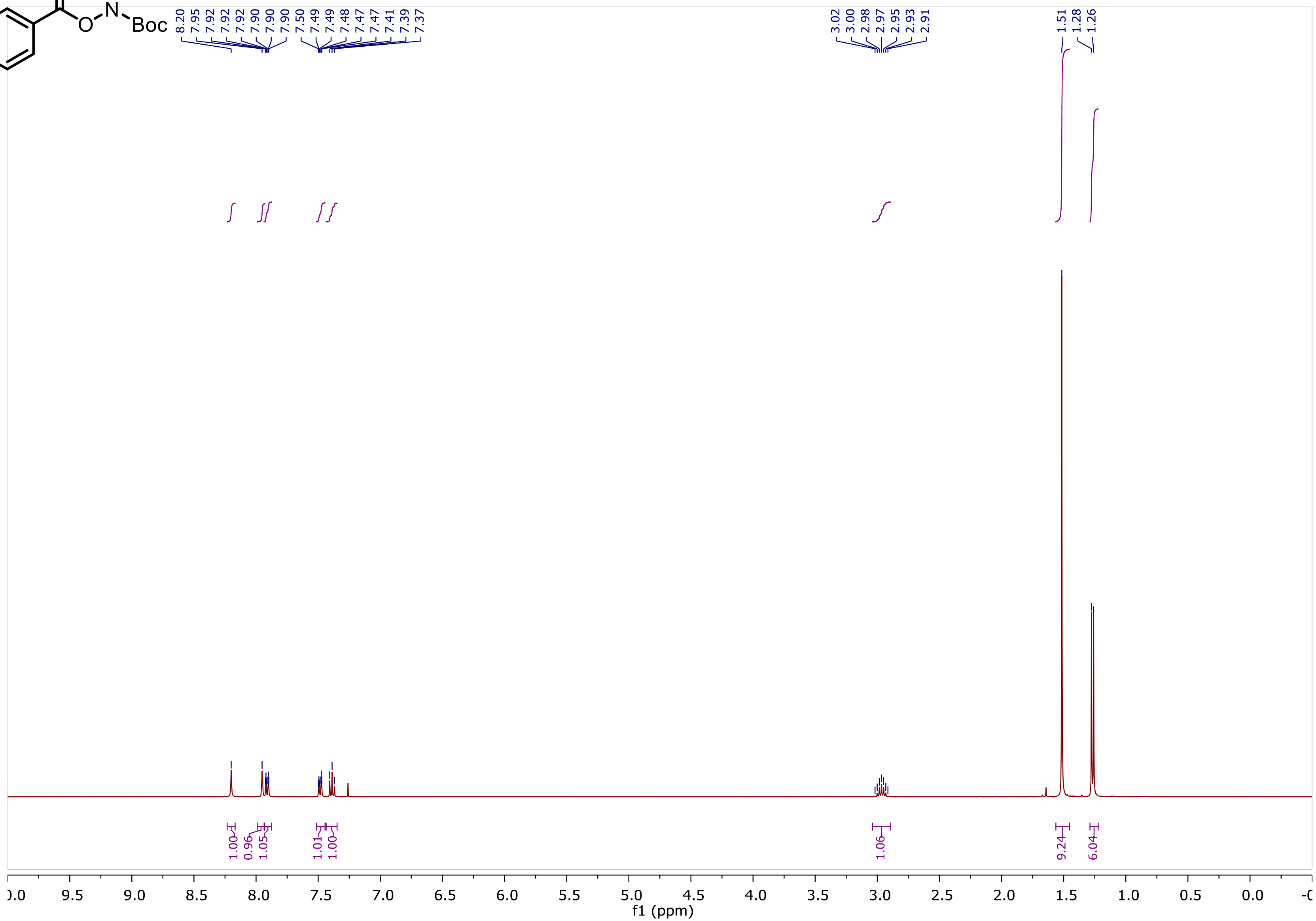

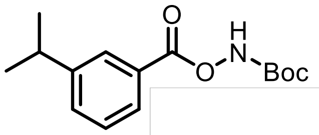

^{13}C NMR spectrum of *tert*-butyl (3-isopropylbenzoyloxy)carbamate **1g** in $CDCl_3$

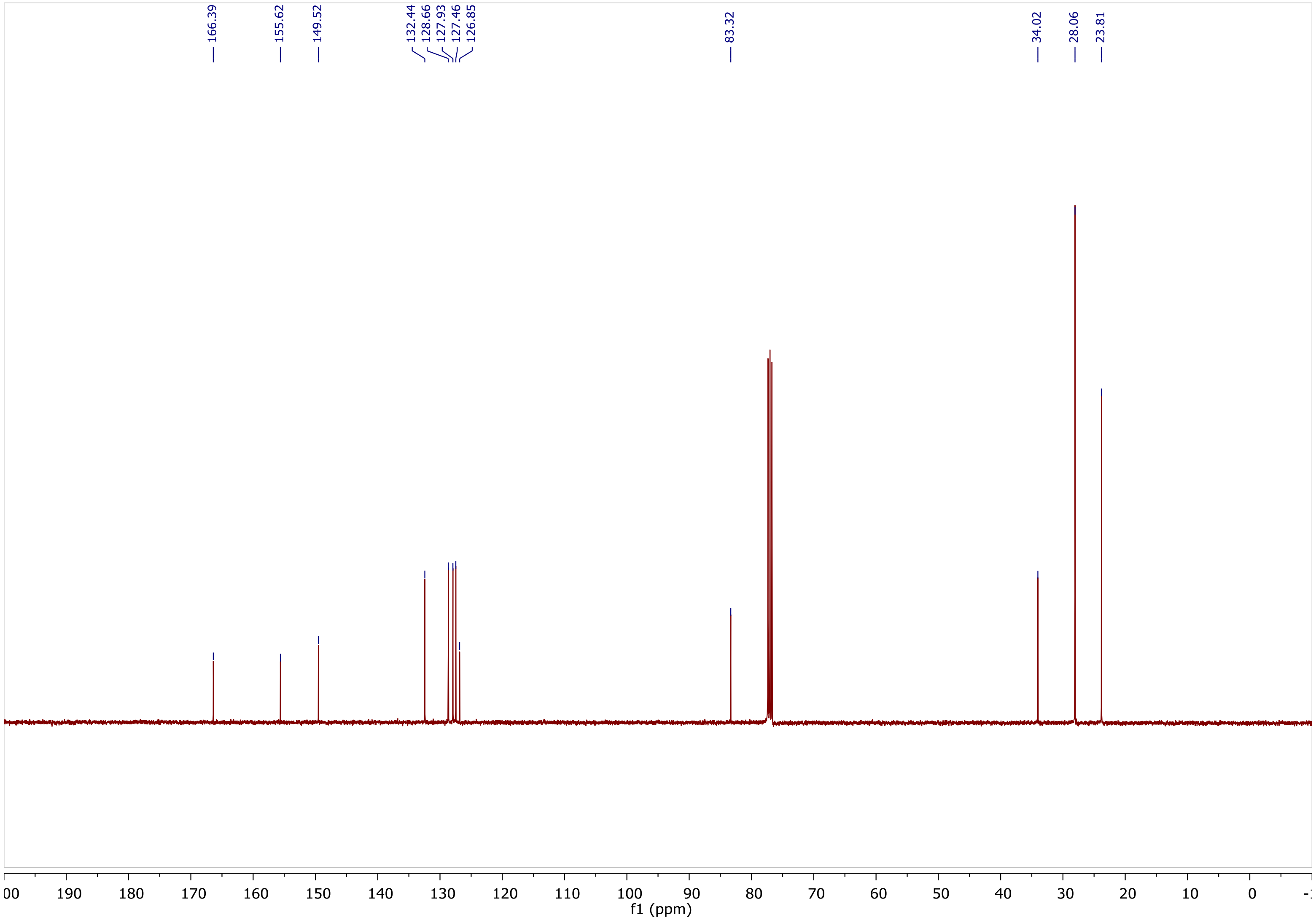

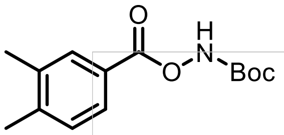

^{1}H NMR spectrum of *tert*-butyl (3,4-dimethylbenzoyloxy)carbamate **1h** in $CDCl_3$

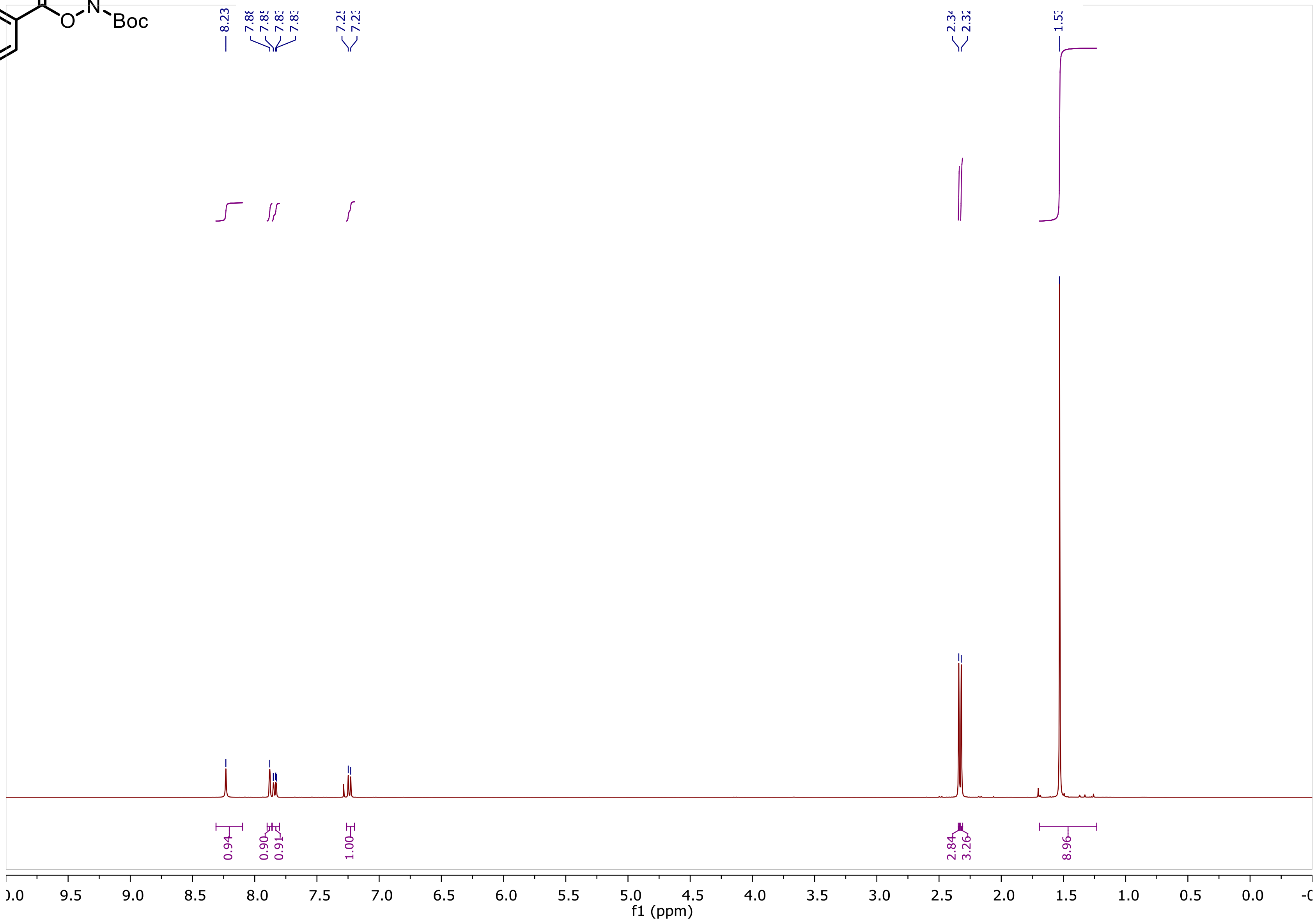

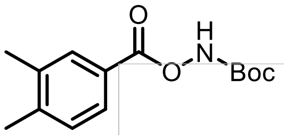

^{13}C NMR spectrum of *tert*-butyl (3,4-dimethylbenzoyloxy)carbamate **1h** in $CDCl_3$

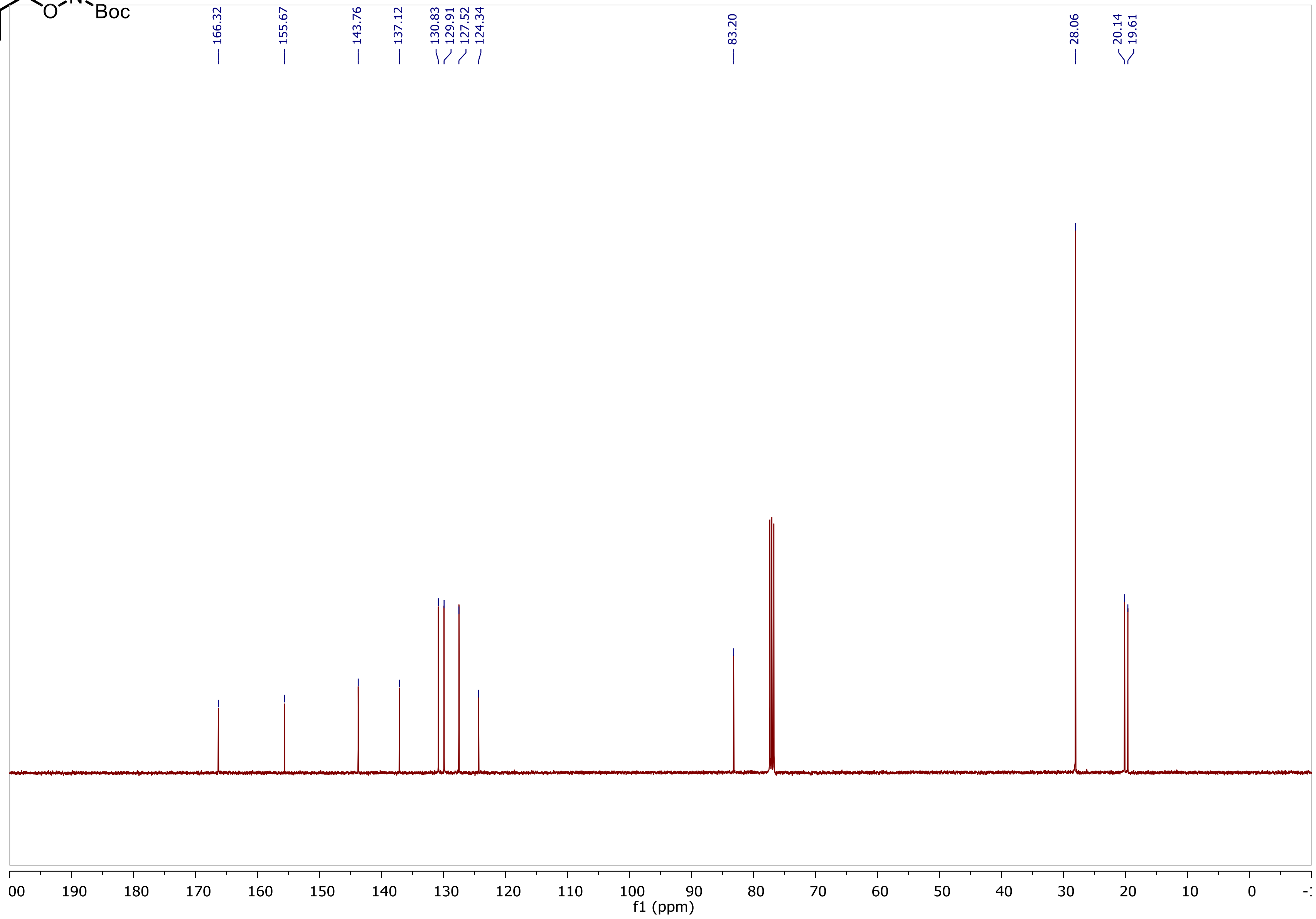

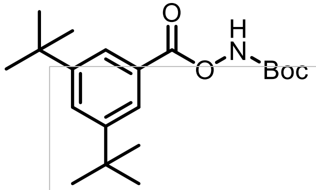

^{1}H NMR spectrum of *tert*-butyl (3,5-di-*tert*-butylbenzoyloxy)carbamate **1i** in $CDCl_3$

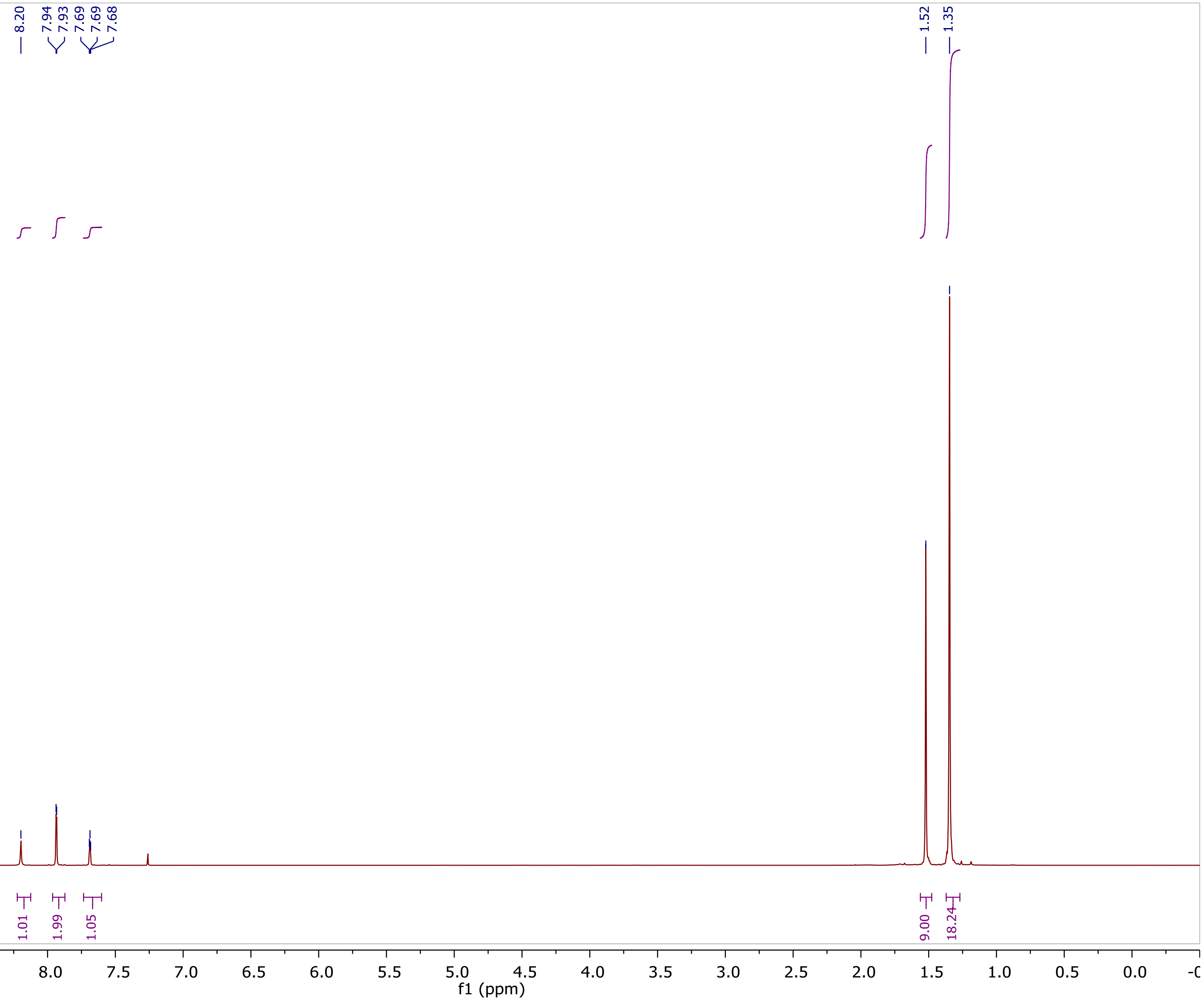

^{13}C NMR spectrum of *tert*-butyl (3,5-di-*tert*-butylbenzoyloxy)carbamate **1i** in $CDCl_3$

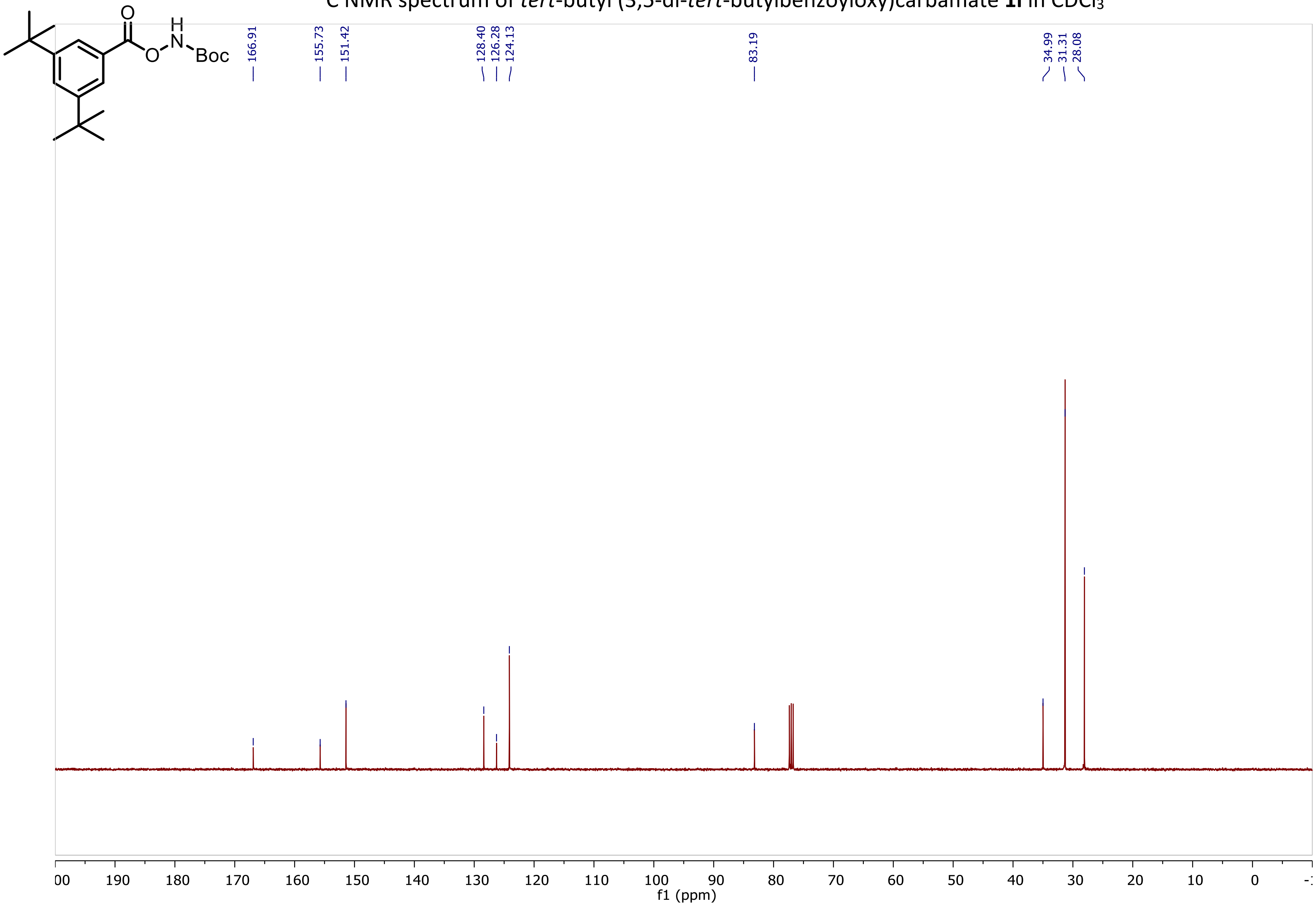

^{1}H NMR spectrum of *tert*-butyl (4-fluorobenzoyloxy)carbamate **1j** in $CDCl_3$

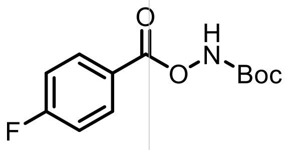

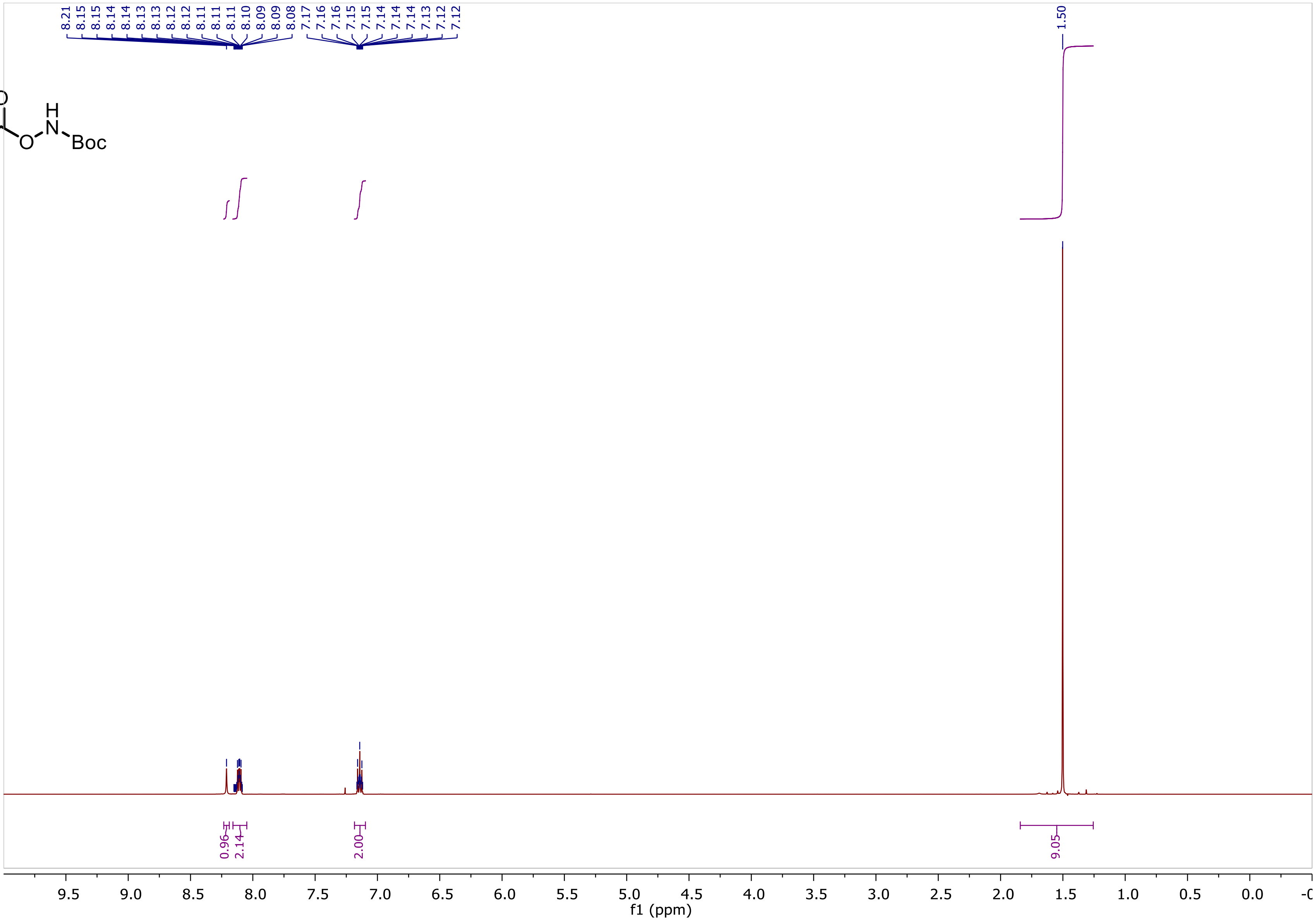

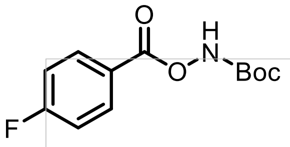

^{13}C NMR spectrum of *tert*-butyl (4-fluorobenzoyloxy)carbamate **1j** in $CDCl_3$

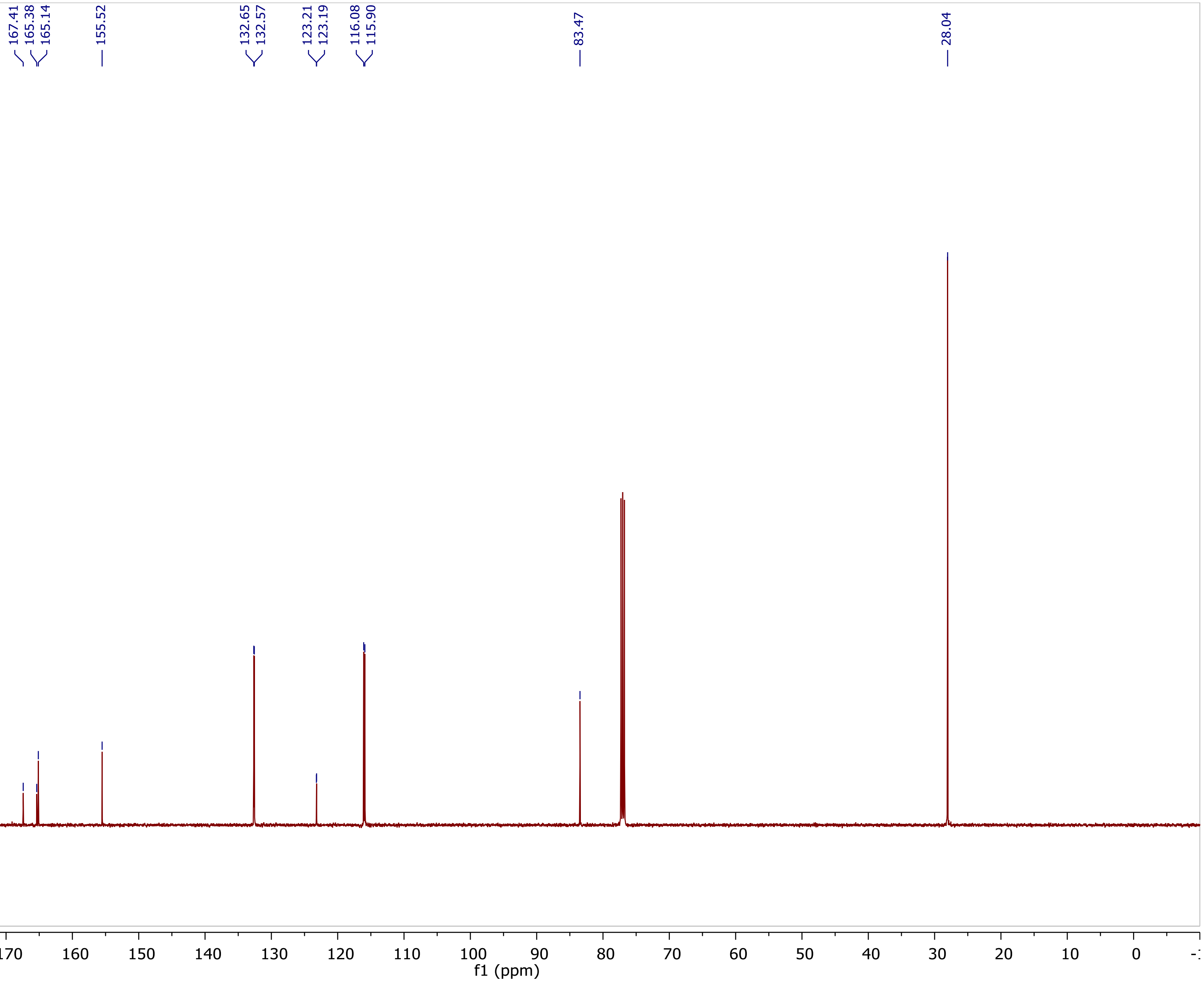

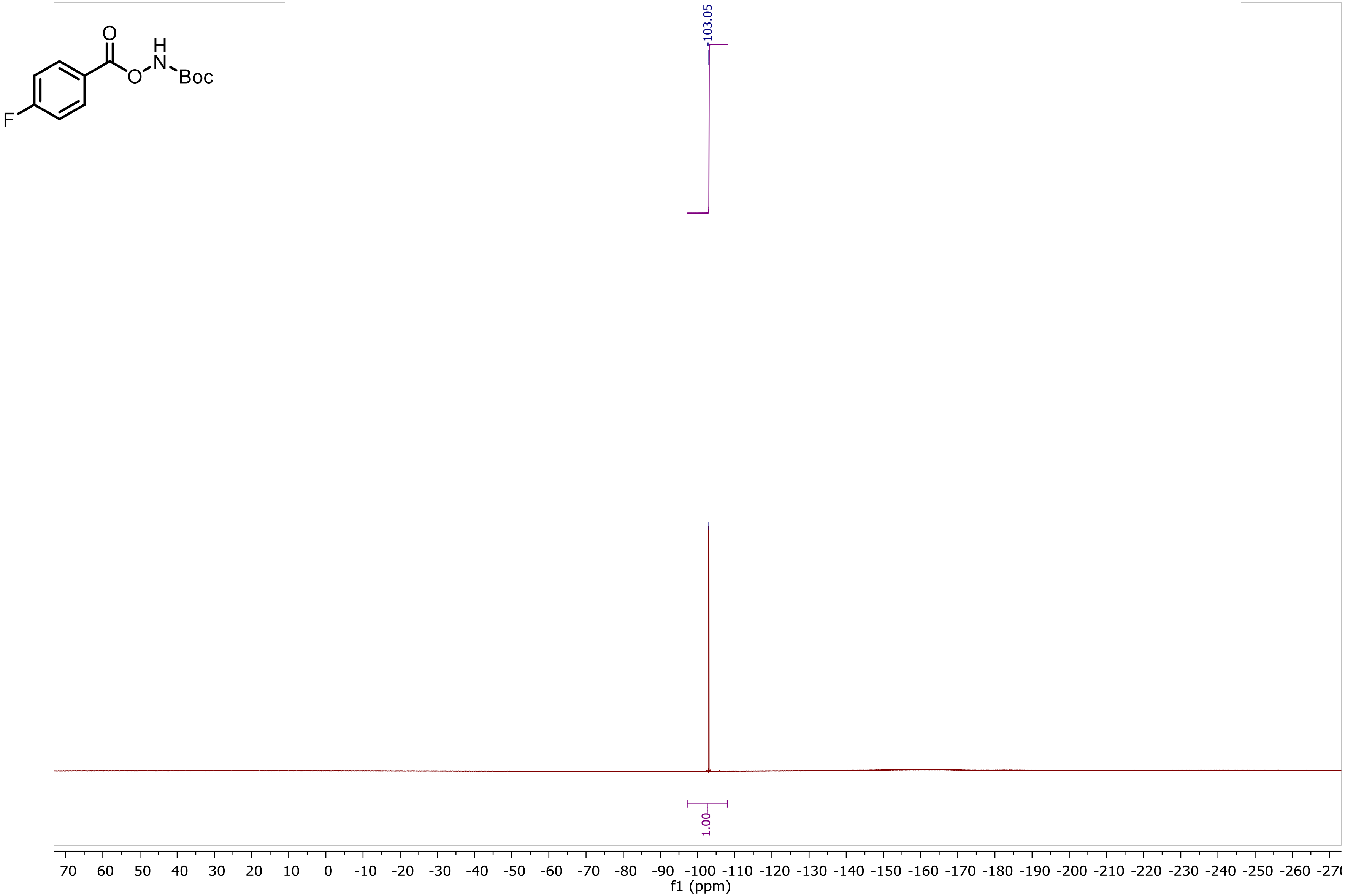

-103.05
1.00
70 60 50 40 30 20 10 0 -10 -20 -30 -40 -50 -60 -70 -80 -90 -100 -110 -120 -130 -140 -150 -160 -170 -180 -190 -200 -210 -220 -230 -240 -250 -260 -270
f1 (ppm)

^{1}H NMR spectrum of *tert*-butyl (4-chlorobenzoyloxy)carbamate **1m** in $CDCl_3$

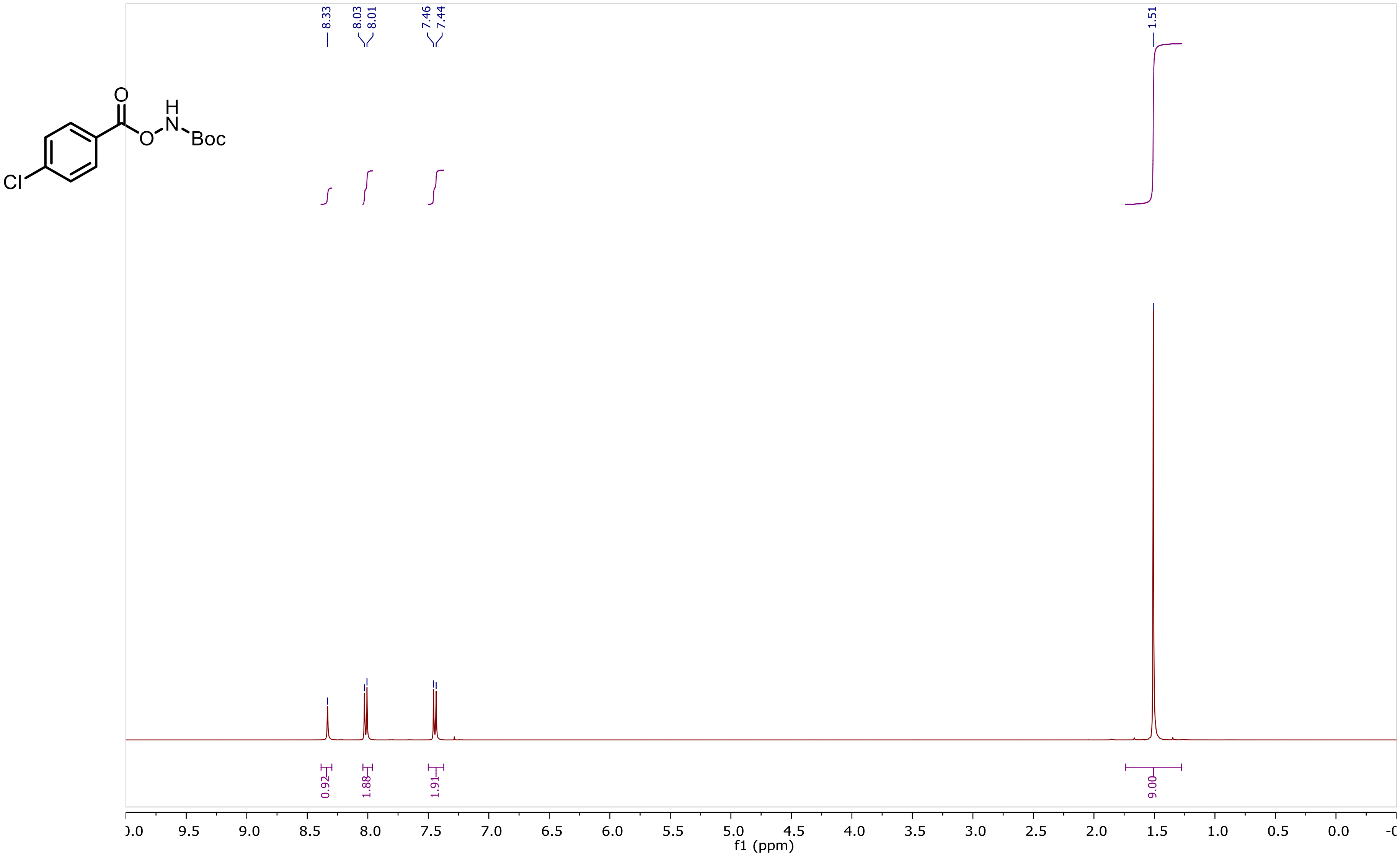

^{13}C NMR spectrum of *tert*-butyl (4-chlorobenzoyloxy)carbamate **1m** in $CDCl_3$

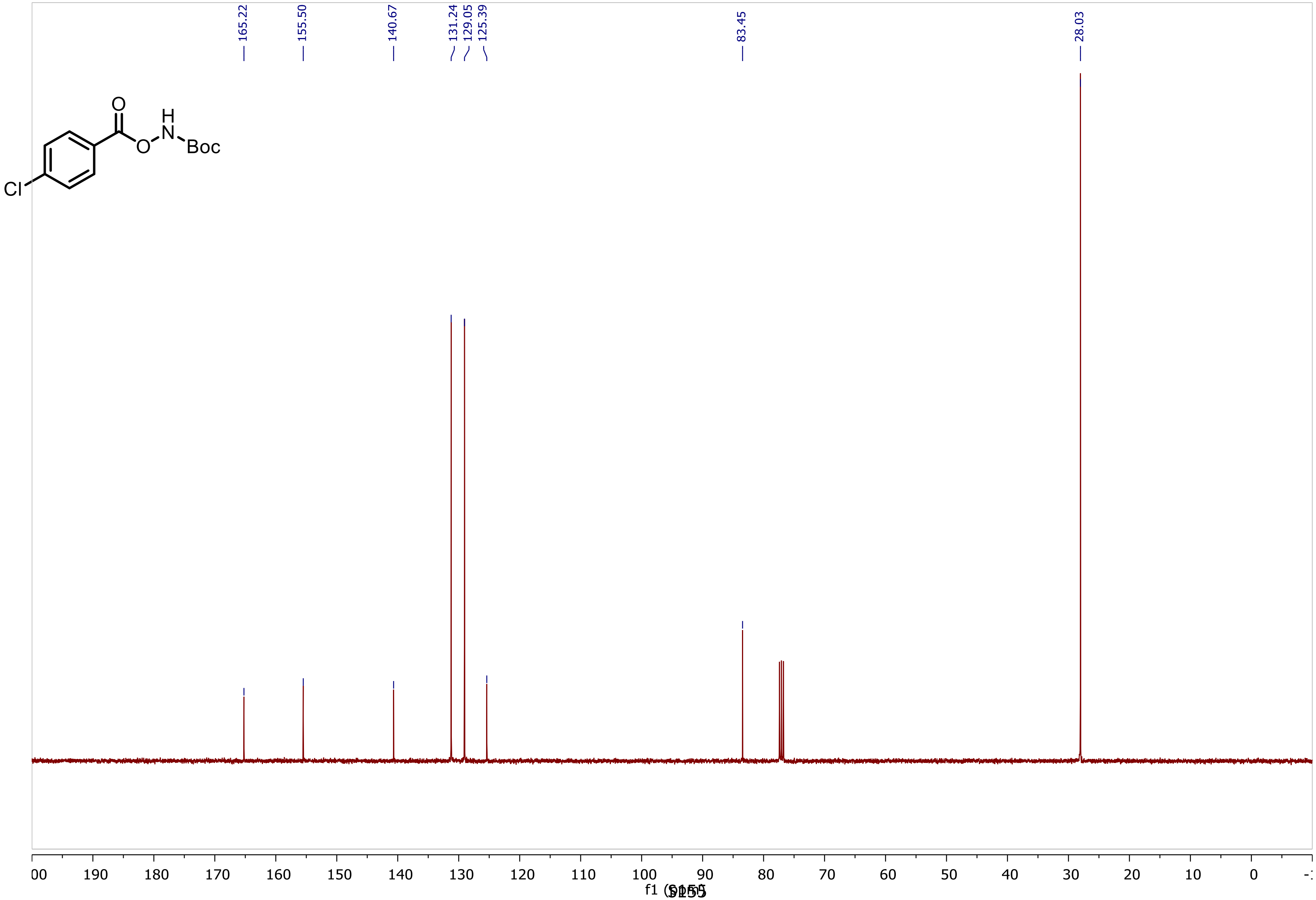

^{1}H NMR spectrum of *tert*-butyl (4-bromobenzoyloxy)carbamate **1r** in $CDCl_3$

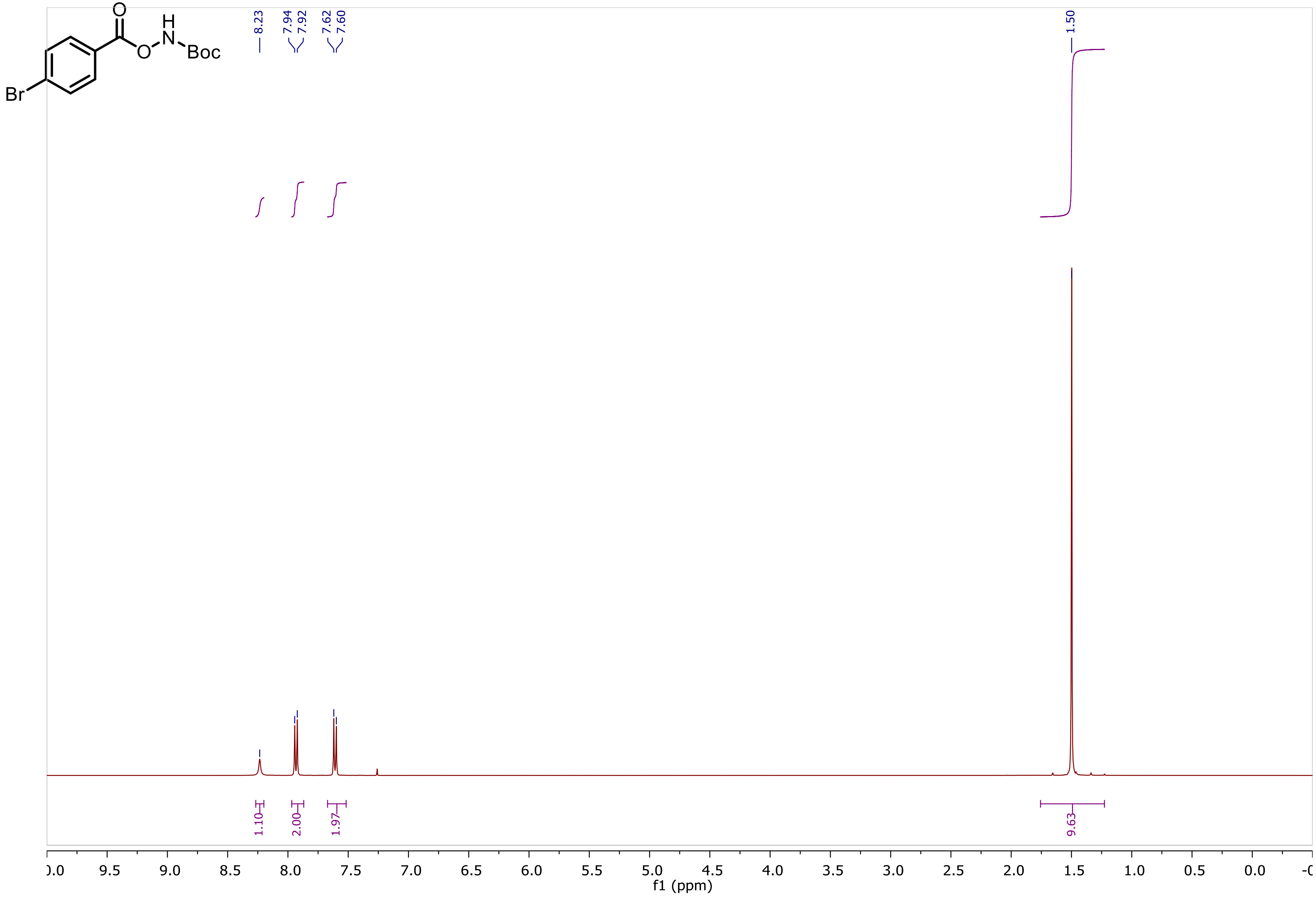

^{13}C NMR spectrum of *tert*-butyl (4-bromobenzoyloxy)carbamate **1r** in $CDCl_3$

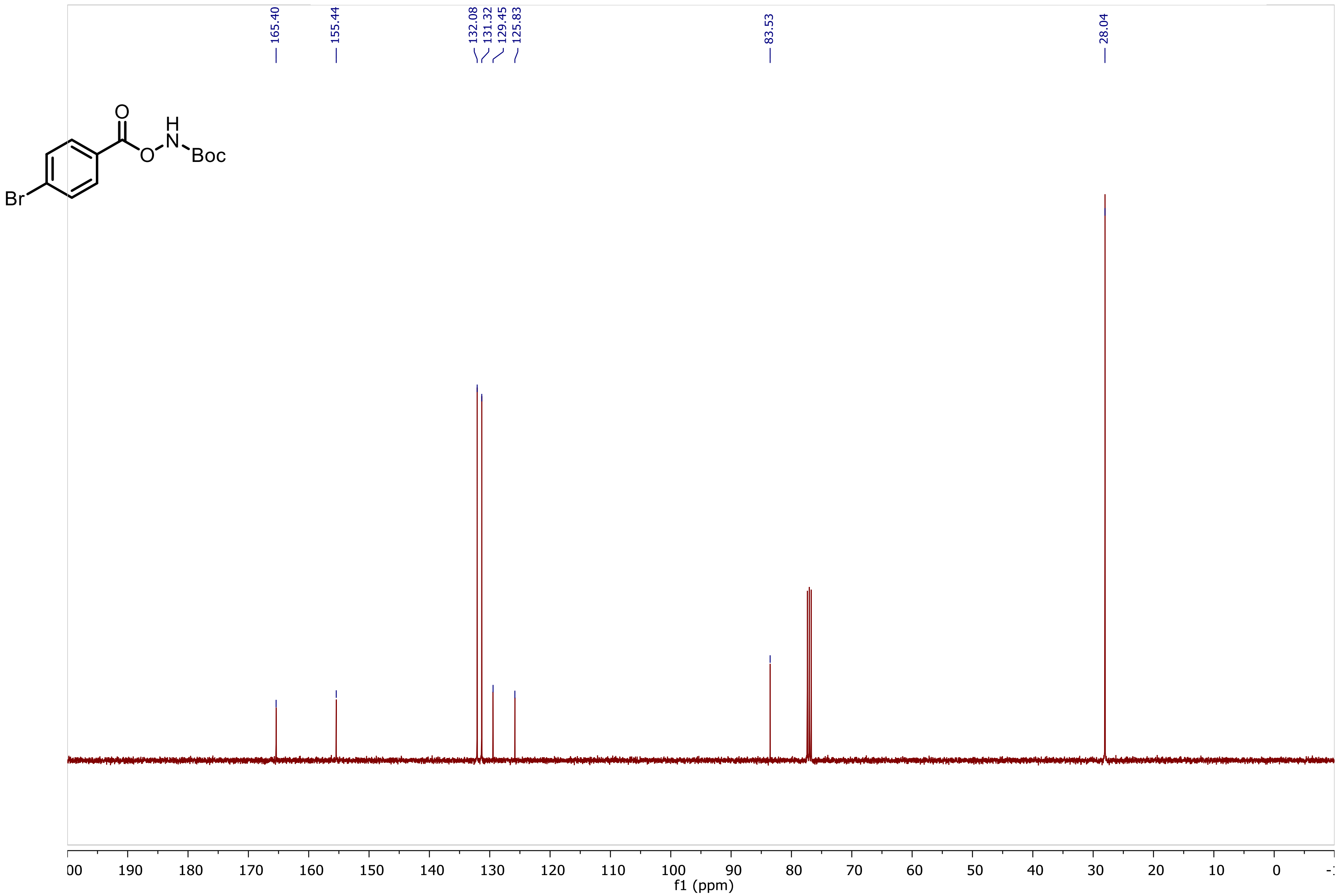

^{1}H NMR spectrum of *tert*-butyl (3-fluorobenzoyloxy)carbamate **1k** in $CDCl_3$

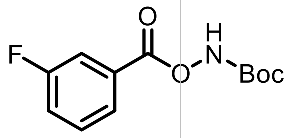

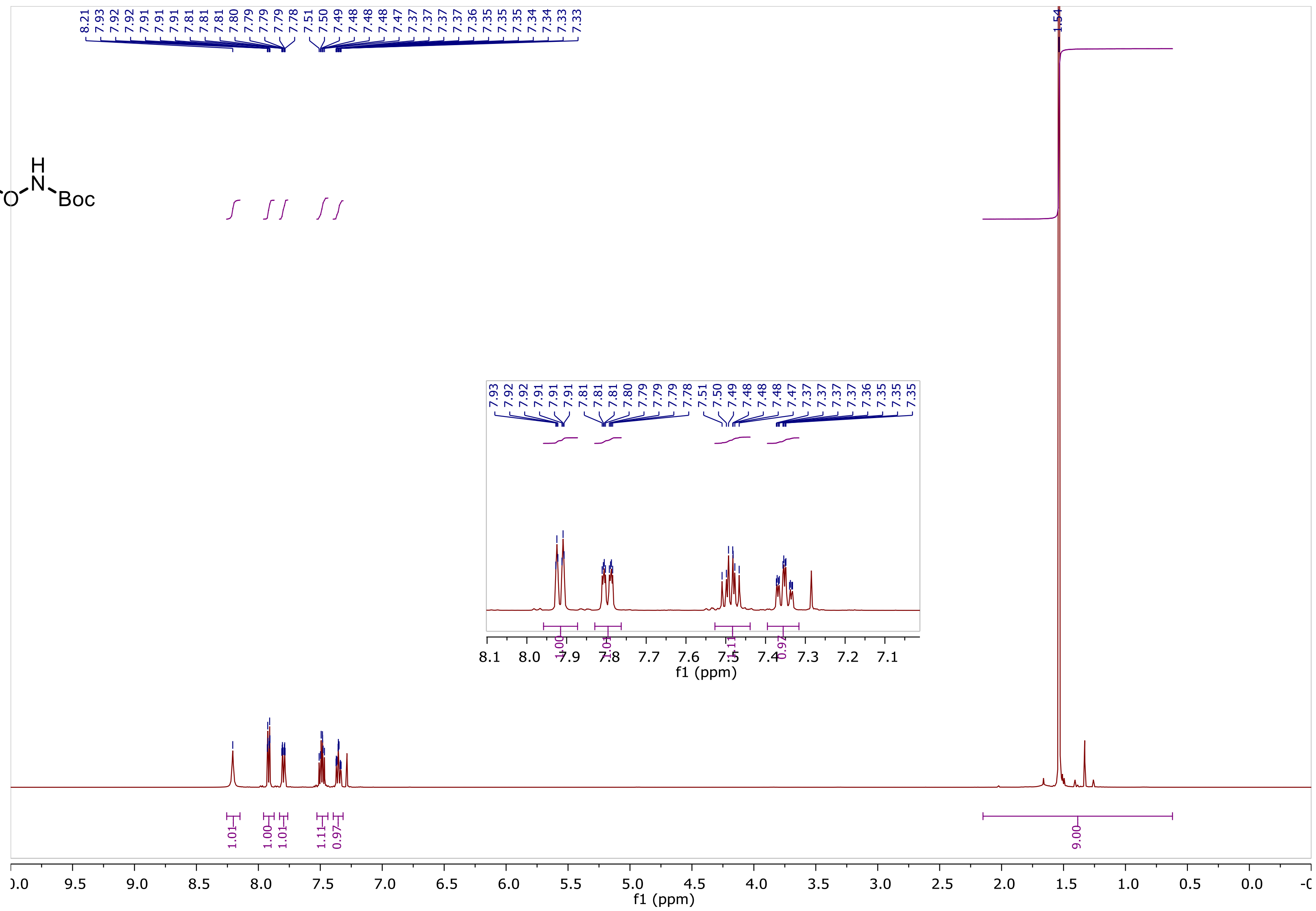

^{13}C NMR spectrum of *tert*-butyl (3-fluorobenzoyloxy)carbamate **1k** in $CDCl_3$

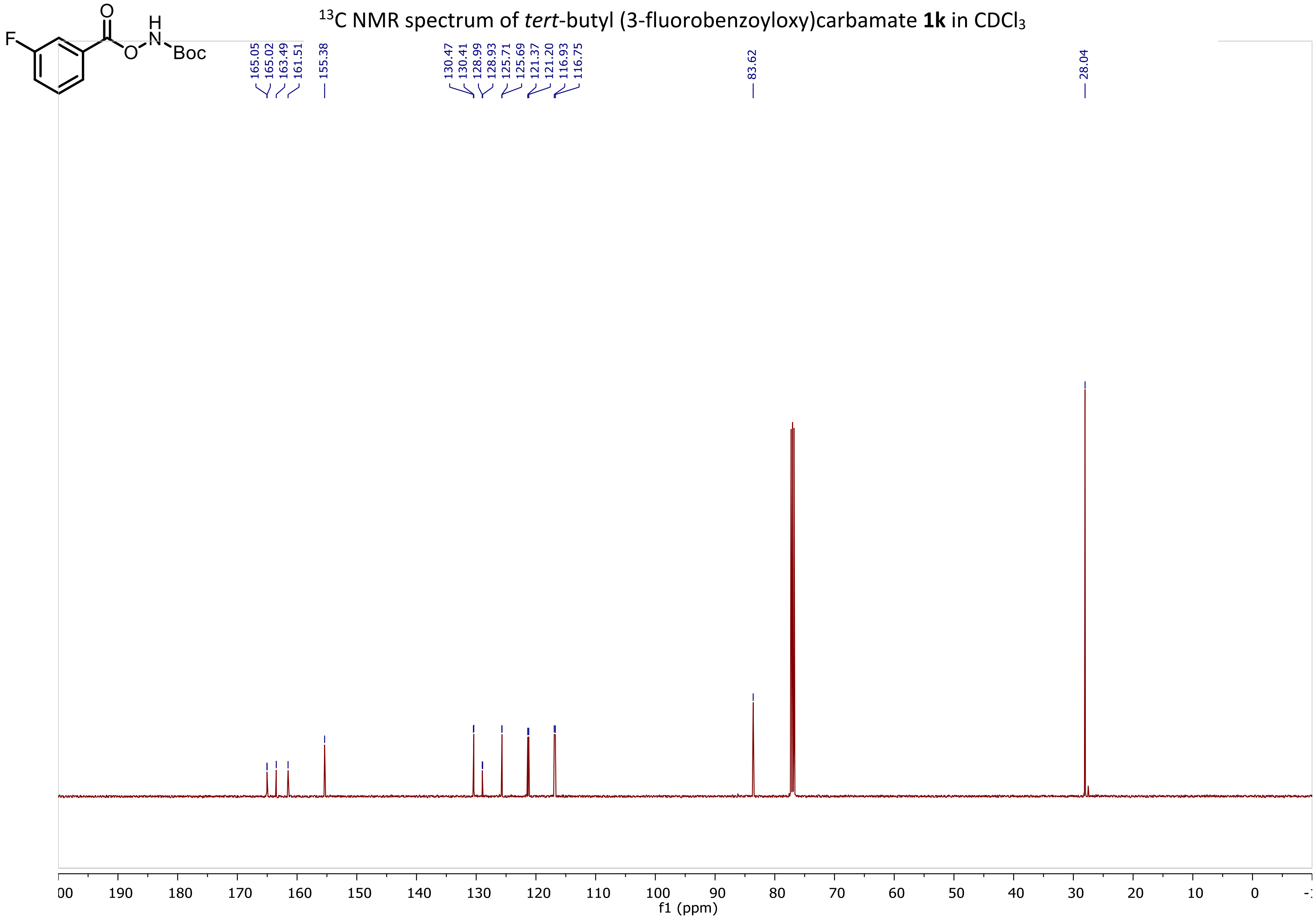

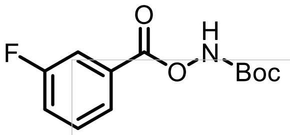

^{19}F NMR spectrum of *tert*-butyl (3-fluorobenzoyloxy)carbamate **1k** in $CDCl_3$

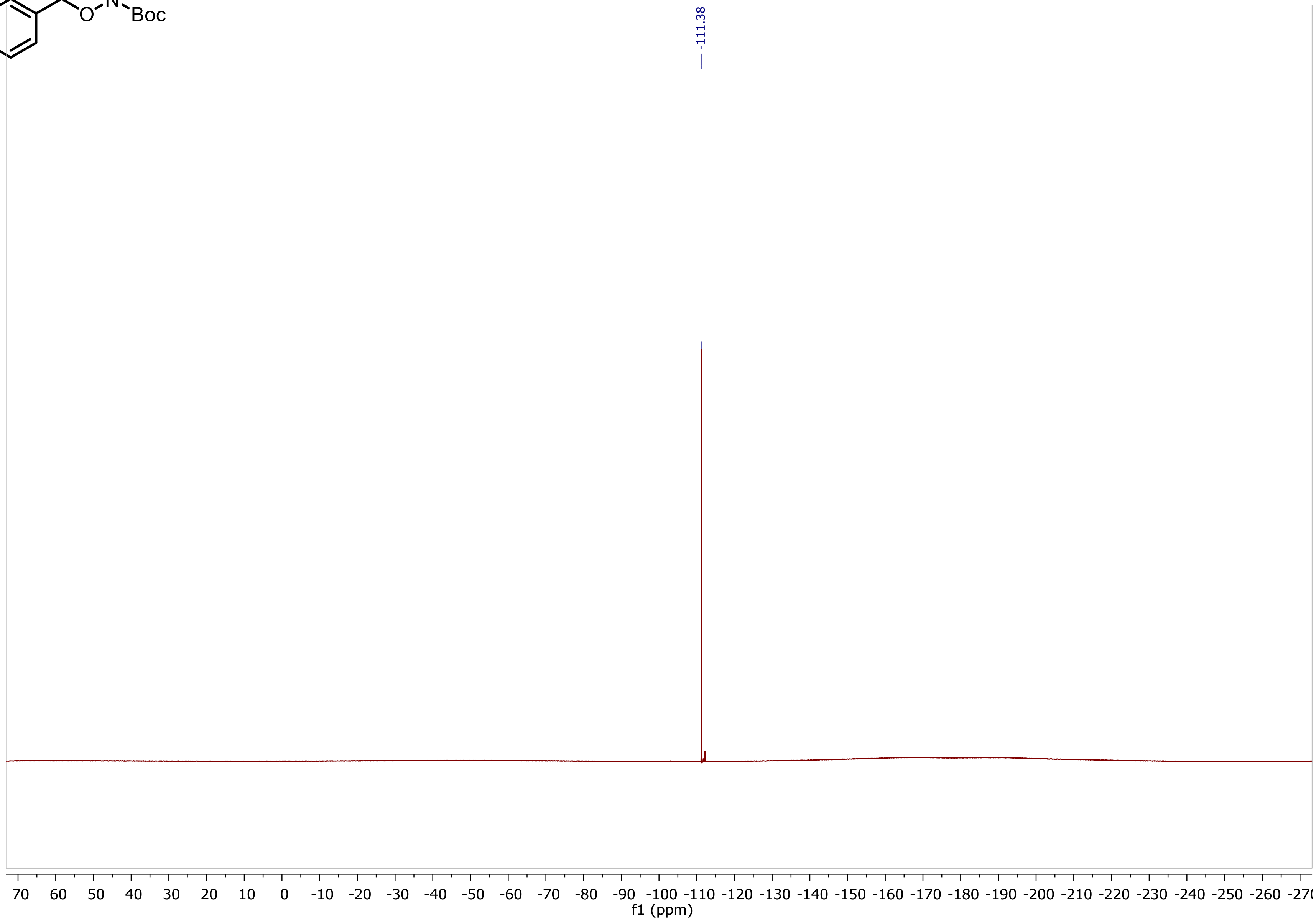

^{1}H NMR spectrum of *tert*-butyl (3-fluoro-2-methylbenzoyloxy)carbamate **1l** in $CDCl_3$

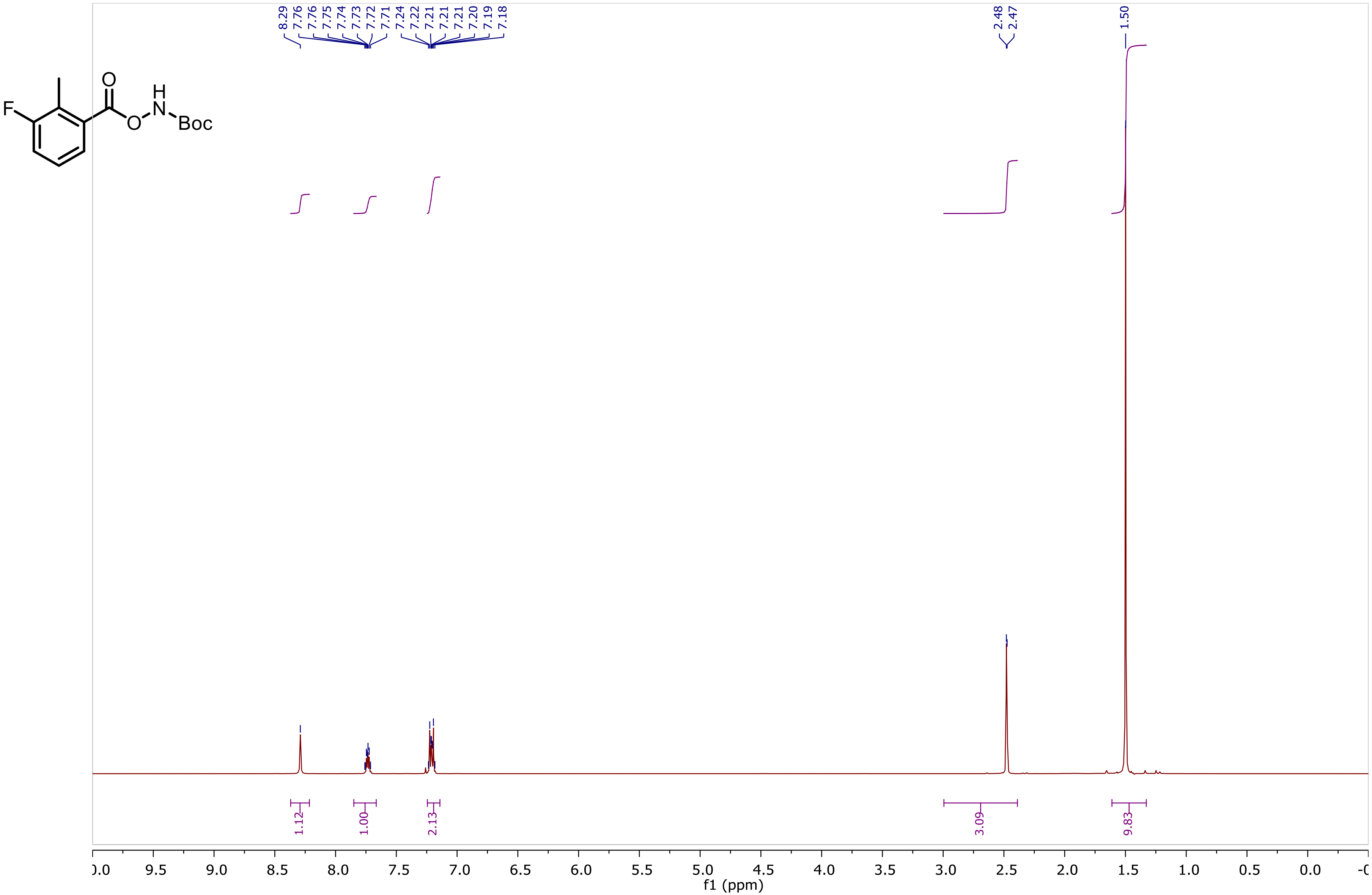

^{13}C NMR spectrum of *tert*-butyl (3-fluoro-2-methylbenzoyloxy)carbamate **1l** in $CDCl_3$

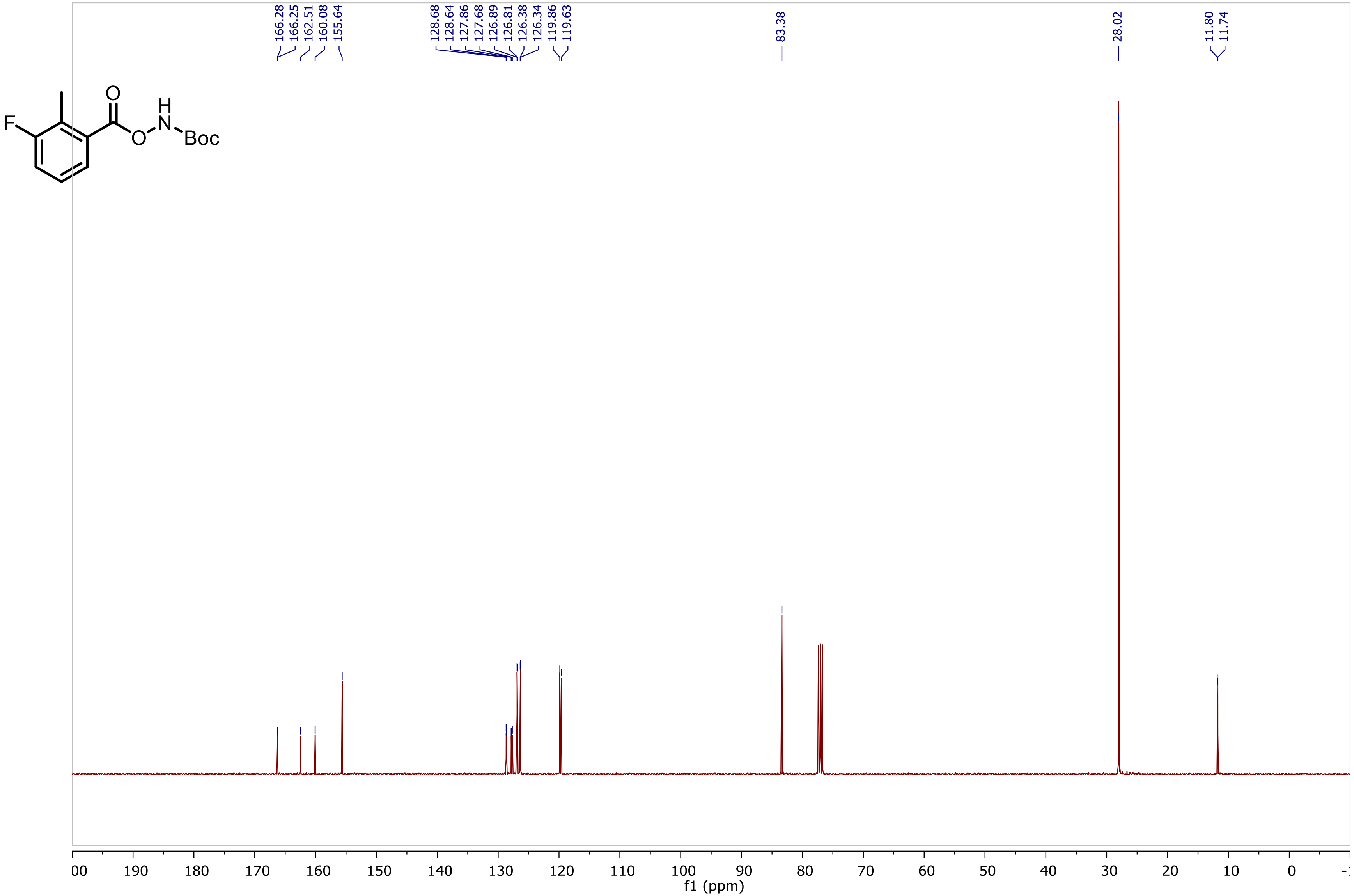

^{19}F NMR spectrum of *tert*-butyl (3-fluoro-2-methylbenzoyloxy)carbamate **1l** in $CDCl_3$

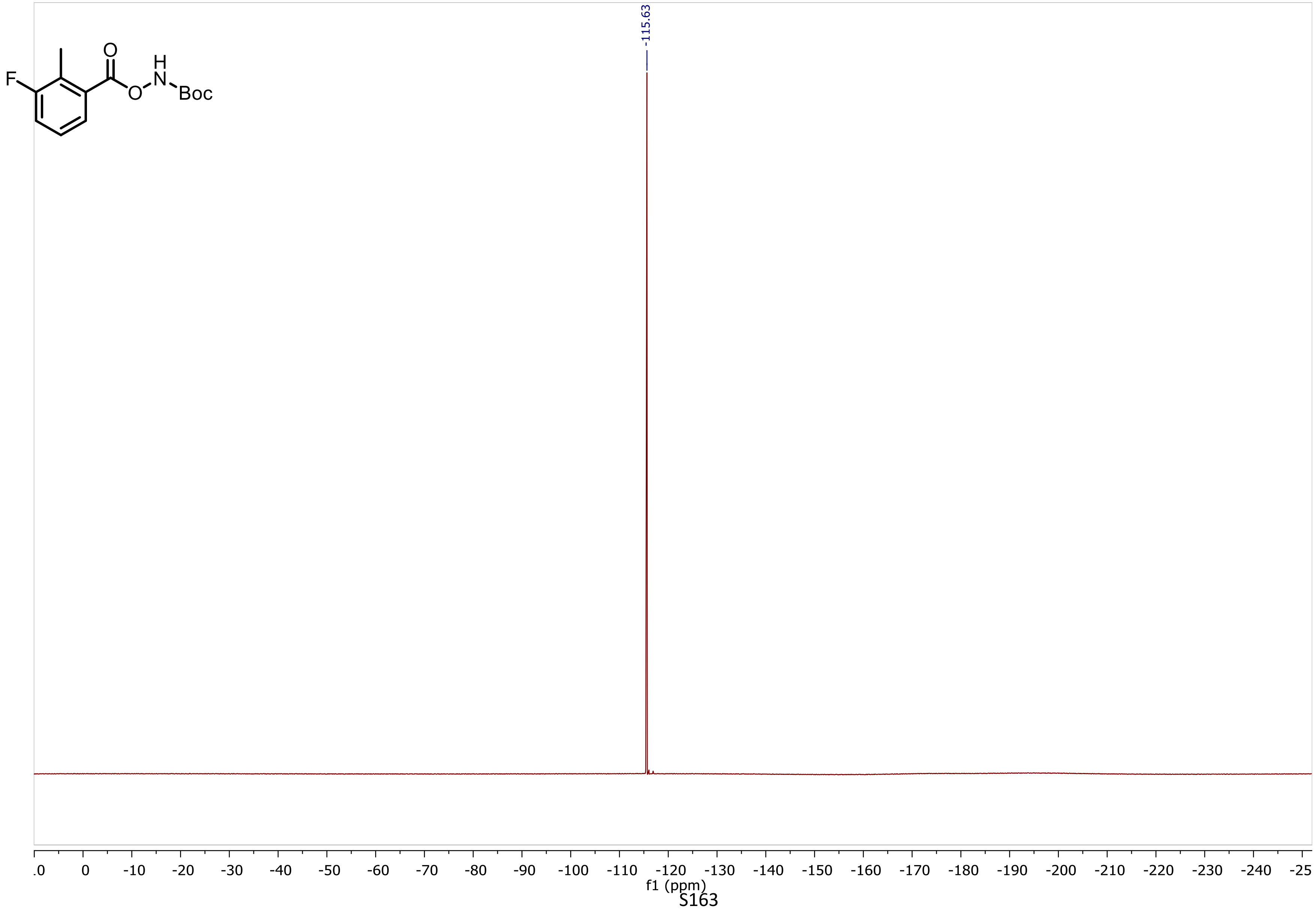

^{1}H NMR spectrum of *tert*-butyl (2-chloro-3-methylbenzoyloxy)carbamate **1n** in $CDCl_3$

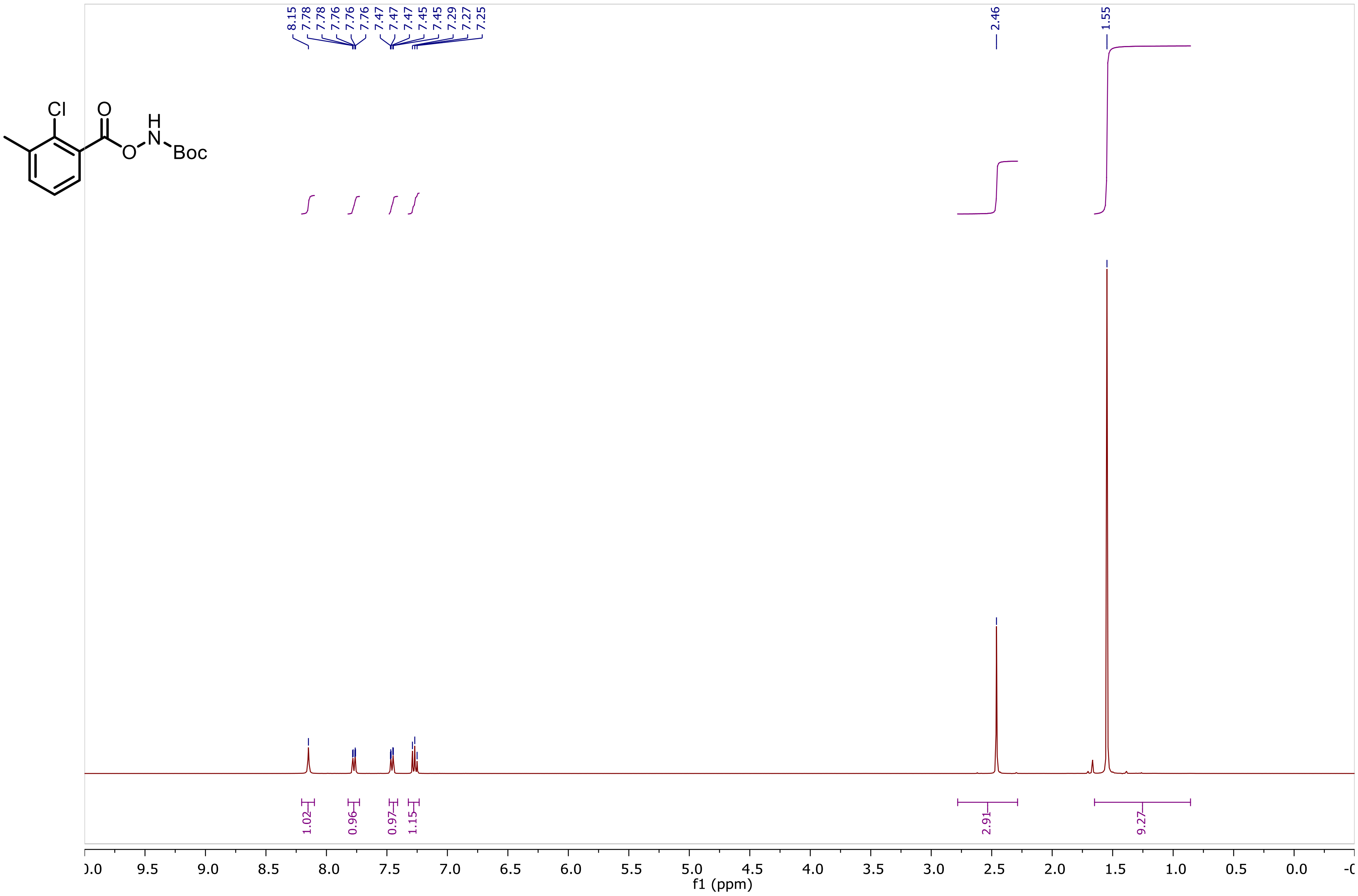

^{13}C NMR spectrum of *tert*-butyl (2-chloro-3-methylbenzoyloxy)carbamate **1n** in $CDCl_3$

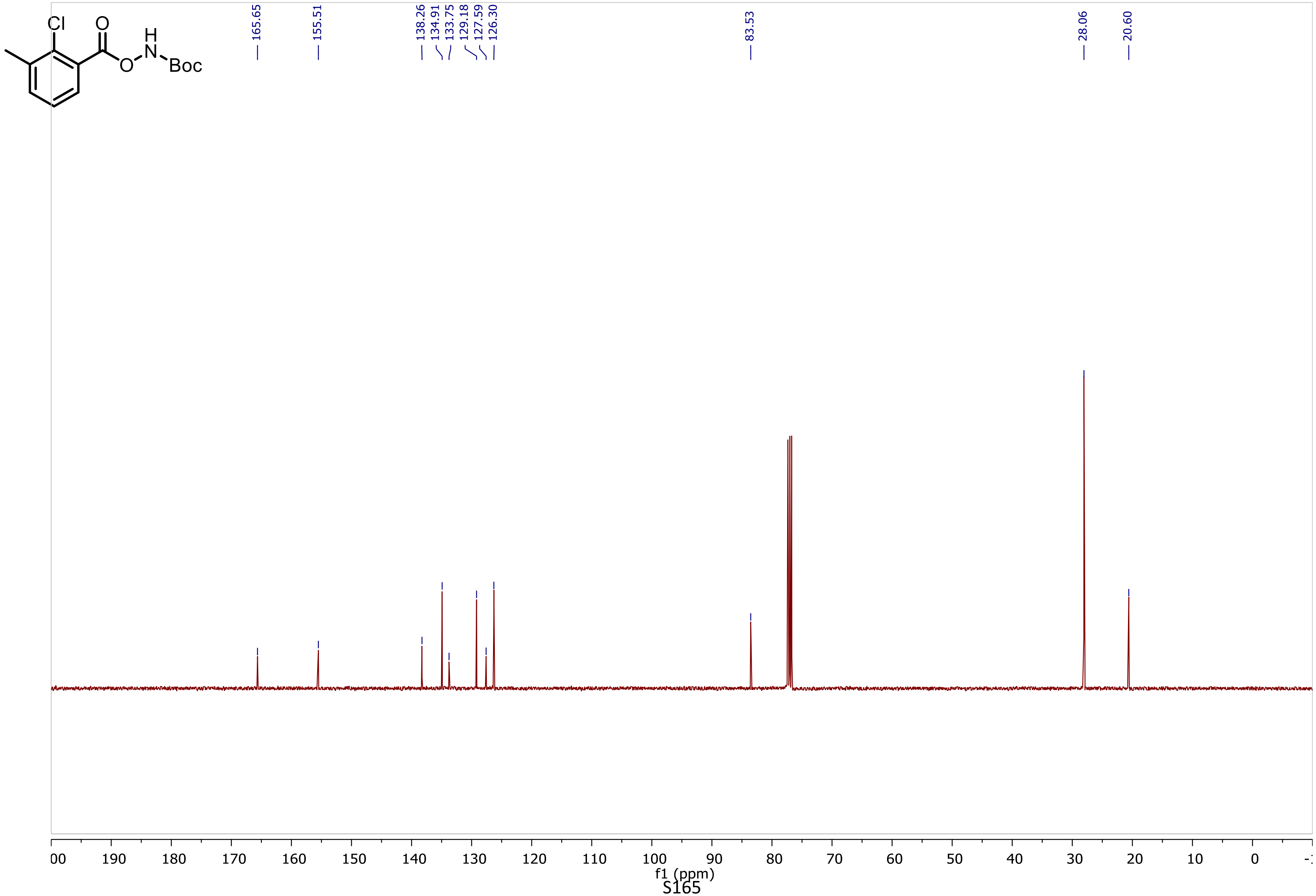

^{1}H NMR spectrum of *tert*-butyl (2-chlorobenzoyloxy)carbamate **1n** in $CDCl_3$

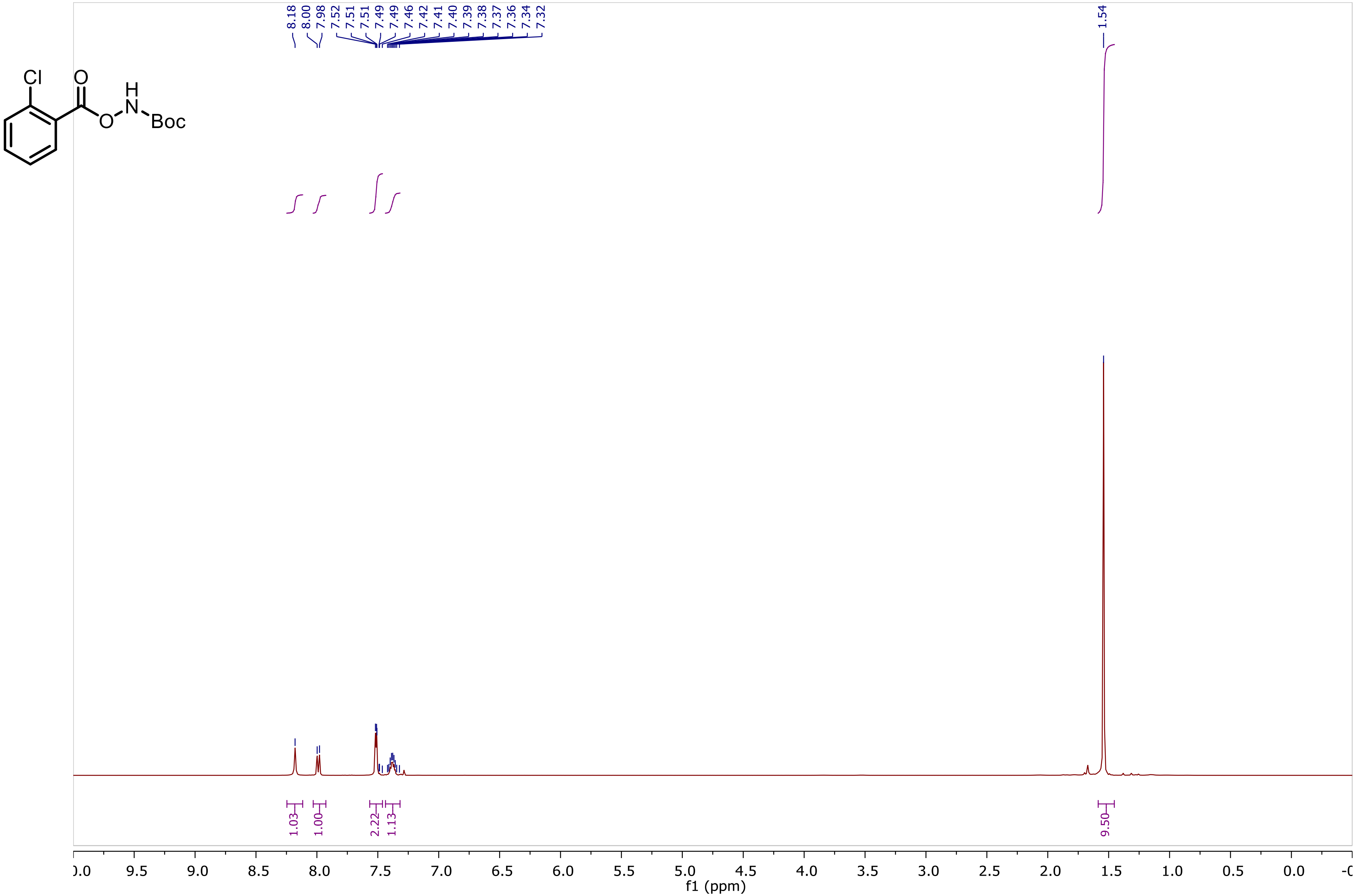

^{13}C NMR spectrum of *tert*-butyl (2-chlorobenzoyloxy)carbamate **1o** in $CDCl_3$

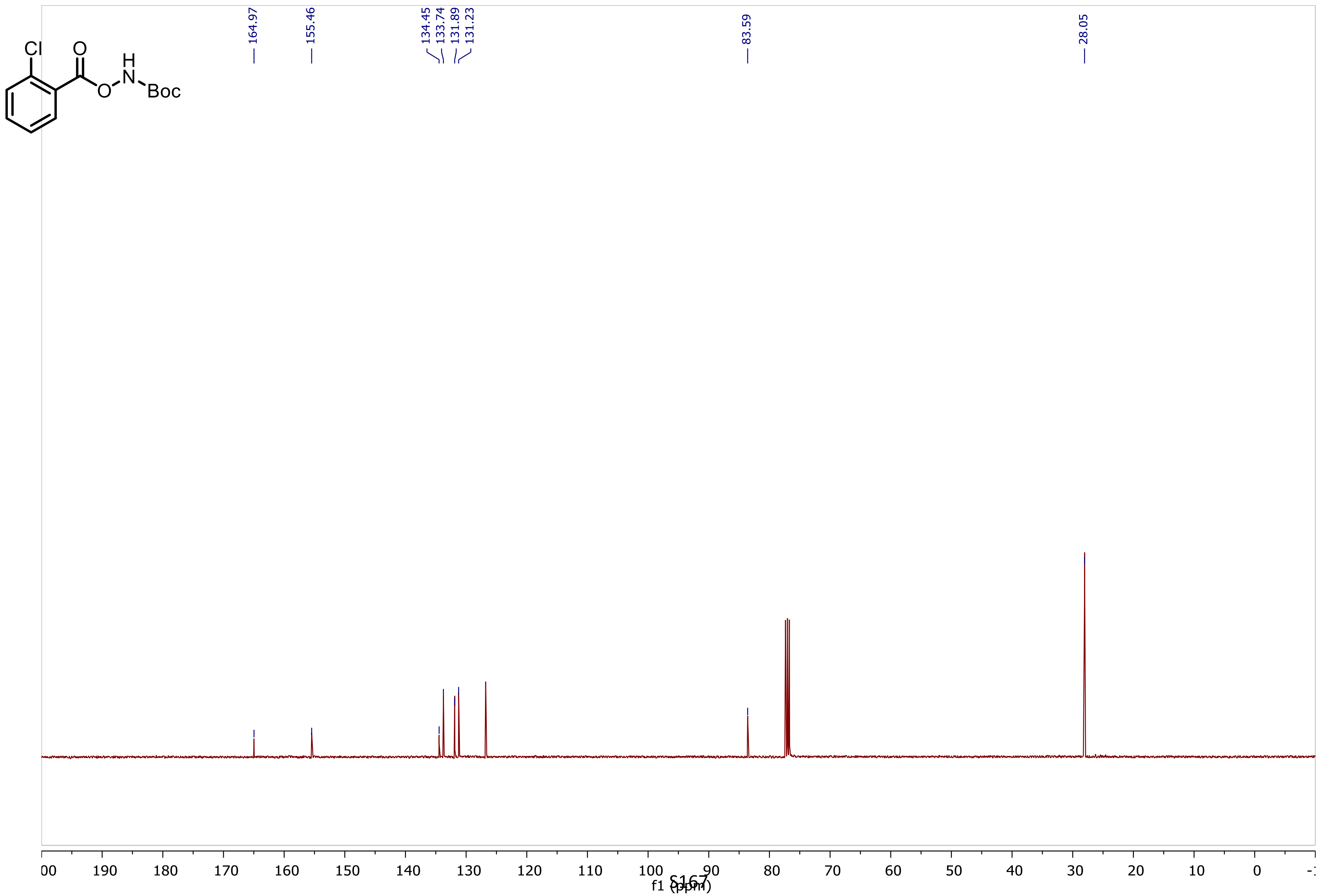

^{1}H NMR spectrum of *tert*-butyl (3-chlorobenzoyloxy)carbamate **1o** in $CDCl_3$

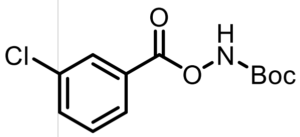

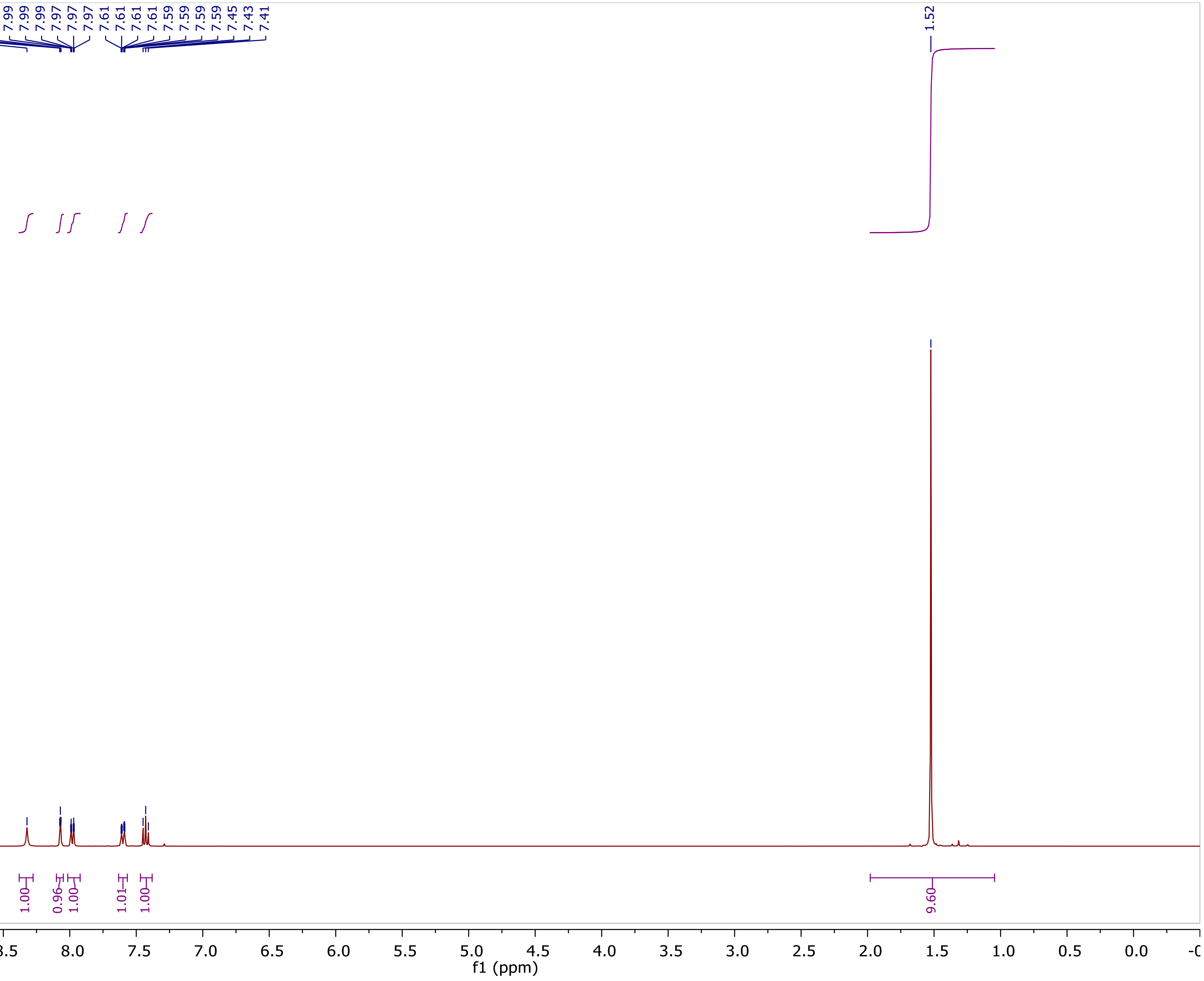

^{13}C NMR spectrum of *tert*-butyl (3-chlorobenzoyloxy)carbamate **1p** in $CDCl_3$

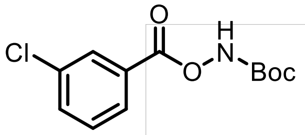

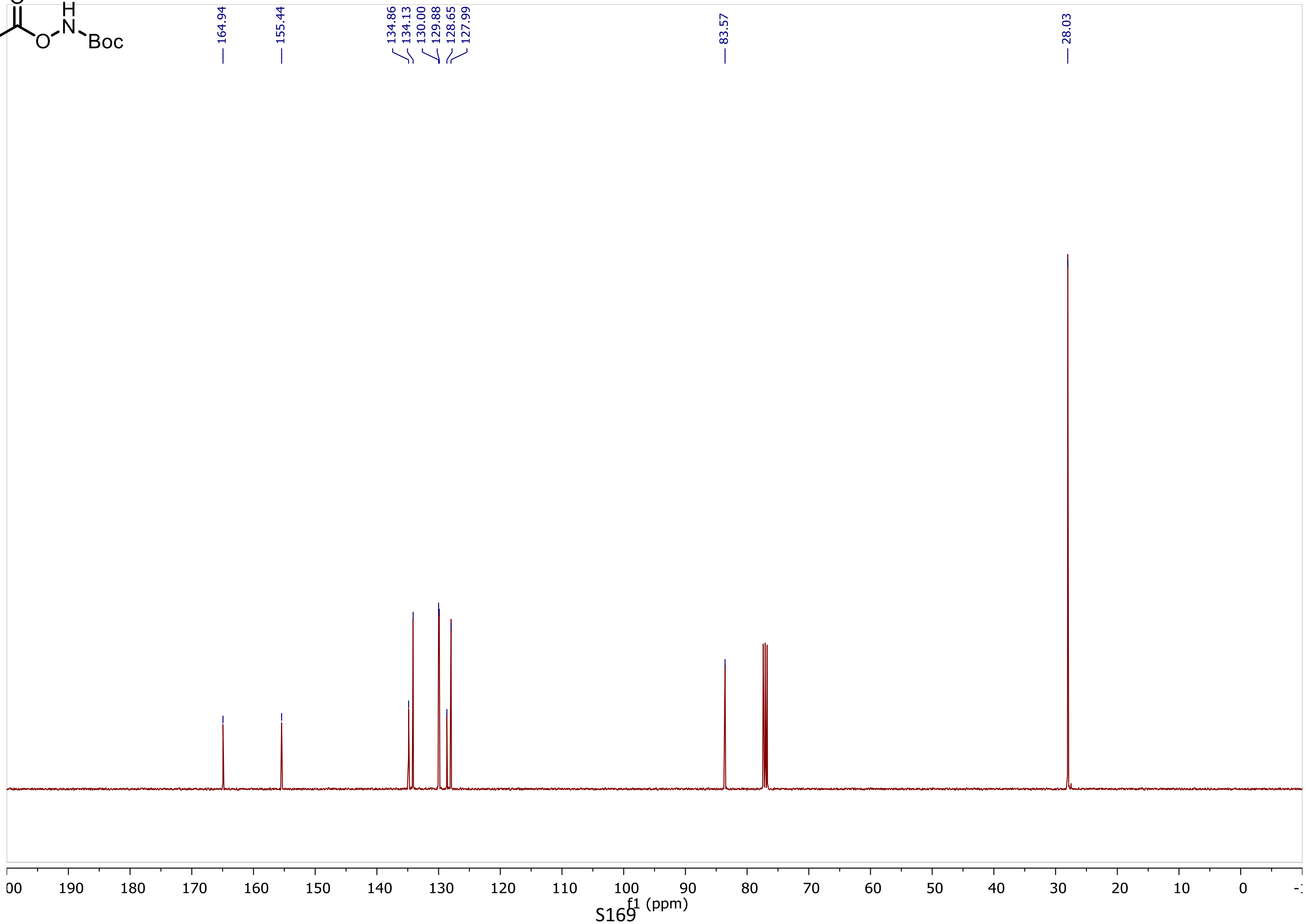

^{1}H NMR spectrum of *tert*-butyl (3-chloro-2-methylbenzoyloxy)carbamate **1p** in $CDCl_3$

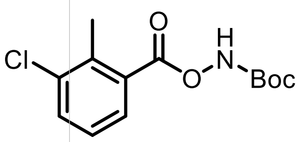

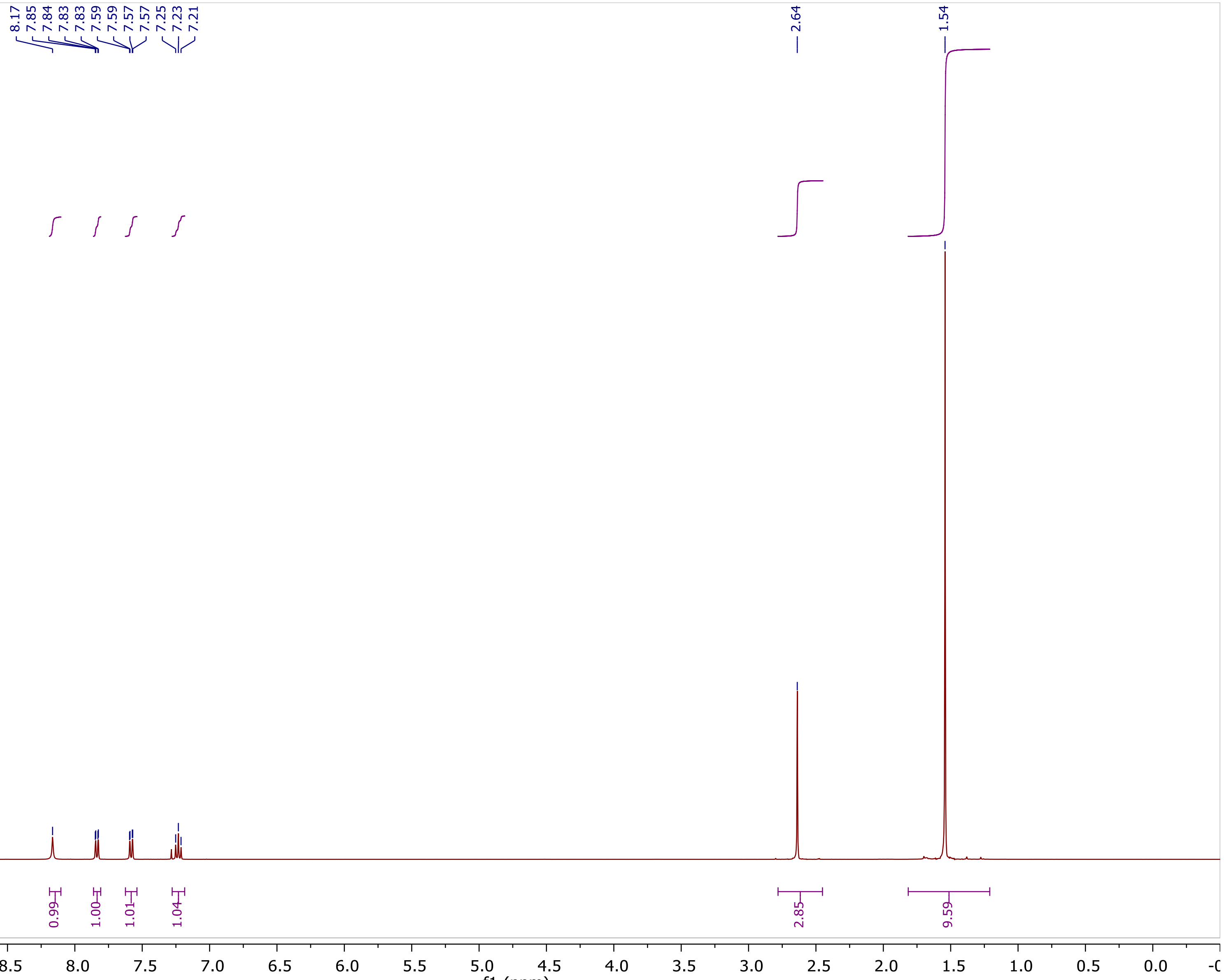

^{13}C NMR spectrum of *tert*-butyl (3-chloro-2-methylbenzoyloxy)carbamate **1q** in $CDCl_3$

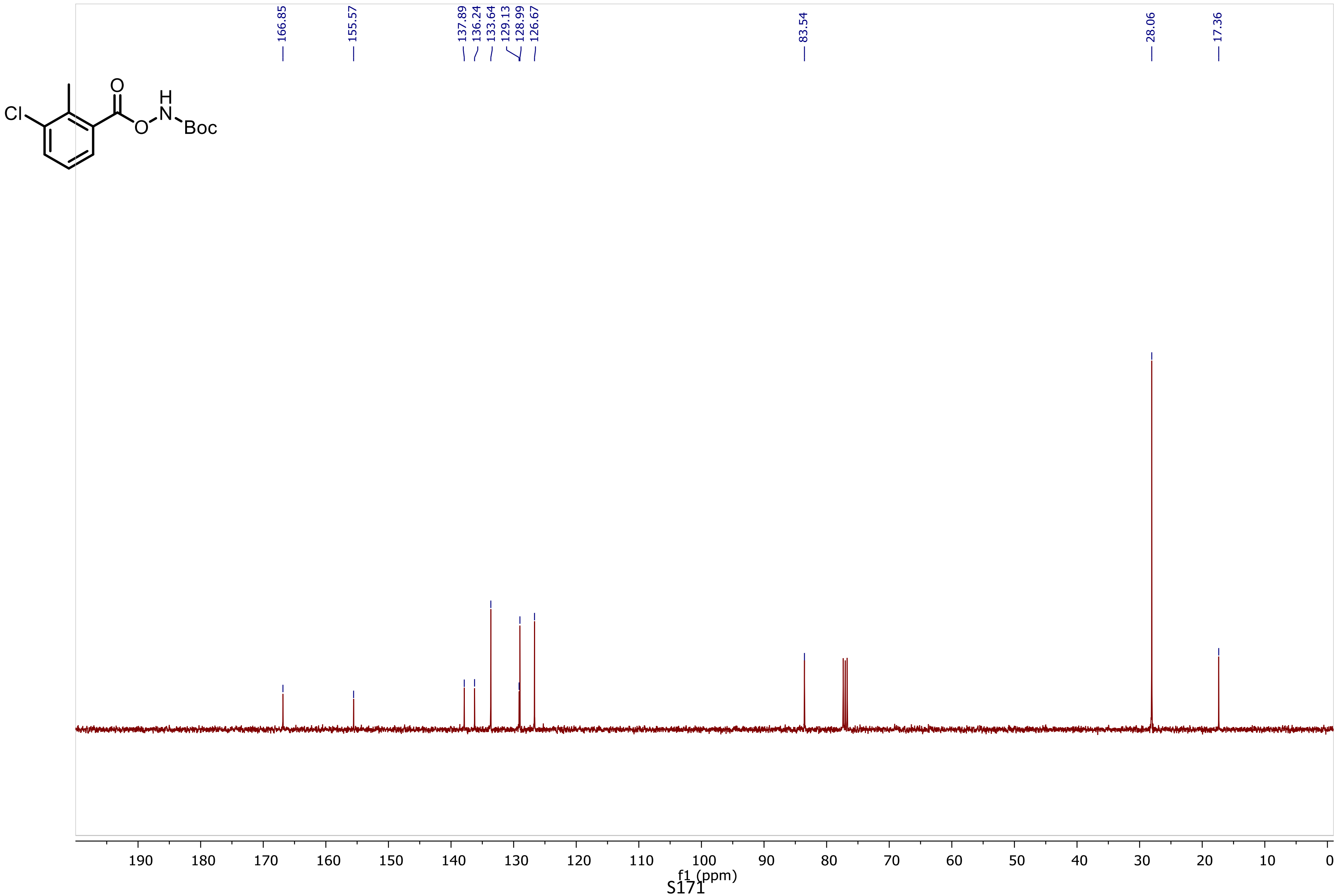

^{1}H NMR spectrum of *tert*-butyl (2-bromobenzoyloxy)carbamate **1s** in $CDCl_3$

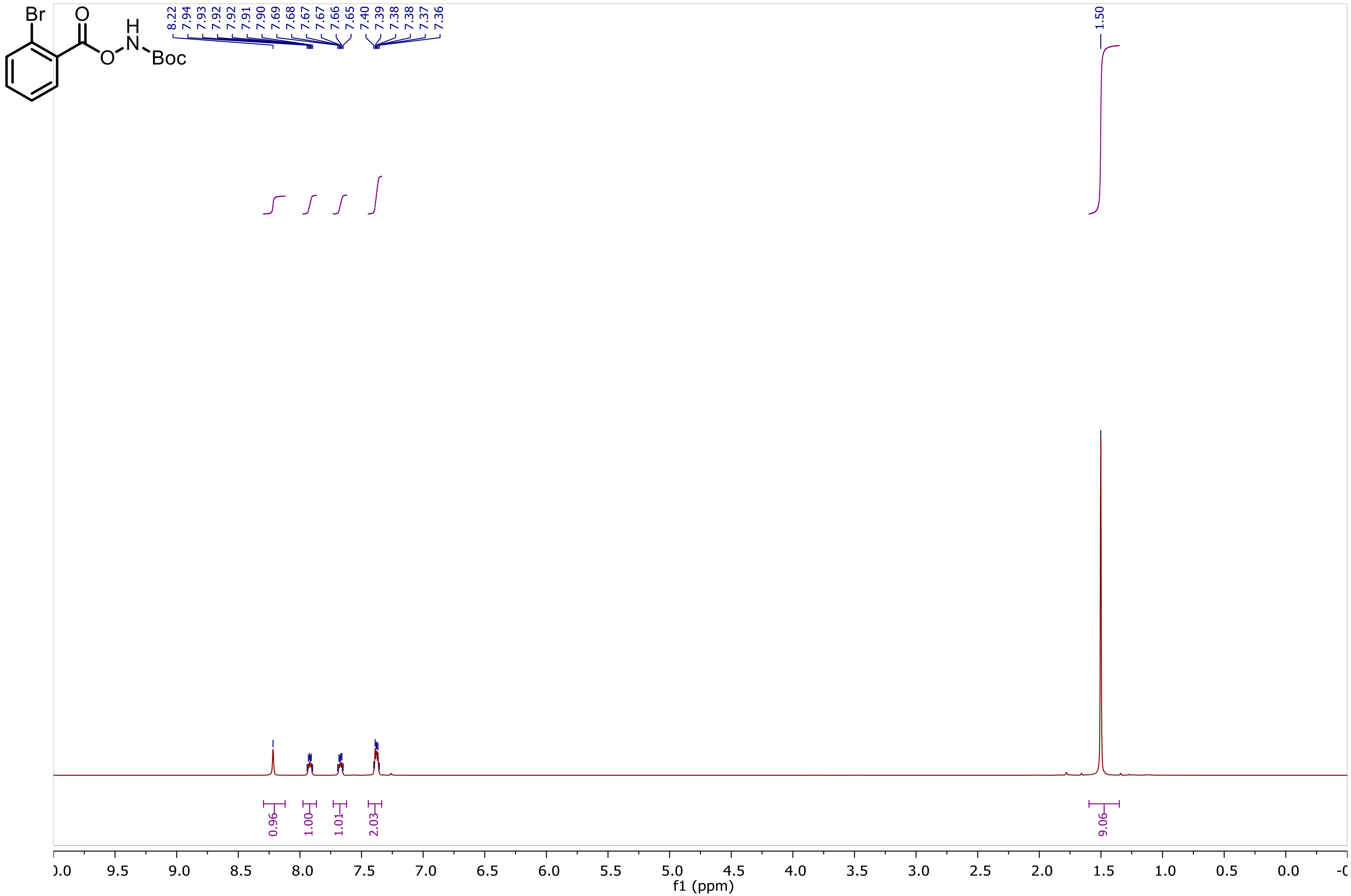

^{13}C NMR spectrum of *tert*-butyl (2-bromobenzoyloxy)carbamate **1s** in $CDCl_3$

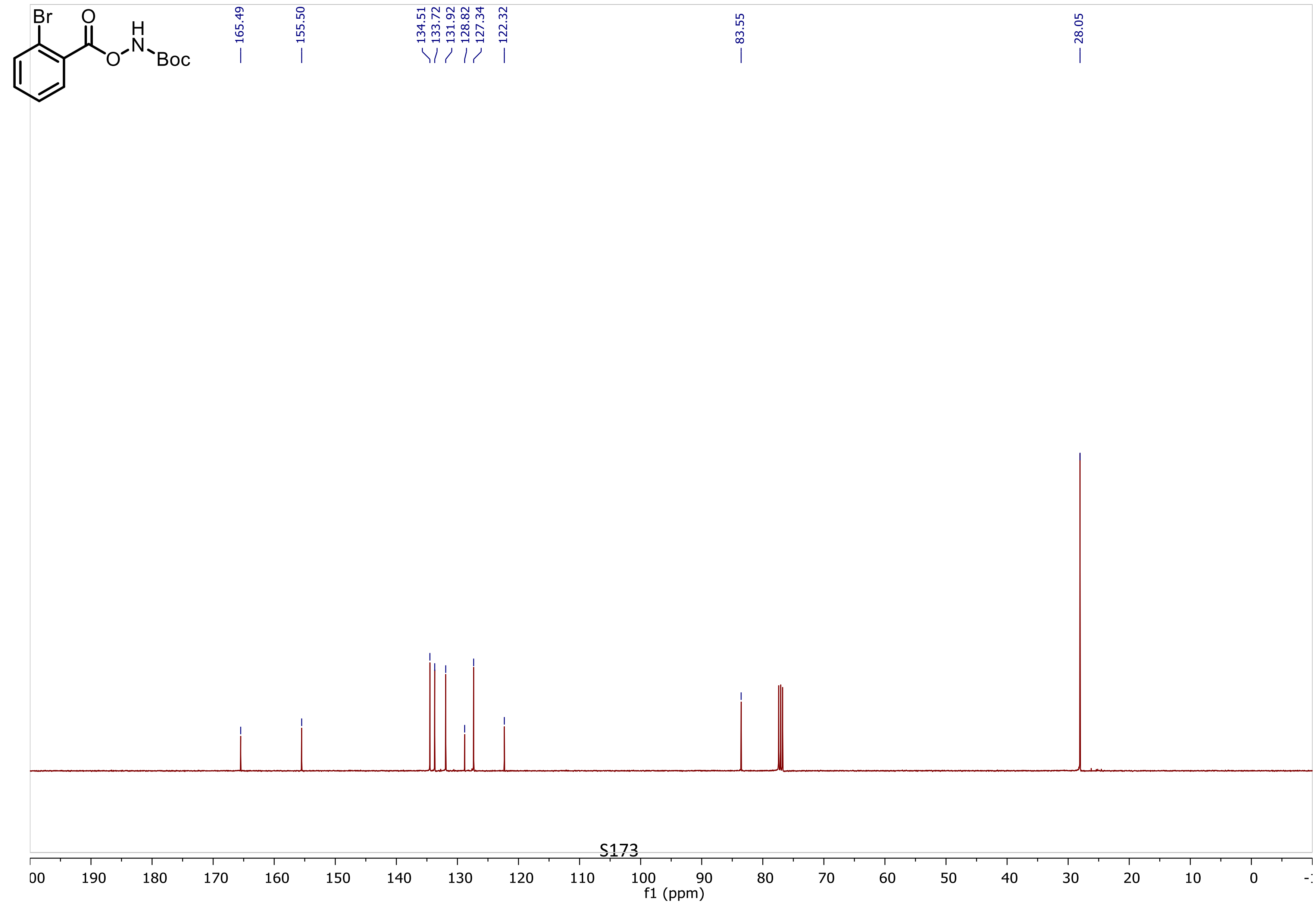

^{1}H NMR spectrum of *tert*-butyl (2-bromo-3-methylbenzoyloxy)carbamate **1t** in $CDCl_3$

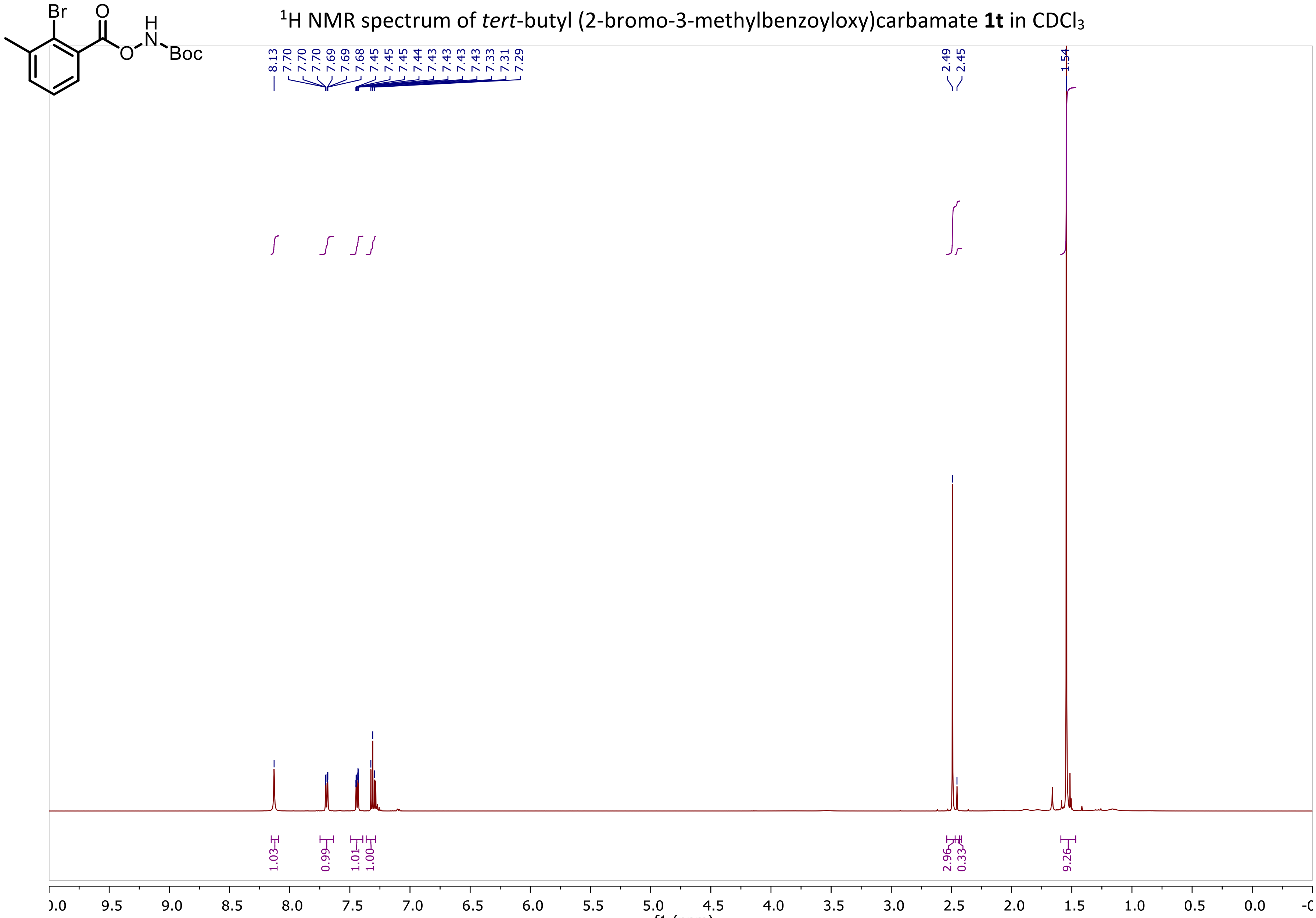

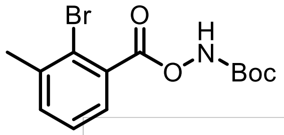

^{13}C NMR spectrum of *tert*-butyl (2-bromo-3-methylbenzoyloxy)carbamate **1t** in $CDCl_3$

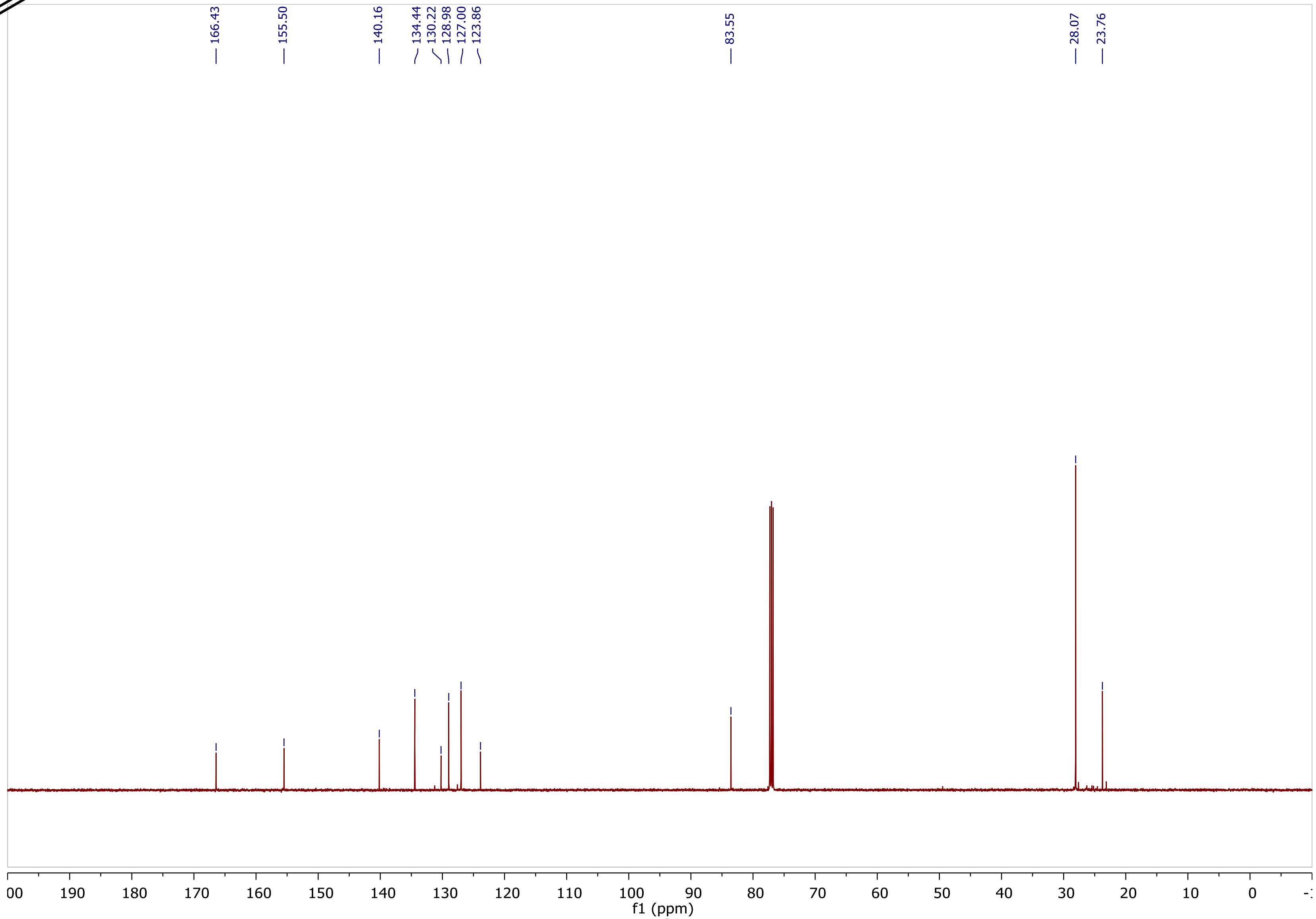

^{1}H NMR spectrum of *tert*-butyl (3-bromobenzoyloxy)carbamate **1u** in $CDCl_3$

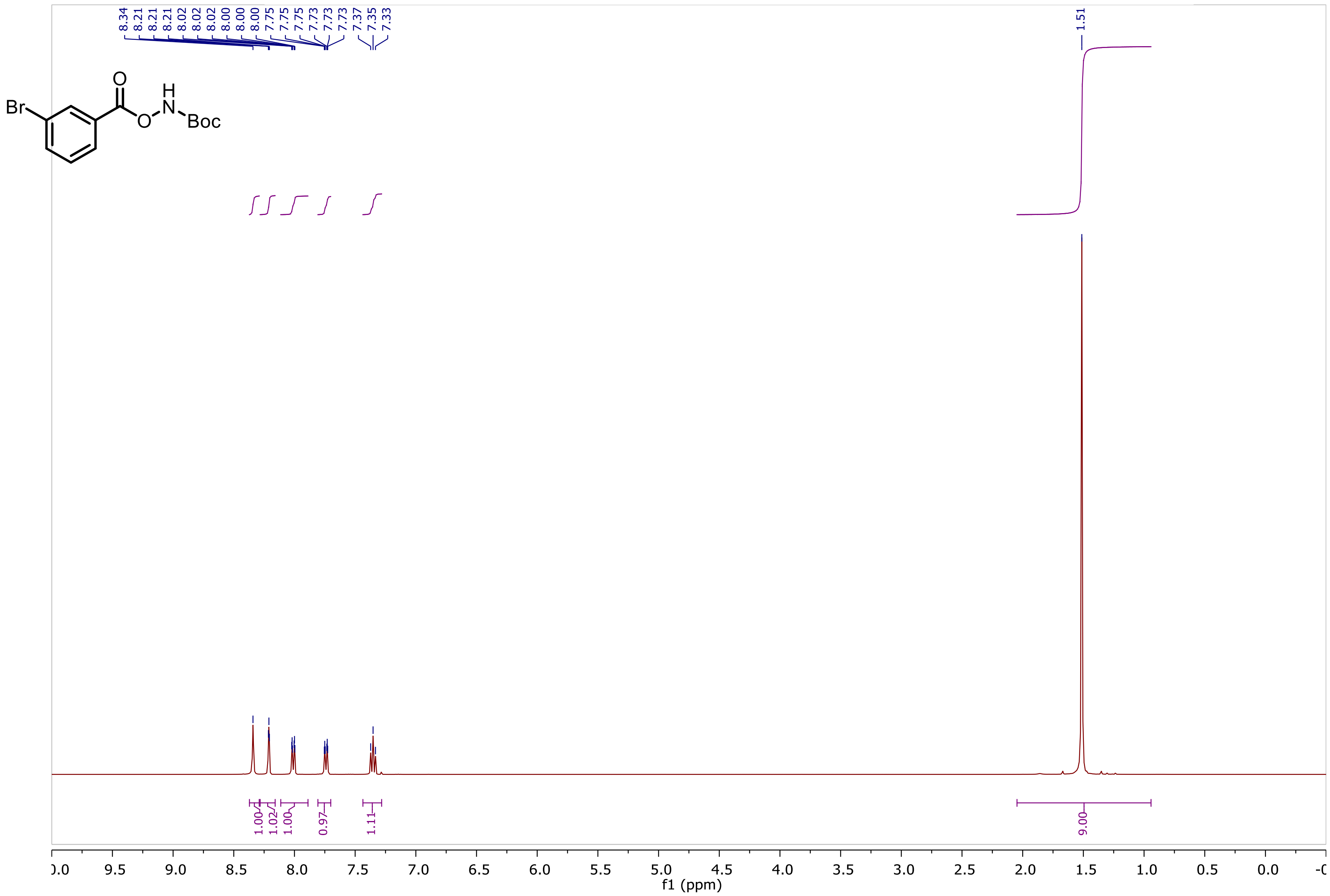

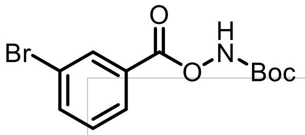

^{13}C NMR spectrum of *tert*-butyl (3-bromobenzoyloxy)carbamate **1u** in $CDCl_3$

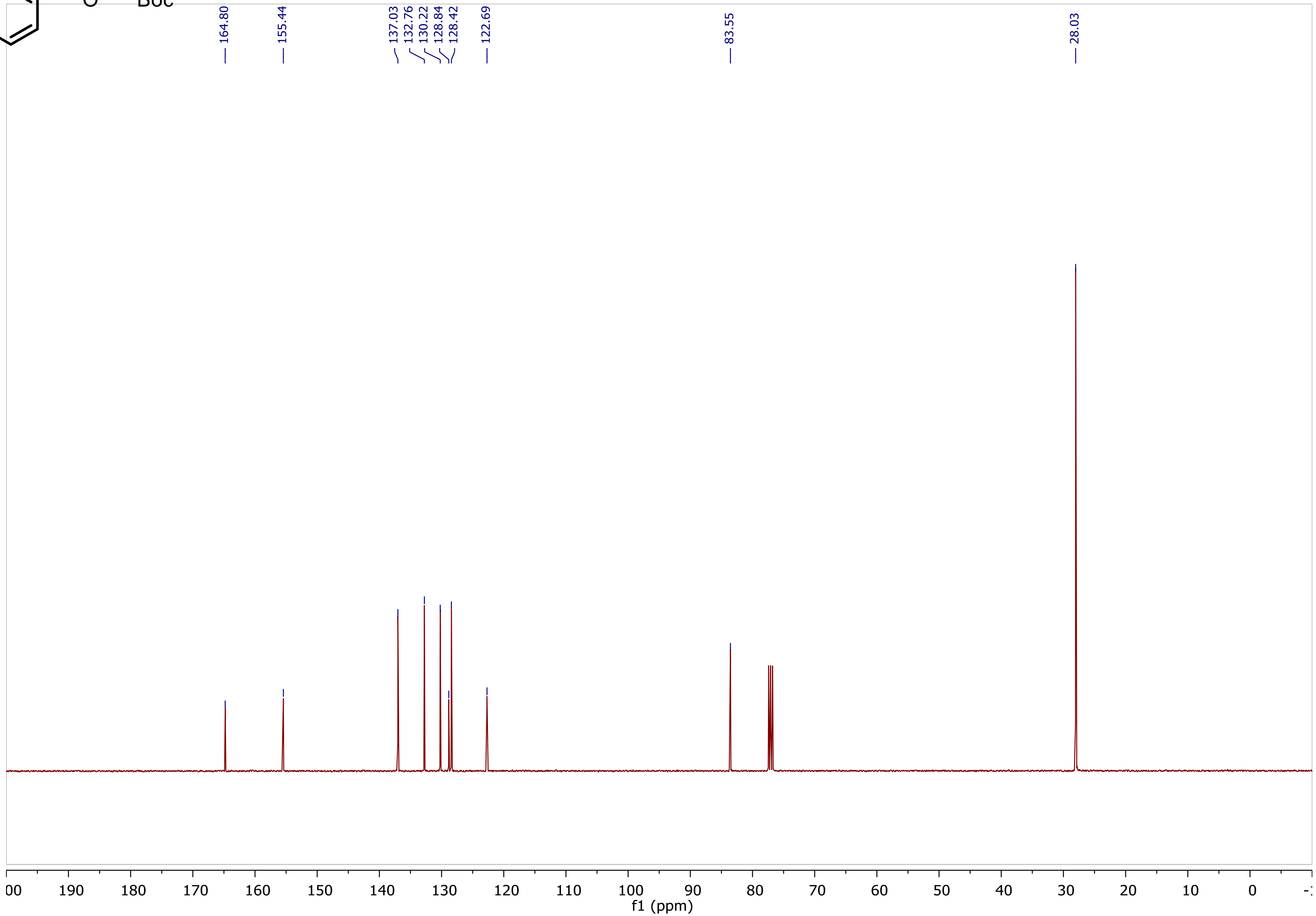

^{1}H NMR spectrum of *tert*-butyl (3-iodobenzoyloxy)carbamate **1v** in $CDCl_3$

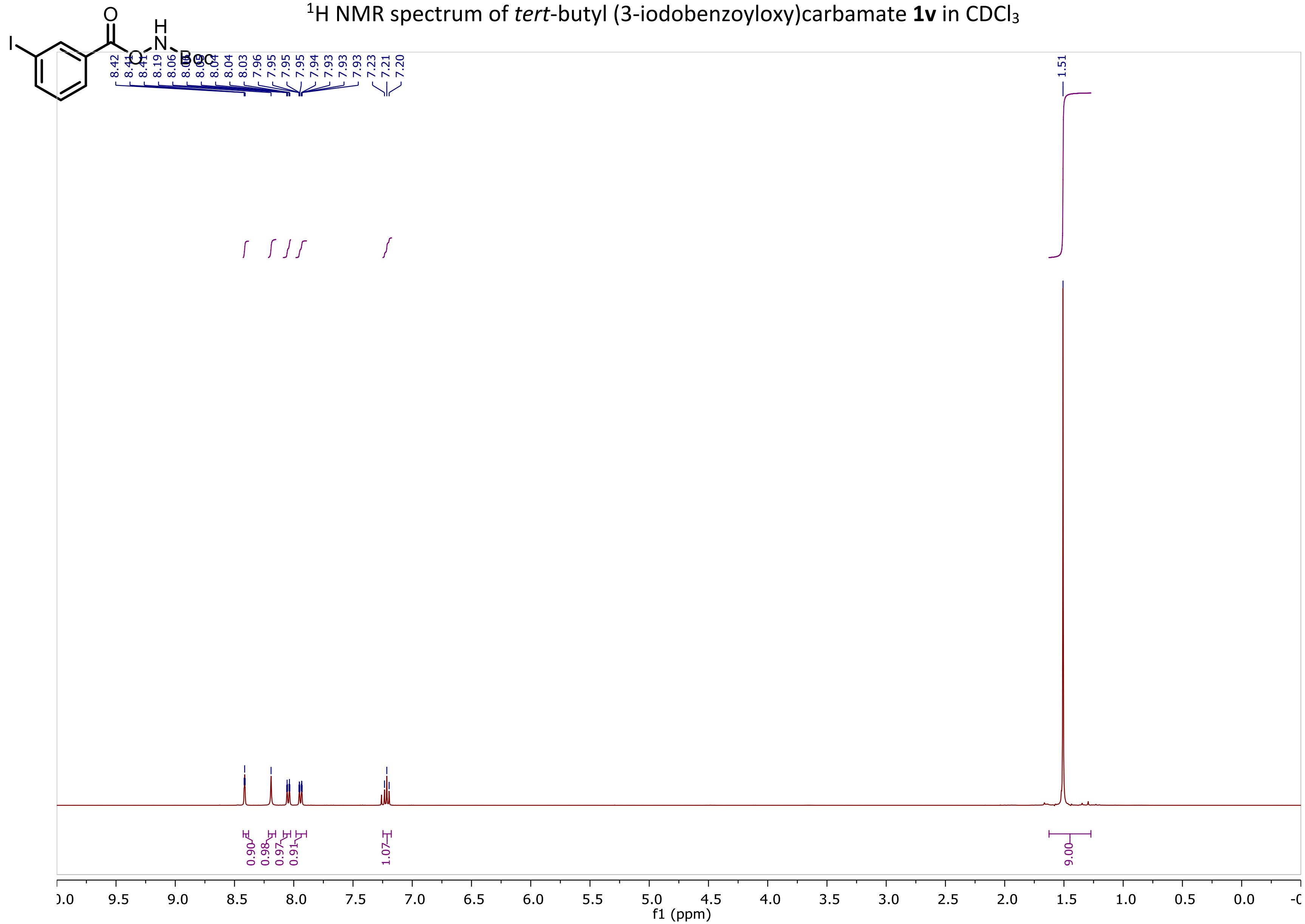

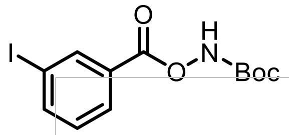

^{13}C NMR spectrum of *tert*-butyl (3-iodobenzoyloxy)carbamate **1v** in $CDCl_3$

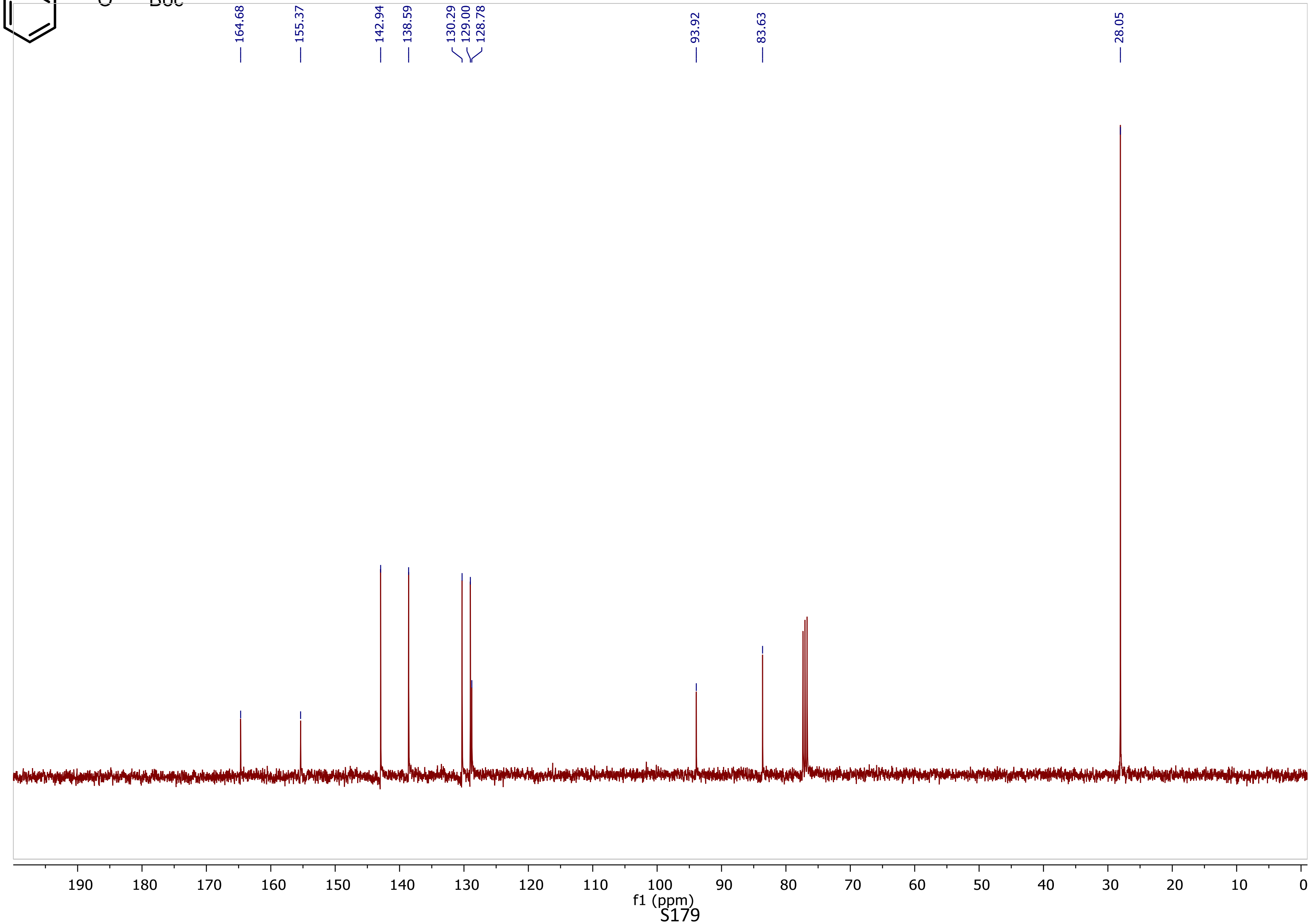

^{1}H NMR spectrum of *tert*-butyl (2,3-dichlorobenzoyloxy)carbamate **1w** in $CDCl_3$

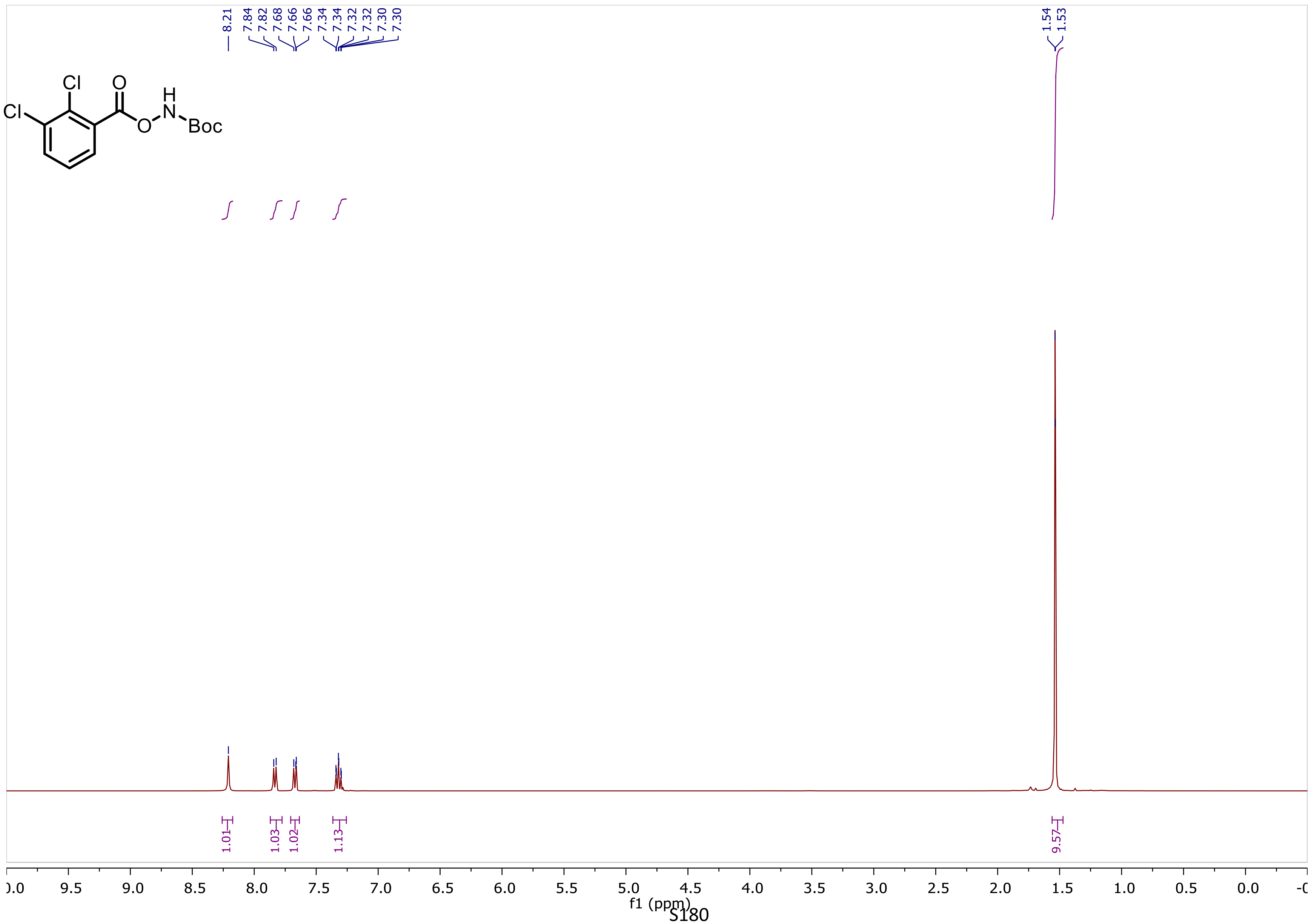

^{13}C NMR spectrum of *tert*-butyl (2,3-dichlorobenzoyloxy)carbamate **1w** in $CDCl_3$

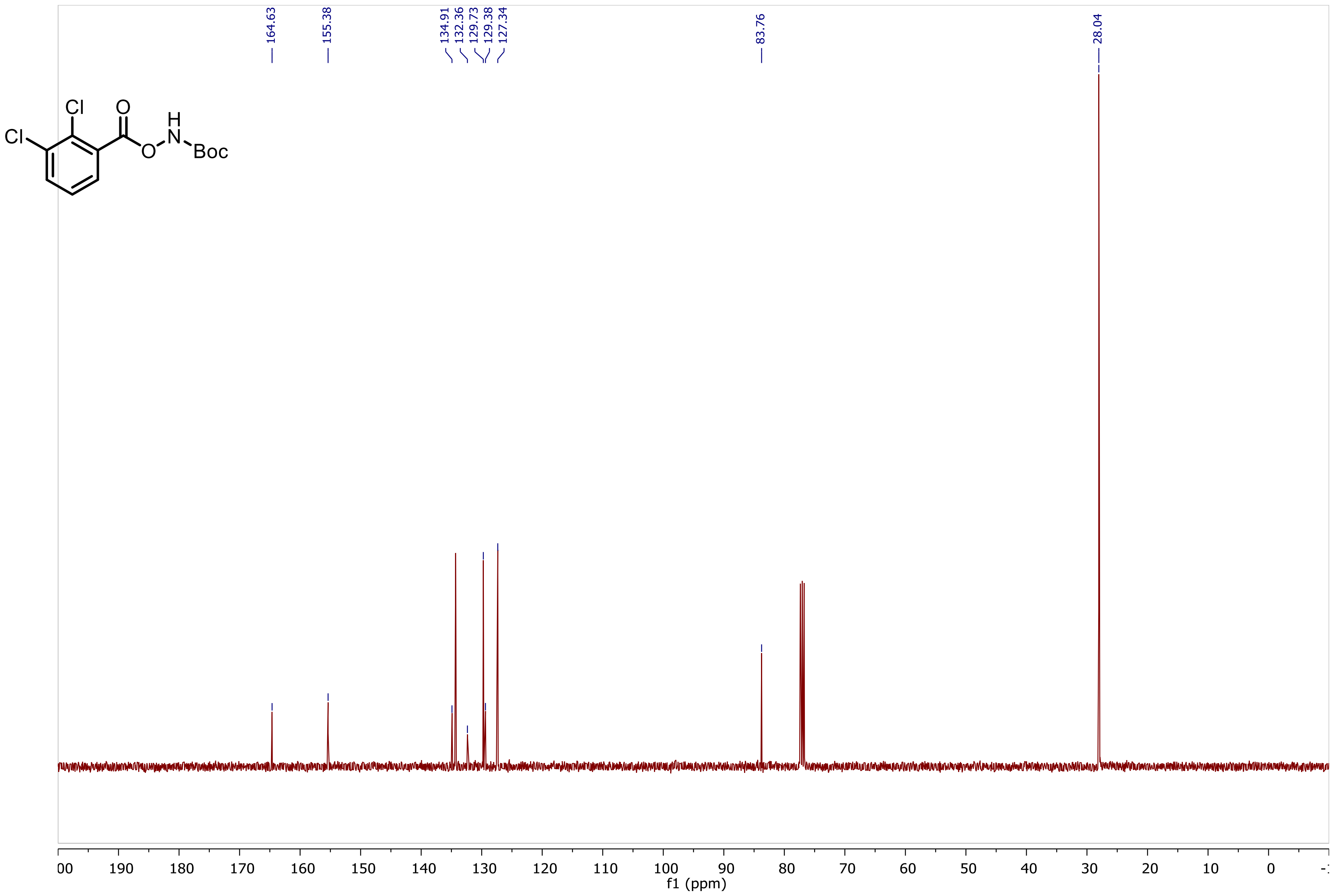

^{1}H NMR spectrum of *tert*-butyl (3,5-dibromobenzoyloxy)carbamate **1x** in $CDCl_3$

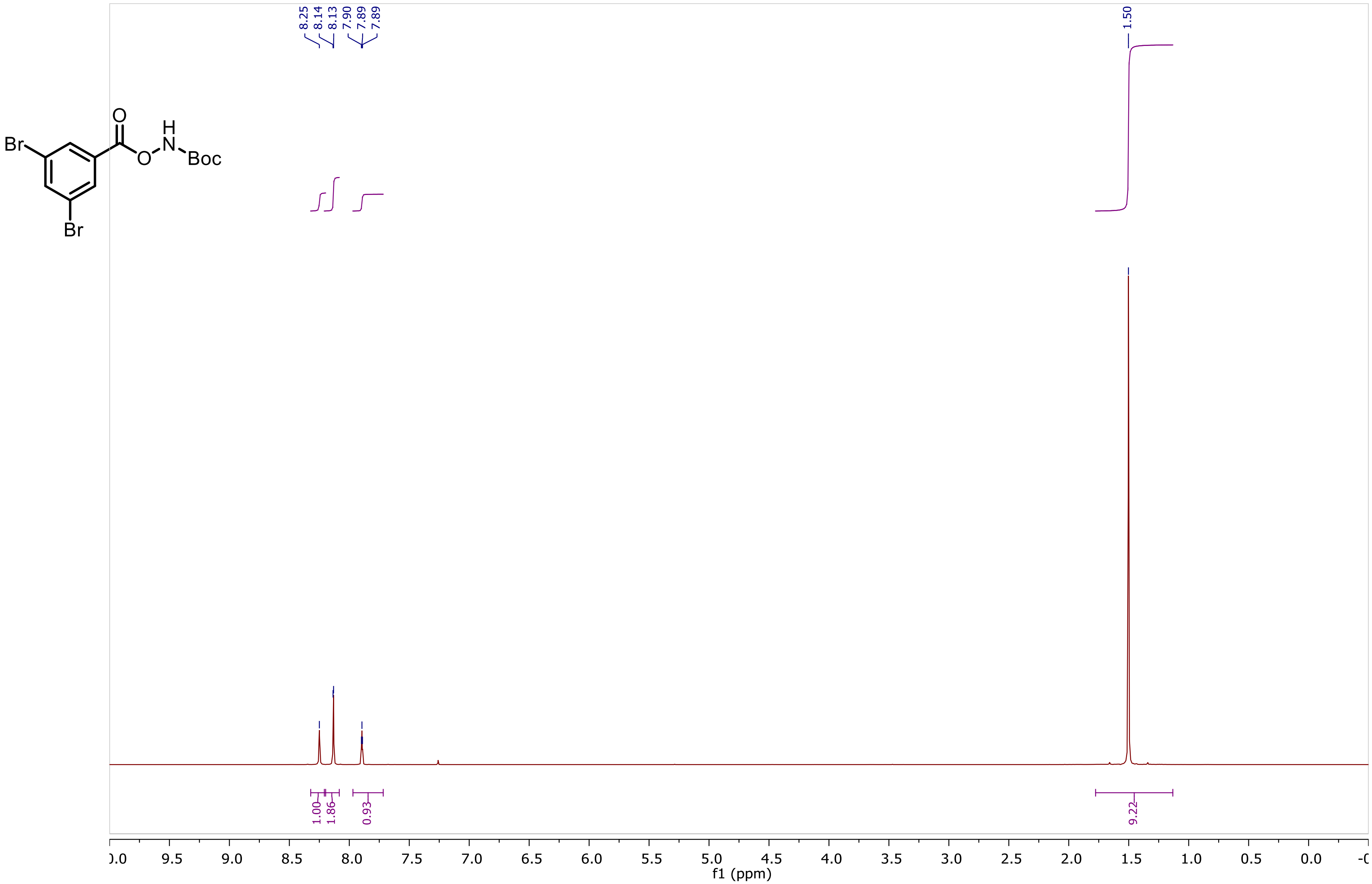

^{13}C NMR spectrum of *tert*-butyl (3,5-dibromobenzoyloxy)carbamate **1x** in $CDCl_3$

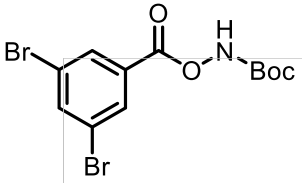

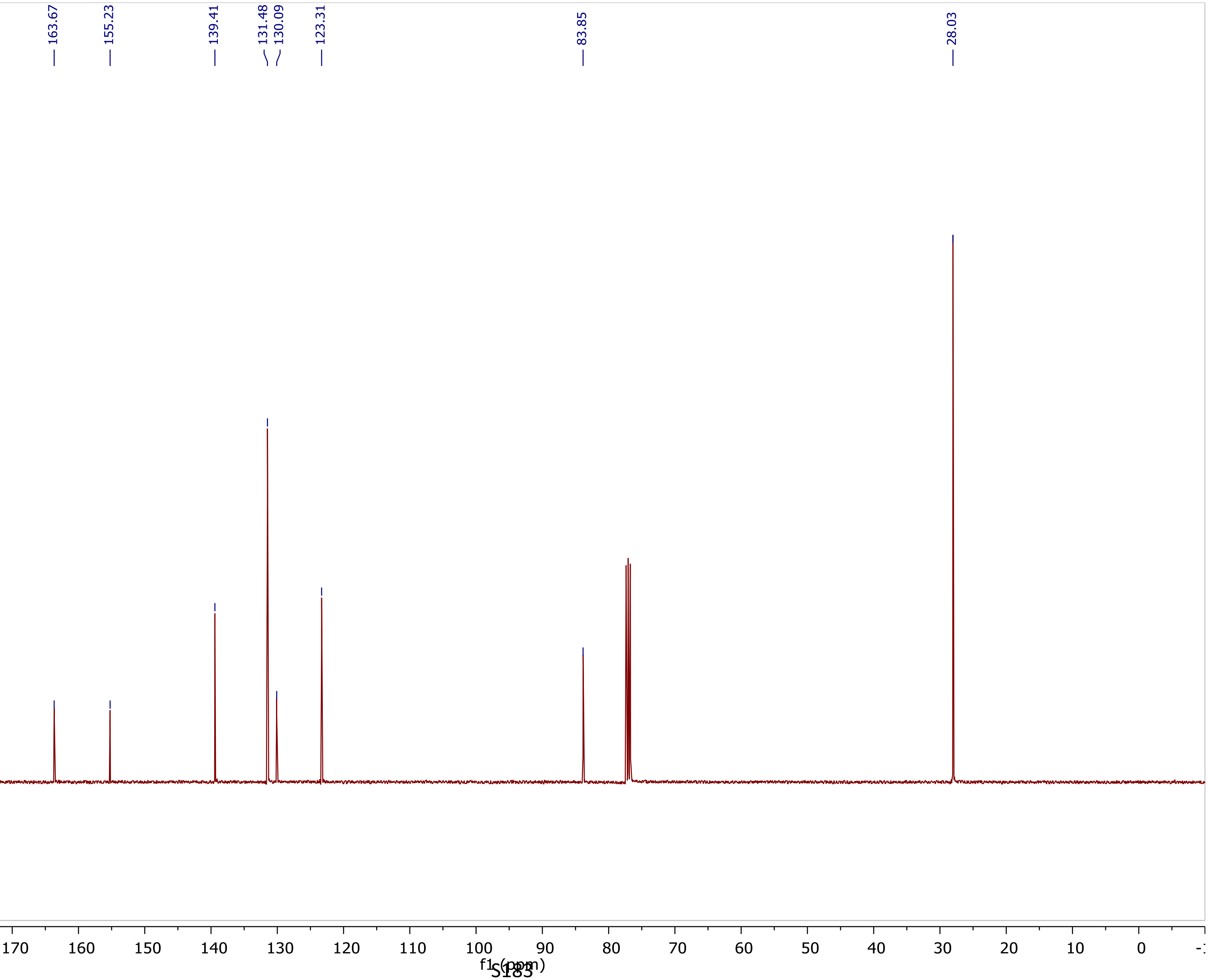

^{1}H NMR spectrum of *tert*-butyl (3,5-difluorobenzoyloxy)carbamate **1y** in $CDCl_3$

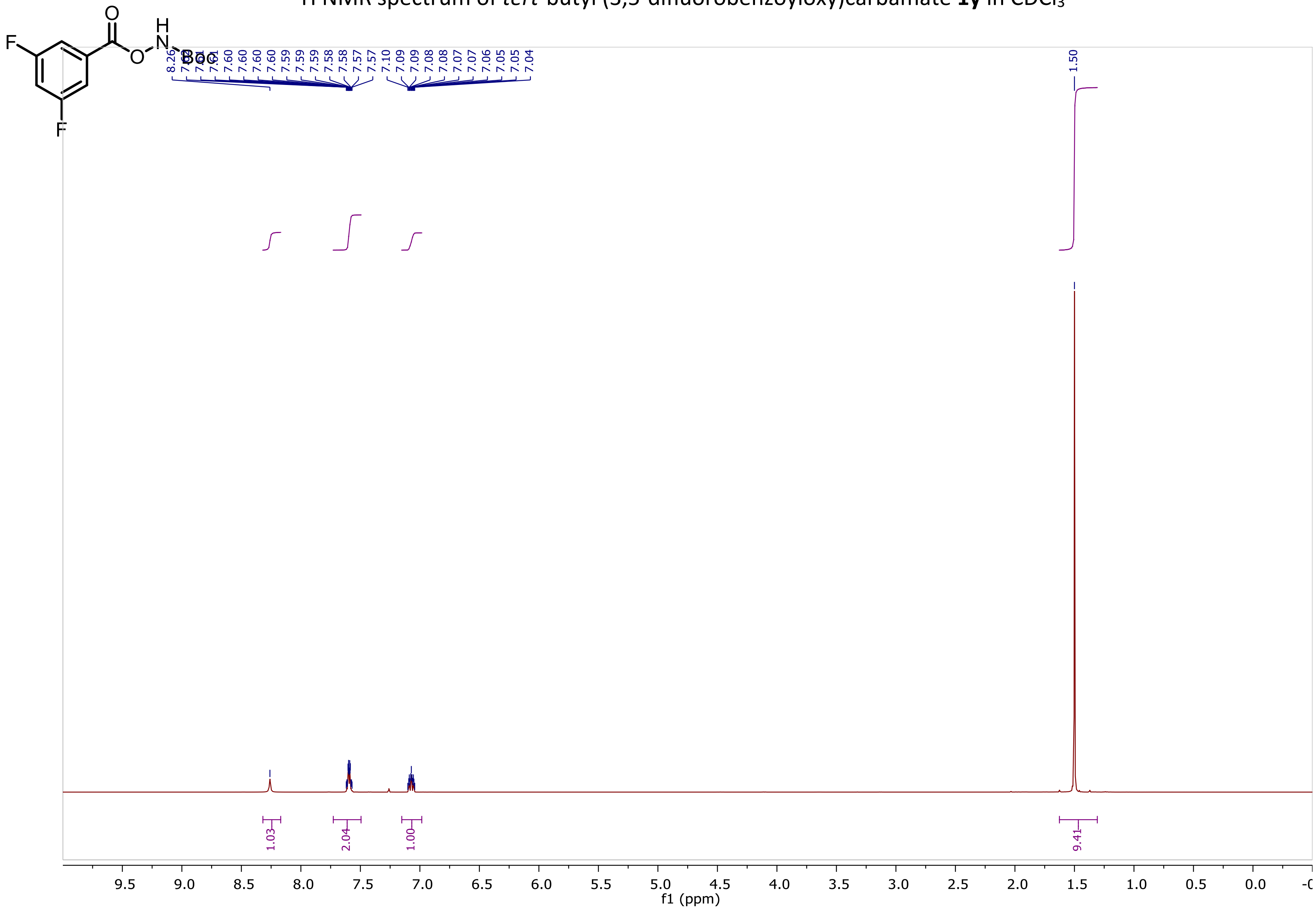

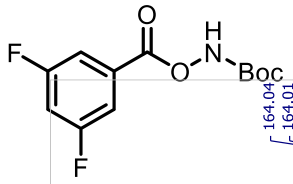

^{13}C NMR spectrum of *tert*-butyl (3,5-difluorobenzoyloxy)carbamate **1y** in $CDCl_3$

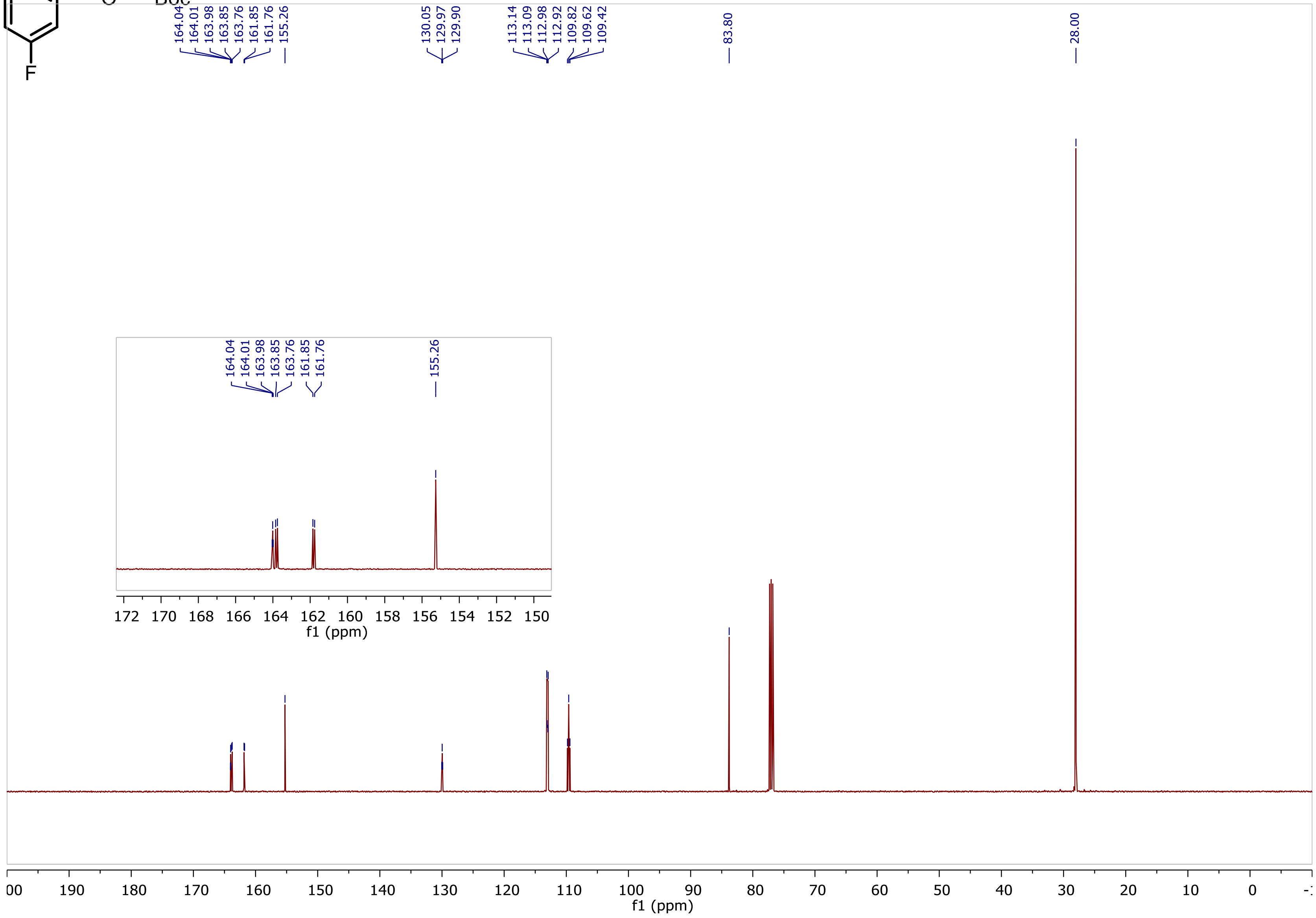

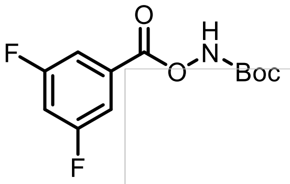

^{19}F NMR spectrum of *tert*-butyl (3,5-difluorobenzoyloxy)carbamate **1y** in $CDCl_3$

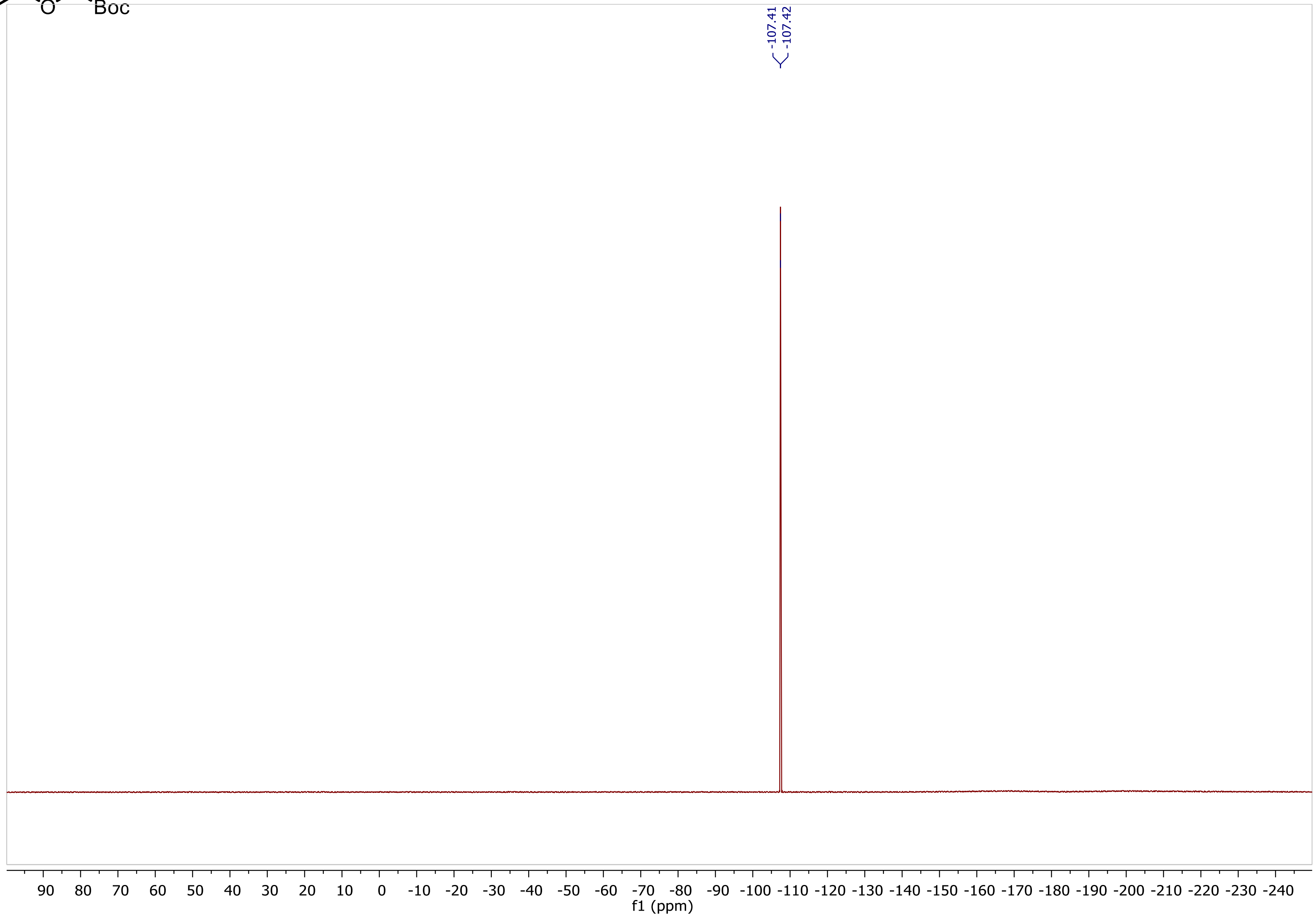

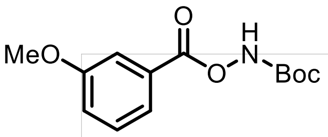

^{1}H NMR spectrum of *tert*-butyl (3-methoxybenzoyloxy)carbamate **1z** in $CDCl_3$

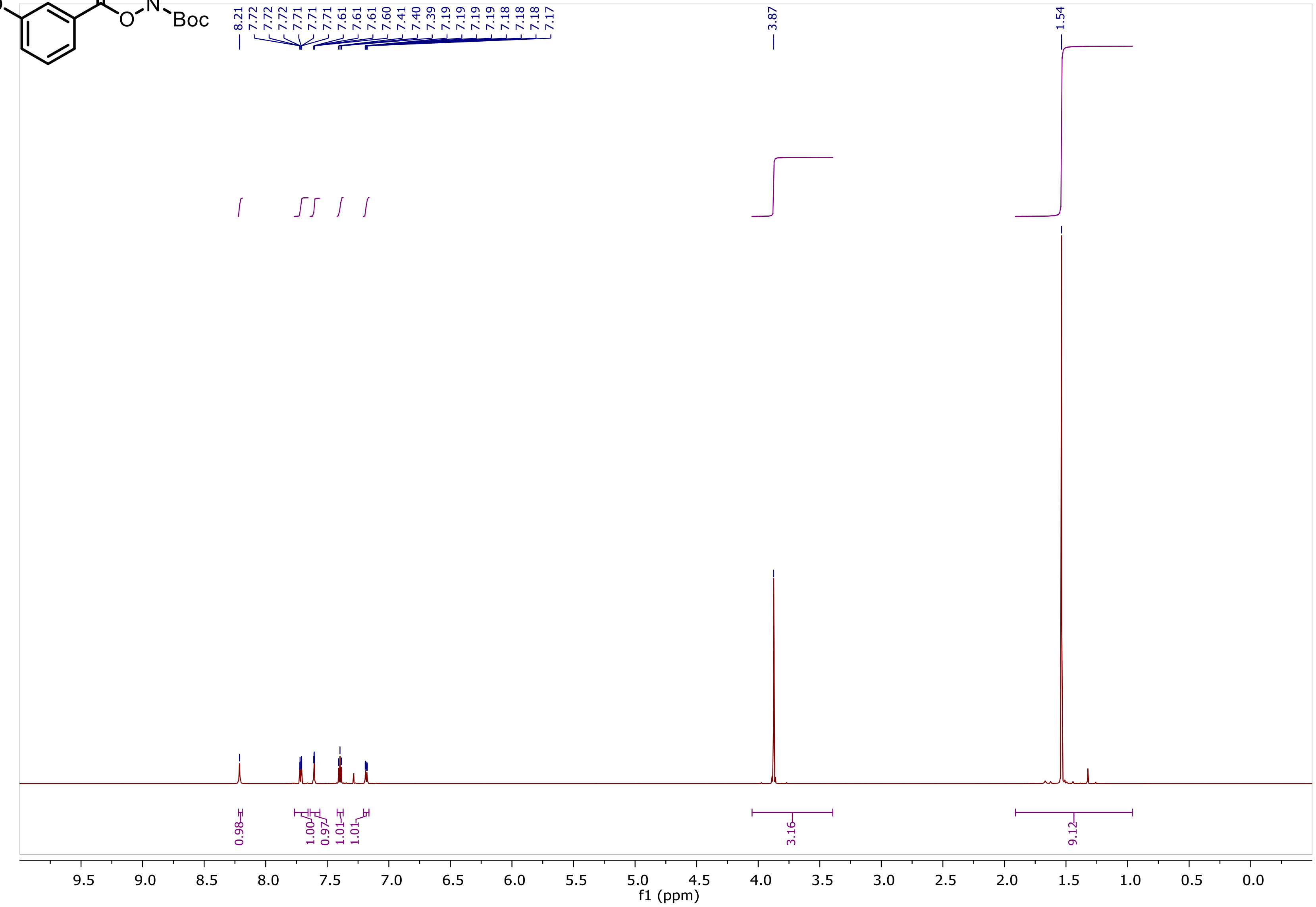

^{13}C NMR spectrum of *tert*-butyl (3-methoxybenzoyloxy)carbamate **1z** in $CDCl_3$

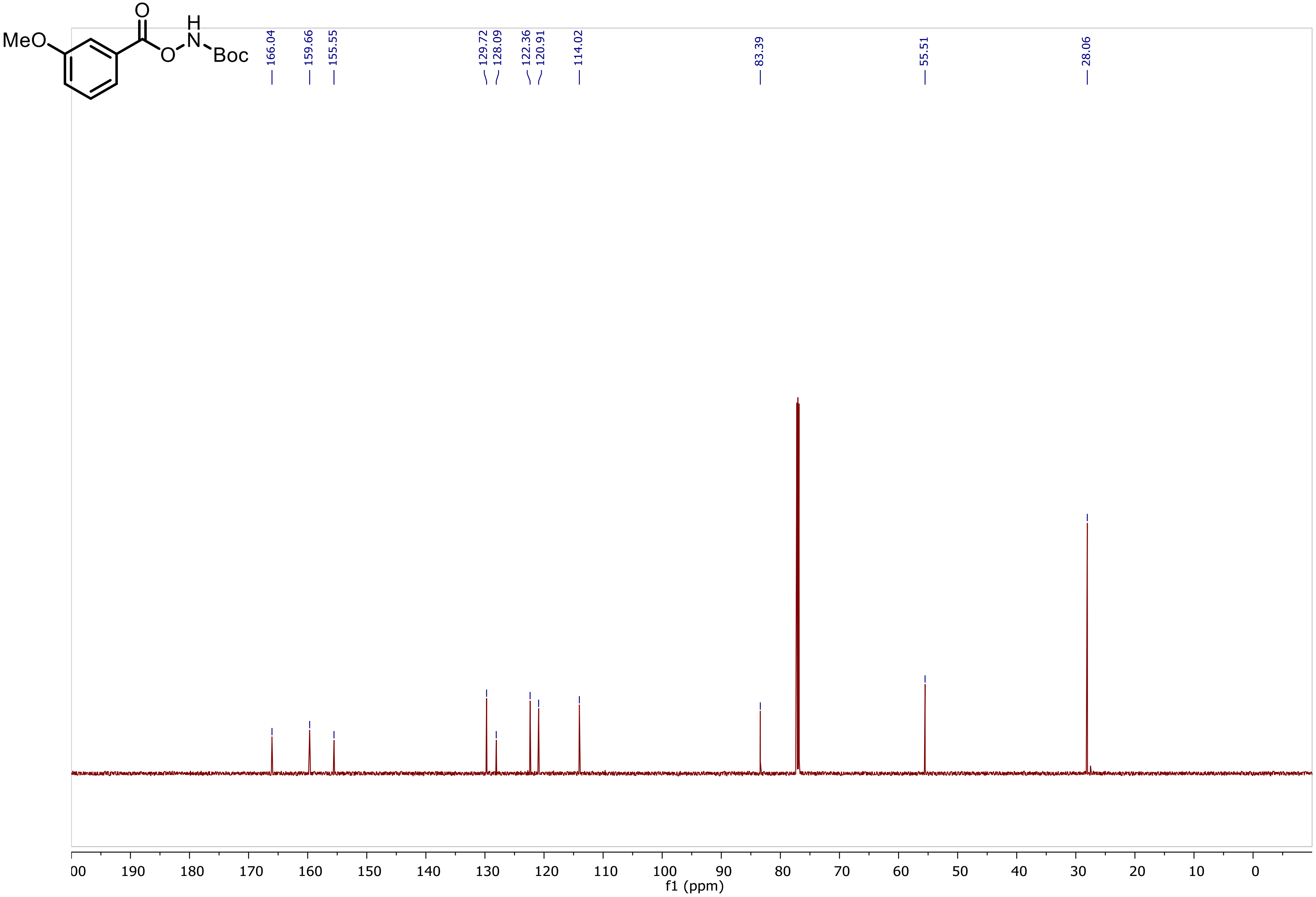

^{1}H NMR spectrum of *tert*-butyl (3-methoxy-2-methylbenzoyloxy)carbamate **1aa** in $CDCl_3$

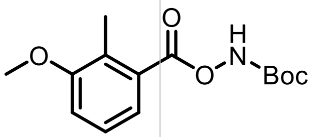

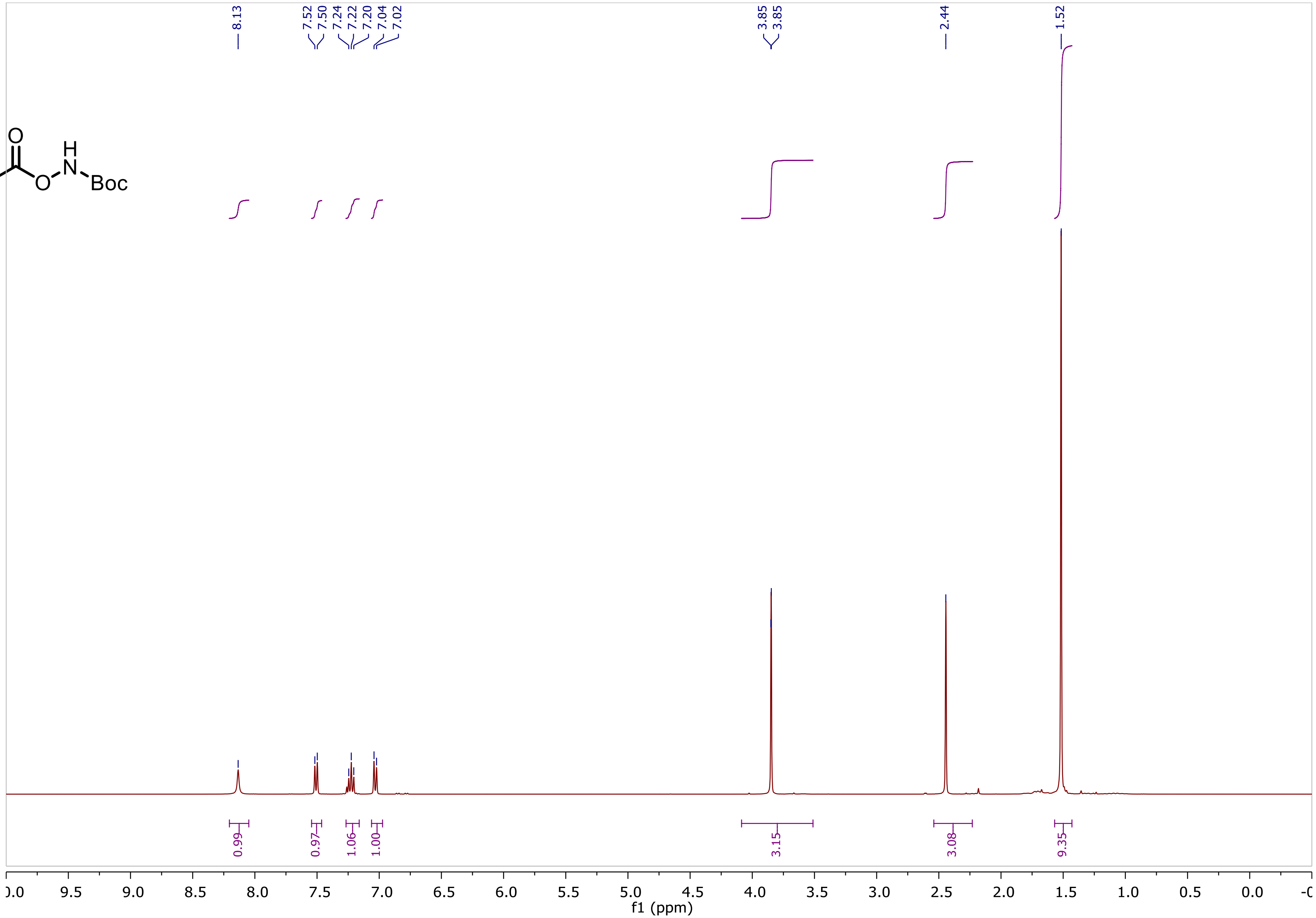

^{13}C NMR spectrum of *tert*-butyl (3-methoxy-2-methylbenzoyloxy)carbamate **1aa** in $CDCl_3$

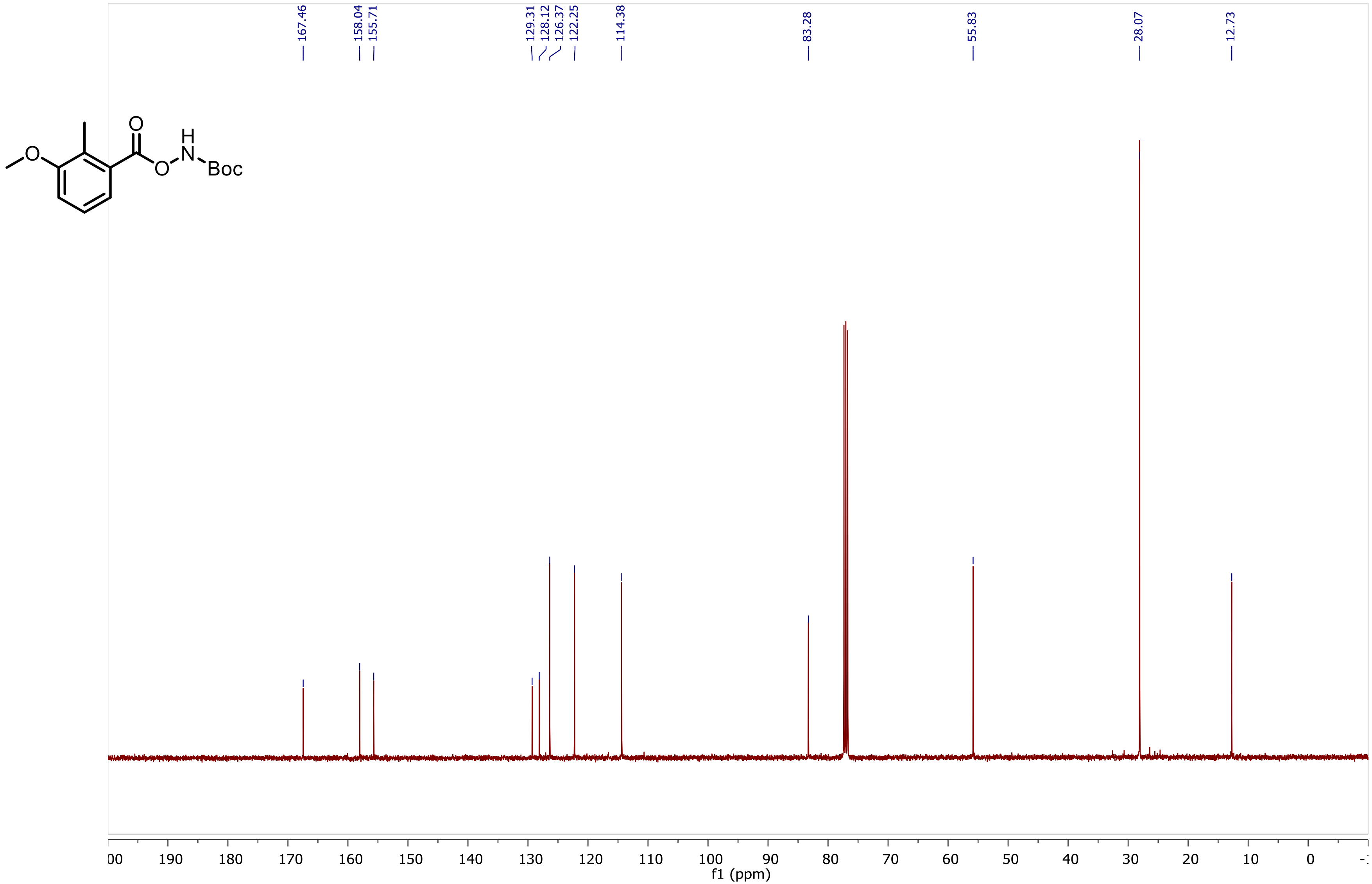

^{1}H NMR spectrum of *tert*-butyl (2-bromo-3-methoxybenzoyloxy)carbamate **1ab** in $CDCl_3$

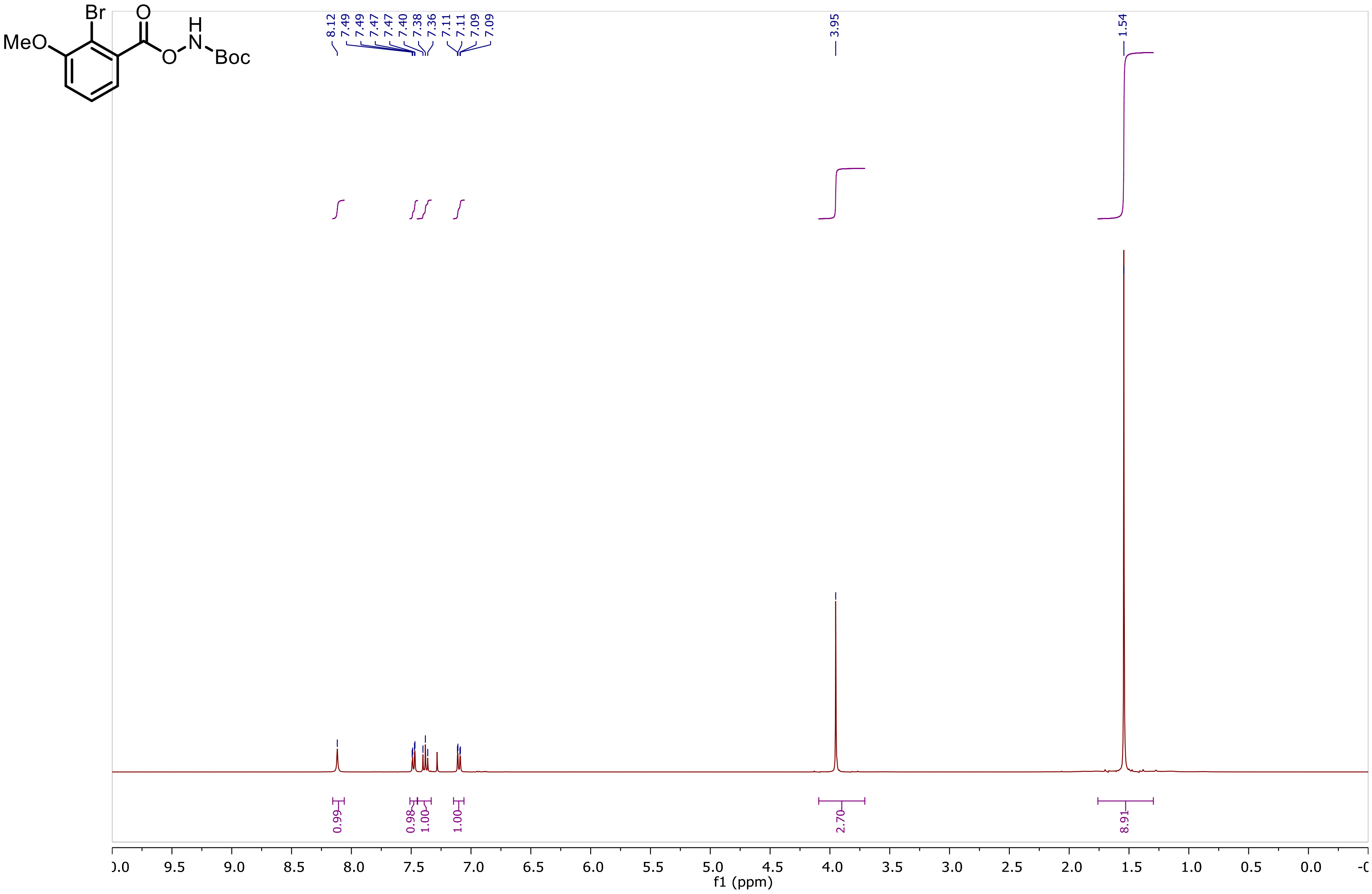

^{13}C NMR spectrum of *tert*-butyl (2-bromo-3-methoxybenzoyloxy)carbamate **1ab** in $CDCl_3$

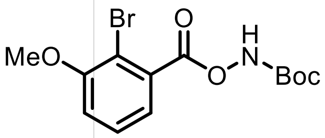

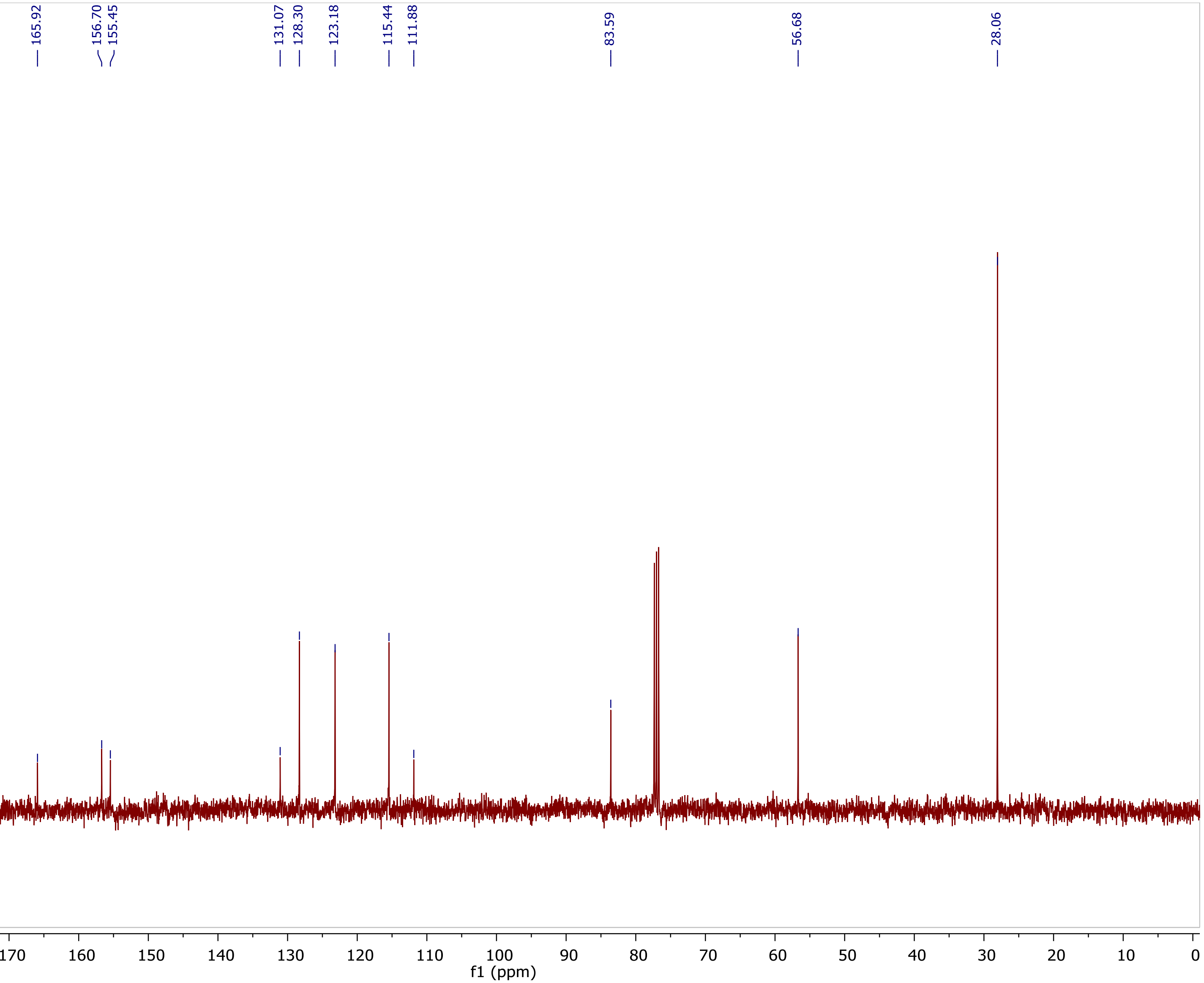

^{1}H NMR spectrum of *tert*-butyl (3,5-dimethoxybenzoyloxy)carbamate **1ac** in $CDCl_3$

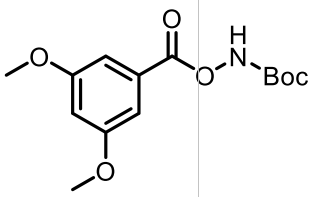

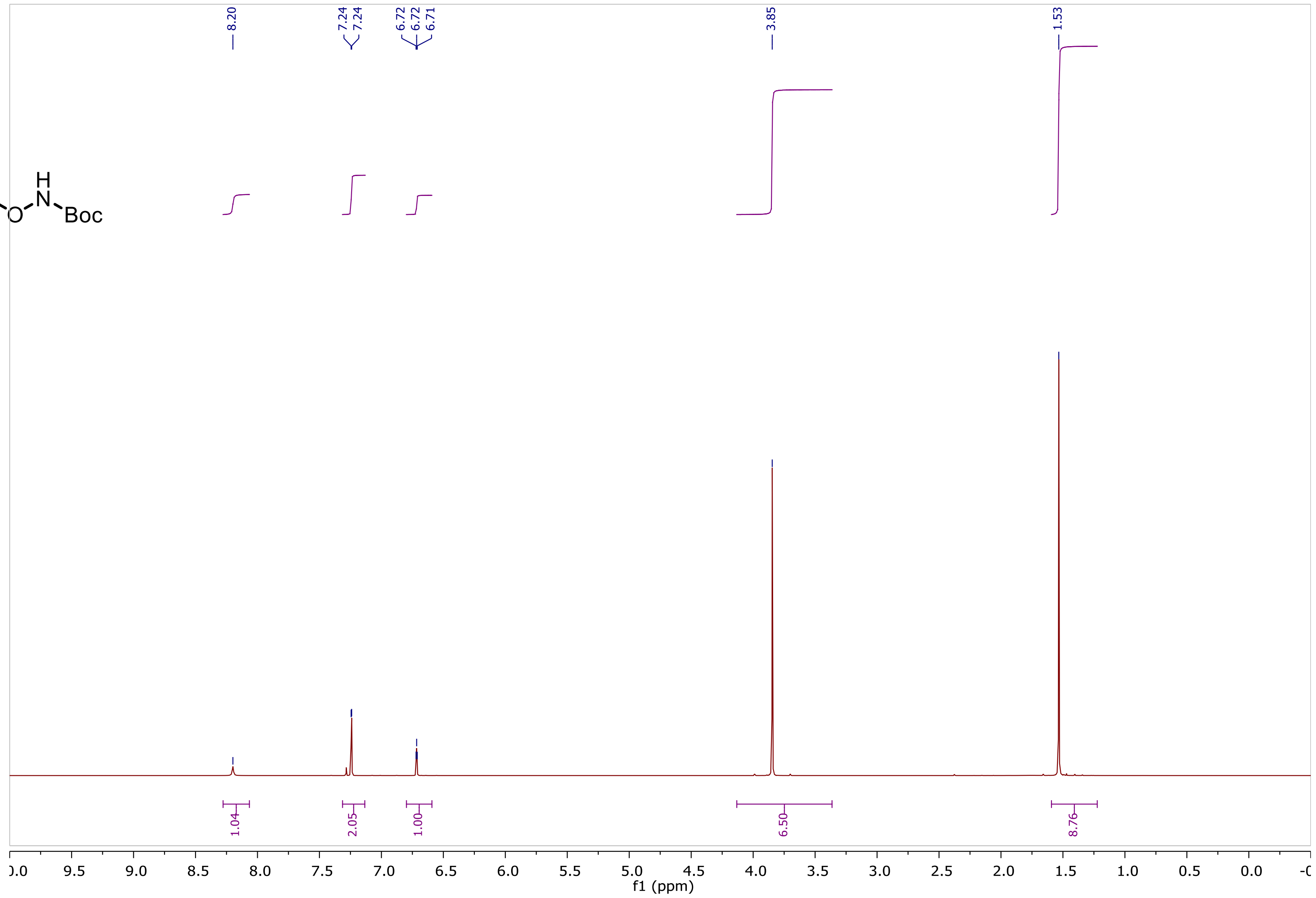

^{13}C NMR spectrum of *tert*-butyl (3,5-dimethoxybenzoyloxy)carbamate **1ac** in $CDCl_3$

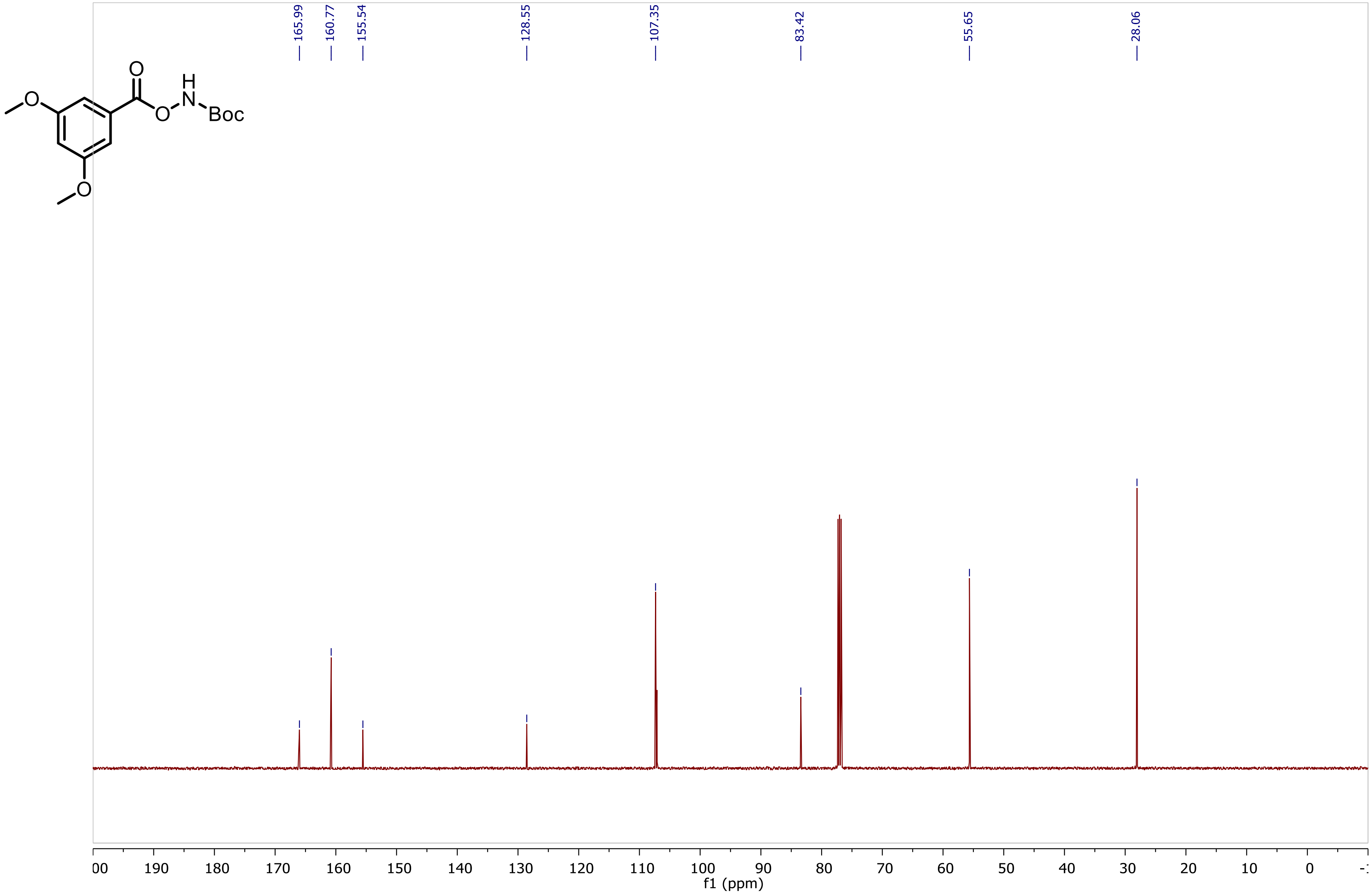

^{1}H NMR spectrum of *tert*-butyl ((4-(chloromethyl)benzoyloxy))carbamate **1ag** in $CDCl_3$

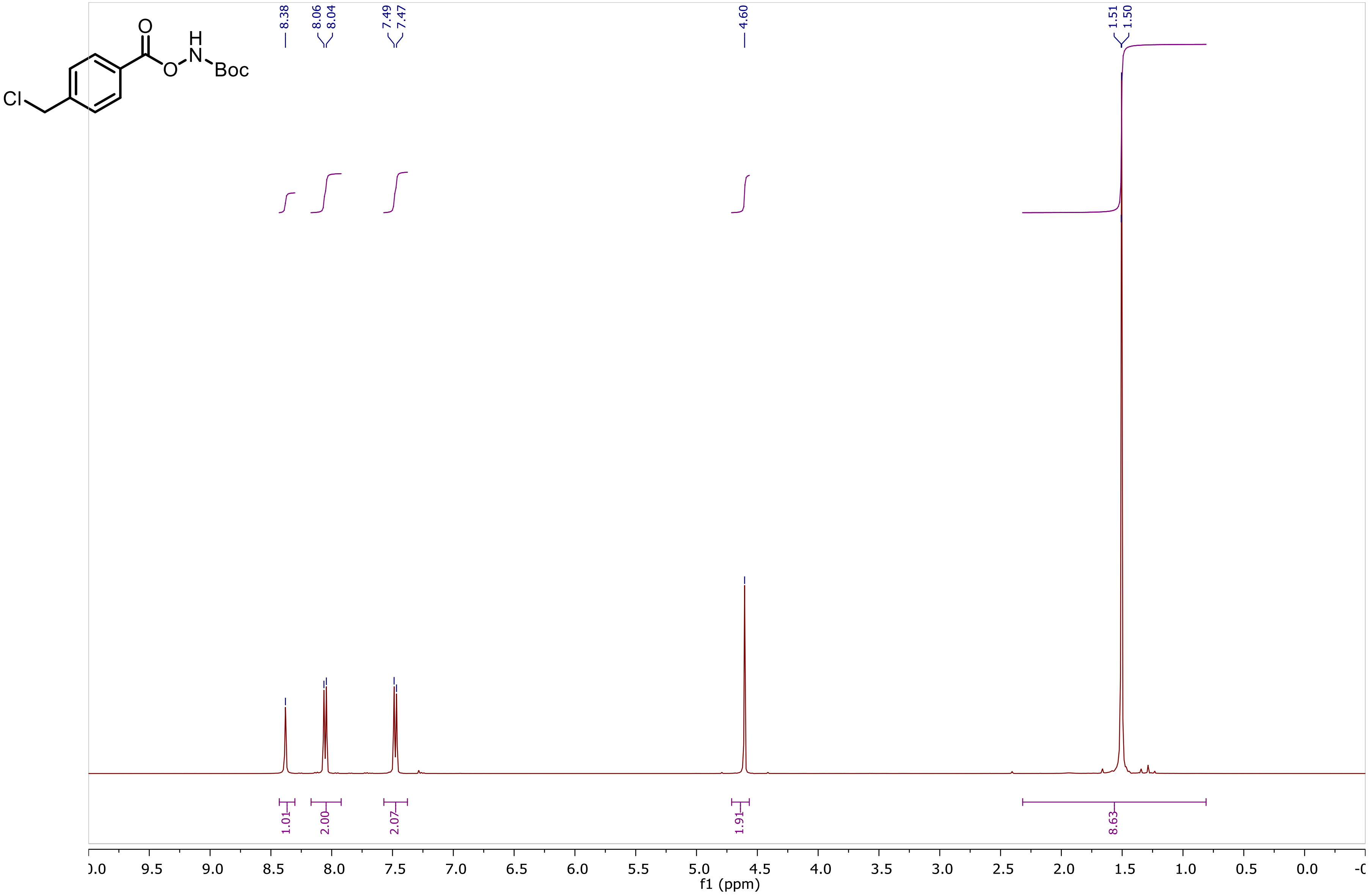

^{13}C NMR spectrum of *tert*-butyl ((4-(chloromethyl)benzoyloxy))carbamate **1ag** in $CDCl_3$

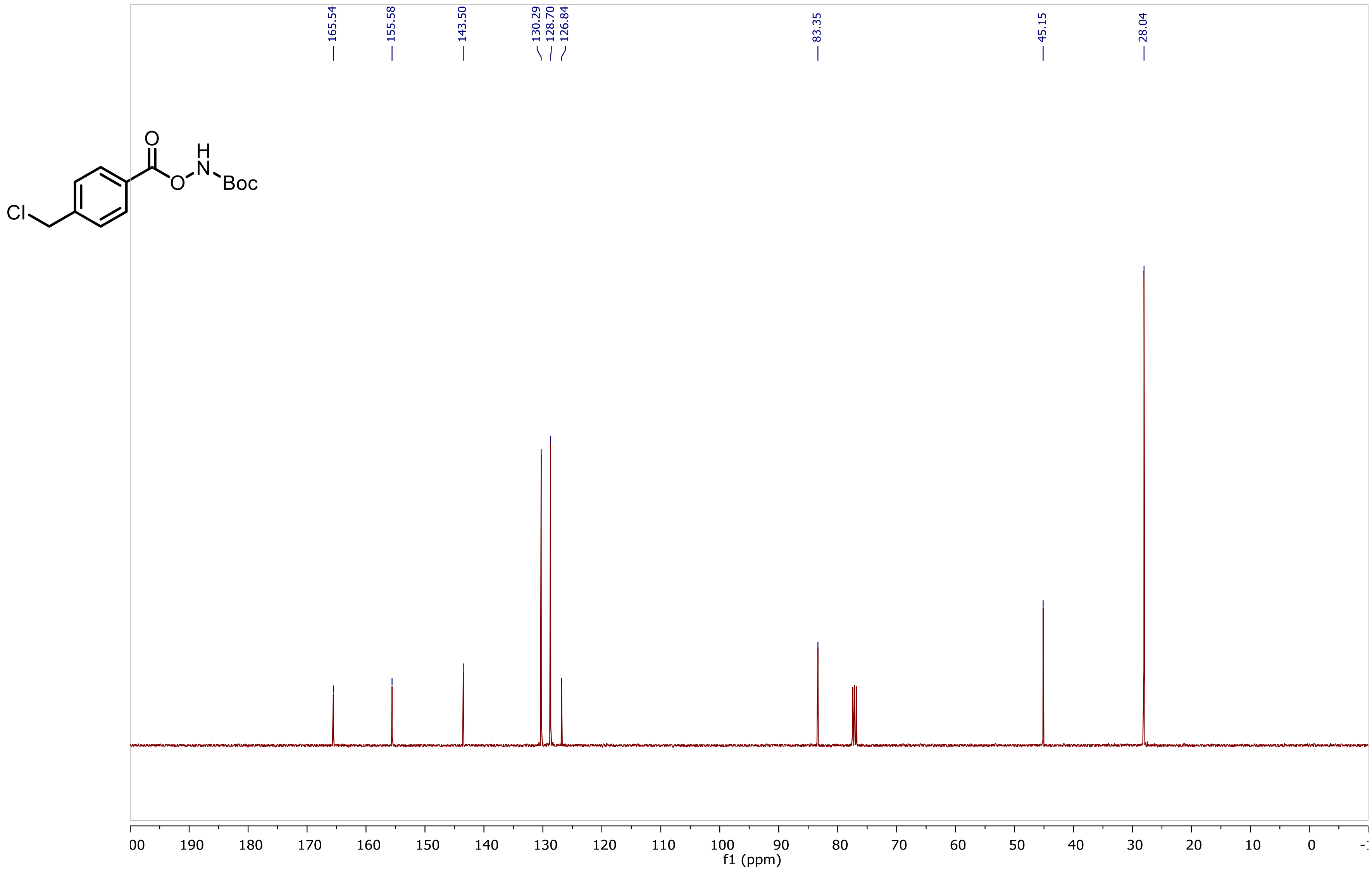

^{1}H NMR spectrum of *tert*-butyl ((4-(bromomethyl)benzoyloxy))carbamate **1ah** in $CDCl_3$

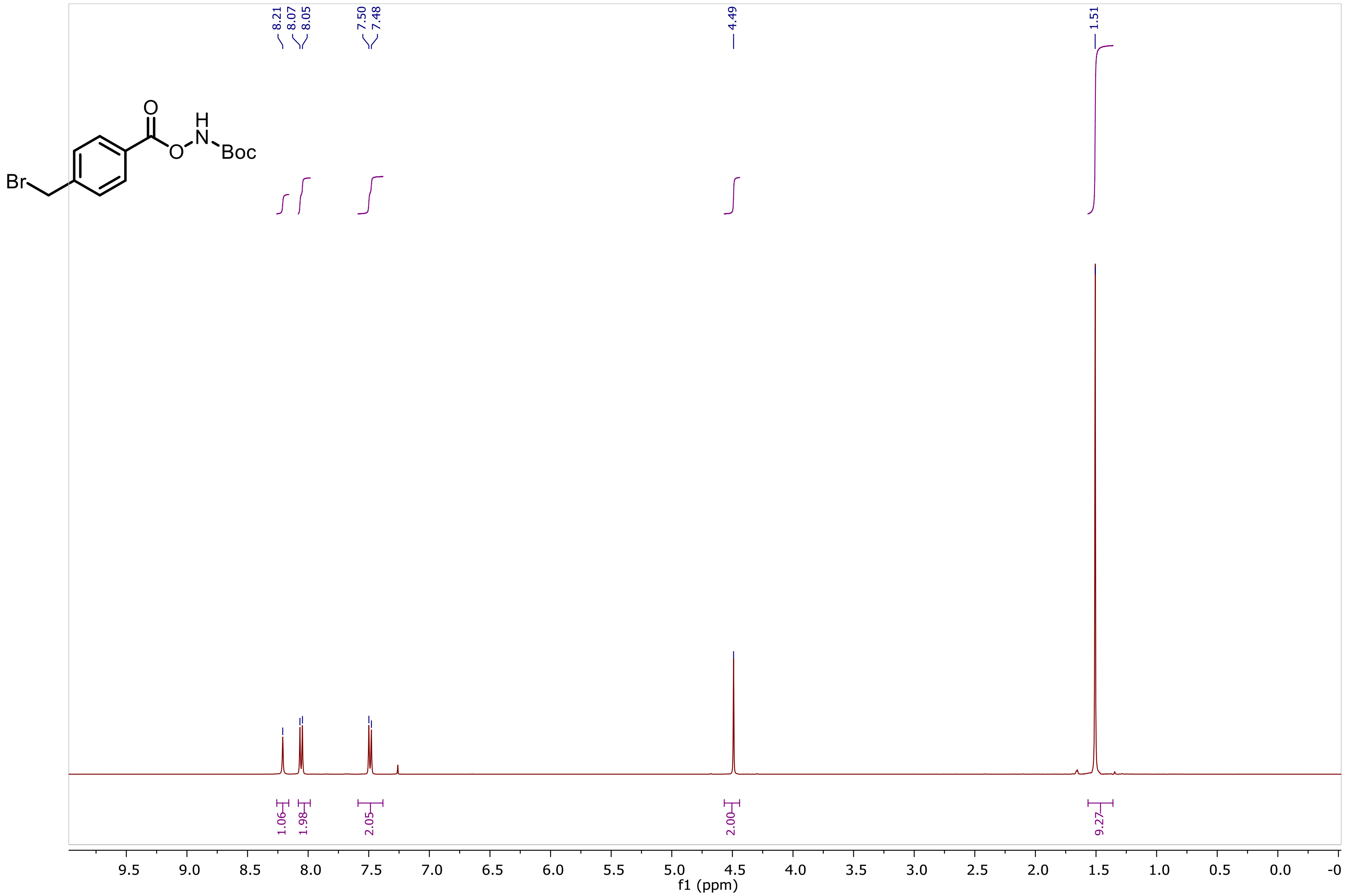

^{1}H NMR spectrum of *tert*-butyl ((4-(bromomethyl)benzoyloxy))carbamate **1ah** in $CDCl_3$

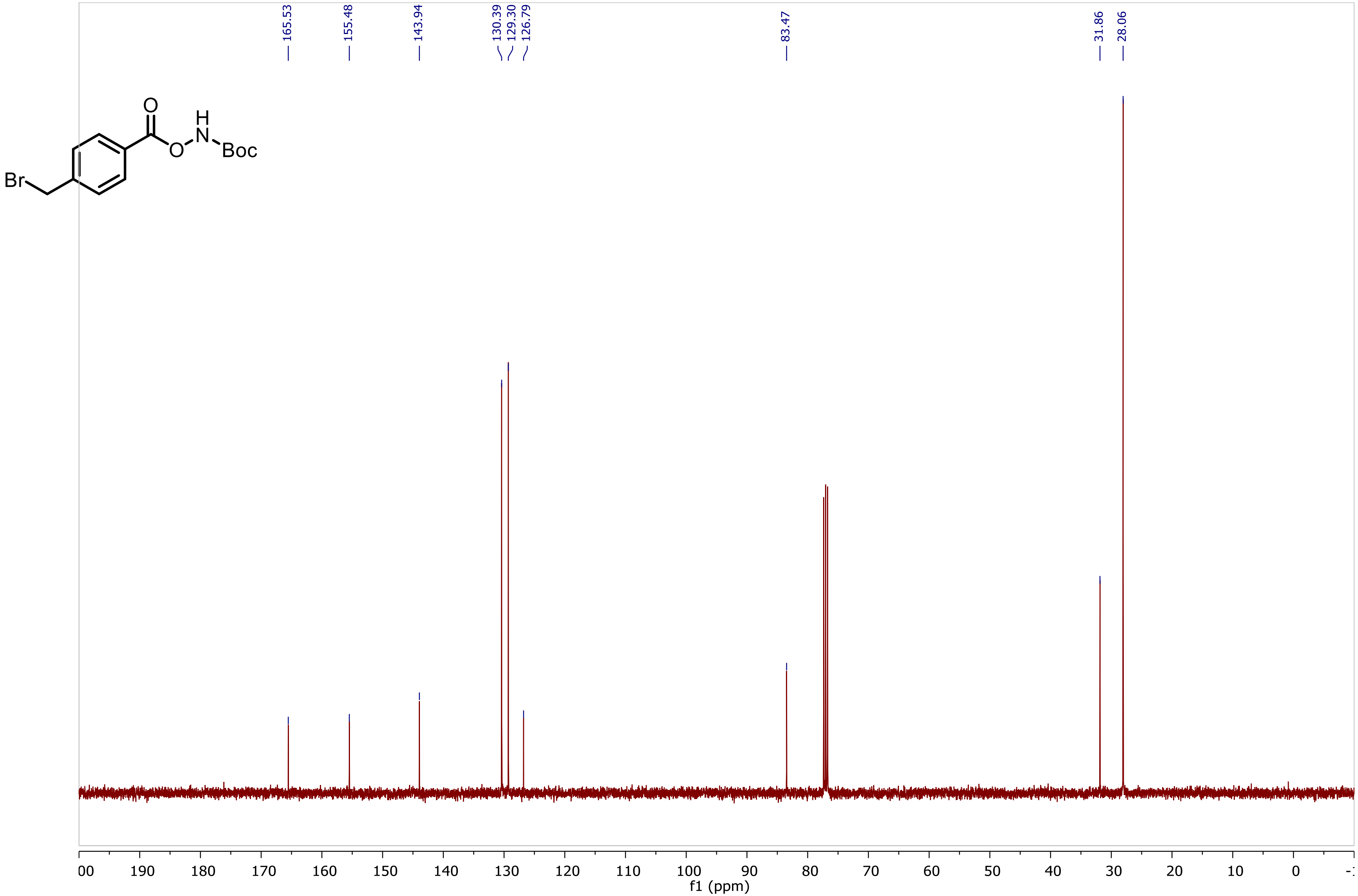

^{1}H NMR spectrum of *tert*-butyl ((2-(trifluoromethyl)benzoyl)oxy)carbamate **1ad** in $CDCl_3$

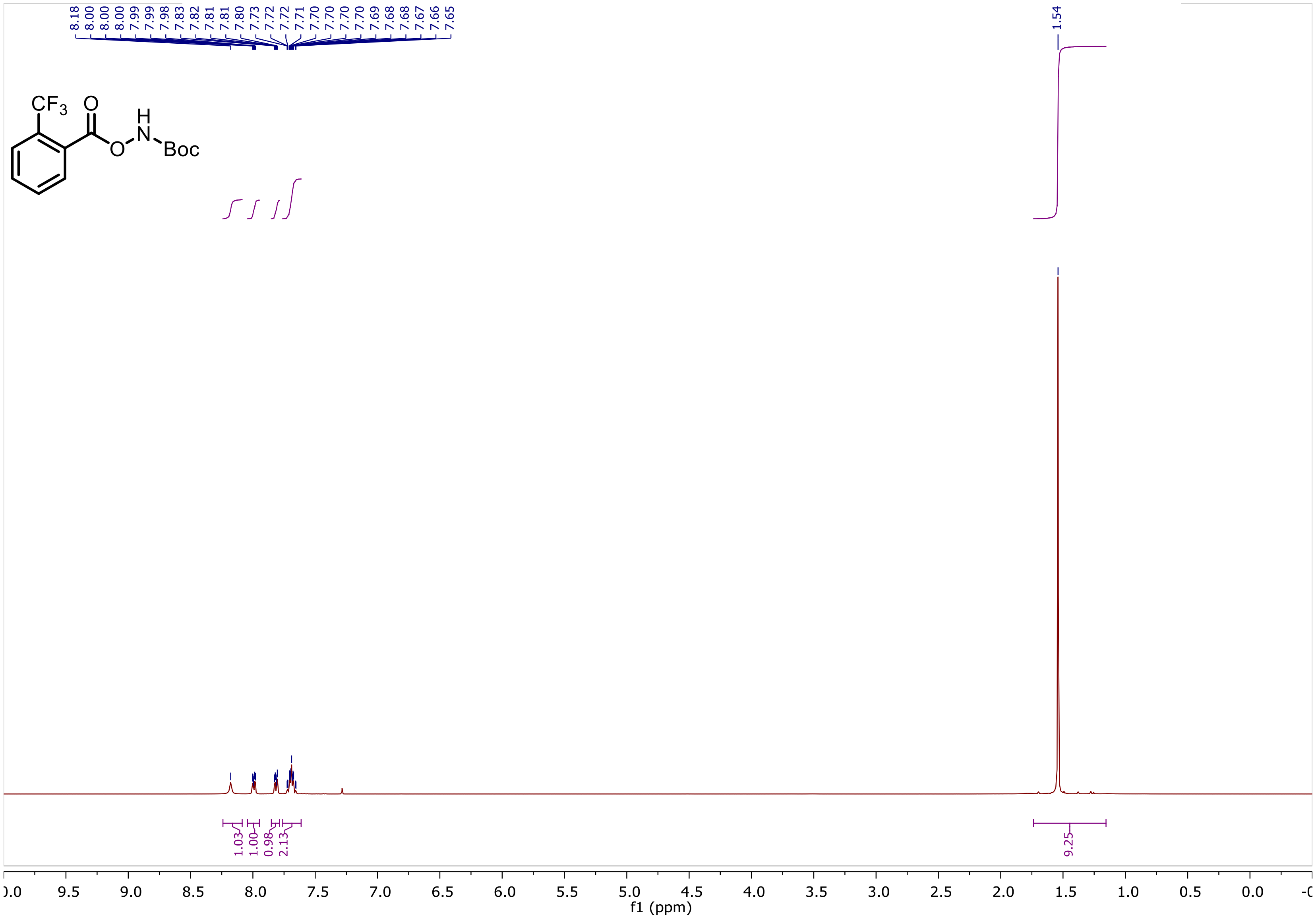

^{13}C NMR spectrum of *tert*-butyl ((2-(trifluoromethyl)benzoyl)oxy)carbamate **1ad** in $CDCl_3$

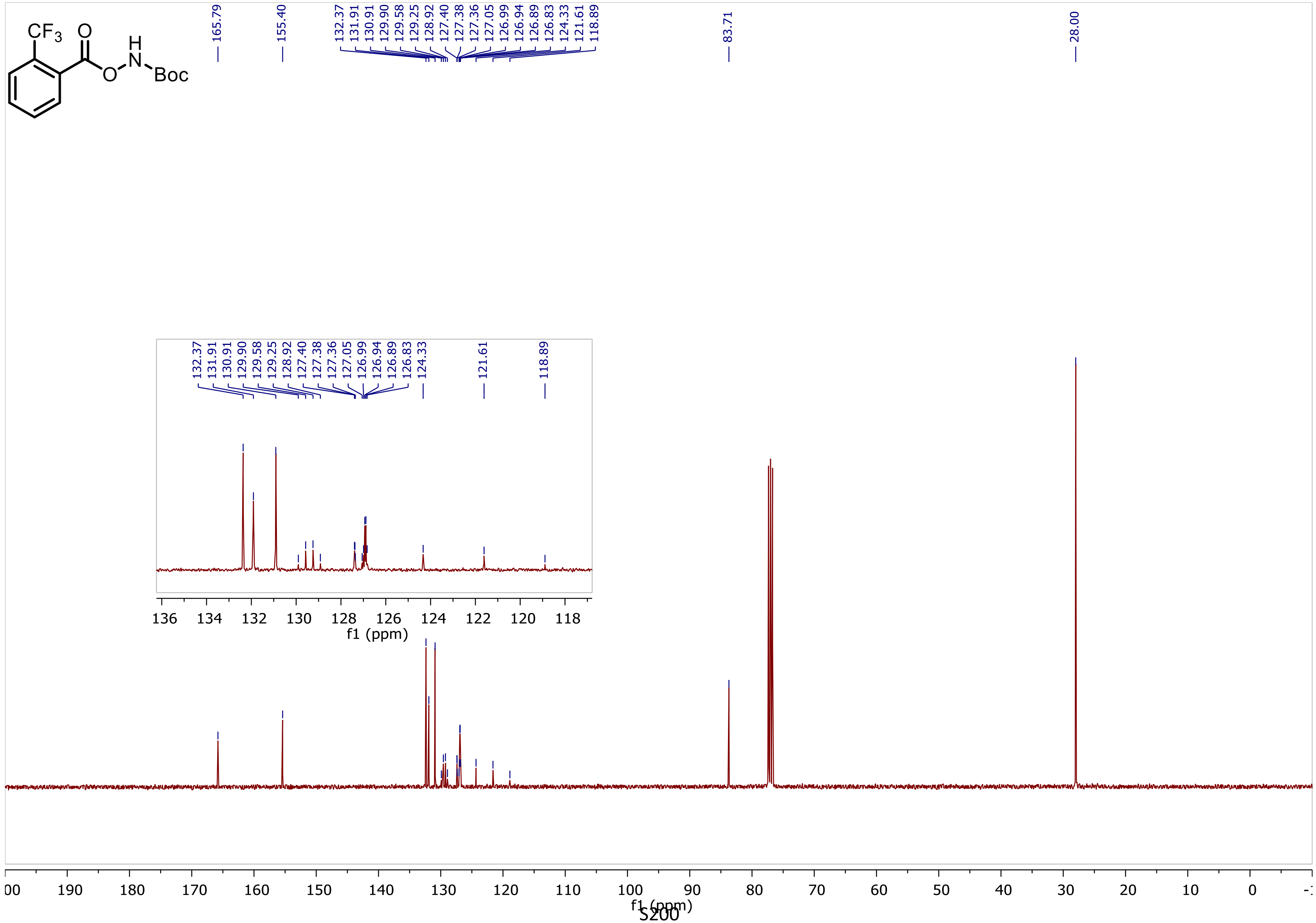

^{19}F NMR spectrum of *tert*-butyl ((2-(trifluoromethyl)benzoyl)oxy)carbamate **1ad** in $CDCl_3$

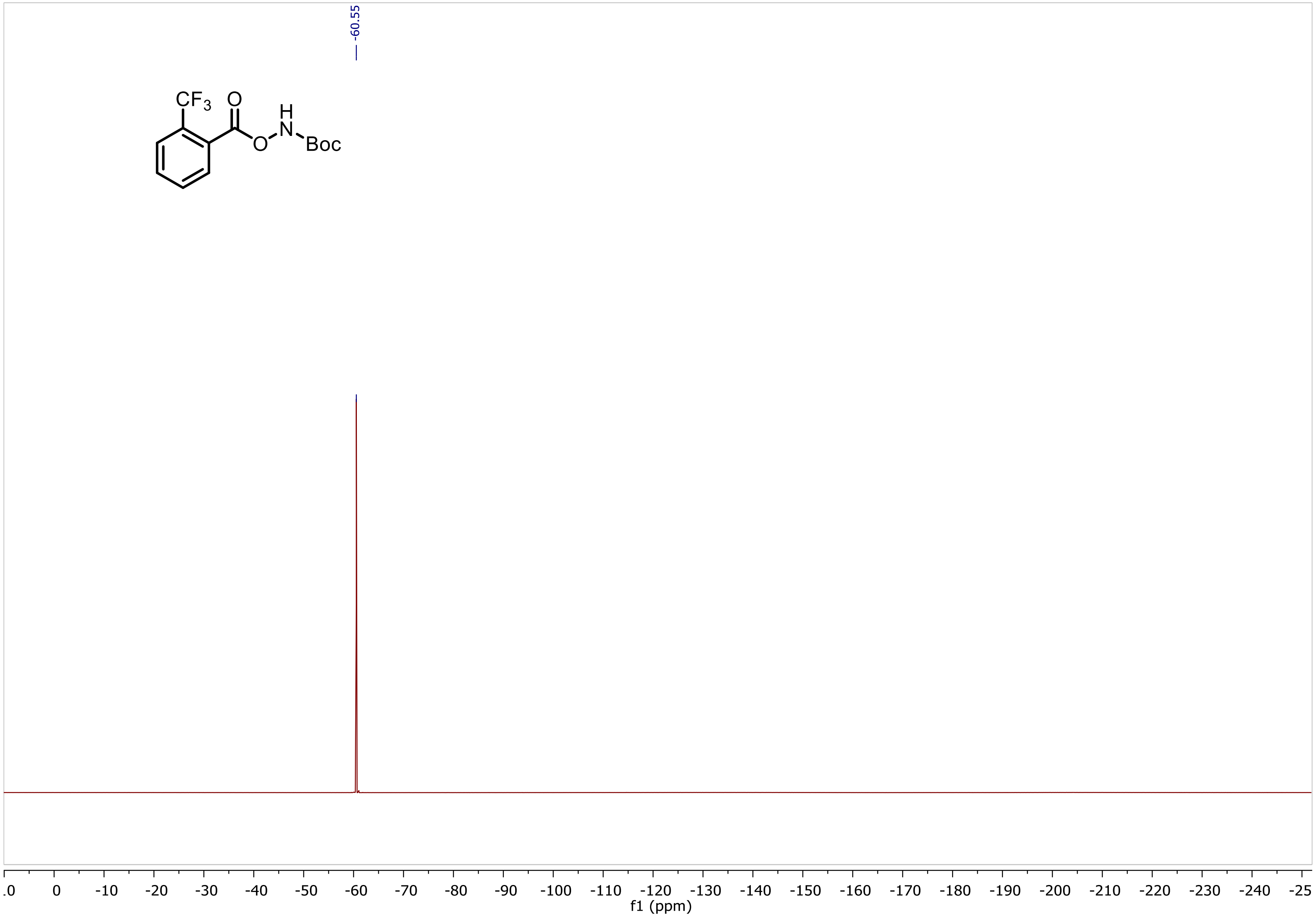

^{1}H NMR spectrum of *tert*-butyl ((4-(trifluoromethyl)benzoyl)oxy)carbamate **1ae** in $CDCl_3$

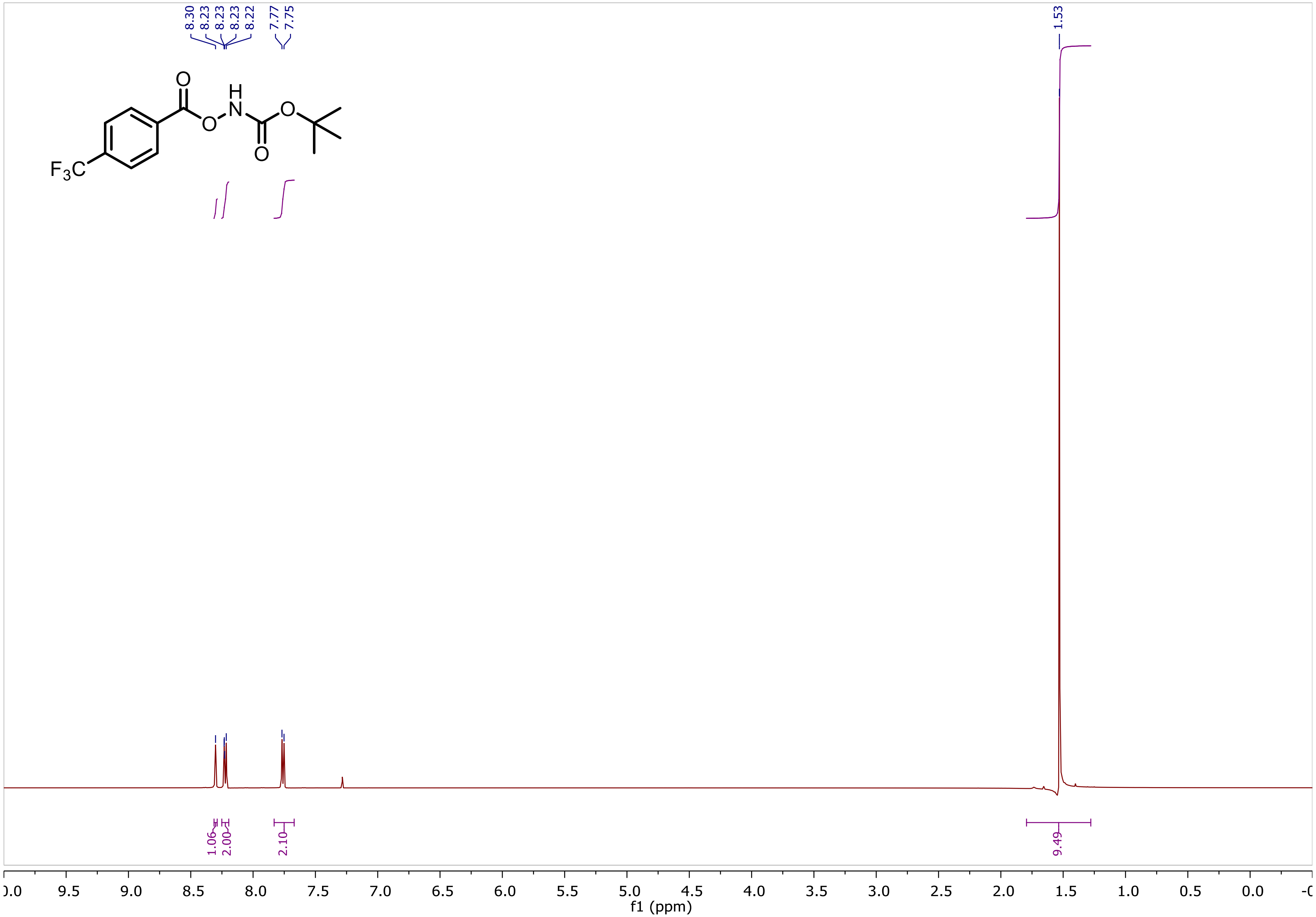

^{13}C NMR spectrum of *tert*-butyl ((4-(trifluoromethyl)benzoyl)oxy)carbamate **1ae** in $CDCl_3$

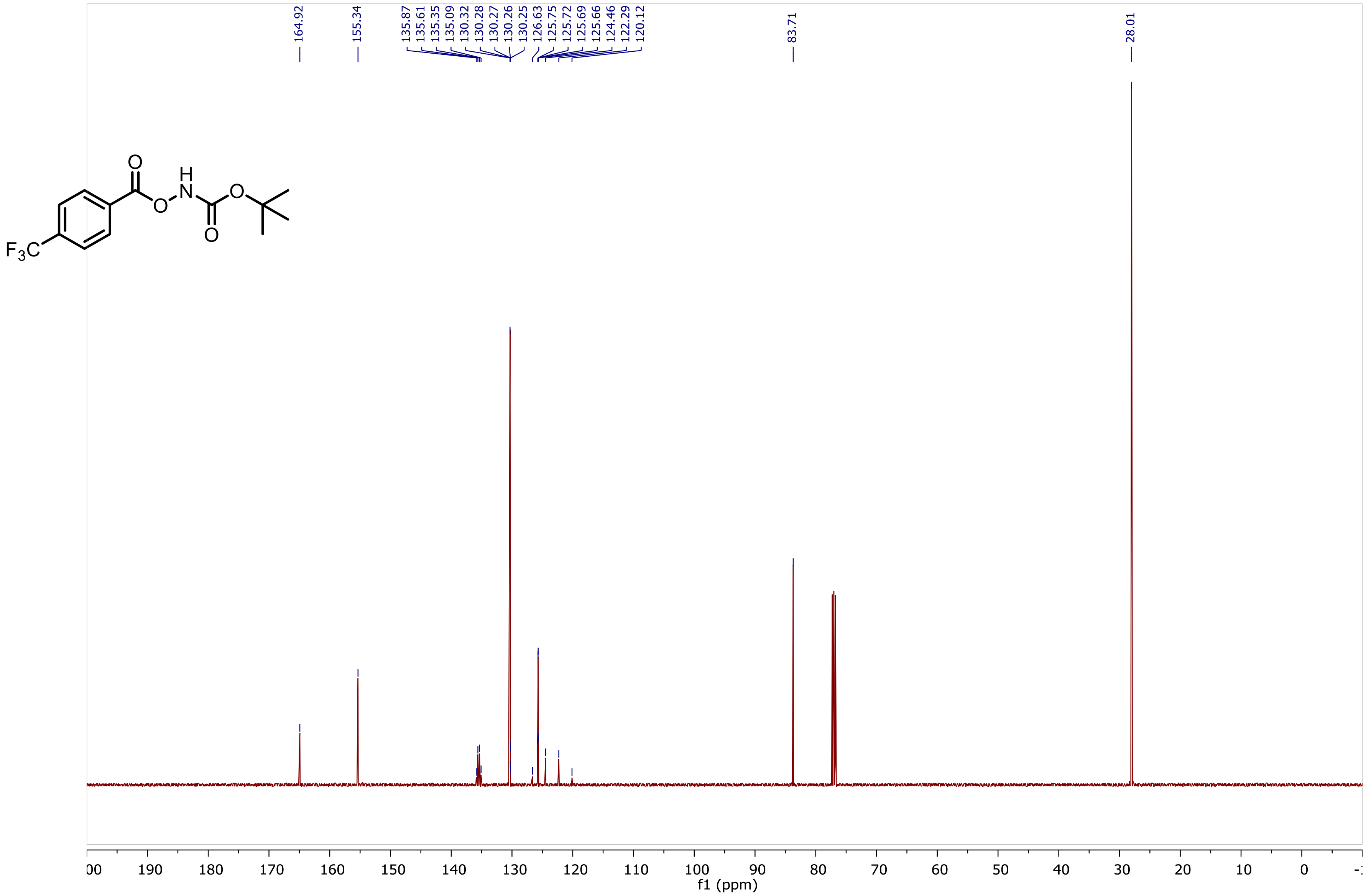

^{19}F NMR spectrum of *tert*-butyl ((4-(trifluoromethyl)benzoyl)oxy)carbamate **1ae** in $CDCl_3$

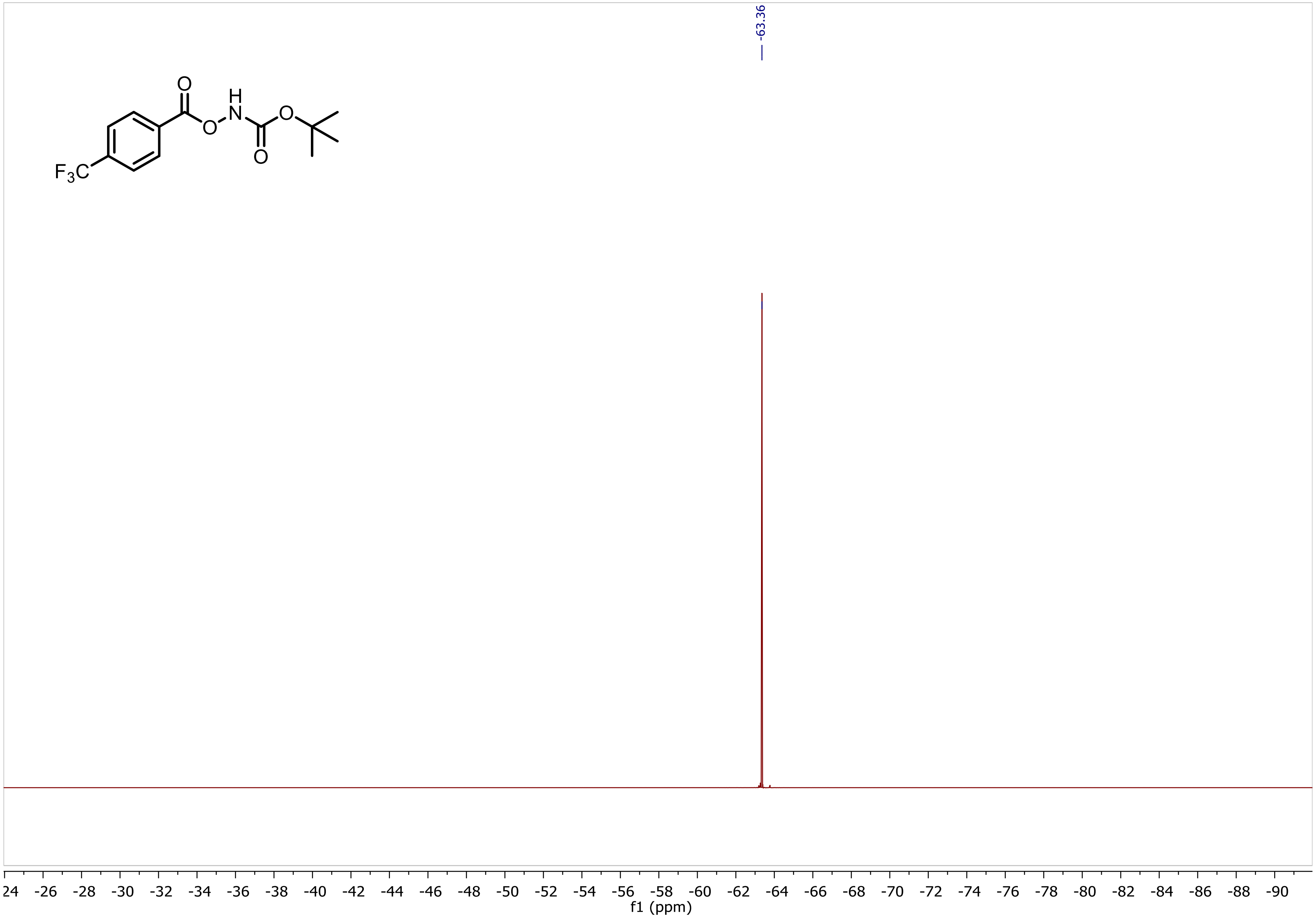

^{1}H NMR spectrum of *tert*-butyl ((4-(difluoromethoxy)benzoyl)oxy)carbamate **1af** in $CDCl_3$

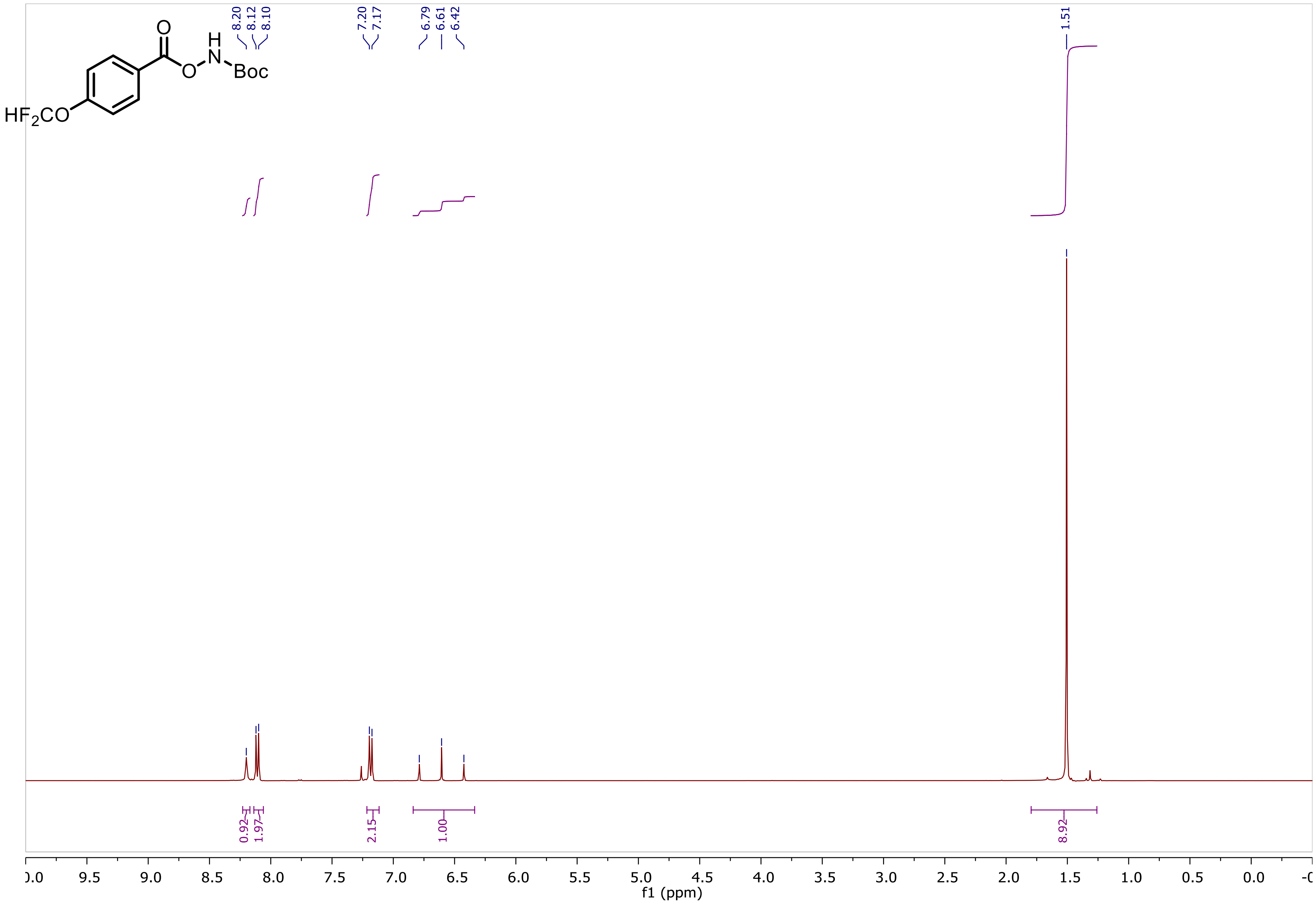

^{13}C NMR spectrum of *tert*-butyl ((4-(difluoromethoxy)benzoyl)oxy)carbamate **1af** in $CDCl_3$

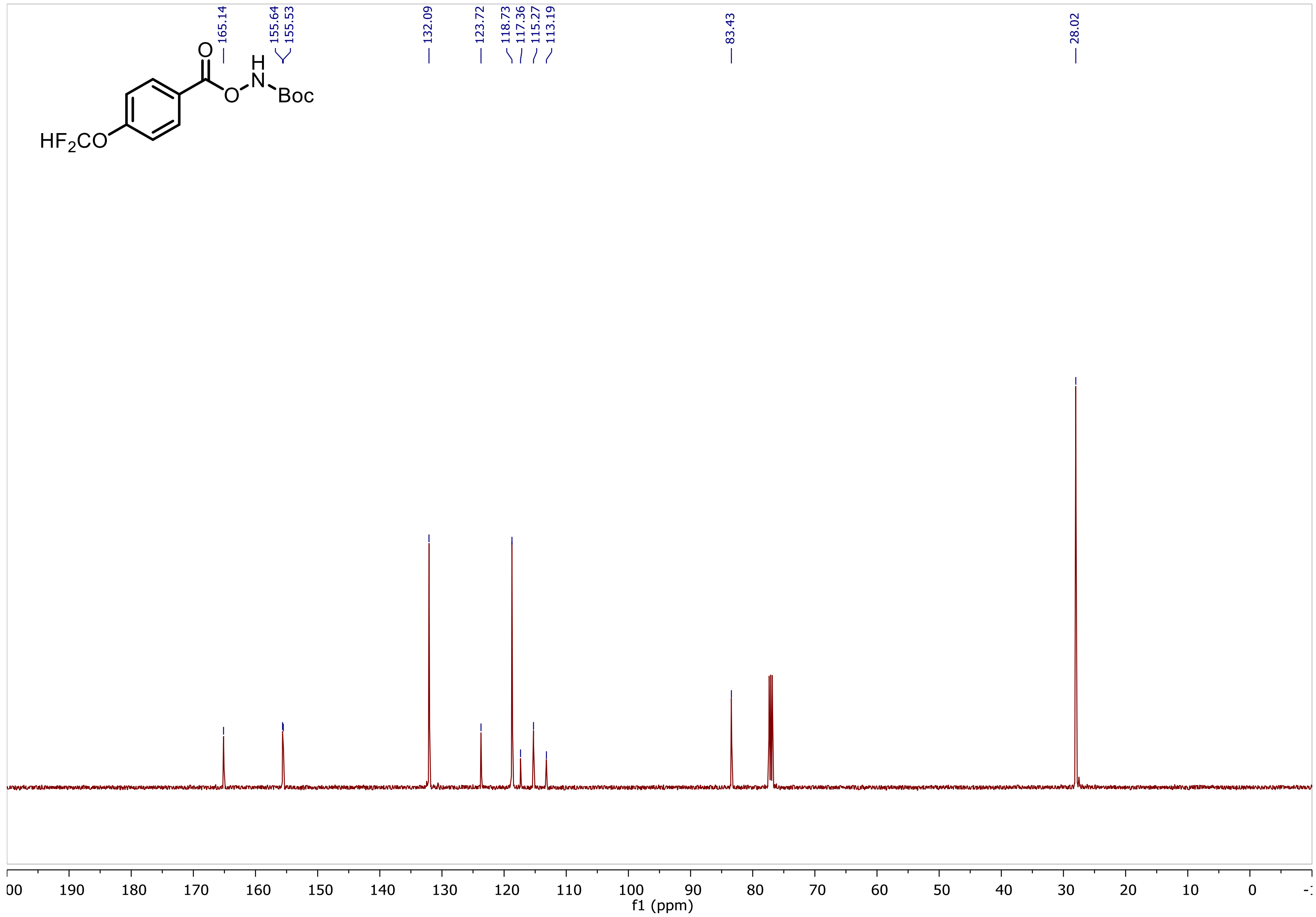

^{19}F NMR spectrum of *tert*-butyl ((4-(difluoromethoxy)benzoyl)oxy)carbamate **1af** in $CDCl_3$

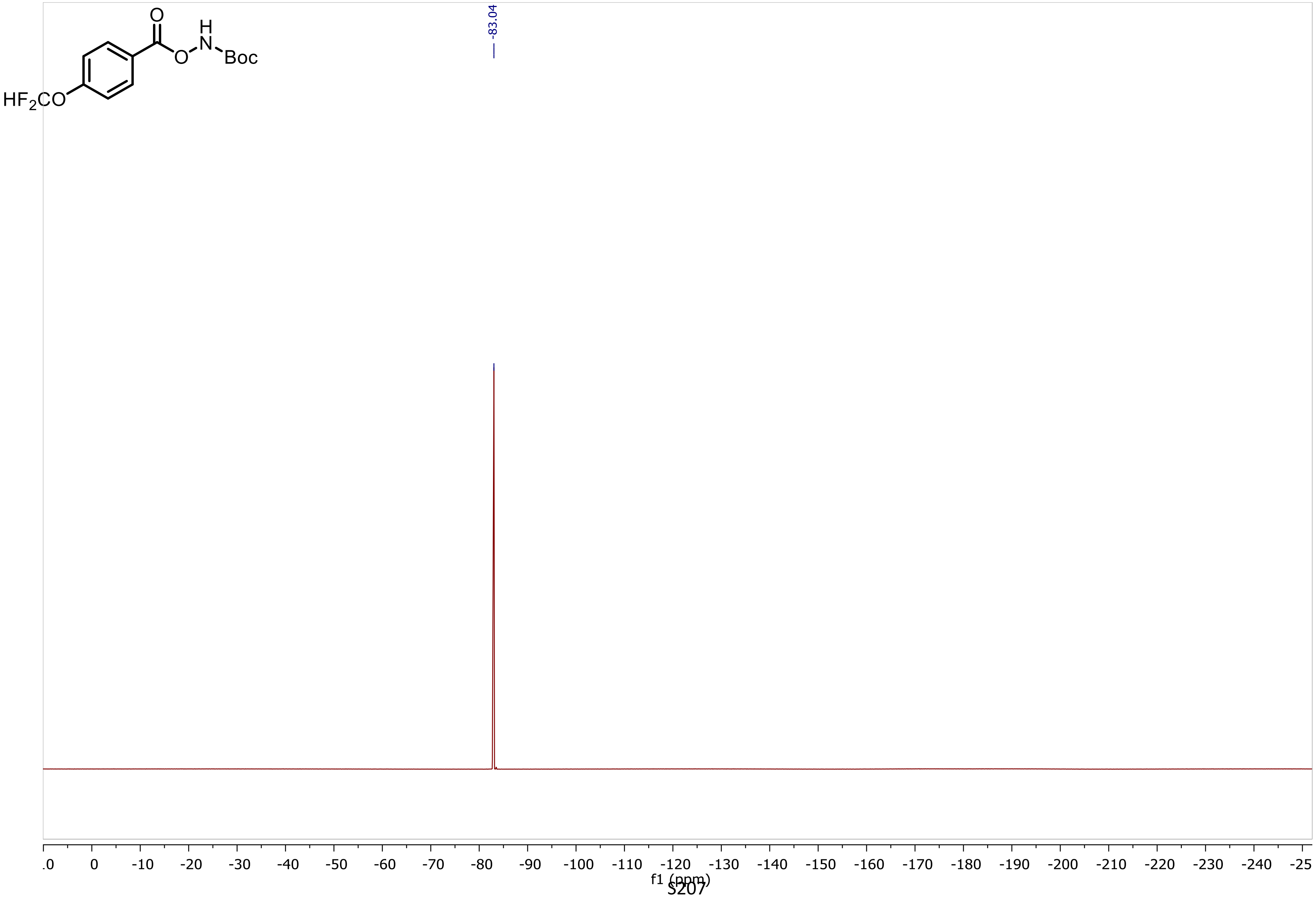

^{1}H NMR spectrum of *tert*-butyl ((2-fluorobenzoyl)oxy)carbamate in $CDCl_3$

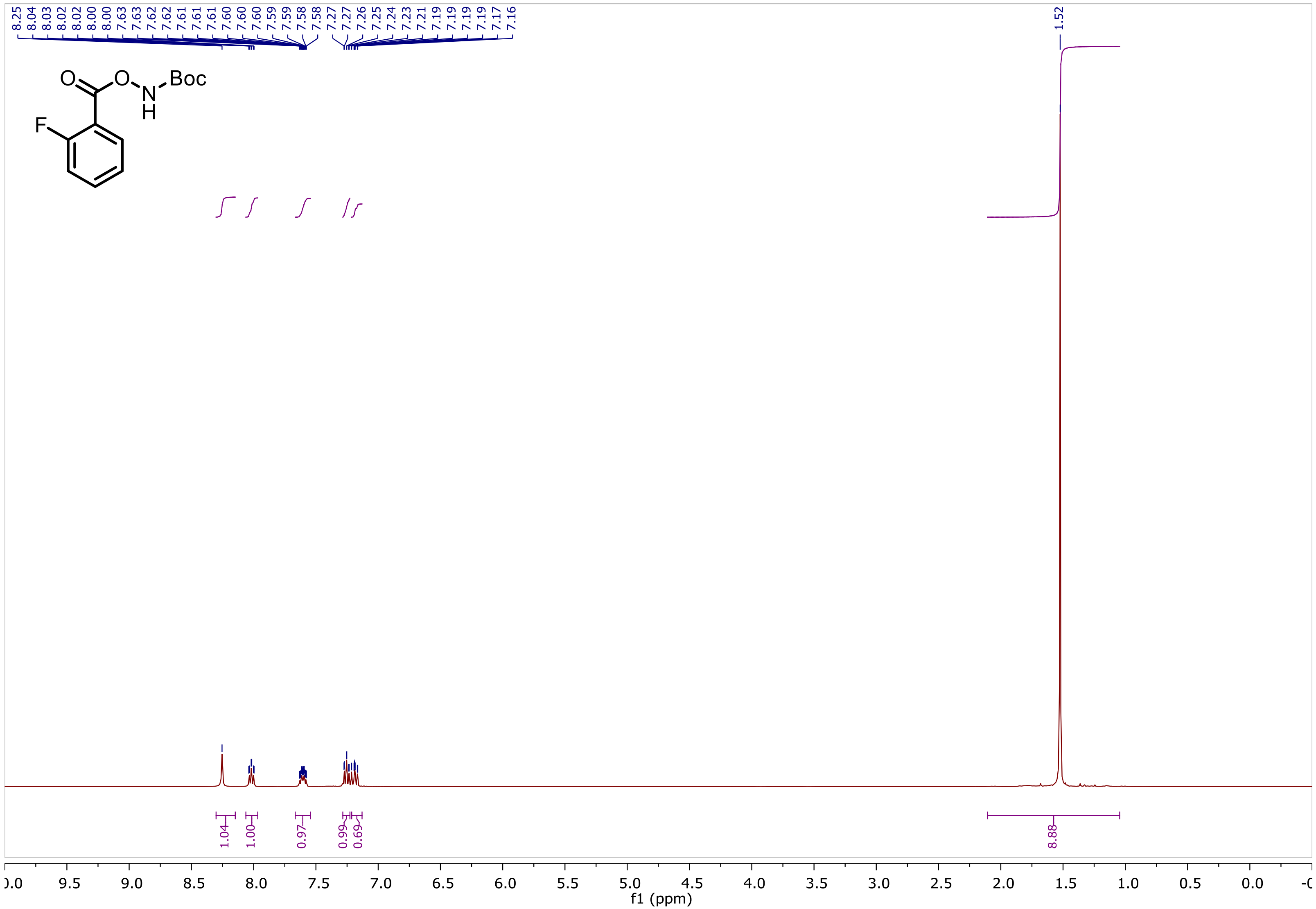

^{13}C NMR spectrum of *tert*-butyl ((2-fluorobenzoyl)oxy)carbamate in $CDCl_3$

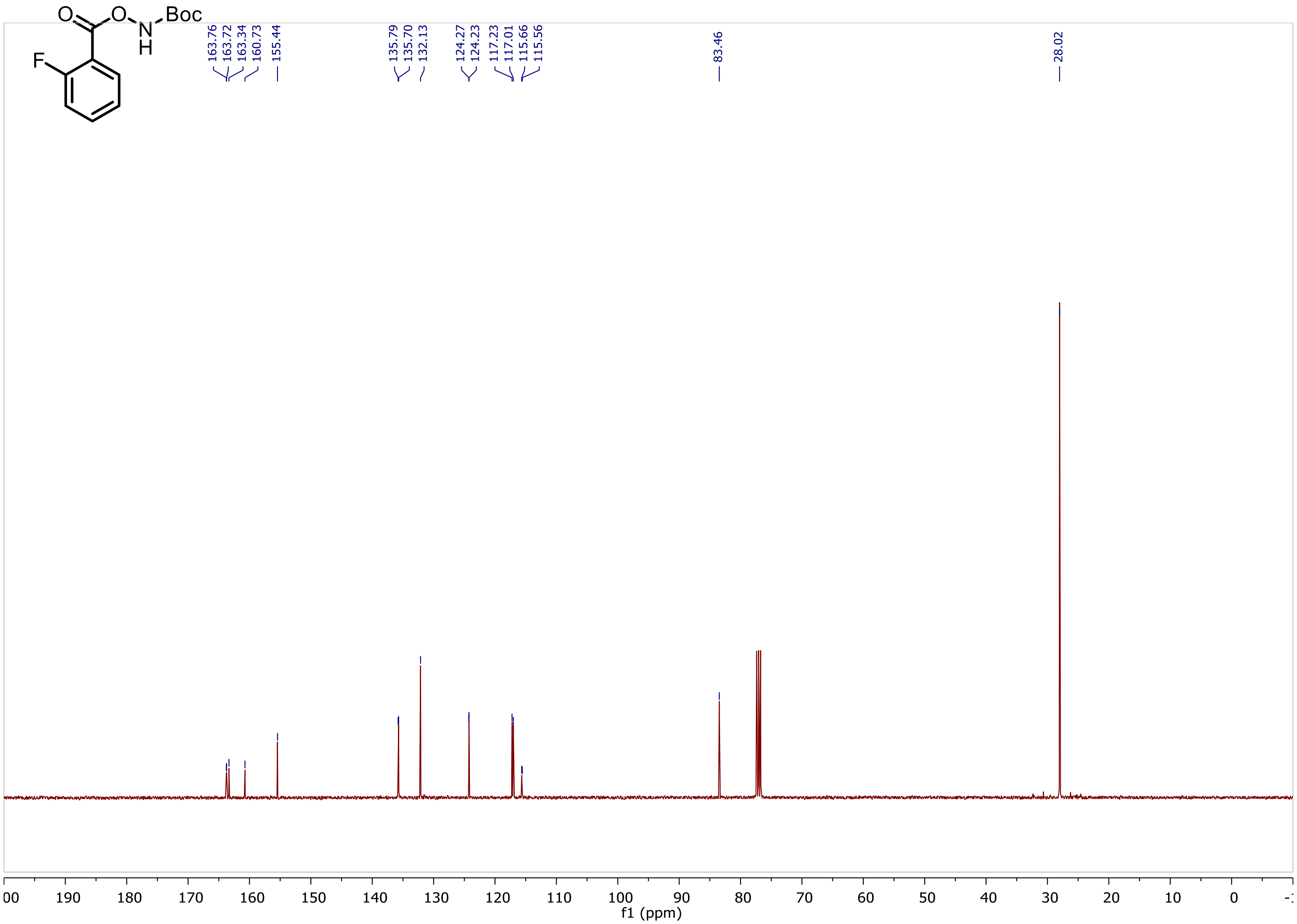

^{19}F NMR spectrum of *tert*-butyl ((2-fluorobenzoyl)oxy)carbamate in $CDCl_3$

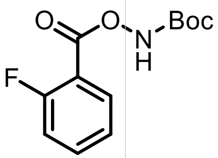

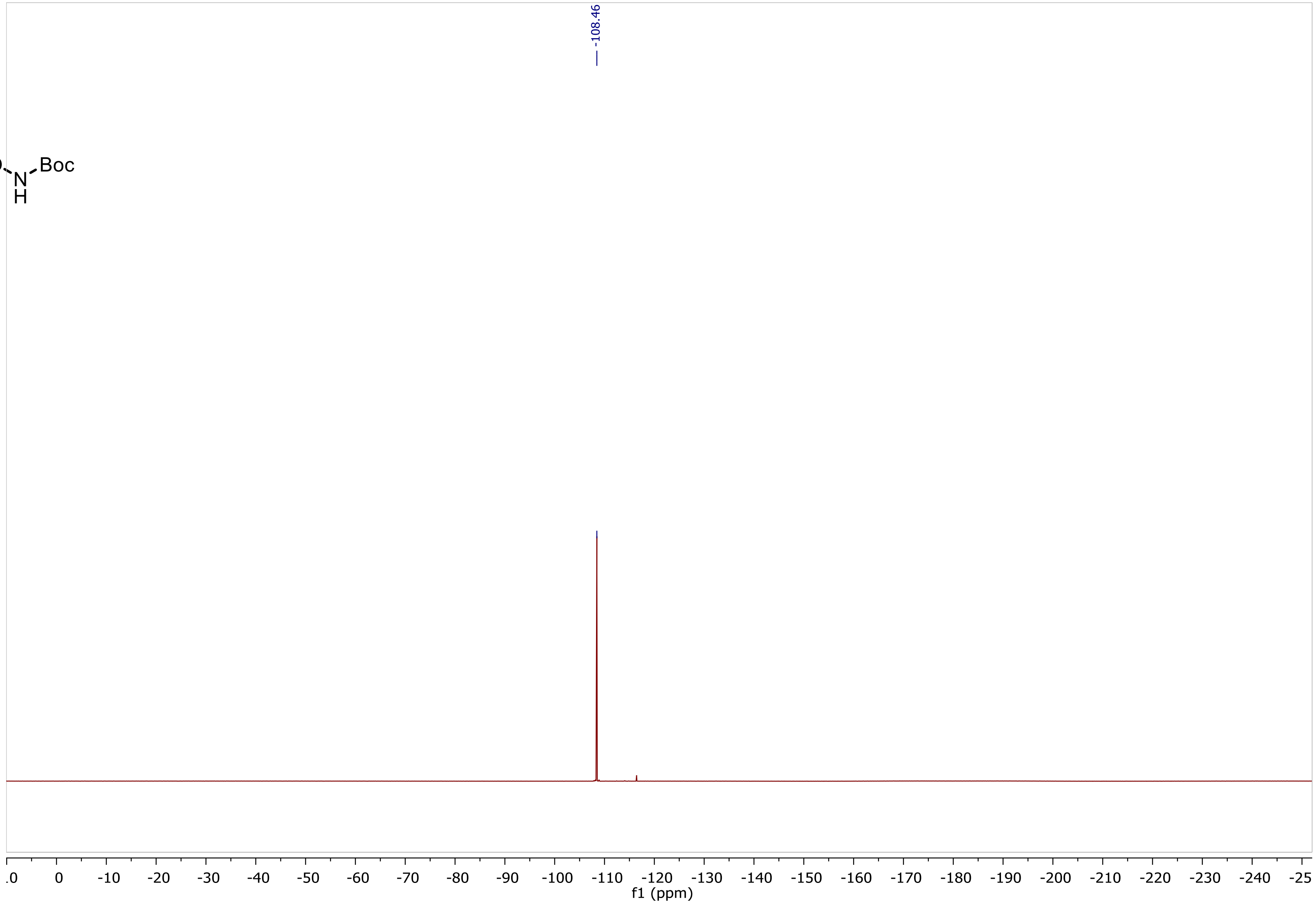

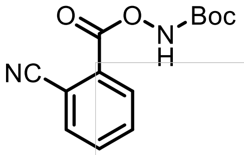

^{1}H NMR spectrum of *tert*-butyl ((2-cyanobenzoyl)oxy)carbamate in $CDCl_3$

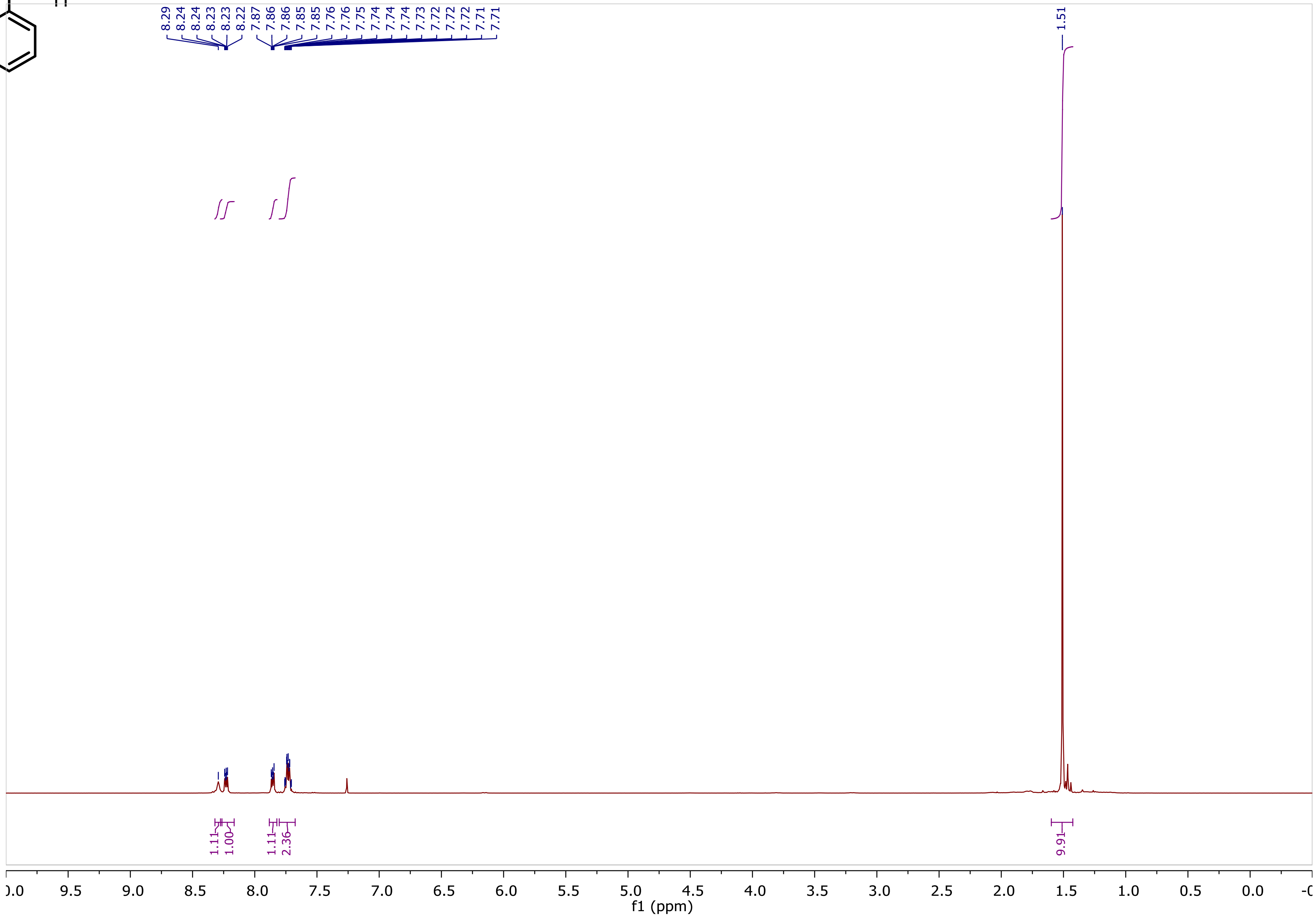

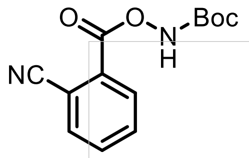

^{13}C NMR spectrum of *tert*-butyl ((2-cyanobenzoyl)oxy)carbamate in $CDCl_3$

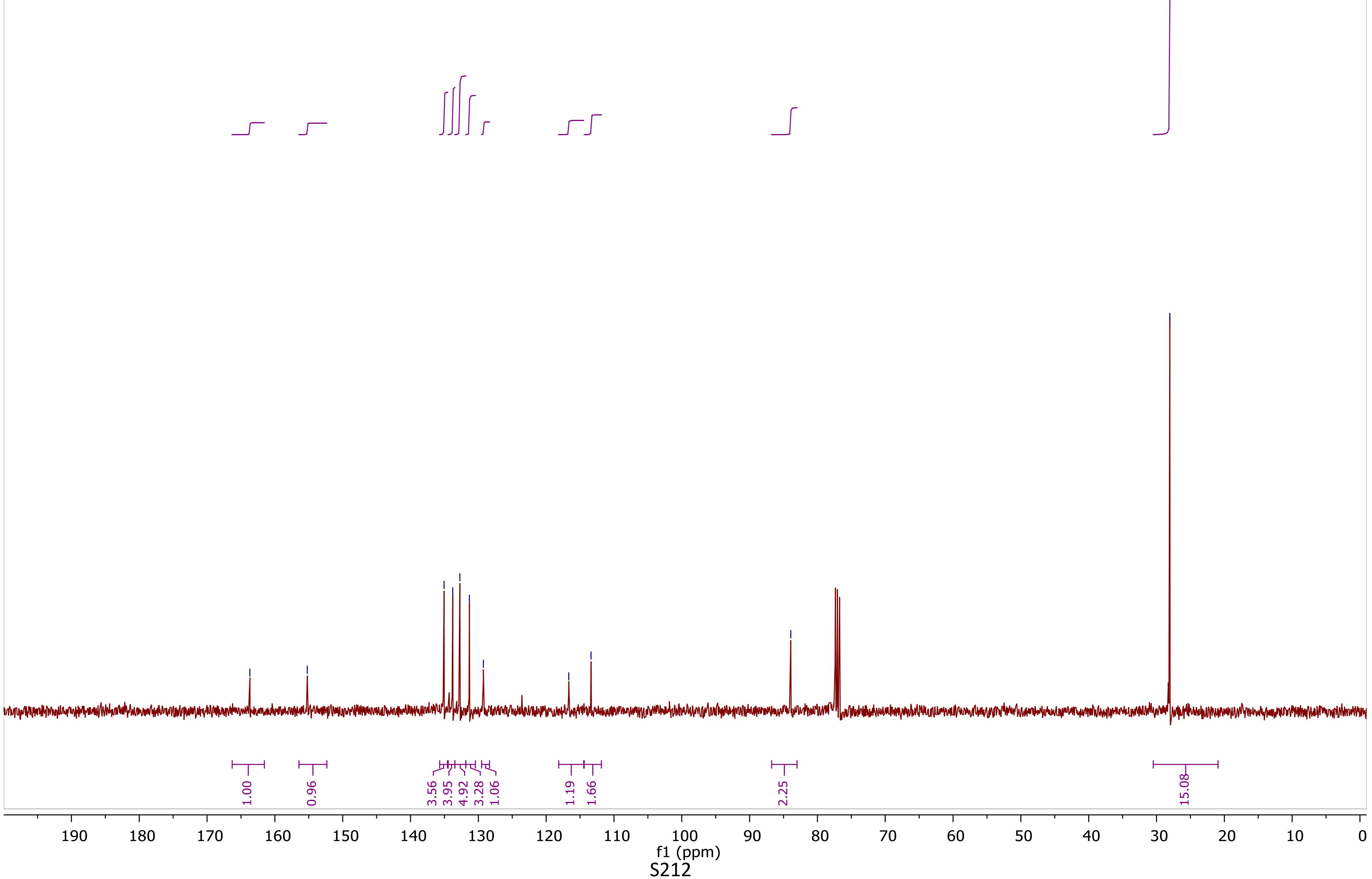

^{1}H NMR spectrum of *tert*-butyl ((4-vinylbenzoyl)oxy)carbamate in $CDCl_3$

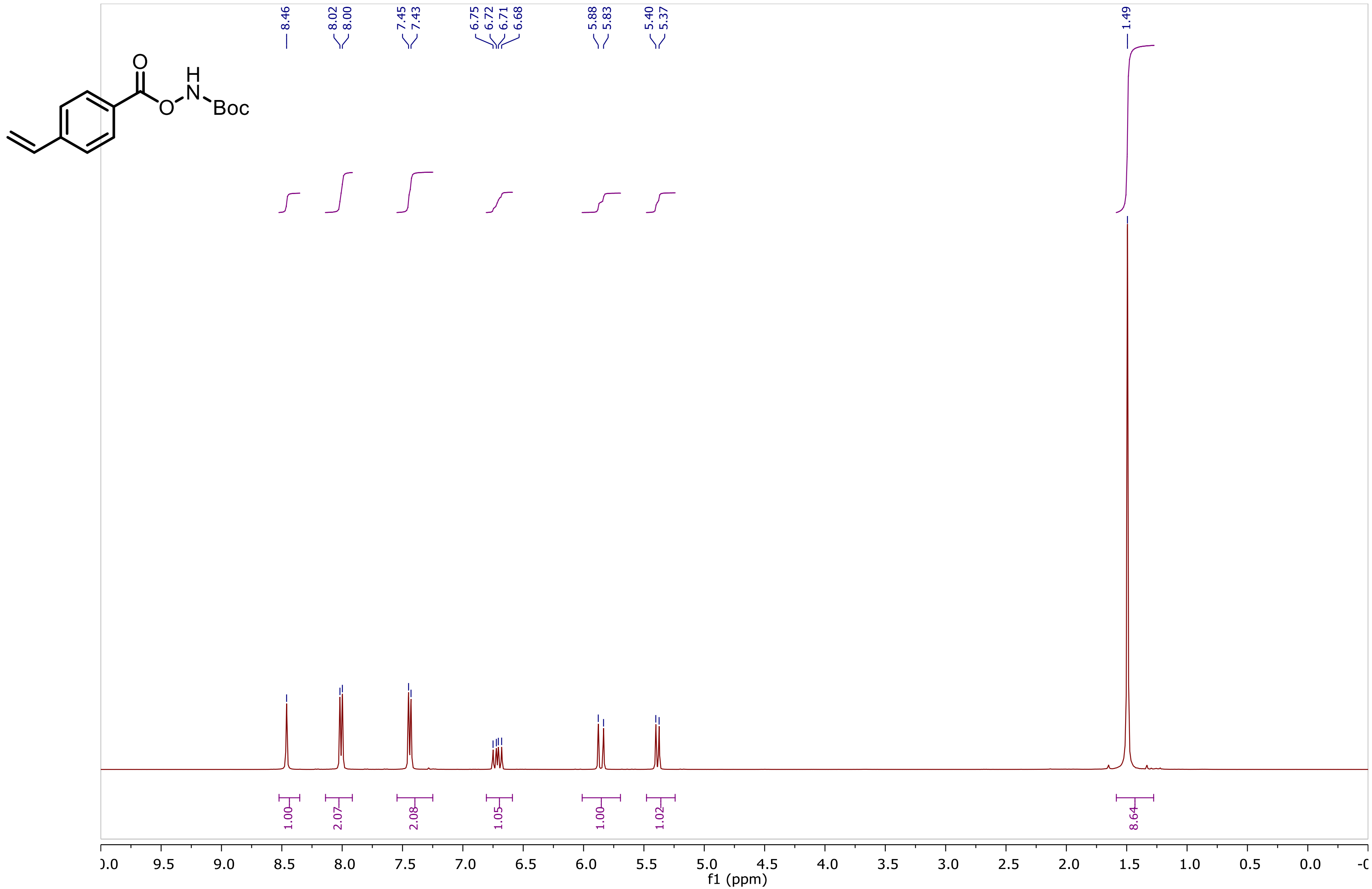

^{13}C NMR spectrum of *tert*-butyl ((4-vinylbenzoyl)oxy)carbamate in $CDCl_3$

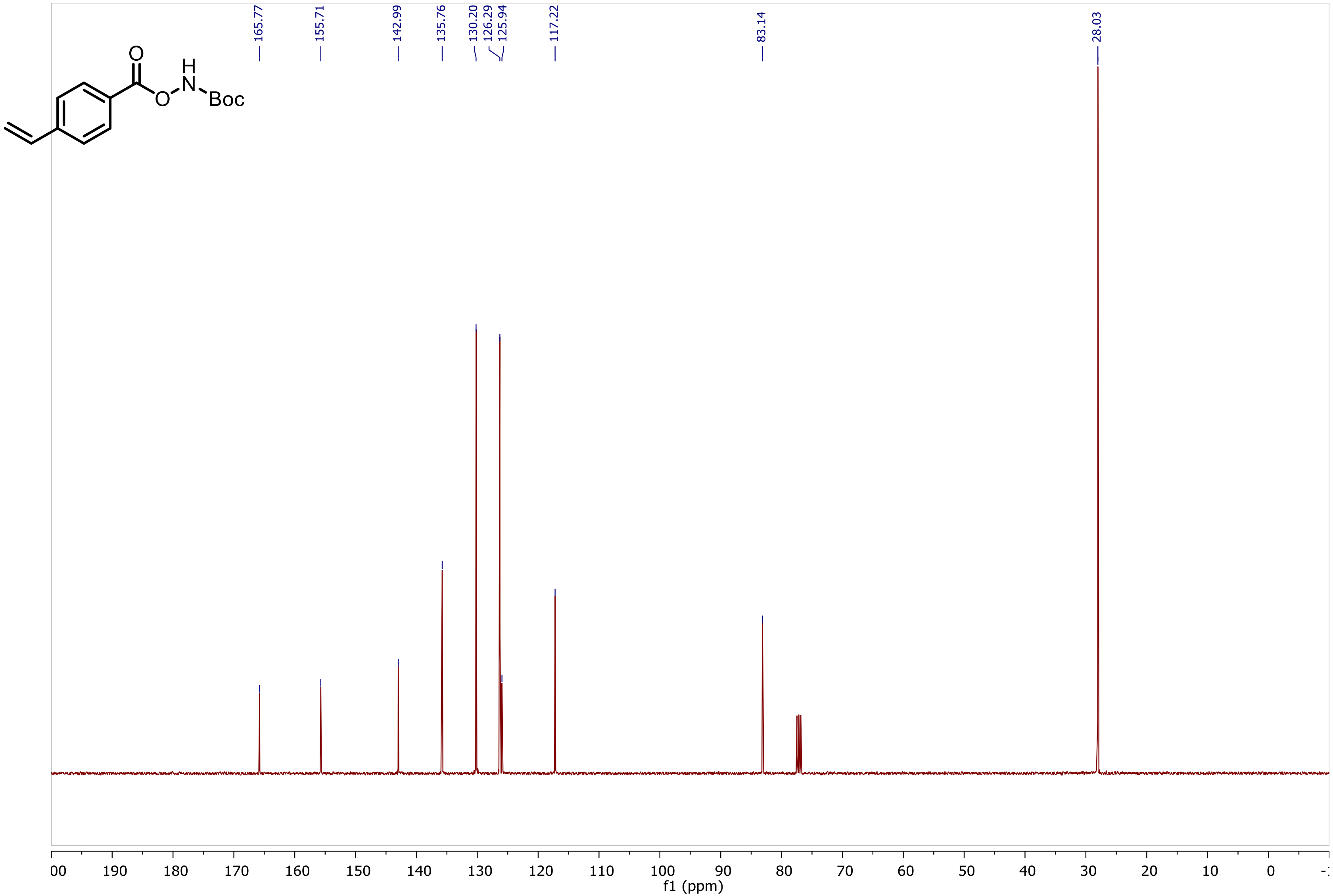

^{1}H NMR spectrum of *tert*-butyl ((4-phenylbenzoyl)oxy)carbamate in $CDCl_3$

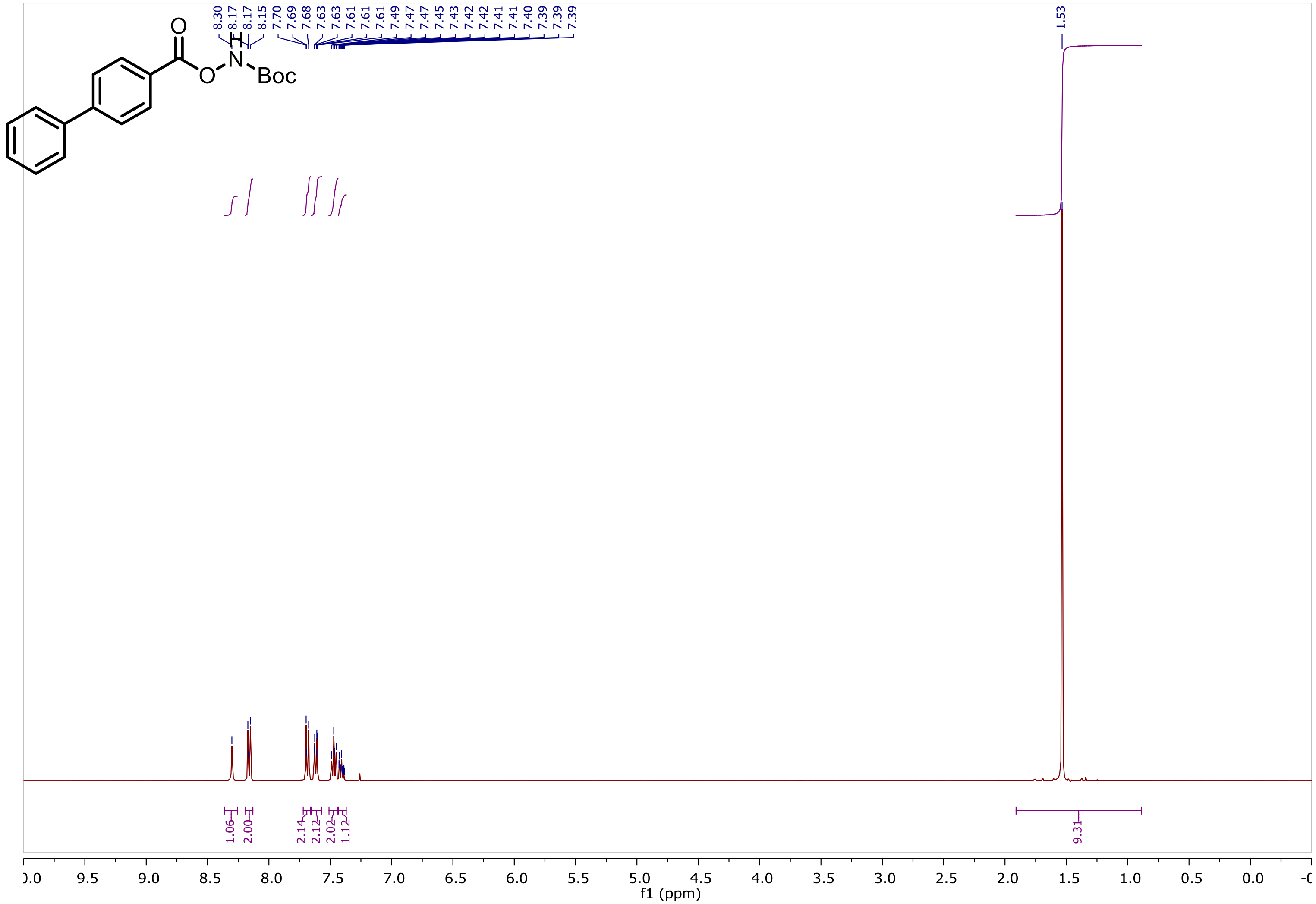

^{13}C NMR spectrum of *tert*-butyl ((4-phenylbenzoyl)oxy)carbamate in $CDCl_3$

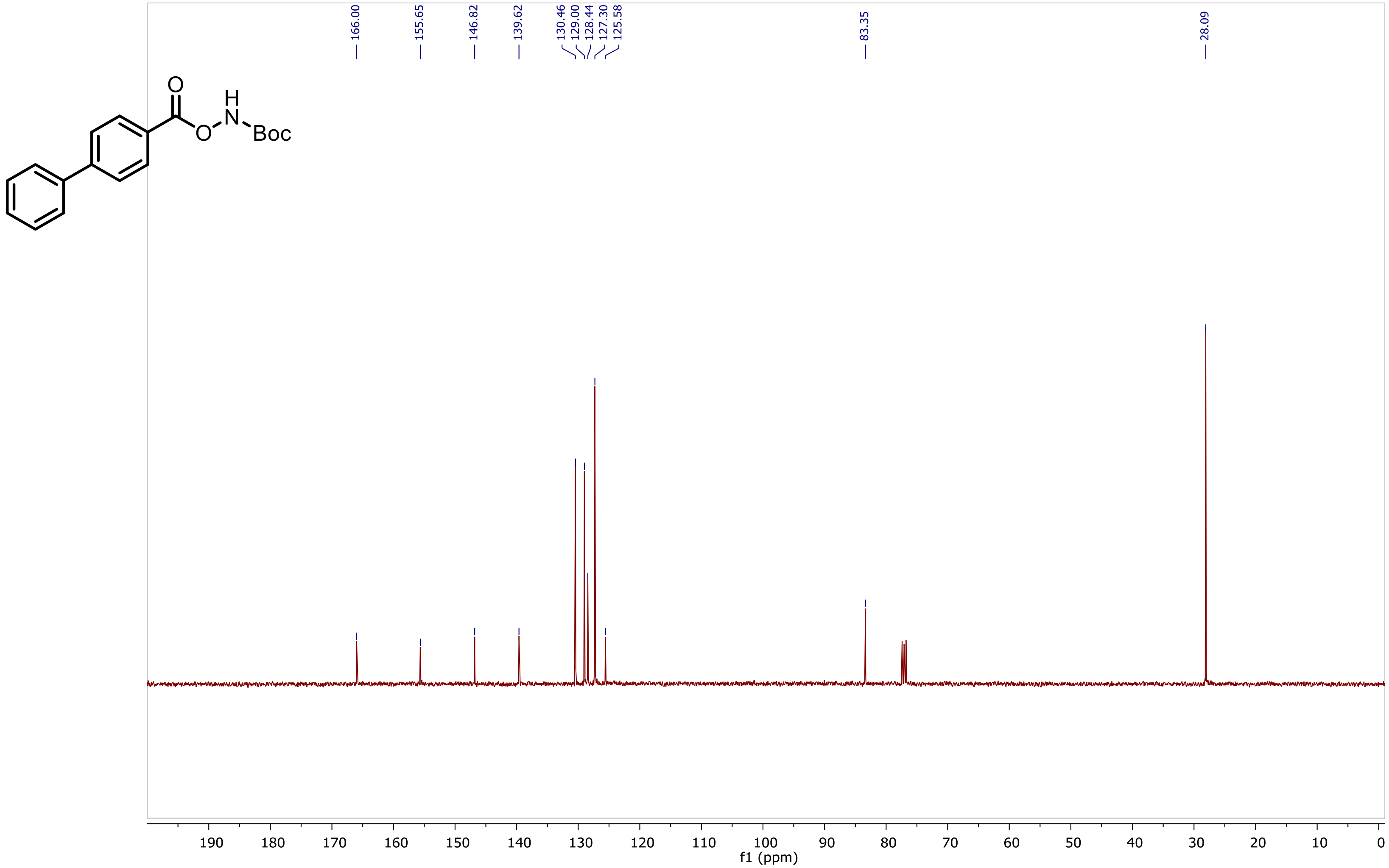

^{1}H NMR spectrum of *tert*-butyl methyl((3-methoxybenzoyl)oxy)carbamate **3a** in $CDCl_3$

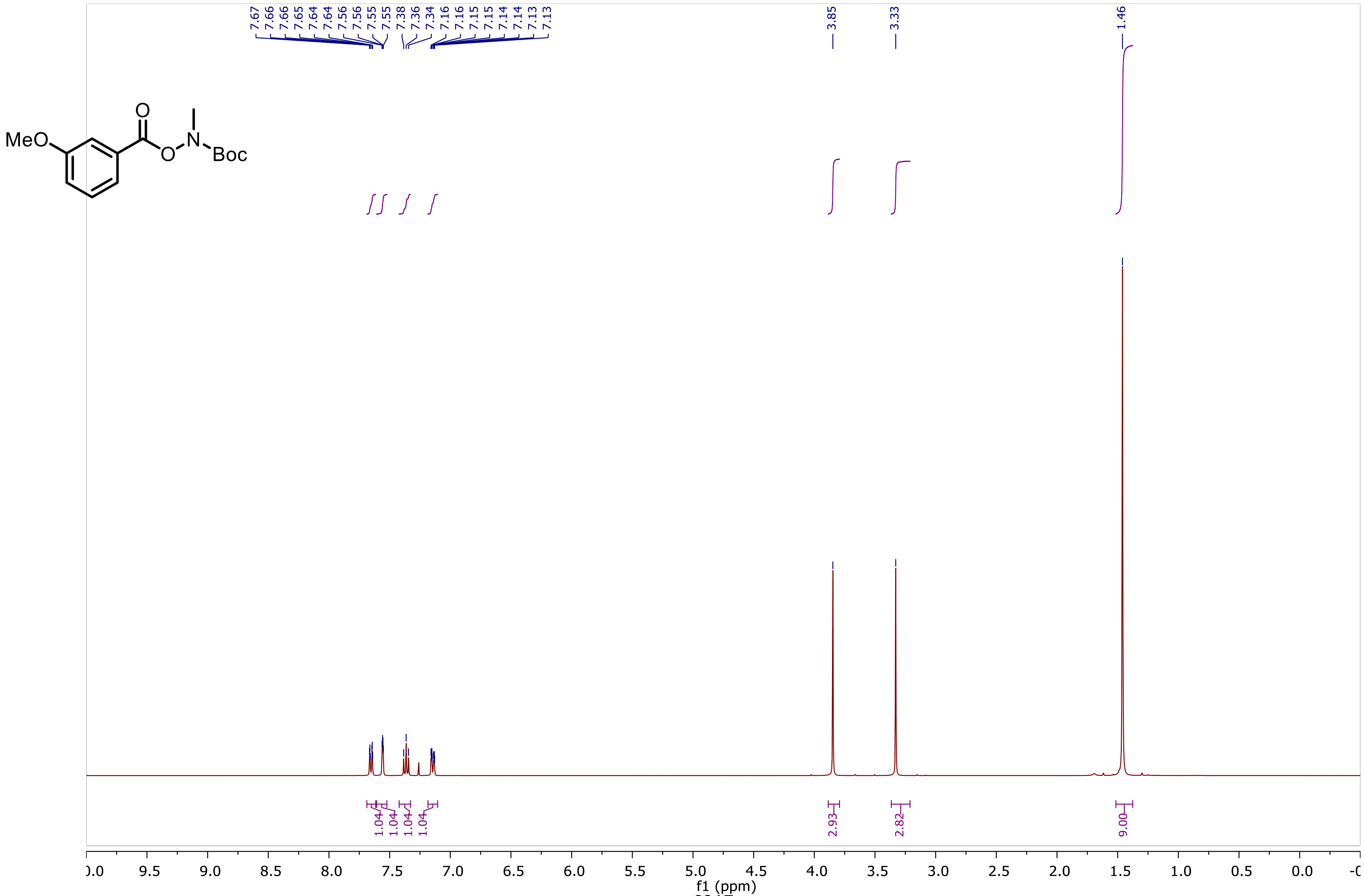

^{13}C NMR spectrum of *tert*-butyl methyl((3-methoxybenzoyl)oxy)carbamate **3a** in $CDCl_3$

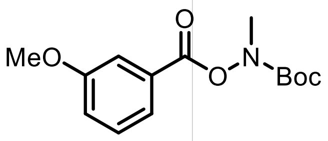

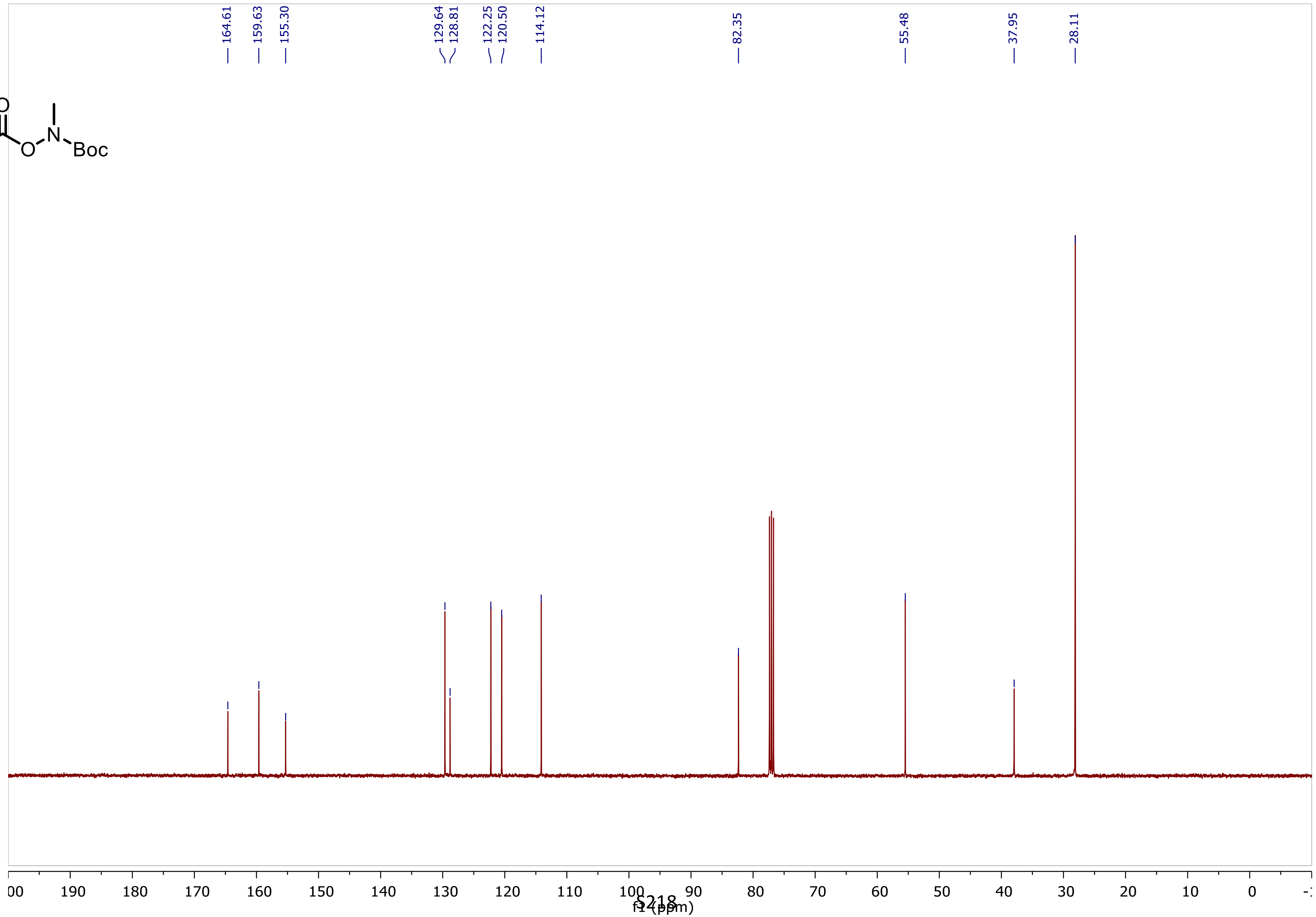

^{1}H NMR spectrum of *tert*-butyl methyl((3-methylbenzoyl)oxy)carbamate **3f** in $CDCl_3$

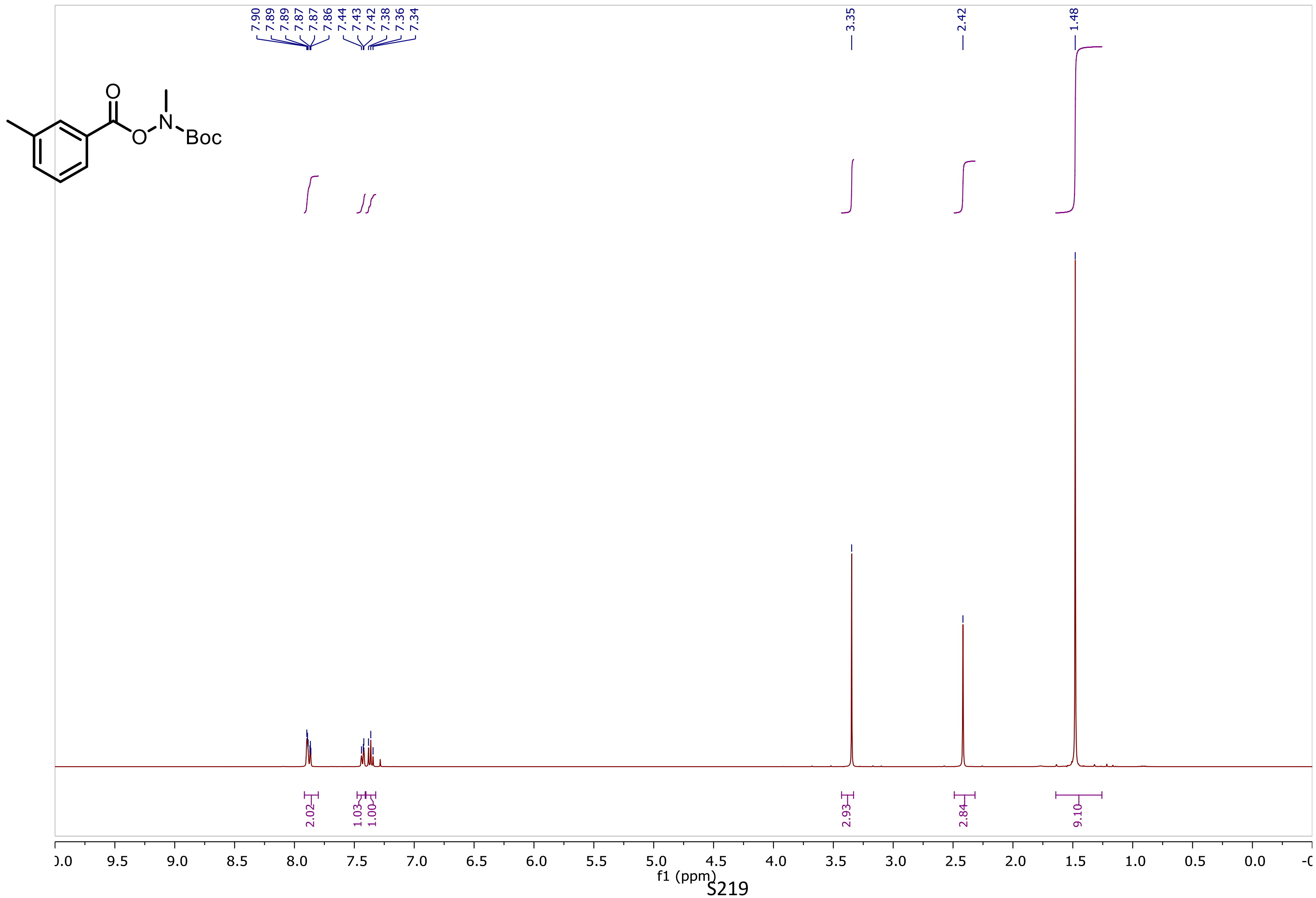

^{13}C NMR spectrum of *tert*-butyl methyl((3-methylbenzoyl)oxy)carbamate **3f** in $CDCl_3$

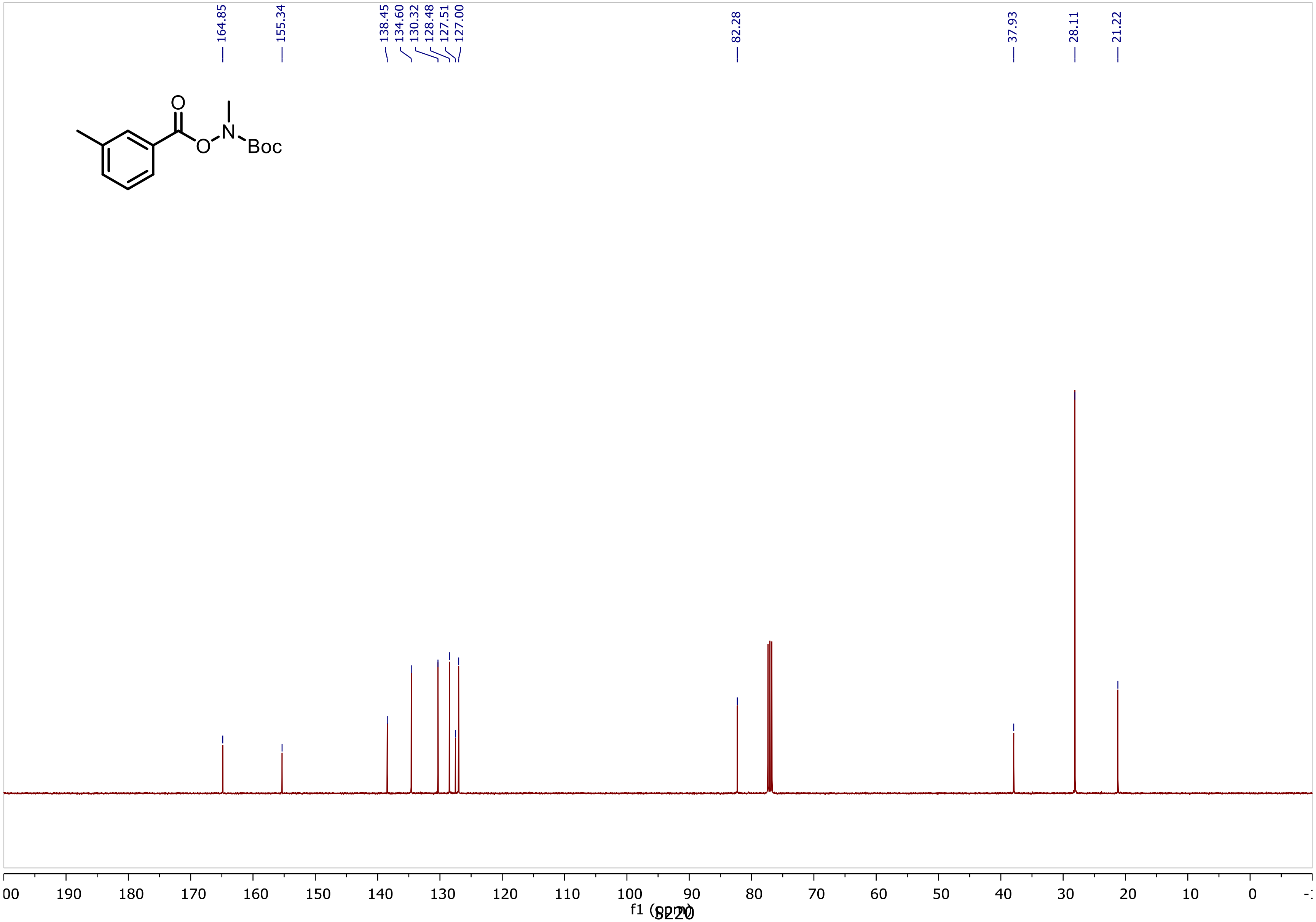

^{1}H NMR spectrum of *tert*-butyl methyl((3-methoxy-2-methylbenzoyl)oxy)carbamate **3e** in $CDCl_3$

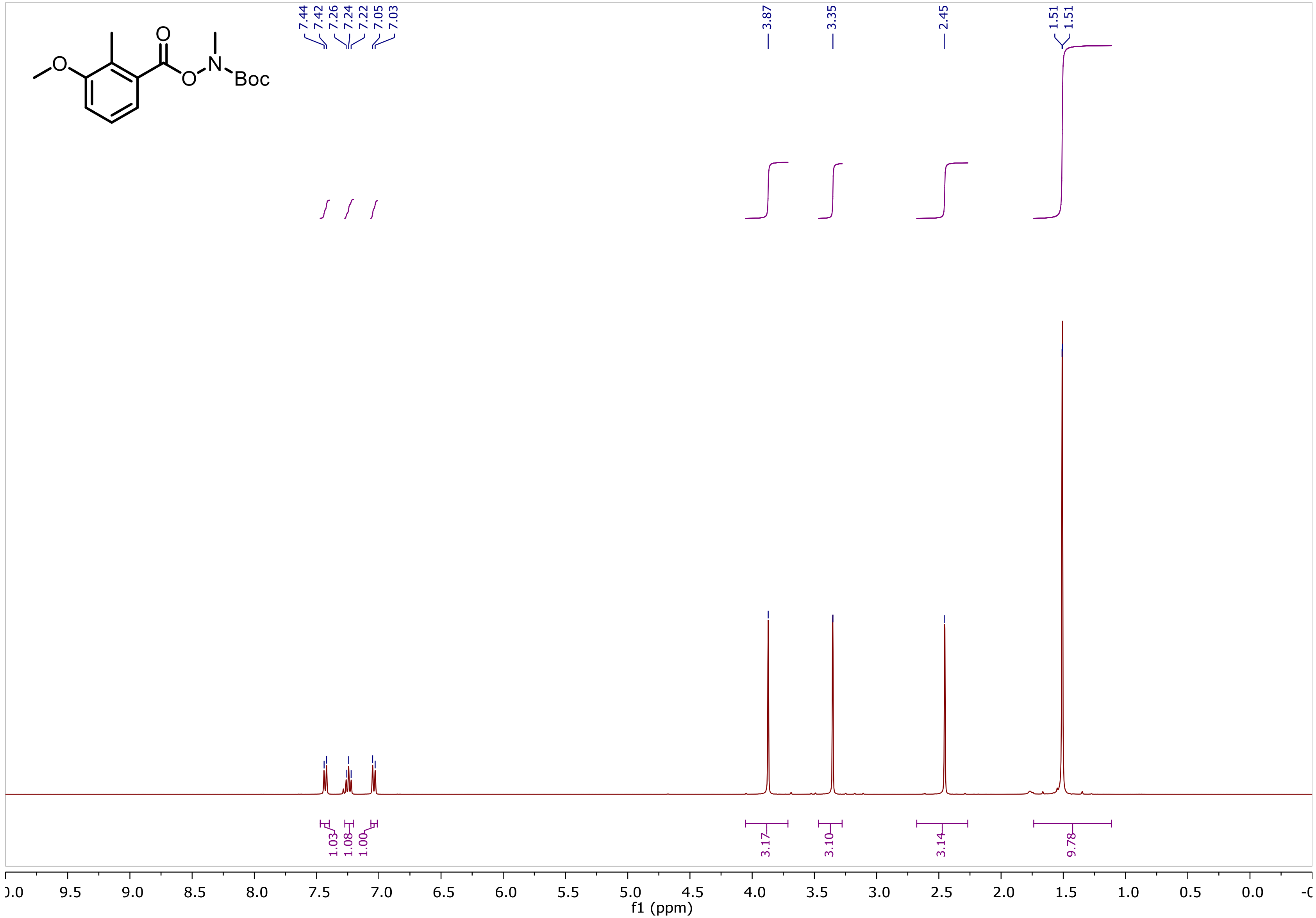

^{13}C NMR spectrum of *tert*-butyl methyl((3-methoxy-2-methylbenzoyl)oxy)carbamate **3e** in $CDCl_3$

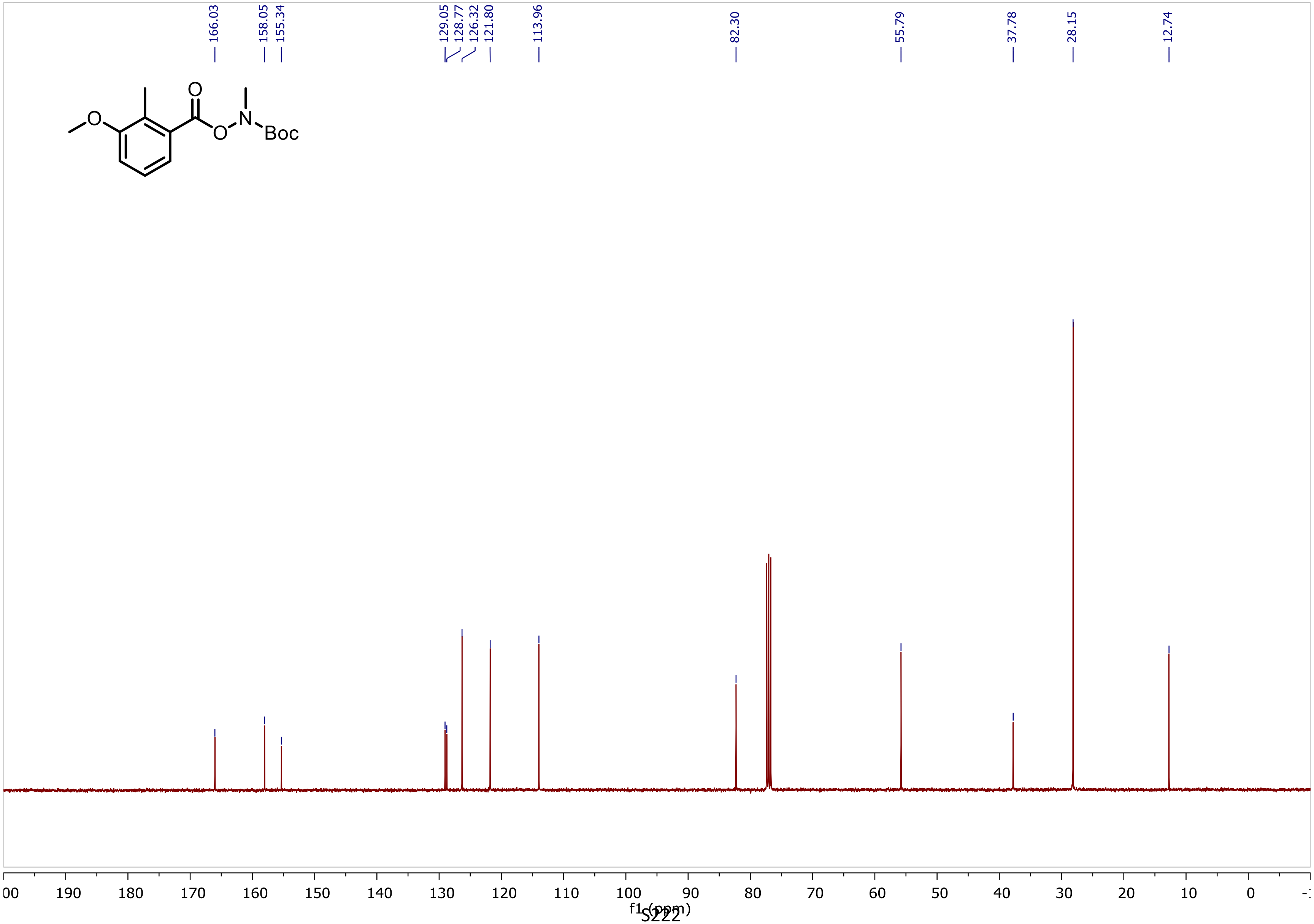

^{1}H NMR spectrum of *tert*-butyl methyl((2-bromo-3-methoxybenzoyl)oxy)carbamate **3d** in $CDCl_3$

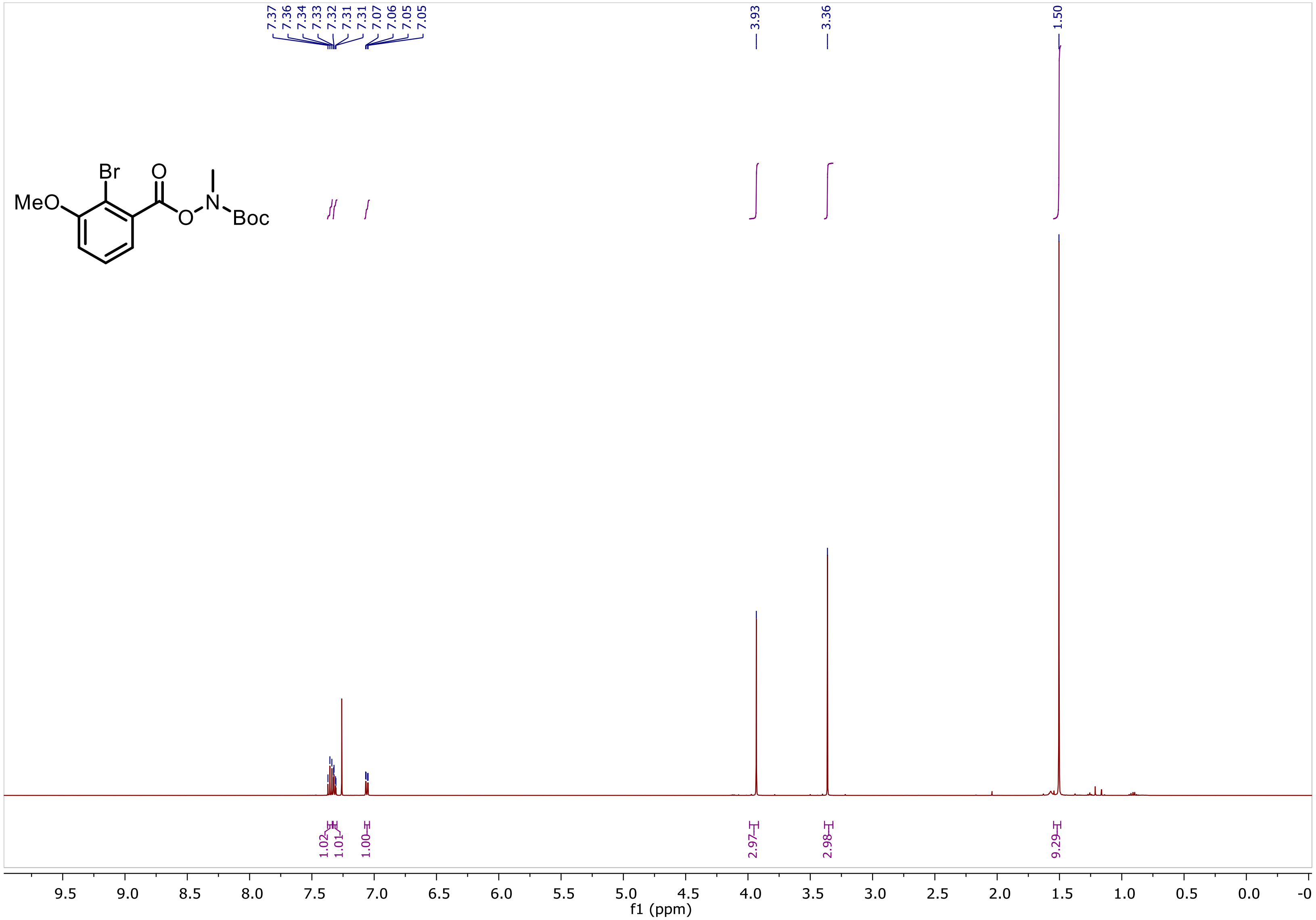

^{13}C NMR spectrum of *tert*-butyl methyl((2-bromo-3-methoxybenzoyl)oxy)carbamate **3d** in $CDCl_3$

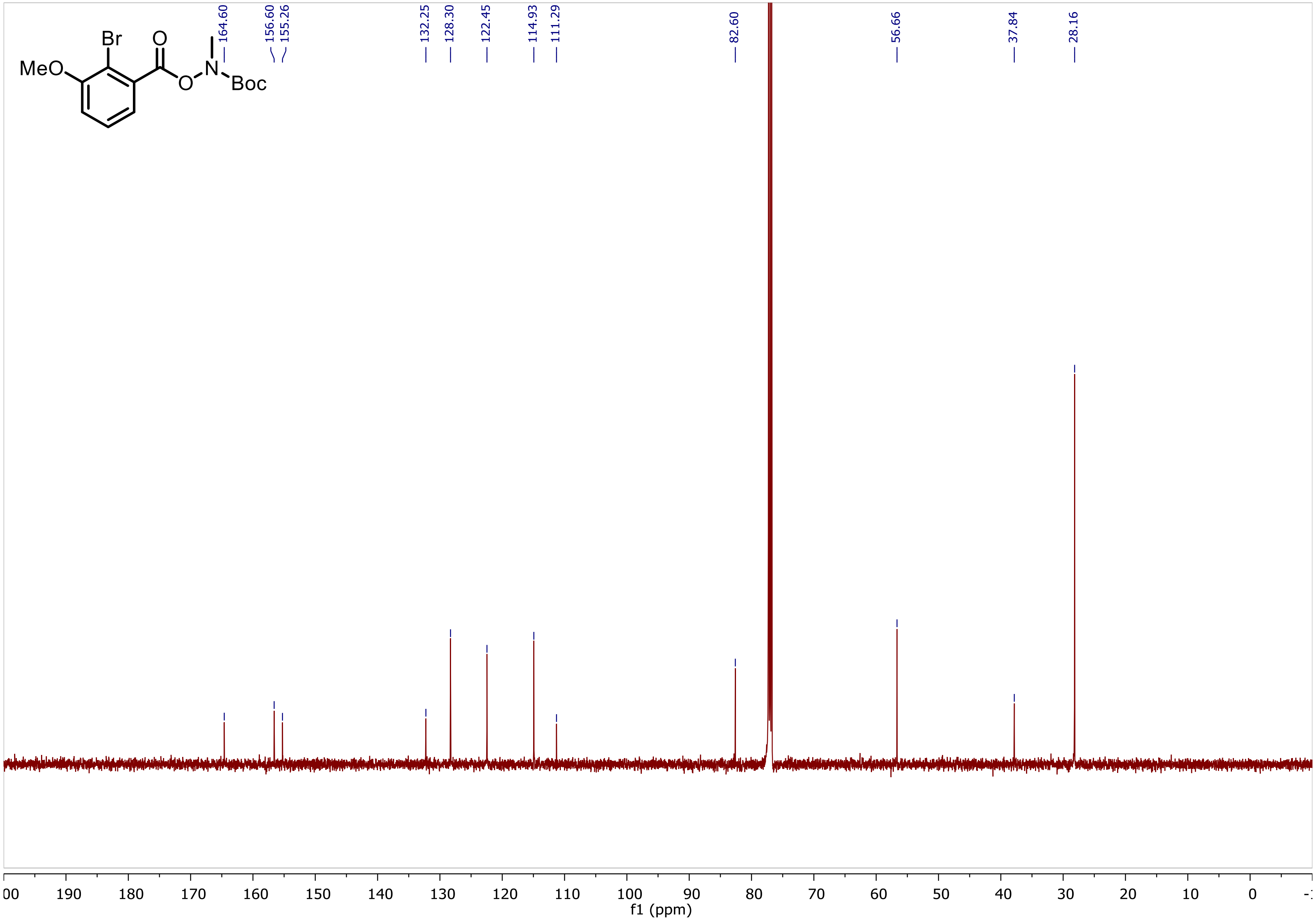

^{1}H NMR spectrum of *tert*-butyl methyl((3,5-dimethoxybenzoyl)oxy)carbamate **3b** in $CDCl_3$

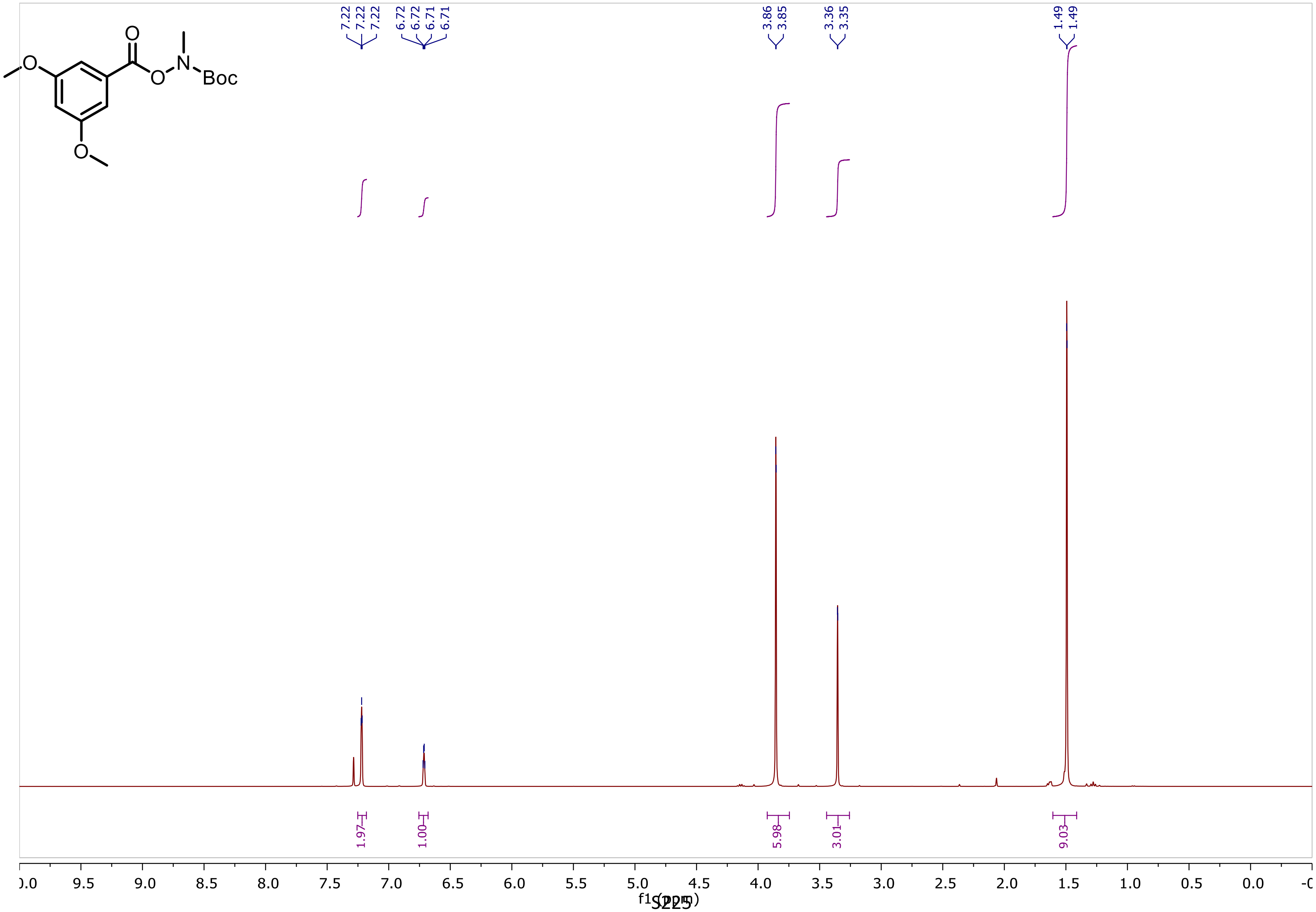

^{13}C NMR spectrum of *tert*-butyl methyl((3,5-dimethoxybenzoyl)oxy)carbamate **3b** in $CDCl_3$

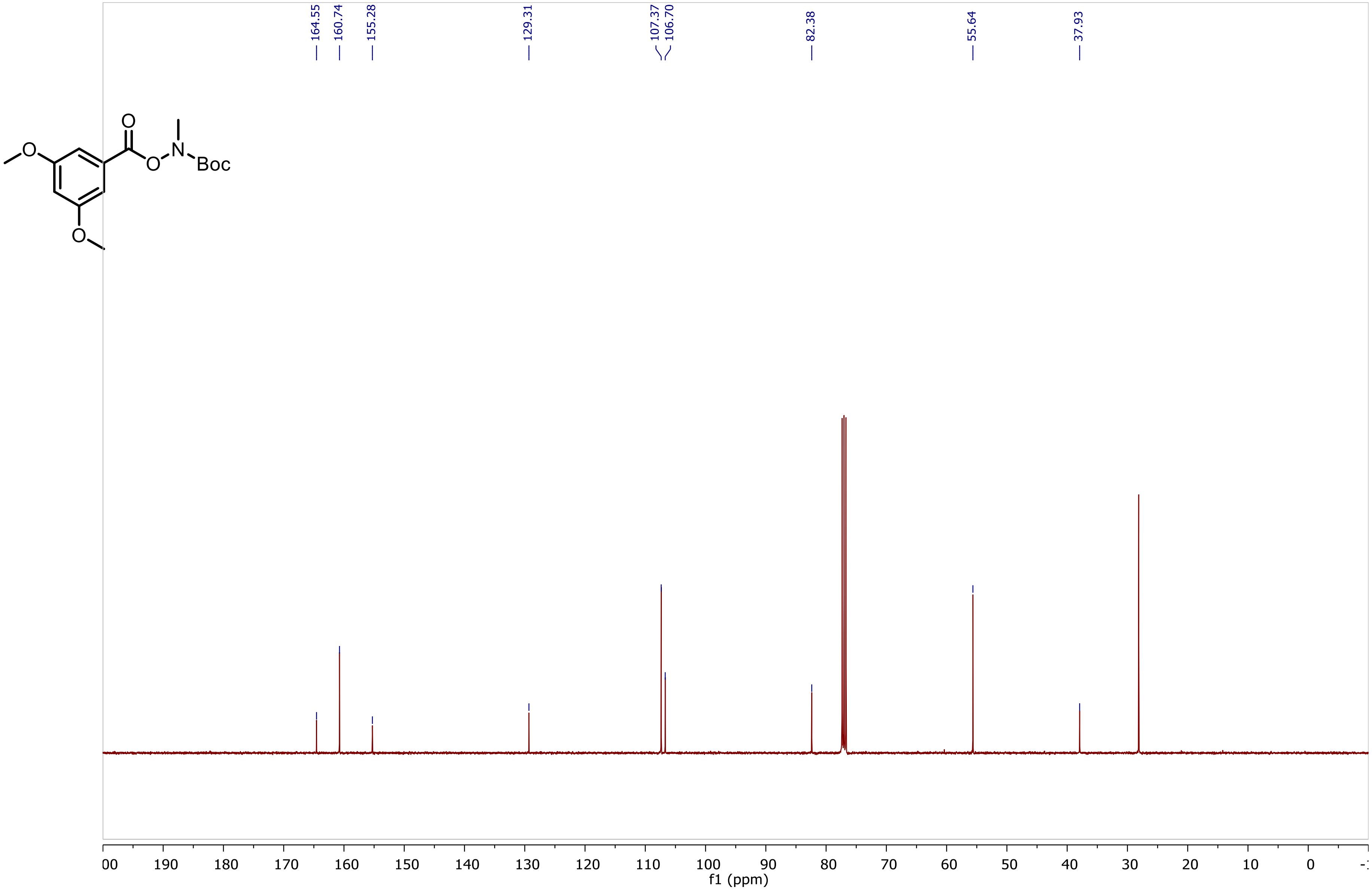

^{1}H NMR spectrum of *tert*-butyl ((2-chloro-5-methoxybenzoyl)oxy)carbamate **S3c** in $CDCl_3$

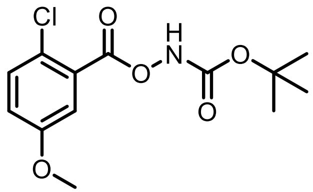

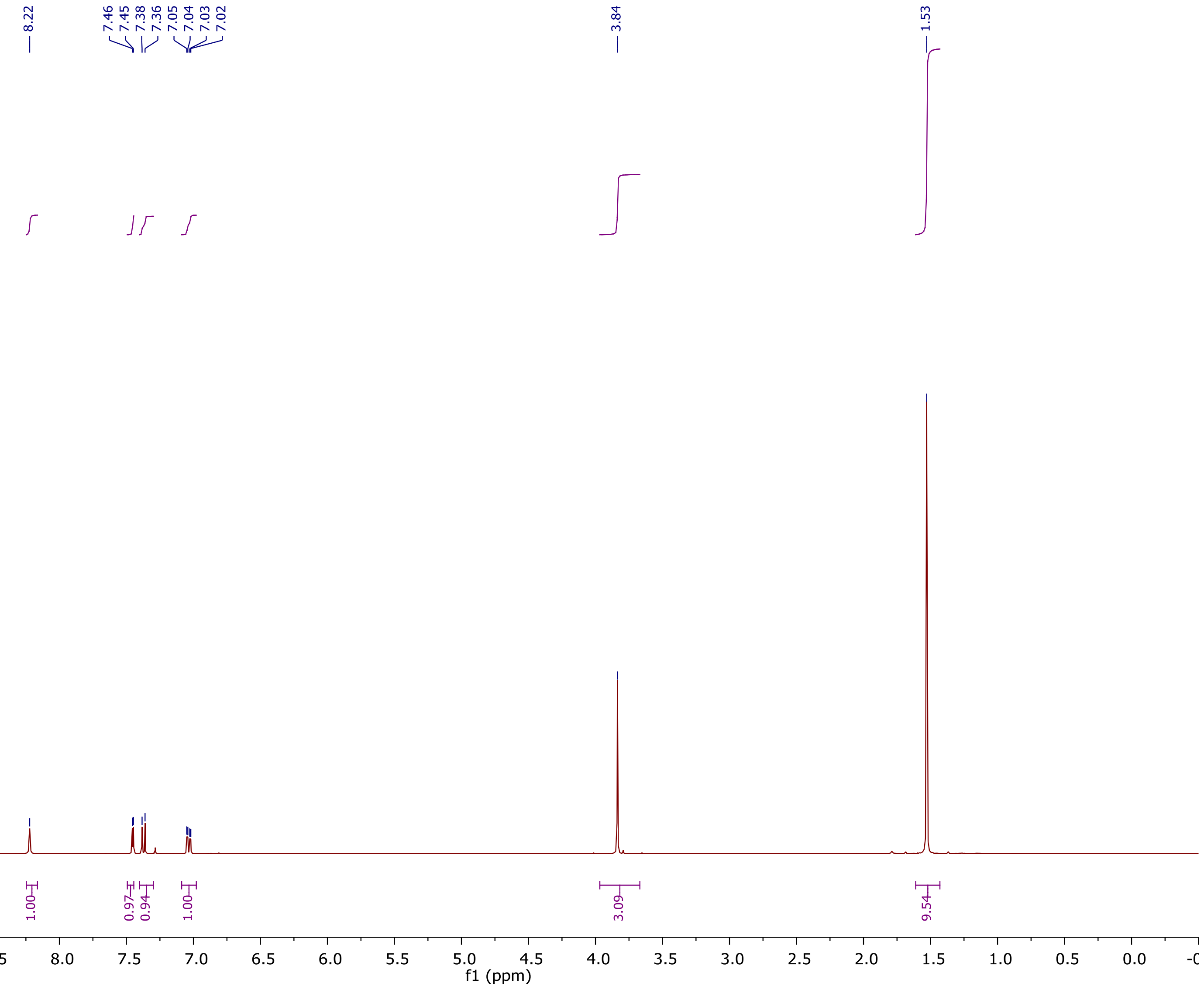

^{13}C NMR spectrum of *tert*-butyl ((2-chloro-5-methoxybenzoyl)oxy)carbamate **S3c** in $CDCl_3$

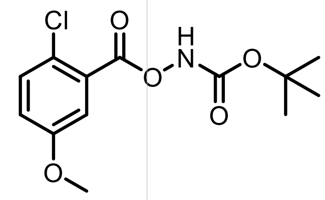

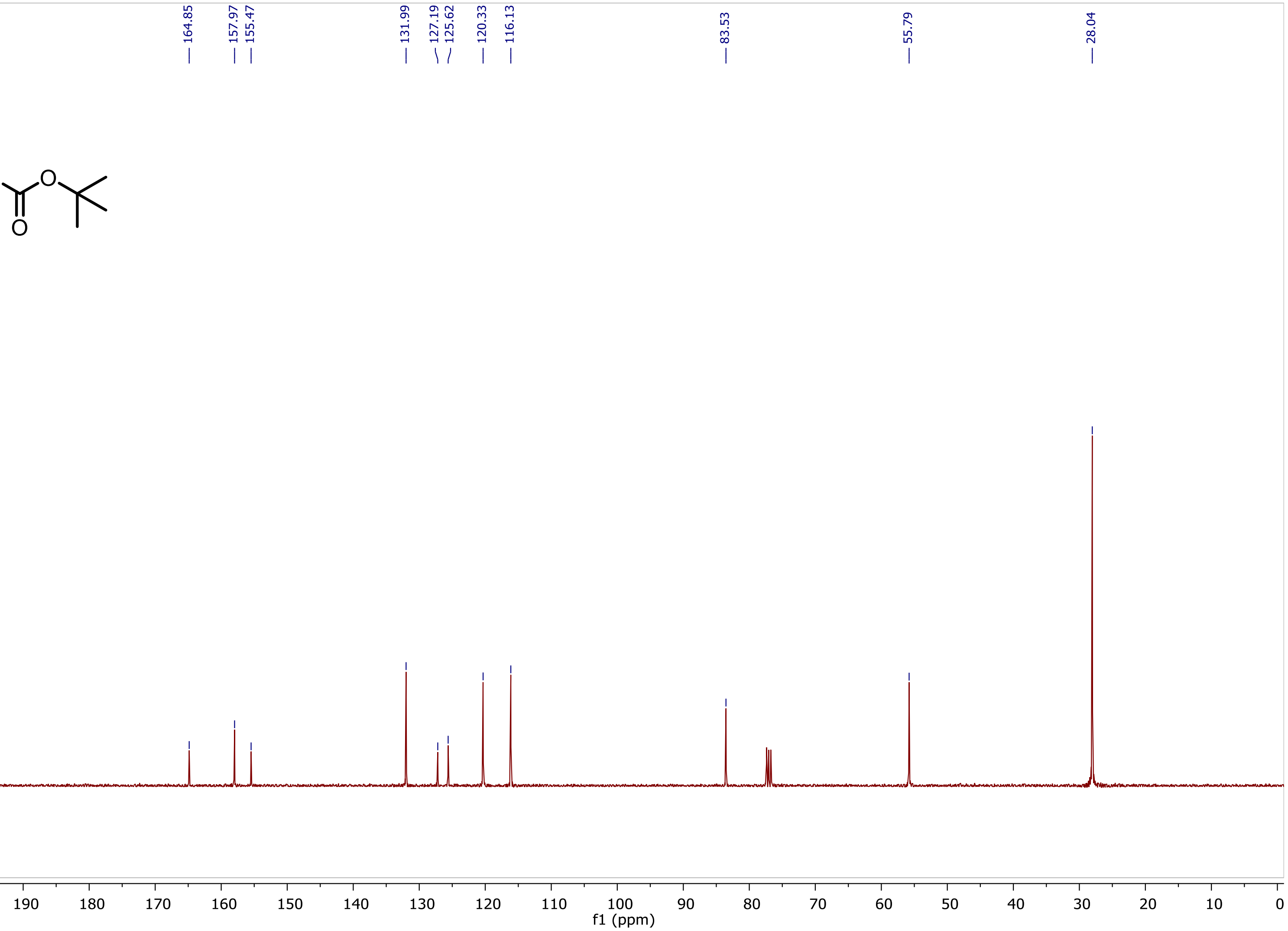

^{1}H NMR spectrum of *tert*-butyl methyl((2-chloro-5-methoxybenzoyl)oxy)carbamate **3c** in $CDCl_3$

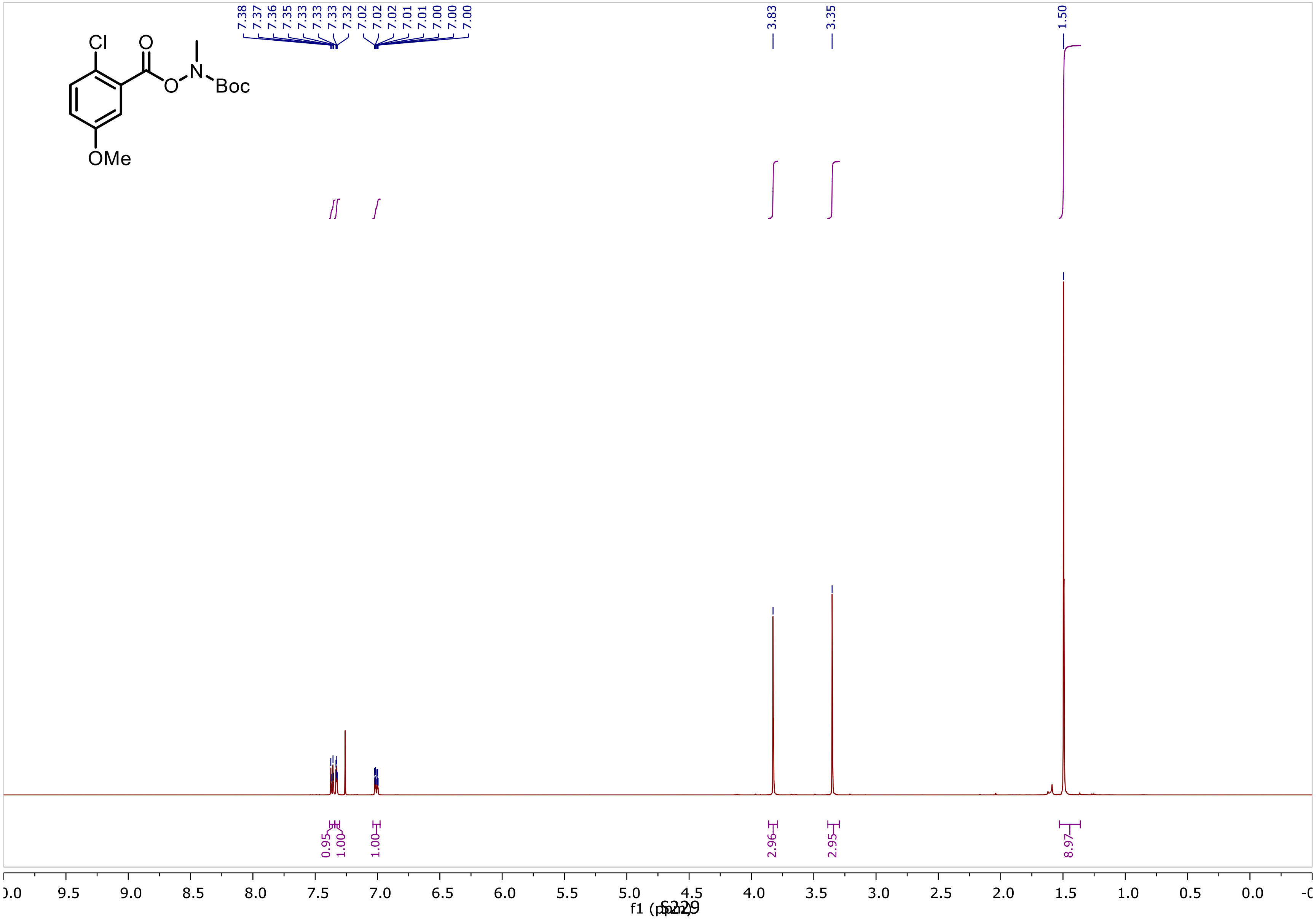

^{13}C NMR spectrum of *tert*-butyl methyl((2-chloro-5-methoxybenzoyl)oxy)carbamate **3c** in $CDCl_3$

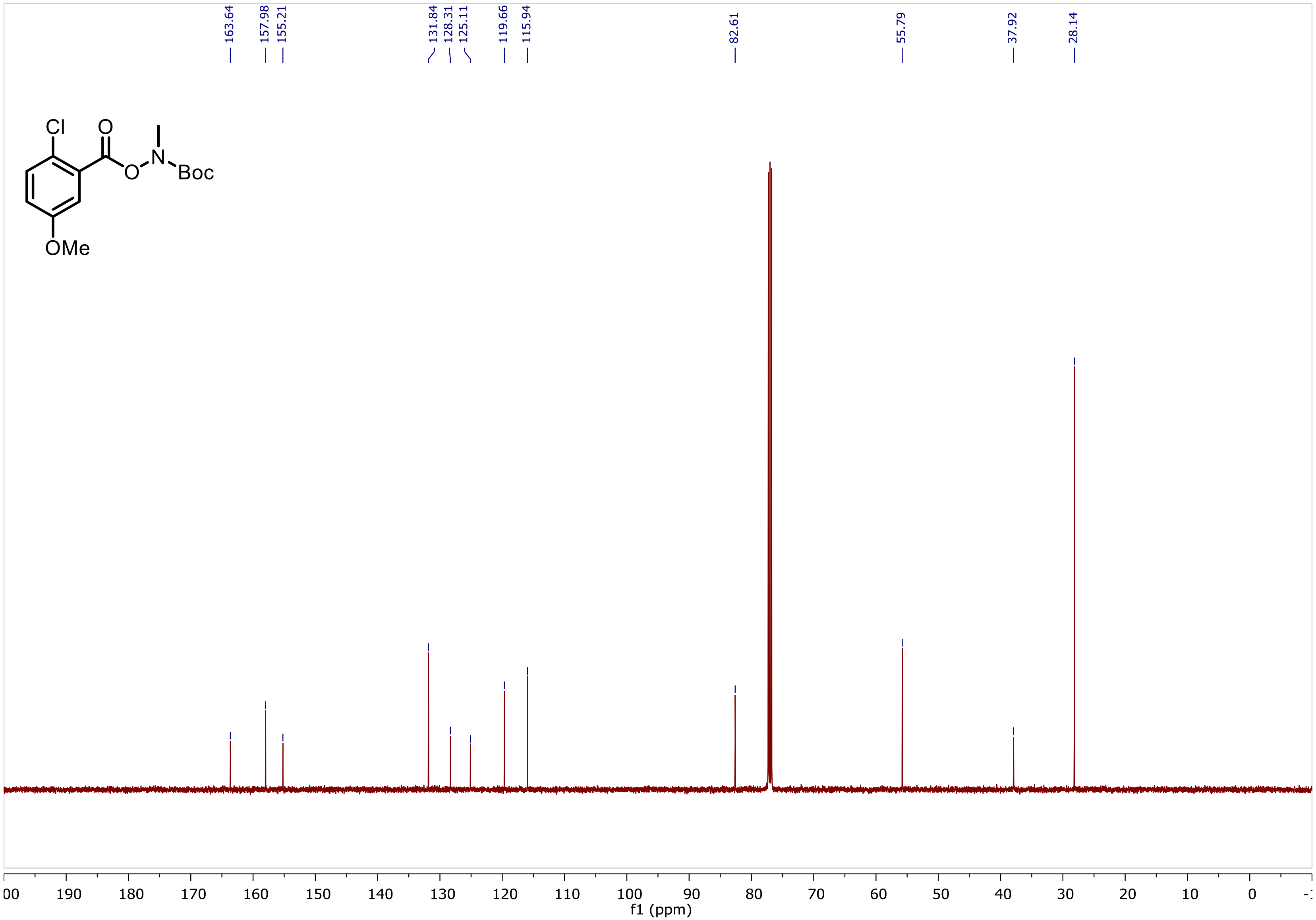

^{1}H NMR spectrum of *tert*-butyl methyl((2-methyl-3-chlorobenzoyl)oxy)carbamate **3g** in $CDCl_3$

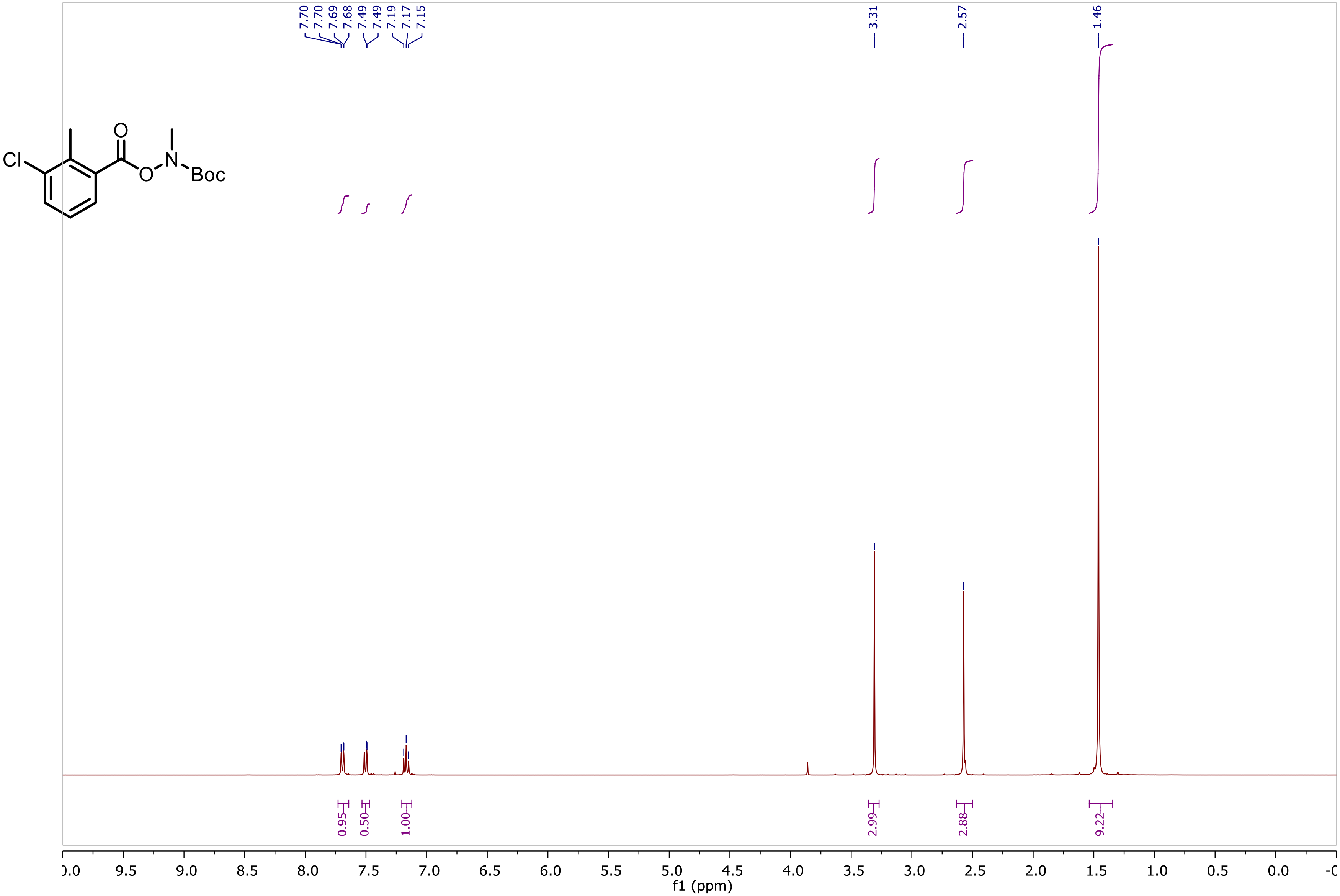

^{13}C NMR spectrum of *tert*-butyl methyl((2-methyl-3-chlorobenzoyl)oxy)carbamate **3g** in $CDCl_3$

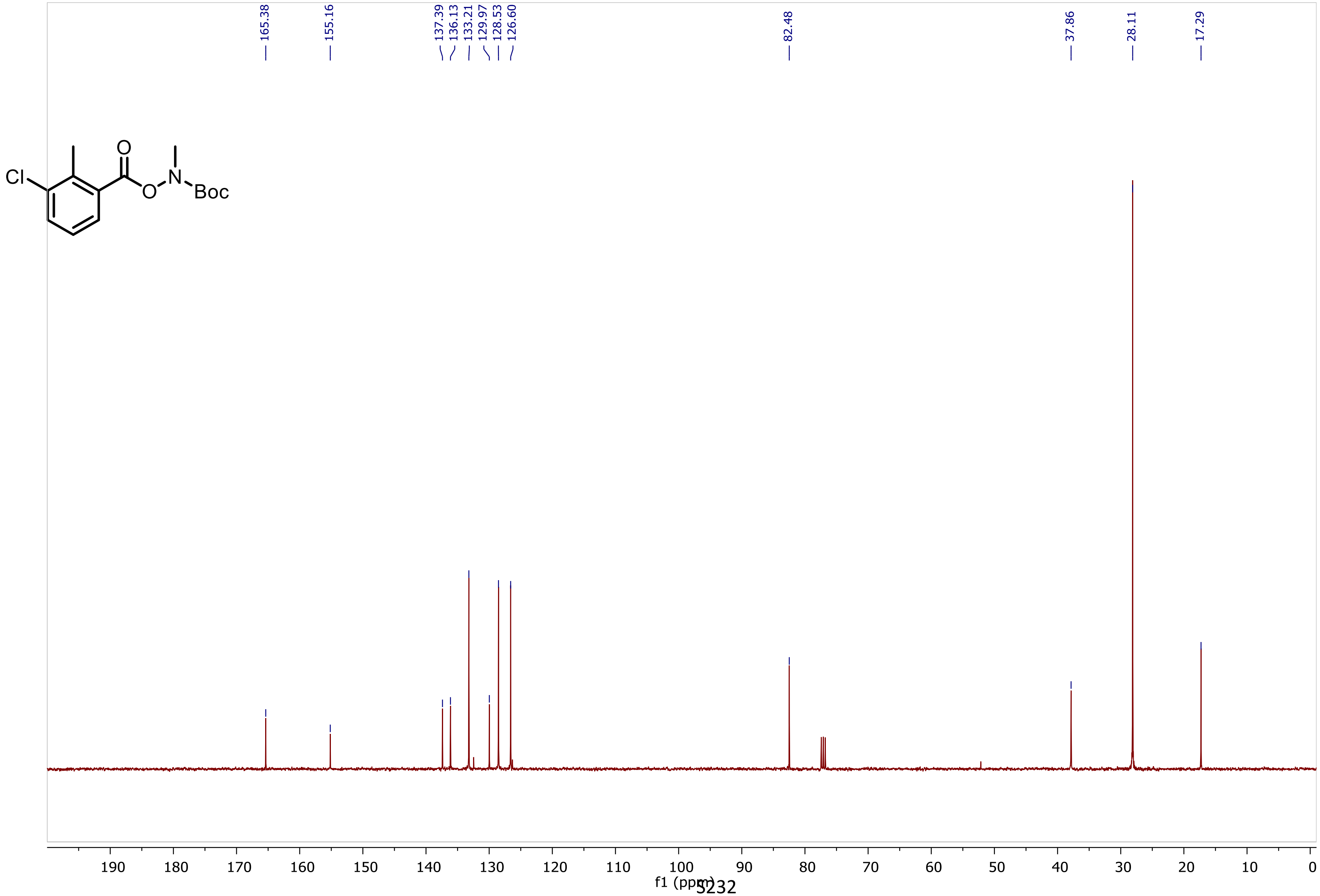

^{1}H NMR spectrum of *tert*-butyl methyl((3,4-dimethylbenzoyl)oxy)carbamate **3h** in $CDCl_3$

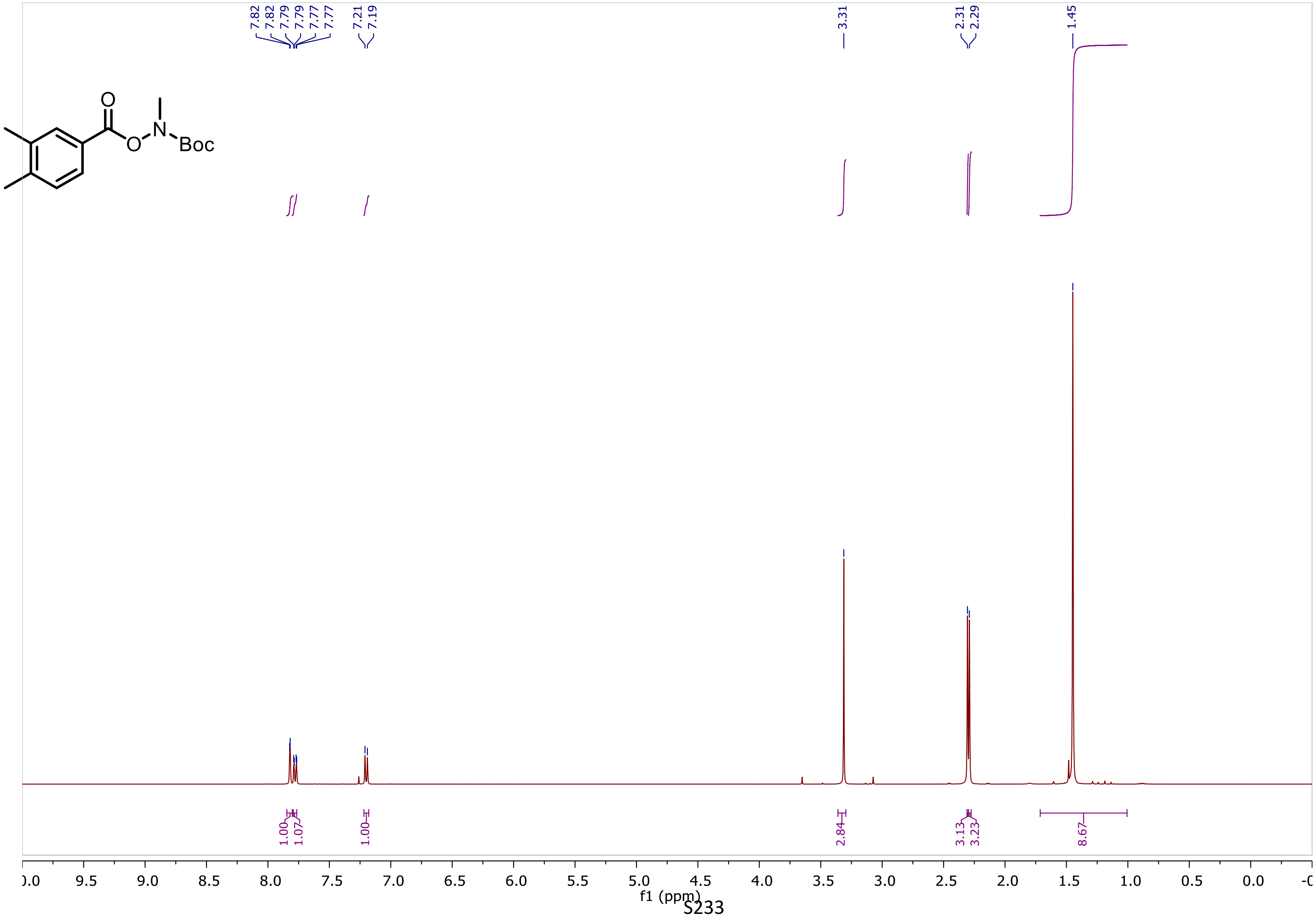

^{13}C NMR spectrum of *tert*-butyl methyl((3,4-dimethylbenzoyl)oxy)carbamate **3h** in $CDCl_3$

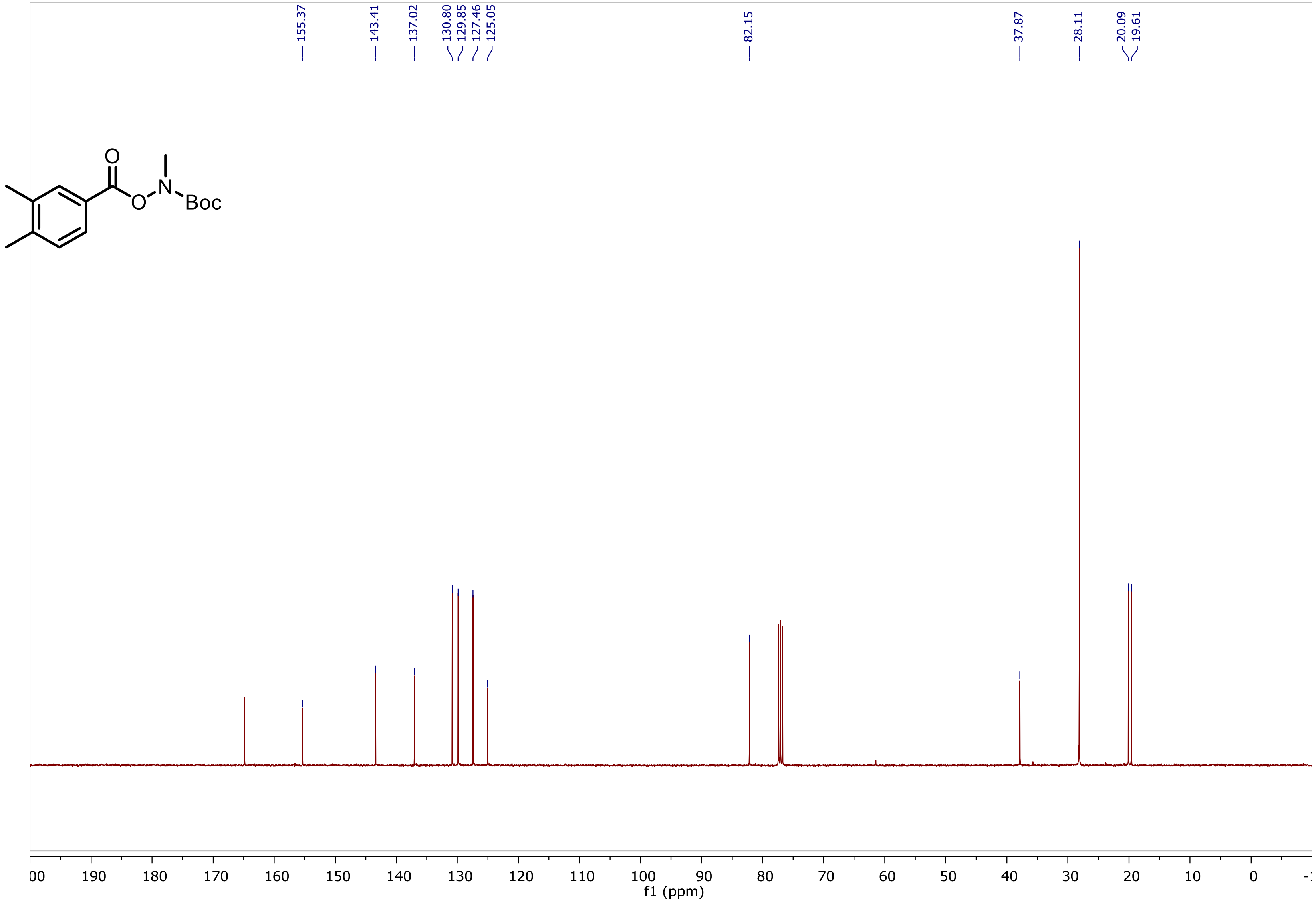

^{1}H NMR spectrum of *tert*-butyl methyl((3,5-dimethylbenzoyl)oxy)carbamate **3i** in $CDCl_3$

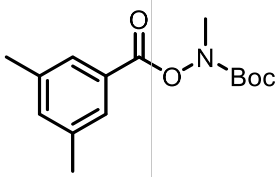

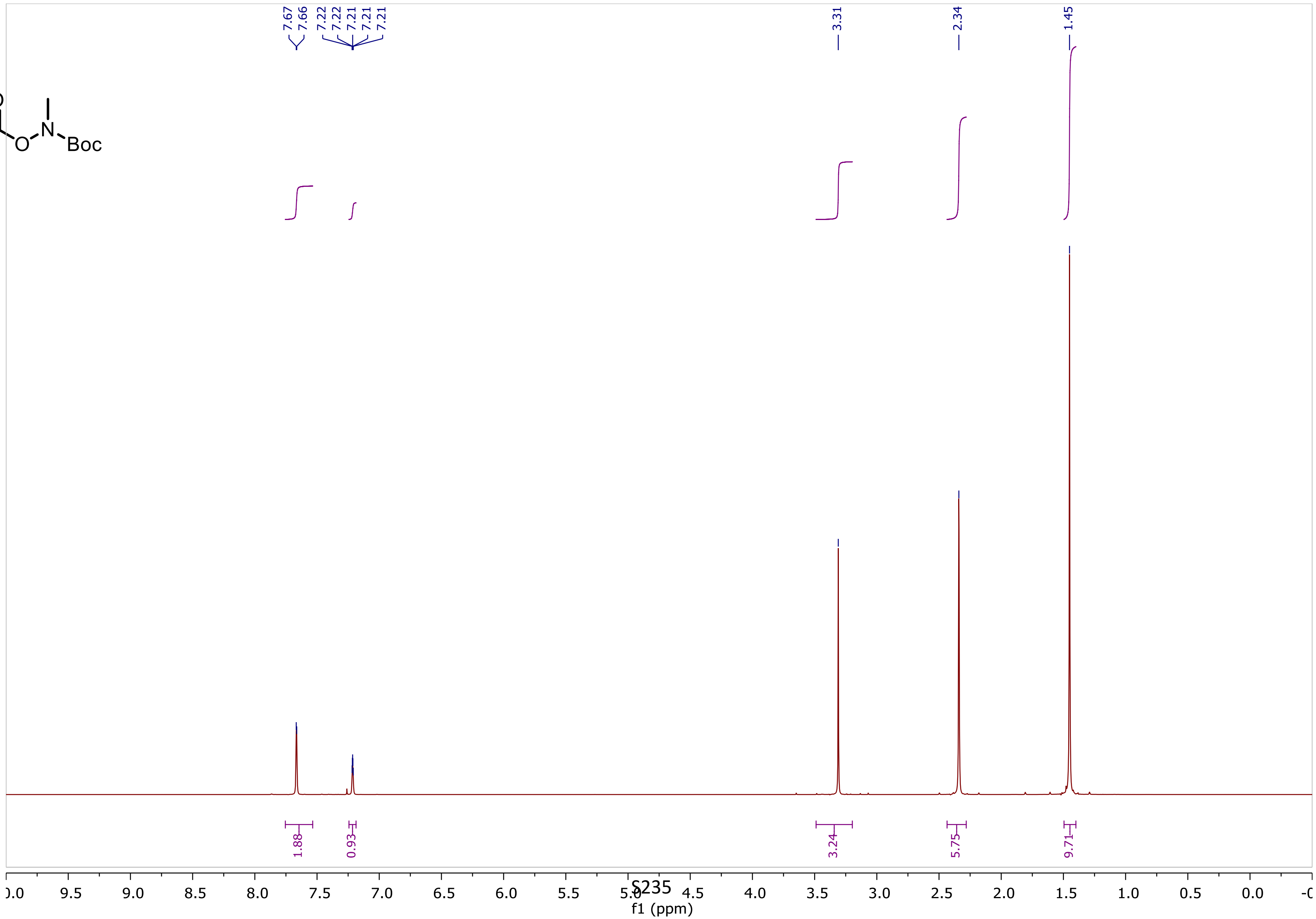

^{13}C NMR spectrum of *tert*-butyl methyl((3,5-dimethylbenzoyl)oxy)carbamate **3i** in $CDCl_3$

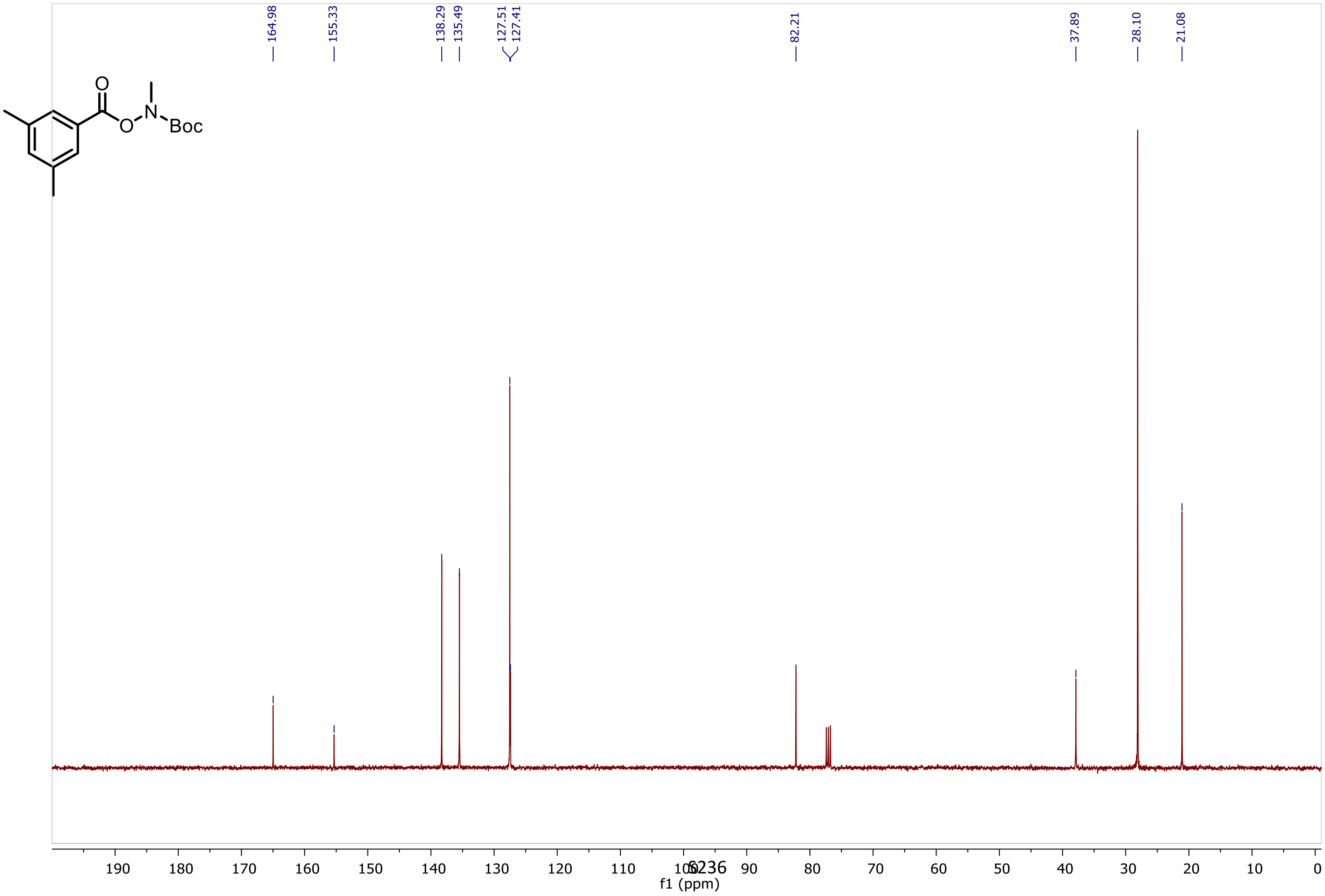

^{1}H NMR spectrum of *tert*-butyl methyl((2-chlorobenzoyl)oxy)carbamate in $CDCl_3$

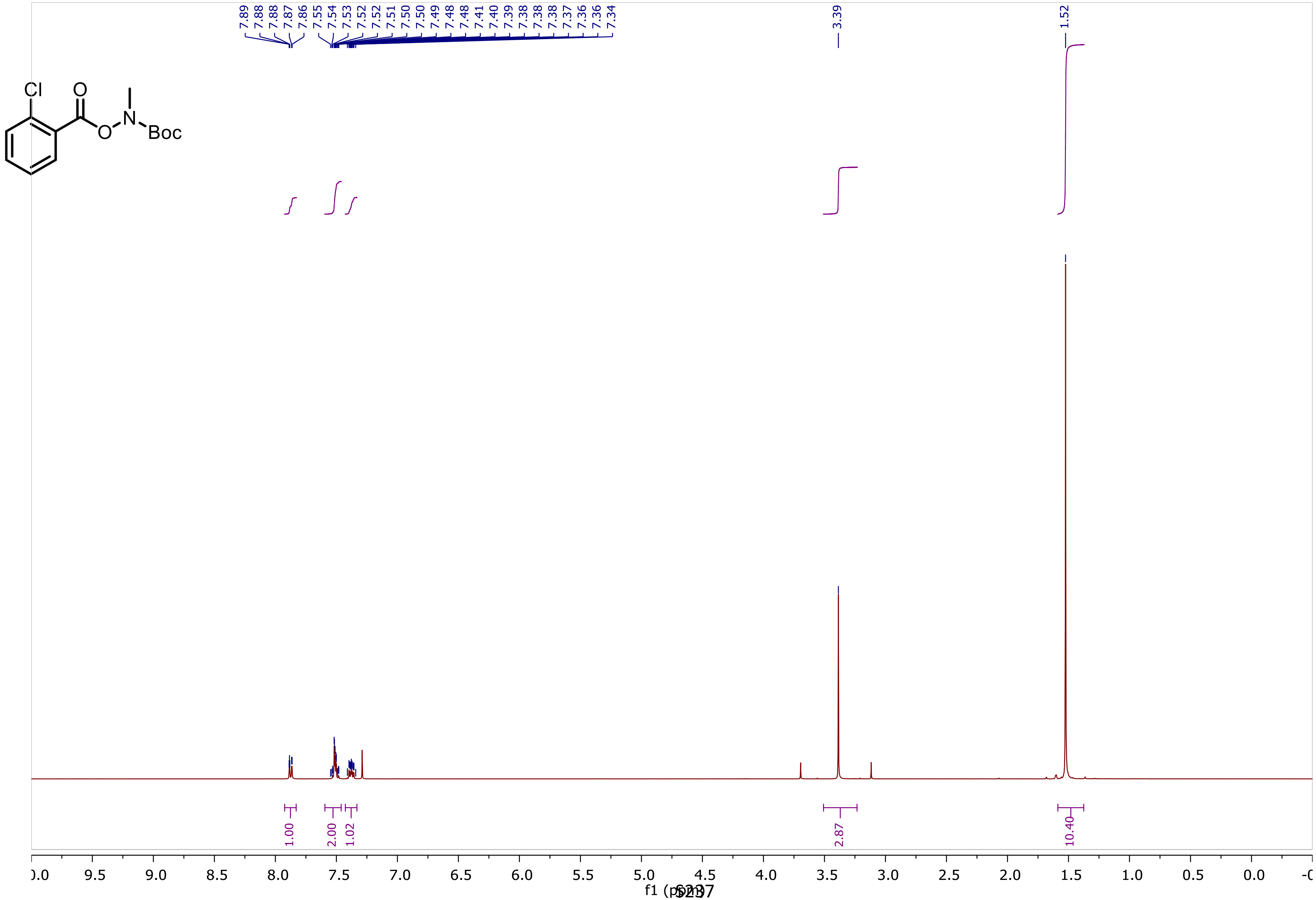

^{13}C NMR spectrum of *tert*-butyl methyl((2-chlorobenzoyl)oxy)carbamate in $CDCl_3$

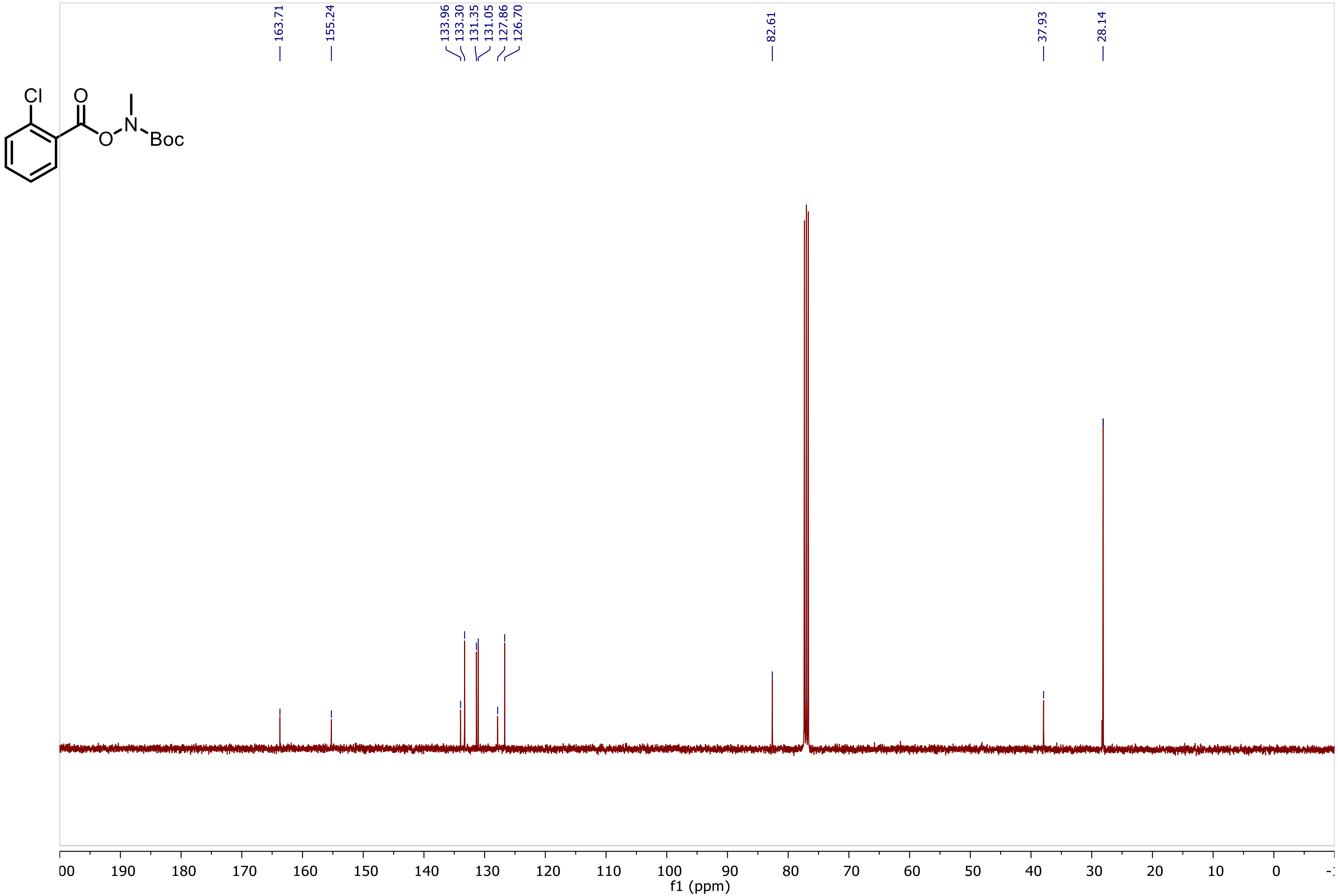

^1H NMR spectrum of *tert*-butyl methyl((3-fluorobenzoyl)oxy)carbamate in $CDCl_3$

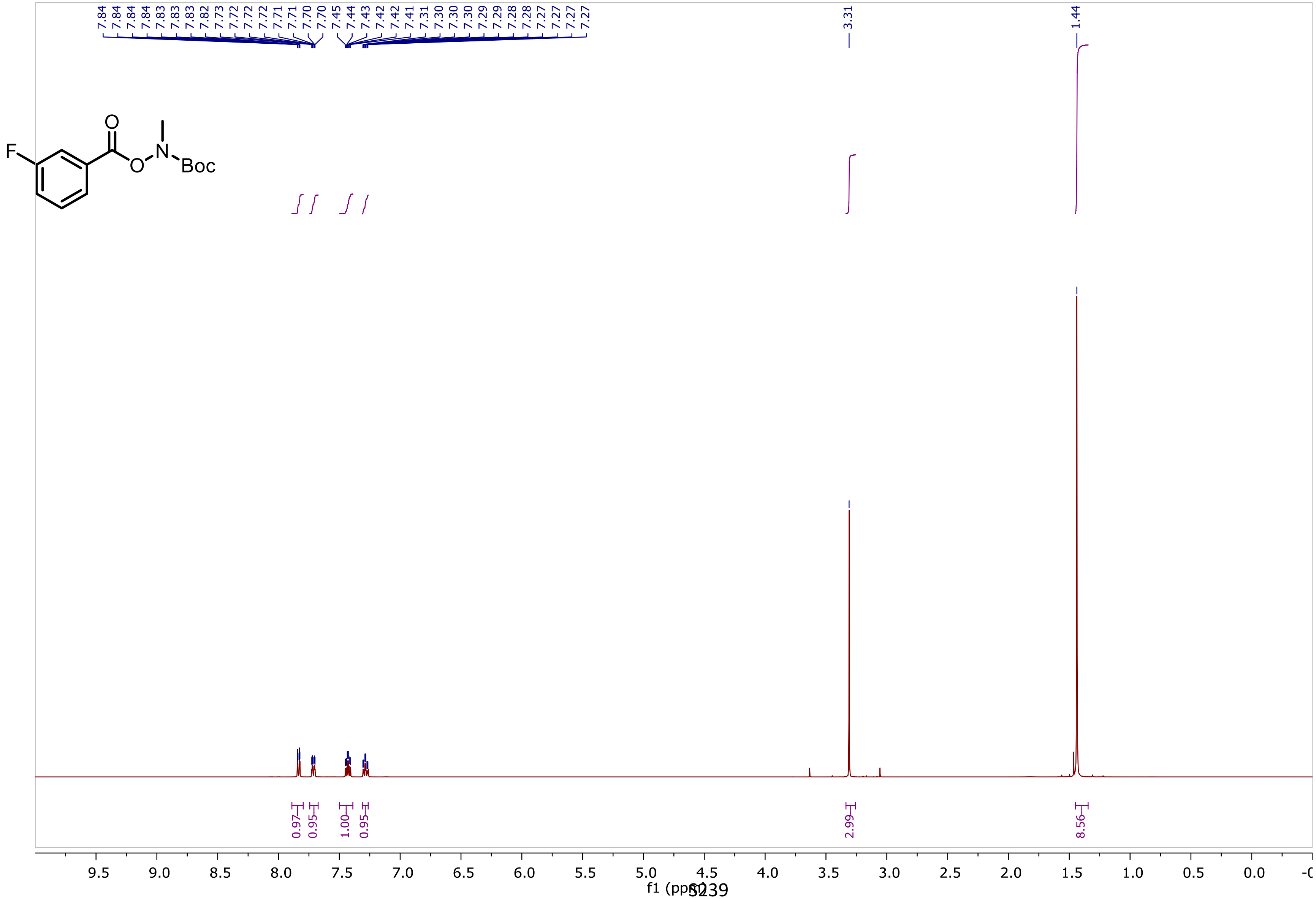

^{13}C NMR spectrum of *tert*-butyl methyl((3-fluorobenzoyl)oxy)carbamate in $CDCl_3$

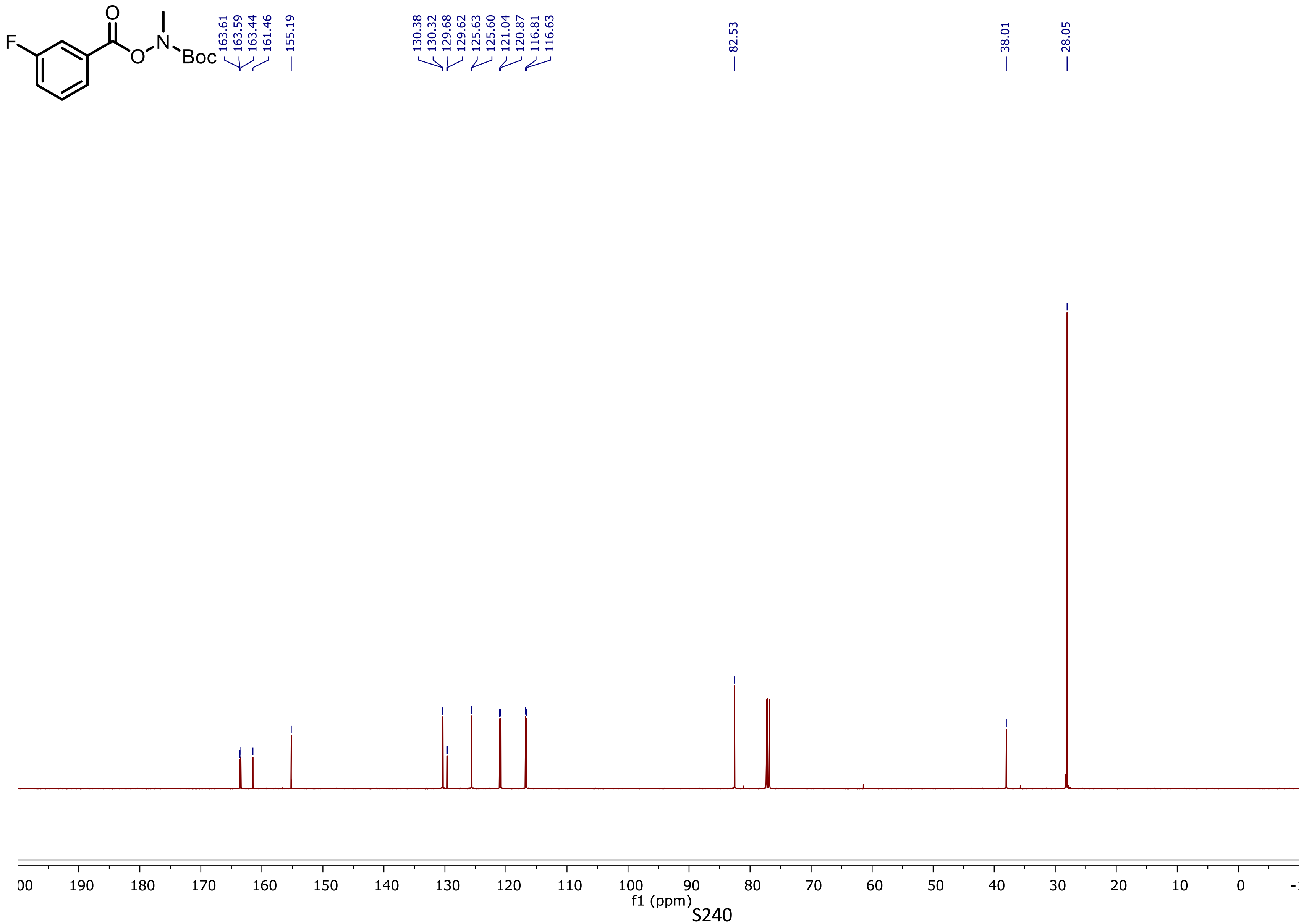

^{19}F NMR spectrum of *tert*-butyl methyl((3-fluorobenzoyl)oxy)carbamate in $CDCl_3$

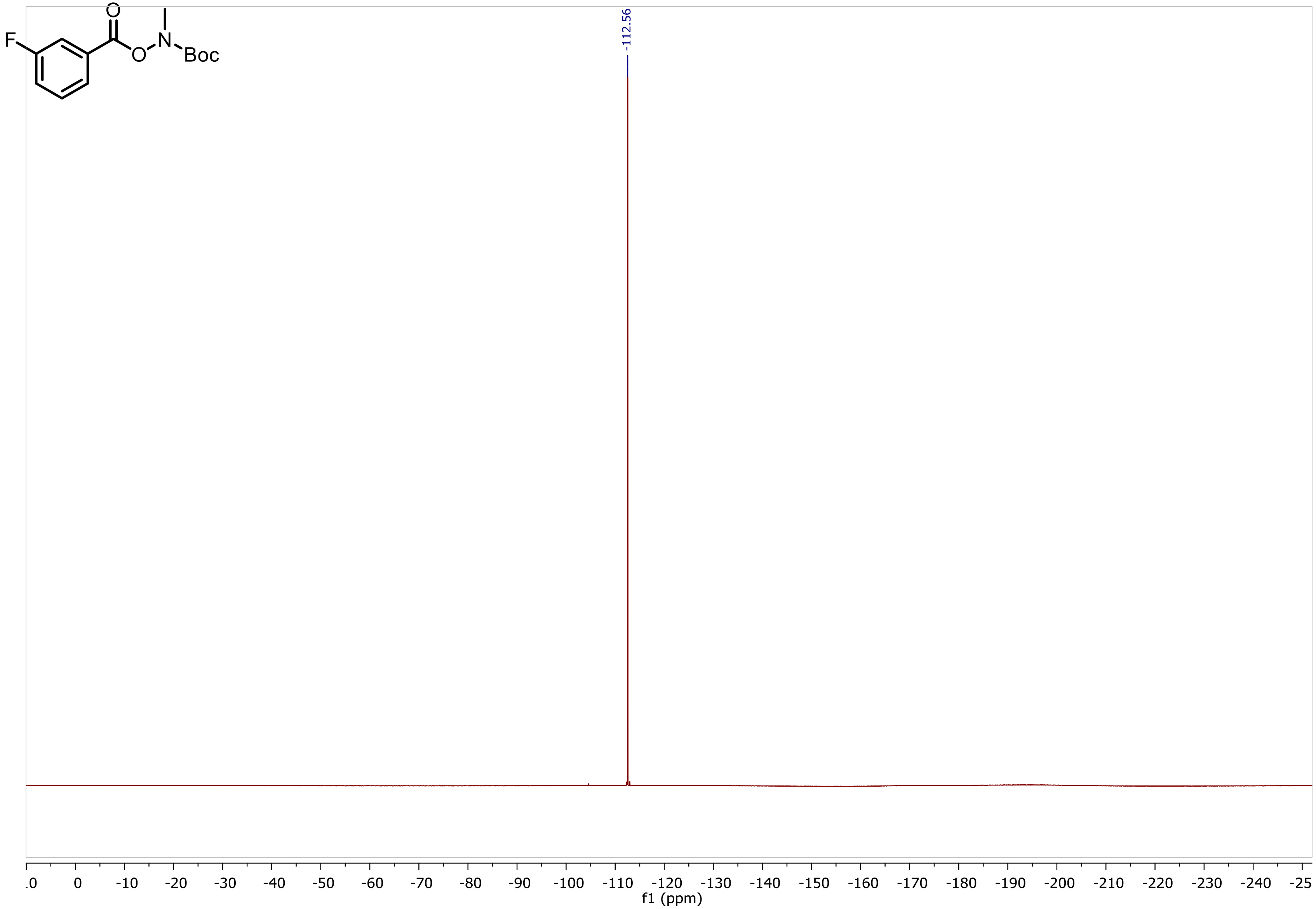

^{1}H NMR spectrum of *tert*-butyl methyl((3-bromobenzoyl)oxy)carbamate in $CDCl_3$

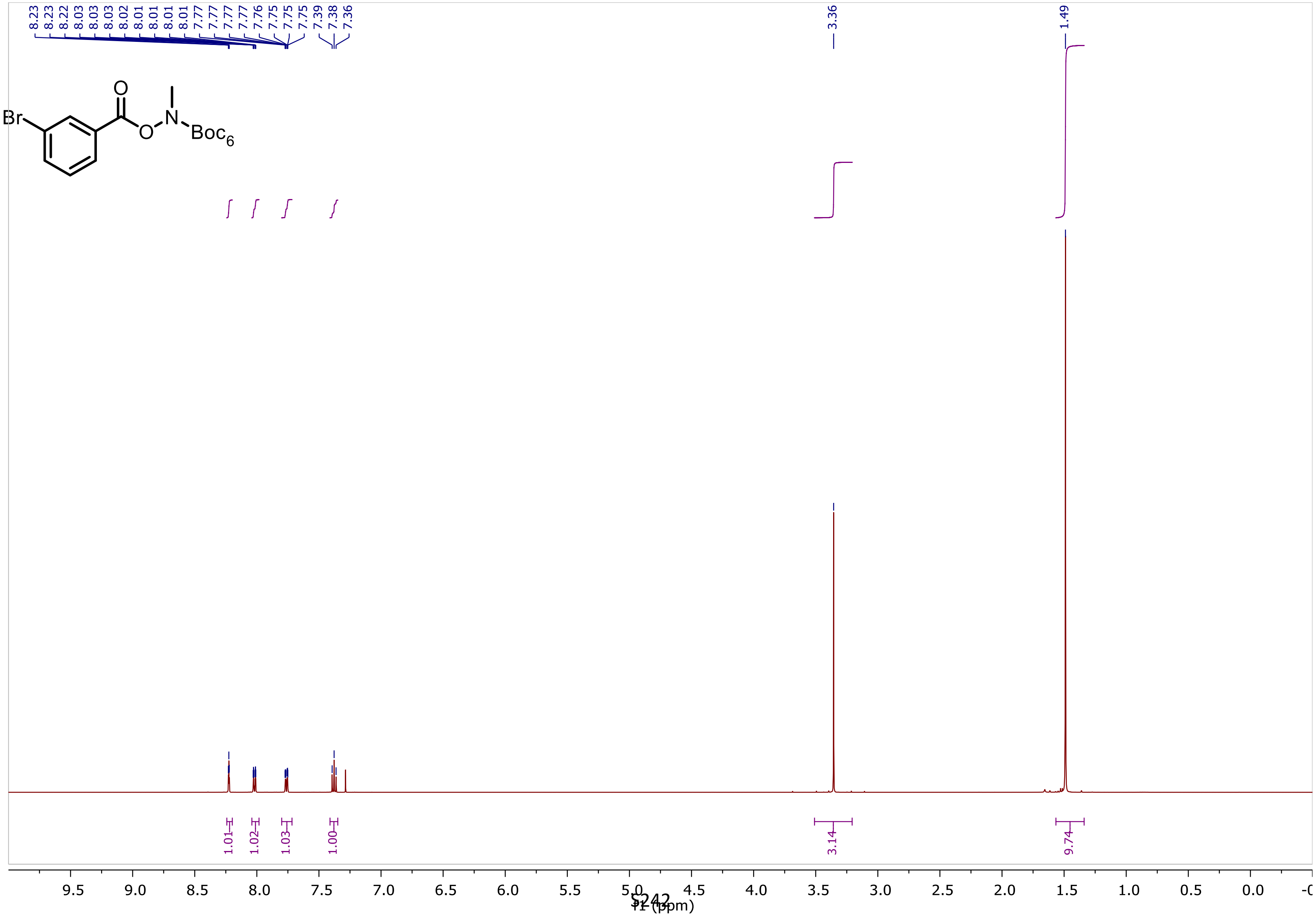

^{13}C NMR spectrum of *tert*-butyl methyl((3-bromobenzoyl)oxy)carbamate in $CDCl_3$

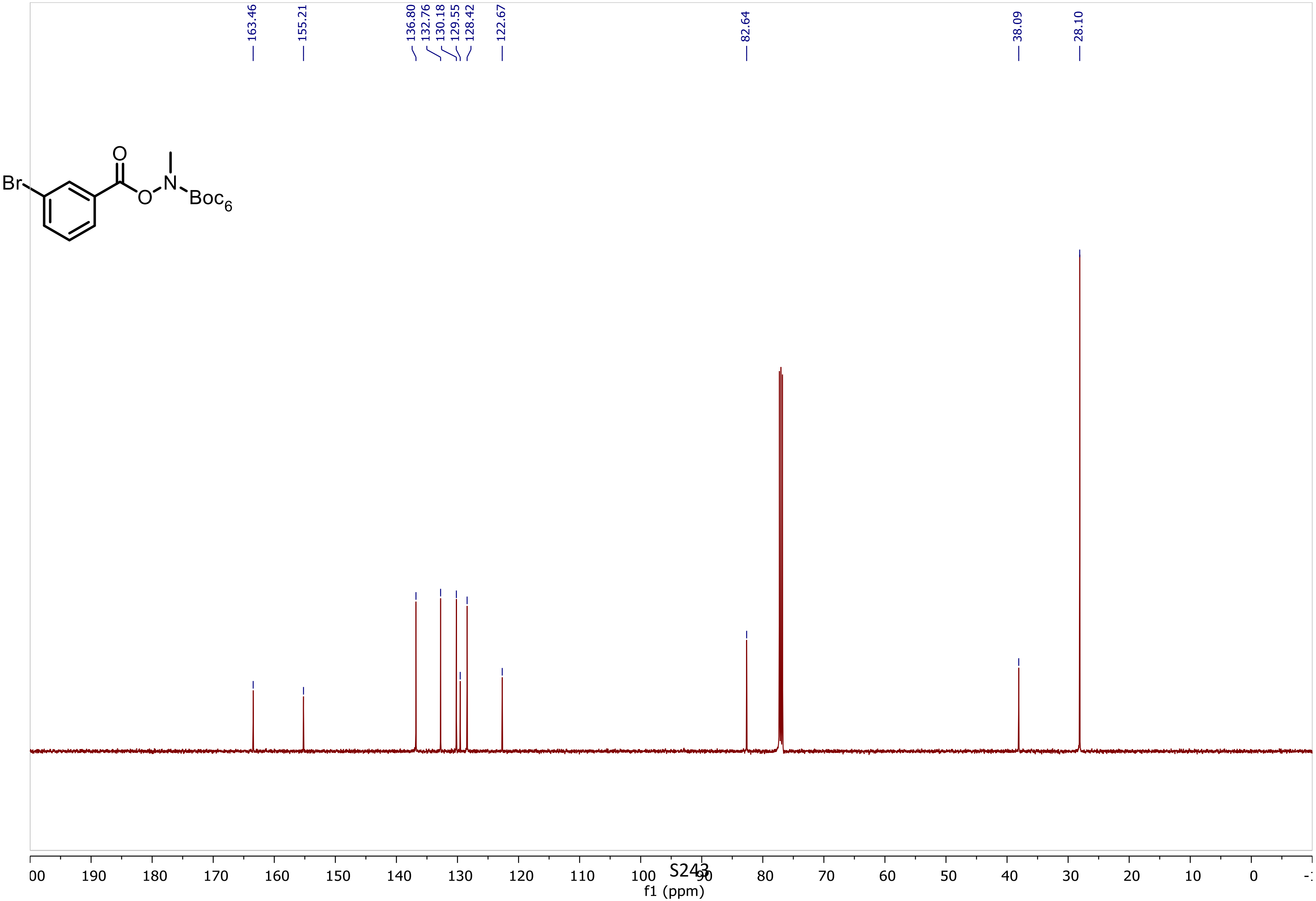

^{1}H NMR spectrum of *tert*-butyl methyl((3,5-difluorobenzoyl)oxy)carbamate in $CDCl_3$

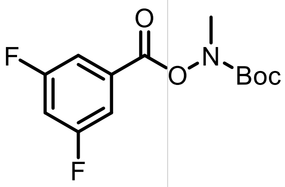

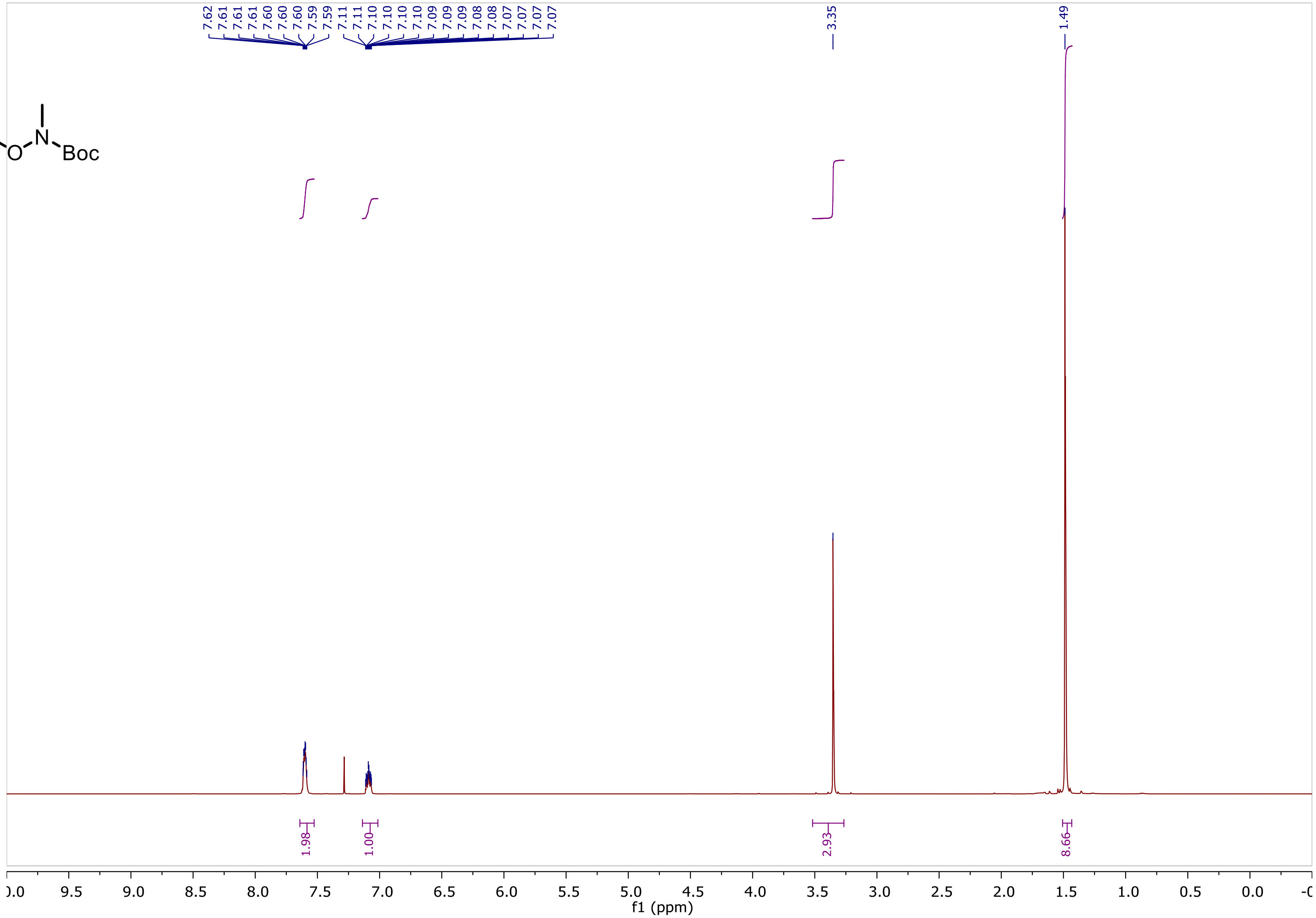

^{13}C NMR spectrum of *tert*-butyl methyl((3,5-difluorobenzoyl)oxy)carbamate in $CDCl_3$

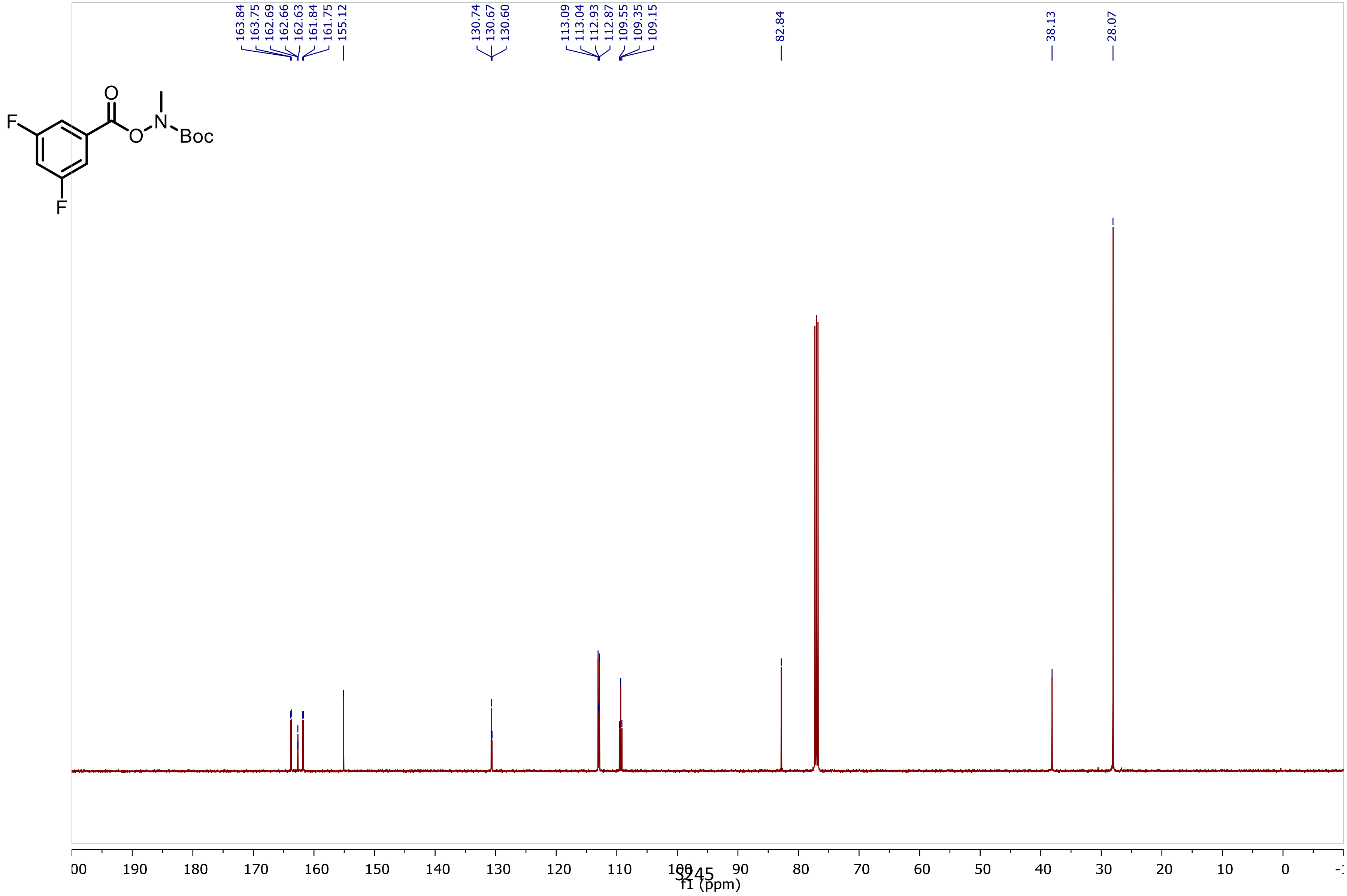

^{19}F NMR spectrum of *tert*-butyl methyl((3,5-difluorobenzoyl)oxy)carbamate in $CDCl_3$

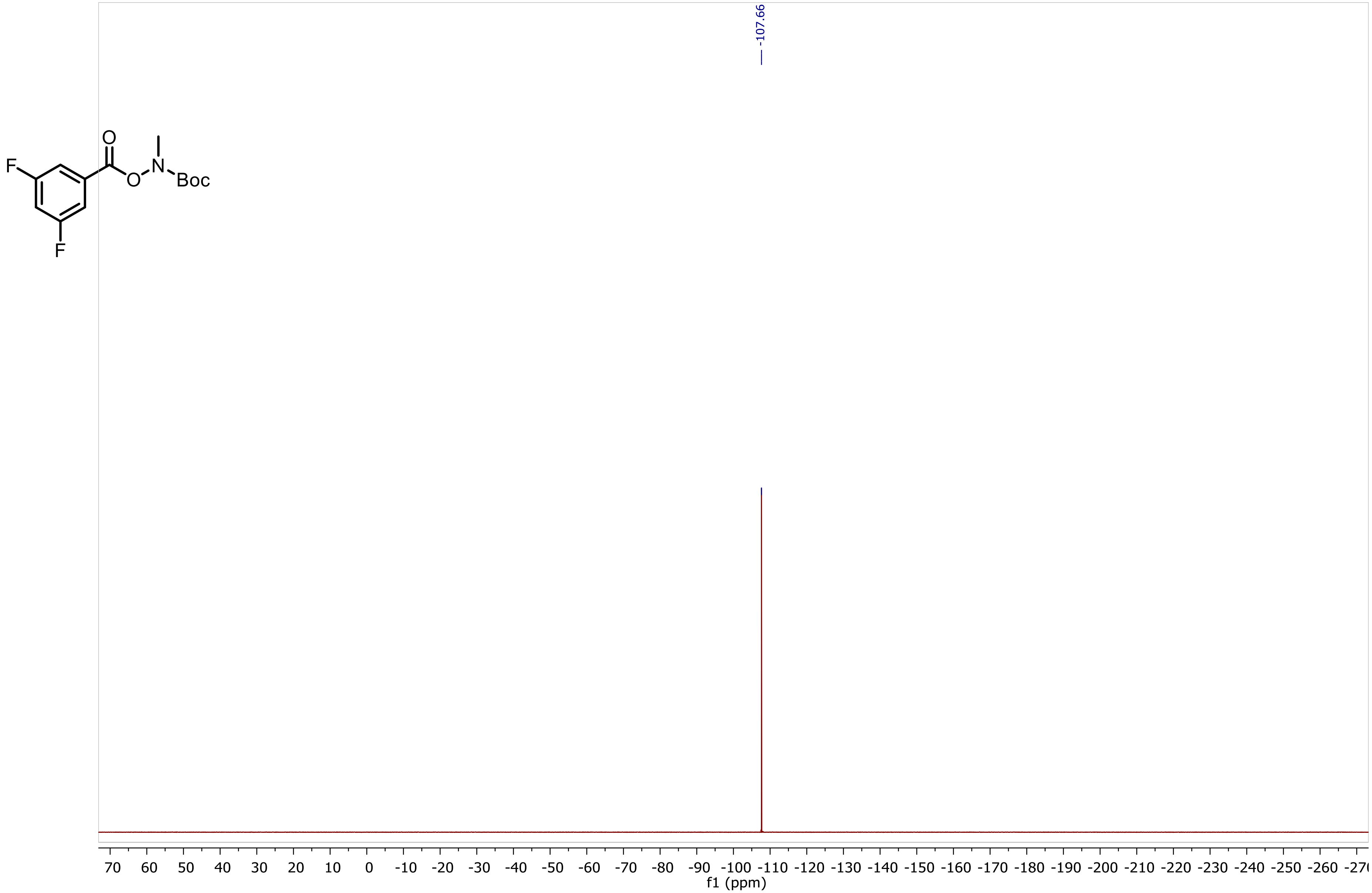

^{1}H NMR spectrum of *tert*-butyl (2-phenylacetoxy)carbamate **5a** in $CDCl_3$

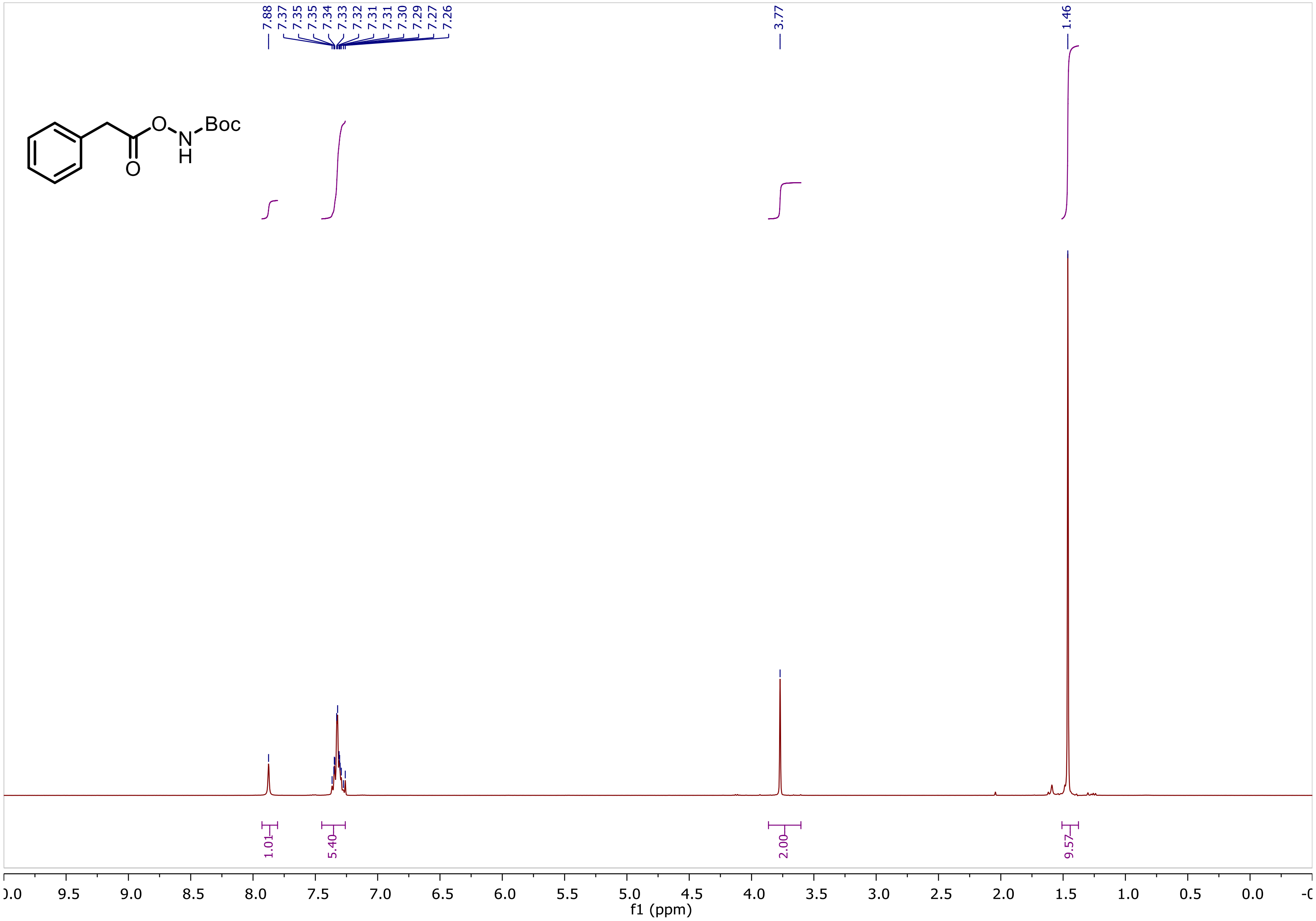

^{13}C NMR spectrum of *tert*-butyl (2-phenylacetoxy)carbamate **5a** in $CDCl_3$

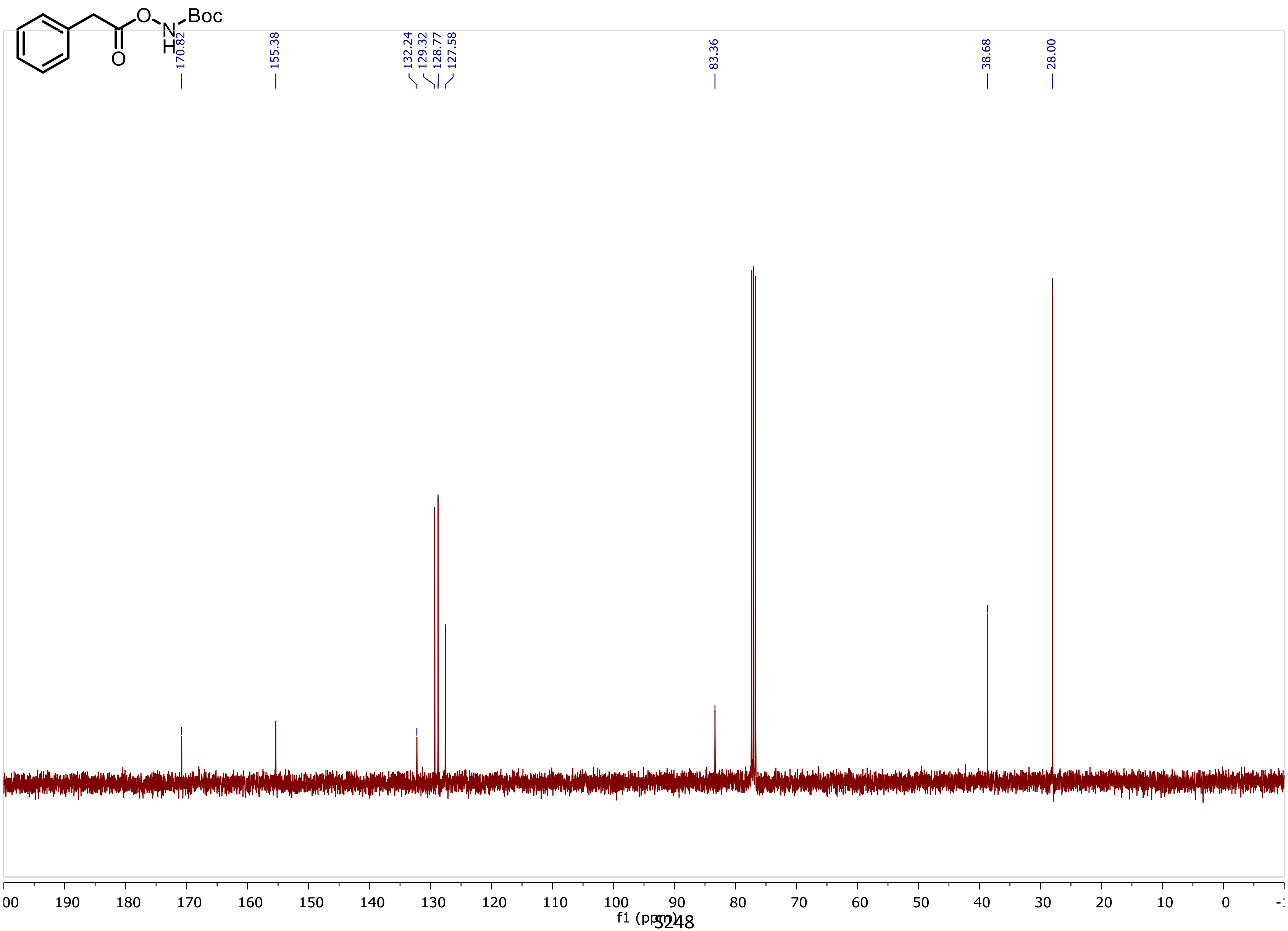

^{1}H NMR spectrum of tert-butyl ((2-phenylpropanoyl)oxy)carbamate **5b** in $CDCl_3$

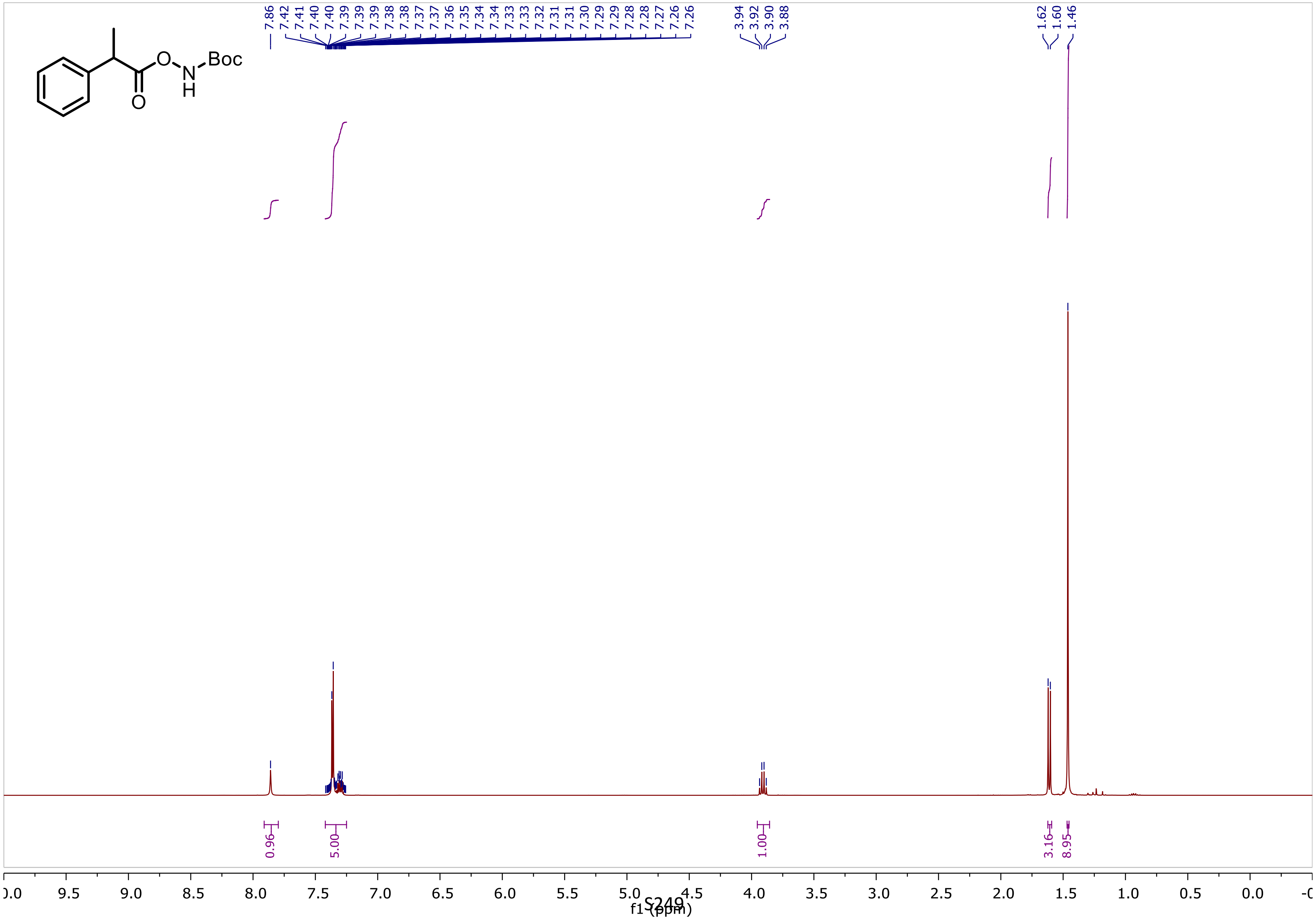

^{13}C NMR spectrum of tert-butyl ((2-phenylpropanoyl)oxy)carbamate **5b** in $CDCl_3$

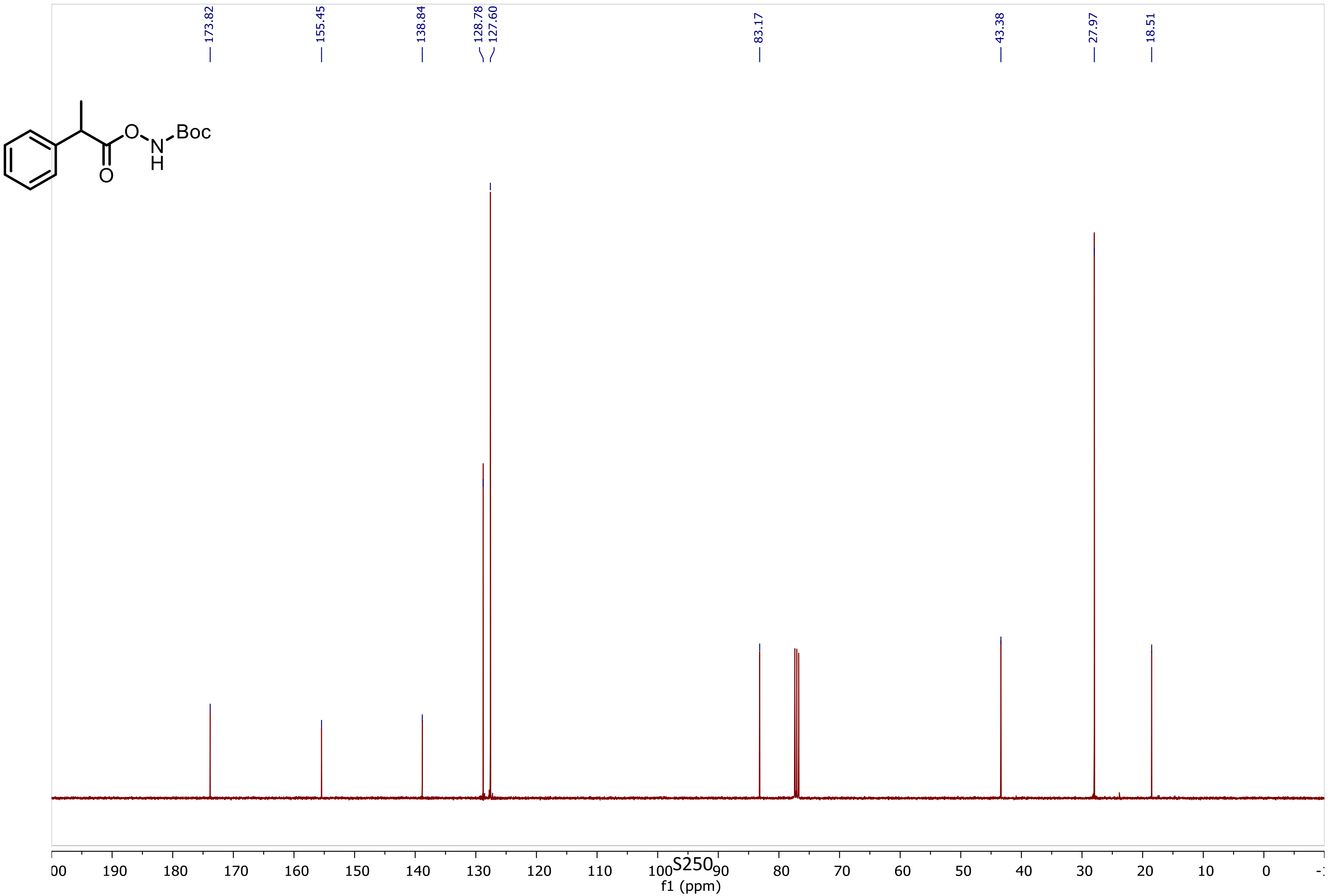

^{1}H NMR spectrum of *tert*-butyl ((2-methyl-2-phenylpropanoyl)oxy)carbamate **5c** in $CDCl_3$

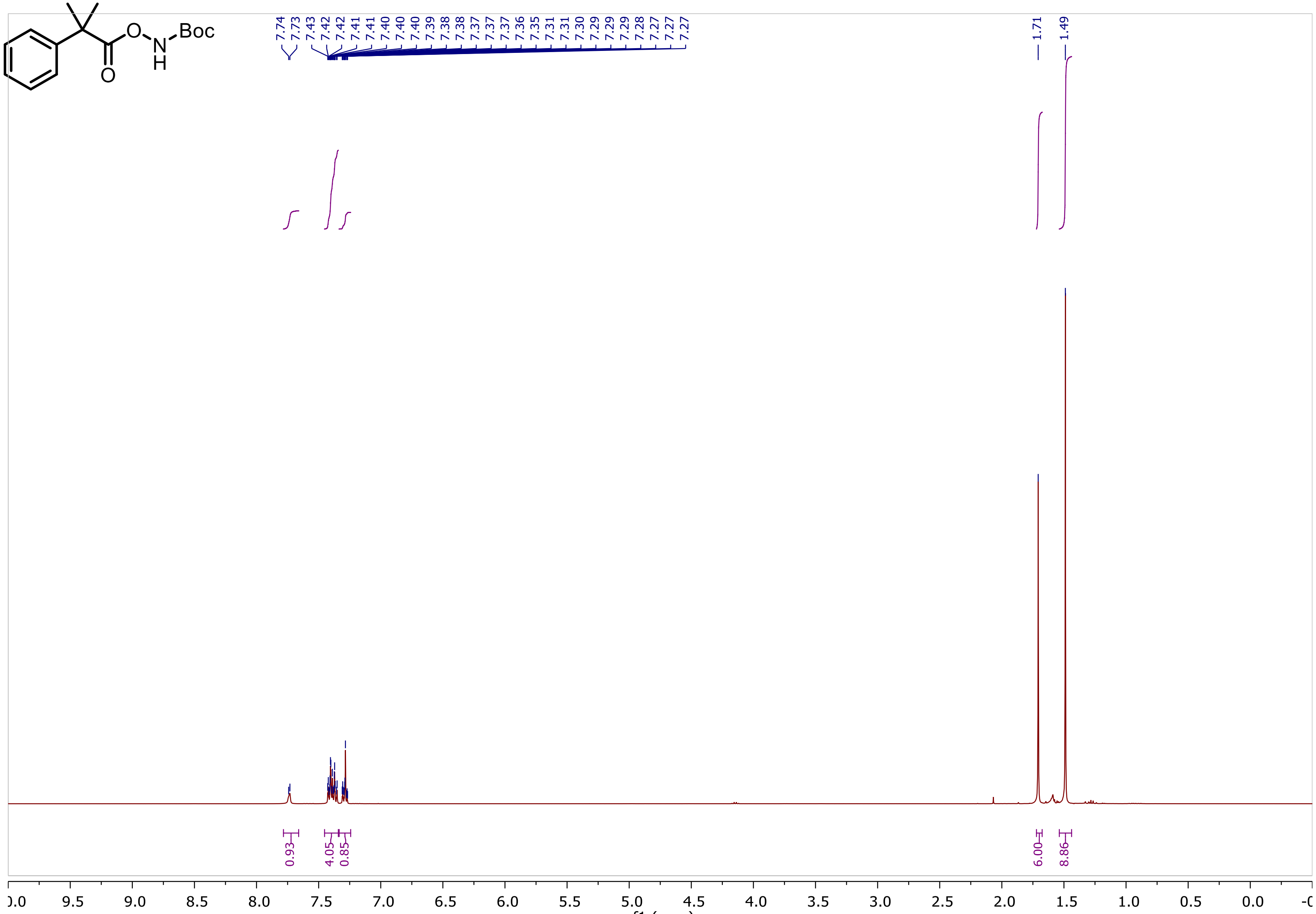

^{13}C NMR spectrum of *tert*-butyl ((2-methyl-2-phenylpropanoyl)oxy)carbamate **5c** in $CDCl_3$

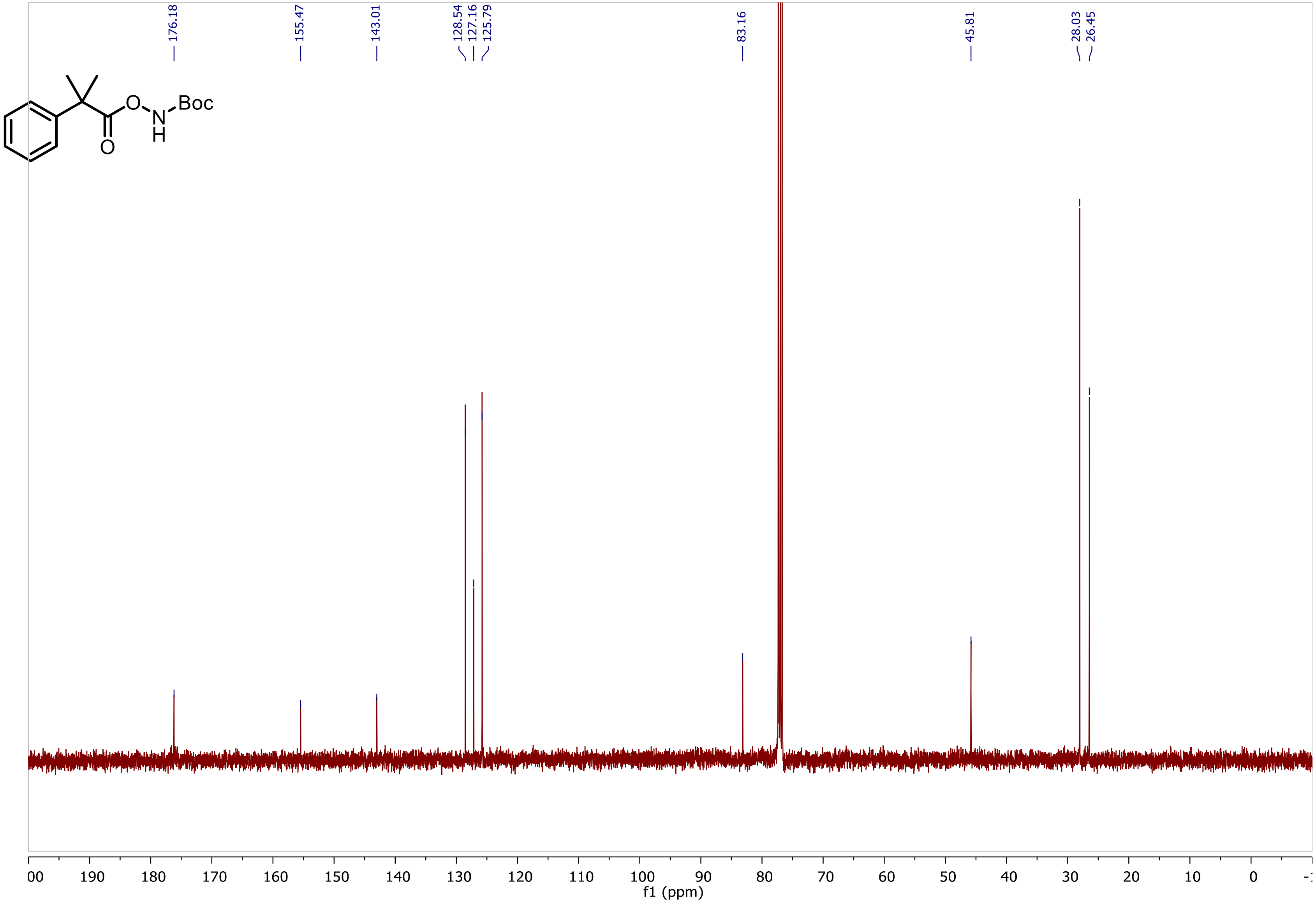

^{1}H NMR spectrum of *tert*-butyl ((1-phenylcyclopropane-1-carbonyl)oxy)carbamate **5d** in $CDCl_3$

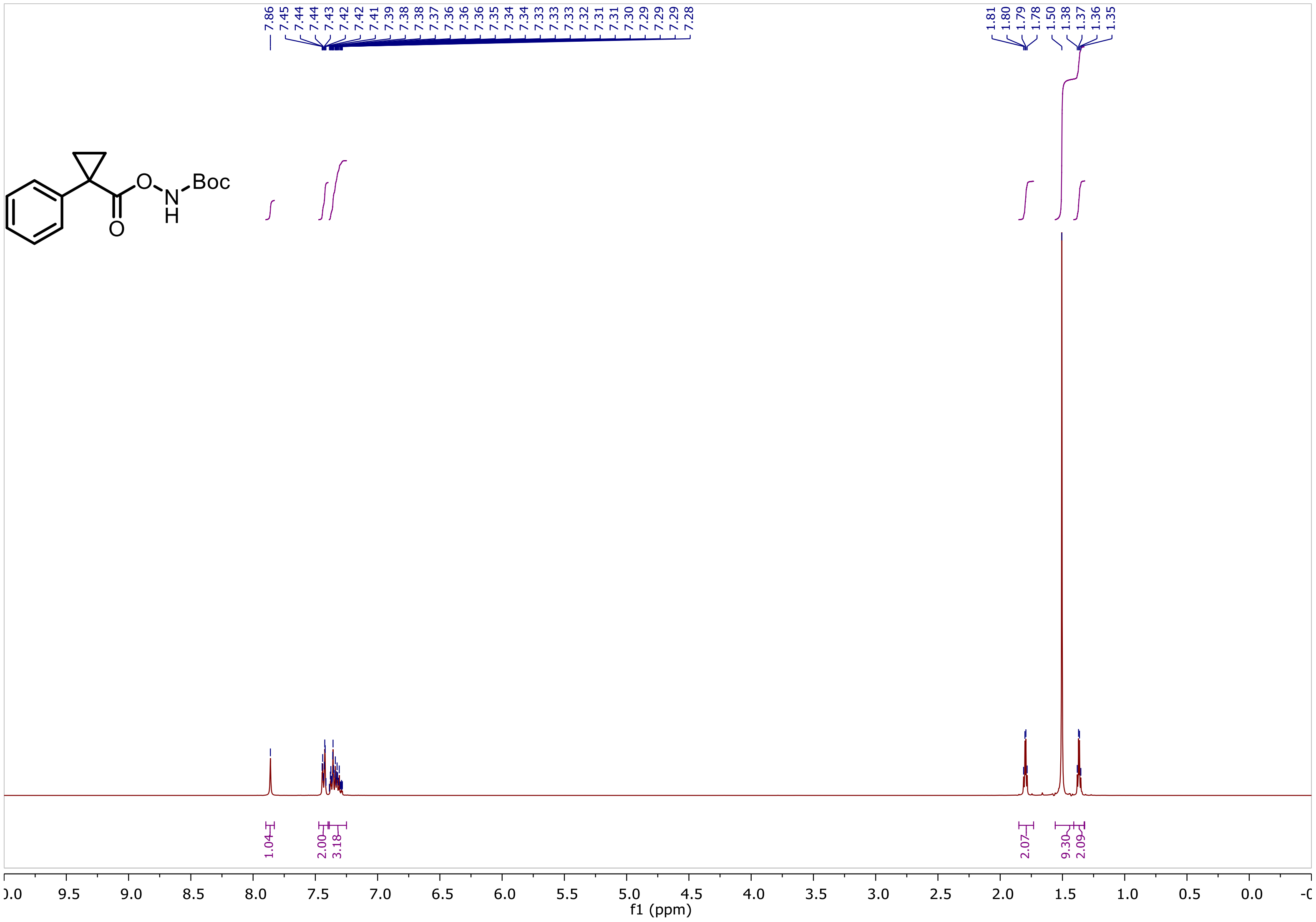

^{13}C NMR spectrum of *tert*-butyl ((1-phenylcyclopropane-1-carbonyl)oxy)carbamate **5d** in $CDCl_3$

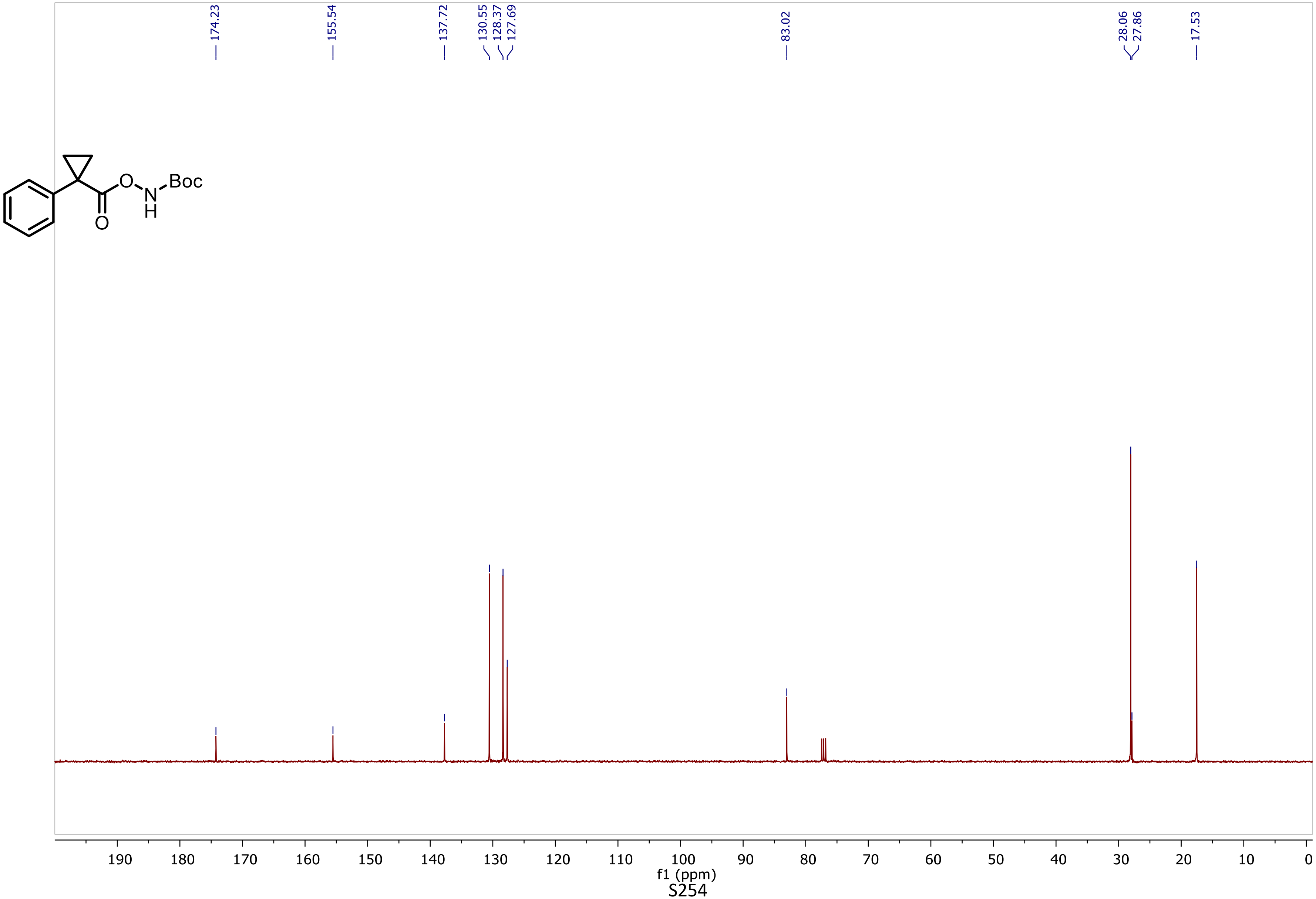

^{1}H NMR spectrum of *tert*-butyl (2-(o-tolyl)acetoxy)carbamate **5f** in $CDCl_3$

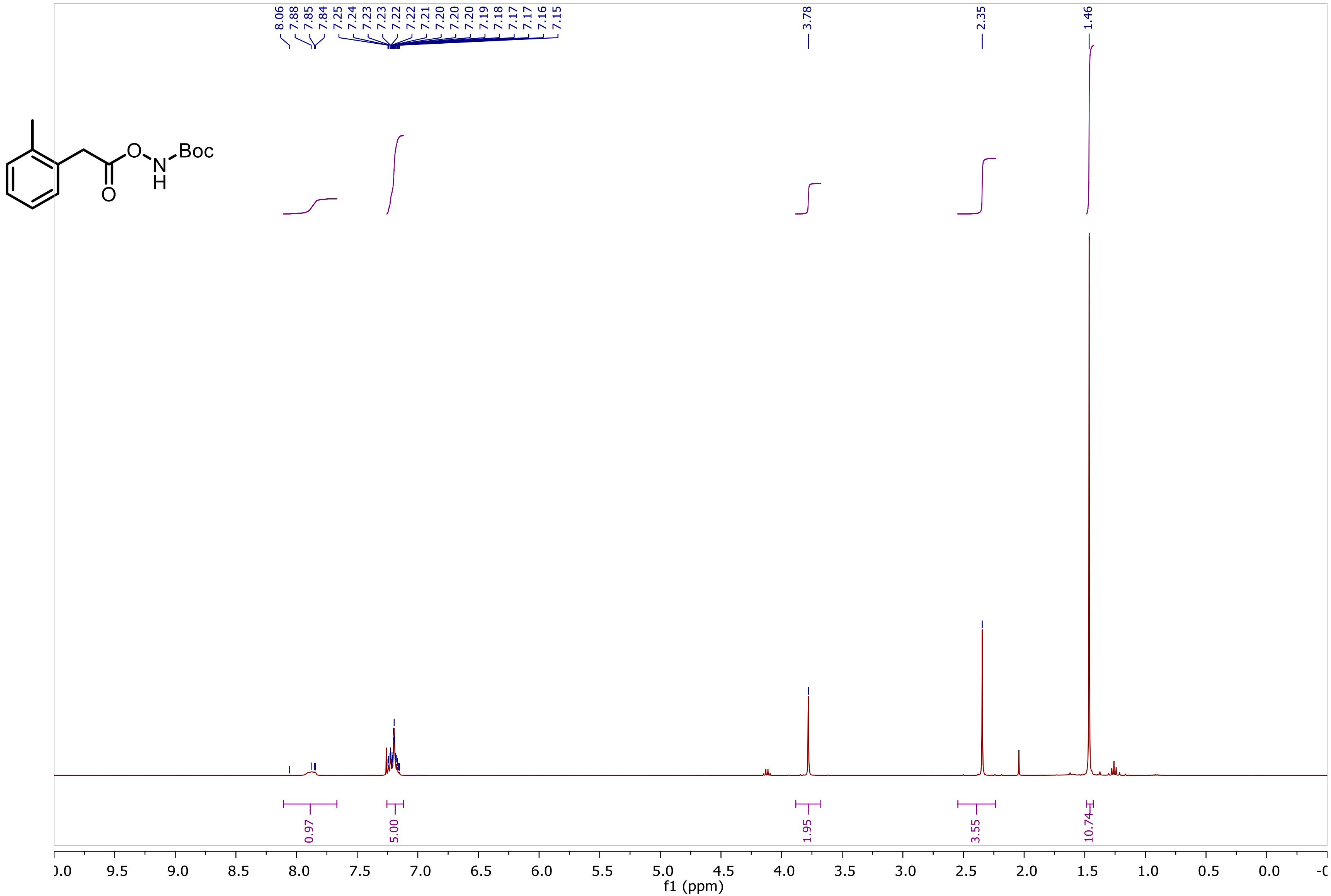

^{13}C NMR spectrum of *tert*-butyl (2-(o-tolyl)acetoxy)carbamate **5f** in $CDCl_3$

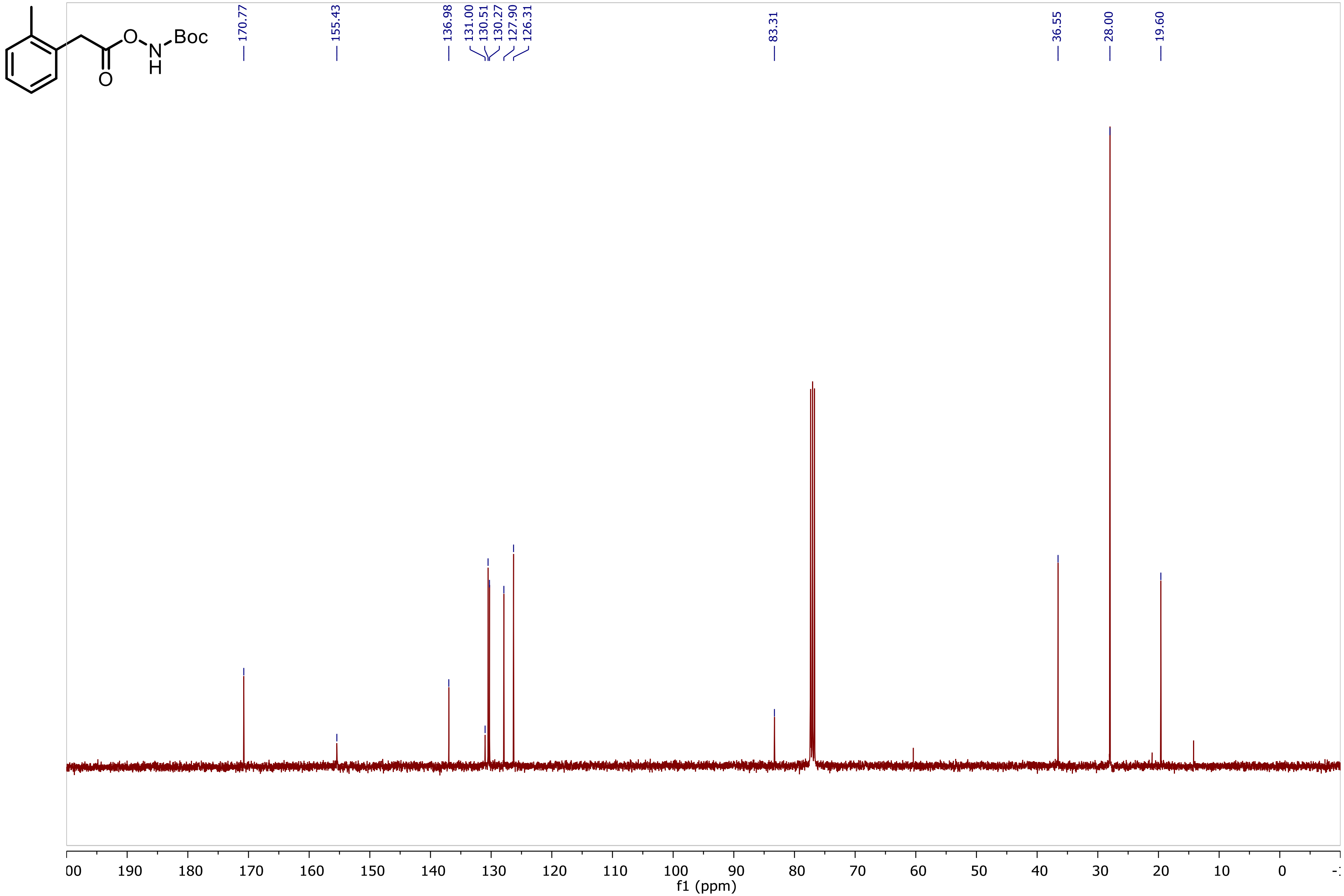

^{1}H NMR spectrum of *tert*-butyl (2-(2-chlorophenyl)acetoxy)carbamate **5e** in $CDCl_3$

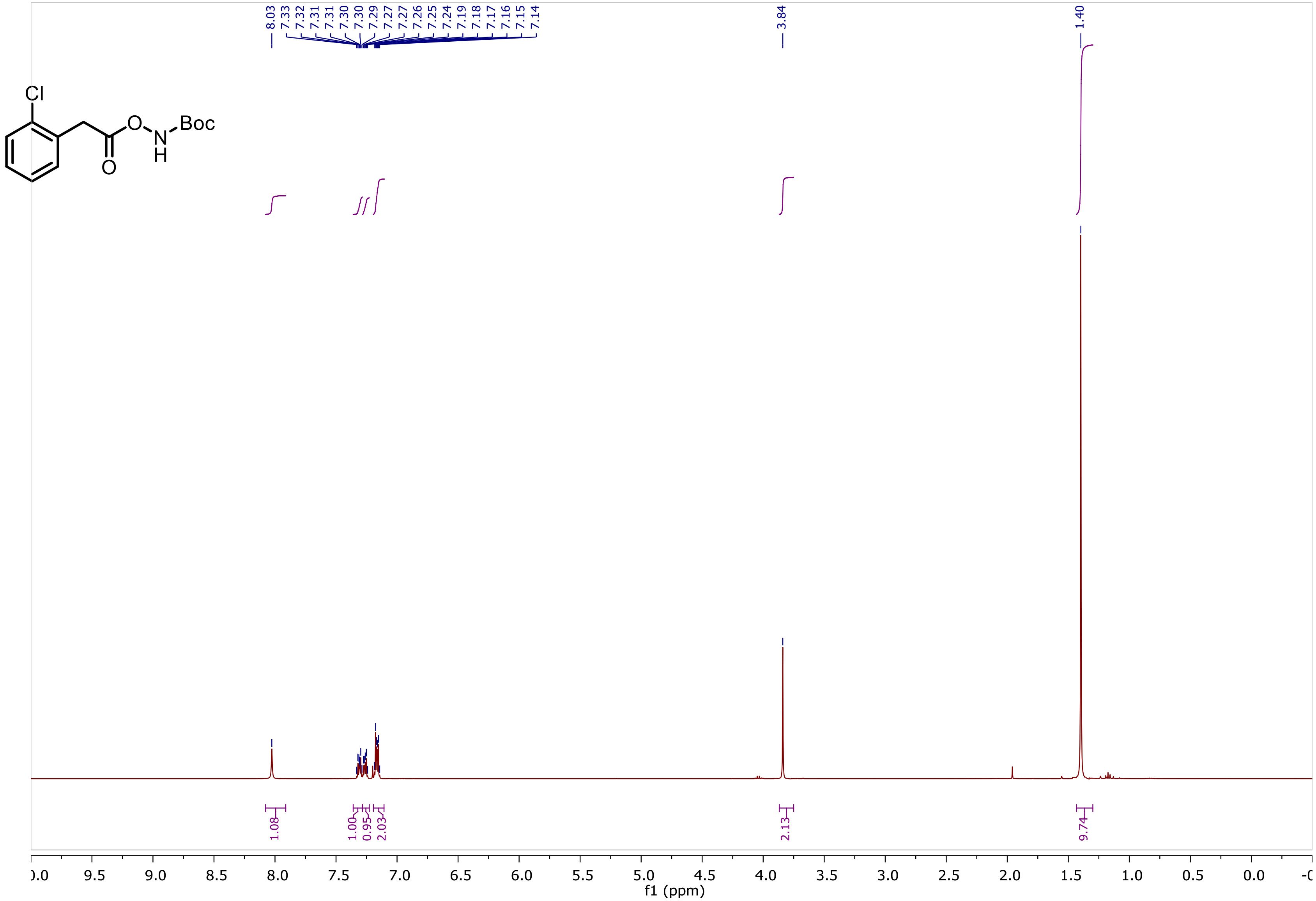

^{13}C NMR spectrum of *tert*-butyl (2-(2-chlorophenyl)acetoxy)carbamate **5e** in $CDCl_3$

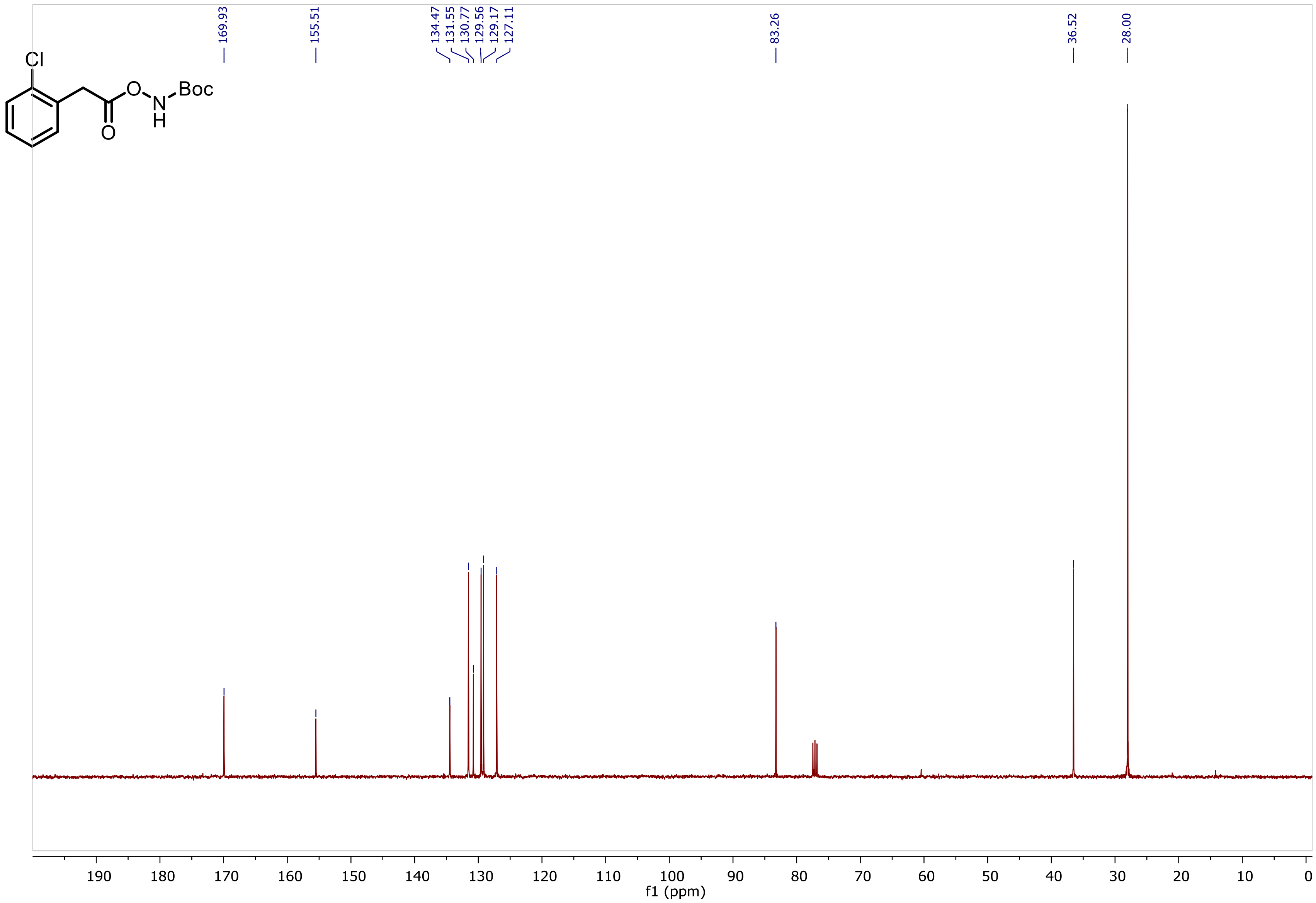

^{1}H NMR spectrum of *tert*-butyl (2-(2-(trifluoromethyl)phenyl)acetoxy)carbamate **5g** in $CDCl_3$

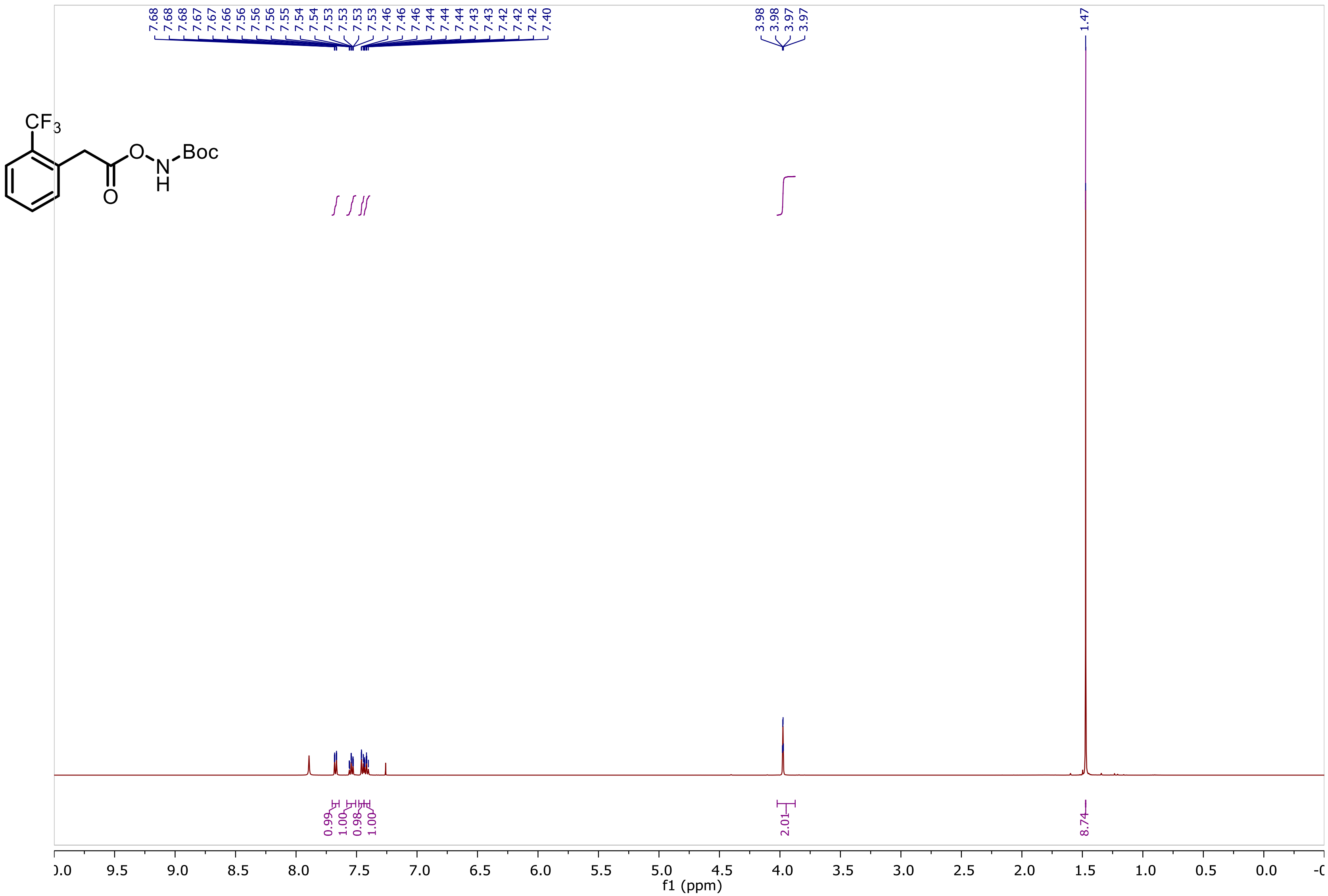

^{13}C NMR spectrum of *tert*-butyl (2-(2-(trifluoromethyl)phenyl)acetoxy)carbamate **5g** in $CDCl_3$

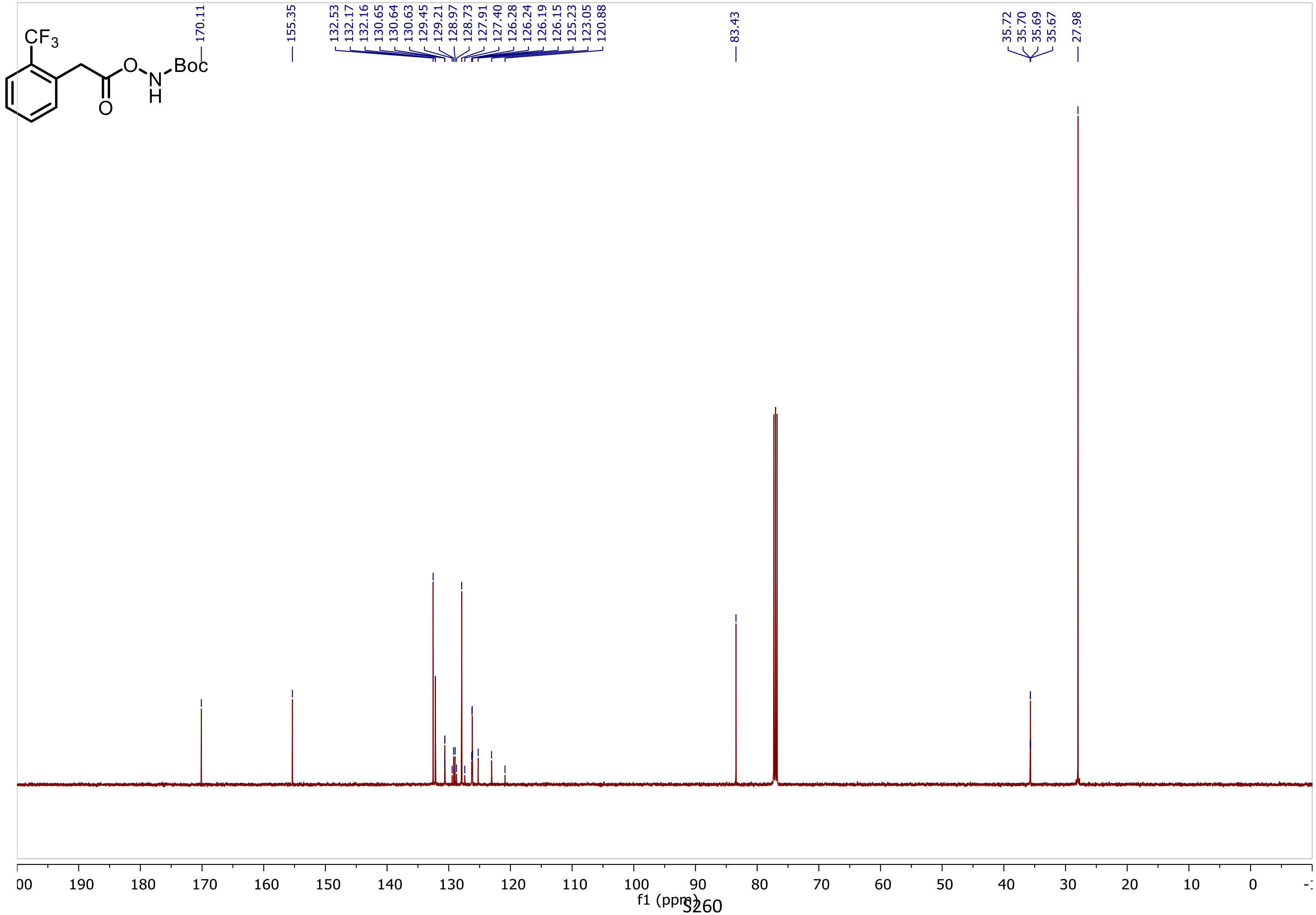

^{19}F NMR spectrum of *tert*-butyl (2-(2-(trifluoromethyl)phenyl)acetoxy)carbamate **5g** in $CDCl_3$

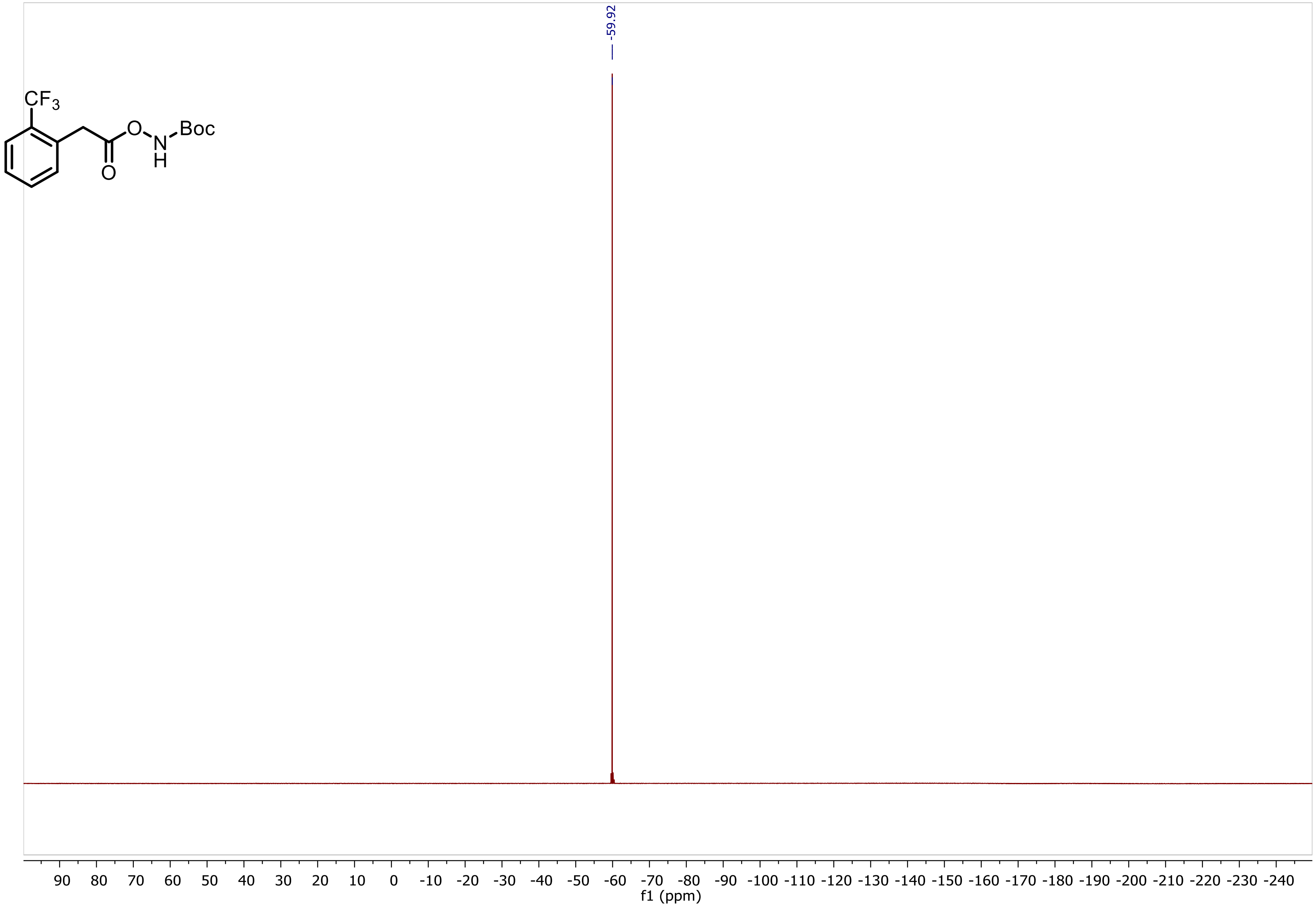

^{1}H NMR spectrum of *tert*-butyl (2-(3,5-dimethylphenyl)acetoxy)carbamate **5h** in $CDCl_3$

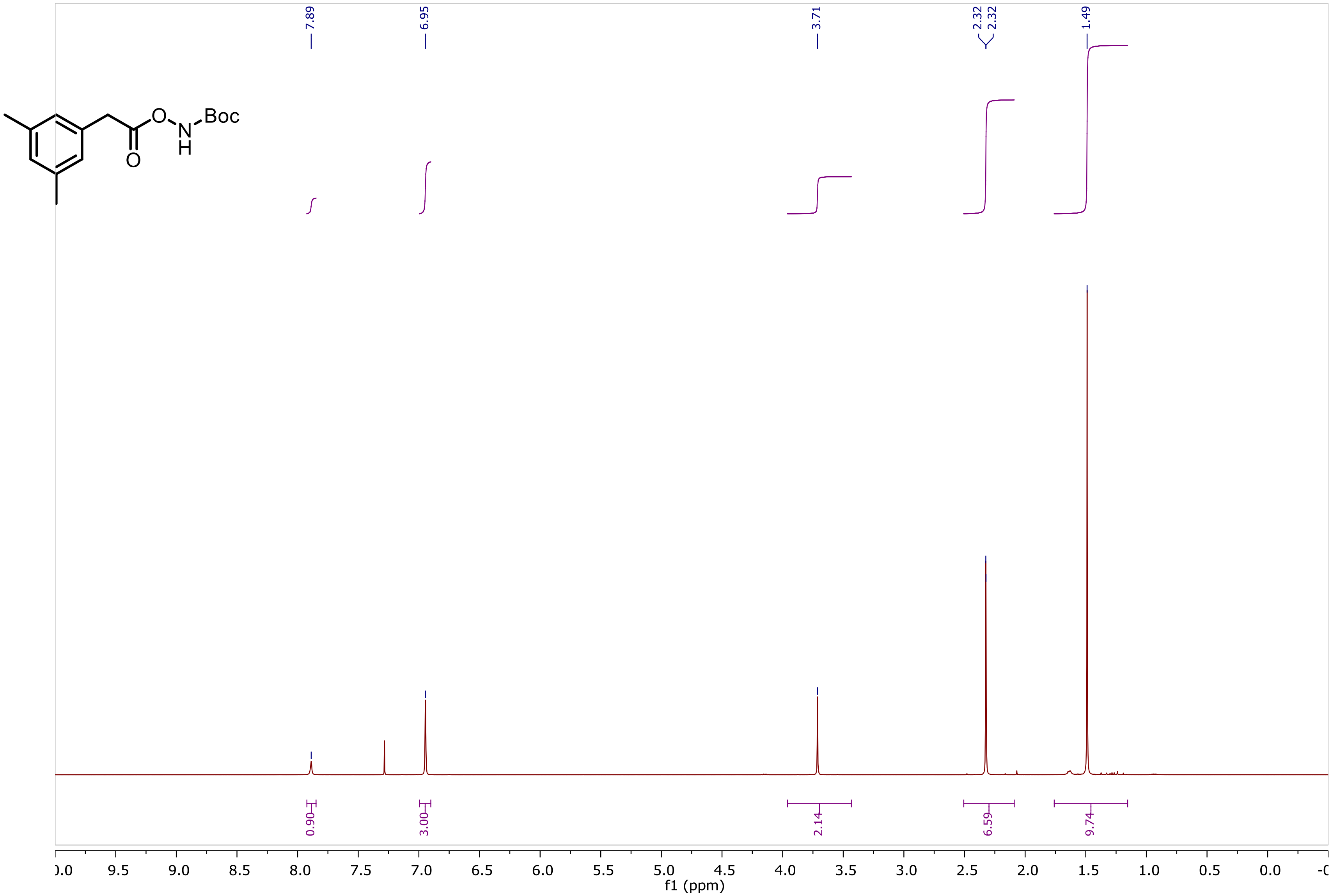

^{13}C NMR spectrum of *tert*-butyl (2-(3,5-dimethylphenyl)acetoxy)carbamate **5h** in $CDCl_3$

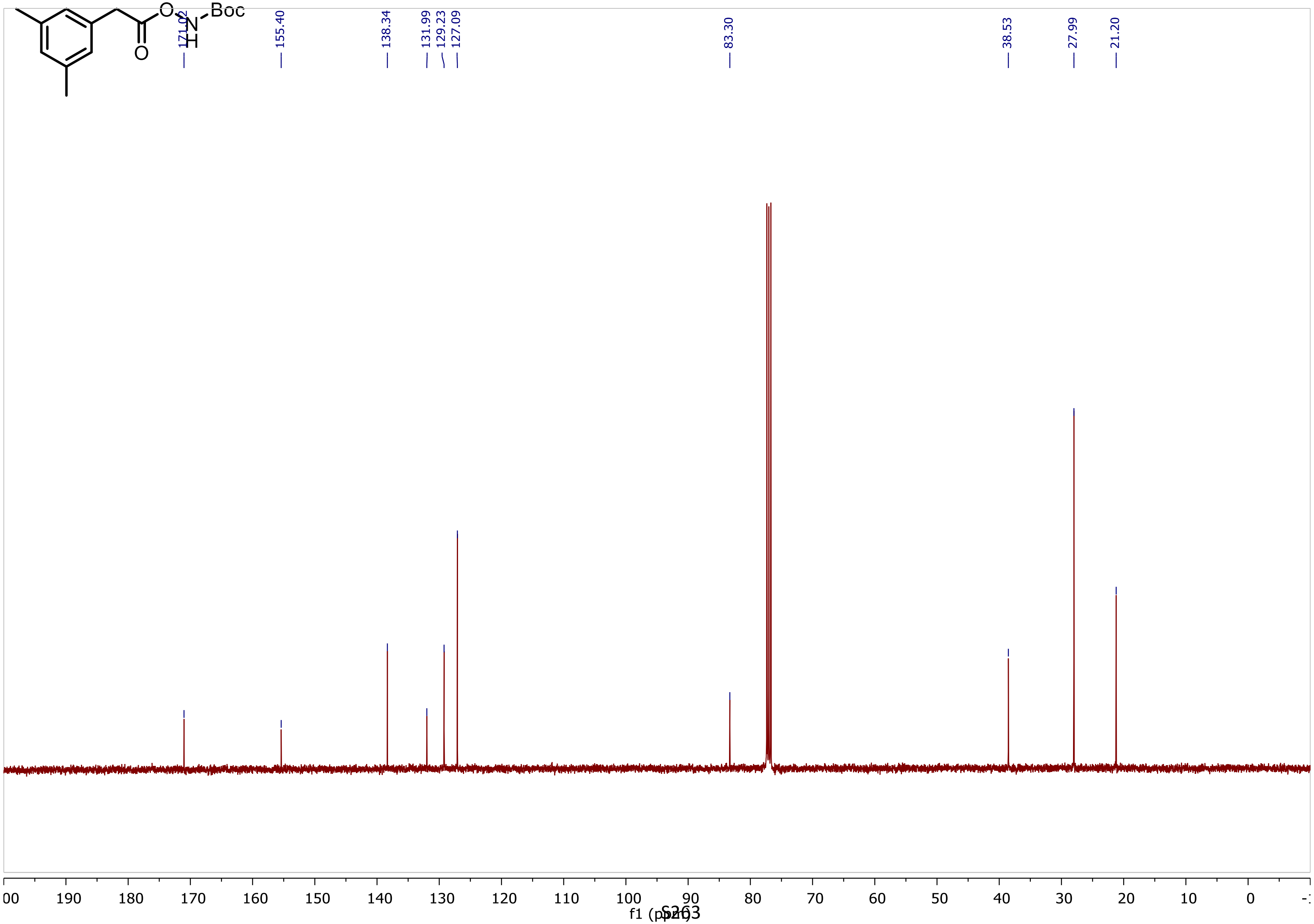

^{1}H NMR spectrum of *tert*-butyl (2-(3-bromophenyl)acetoxy)carbamate **5i** in $CDCl_3$

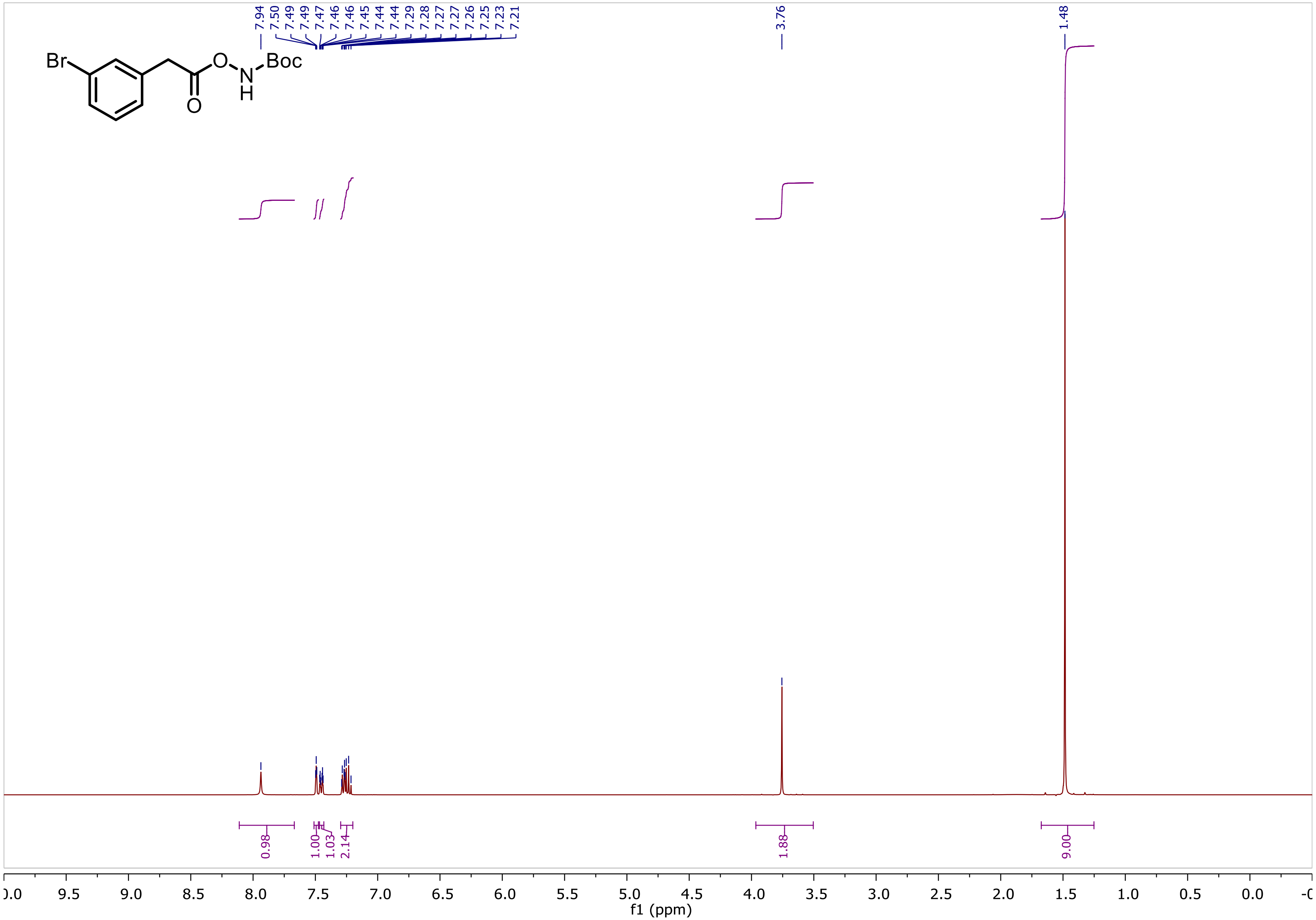

^{13}C NMR spectrum of *tert*-butyl (2-(3-bromophenyl)acetoxy)carbamate **5i** in $CDCl_3$

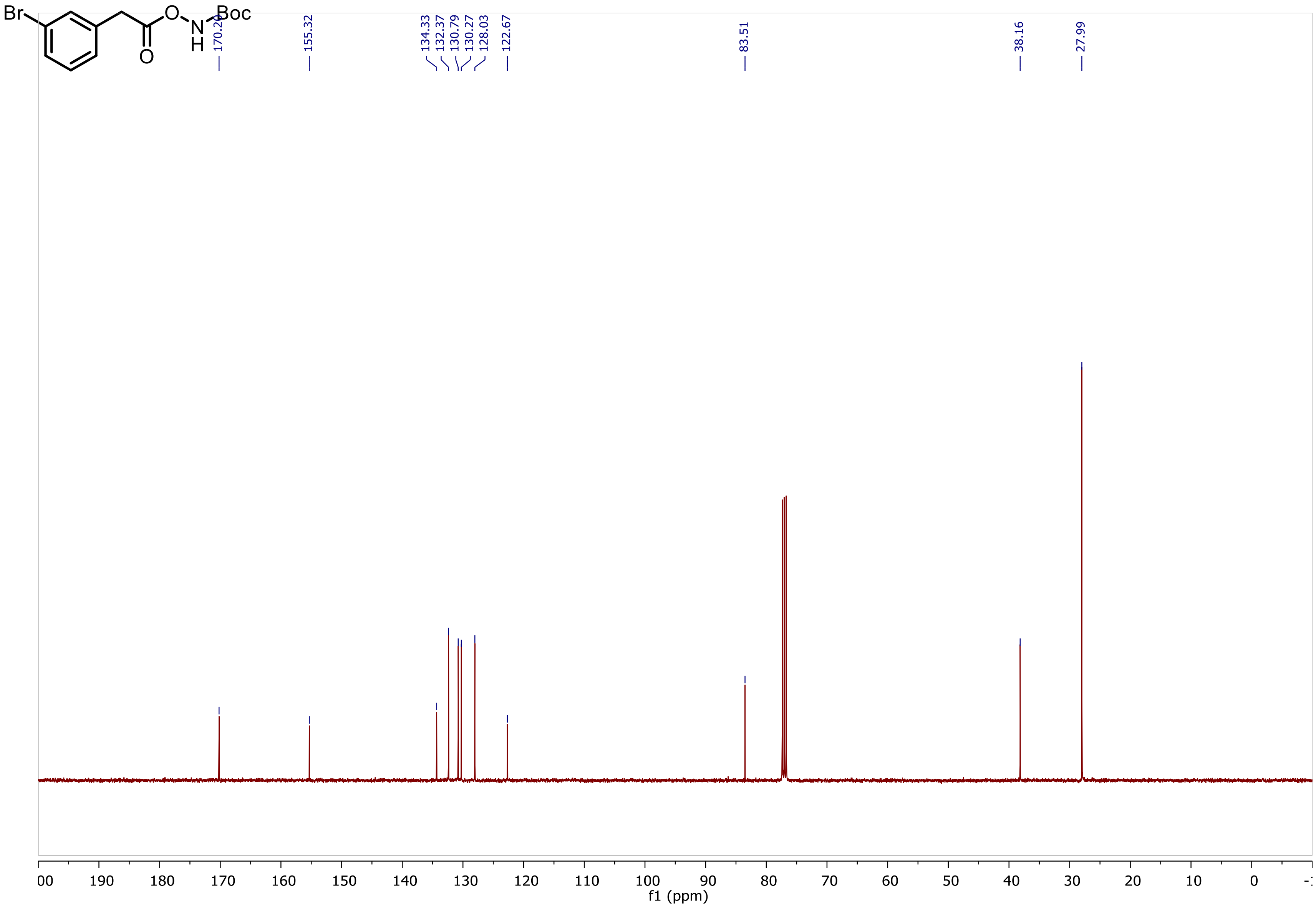

^{1}H NMR spectrum of *tert*-butyl ((3-(3-bromophenyl)propanoyl)oxy)carbamate **5j** in $CDCl_3$

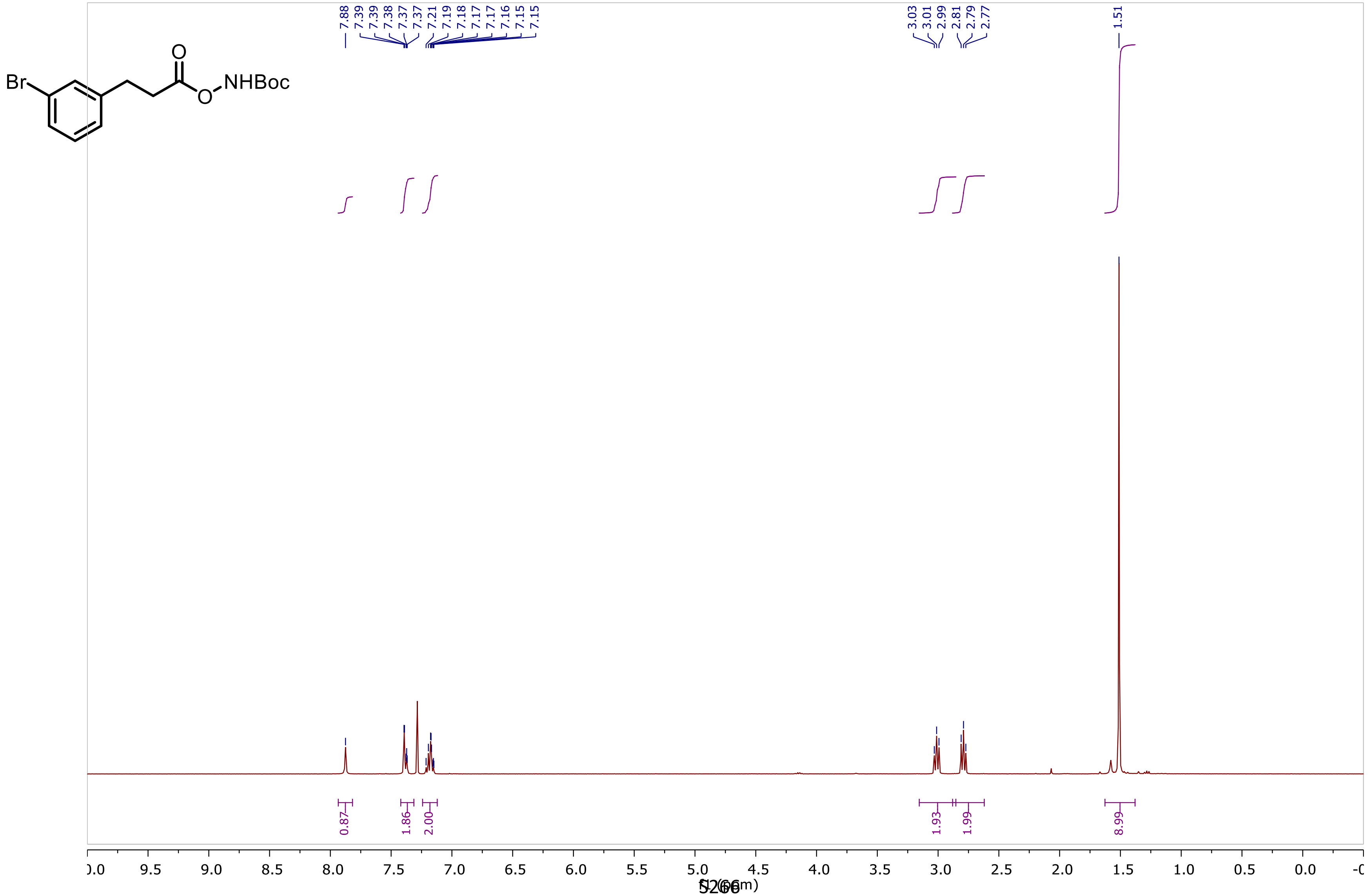

^{13}C NMR spectrum of *tert*-butyl ((3-(3-bromophenyl)propanoyl)oxy)carbamate **5j** in $CDCl_3$

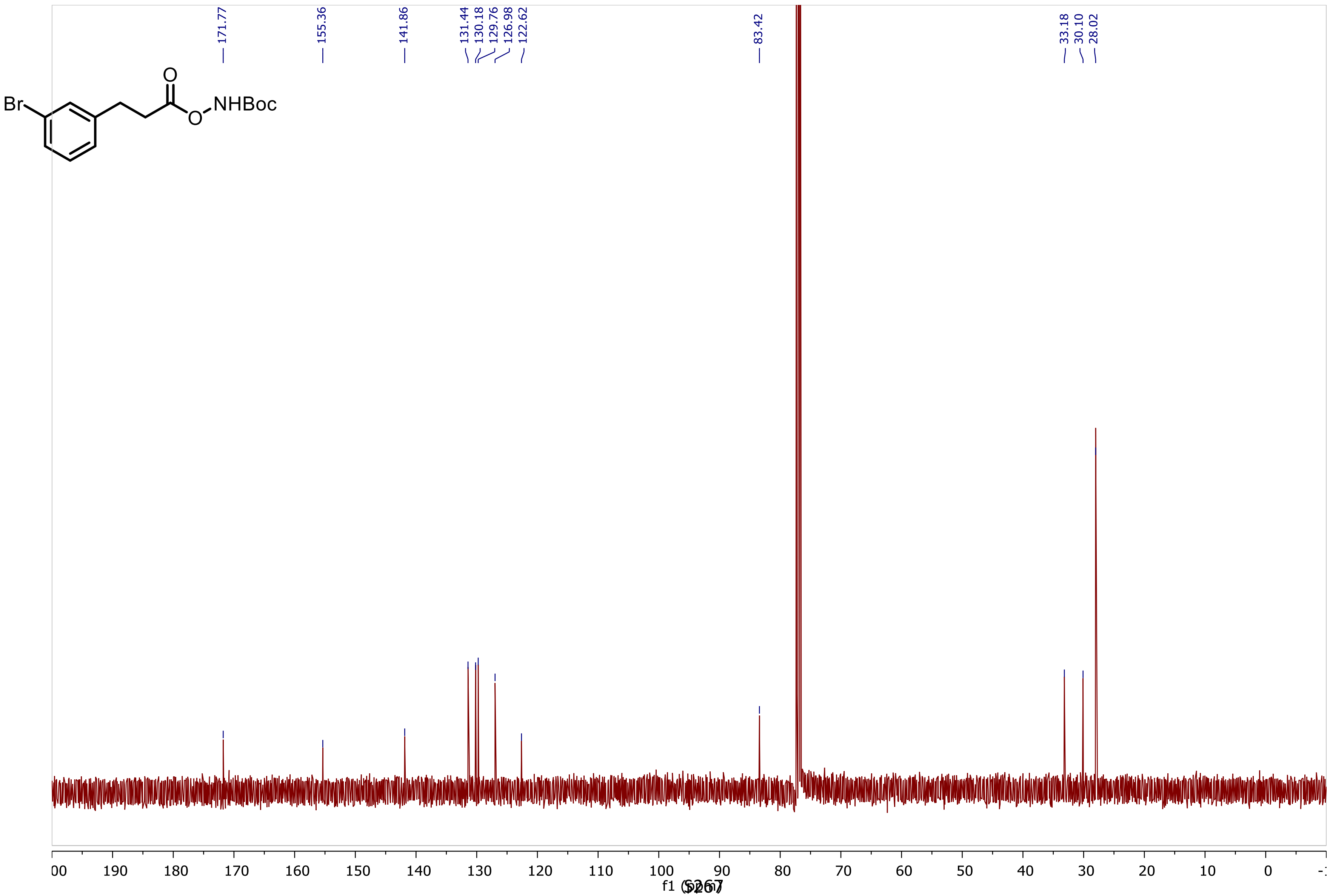

^{1}H NMR spectrum of *tert*-butyl ((3-(3,5-dimethoxyphenyl)propanoyl)oxy)carbamate **5k** in $CDCl_3$

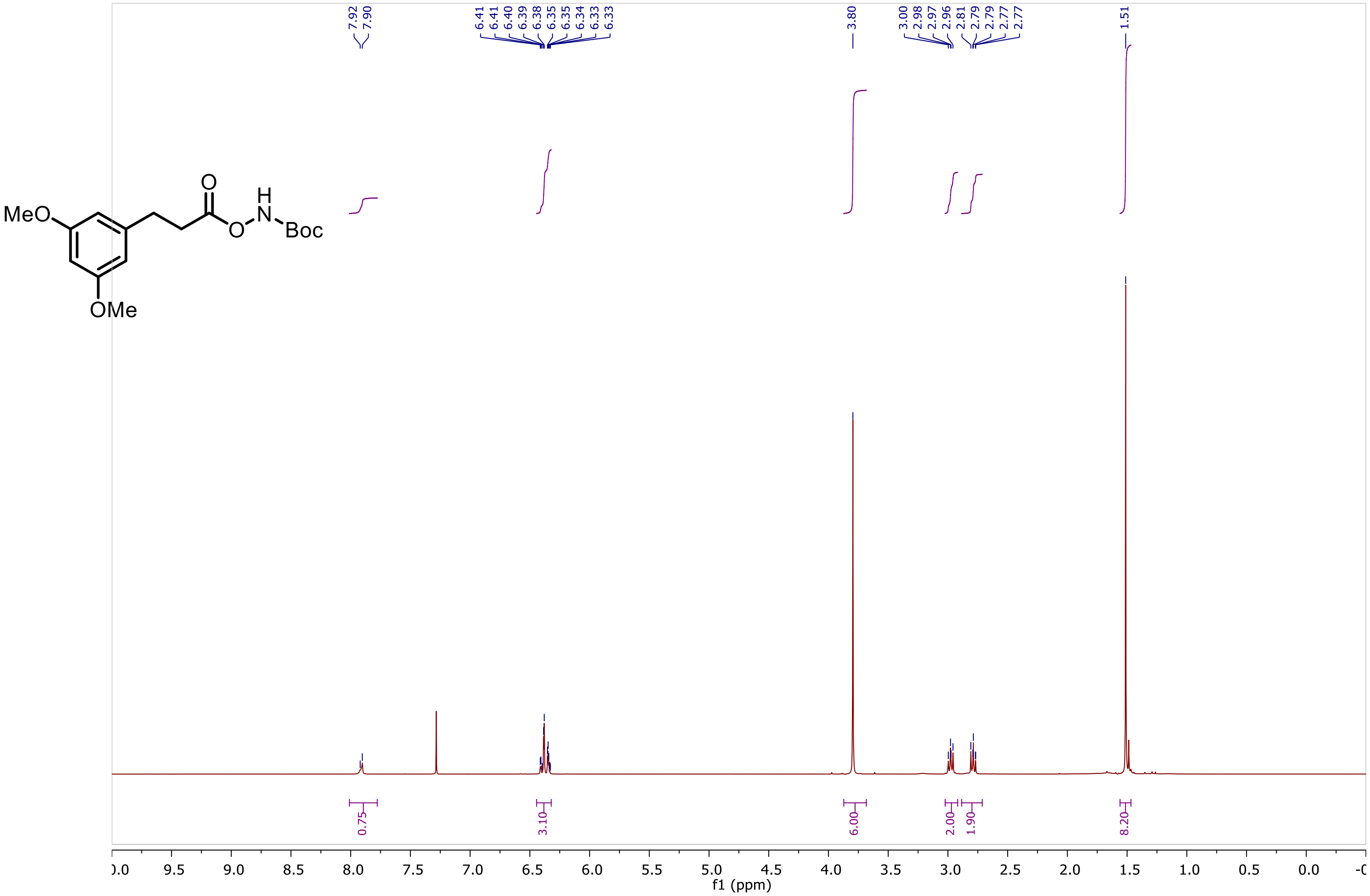

^{13}C NMR spectrum of *tert*-butyl ((3-(3,5-dimethoxyphenyl)propanoyl)oxy)carbamate **5k** in $CDCl_3$

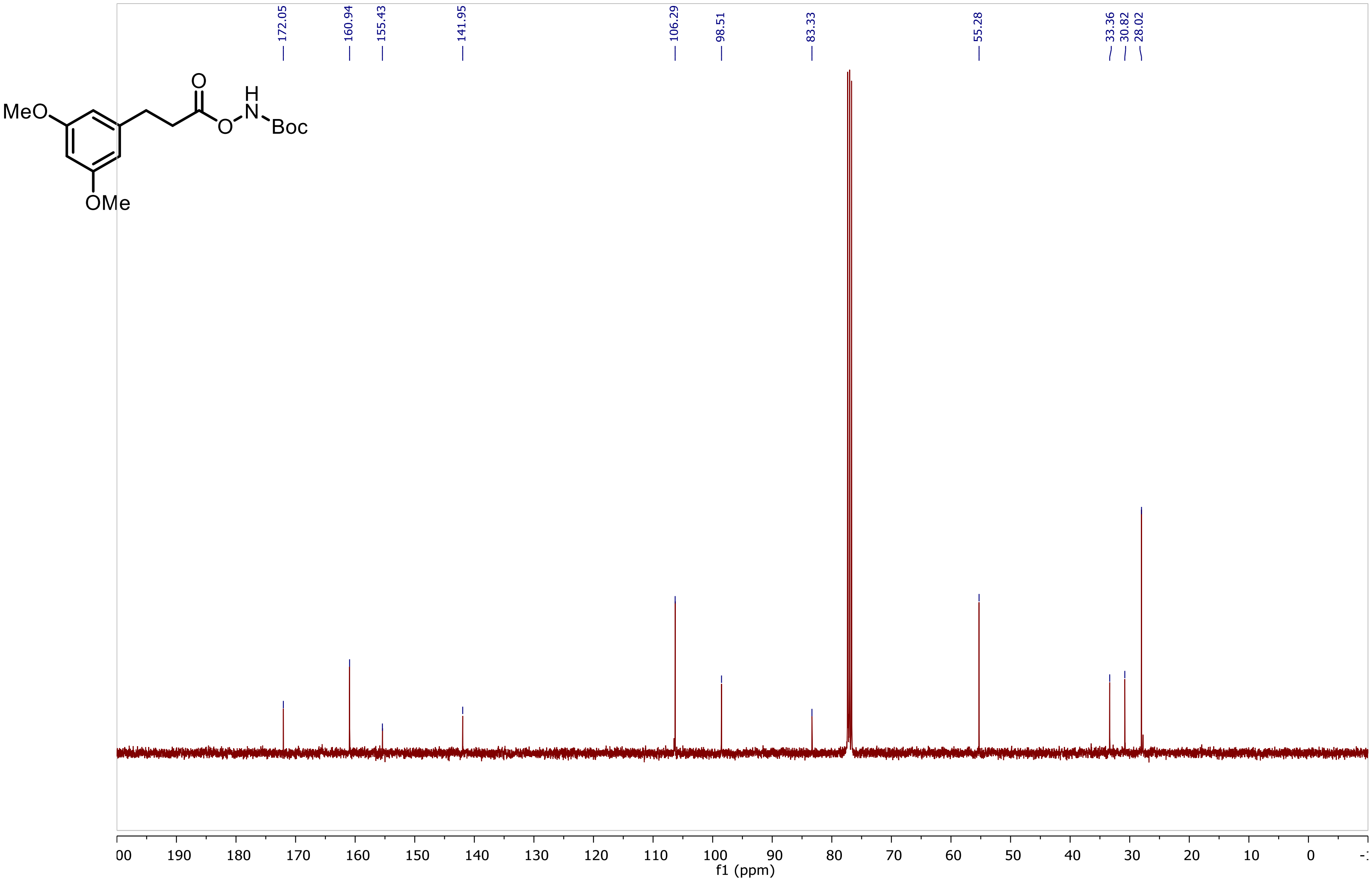

^{1}H NMR spectrum of *tert*-butyl ((4-methylphenyl)propanoyl)oxy)carbamate **5l** in $CDCl_3$

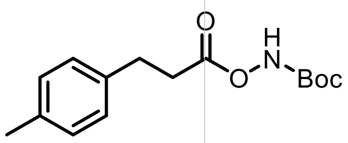

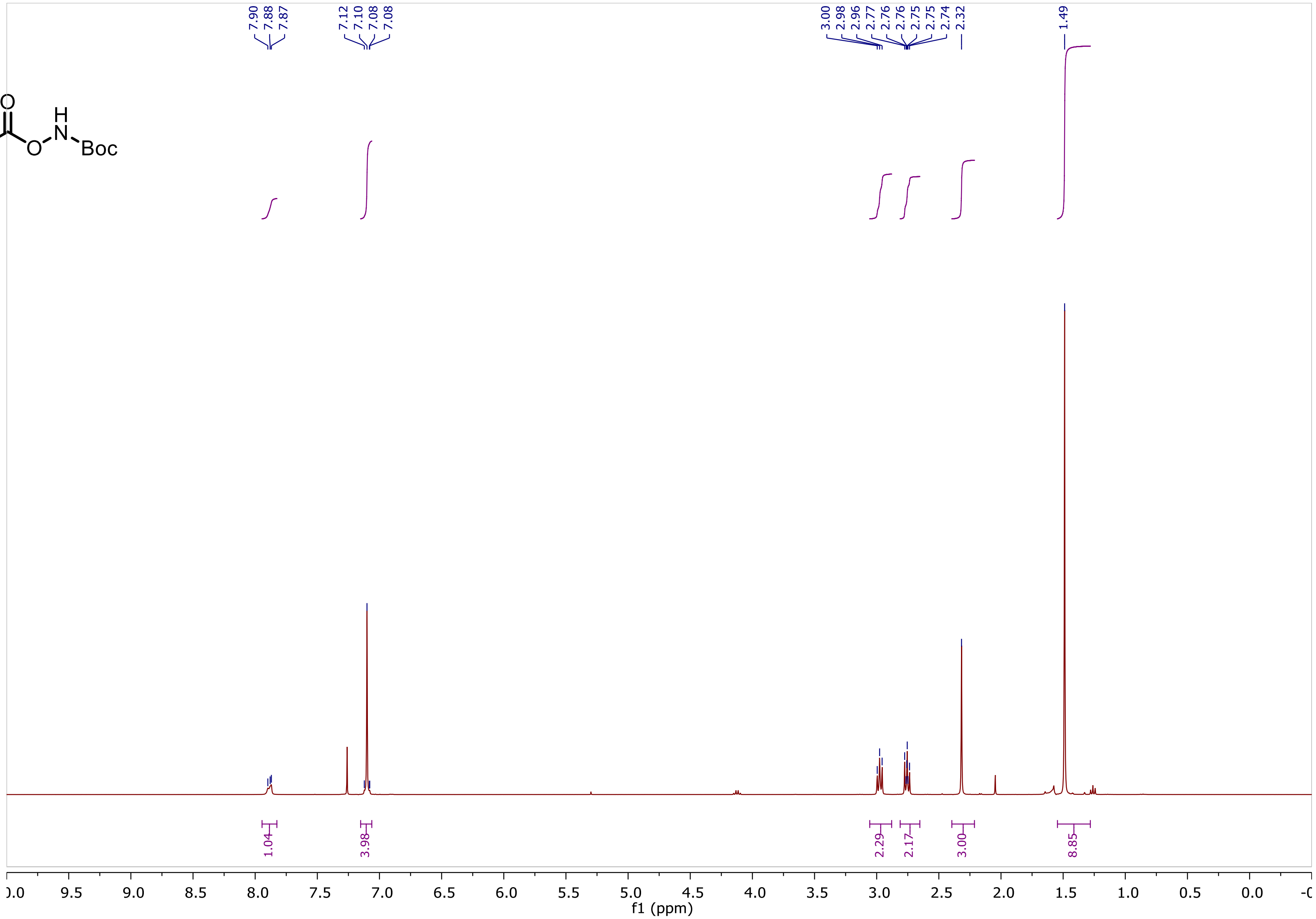

^{13}C NMR spectrum of *tert*-butyl ((4-methylphenyl)propanoyl)oxy)carbamate **5l** in $CDCl_3$

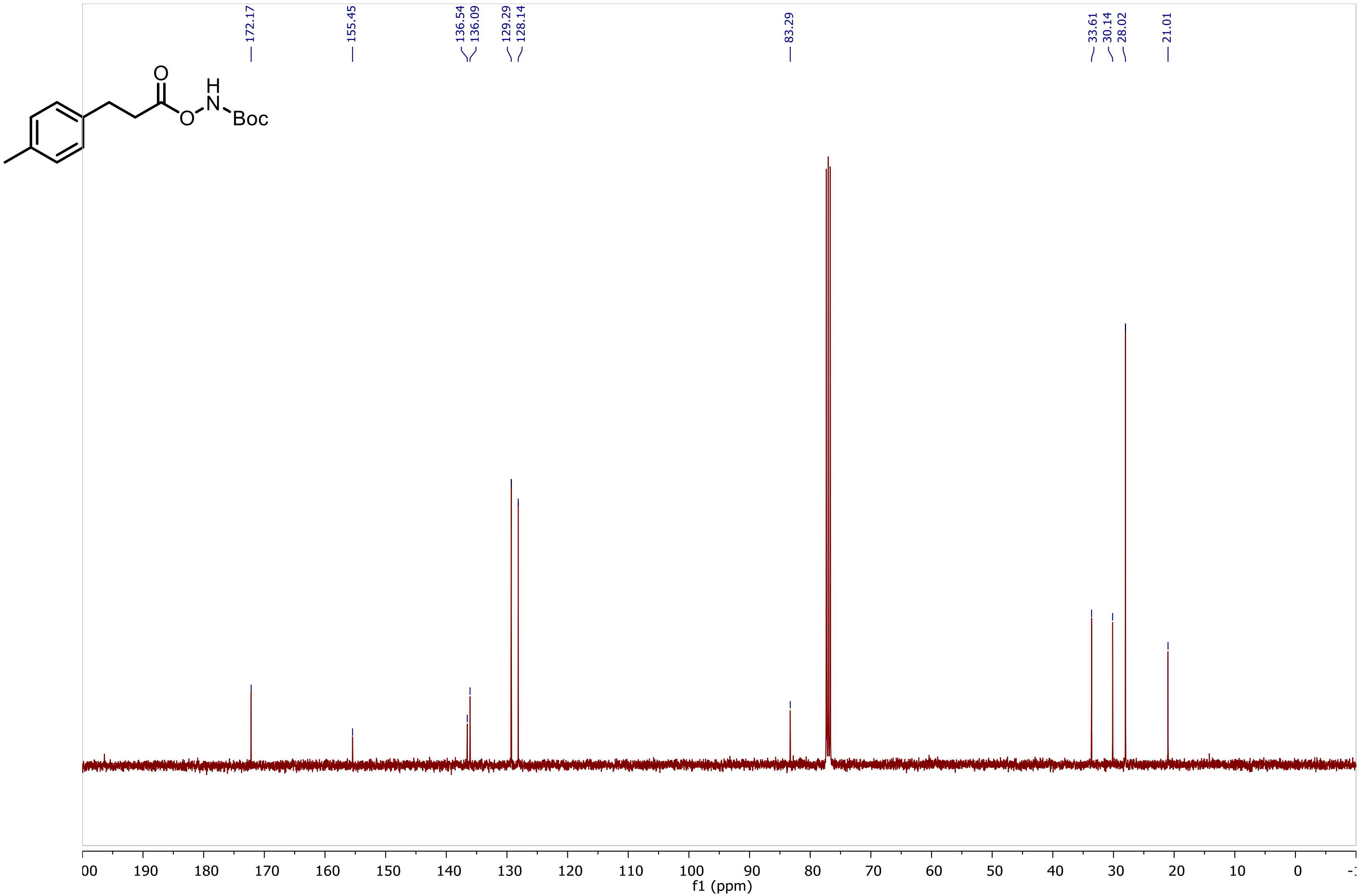

Amination Products

^{1}H NMR spectrum of 2-aminobenzoic acid hydrochloride **2a** in MeOD-d_4

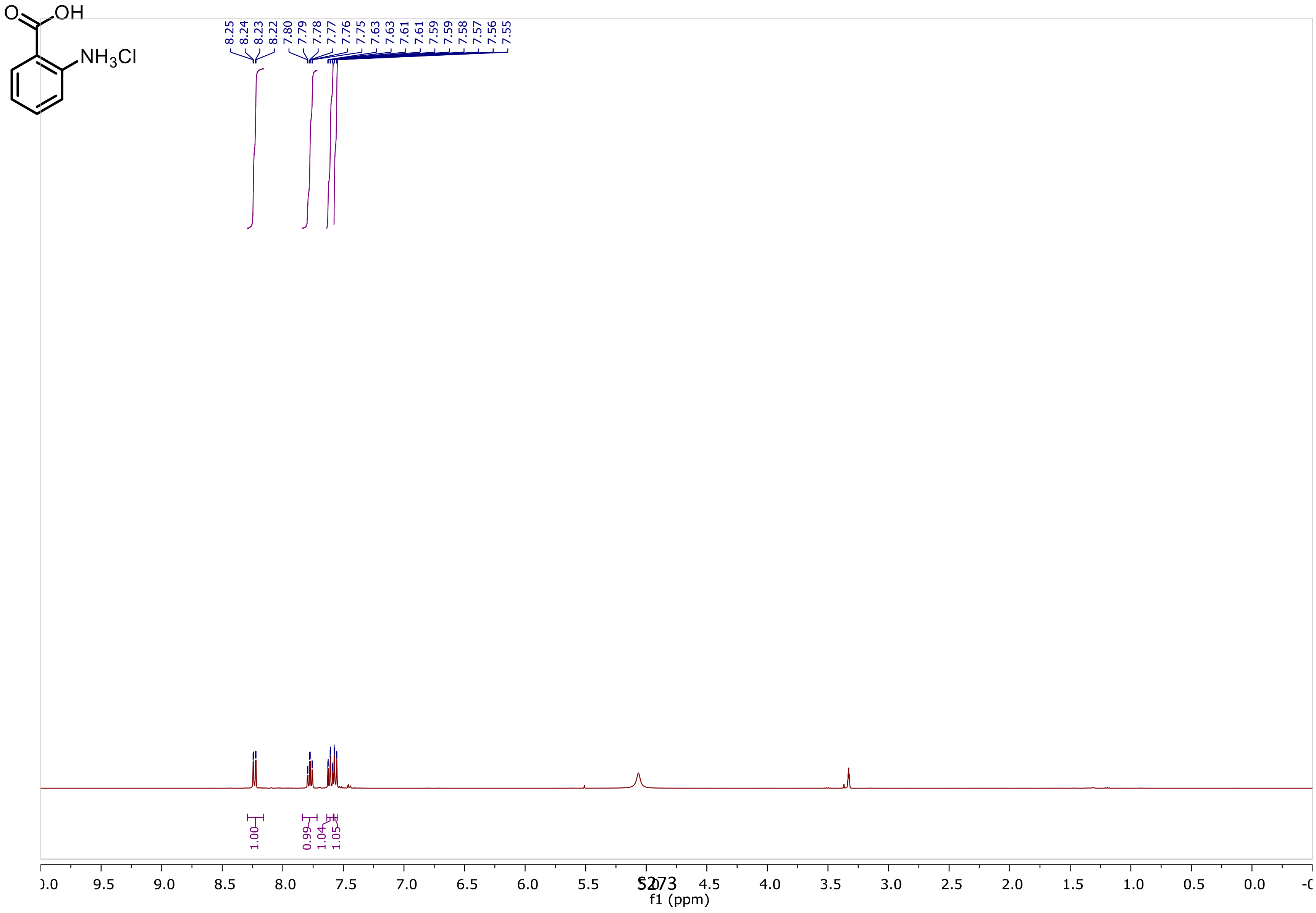

^{13}C NMR spectrum of 2-aminobenzoic acid hydrochloride **2a** in MeOD-d_4

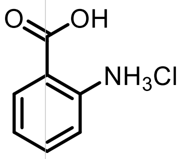

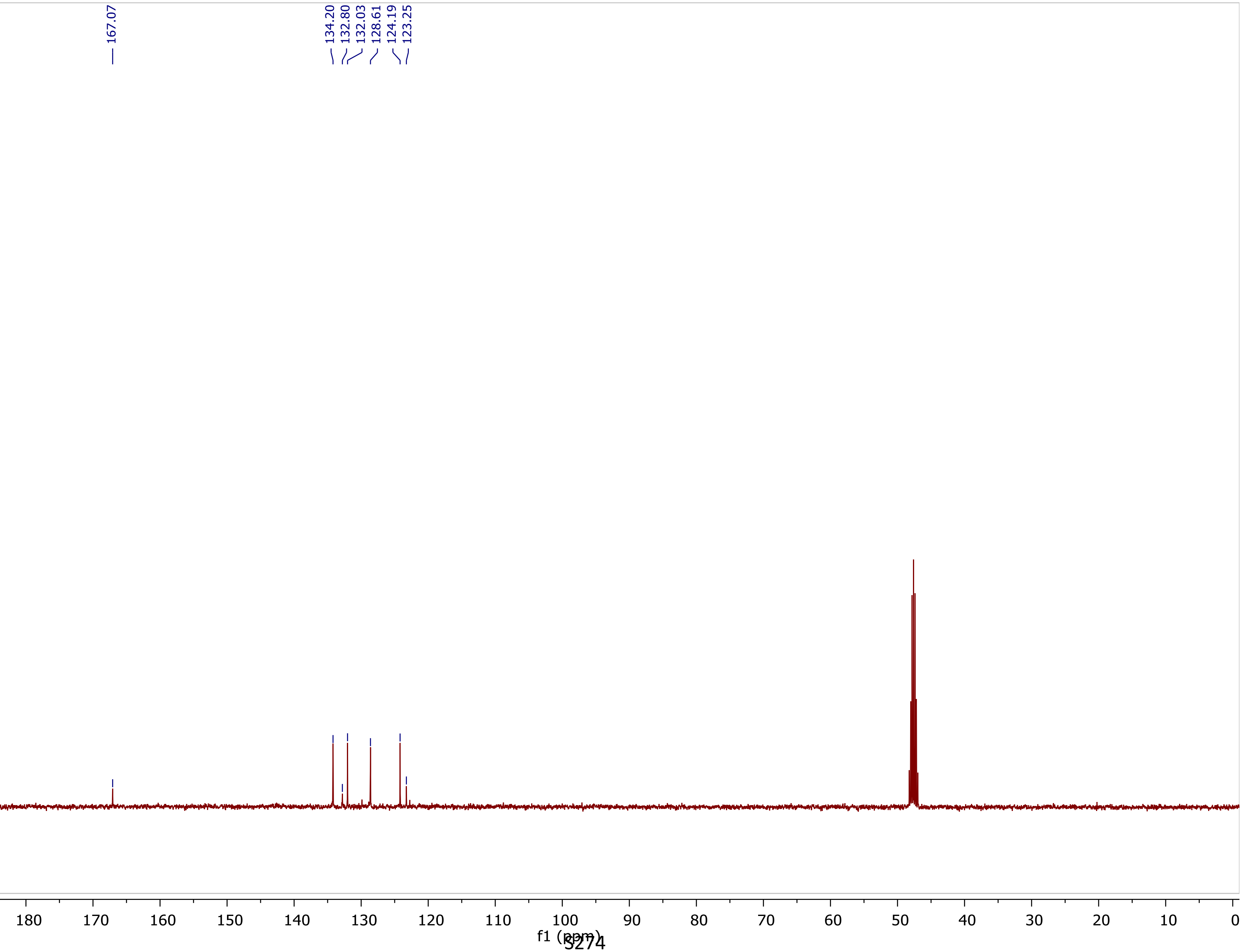

^{1}H NMR spectrum of 2-amino-4-methylbenzoic acid hydrochloride **2b** in MeOD-d_4

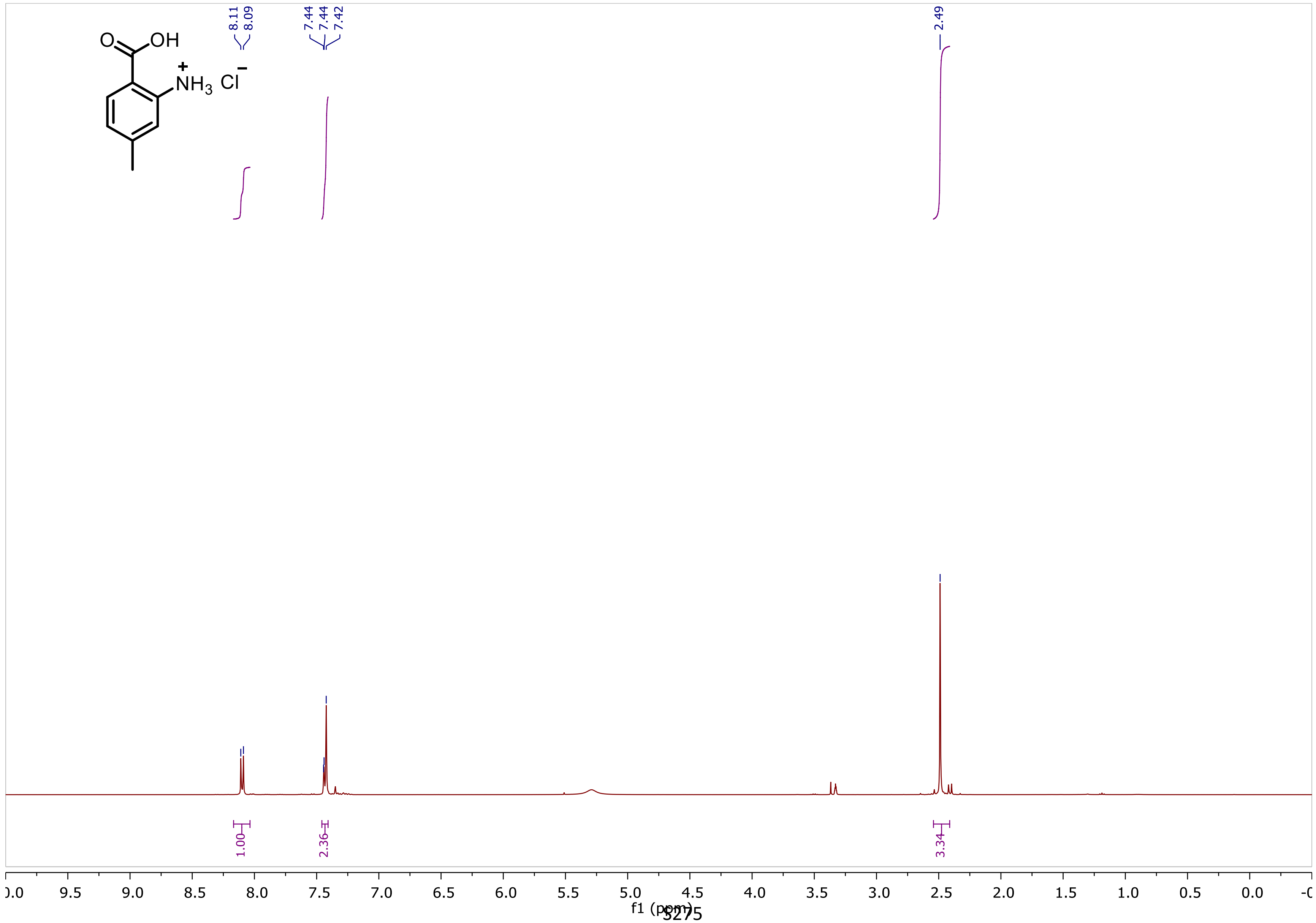

^{13}C NMR spectrum of 2-amino-4-methylbenzoic acid hydrochloride **2b** in MeOD-d_4

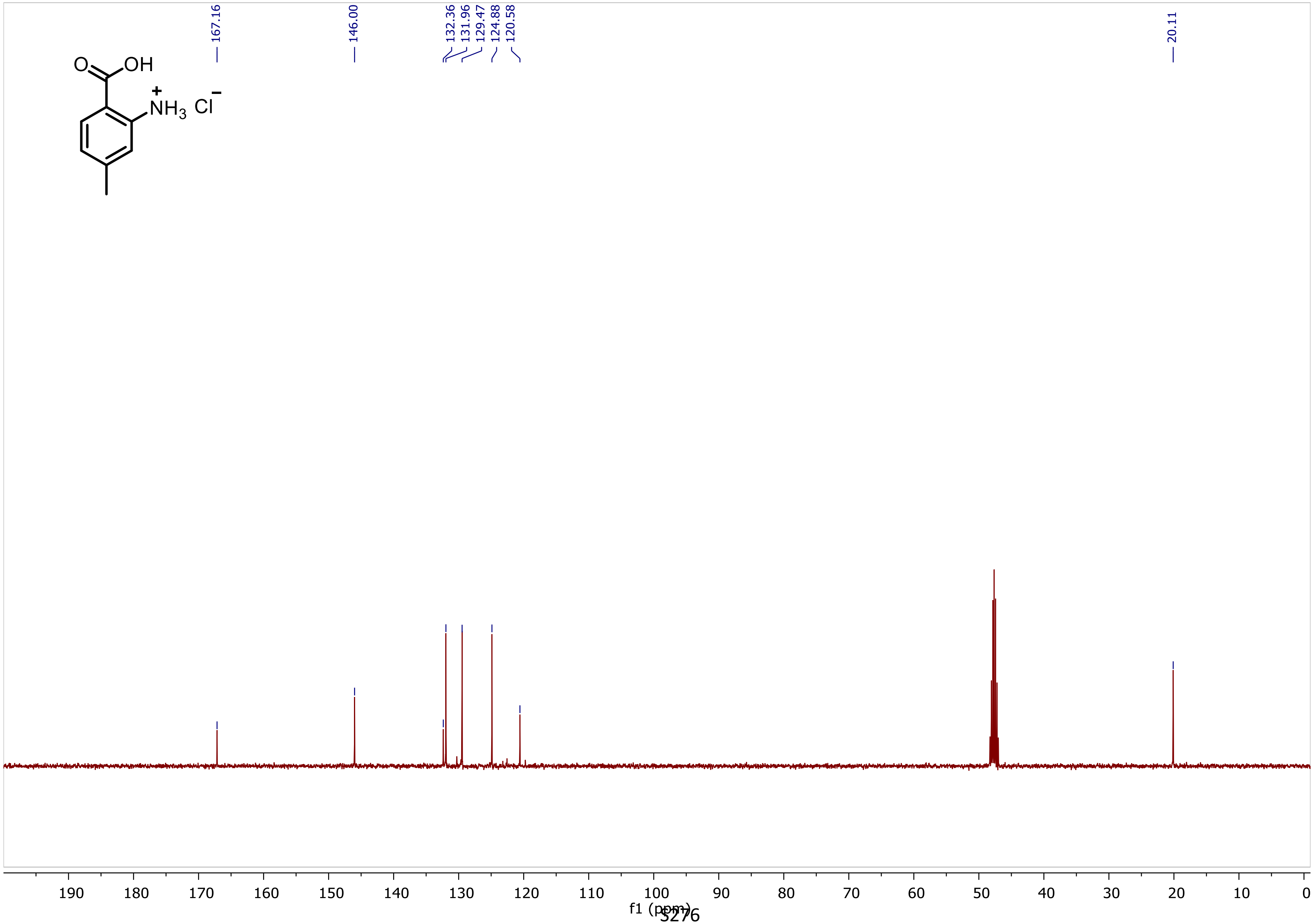

^{1}H NMR spectrum of 2-amino-4-propylbenzoic acid hydrochloride **2c** in MeOD-d_4

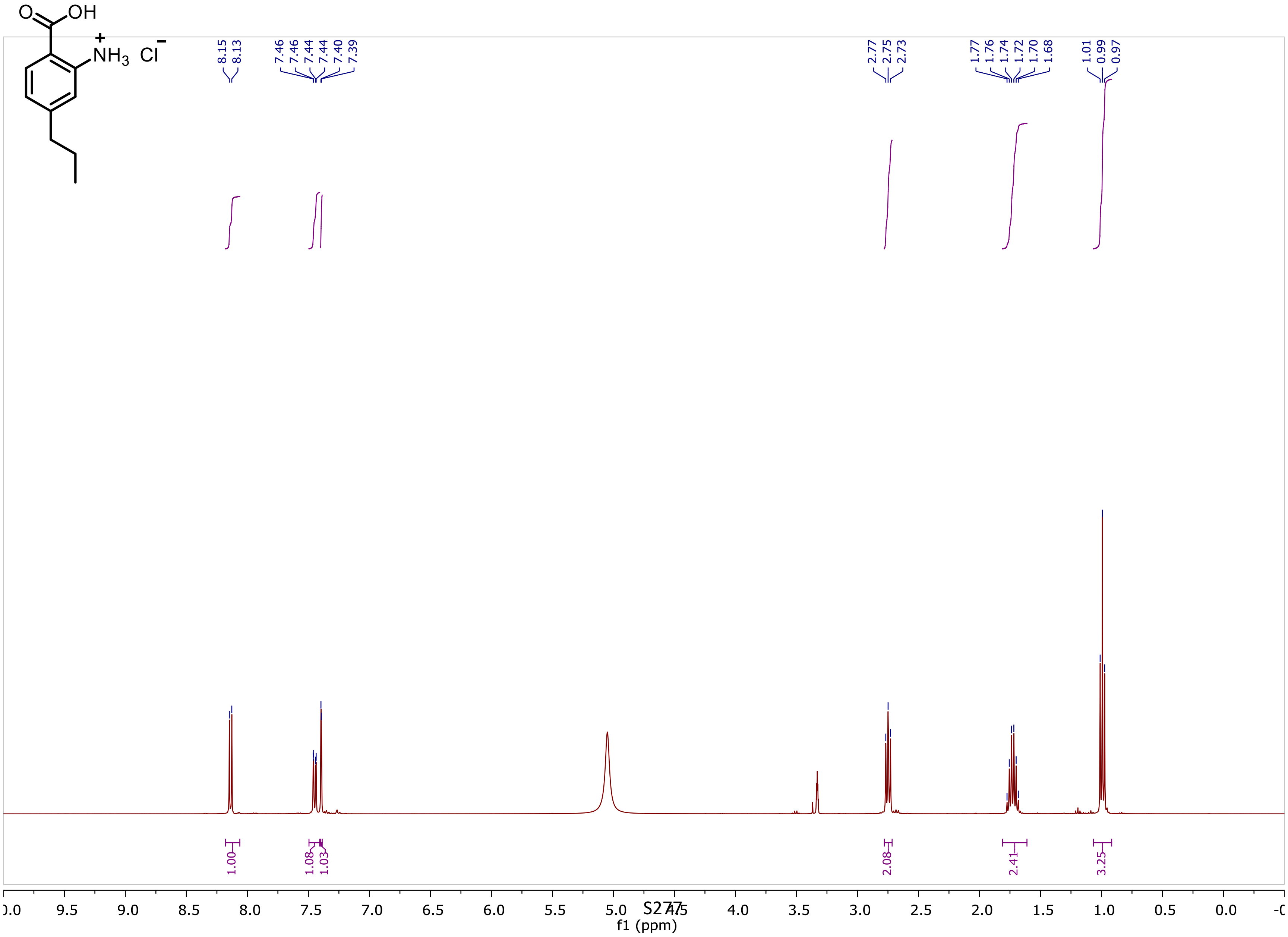

^{13}C NMR spectrum of 2-amino-4-propylbenzoic acid hydrochloride **2c** in MeOD-d_4

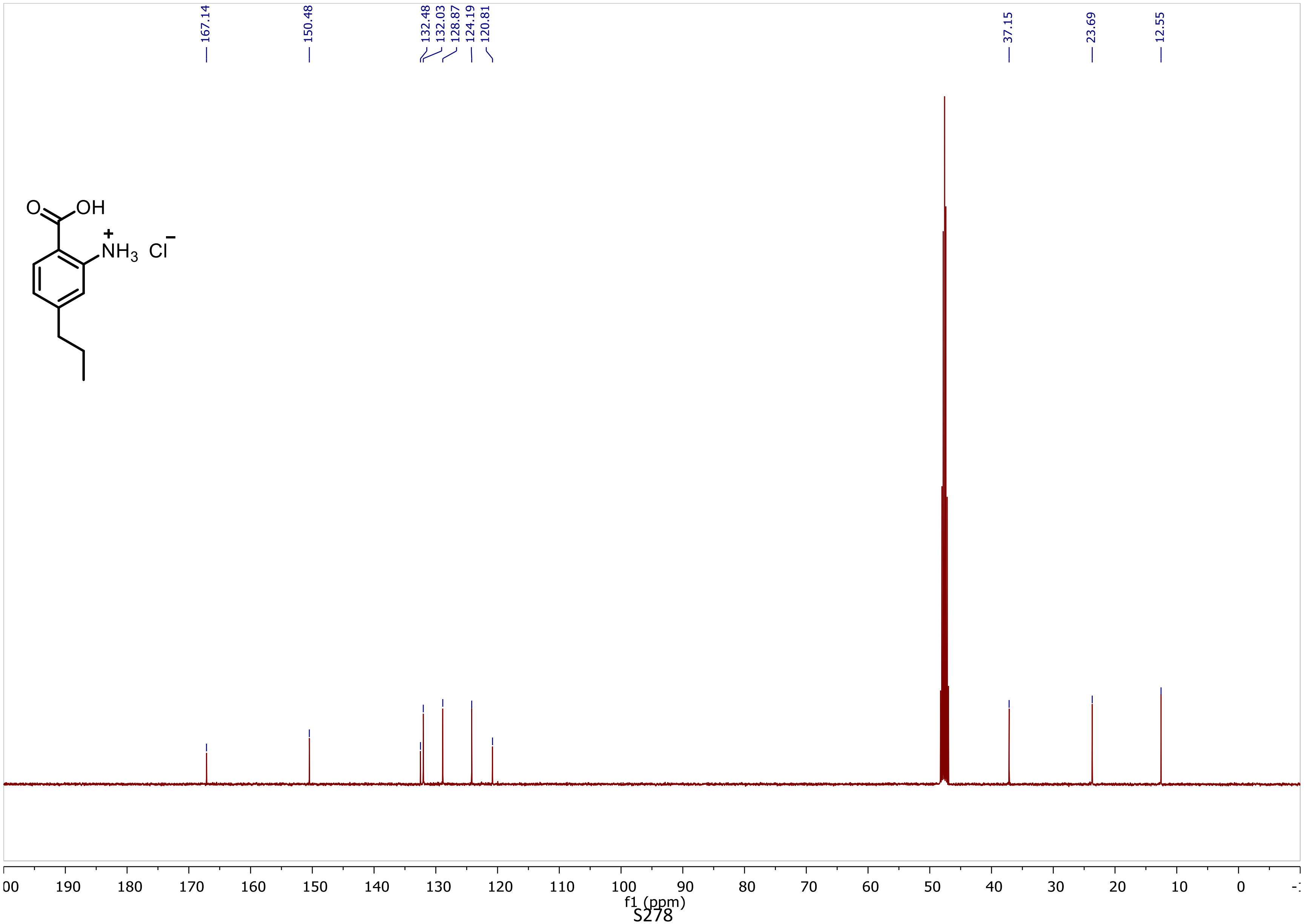

^{1}H NMR spectrum of 2-amino-4-isopropylbenzoic acid hydrochloride **2d** in MeOD-d_4

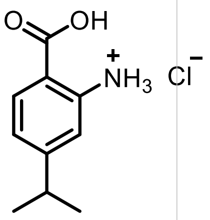

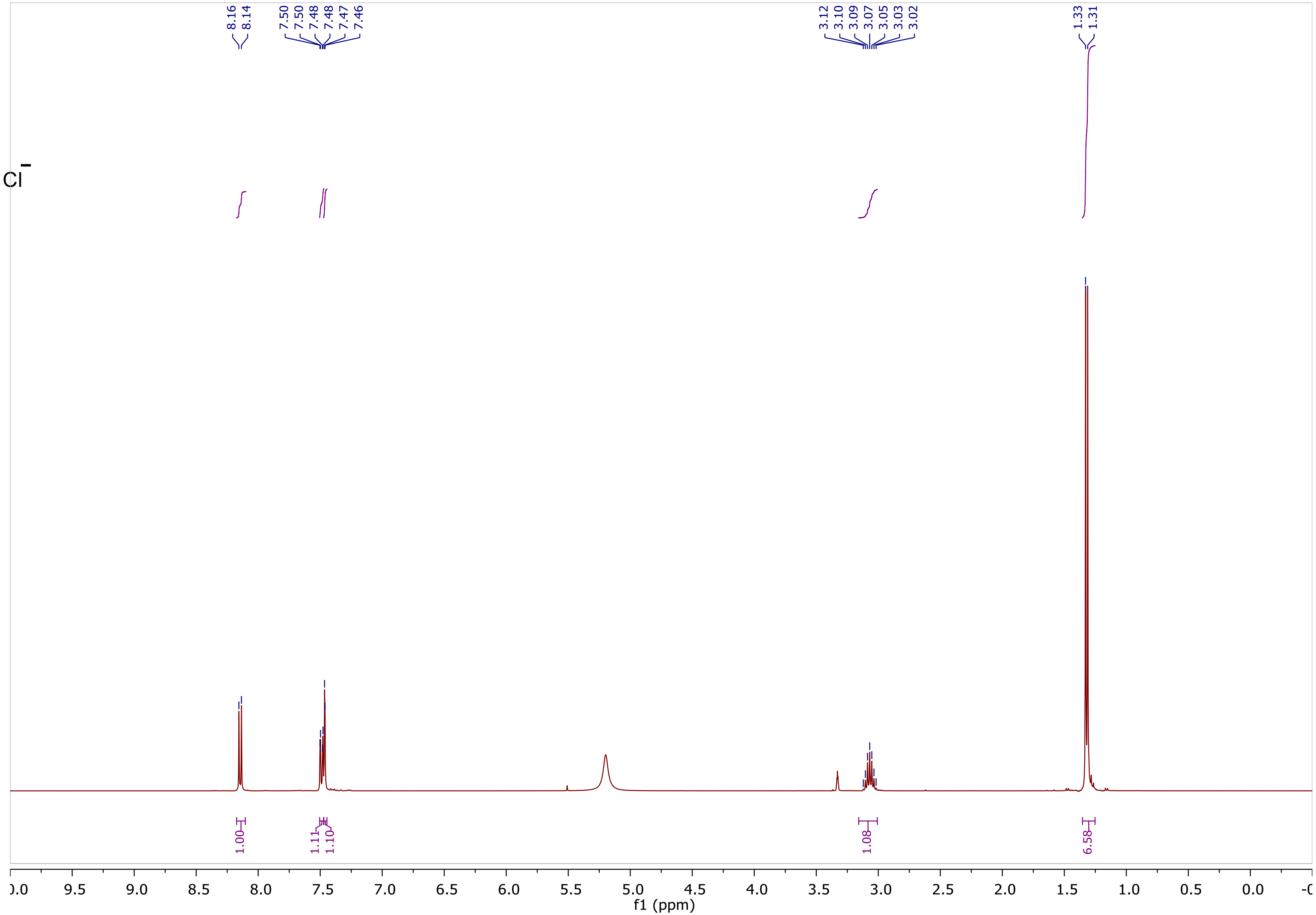

^{13}C NMR spectrum of 2-amino-4-isopropylbenzoic acid hydrochloride **2d** in MeOD-d_4

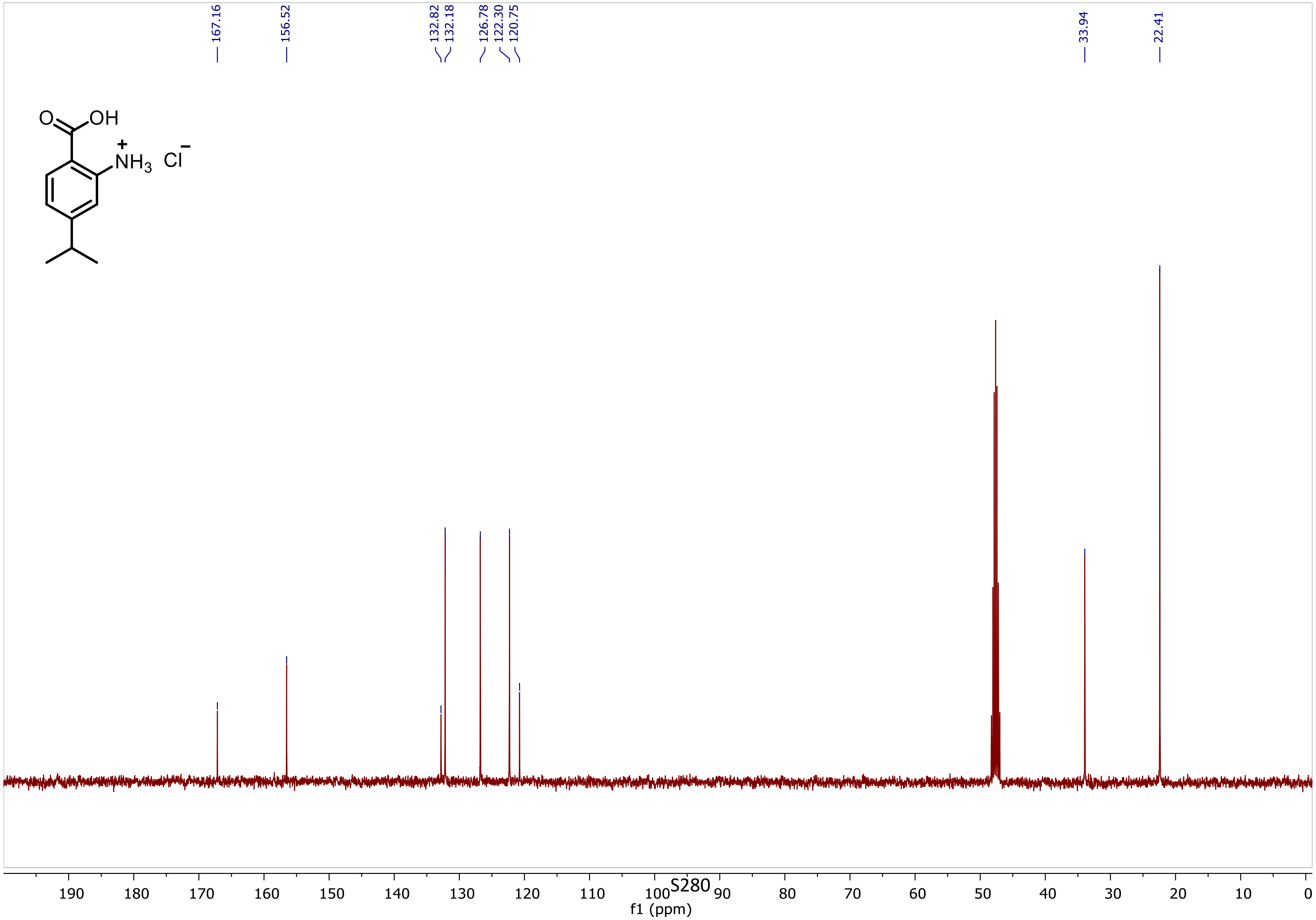

^{1}H NMR spectrum of 2-amino-5-methylbenzoic acid hydrochloride **2e (a)** and 2-amino-3-methylbenzoic acid hydrochloride ***iso*-2e (b)** in MeOD-d_4

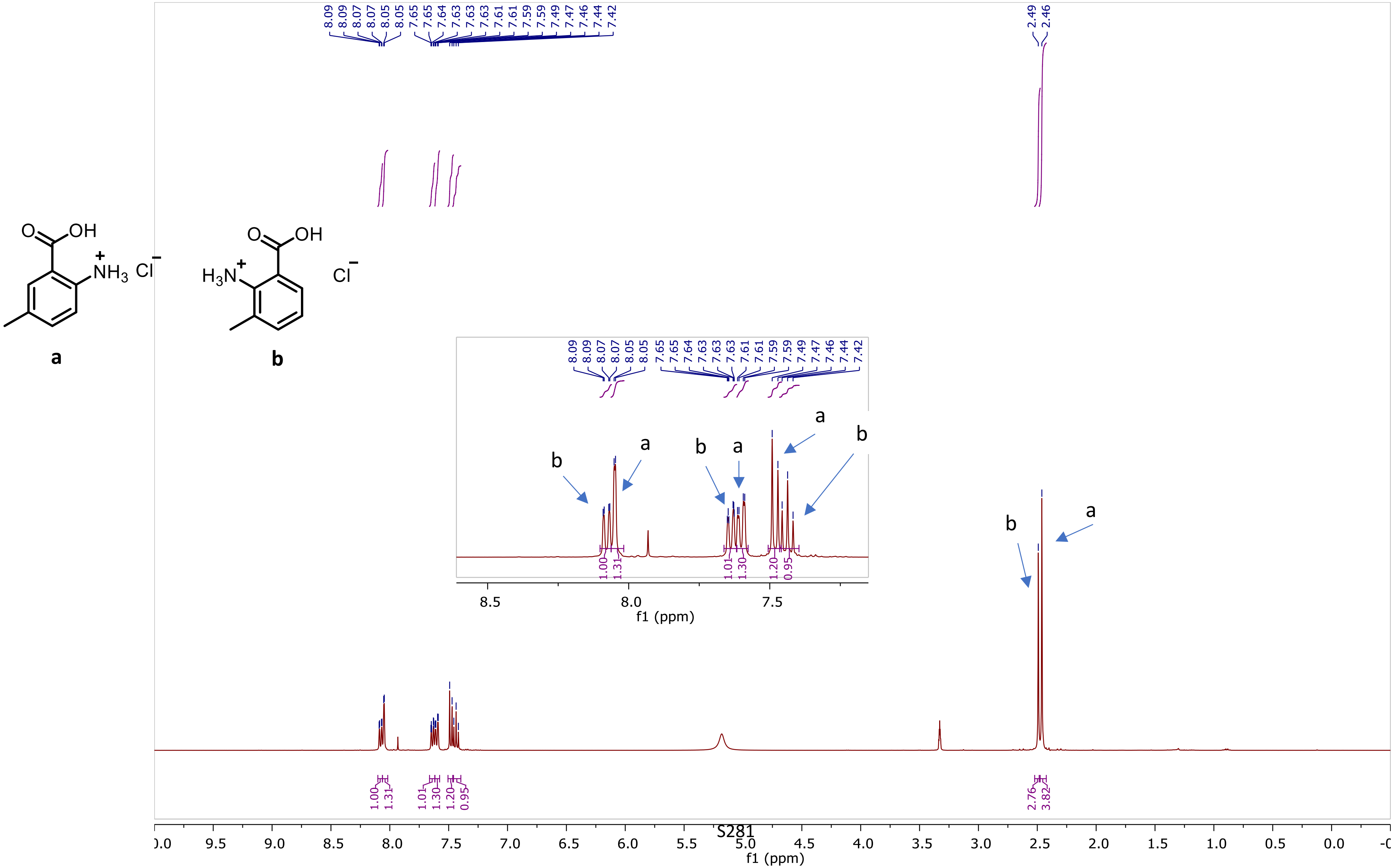

^{13}C NMR spectrum of 2-amino-5-methylbenzoic acid hydrochloride **2e** and 2-amino-3-methylbenzoic acid hydrochloride ***iso*-2e** in MeOD-d_4

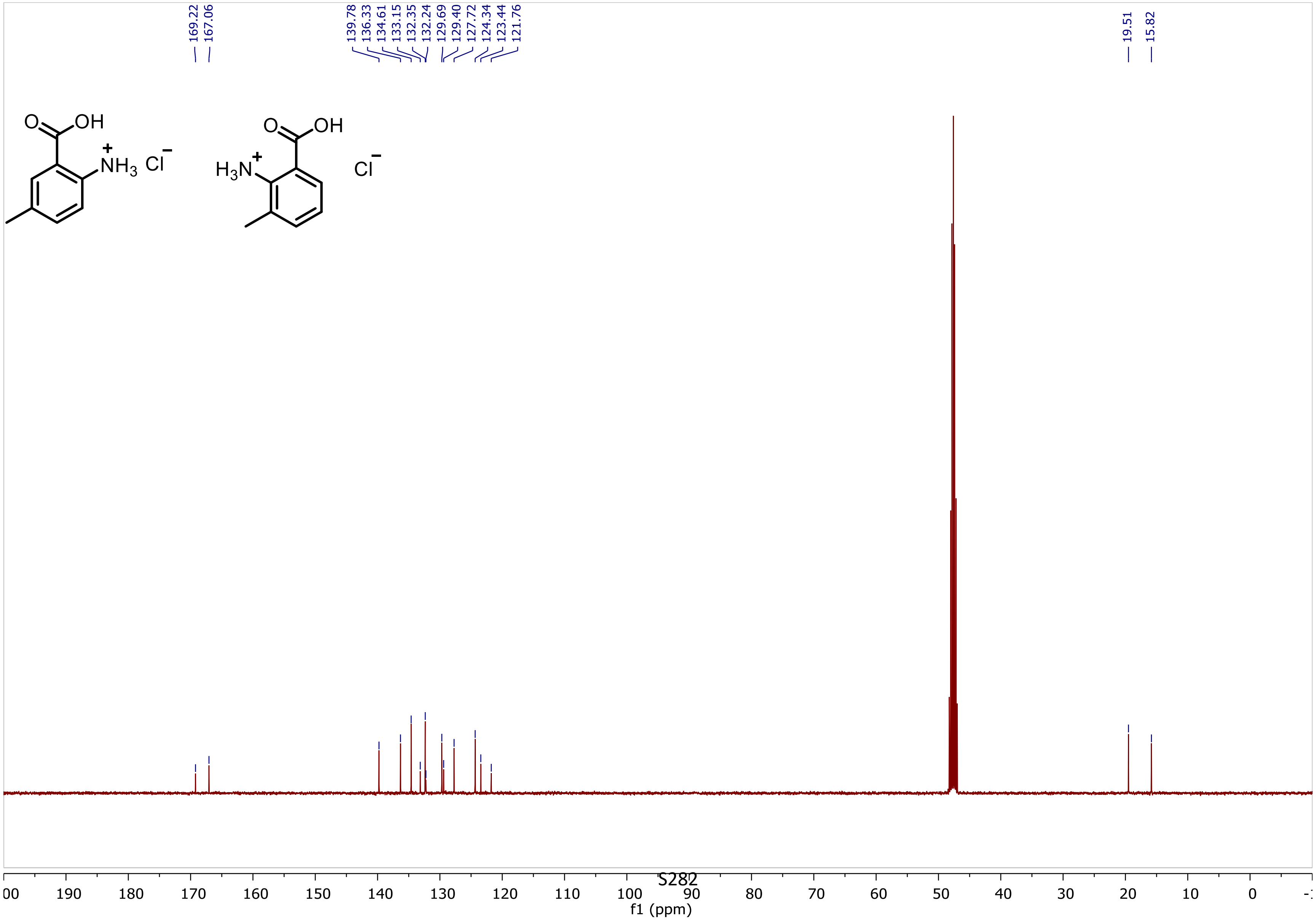

^{1}H NMR spectrum of 2-amino-3,5-dimethylbenzoic acid hydrochloride **2f** in MeOD-d_4

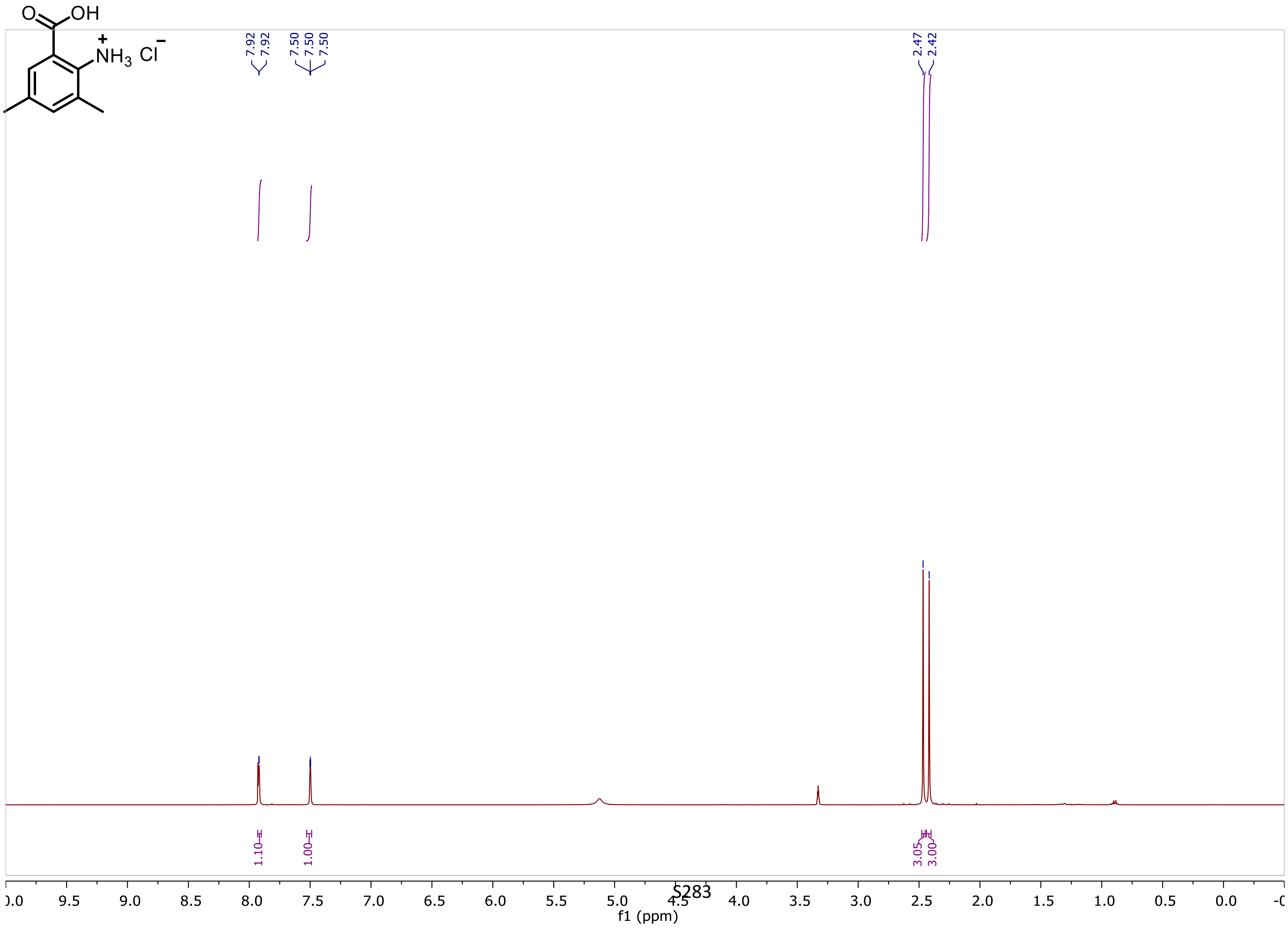

^{13}C NMR spectrum of 2-amino-3,5-dimethylbenzoic acid hydrochloride **2f** in MeOD-d_4

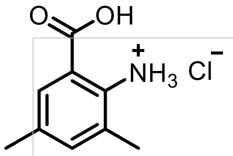

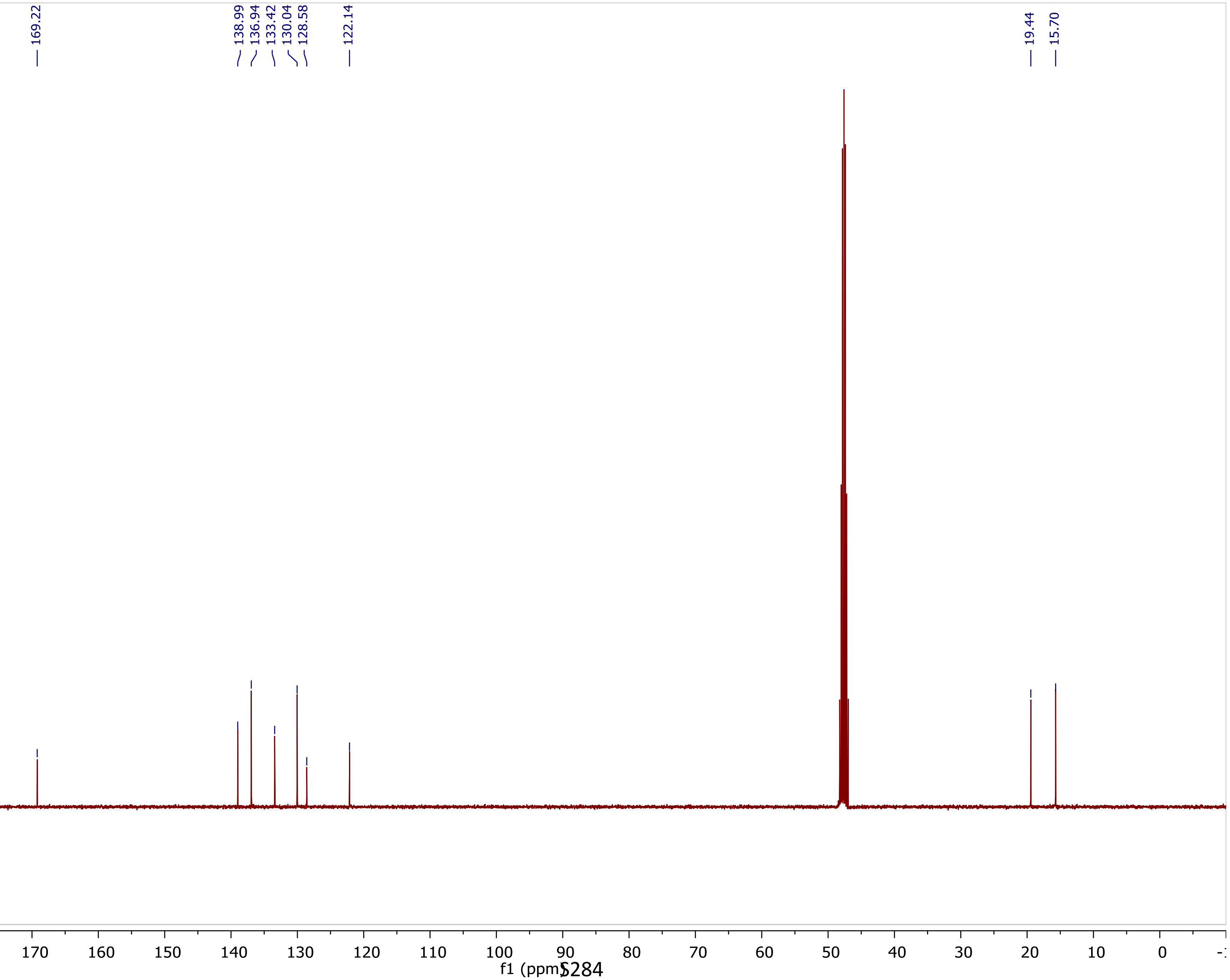

^{1}H NMR spectrum of 2-amino-5-isopropylbenzoic acid hydrochloride **2g (a)** and 2-amino-3-isopropylbenzoic acid hydrochloride ***iso*-2g (b)** in MeOD-d_4

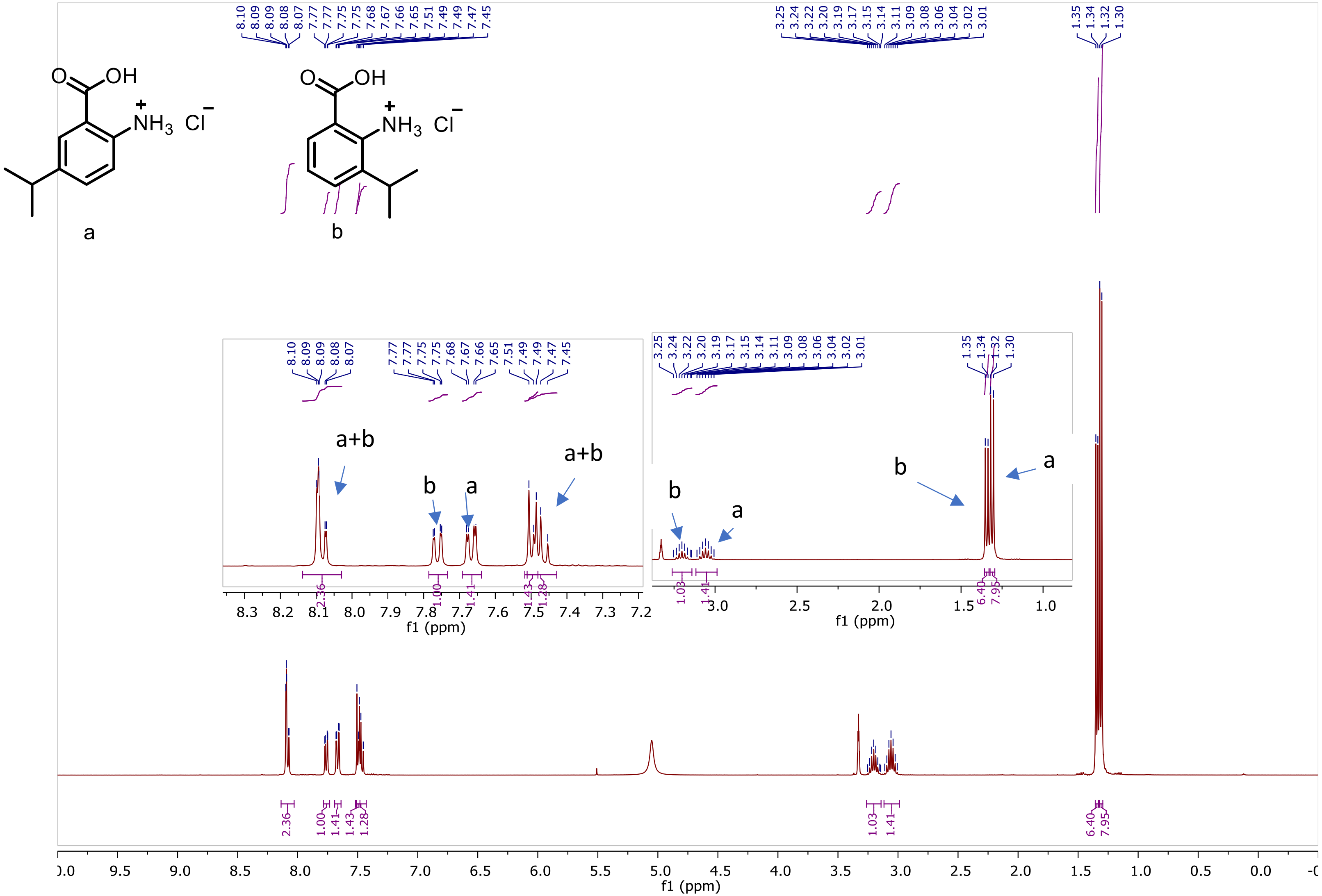

^{13}C NMR spectrum of 2-amino-5-isopropylbenzoic acid hydrochloride **2g** and 2-amino-3-isopropylbenzoic acid hydrochloride ***iso*-2g** in MeOD-d_4

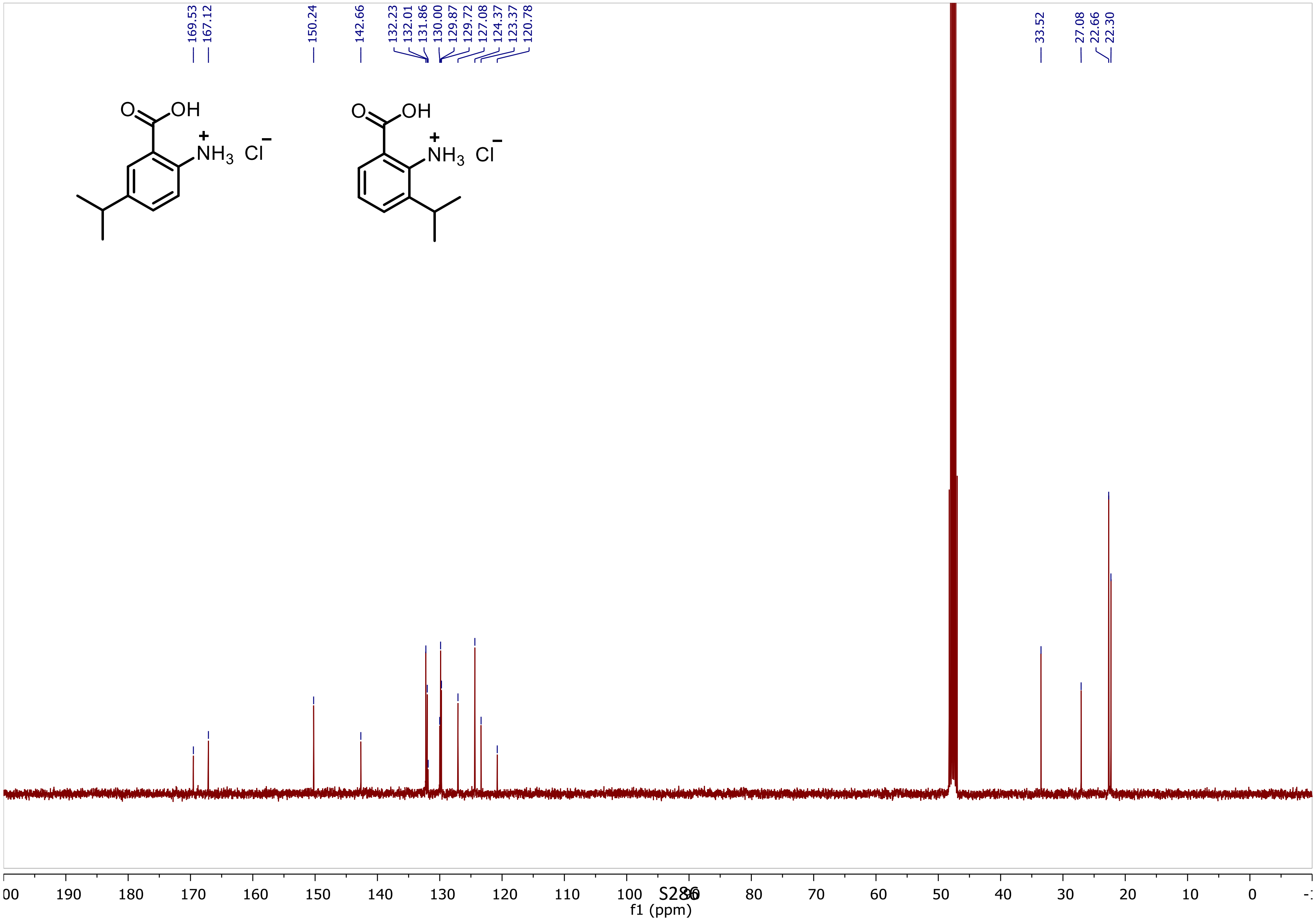

^{1}H NMR spectrum of 2-amino-4,5-dimethylbenzoic acid hydrochloride **2h (a)** and 2-amino-3,4-dimethylbenzoic acid hydrochloride ***iso*-2h (b)** in MeOD-d_4

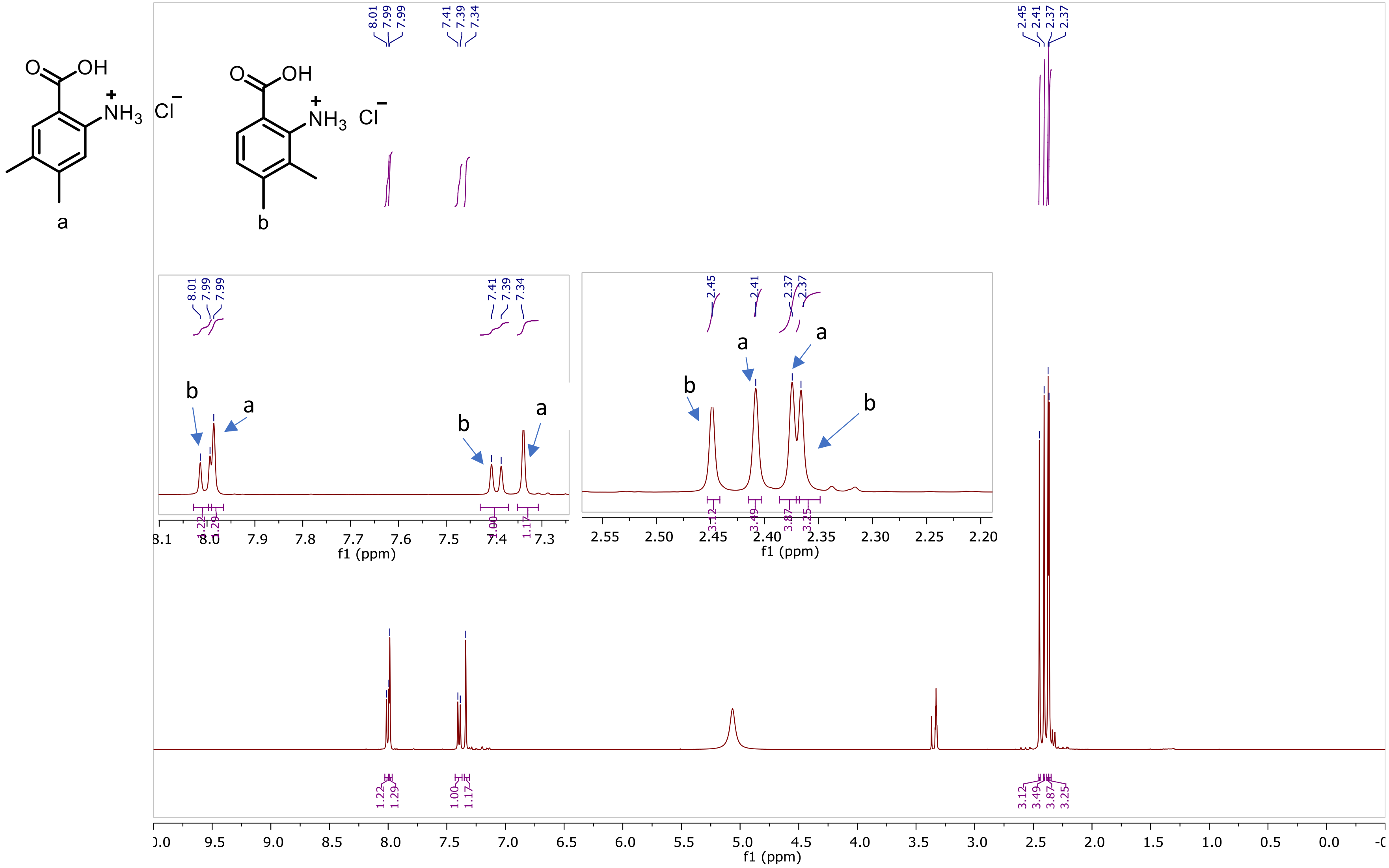

^{13}C NMR spectrum of 2-amino-4,5-dimethylbenzoic acid hydrochloride **2h** and 2-amino-3,4-dimethylbenzoic acid hydrochloride ***iso*-2h** in MeOD-d_4

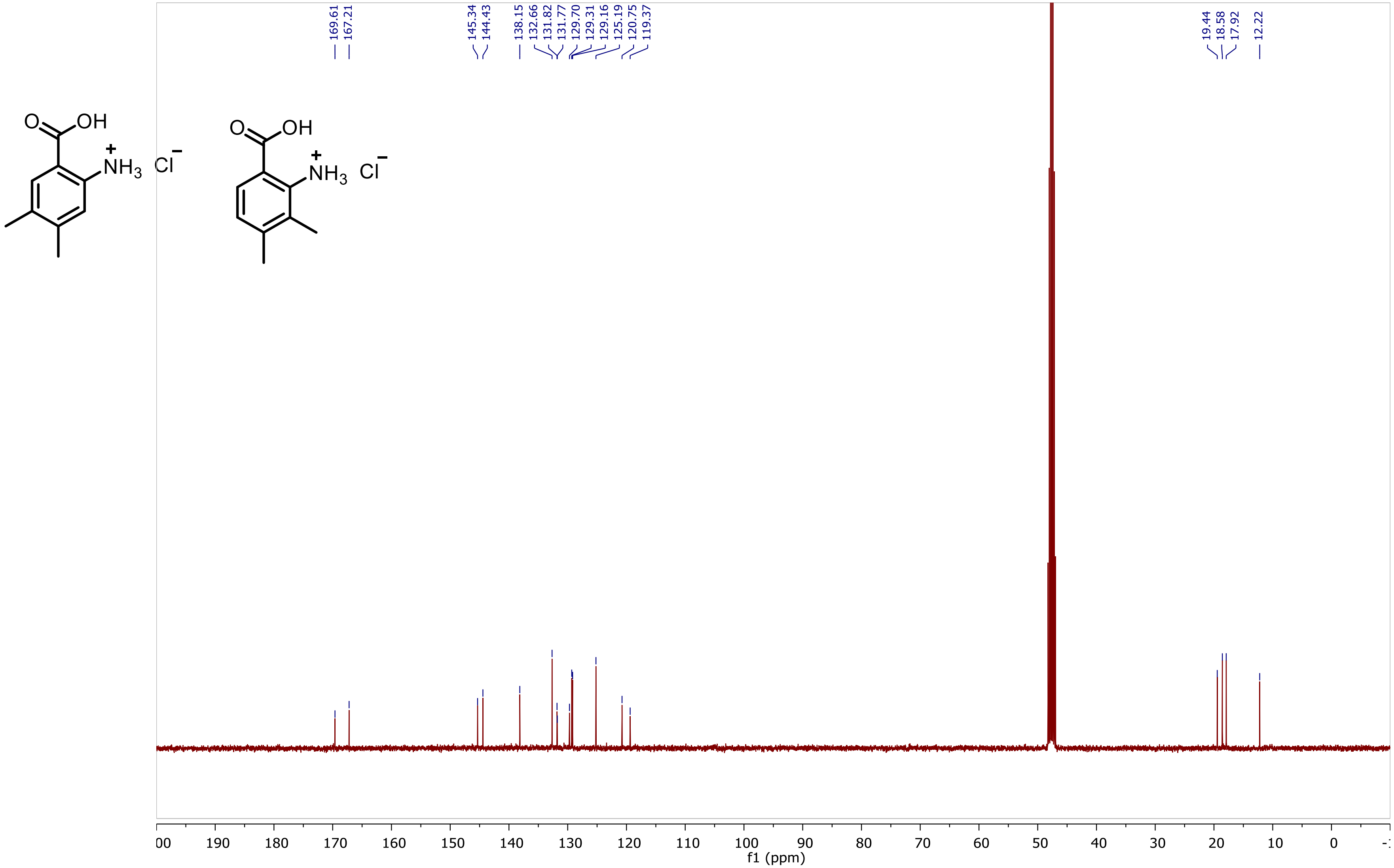

^{1}H NMR spectrum of 2-amino-3,5-di-*tert*-butylbenzoic acid hydrochloride **2i** in MeOD-d_4

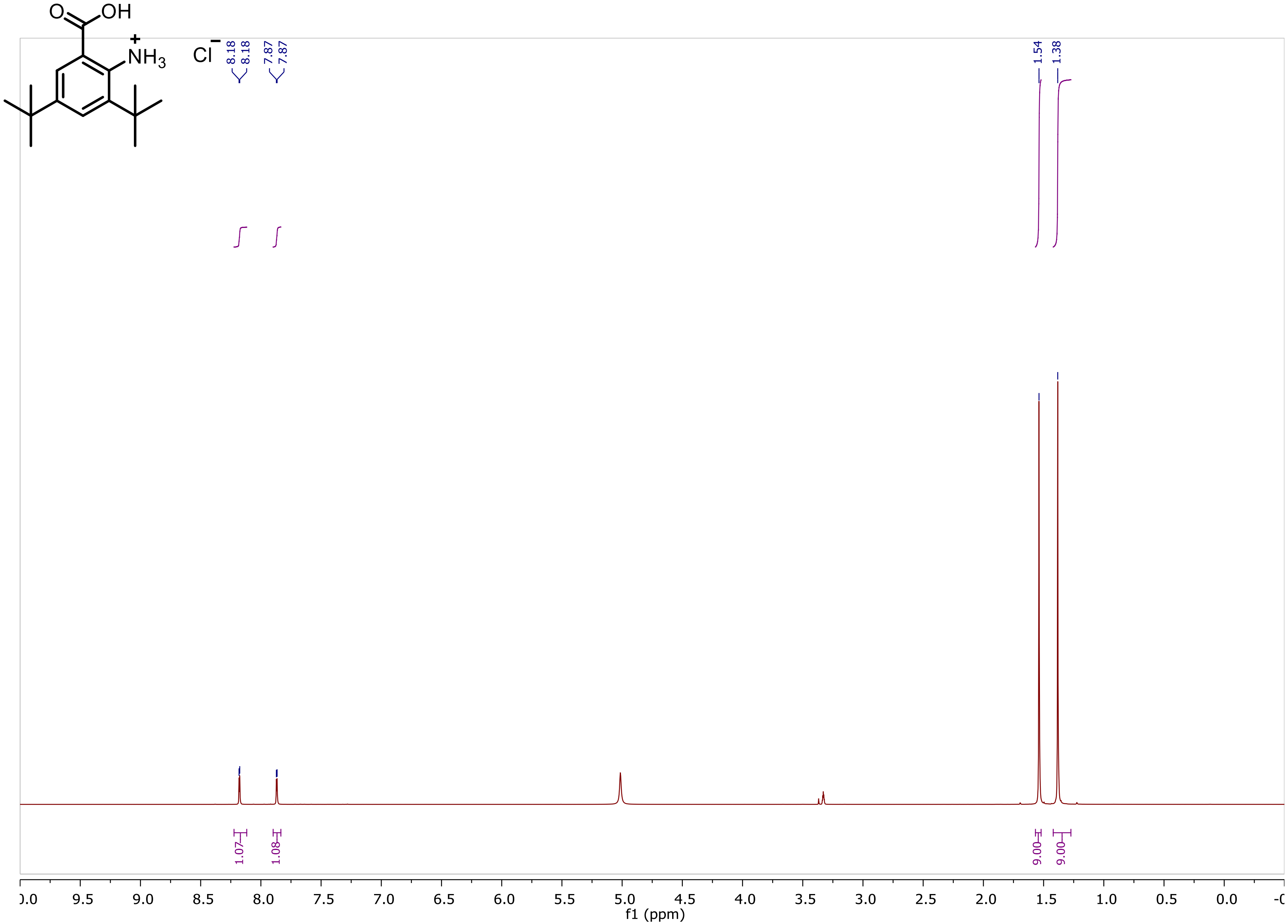

^{13}C NMR spectrum of 2-amino-3,5-di-*tert*-butylbenzoic acid hydrochloride **2i** in MeOD-d_4

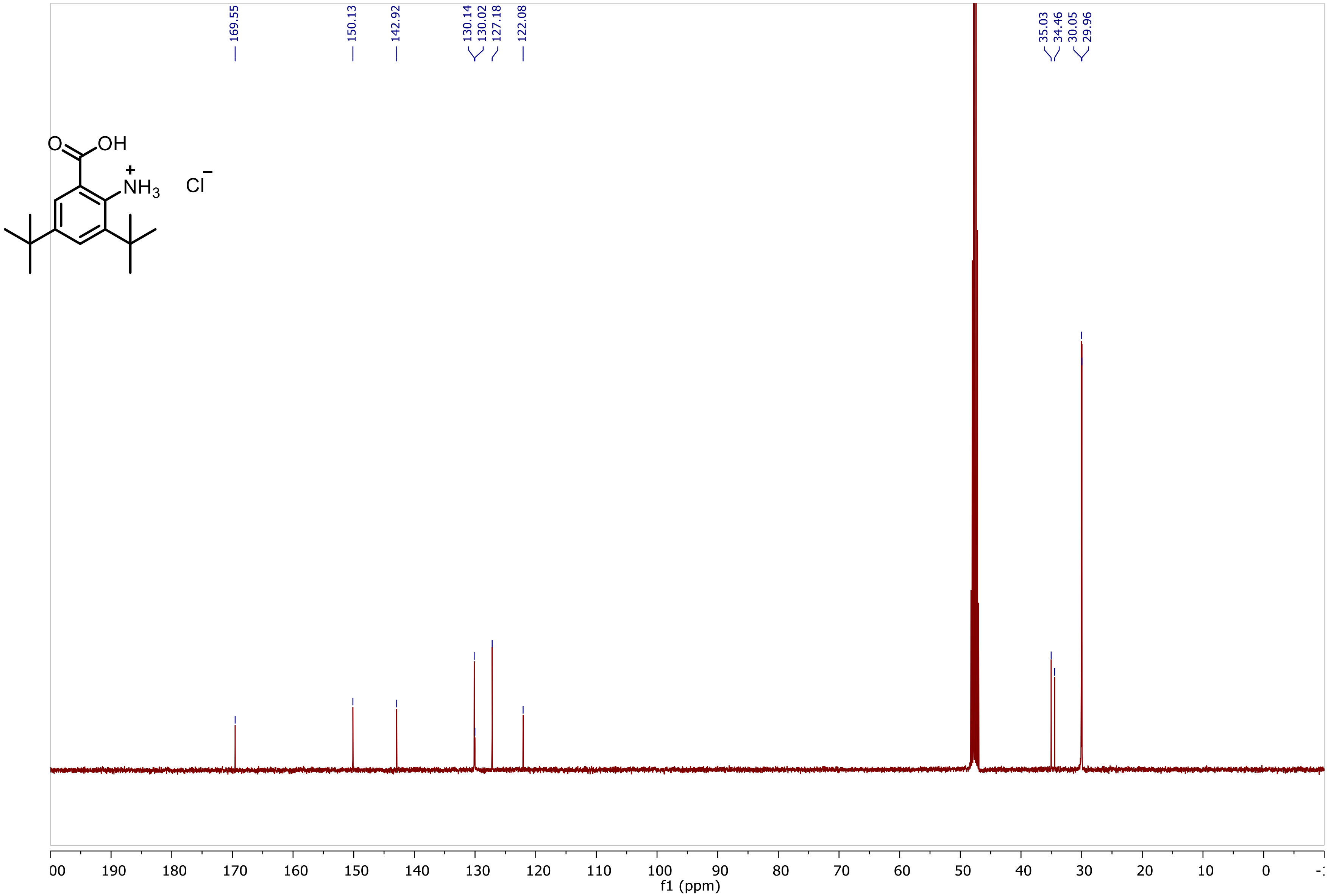

^{1}H NMR spectrum of 2-amino-4-fluorobenzoic acid **2j** in MeOD-d_4

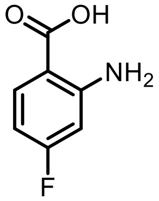

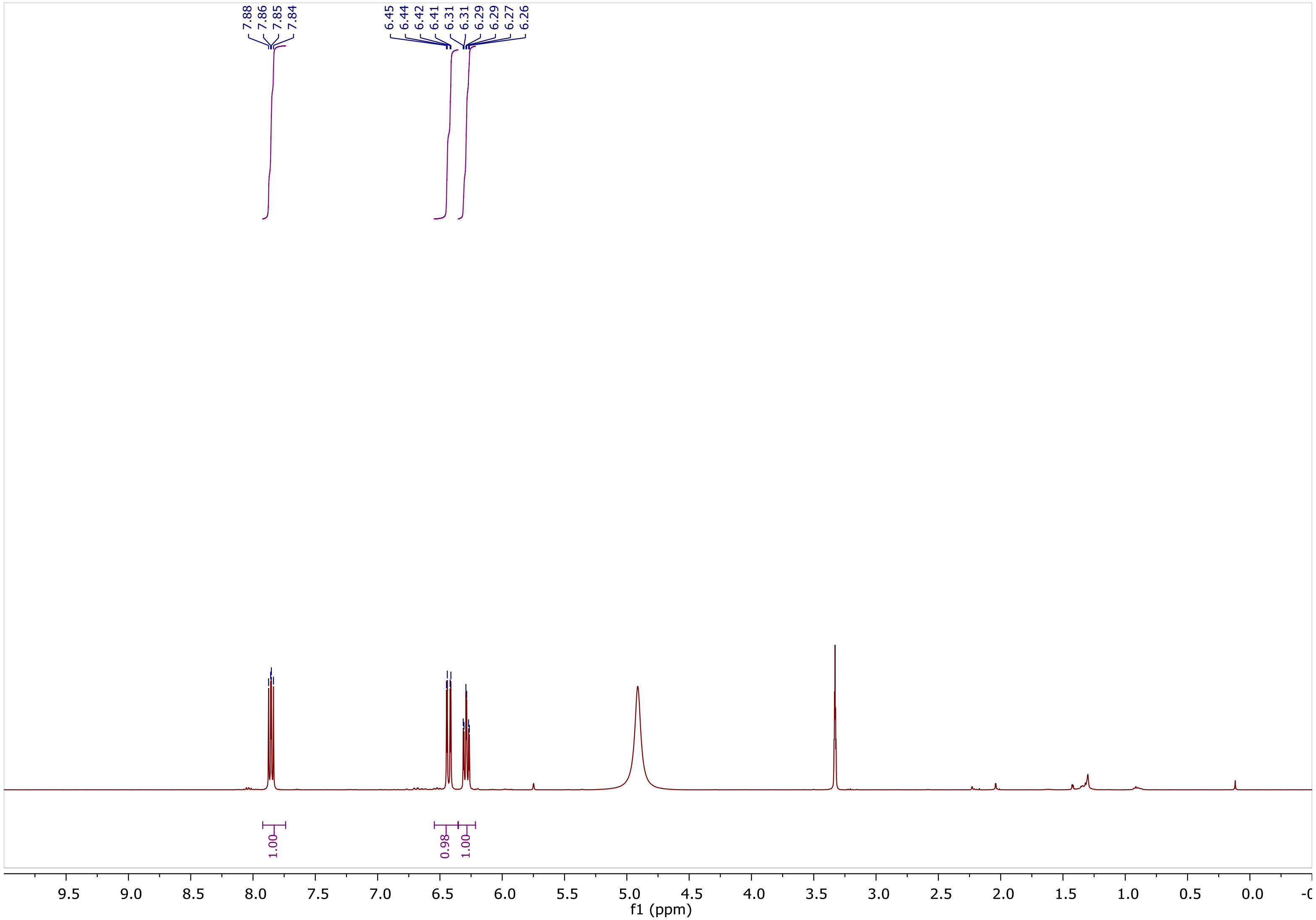

^{13}C NMR spectrum of 2-amino-4-fluorobenzoic acid **2j** in MeOD-d_4

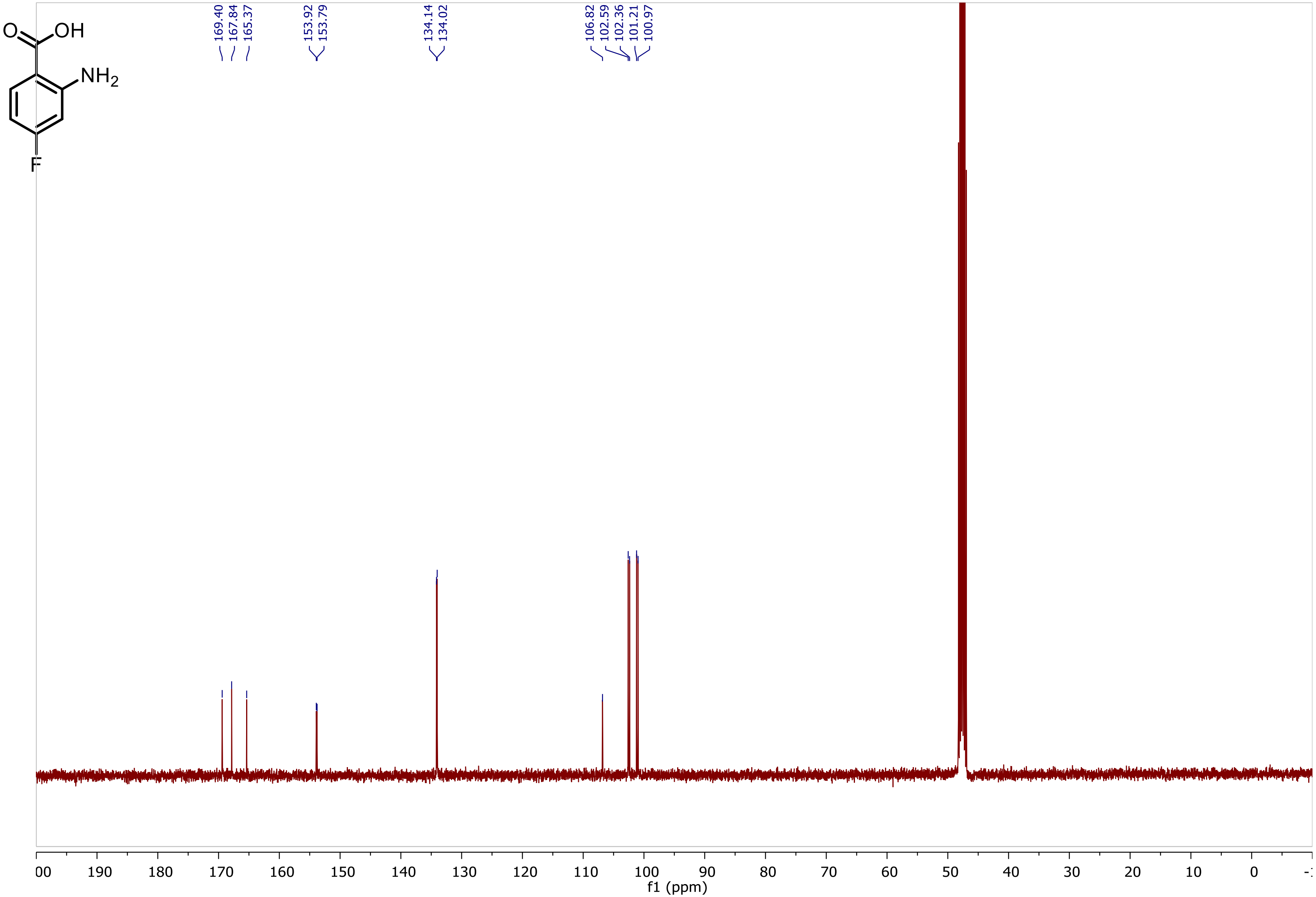

^{19}F NMR spectrum of 2-amino-4-fluorobenzoic acid **2j** in MeOD-d_4

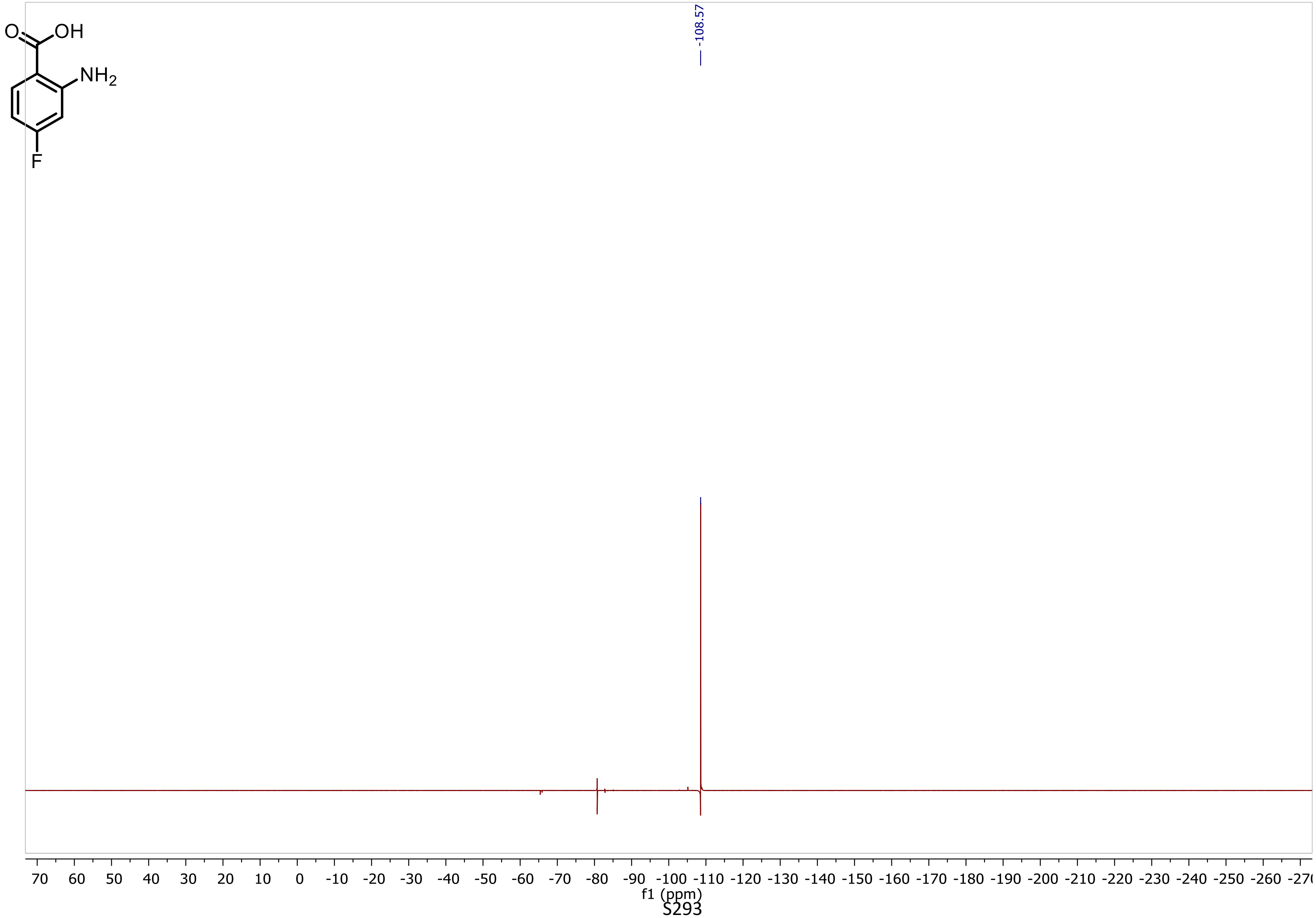

^{1}H NMR spectrum of 2-amino-4-chlorobenzoic acid **2m** in MeOD-d_4

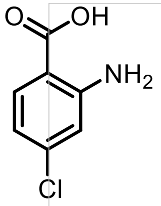

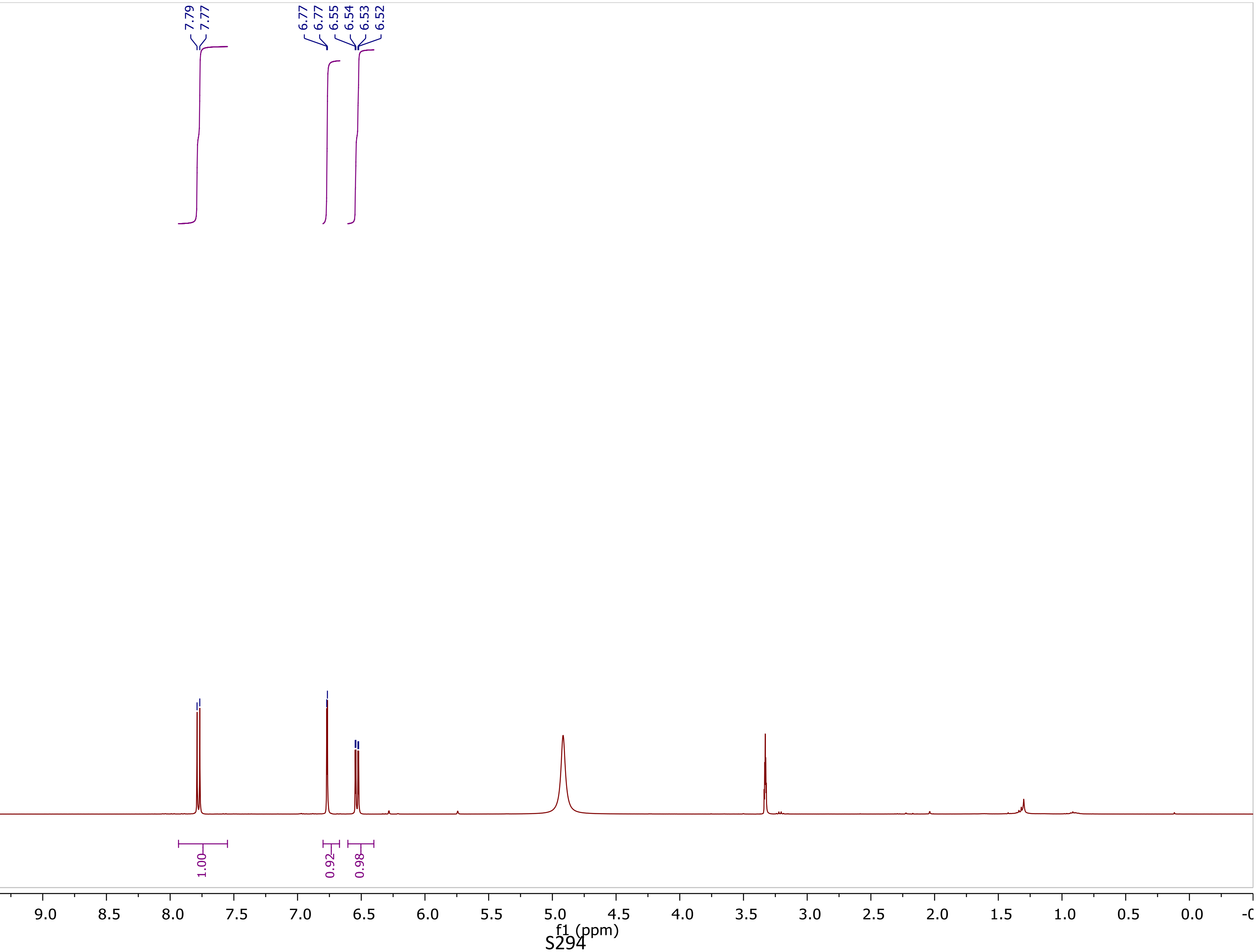

^{13}C NMR spectrum of 2-amino-4-chlorobenzoic acid **2m** in MeOD-d_4

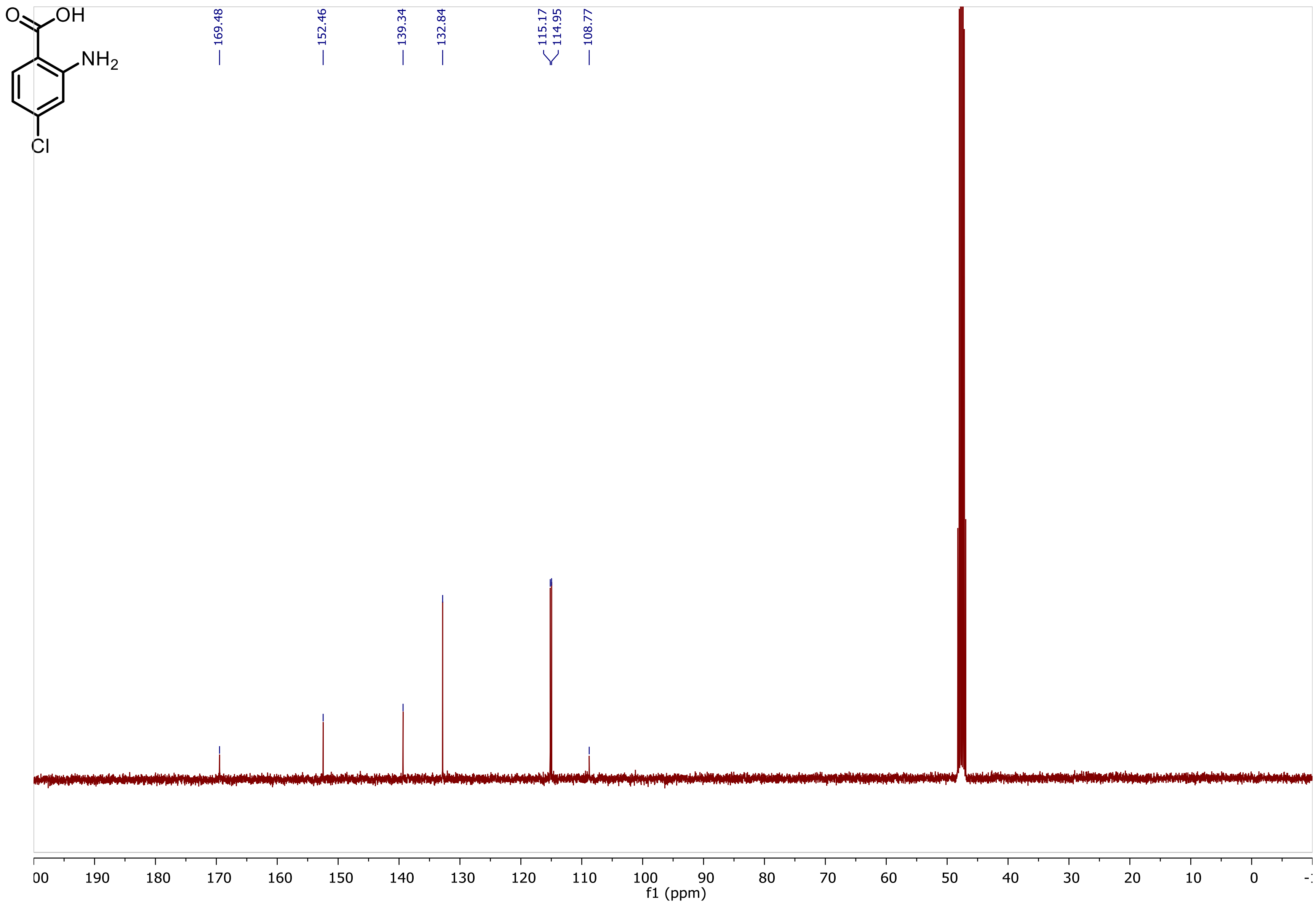

^{1}H NMR spectrum of 2-amino-4-bromobenzoic acid hydrochloride **2r** in MeOD-d_4

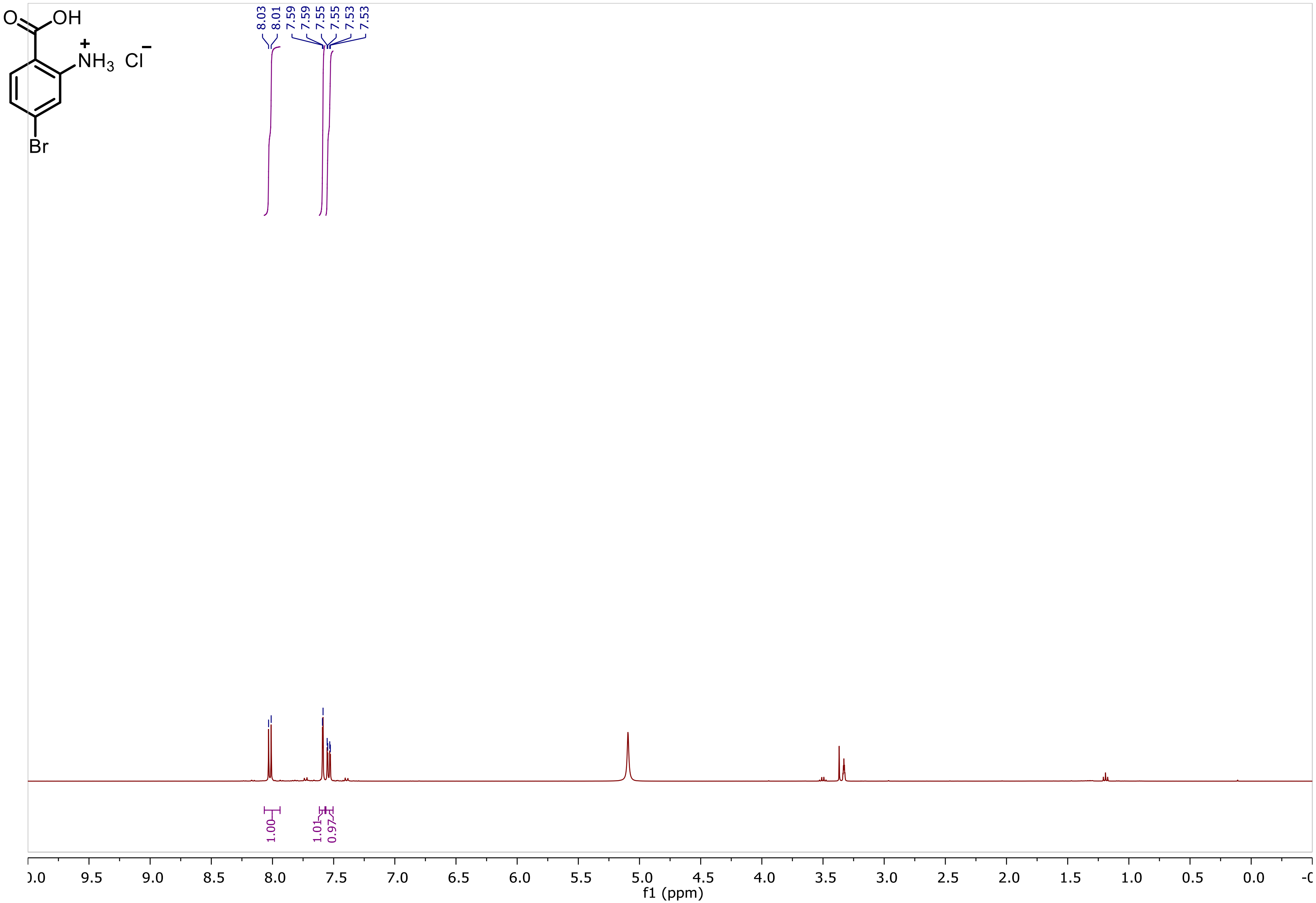

^{13}C NMR spectrum of 2-amino-4-bromobenzoic acid hydrochloride **2r** in MeOD-d_4

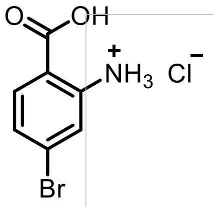

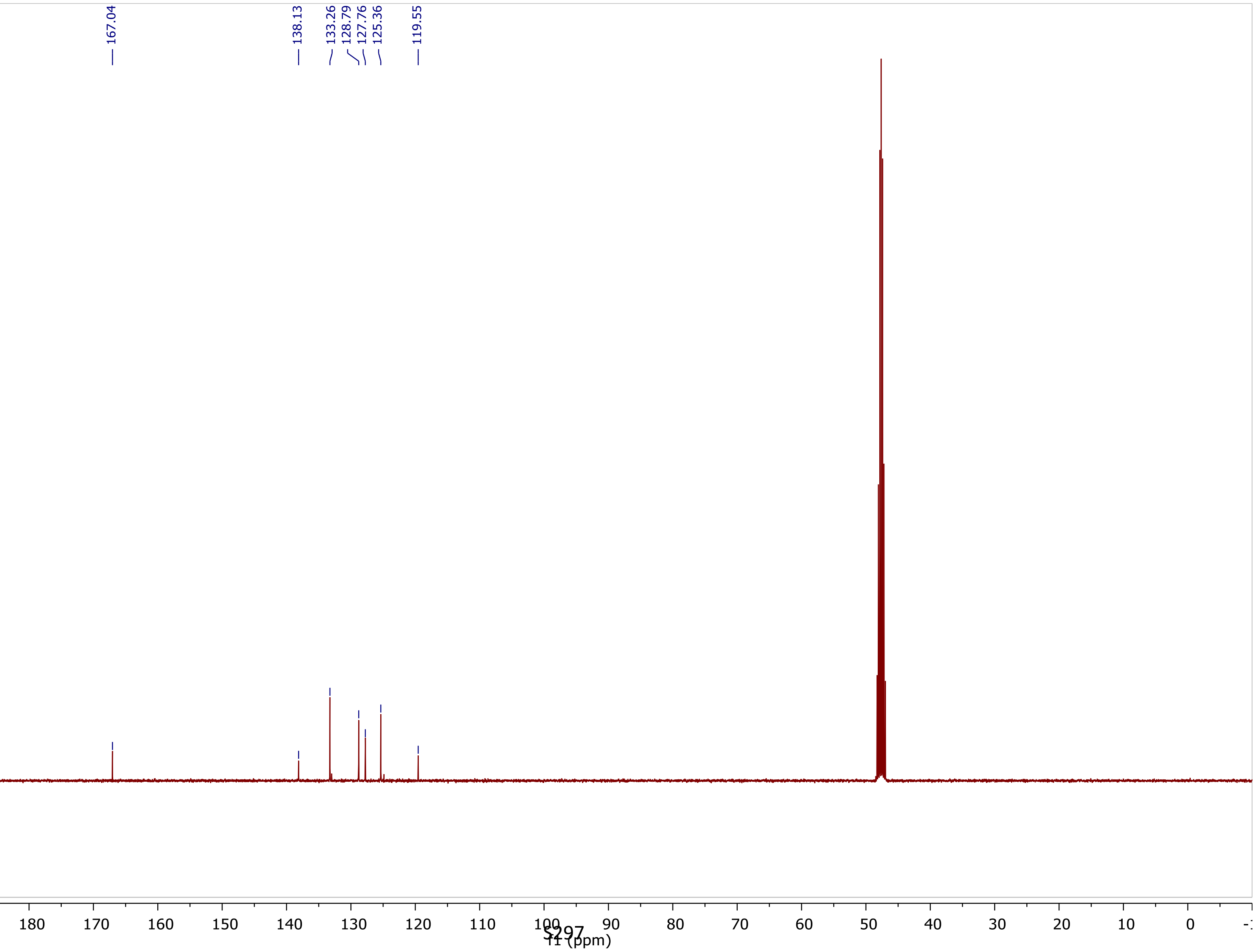

^{1}H NMR spectrum of 2-amino-5-fluorobenzoic acid hydrochloride **2k (a)** and 2-amino-3-fluorobenzoic acid hydrochloride ***iso*-2k (b)** in MeOD-

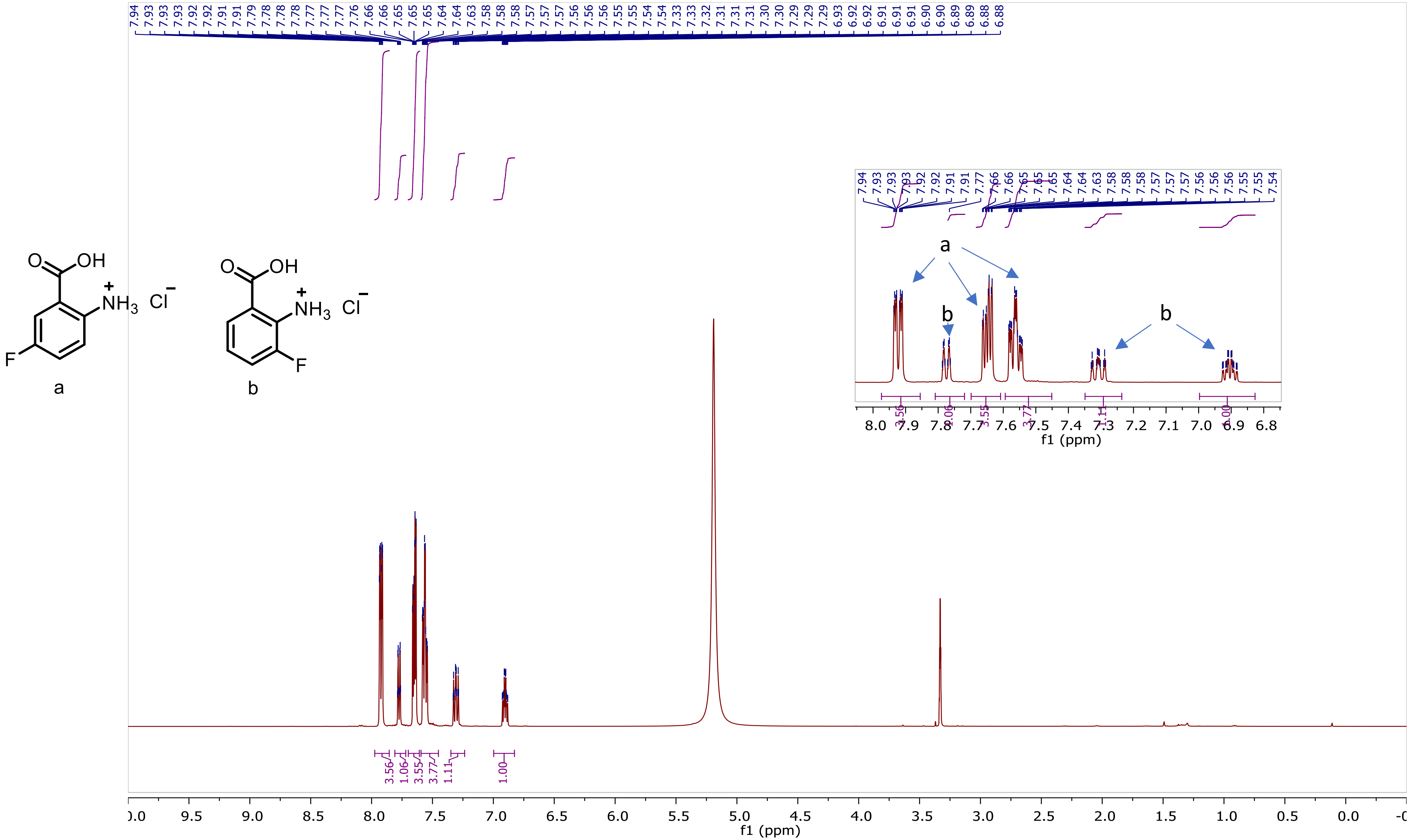

^{13}C NMR spectrum of 2-amino-5-fluorobenzoic acid hydrochloride **2k** and 2-amino-3-fluorobenzoic acid hydrochloride ***iso*-2k** in MeOD-d_4

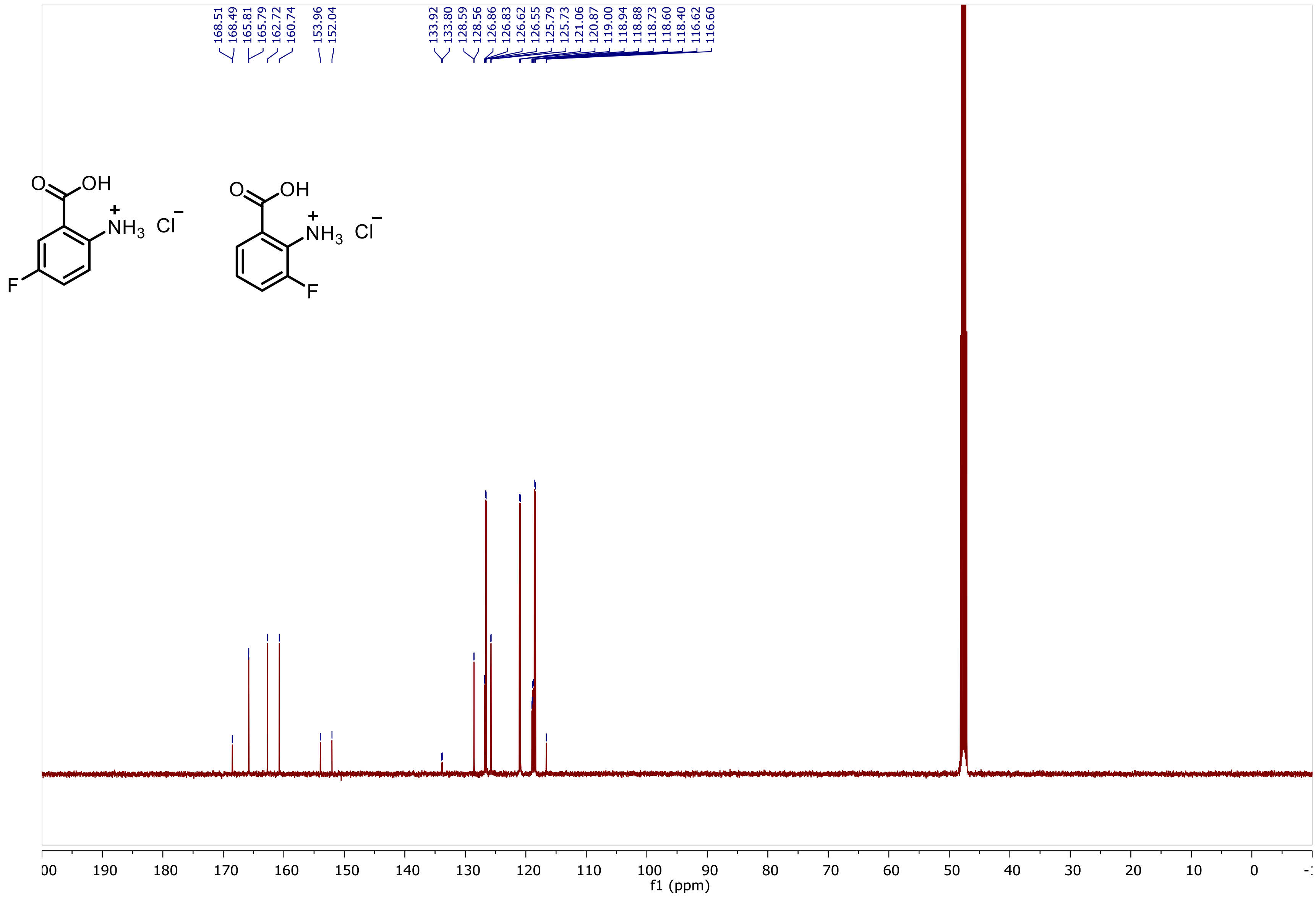

^{19}F NMR spectrum of 2-amino-5-fluorobenzoic acid hydrochloride **2k** and 2-amino-3-fluorobenzoic acid hydrohchloride ***iso*-2k** in MeOD-d_4

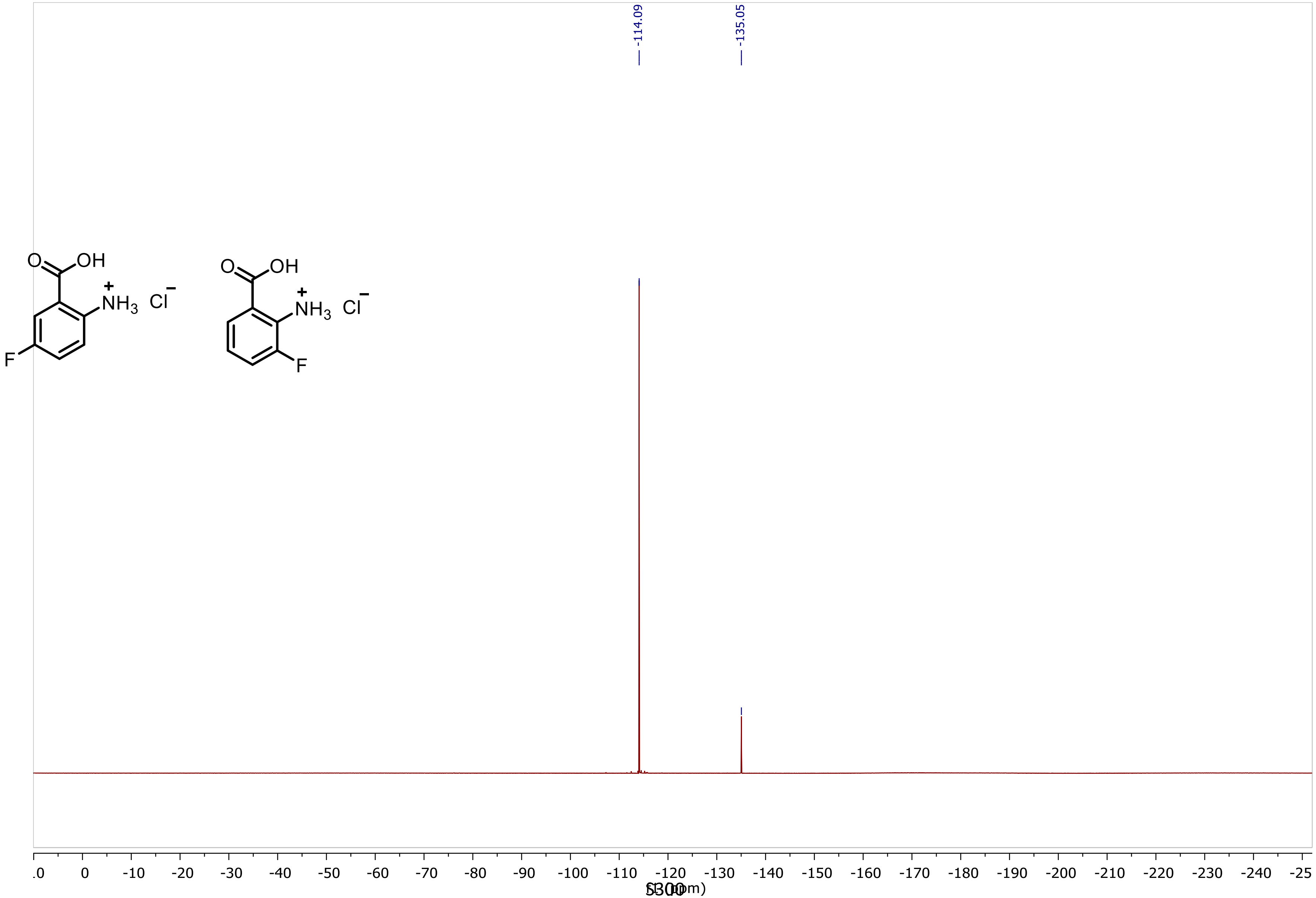

^{1}H NMR spectrum of 6-amino-3-fluoro-2-methylbenzoic acid hydrochloride **2l** in MeOD-d_4

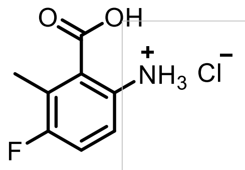

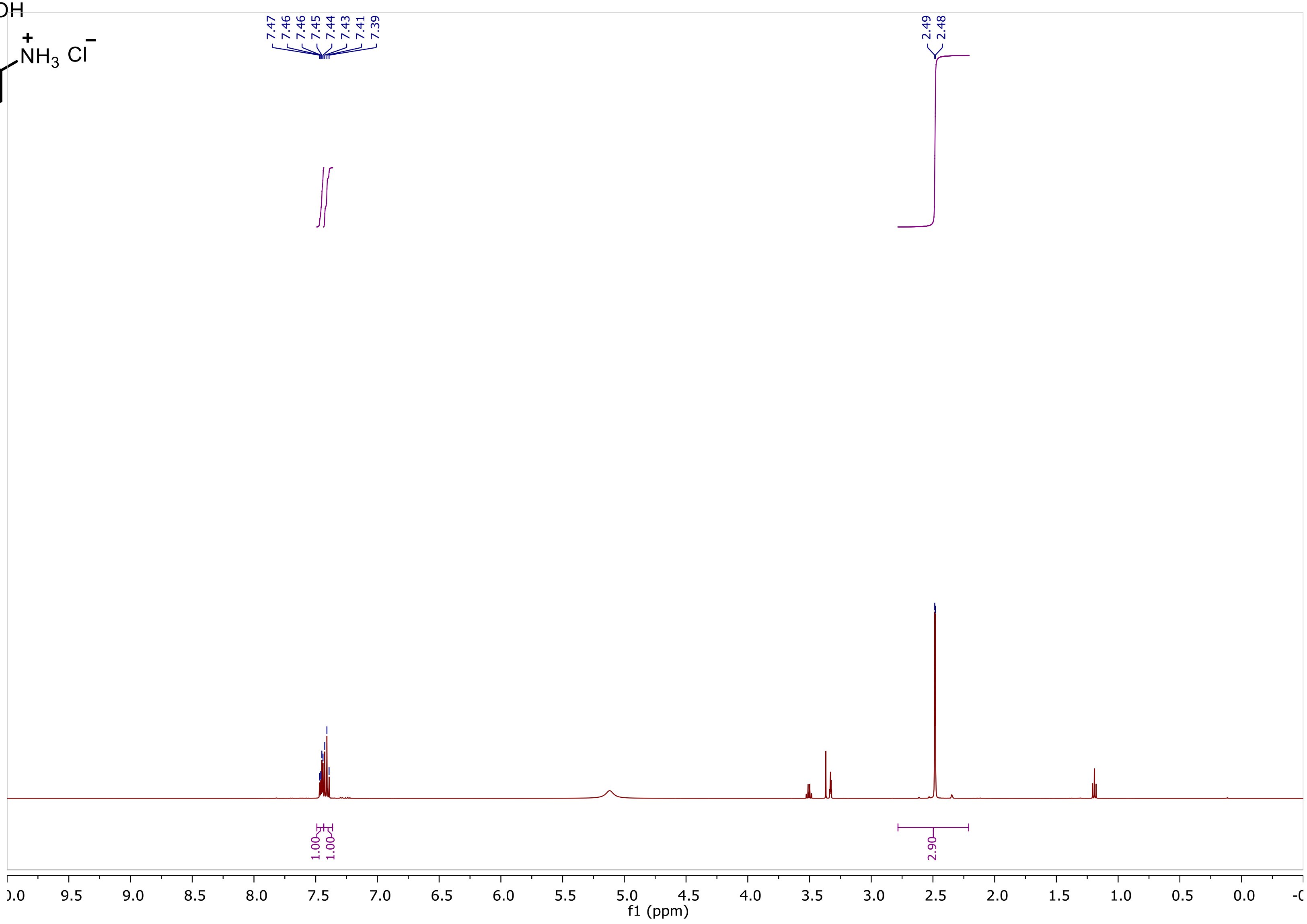

^{13}C NMR spectrum of 6-amino-3-fluoro-2-methylbenzoic acid hydrochloride **2l** in MeOD-d_4

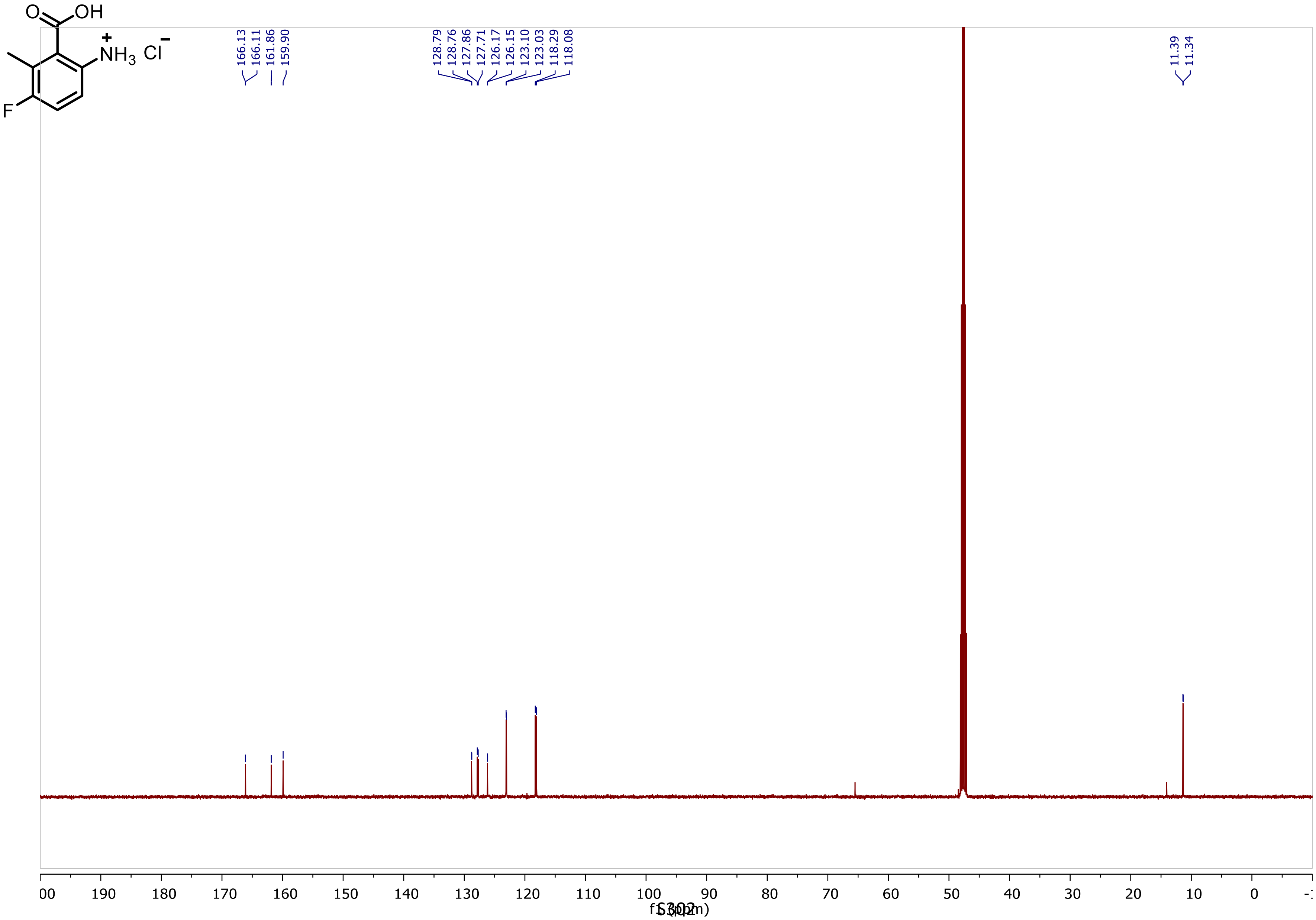

^{19}F NMR spectrum of 6-amino-3-fluoro-2-methylbenzoic acid hydrochloride **2l** in MeOD-d_4

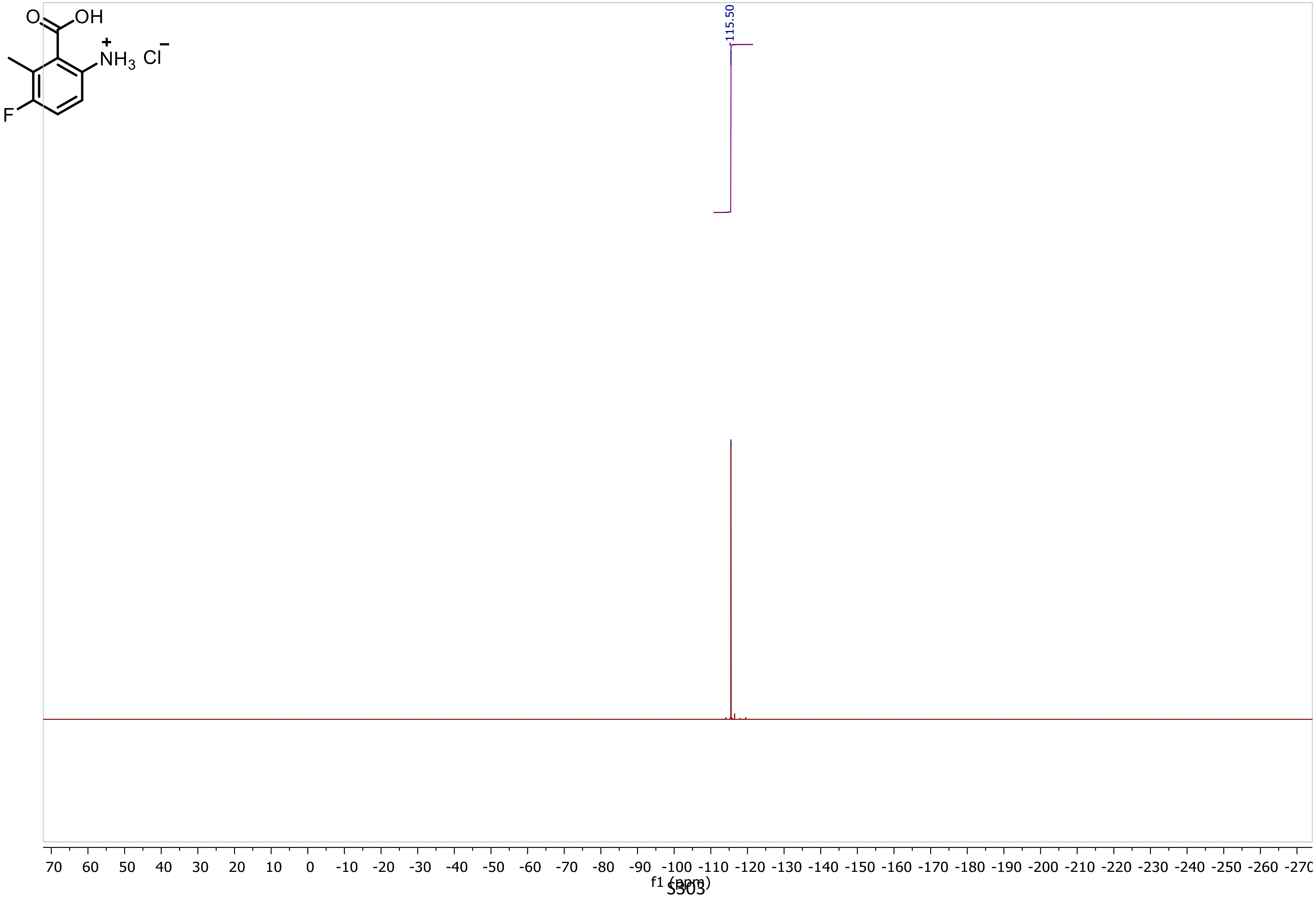

^{1}H NMR spectrum of 6-amino-2-chloro-3-methylbenzoic acid hydrochloride **2n** in MeOD-d_4

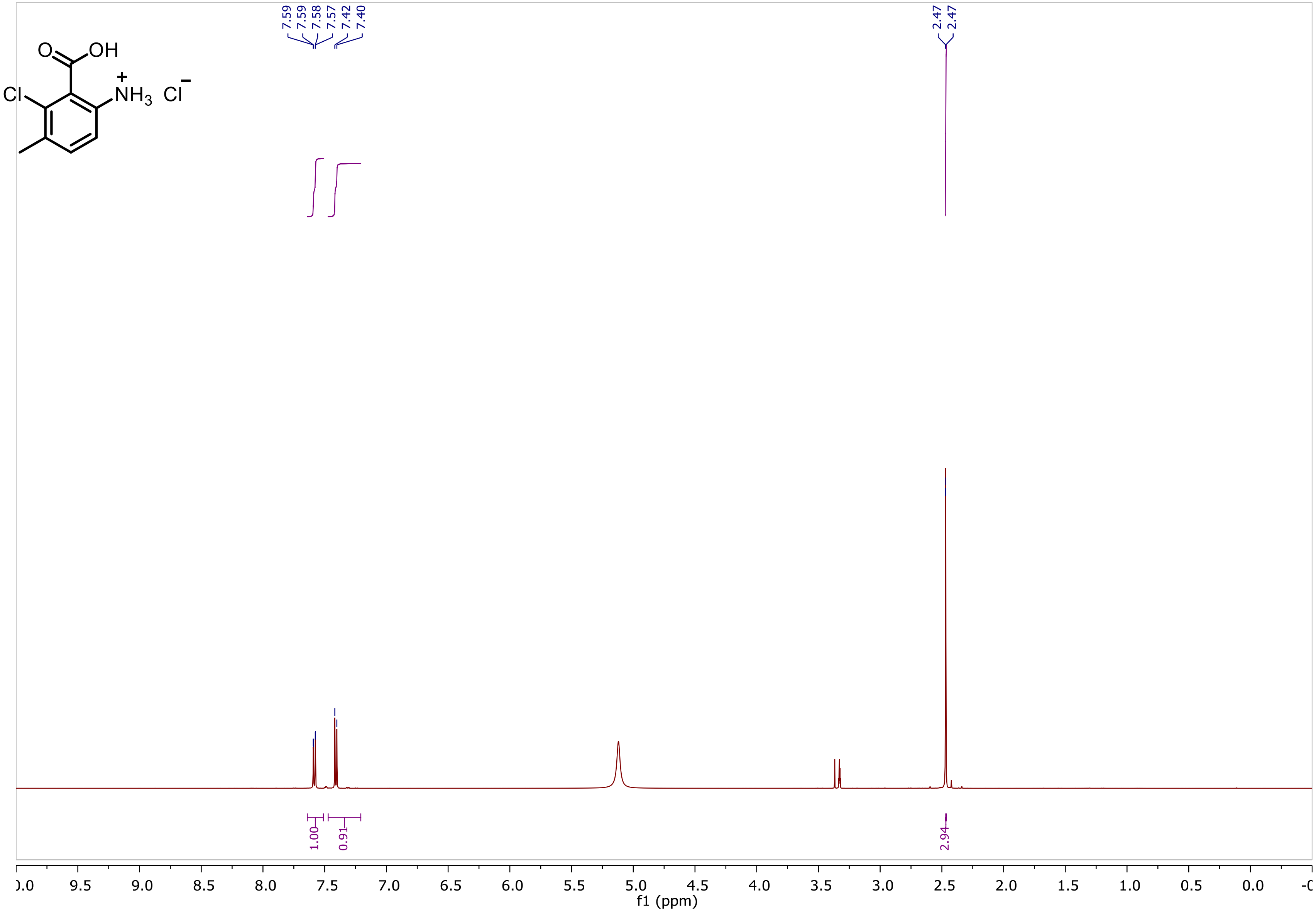

^{13}C NMR spectrum of 6-amino-2-chloro-3-methylbenzoic acid hydrochloride **2n** in MeOD-d_4

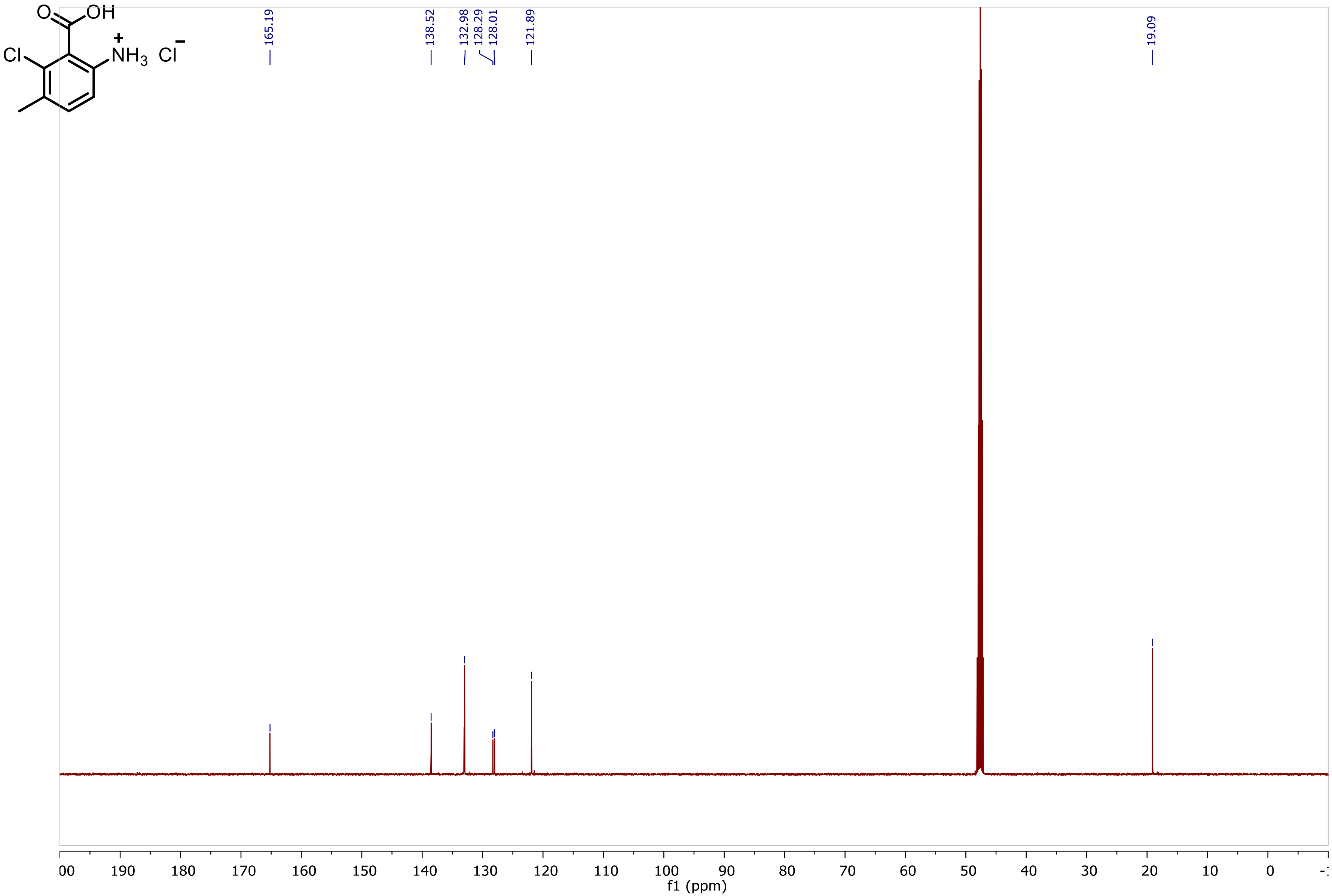

^{1}H NMR spectrum of 2-amino-6-chlorobenzoic acid hydrochloride **2o** in MeOD-d_4

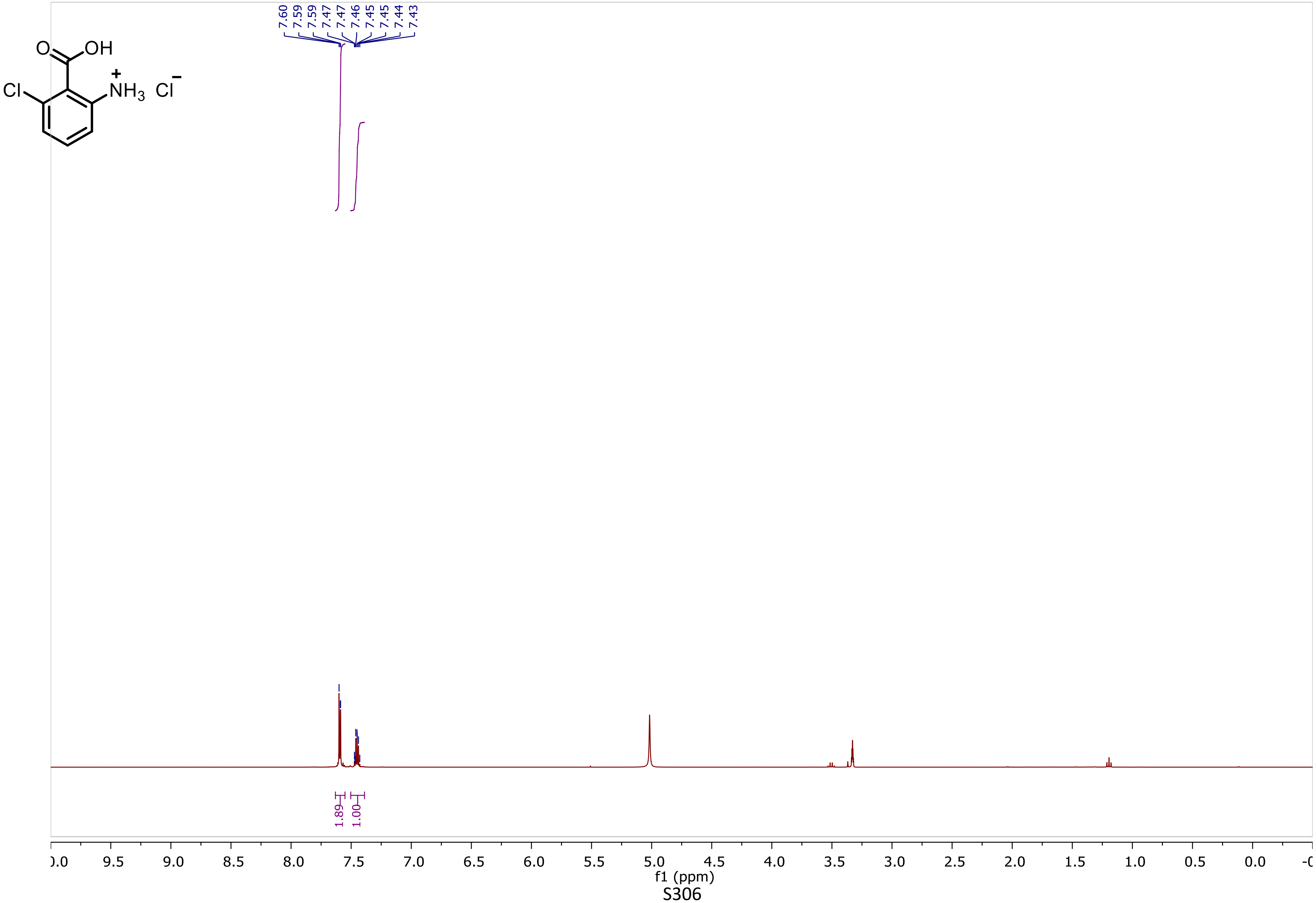

^{13}C NMR spectrum of 2-amino-6-chlorobenzoic acid hydrochloride **2o** in MeOD-d_4

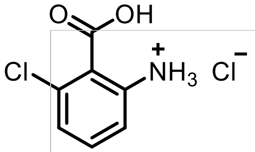

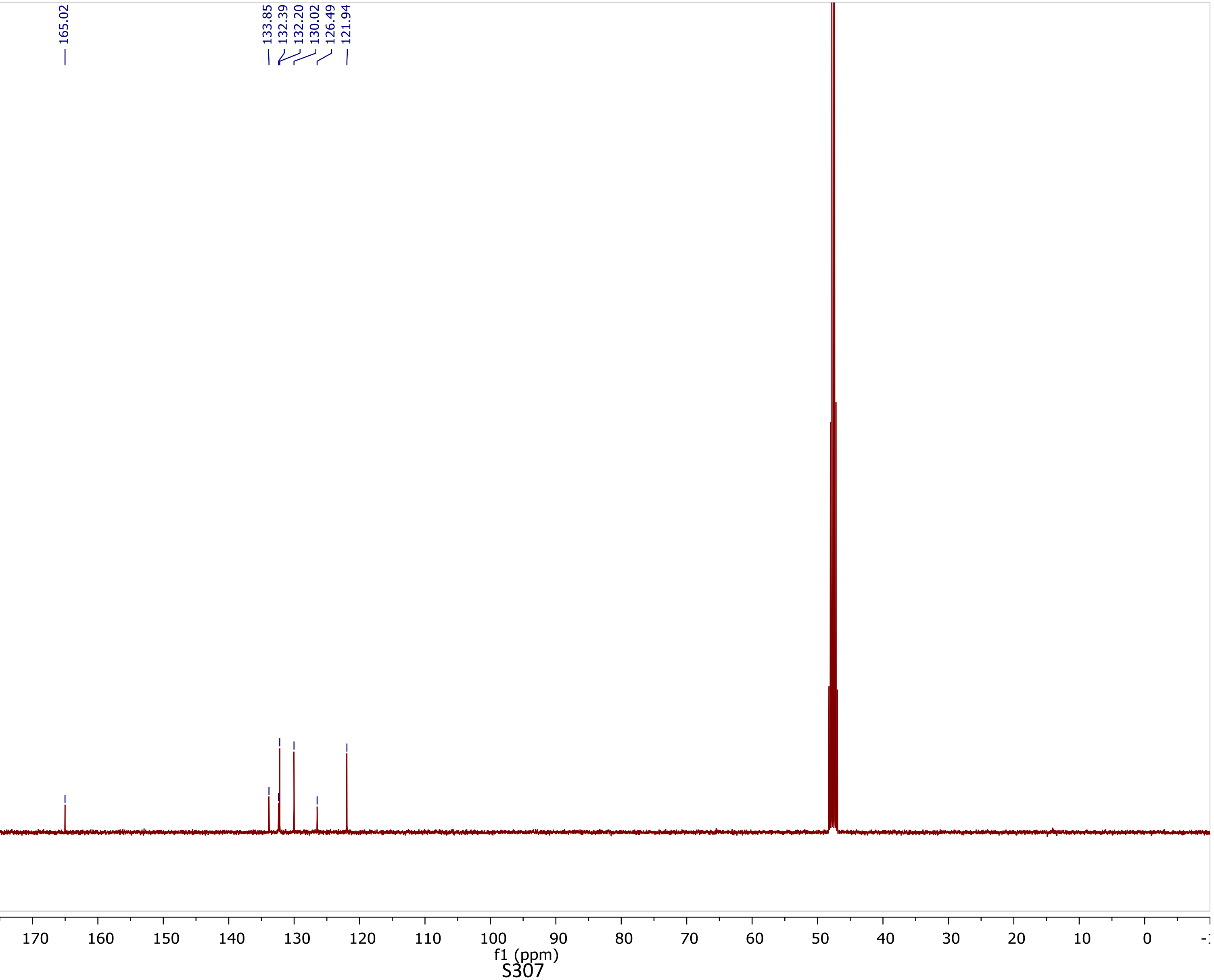

^{1}H NMR spectrum of 2-amino-5-chlorobenzoic acid hydrochloride **2p (a)** and 2-amino-3-chlorobenzoic acid hydrochloride ***iso*-2p (b)** in MeOD-d_4

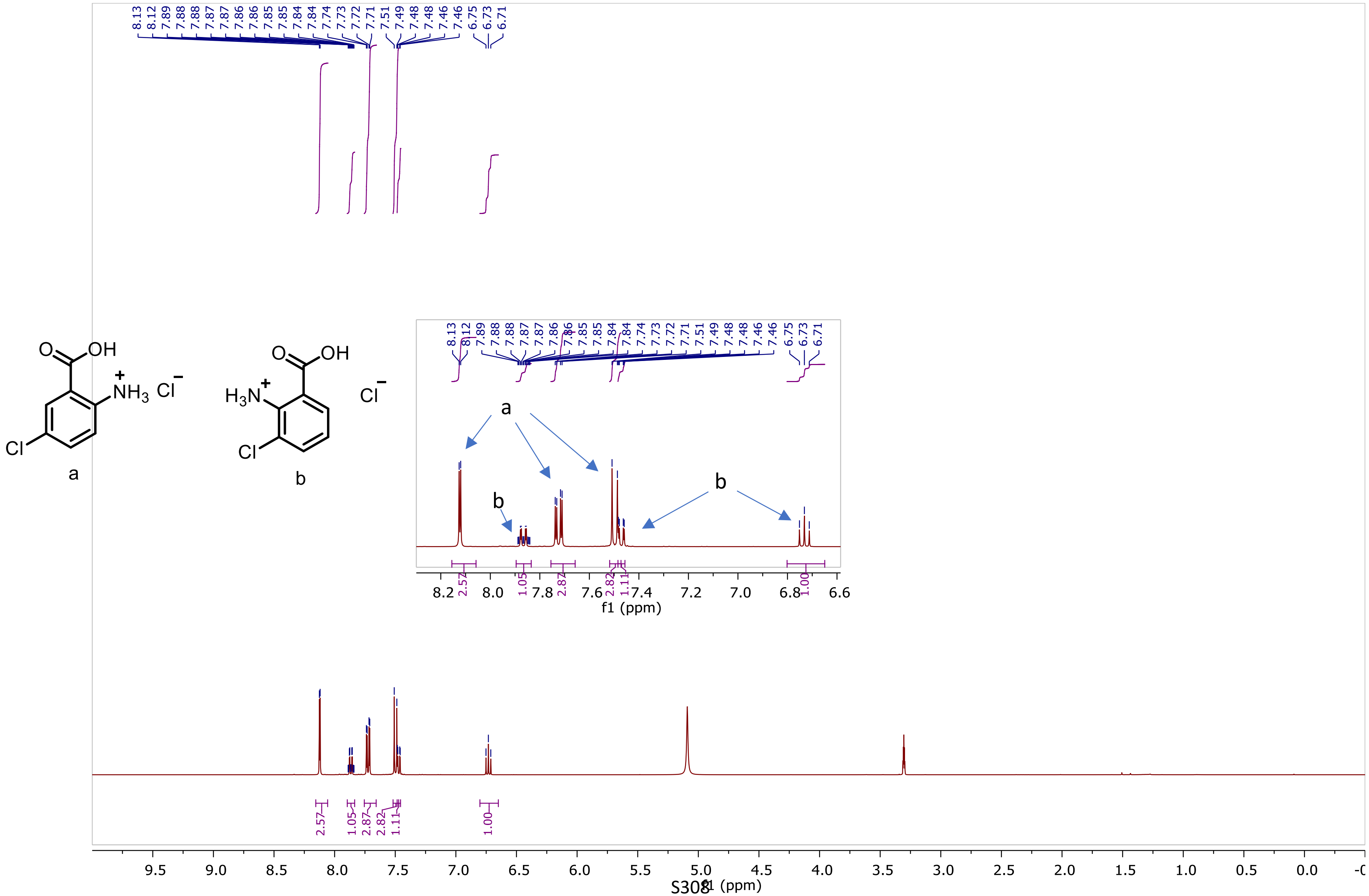

^{13}C NMR spectrum of 2-amino-5-chlorobenzoic acid hydrochloride **2p** and 2-amino-3-chlorobenzoic acid hydrochloride ***iso*-2p** in MeOD-d_4

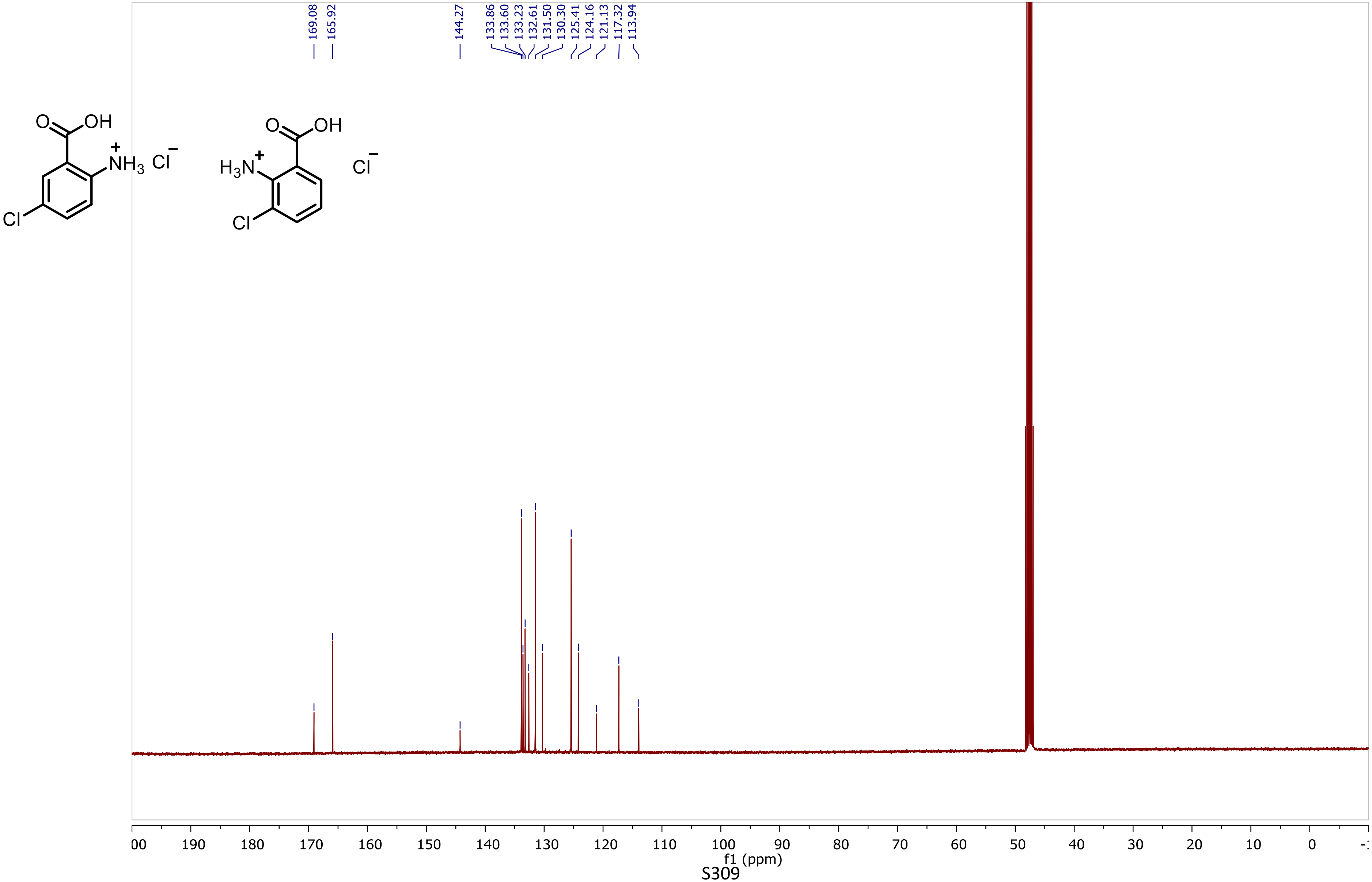

^{1}H NMR spectrum of 6-amino-3-chloro-2-methylbenzoic acid hydrochloride **2q** in MeOD-d_4

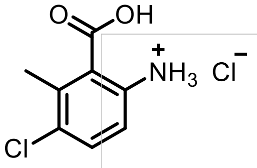

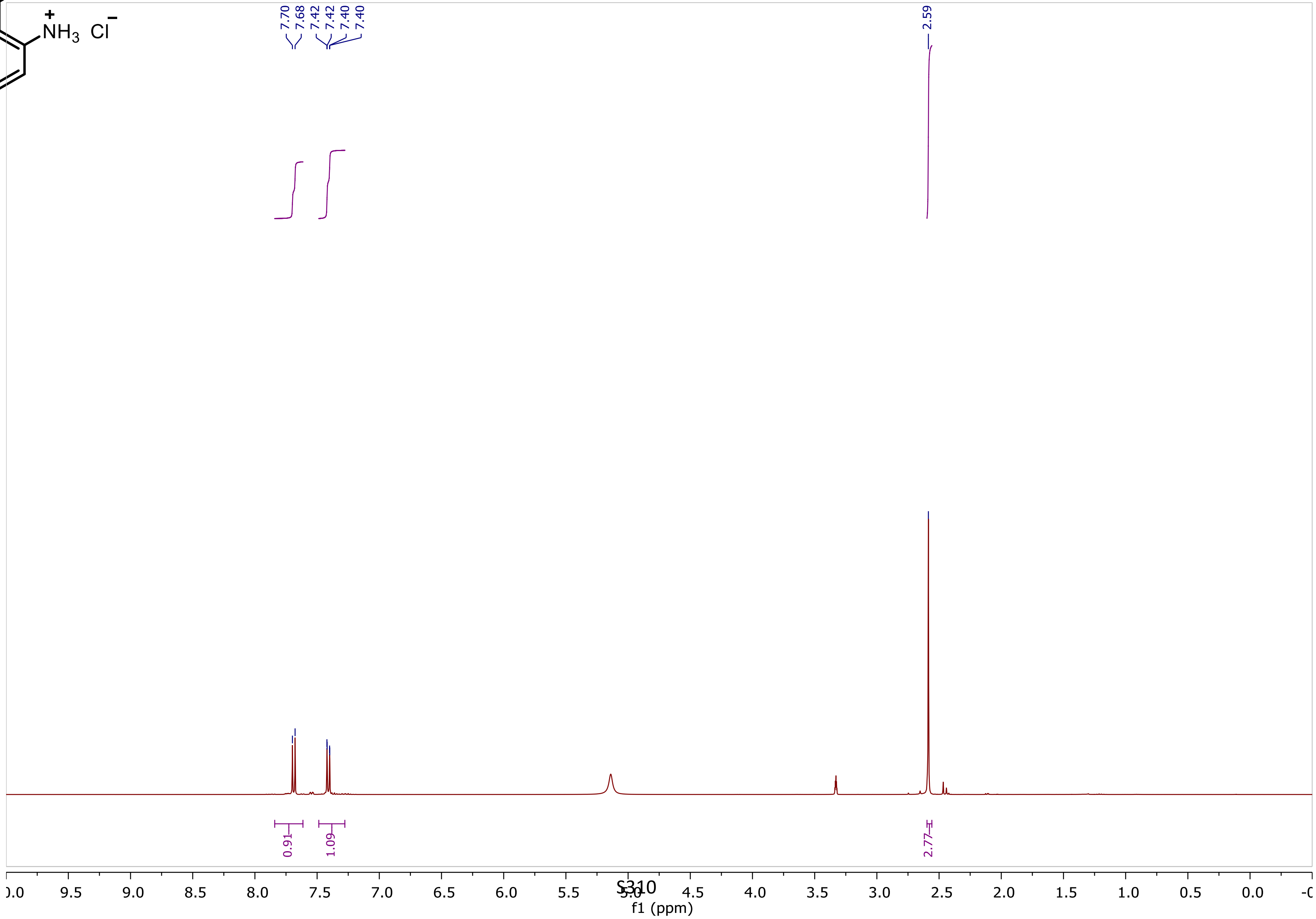

^{13}C NMR spectrum of 6-amino-3-chloro-2-methylbenzoic acid hydrochloride **2q** in MeOD-d_4

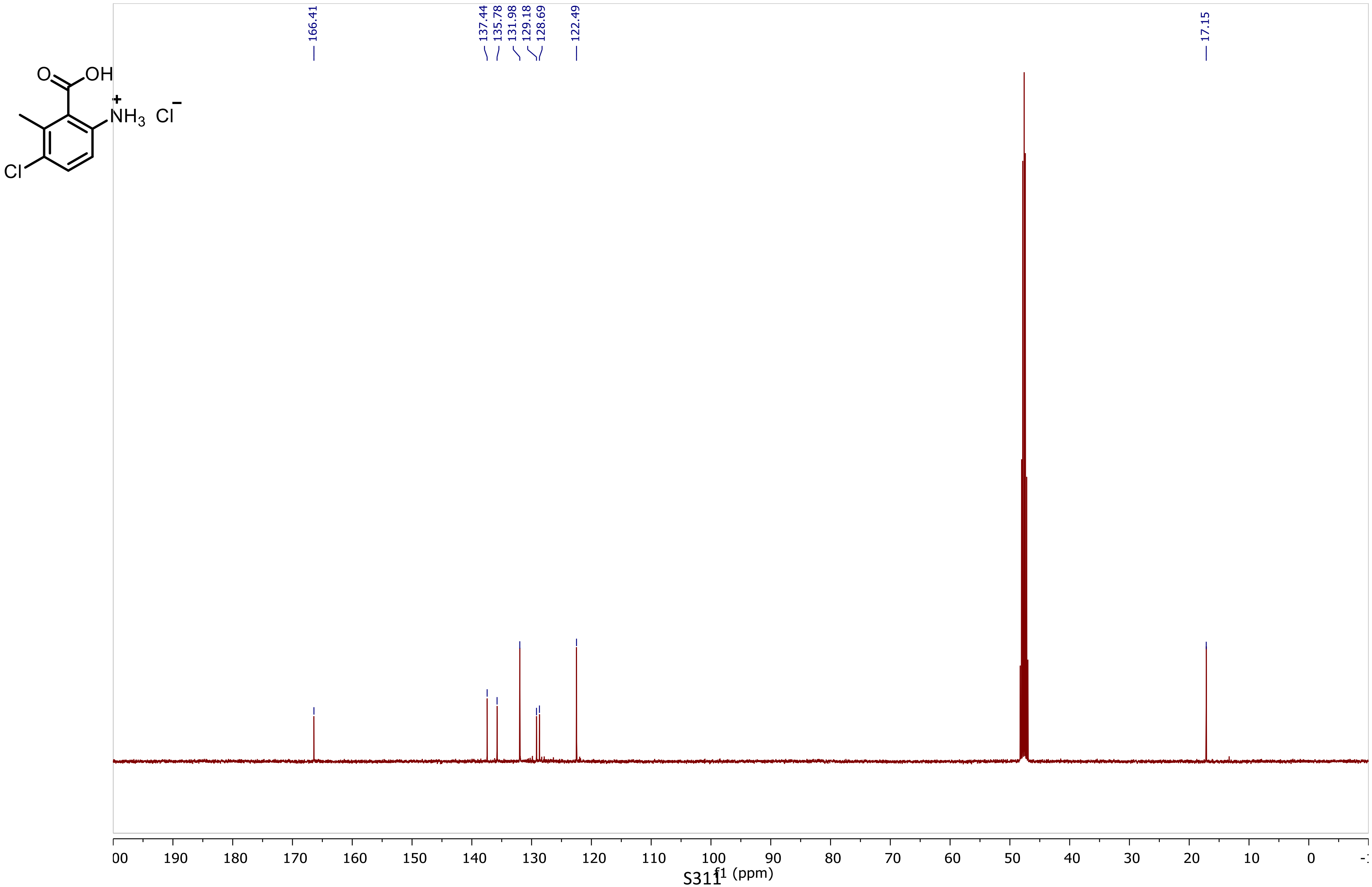

^{1}H NMR spectrum of 6-chloro-5-methylquinazolin-4(3*H*)-one **S2q** in DMSO-d_6

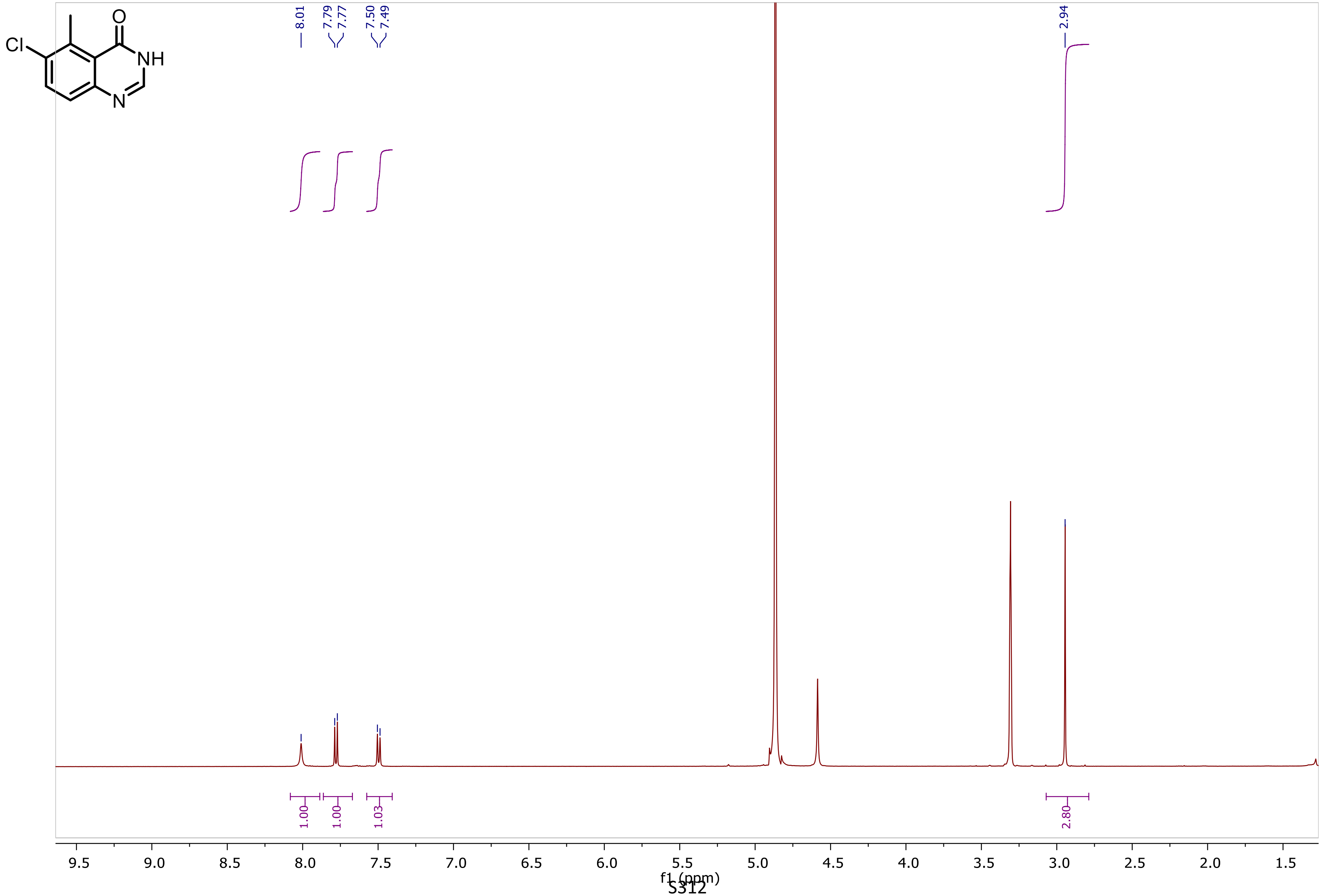

^{13}C NMR spectrum of 6-chloro-5-methylquinazolin-4(3*H*)-one **S2q** in DMSO-d_6

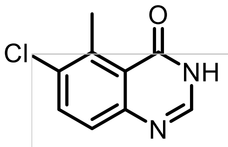

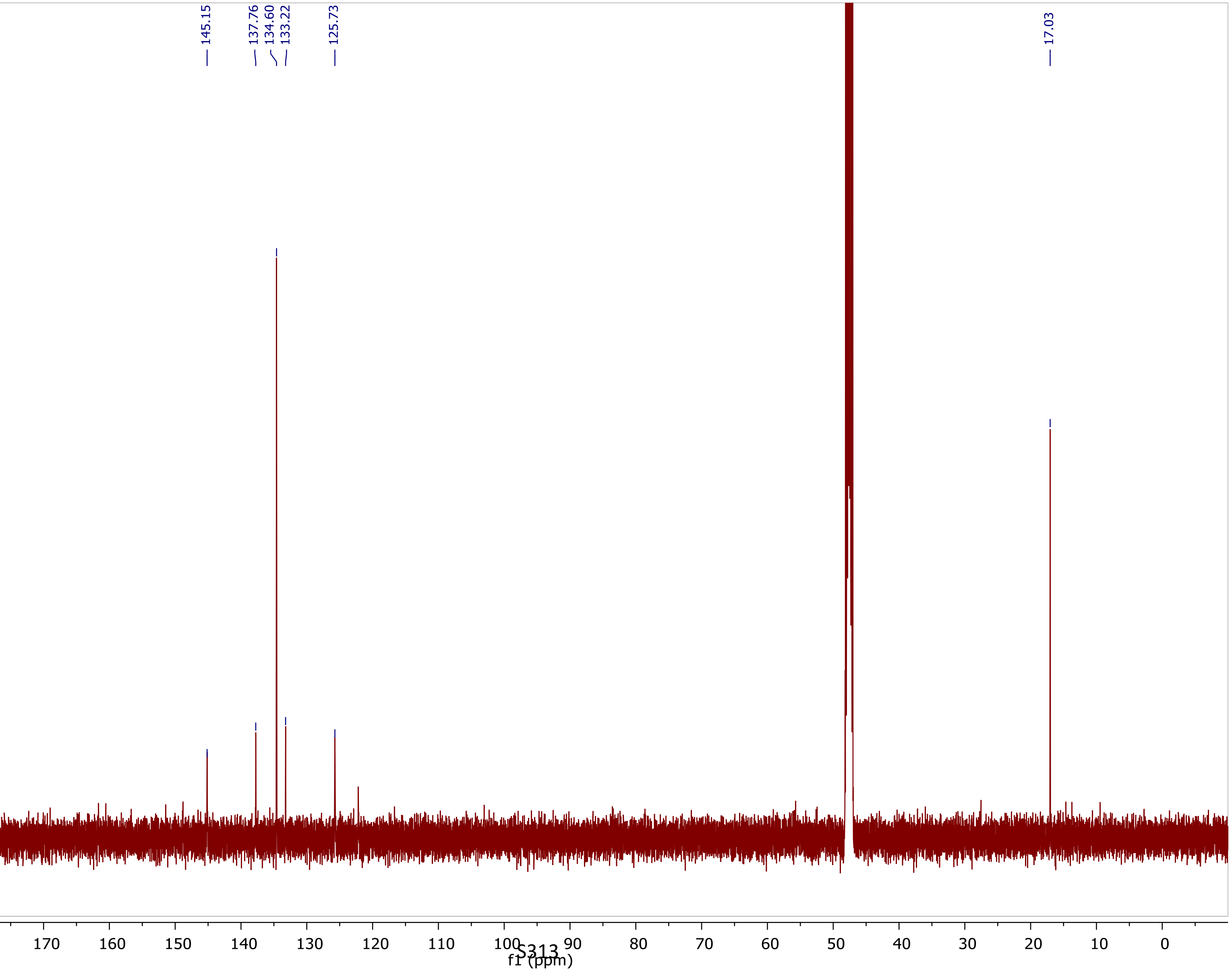

^{1}H NMR spectrum of 2-amino-6-bromobenzoic acid **2s** in MeOD-d_4

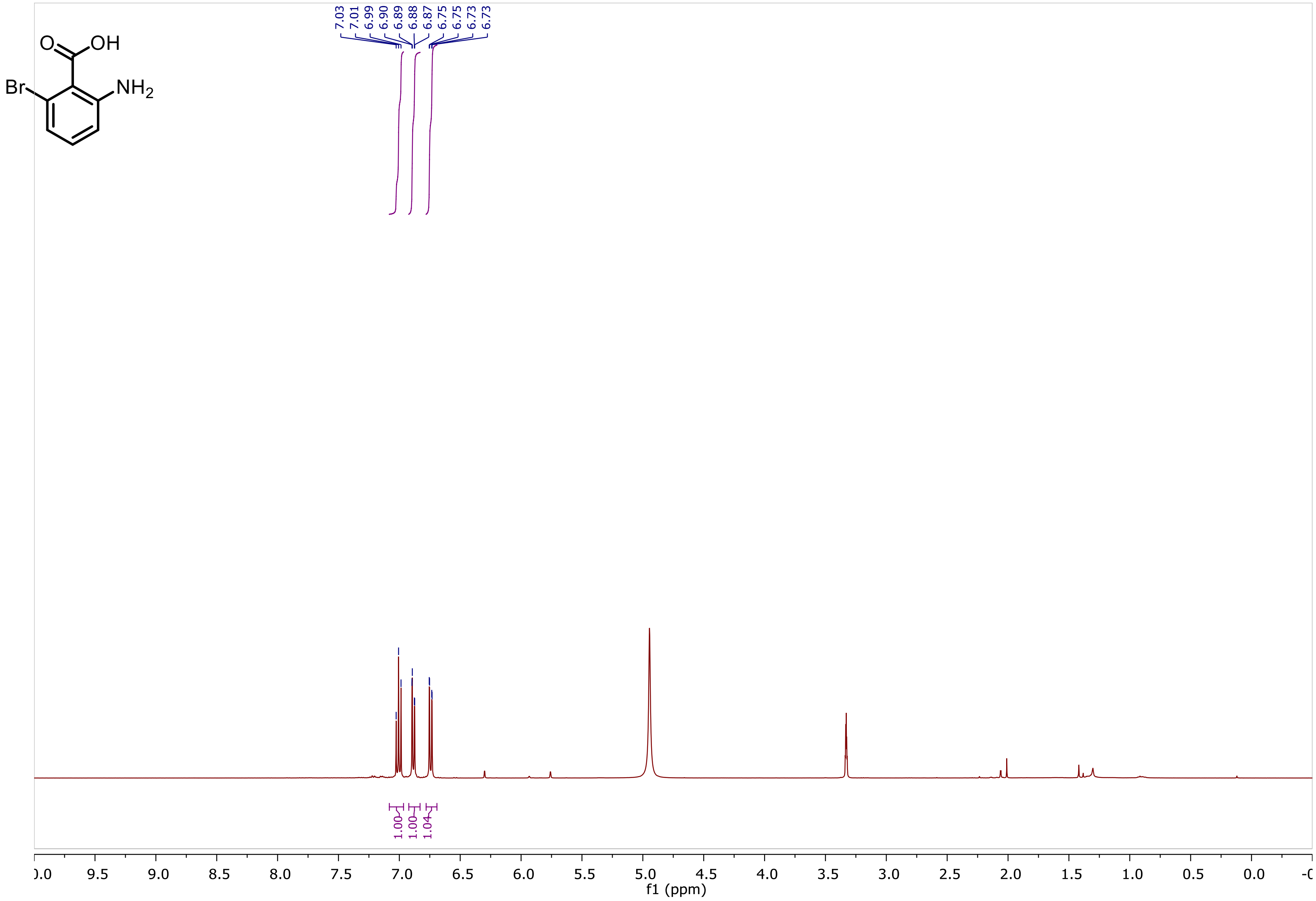

^{13}C NMR spectrum of 2-amino-6-bromobenzoic acid **2s** in MeOD-d_4

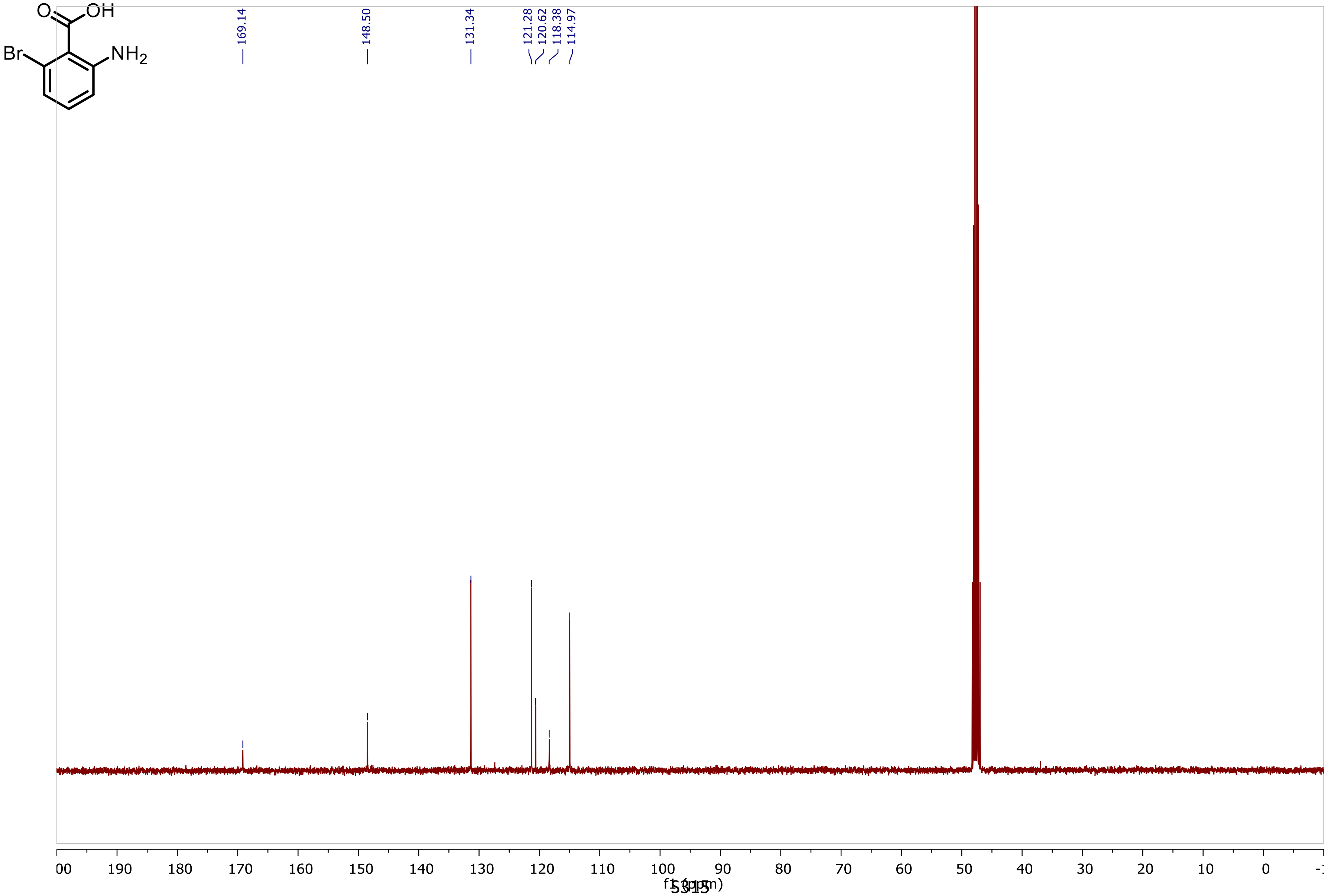

^{1}H NMR spectrum of 6-amino-2-bromo-3-methylbenzoic acid hydrochloride **2t** in MeOD-d_4

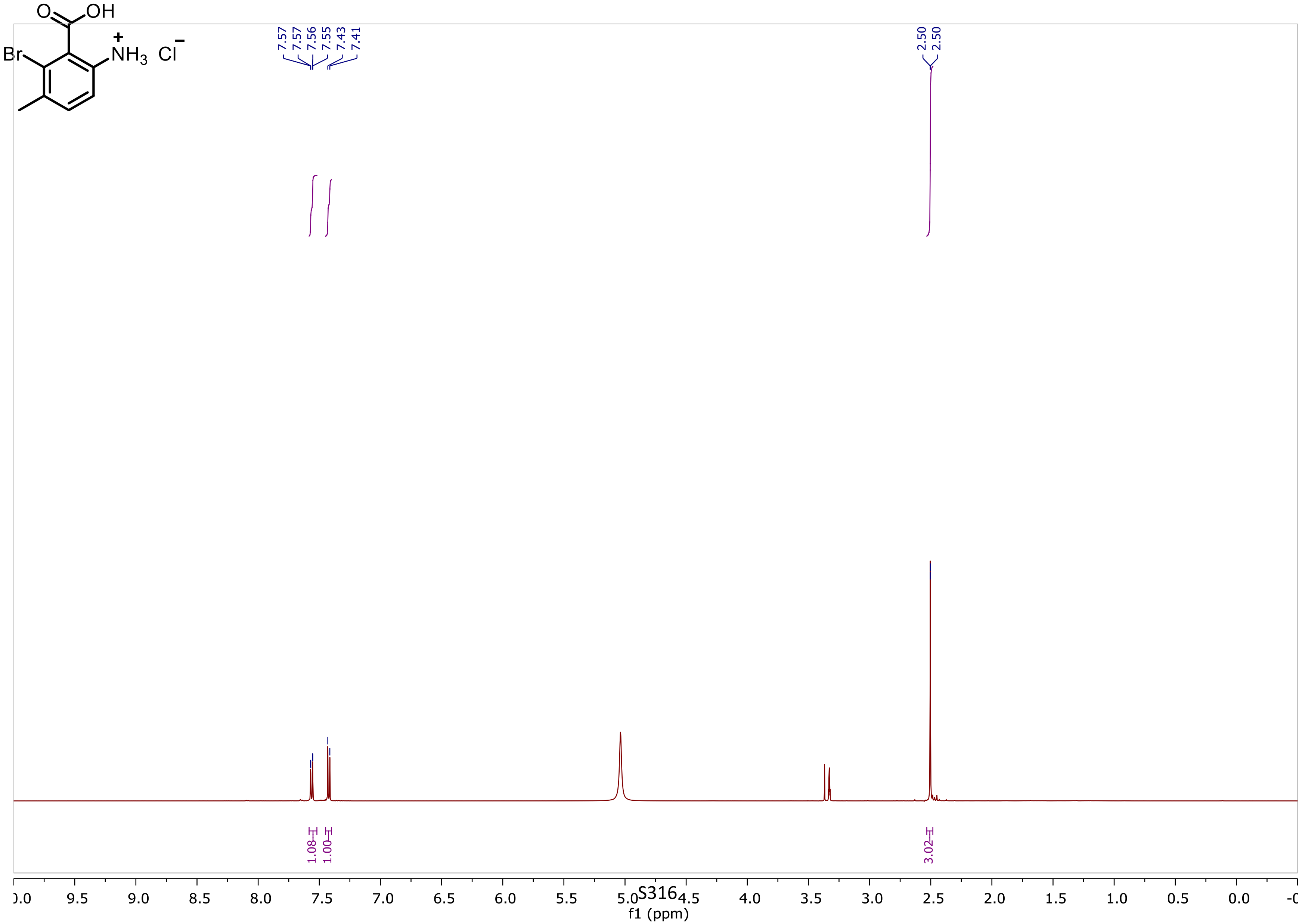

^{13}C NMR spectrum of 6-amino-2-bromo-3-methylbenzoic acid hydrochloride **2t** in MeOD-d_4

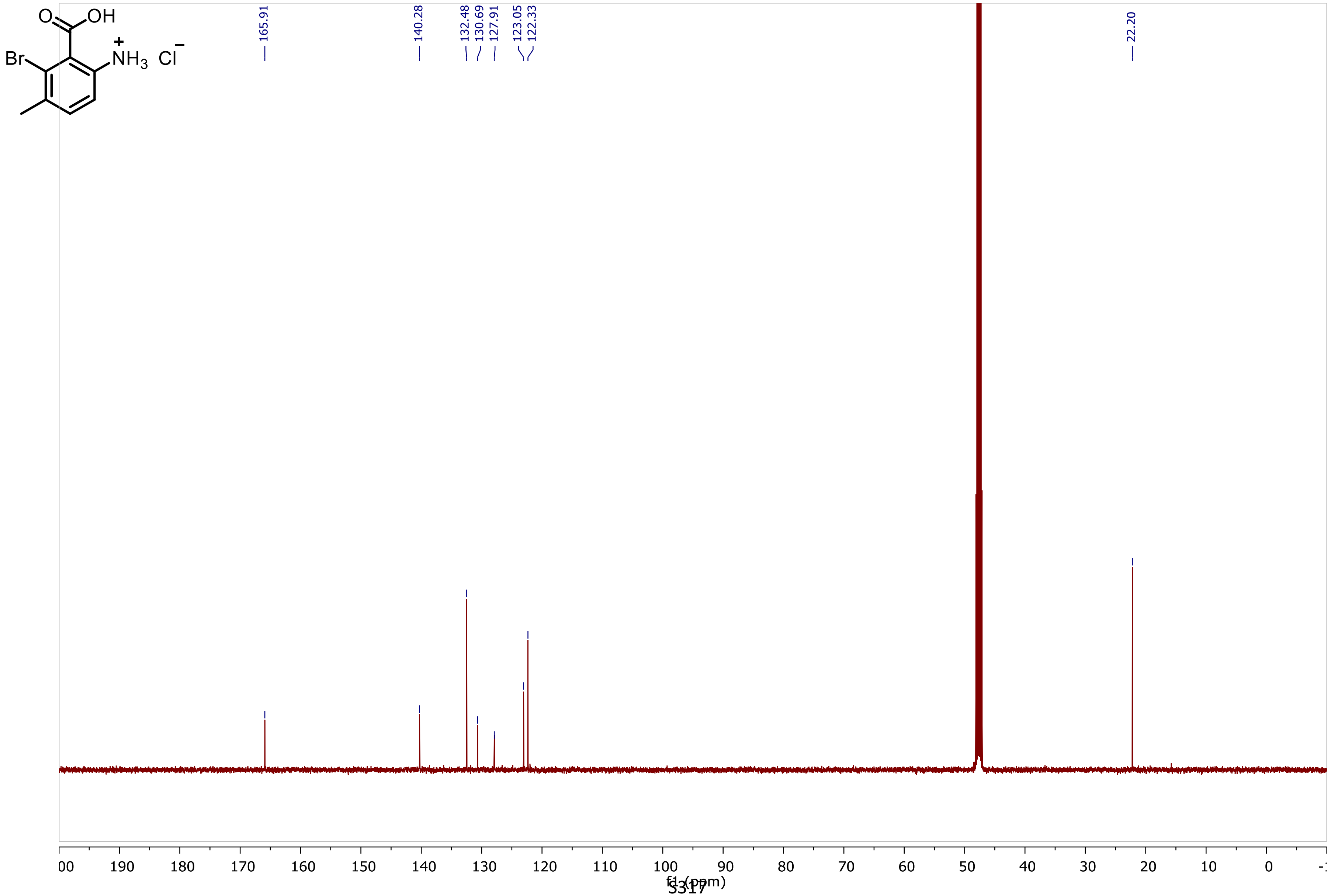

^{1}H NMR spectrum of 2-amino-5-bromobenzoic acid hydrochloride **2u (a)** and 2-amino-3-bromobenzoic acid hydrochloride ***iso*-2u (b)** in MeOD-d_4

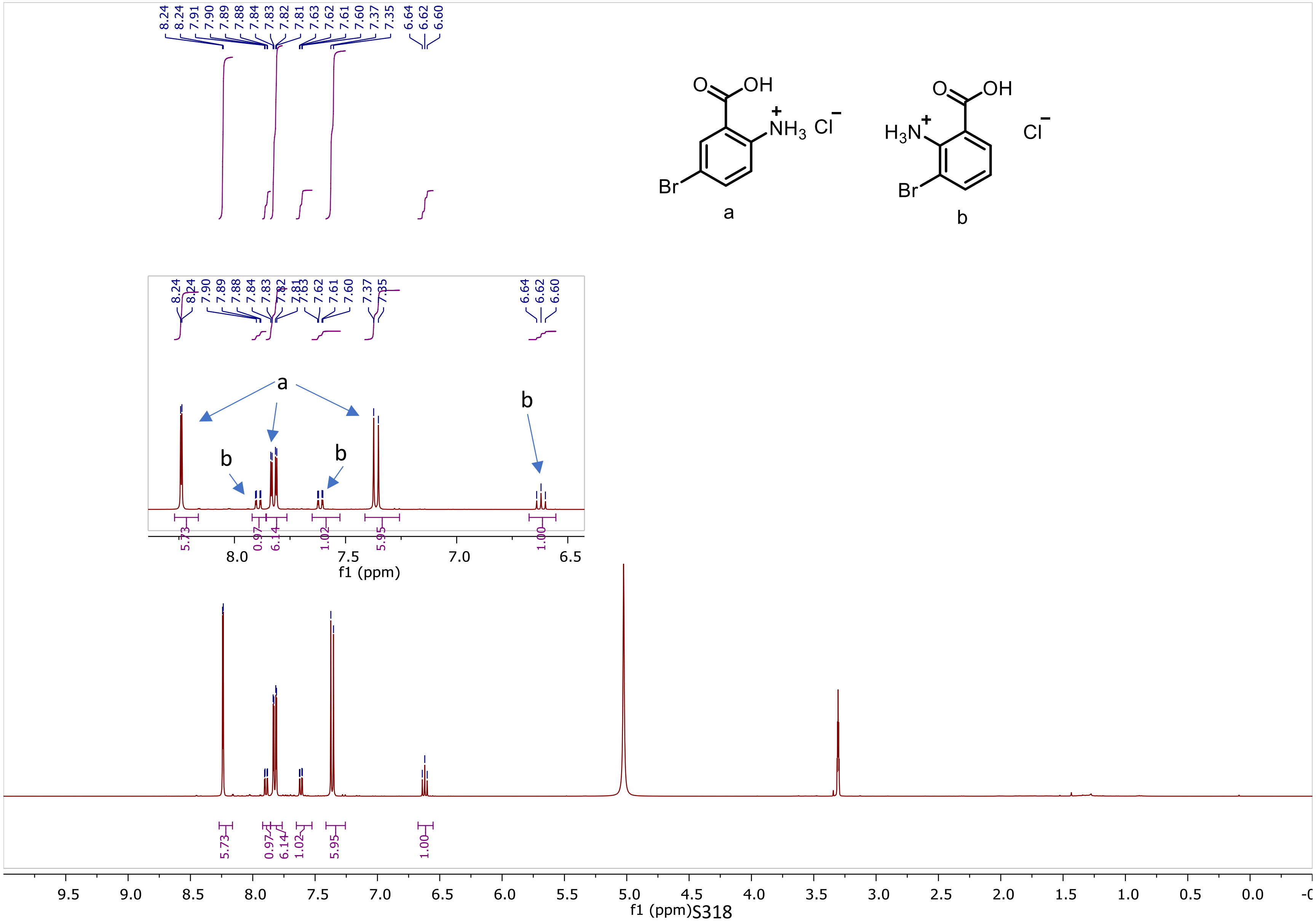

^{13}C NMR spectrum of 2-amino-5-bromobenzoic acid hydrochloride **2u** and 2-amino-3-bromobenzoic acid hydrochloride ***iso*-2u** in MeOD-d_4

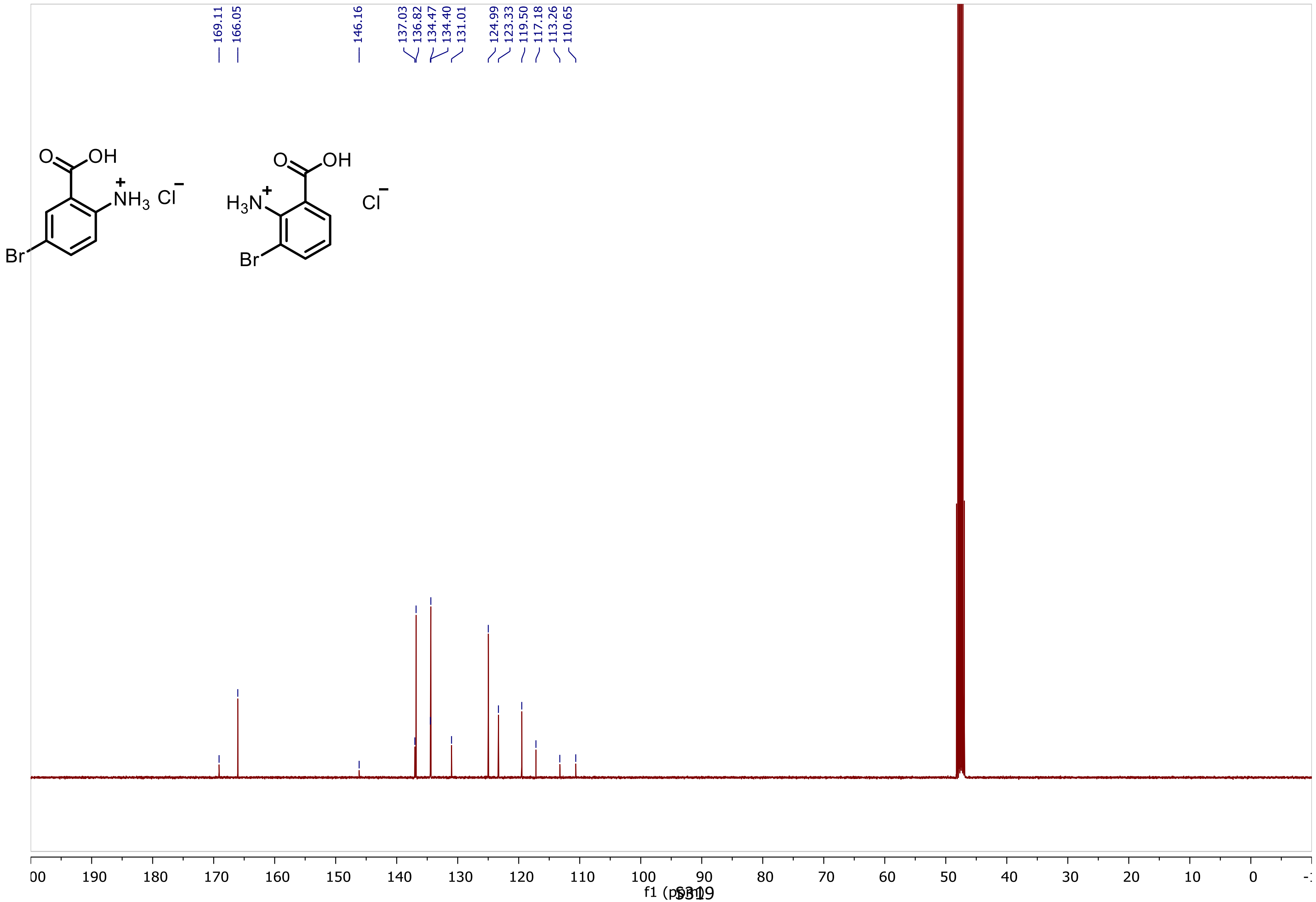

^{1}H NMR spectrum of 2-amino-5-iodobenzoic acid hydrochloride **2v (a)** and 2-amino-3-iodobenzoic acid hydrochloride ***iso*-2v (b)** in MeOD-d_4

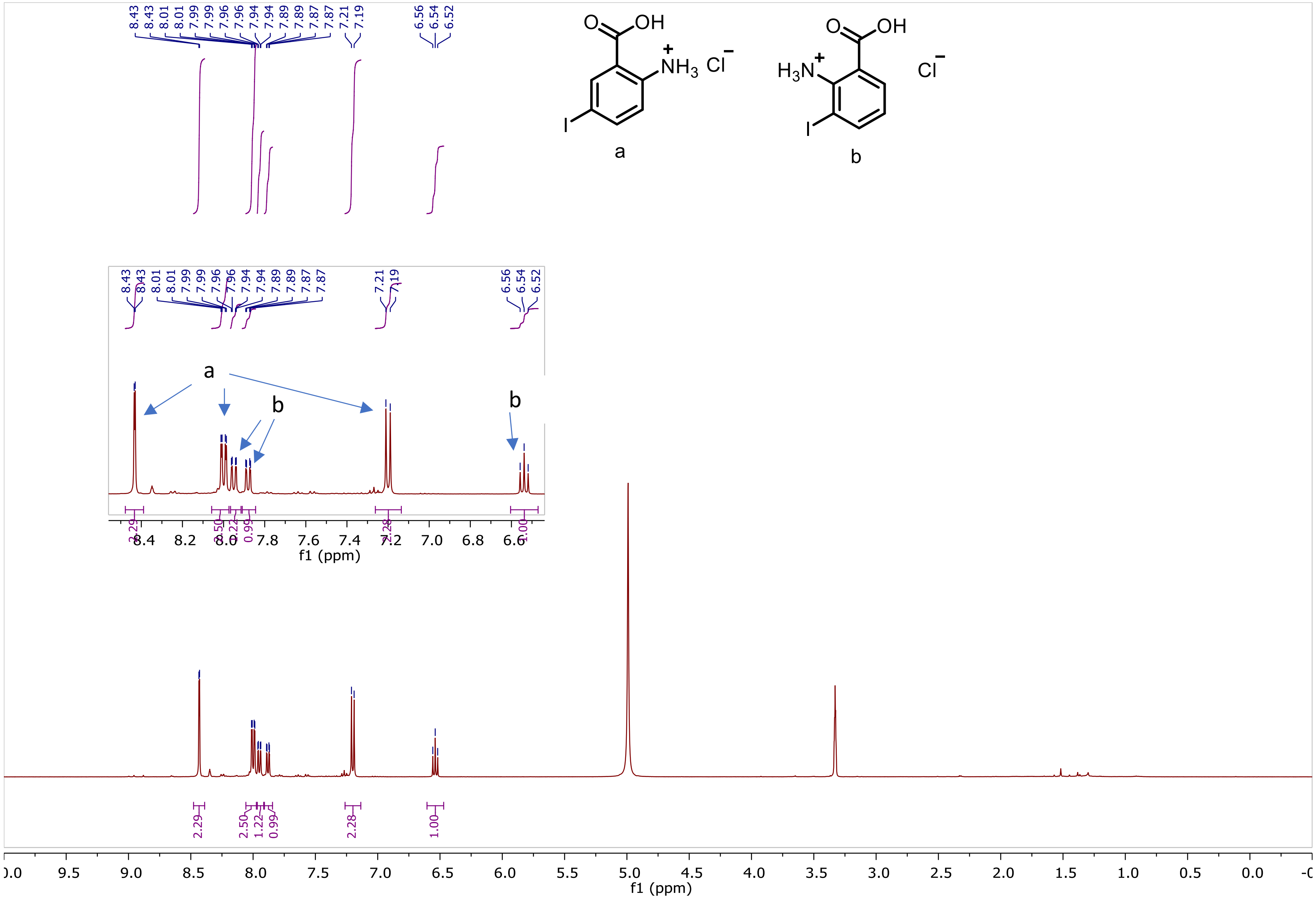

^{13}C NMR spectrum of 2-amino-5-iodobenzoic acid hydrochloride **2v** and 2-amino-3-iodobenzoic acid hydrochloride ***iso*-2v** in MeOD-d_4

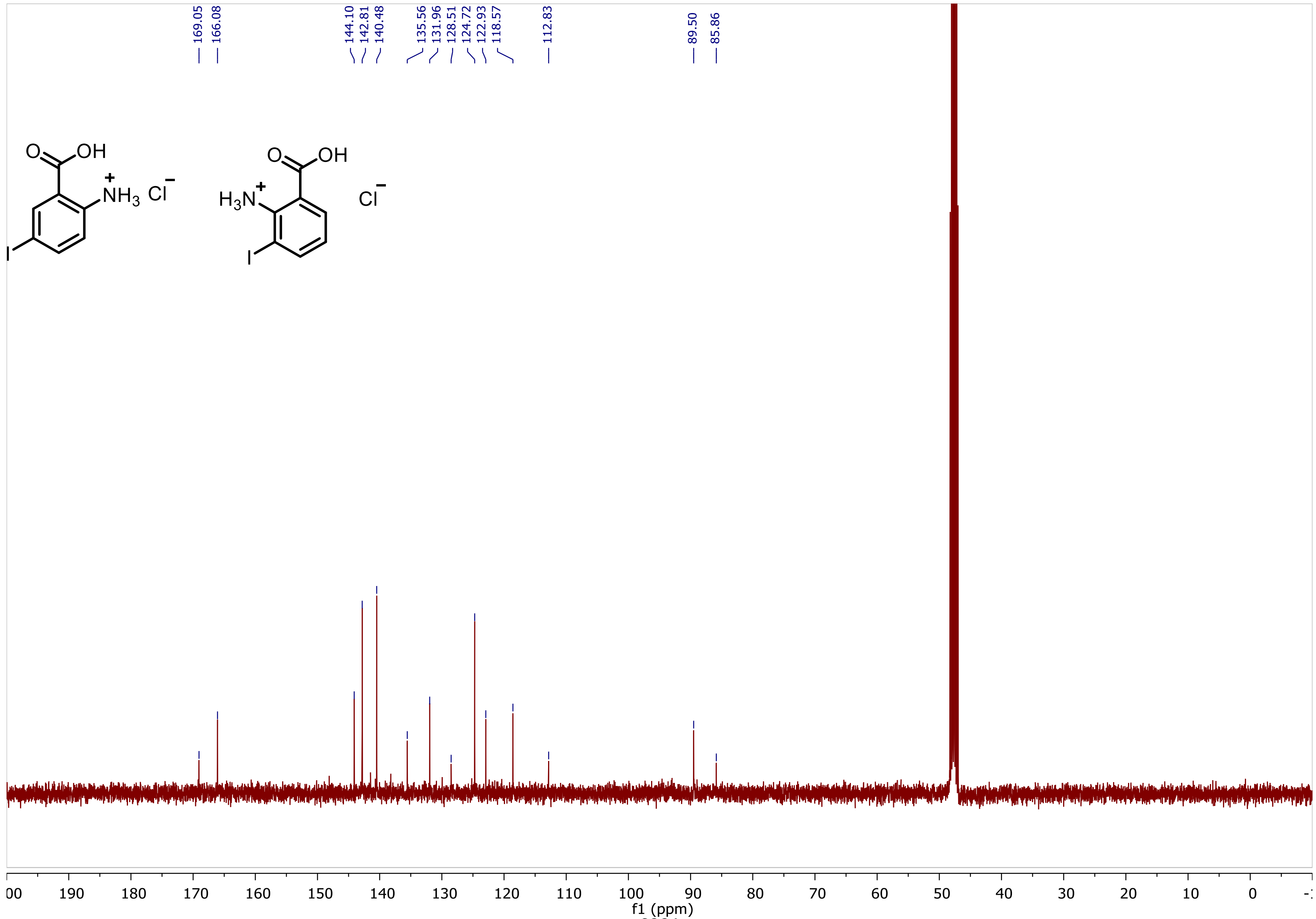

^{1}H NMR spectrum of 6-amino-2,3-dichlorobenzoic acid hydrochloride **2w** in MeOD-d_4

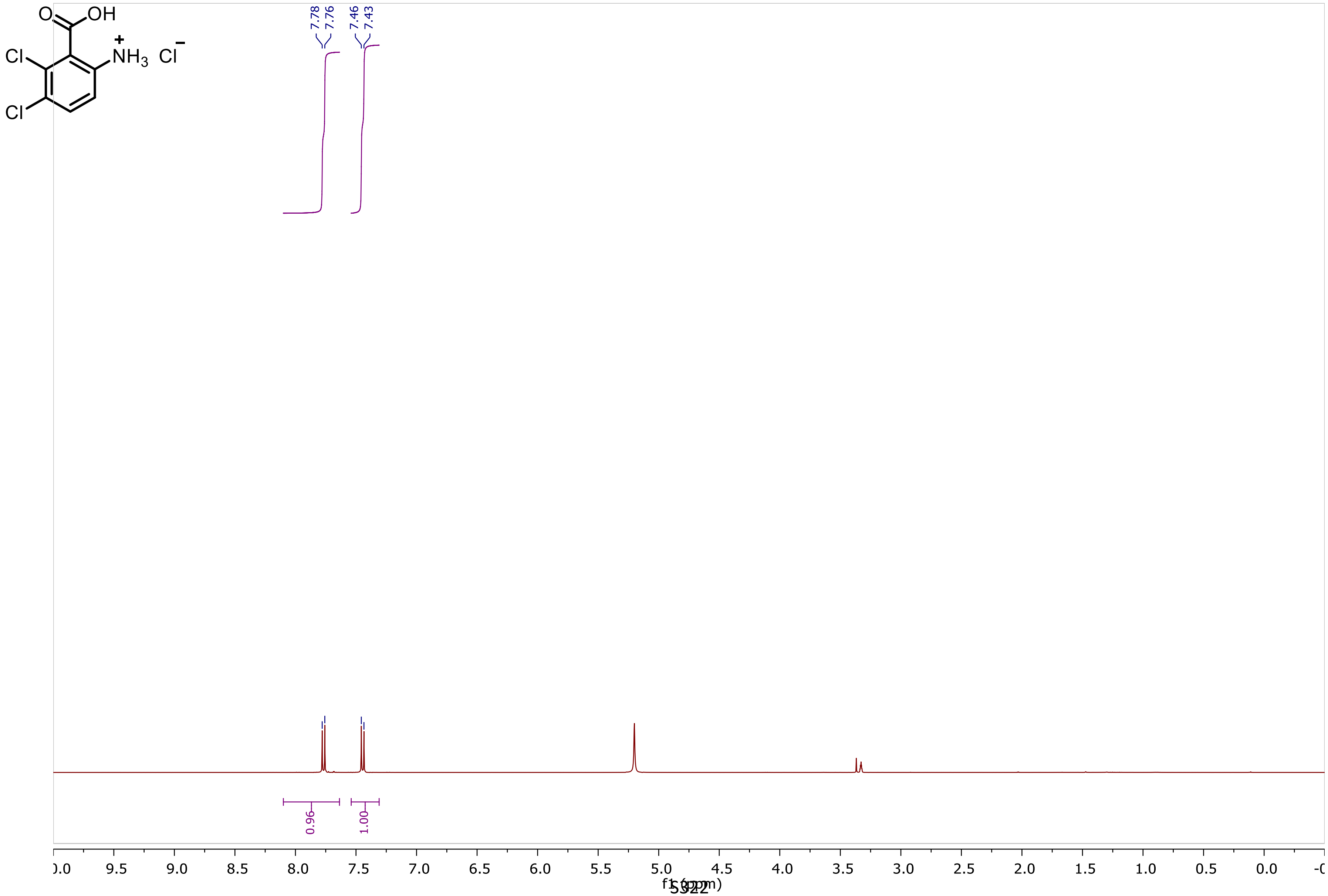

^{13}C NMR spectrum of 6-amino-2,3-dichlorobenzoic acid hydrochloride **2w** in MeOD-d_4

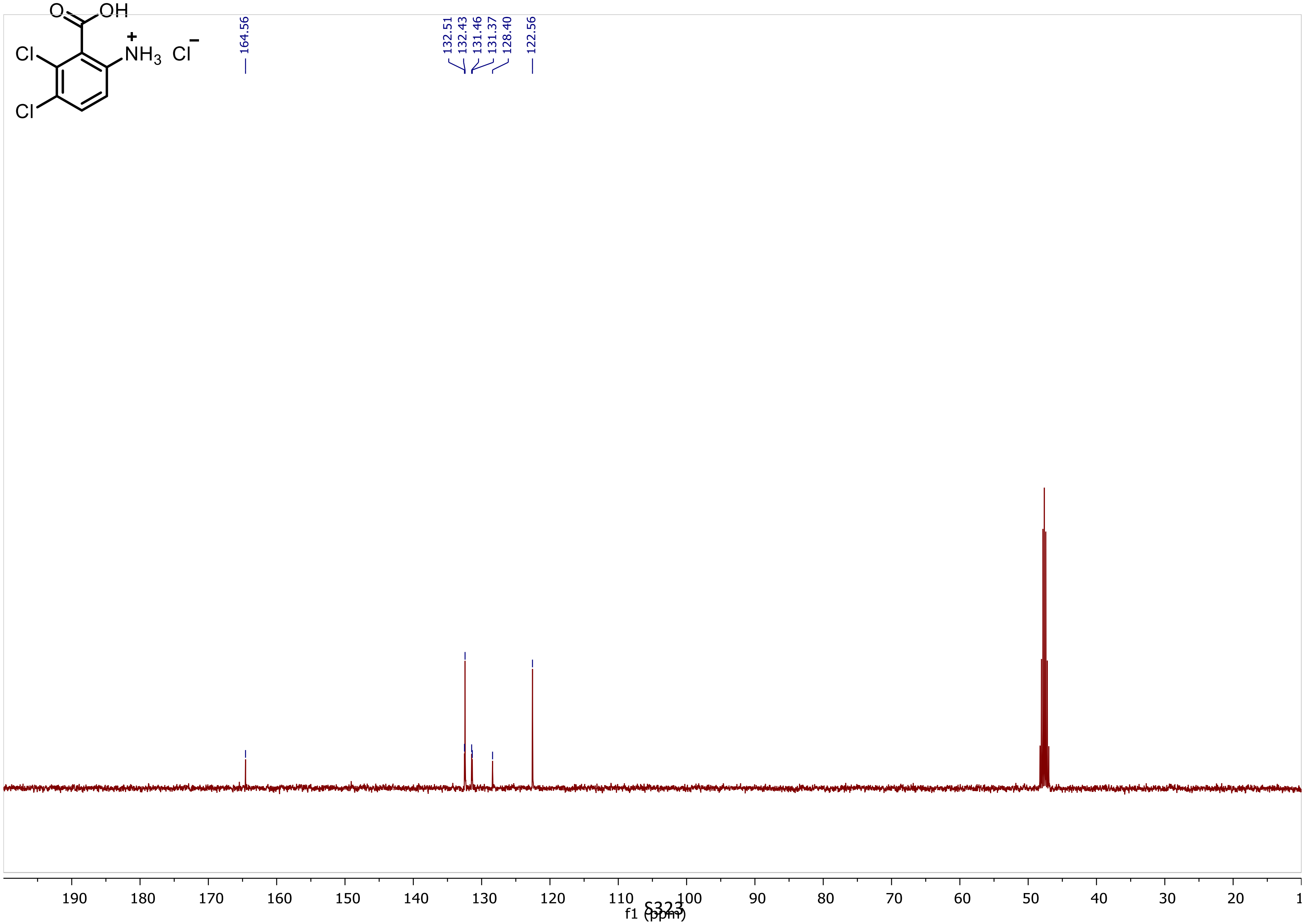

^{1}H NMR spectrum of 5,6-dichloroquinazolin-4(3*H*)-one **S2w** in DMSO-d_6

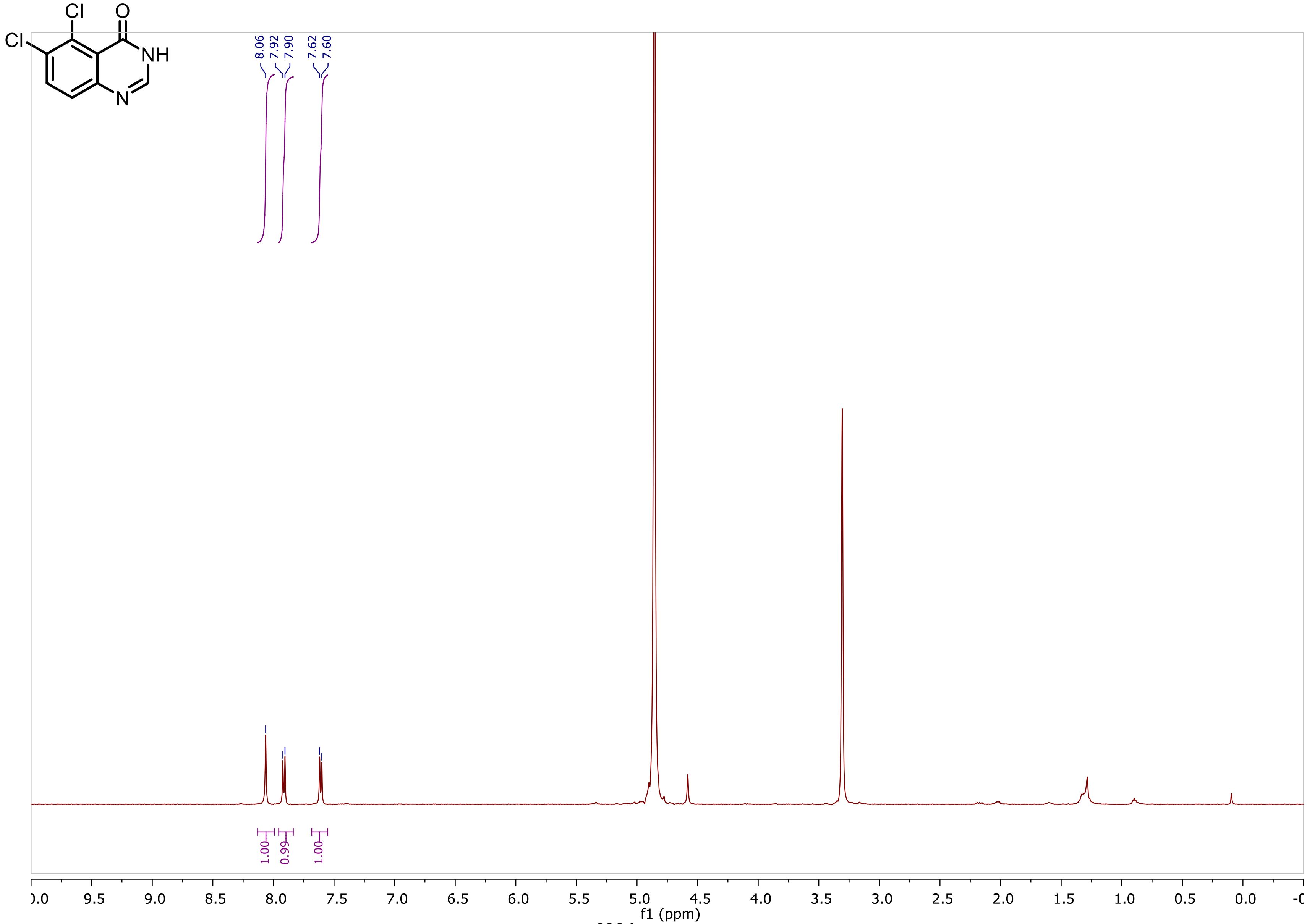

^{13}C NMR spectrum of 5,6-dichloroquinazolin-4(3*H*)-one **S2w** in DMSO-d_6

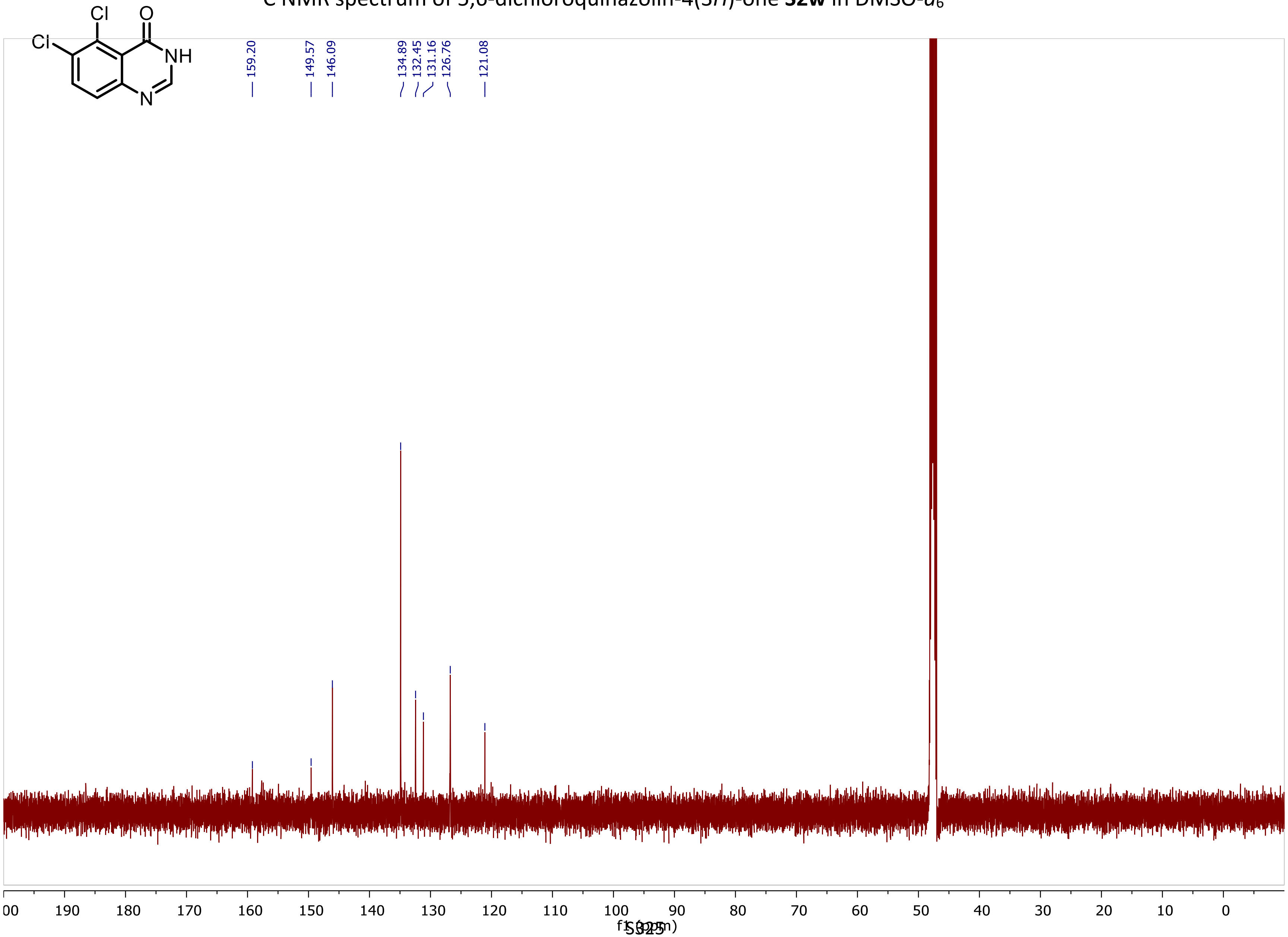

^{1}H NMR spectrum of 2-amino-3,5-dibromobenzoic acid hydrochloride **2x** in MeOD-d_4

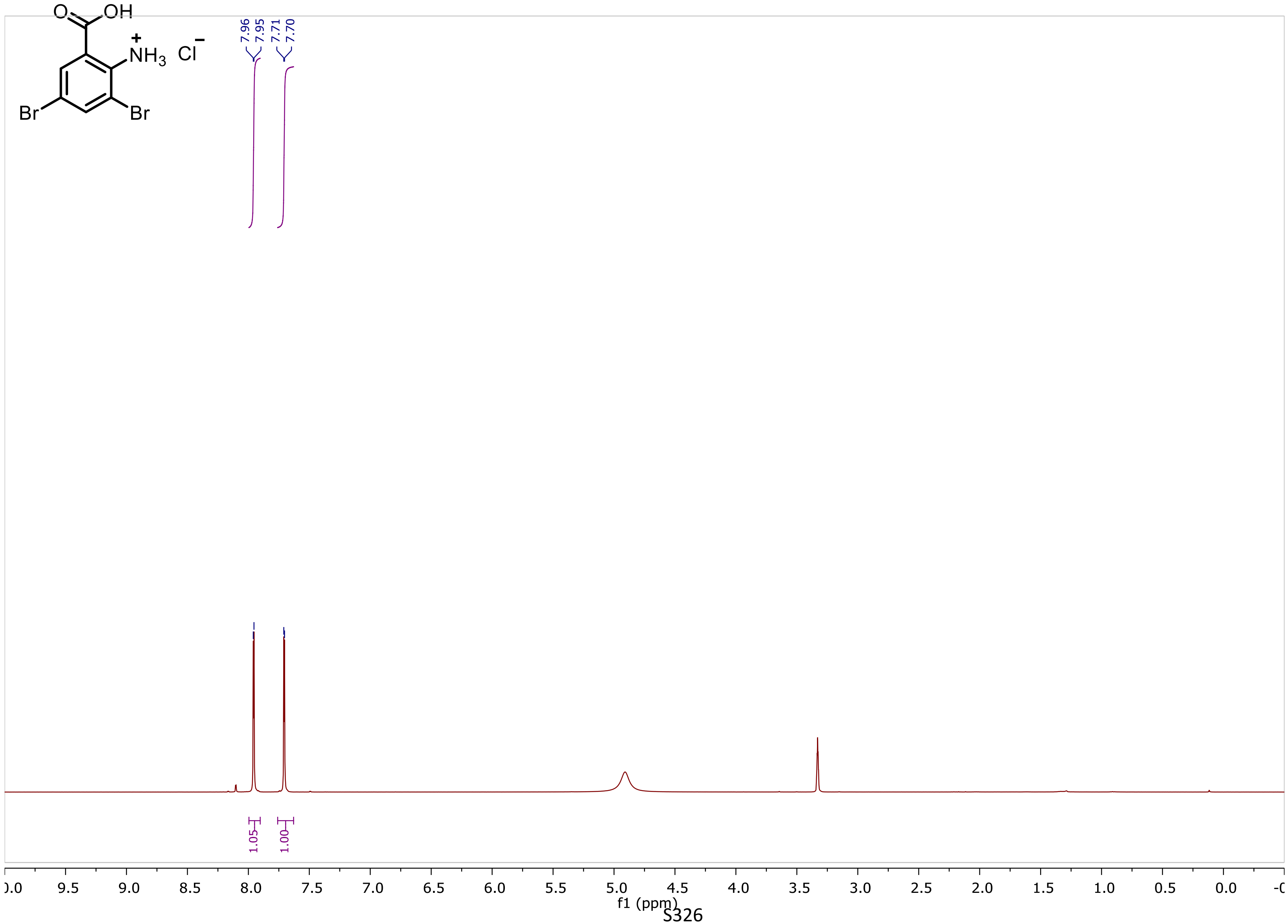

^{13}C NMR spectrum of 2-amino-3,5-dibromobenzoic acid hydrochloride **2x** in MeOD-d_4

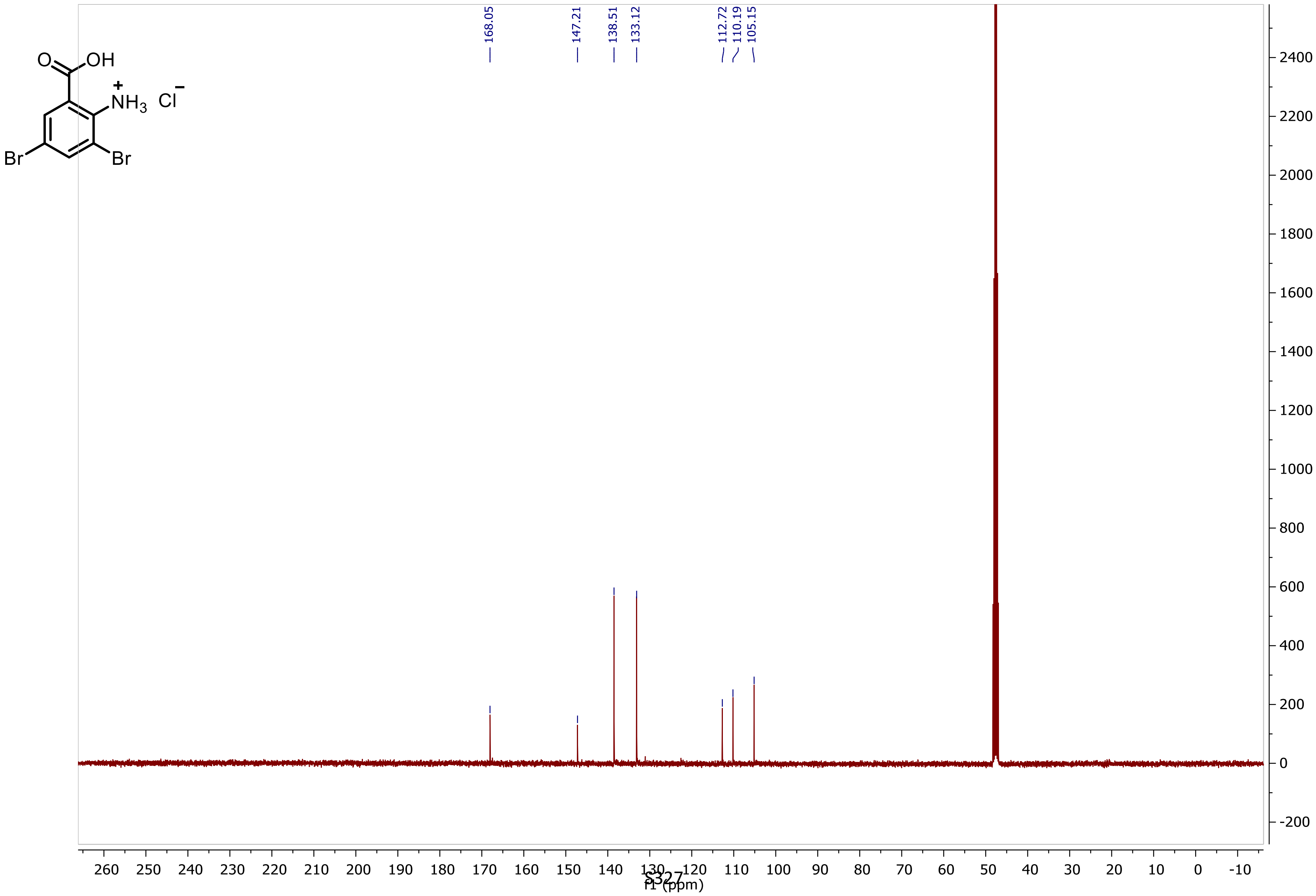

^{1}H NMR spectrum of 2-amino-3,5-difluorobenzoic acid hydrochloride **2y** in MeOD-d_4

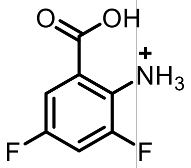

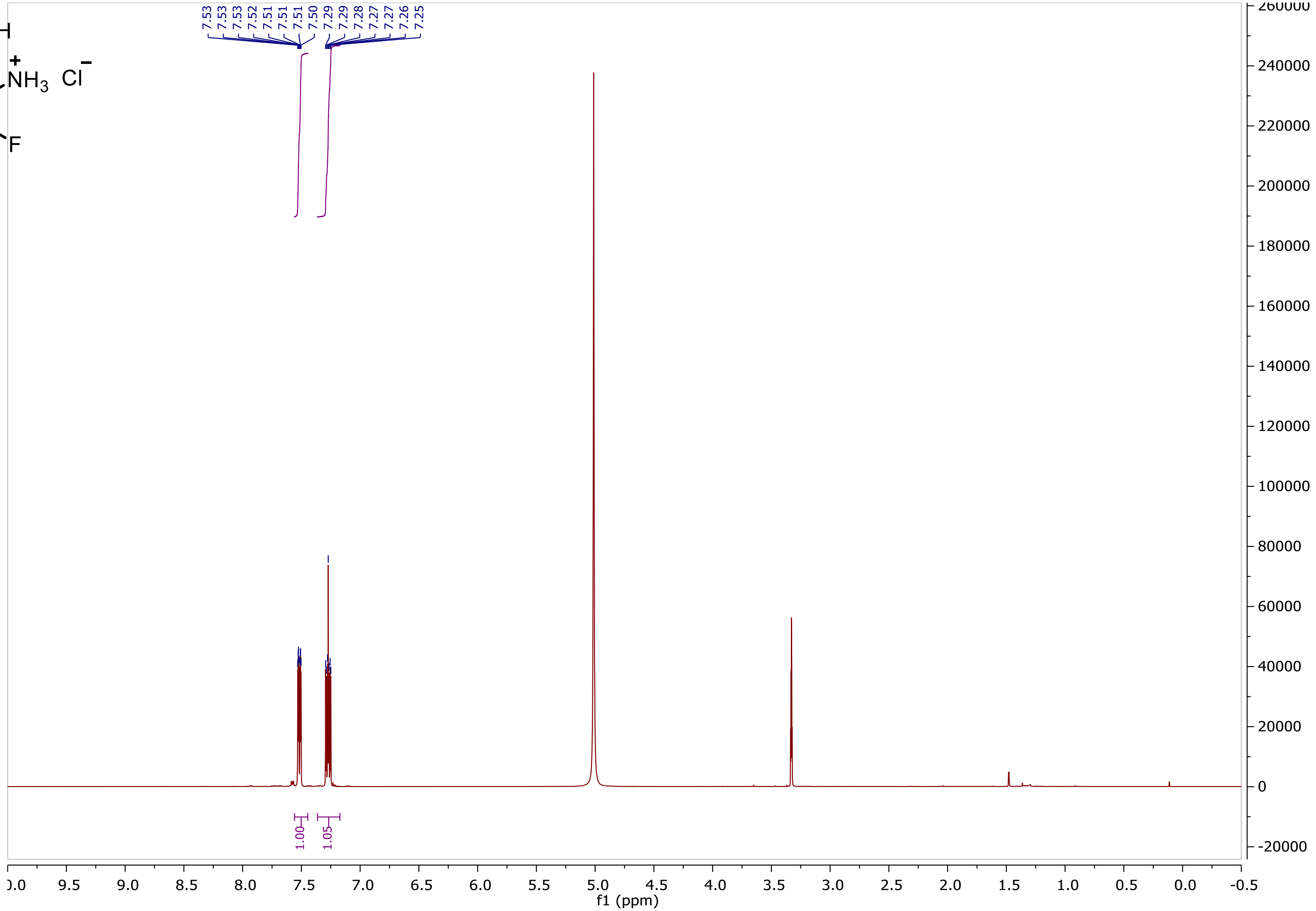

^{13}C NMR spectrum of 2-amino-3,5-difluorobenzoic acid hydrochloride **2y** in MeOD-d_4

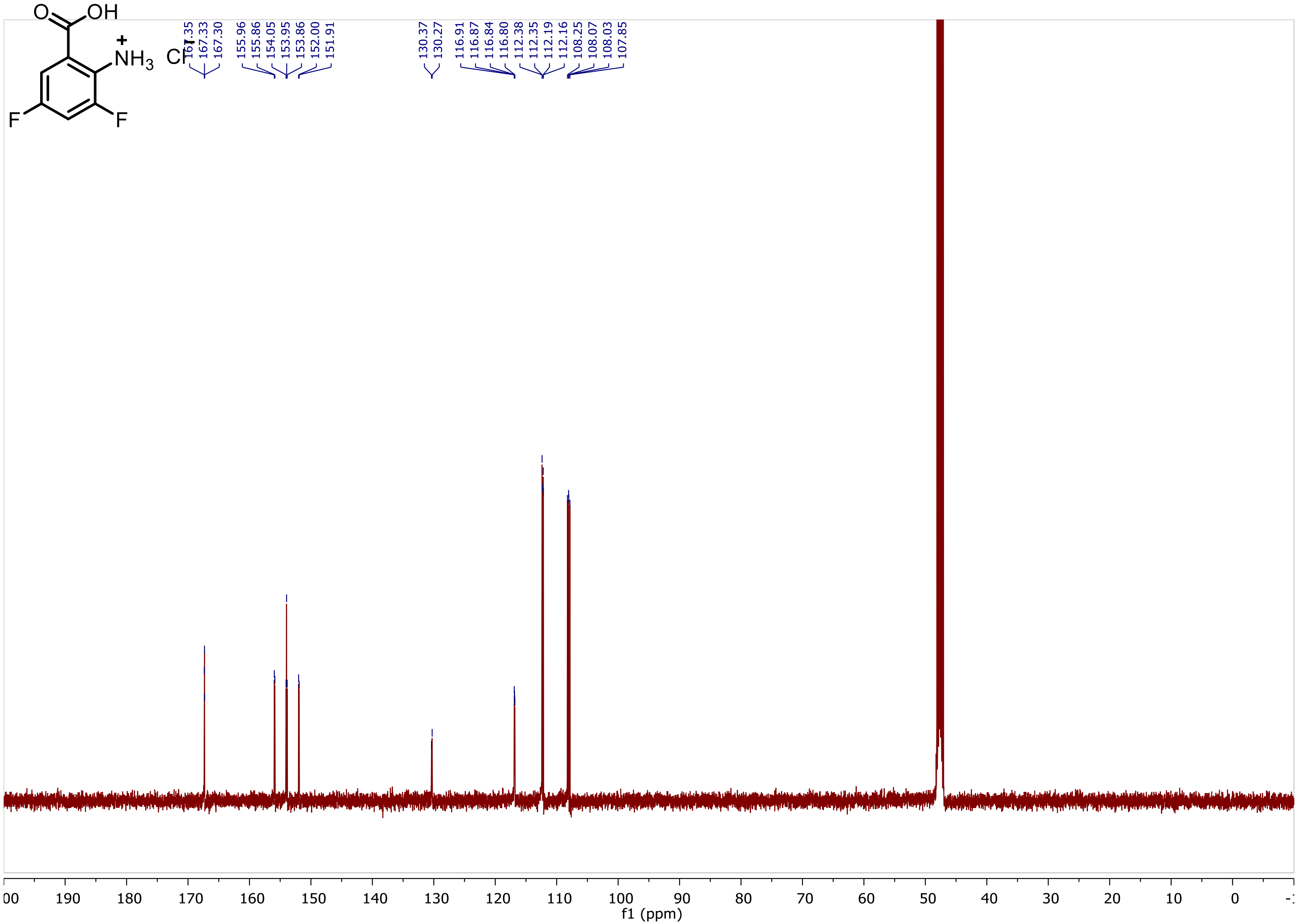

^{19}F NMR spectrum of 2-amino-3,5-difluorobenzoic acid hydrochloride **2y** in MeOD-d_4

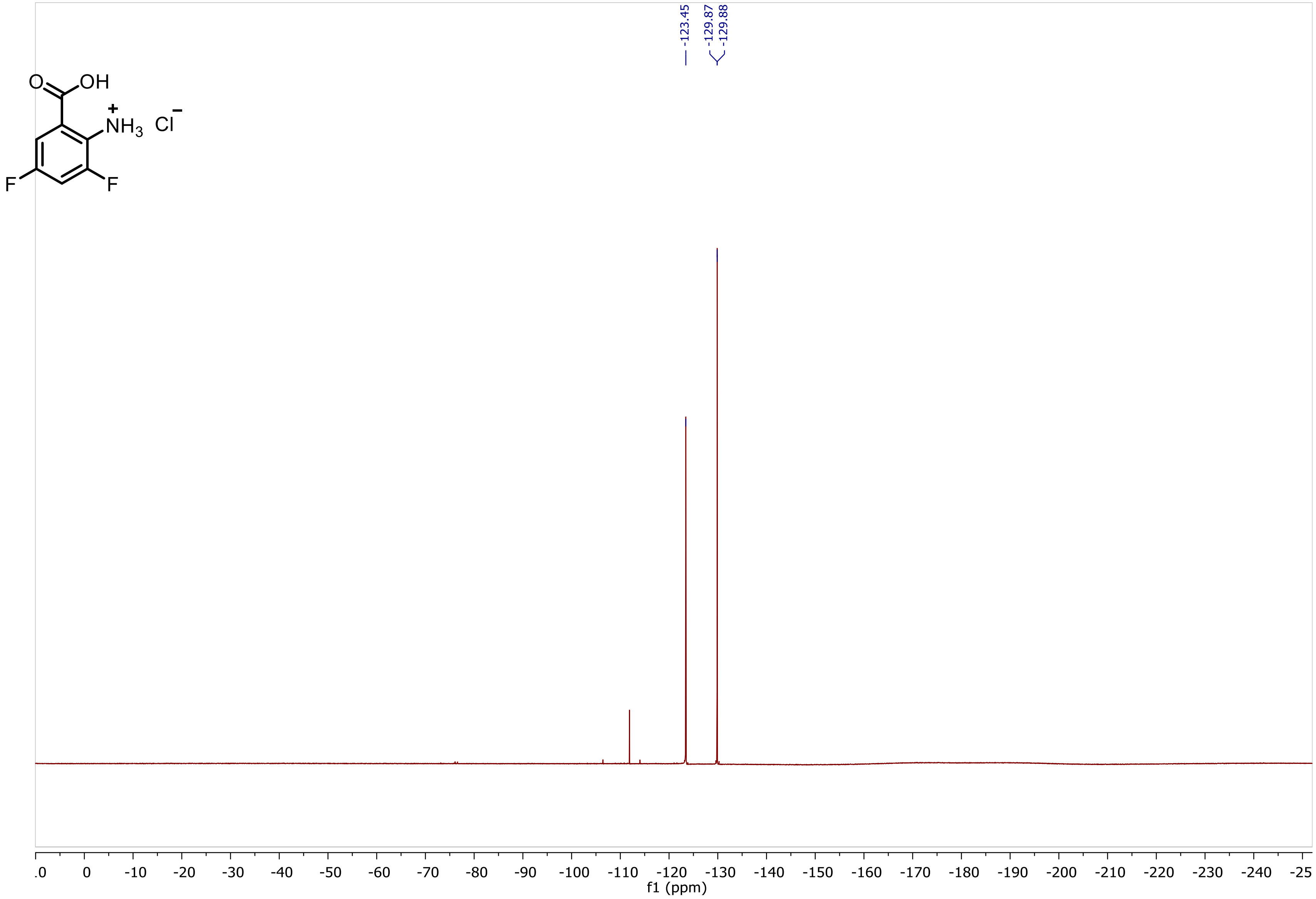

^{1}H NMR spectrum of 2-amino-5-methoxybenzoic acid hydrochloride **2z (a)** and 2-amino-3-methoxybenzoic acid ***iso*-2z (b)** in DMSO-d_6

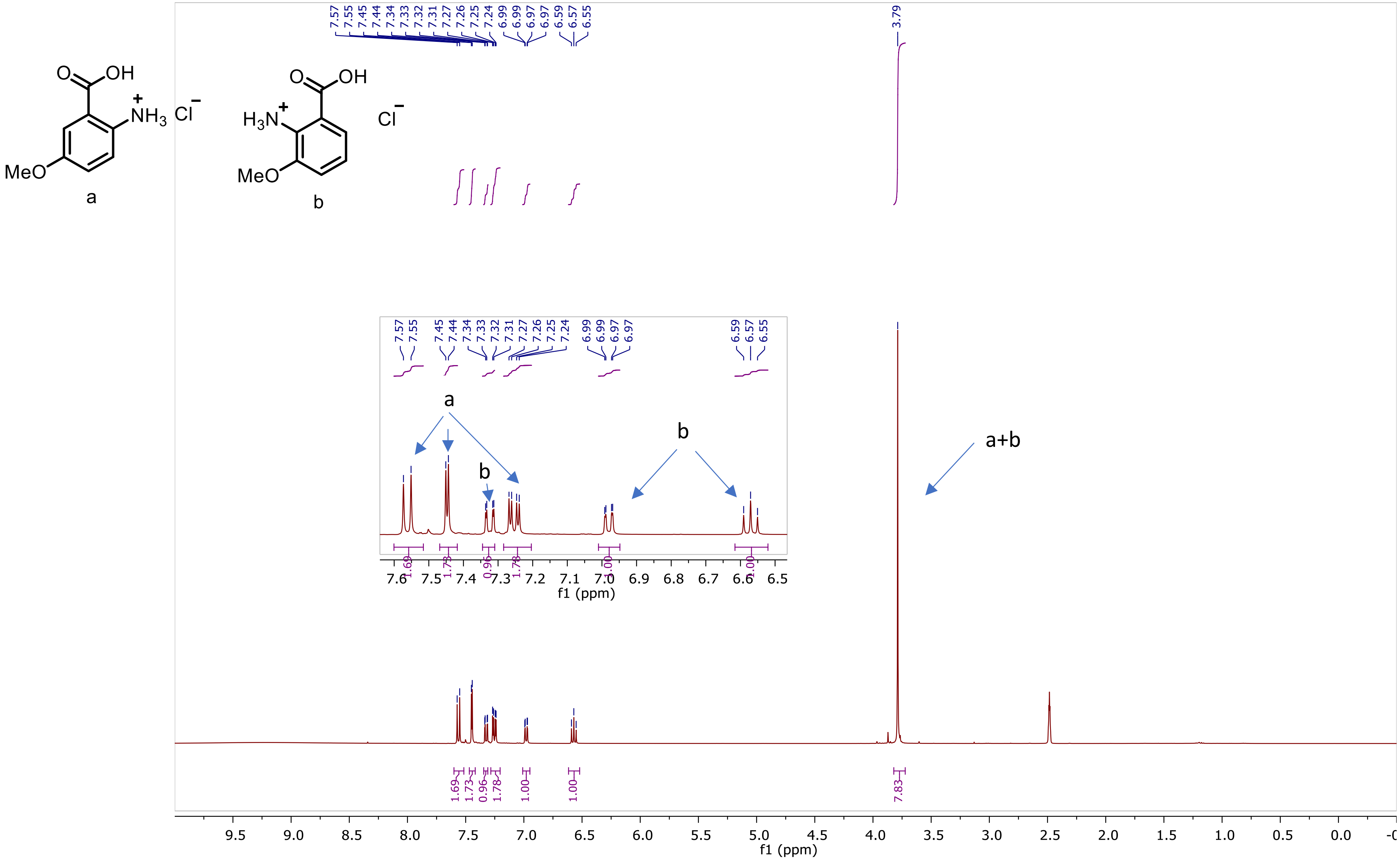

^{13}C NMR spectrum of 2-amino-5-methoxybenzoic acid hydrochloride **2z** and 2-amino-3-methoxybenzoic acid ***iso*-2z** in DMSO-d_6

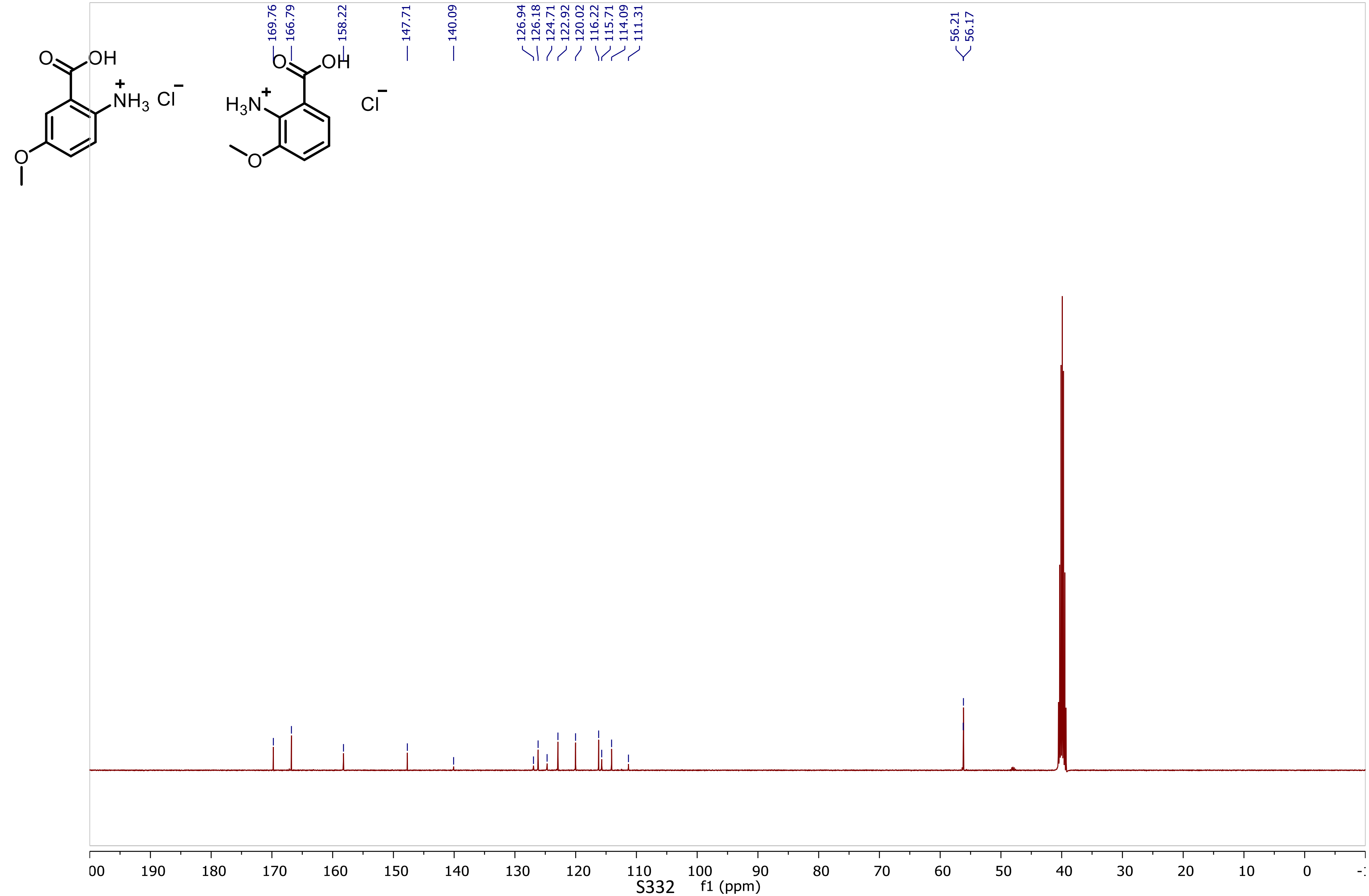

^{1}H NMR spectrum of 6-amino-3-methoxy-2-methylbenzoic acid hydrochloride **2aa** in MeOD-d_4

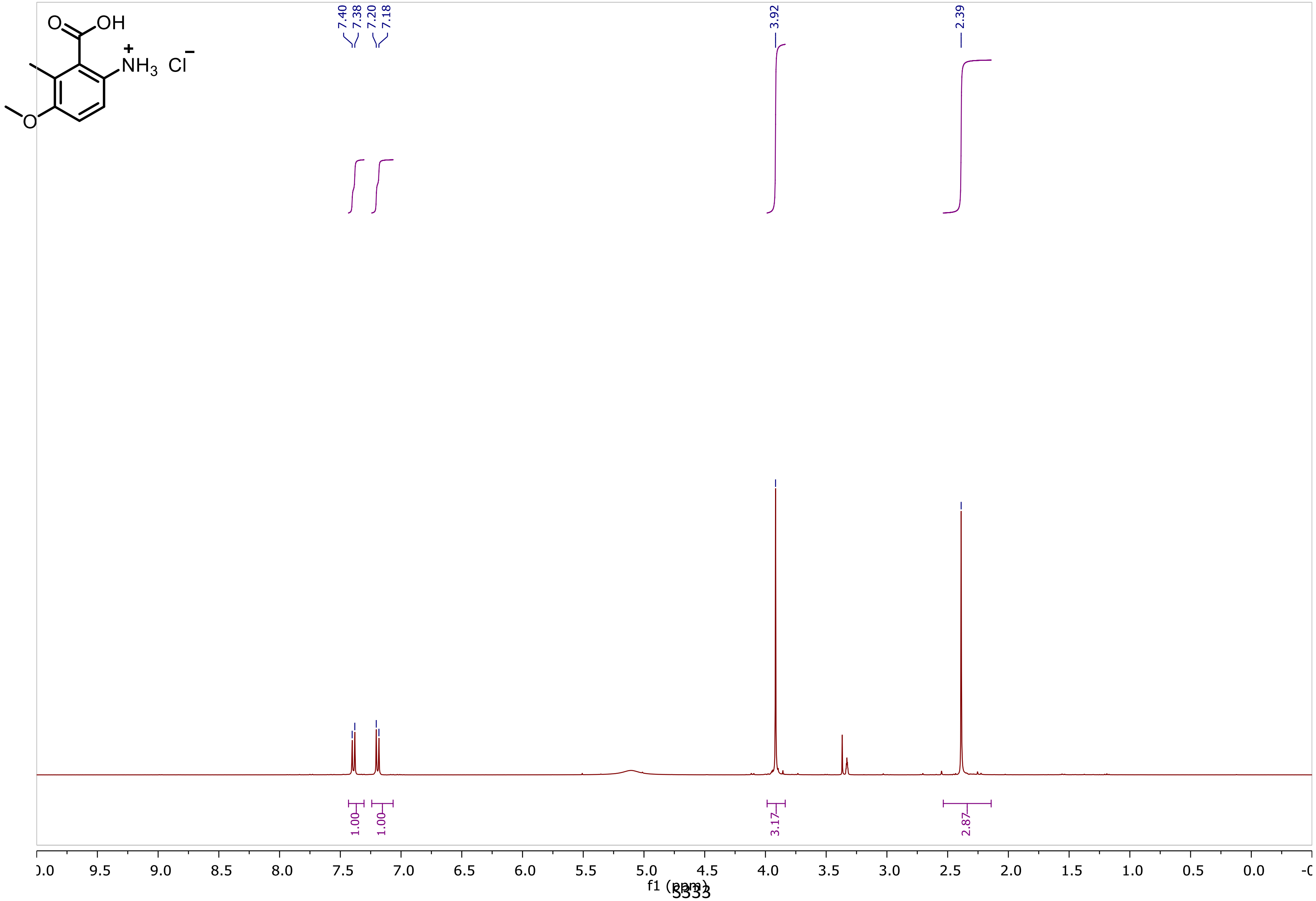

^{13}C NMR spectrum of 6-amino-3-methoxy-2-methylbenzoic acid hydrochloride **2aa** in MeOD-d_4

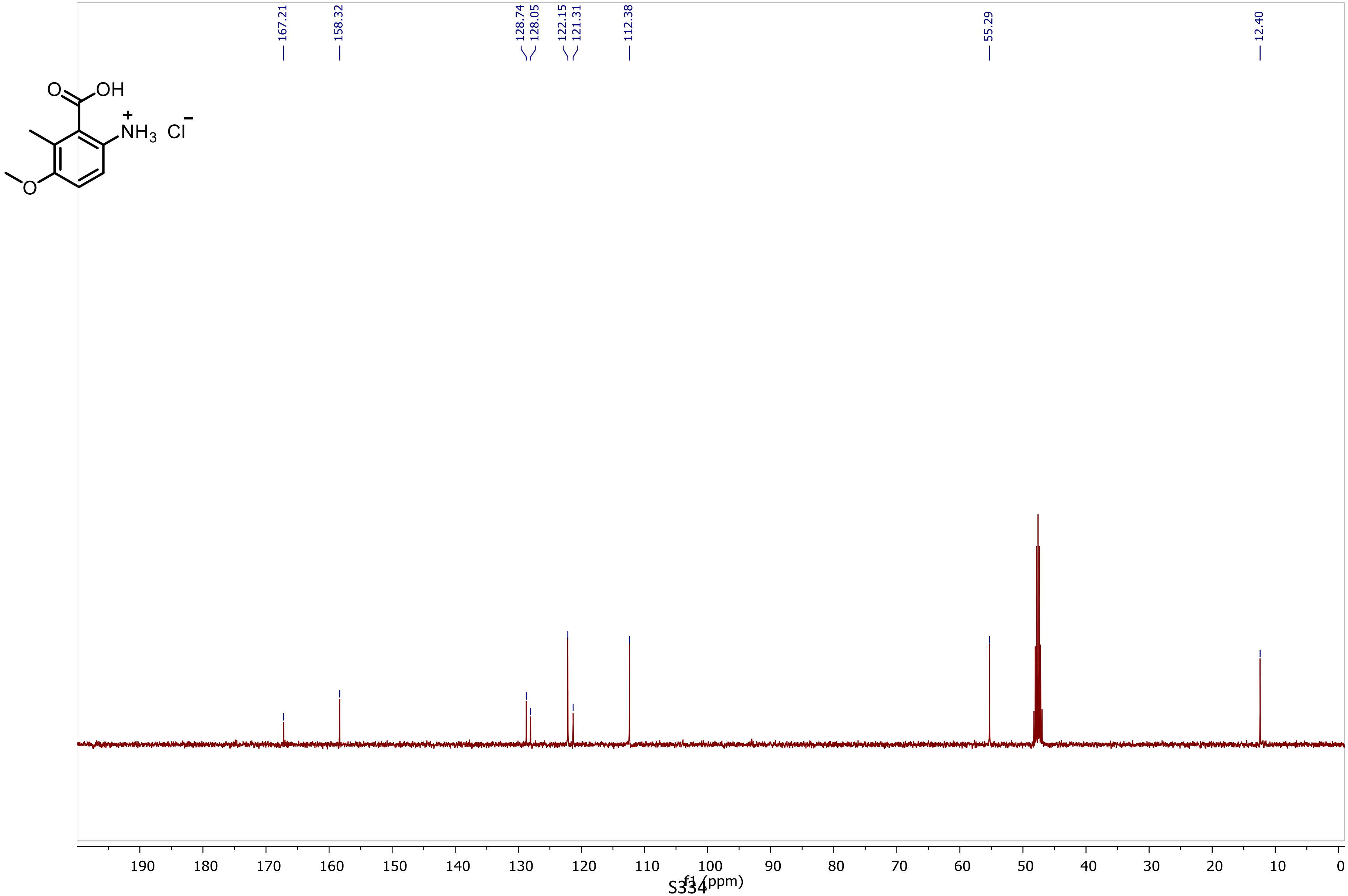

^{1}H NMR spectrum of 6-methoxy-5-methylquinazolin-4(3*H*)-one **S2aa** in MeOD-d_4

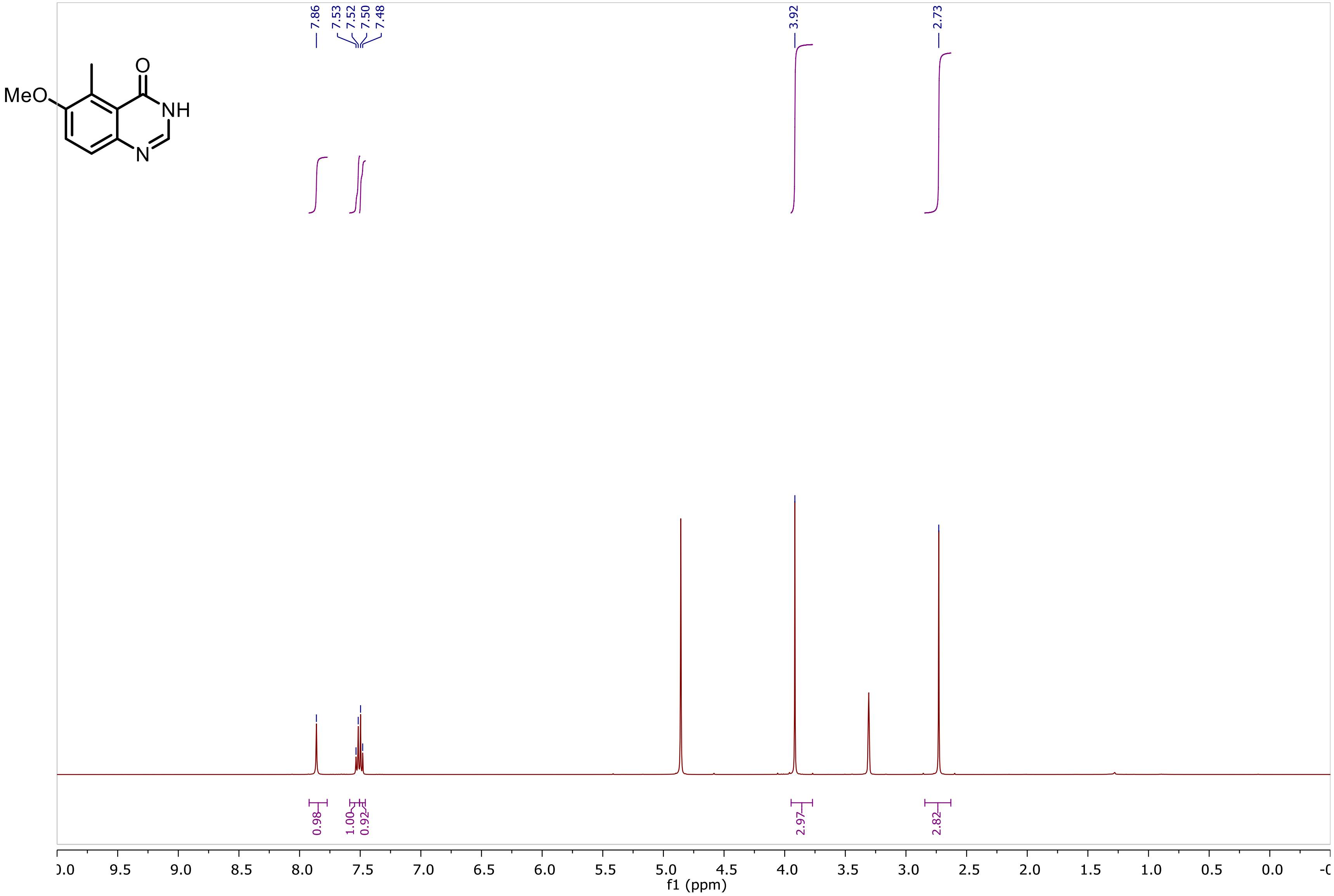

^{13}C NMR spectrum of 6-methoxy-5-methylquinazolin-4(3*H*)-one **S2aa** in MeOD-d_4

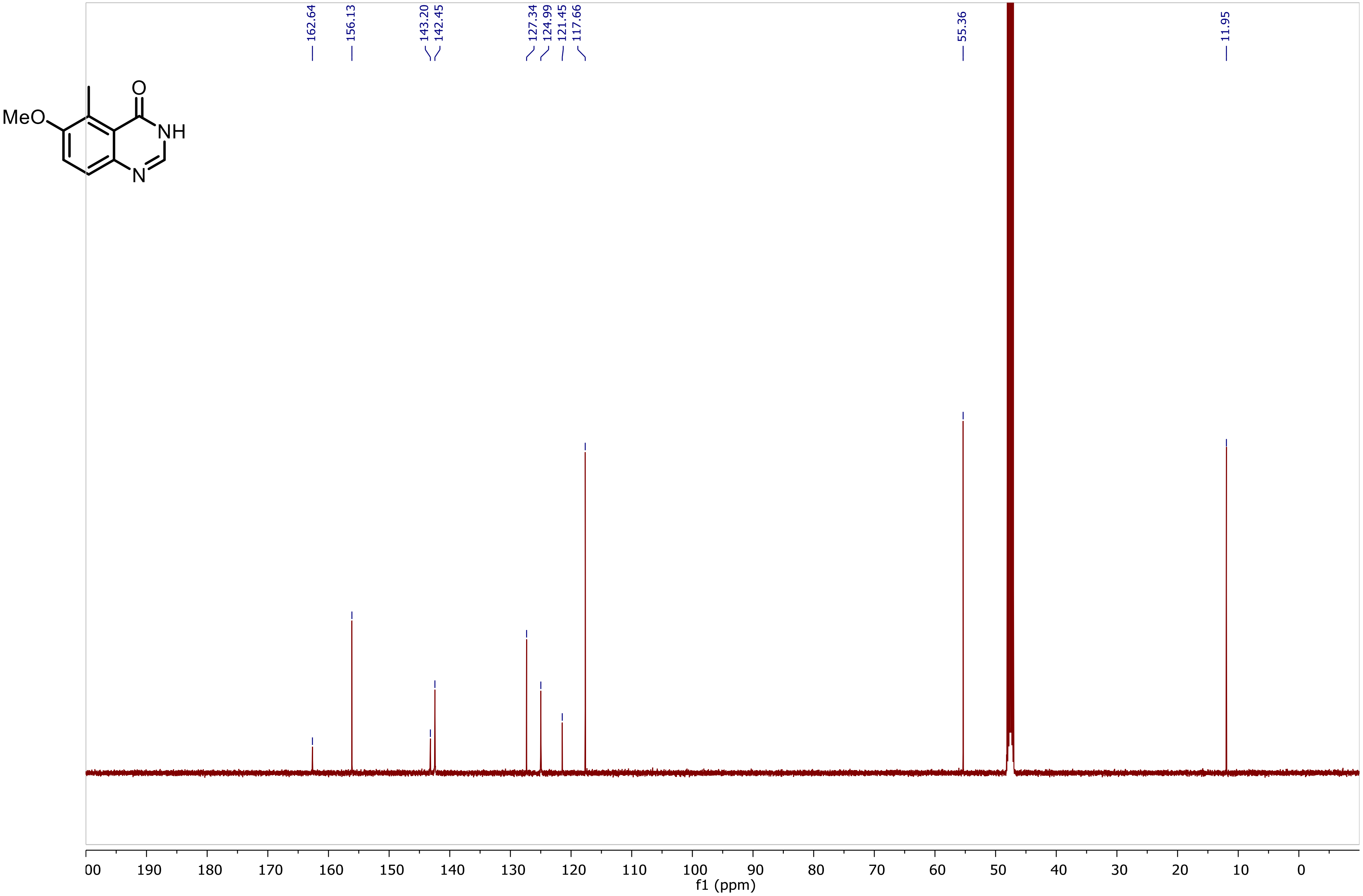

^{1}H NMR spectrum of 6-amino-2-bromo-3-methoxybenzoic acid hydrochloride **2ab** in MeOD-d_4

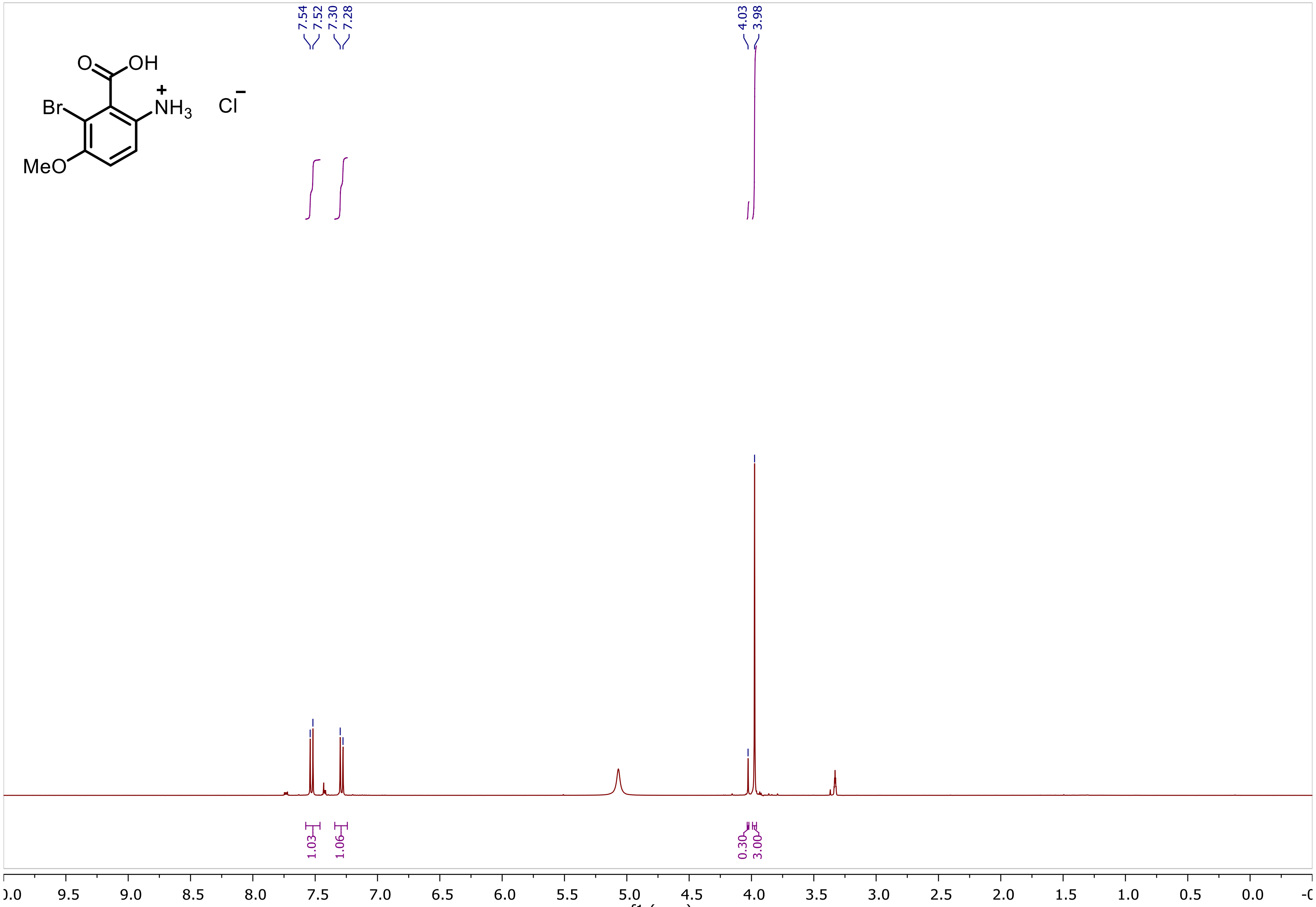

^{13}C NMR spectrum of 6-amino-2-bromo-3-methoxybenzoic acid hydrochloride **2ab** in MeOD-d_4

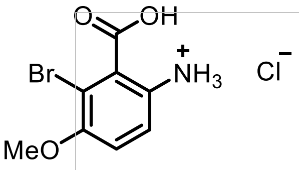

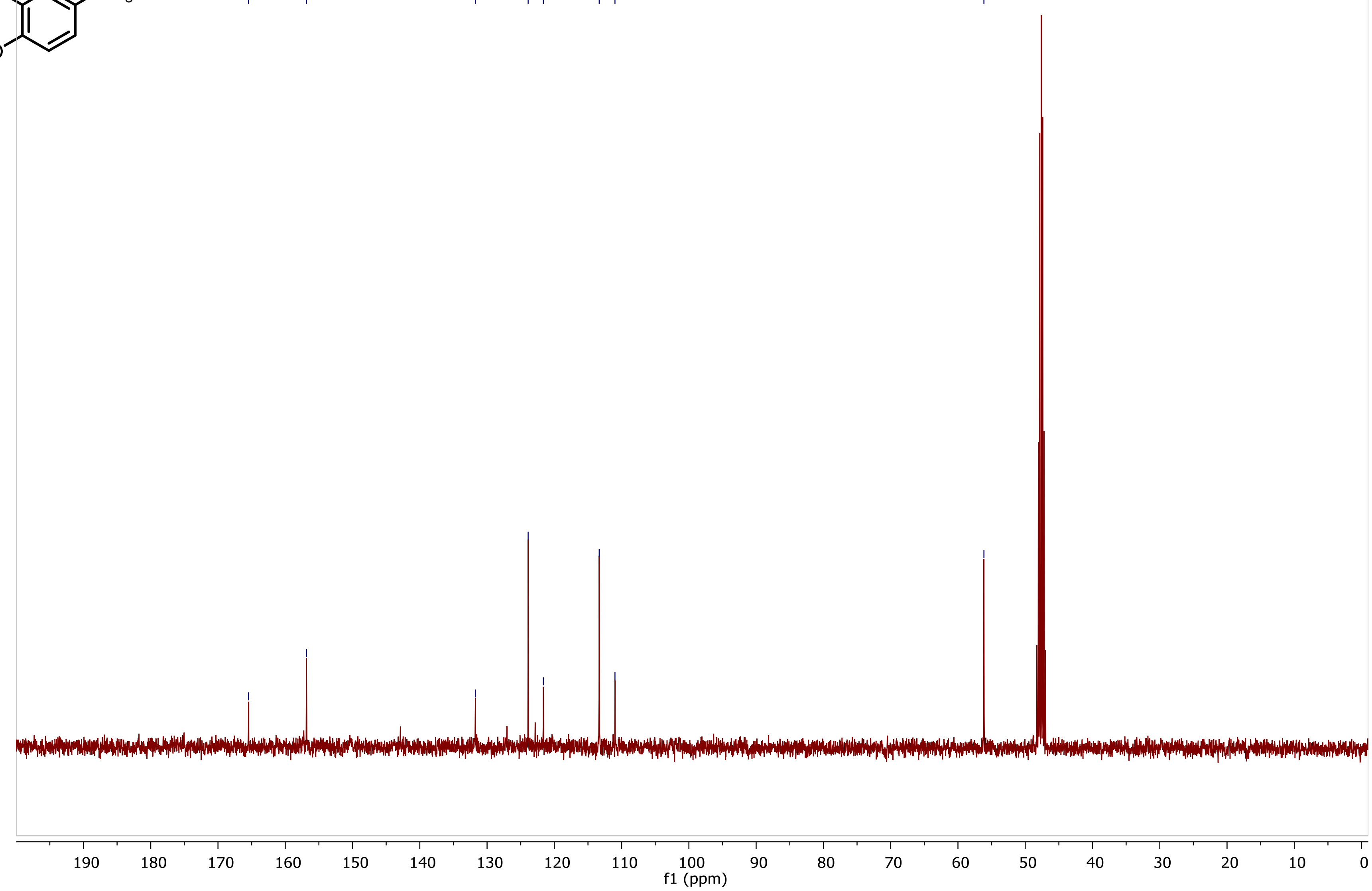

^{1}H NMR spectrum of 5-bromo-6-methoxyquinazolin-4(3*H*)-one **S2ab** in DMSO-d_6

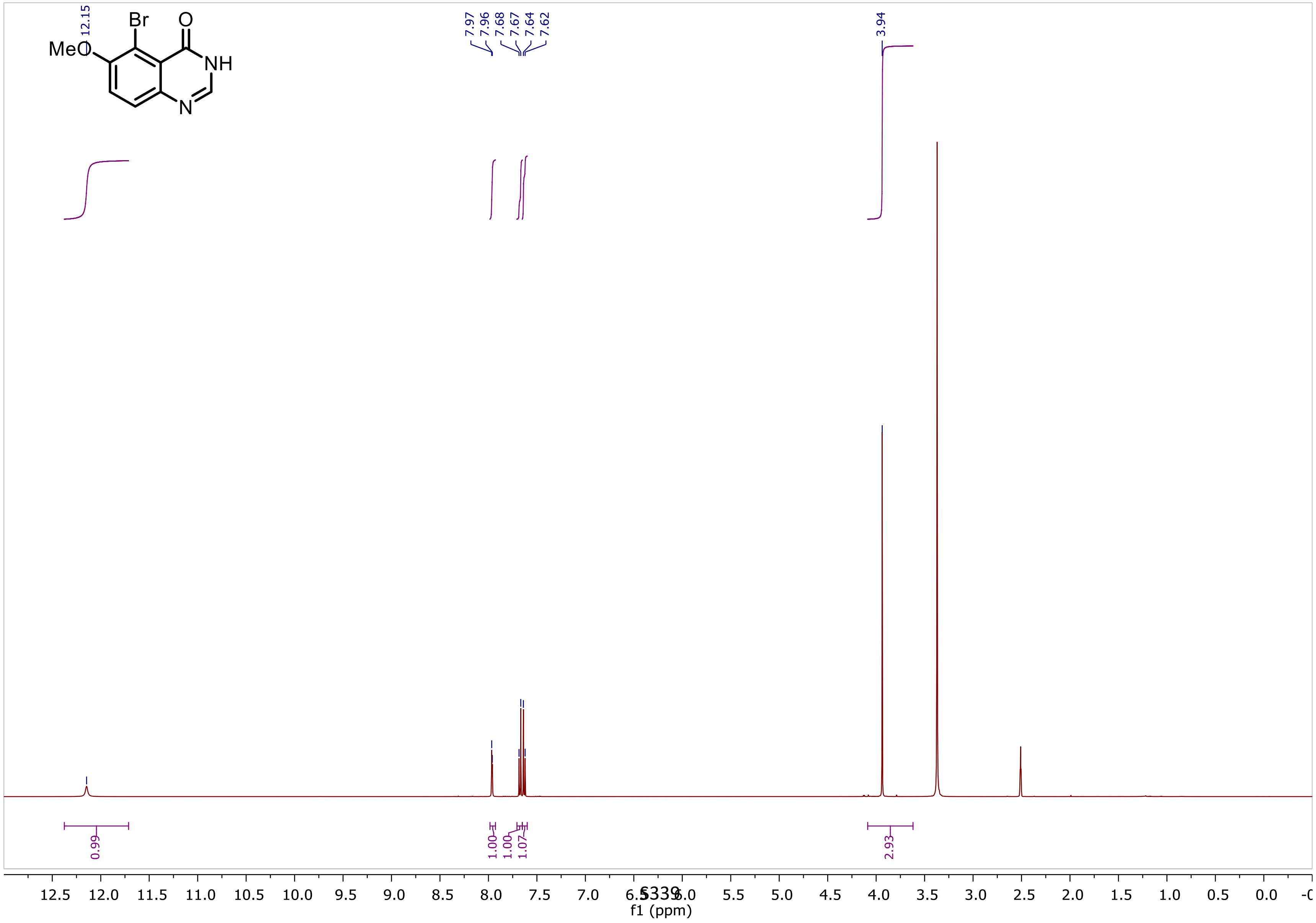

^{13}C NMR spectrum of 5-bromo-6-methoxyquinazolin-4(3*H*)-one **S2ab** in DMSO-d_6

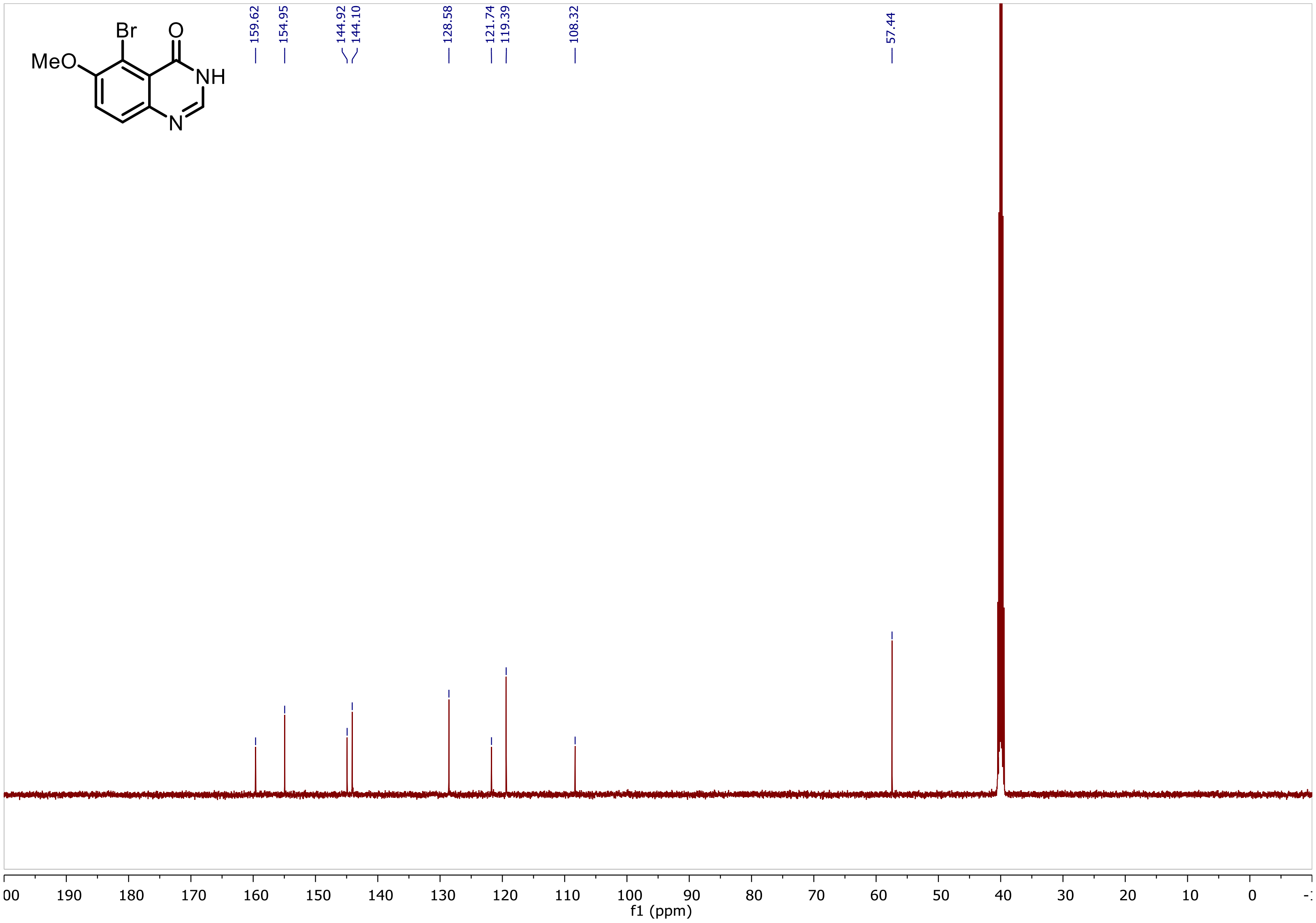

^{1}H NMR spectrum of 2-amino-3,5-dimethoxybenzoic acid hydrochloride **2ac** in MeOD-d_4

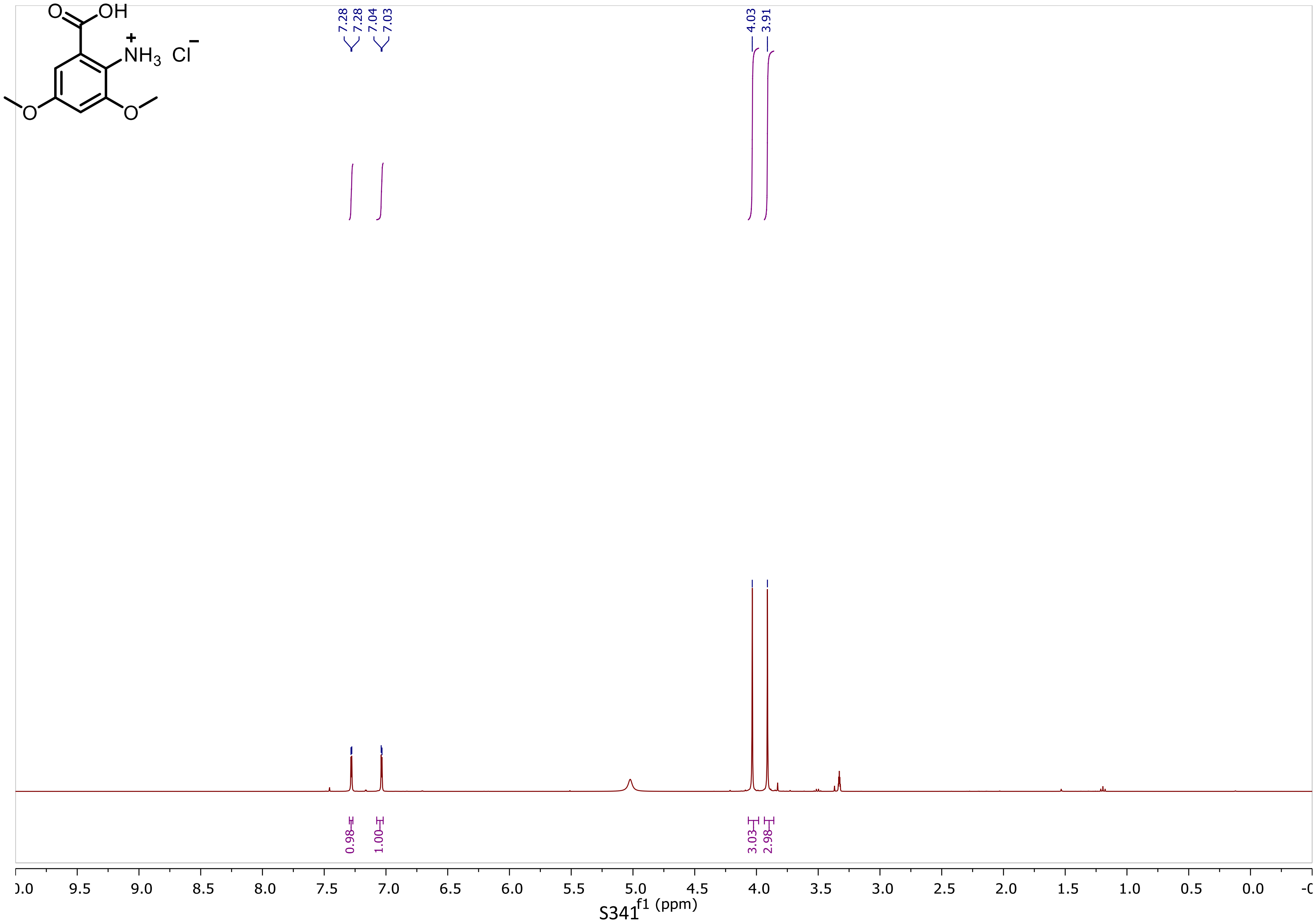

^{13}C NMR spectrum of 2-amino-3,5-dimethoxybenzoic acid hydrochloride **2ac** in MeOD-d_4

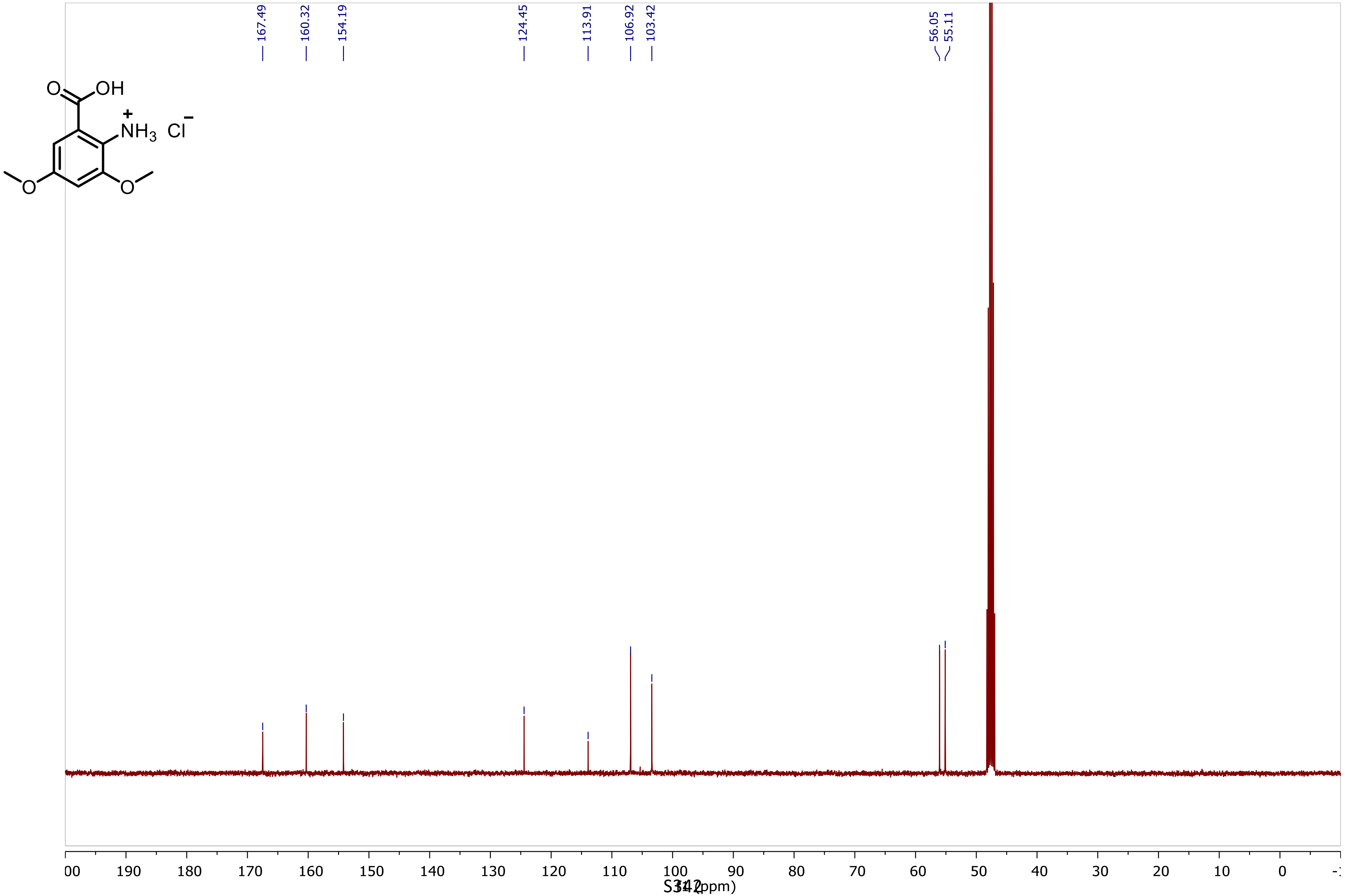

^{1}H NMR spectrum of 2-amino-4-(chloromethyl)benzoic acid hydrochloride **2ag** in MeOD-d_4

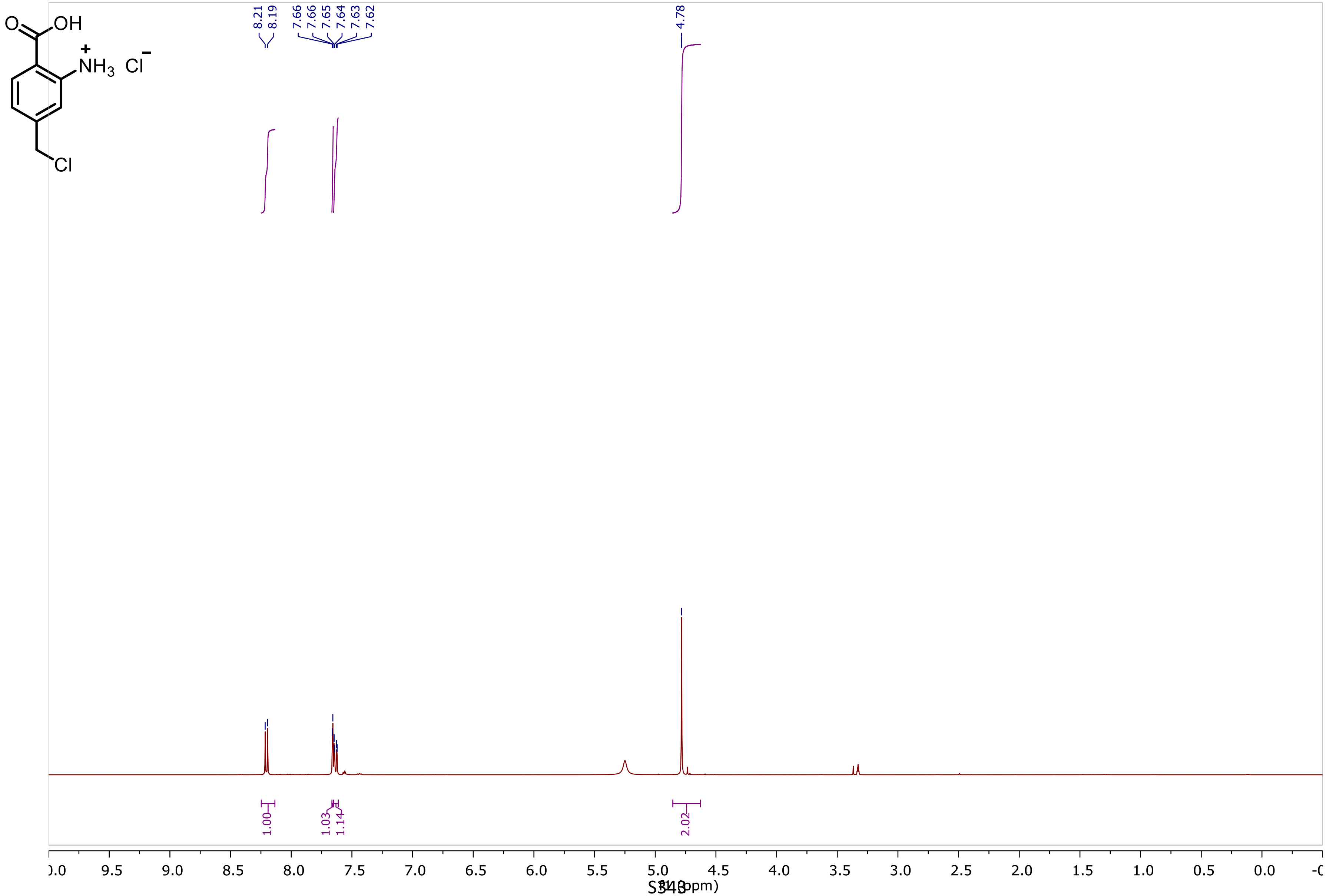

^{13}C NMR spectrum of 2-amino-4-(chloromethyl)benzoic acid hydrochloride **2ag** in MeOD-d_4

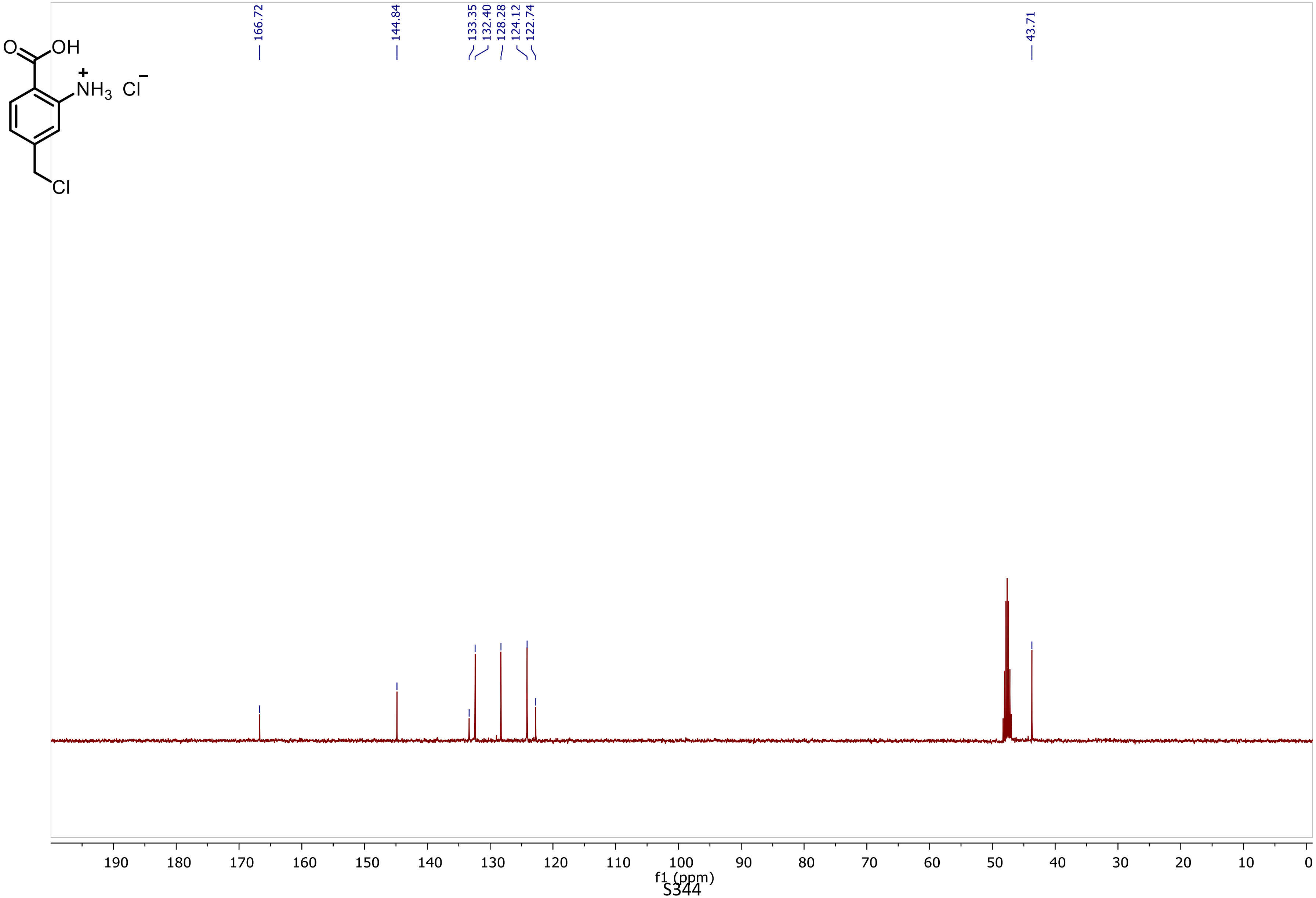

^{1}H NMR spectrum of 2-amino-4-(bromomethyl)benzoic acid hydrochloride **2ah** in MeOD-d_4

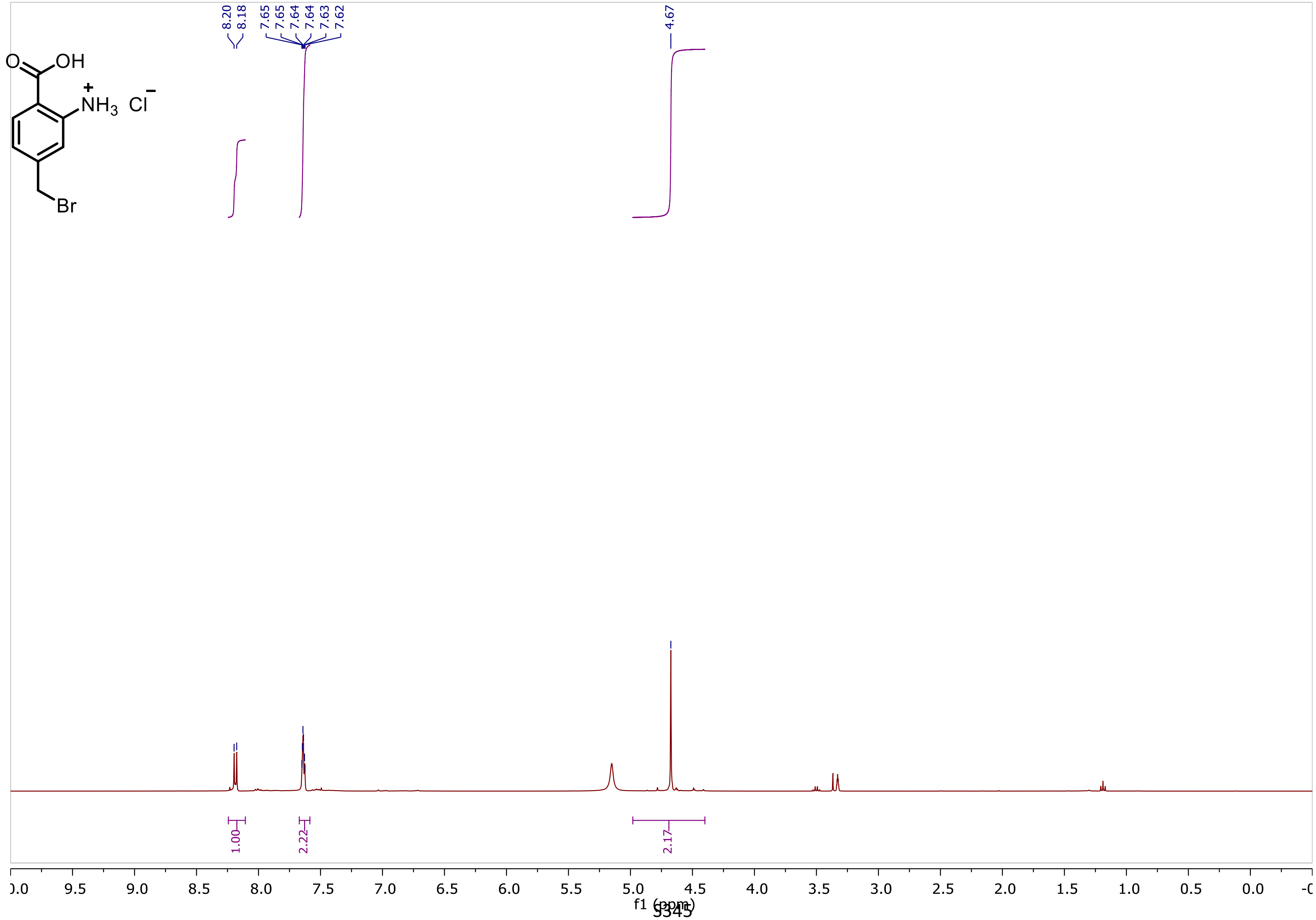

^{13}C NMR spectrum of 2-amino-4-(bromomethyl)benzoic acid hydrochloride **2ah** in MeOD-d_4

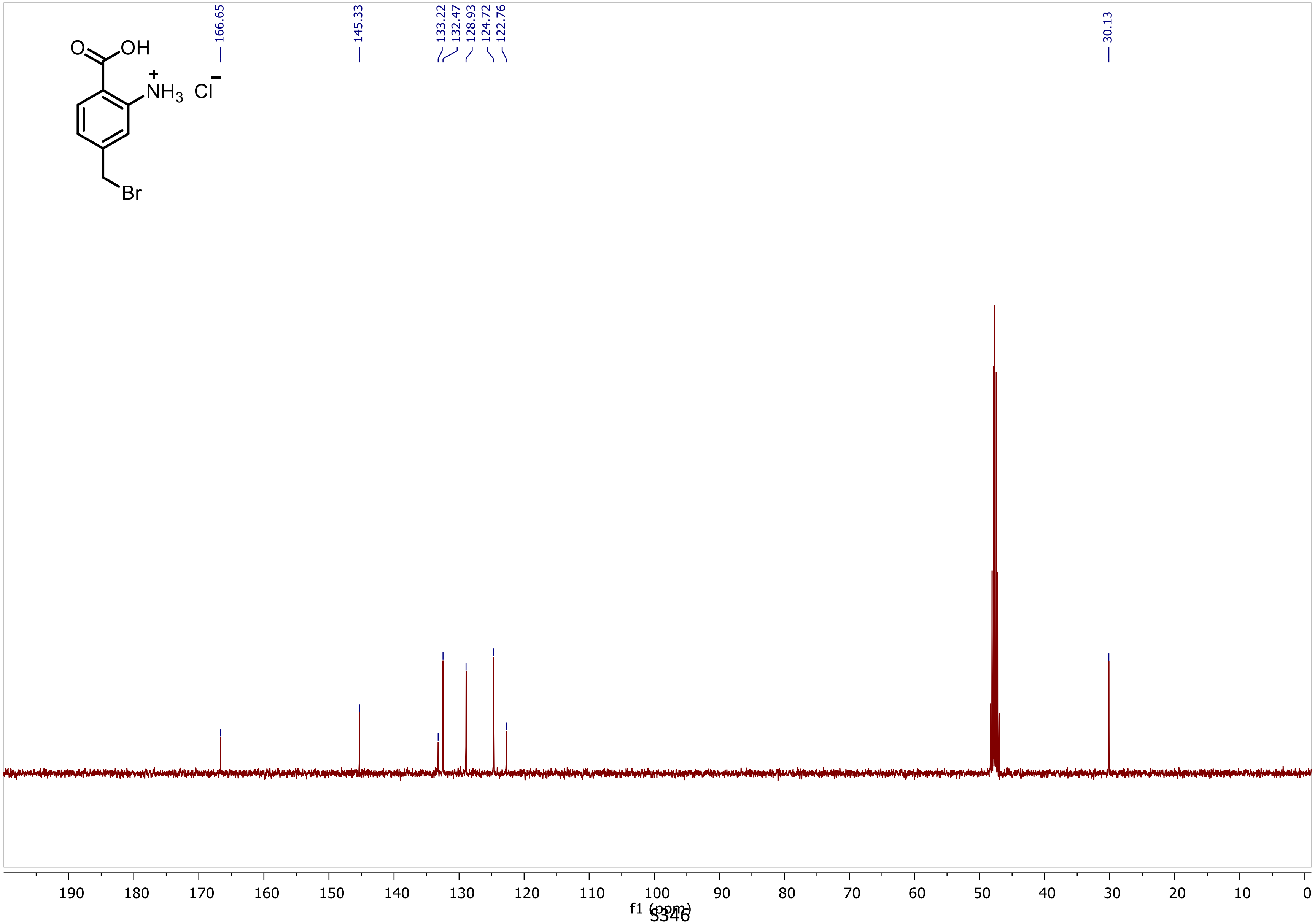

^{1}H NMR spectrum of 2-amino-6-(trifluoromethyl)benzoic acid hydrochloride **2ad** in MeOD-d_4

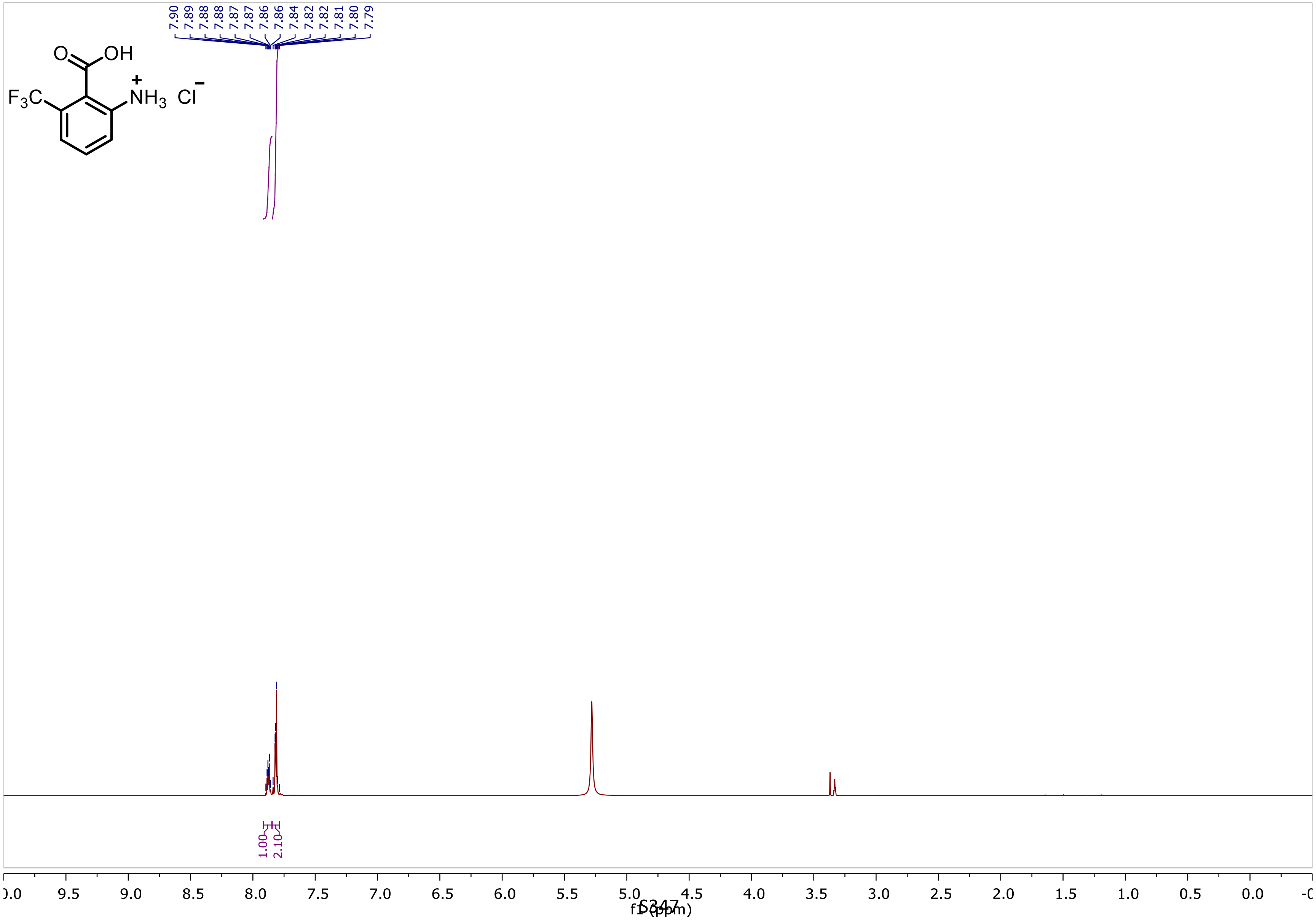

^{13}C NMR spectrum of 2-amino-6-(trifluoromethyl)benzoic acid hydrochloride **2ad** in MeOD-d_4

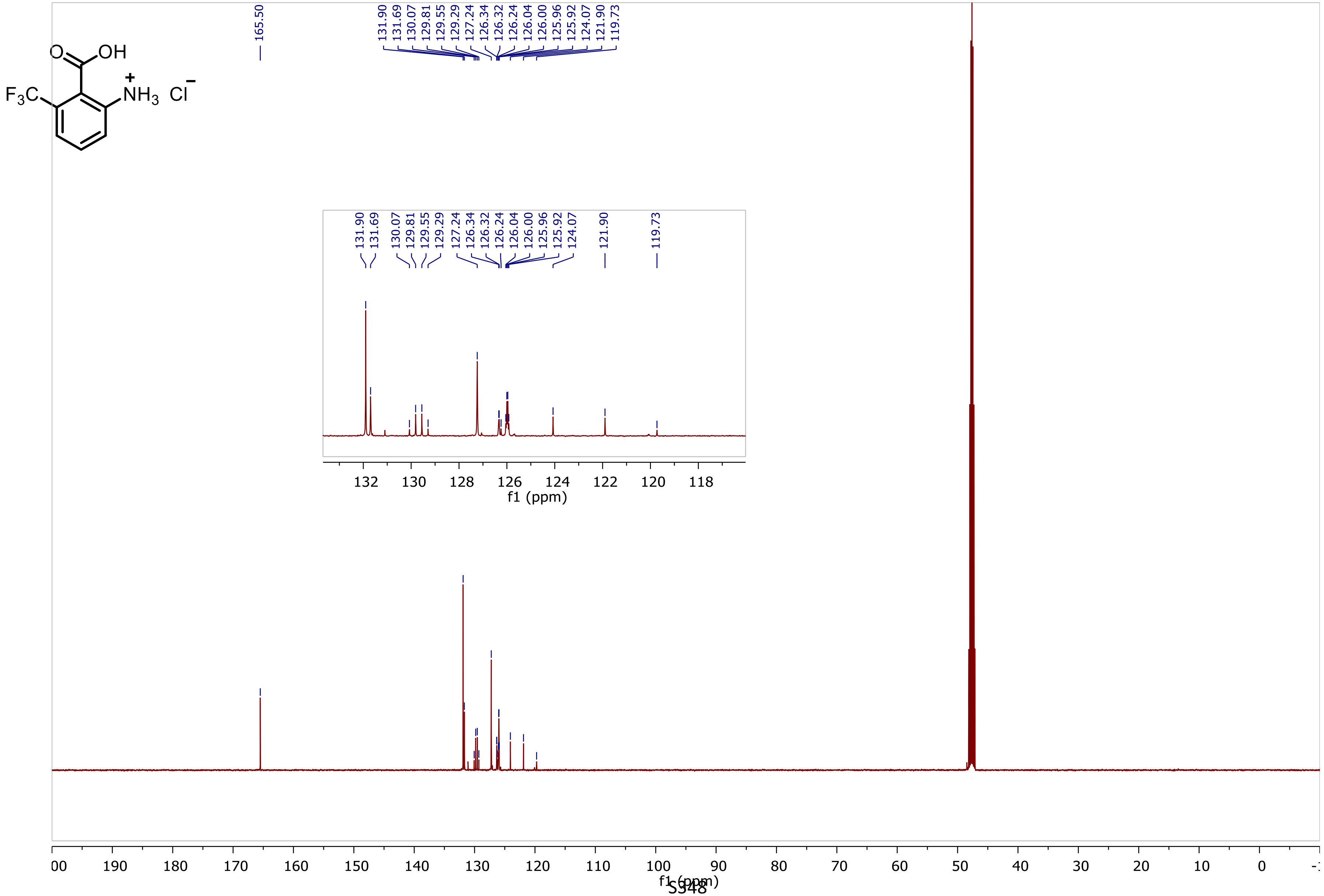

^{19}F NMR spectrum of 2-amino-6-(trifluoromethyl)benzoic acid hydrochloride **2ad** in MeOD-d_4

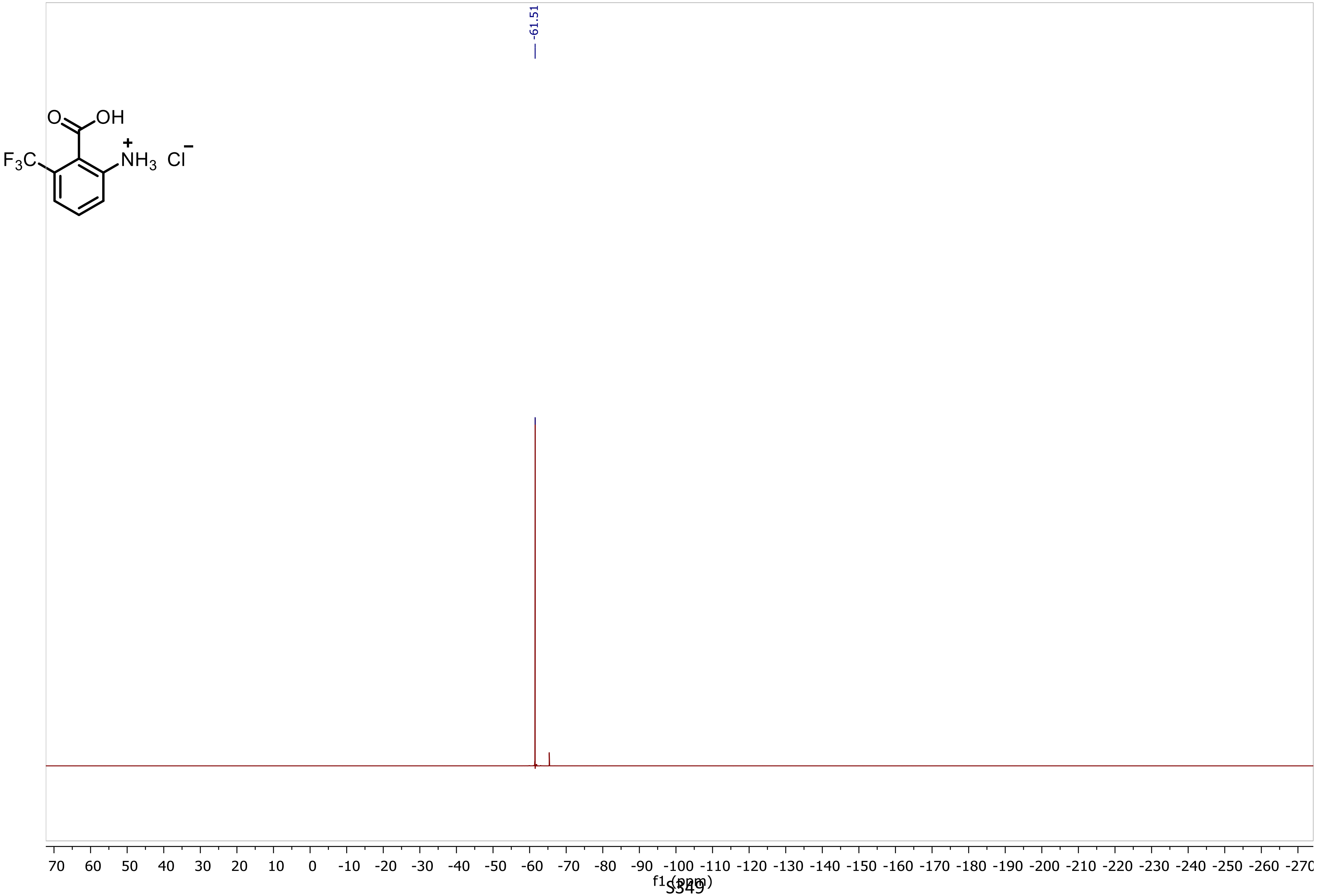

^{1}H NMR spectrum of 2-amino-4-(trifluoromethyl)benzoic acid **2ae** in MeOD-d_4

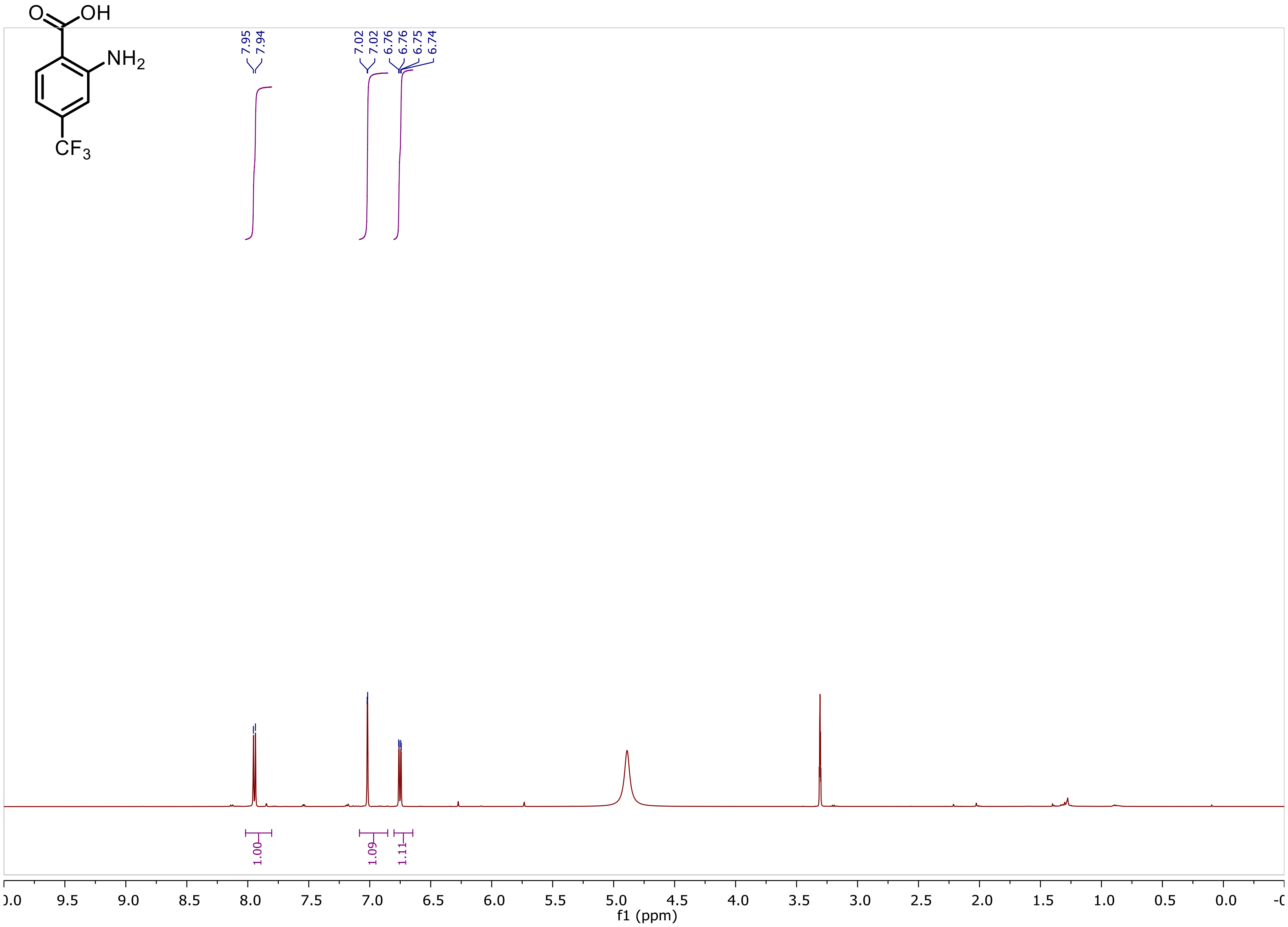

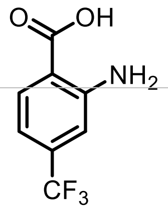

^{13}C NMR spectrum of 2-amino-4-(trifluoromethyl)benzoic acid **2ae** in MeOD-d_4

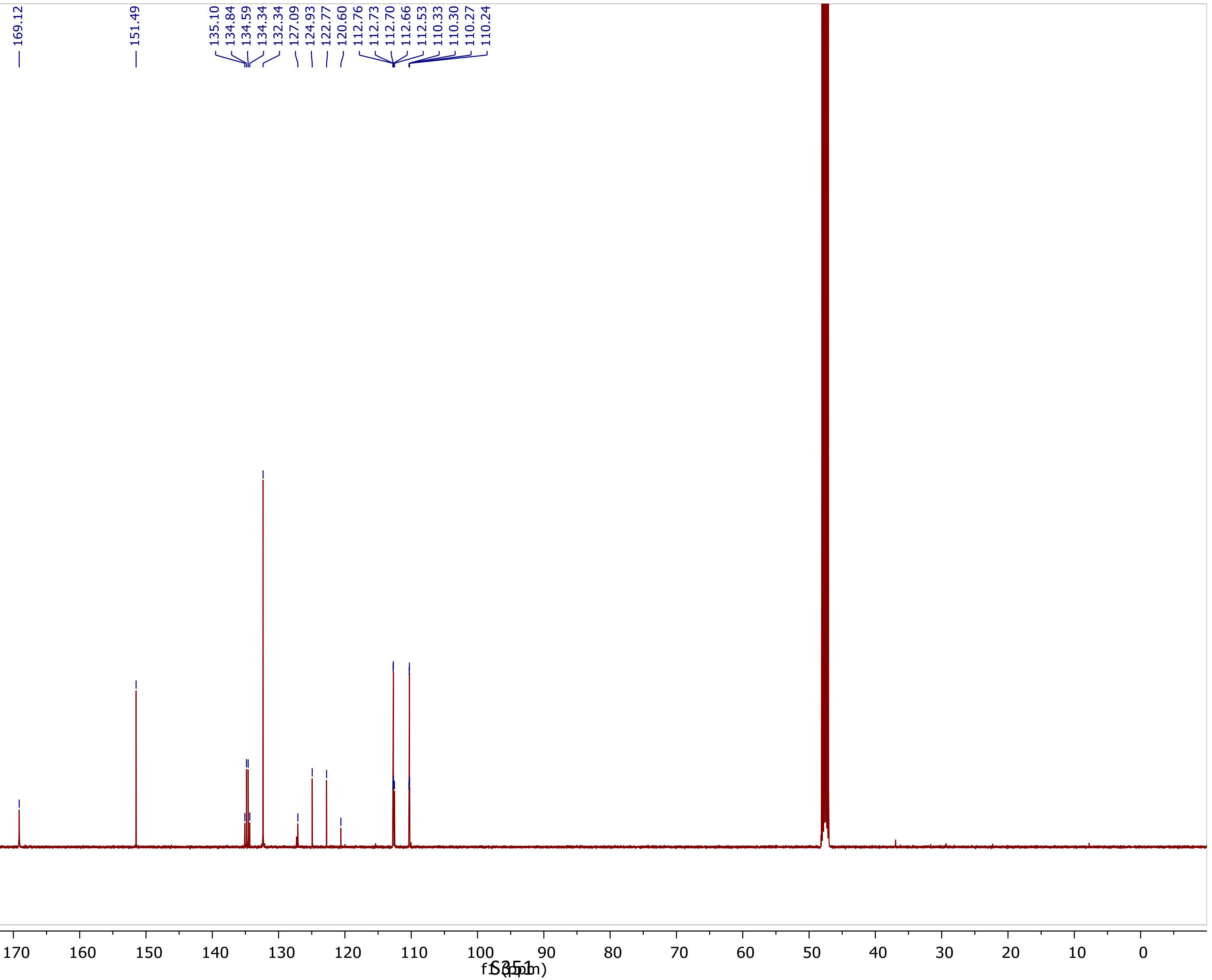

^{19}F NMR spectrum of 2-amino-4-(trifluoromethyl)benzoic acid **2ae** in MeOD-d_4

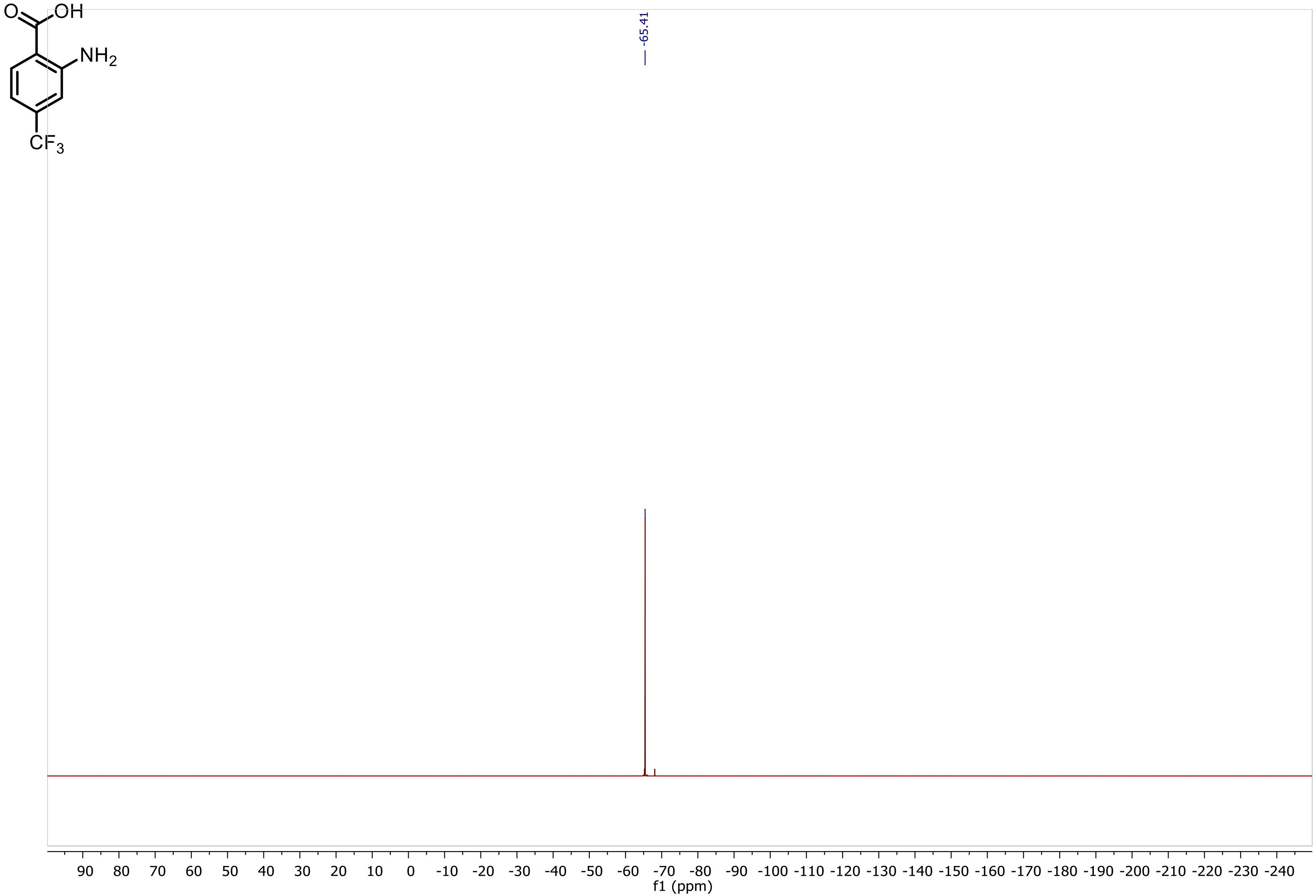

^{1}H NMR spectrum of 2-amino-4-(difluoromethoxy)benzoic acid **2af** in MeOD-d_4

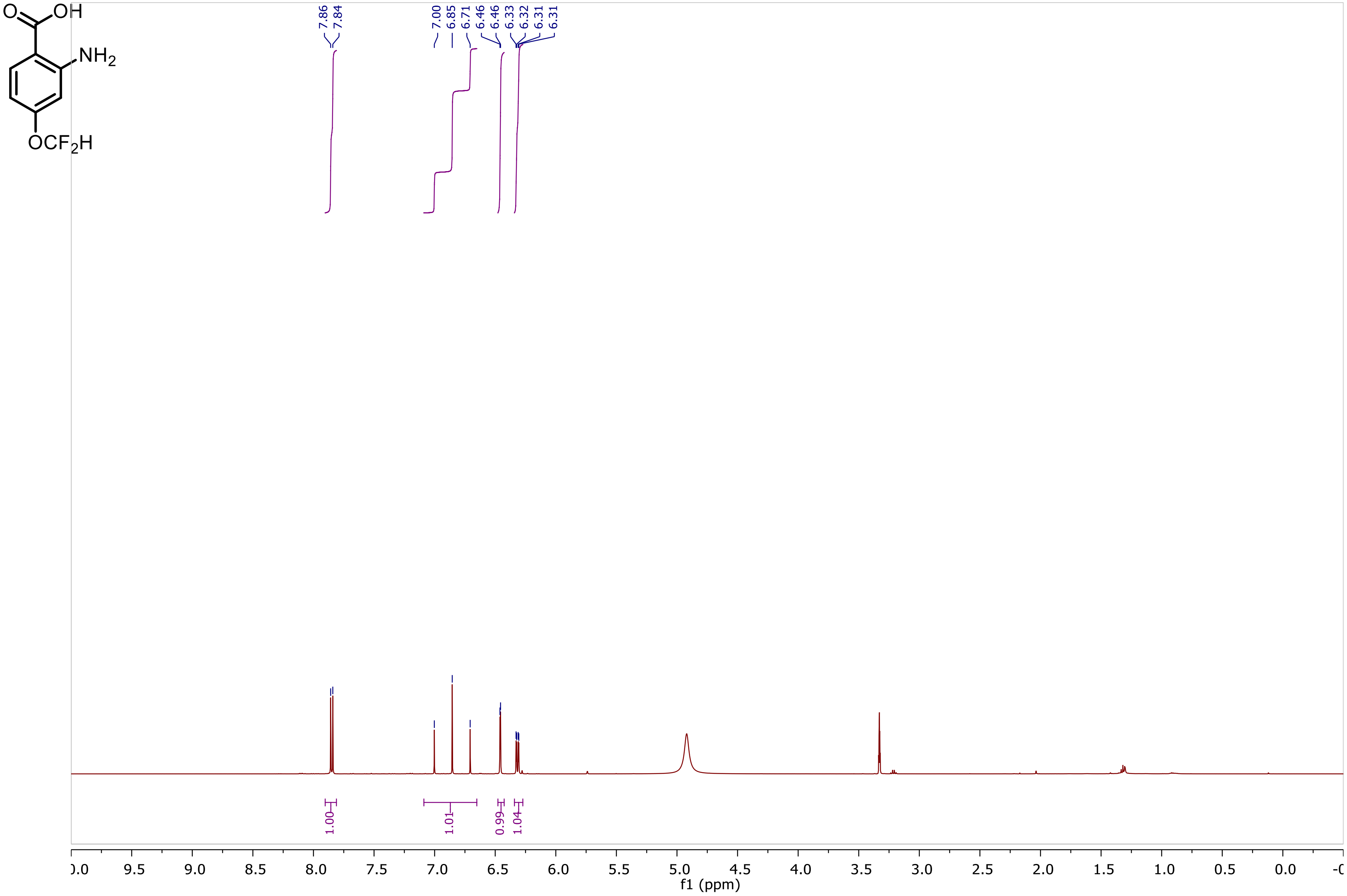

^{13}C NMR spectrum of 2-amino-4-(difluoromethoxy)benzoic acid **2af** in MeOD-d_4

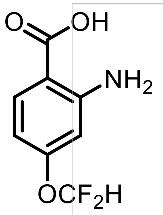

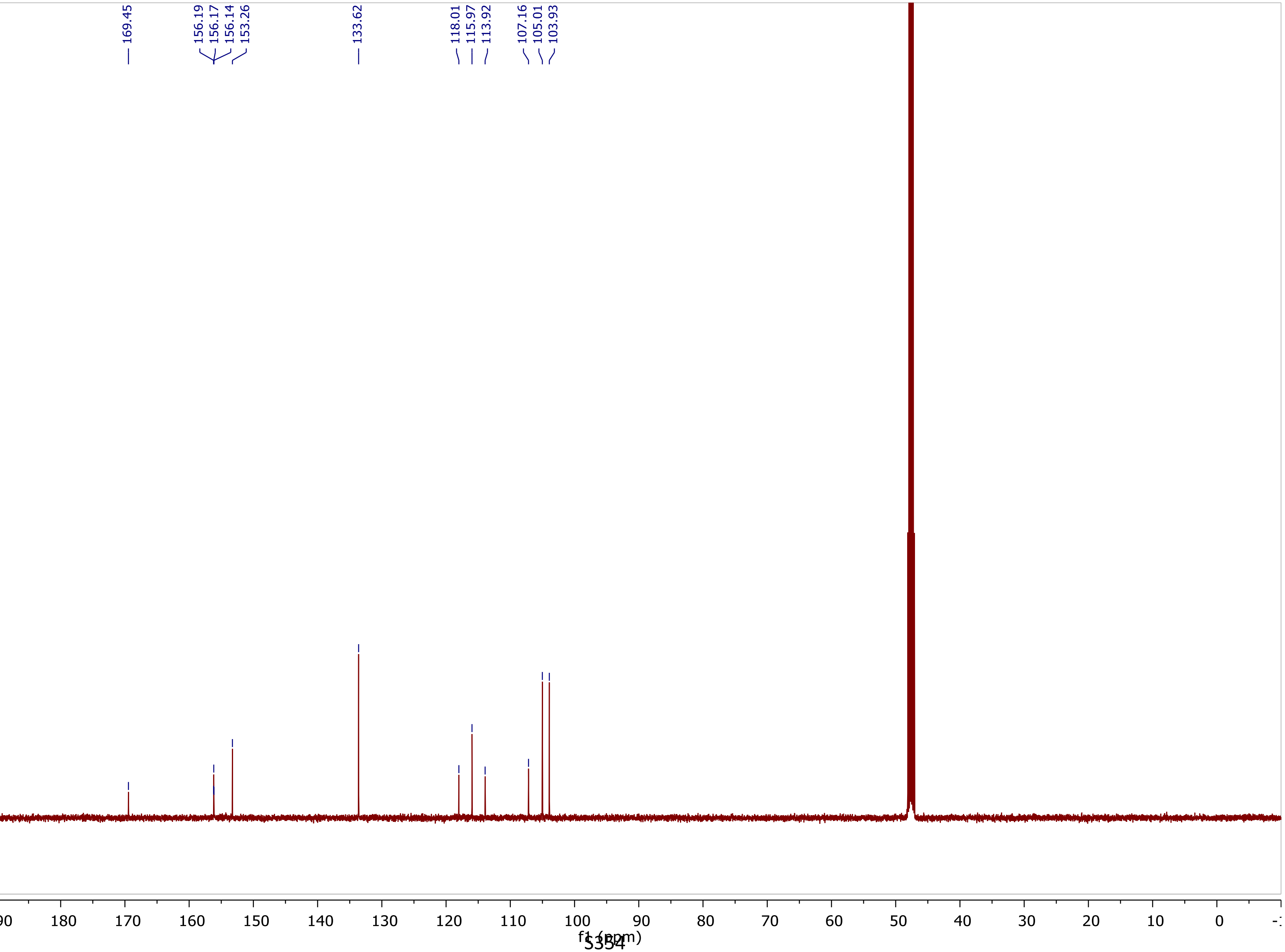

^{19}F NMR spectrum of 2-amino-4-(difluoromethoxy)benzoic acid **2af** in MeOD-d_4

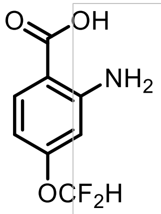

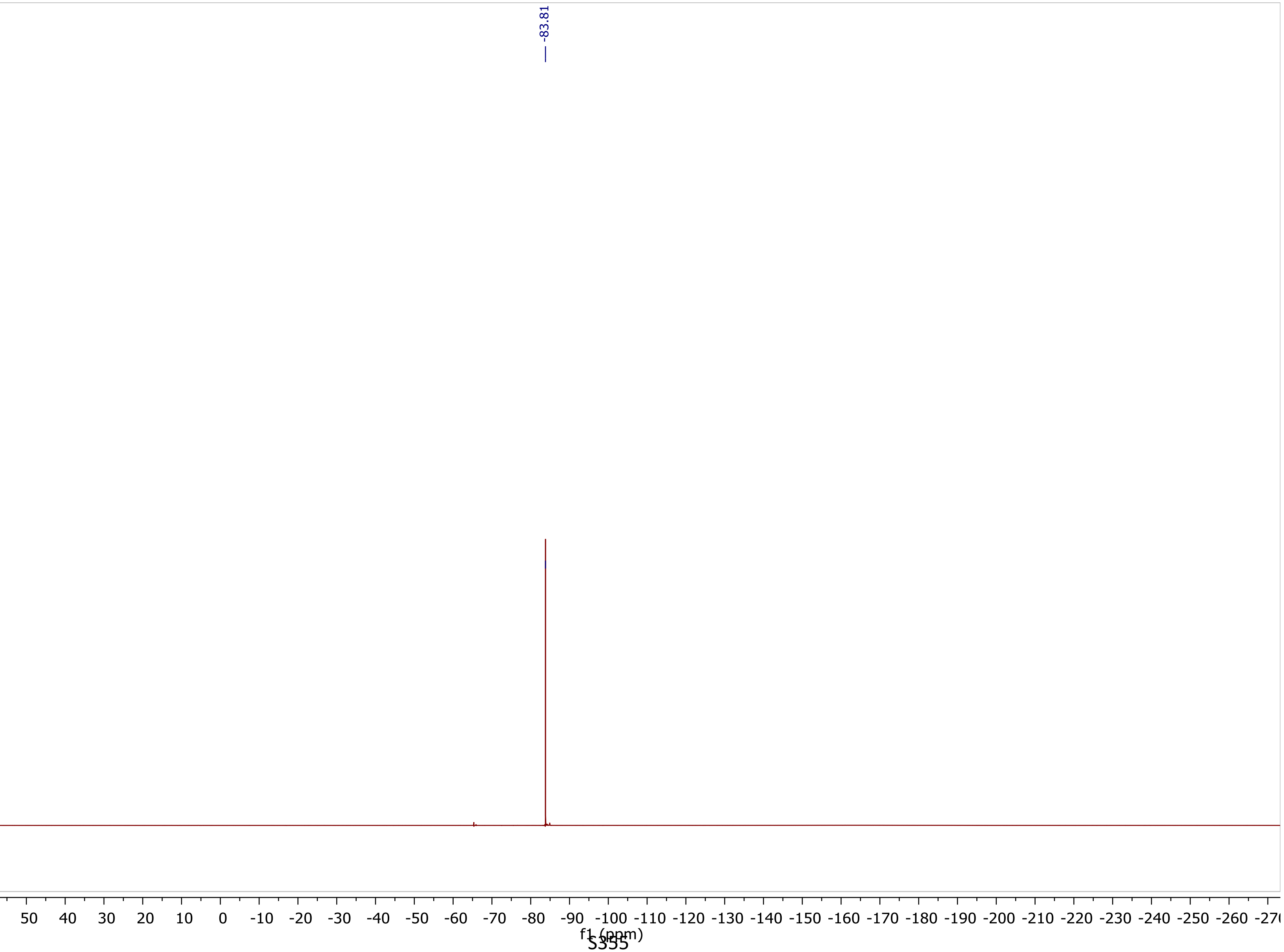

^{1}H NMR spectrum of 7-(difluoromethoxy)quinazolin-4(3*H*)-one **S2af** in MeOD-d_4

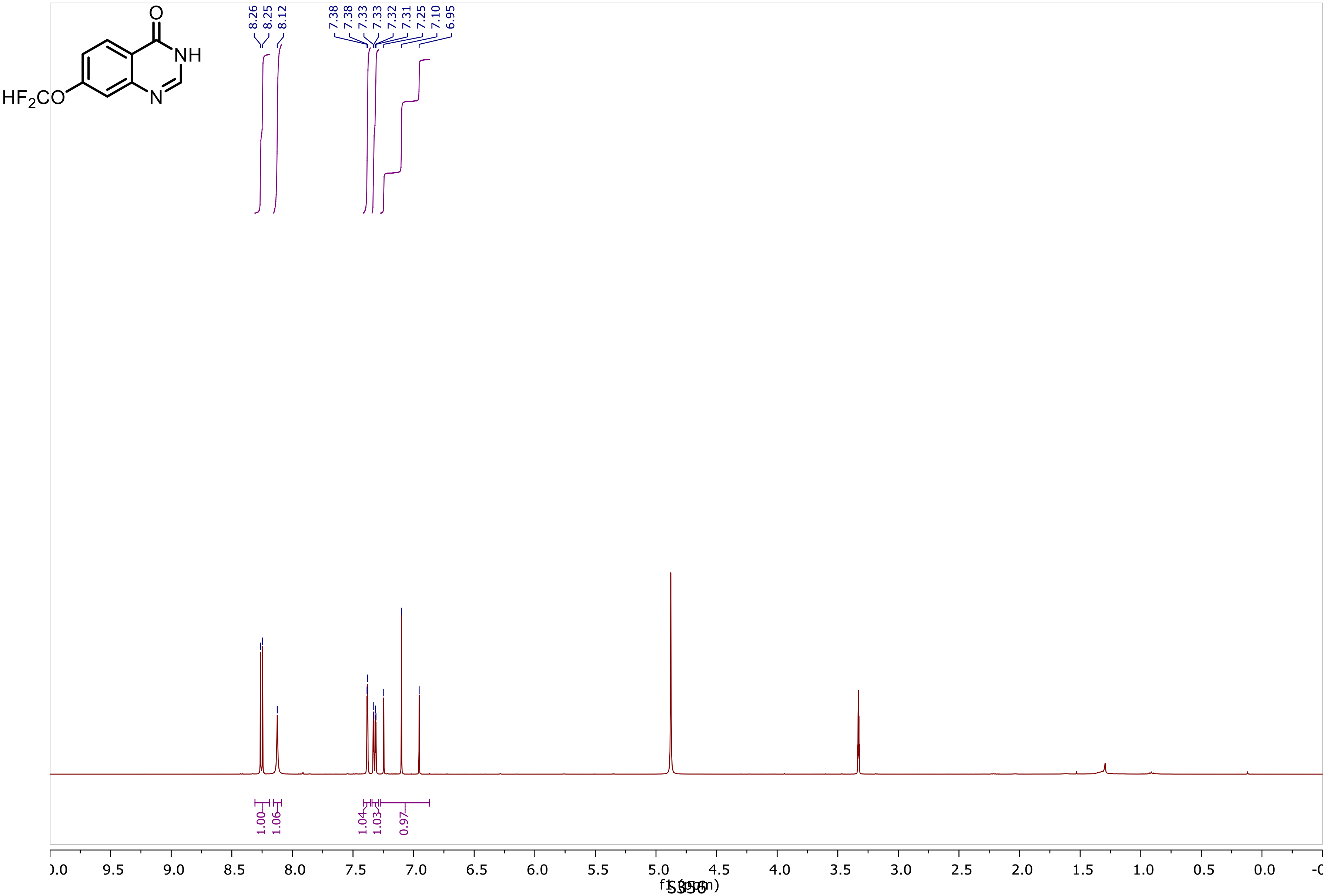

^{13}C NMR spectrum of 7-(difluoromethoxy)quinazolin-4(3*H*)-one **S2af** in MeOD-d_4

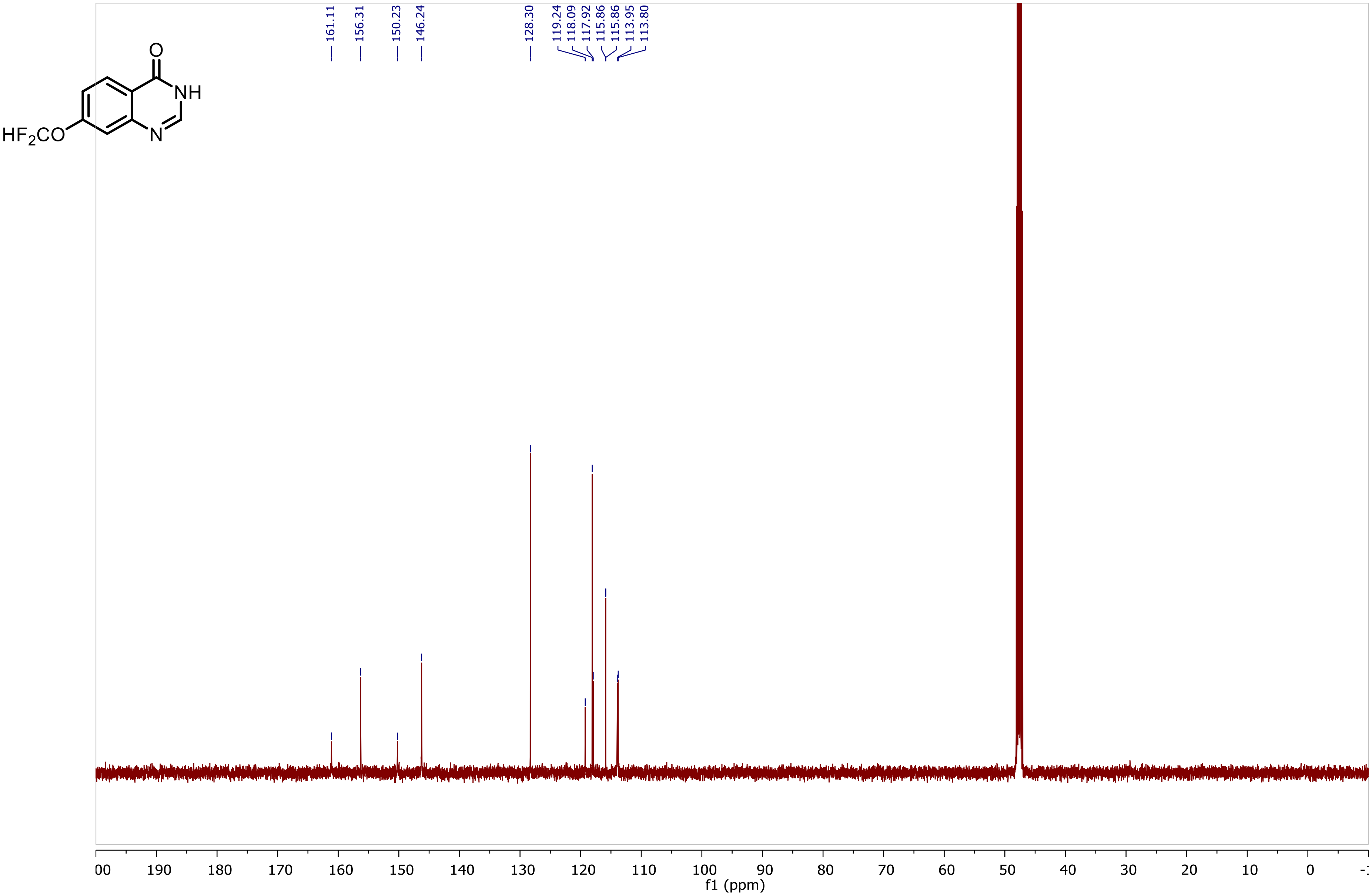

^{19}F NMR spectrum of 7-(difluoromethoxy)quinazolin-4(3*H*)-one **S2af** in MeOD-d_4

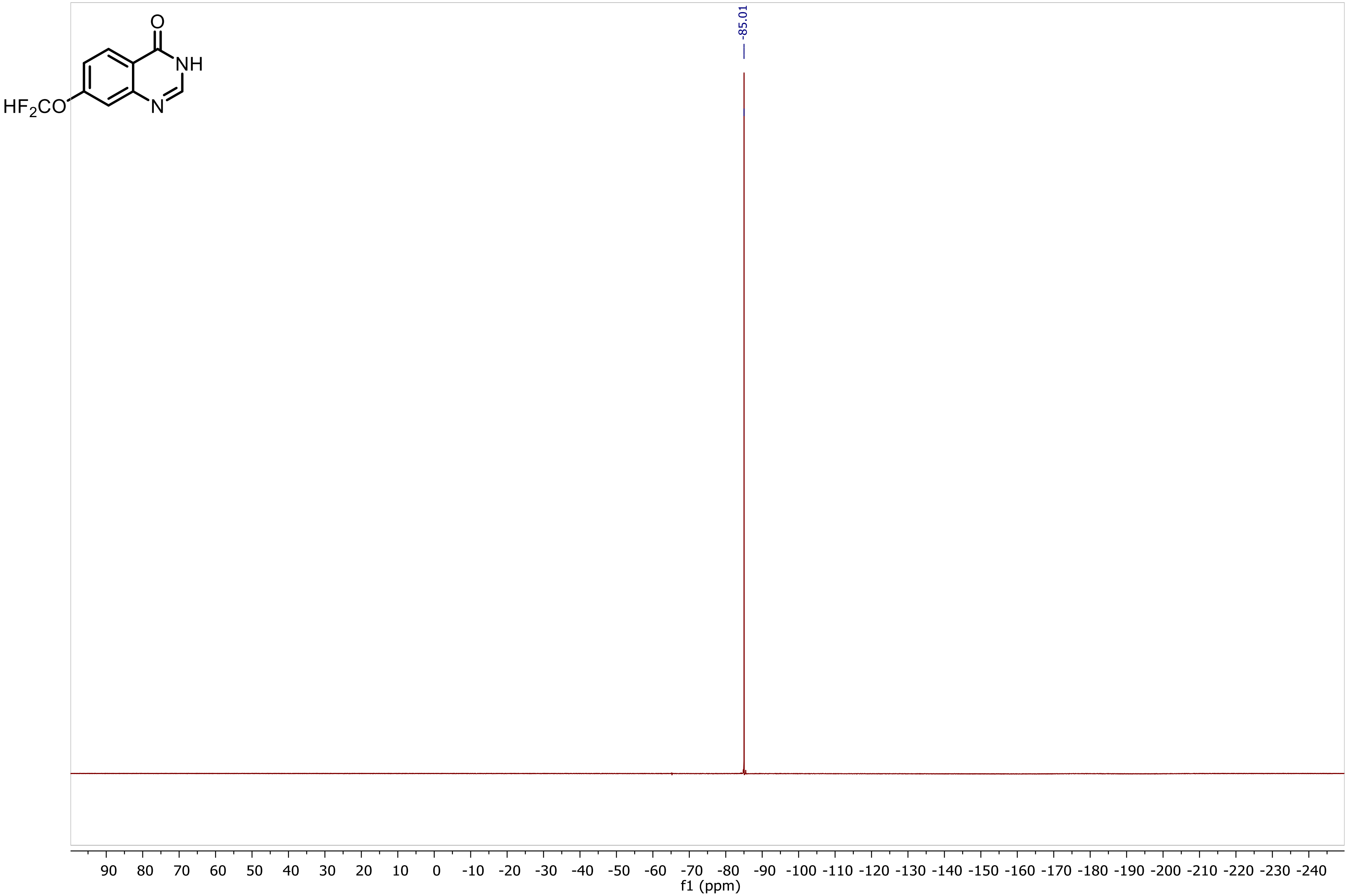

^{1}H NMR spectrum of 5-methoxy-2-(methylamino)benzoic acid hydrochloride **4a (a)** and 3-methoxy-2-(methylamino)benzoic acid hydrochloride ***iso*-4a (b)** in MeOD-d_4

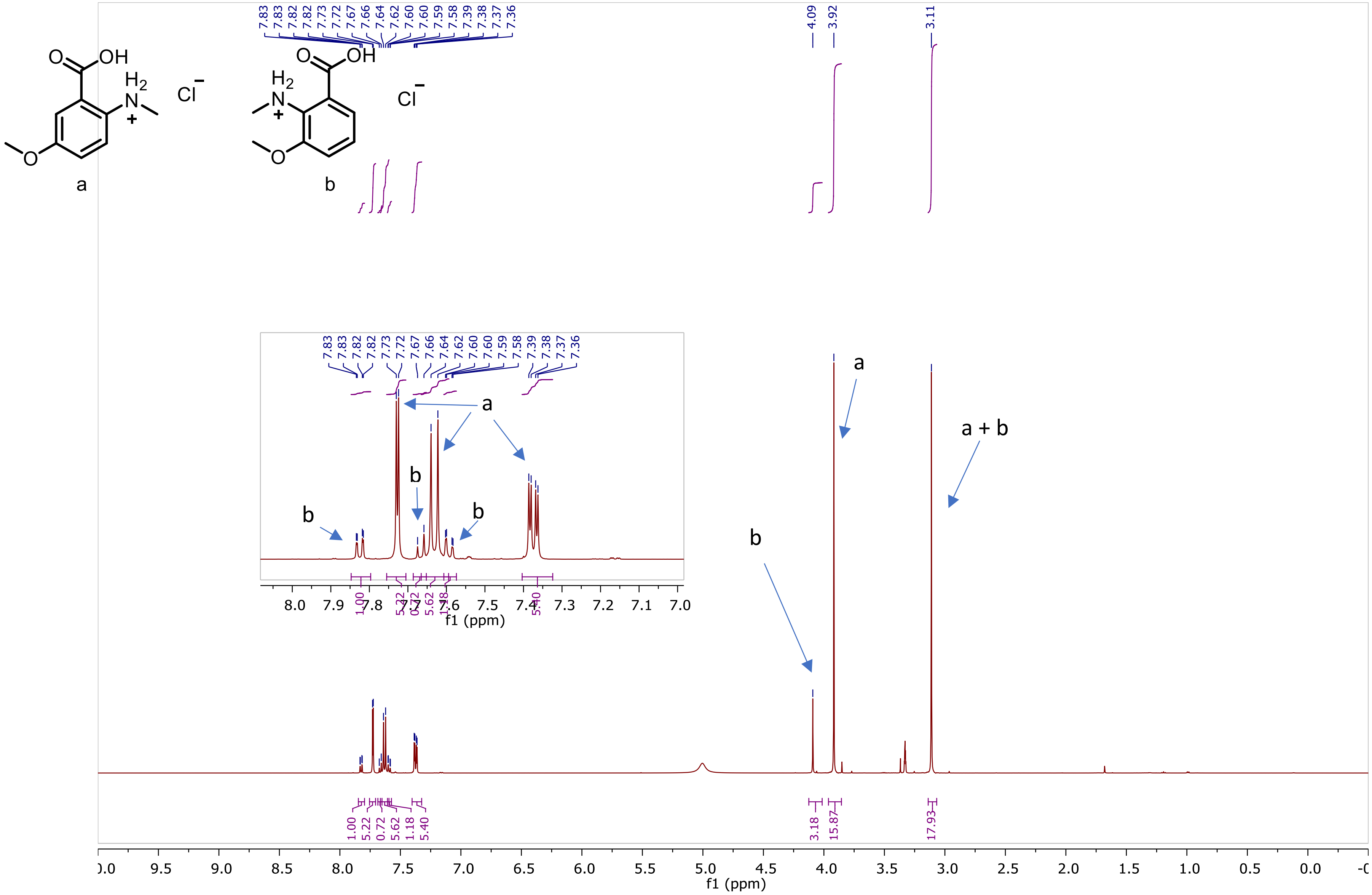

^{13}C NMR spectrum of 5-methoxy-2-(methylamino)benzoic acid hydrochloride **4a** and 3-methoxy-2-(methylamino)benzoic acid hydrochloride ***iso*-4a** in MeOD-d_4

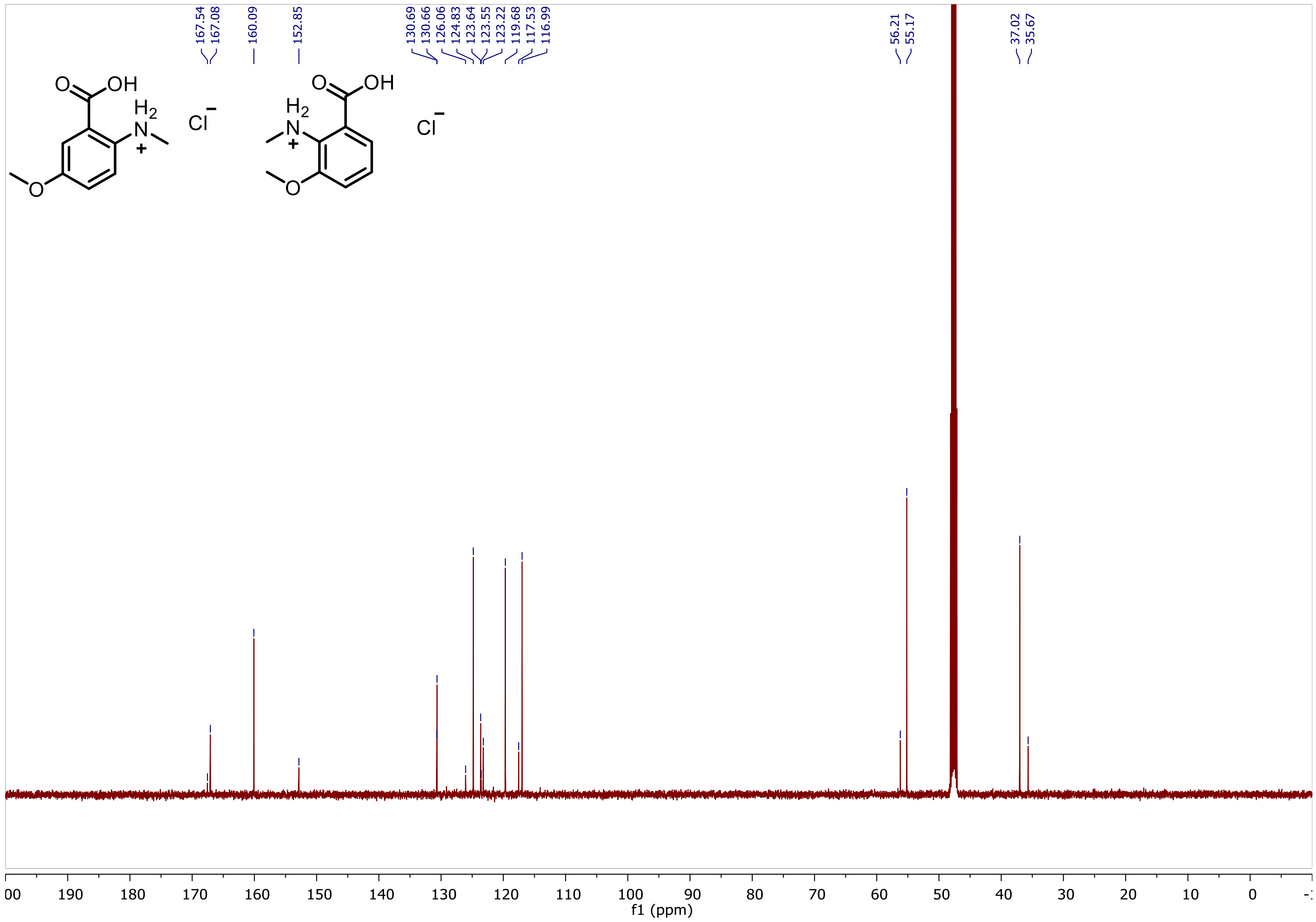

^{1}H NMR spectrum of 5-methyl-2-(methylamino)benzoic acid hydrochloride **4f (a)** and 3-methyl-2-(methylamino)benzoic acid hydrochloride ***iso*-4f (b)** in MeOD-d_4

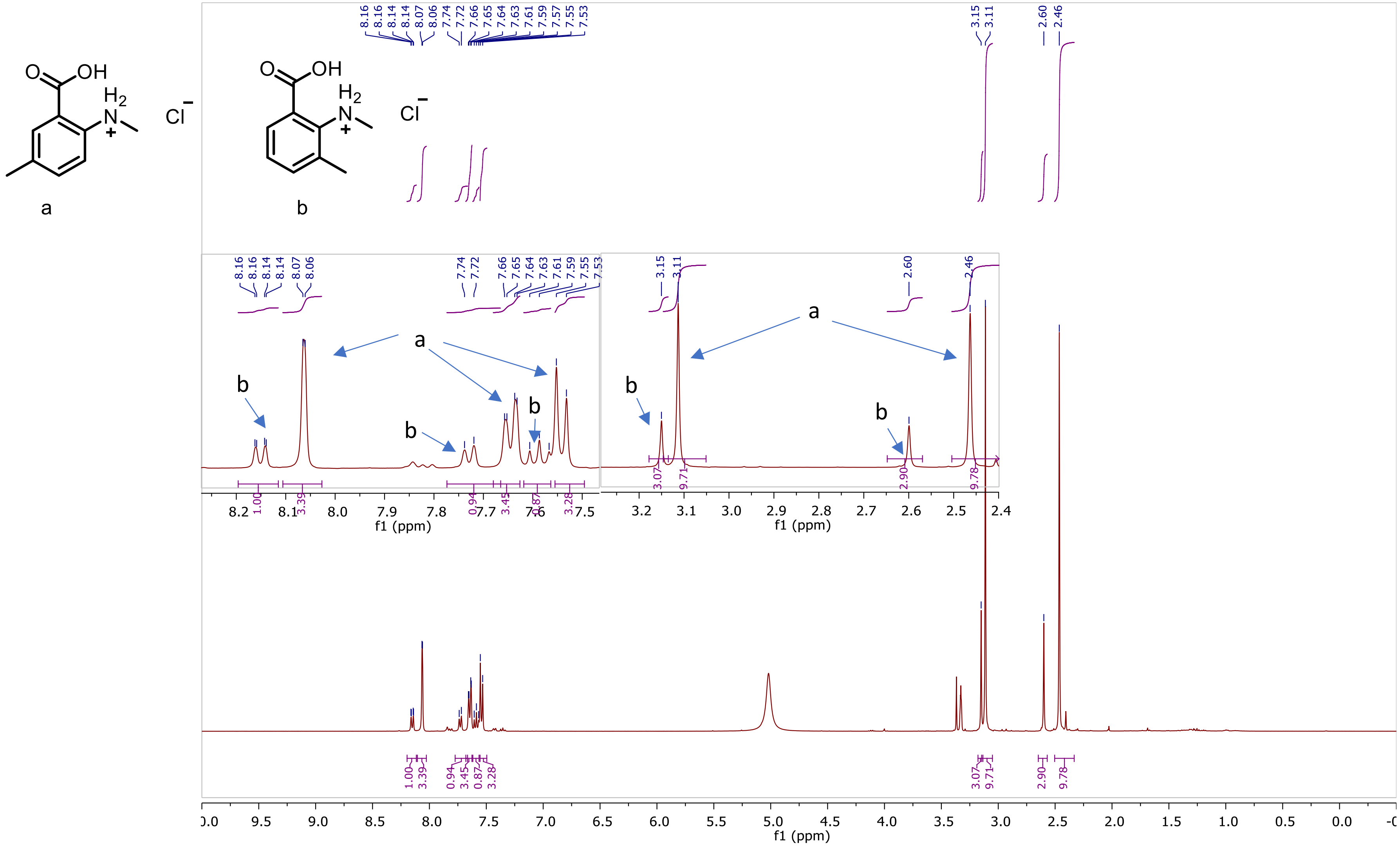

^{13}C NMR spectrum of 5-methyl-2-(methylamino)benzoic acid hydrochloride **4f** and 3-methyl-2-(methylamino)benzoic acid hydrochloride ***iso*-4f** in MeOD-d_4

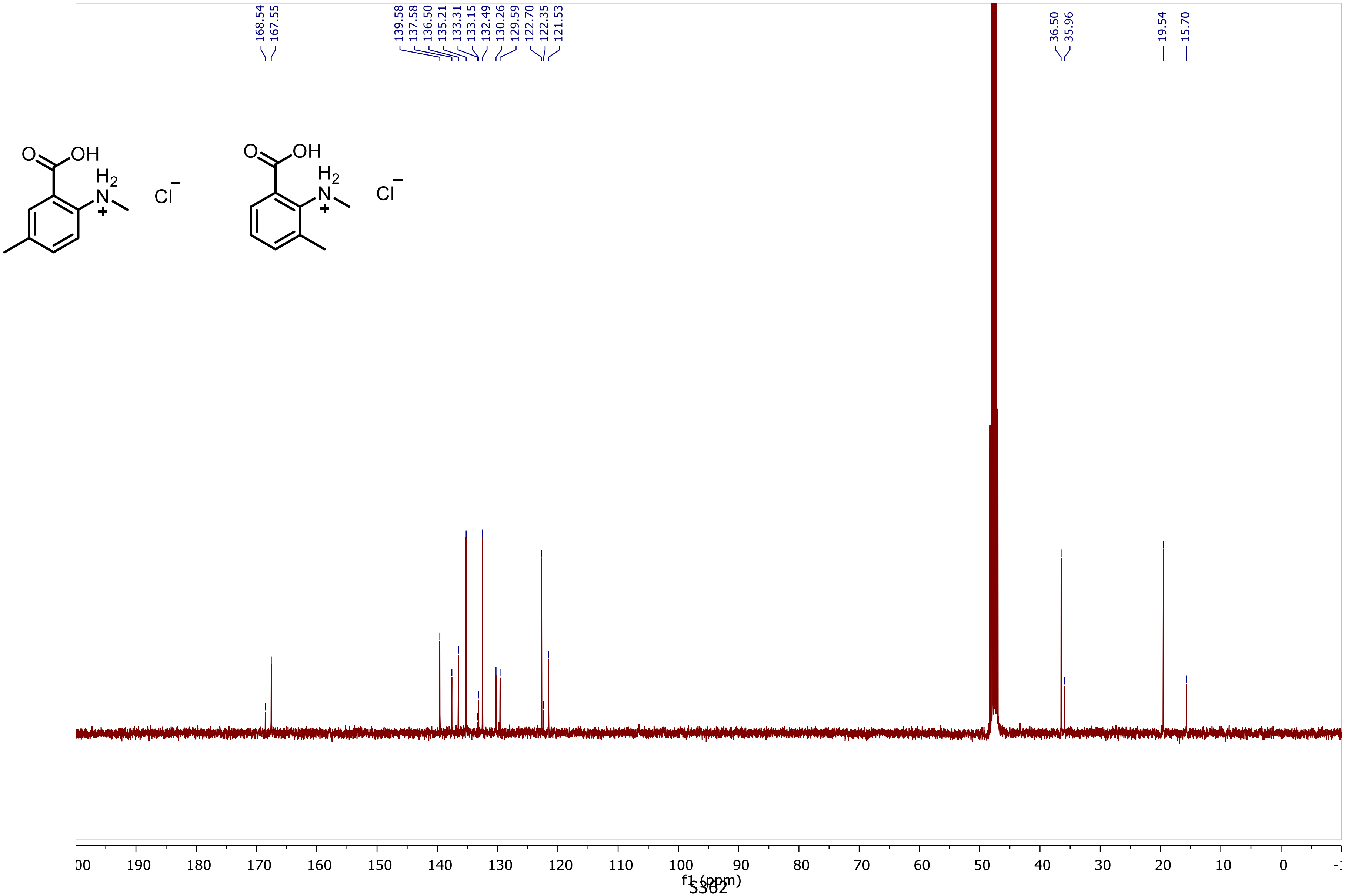

^{1}H NMR spectrum of 3-methoxy-2-methyl-6-(methylamino)benzoic acid hydrochloride **4e** in MeOD-d_4

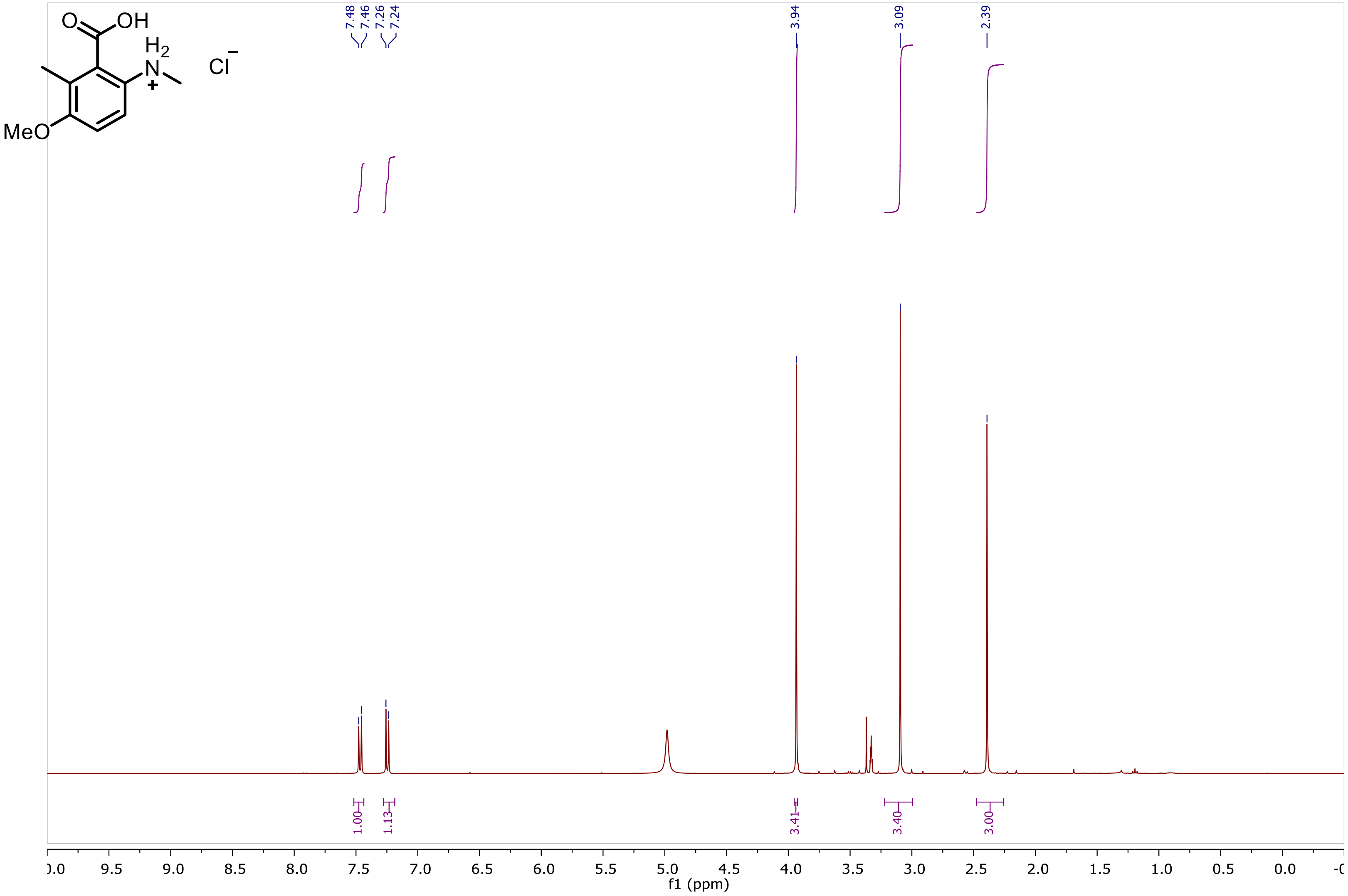

^{13}C NMR spectrum of 3-methoxy-2-methyl-6-(methylamino)benzoic acid hydrochloride **4e** in MeOD-d_4

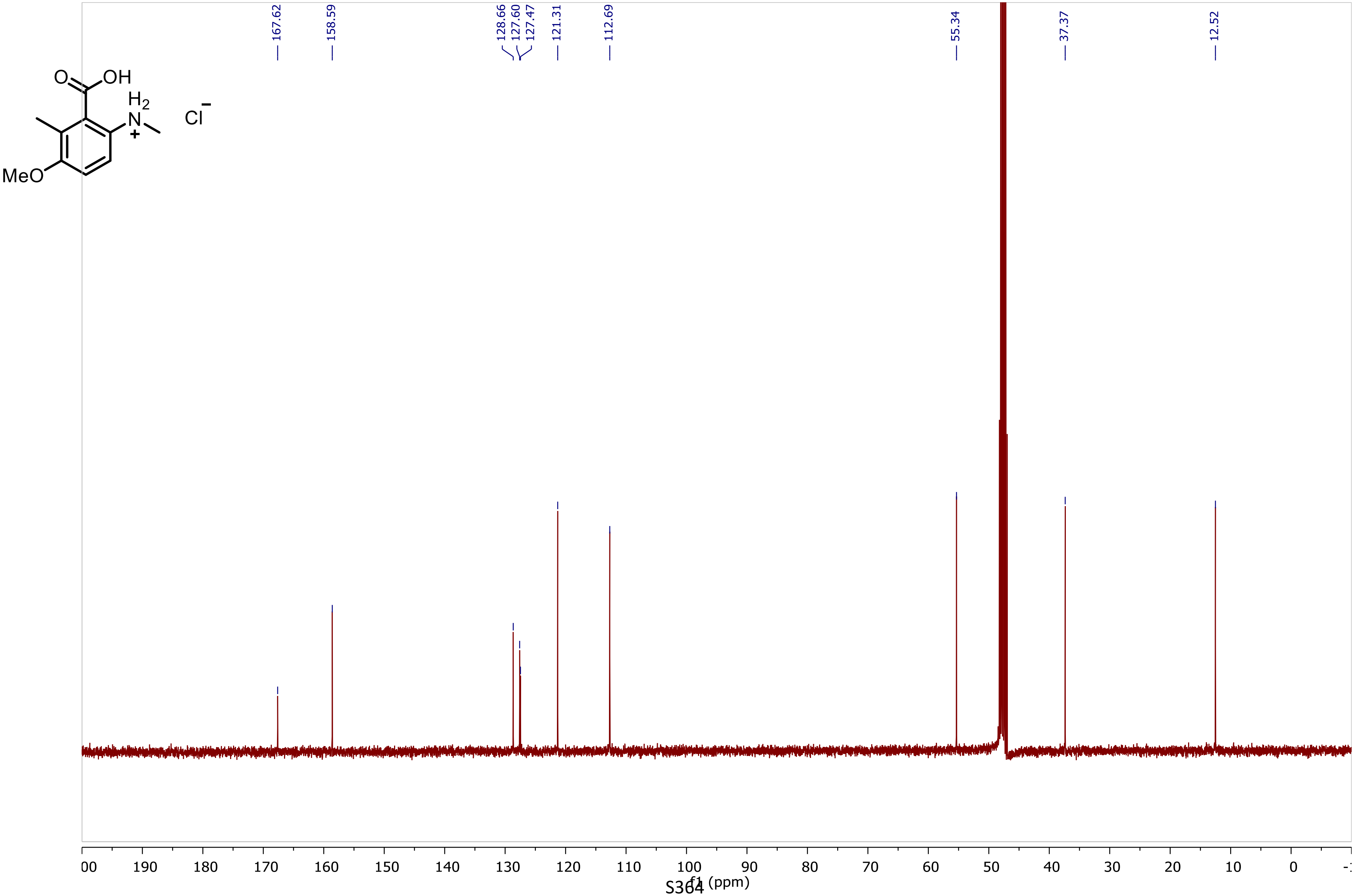

^{1}H NMR spectrum of 2-bromo-3-methoxy-6-(methylamino)benzoic acid hydrochloride **4d** in MeOD-d_4

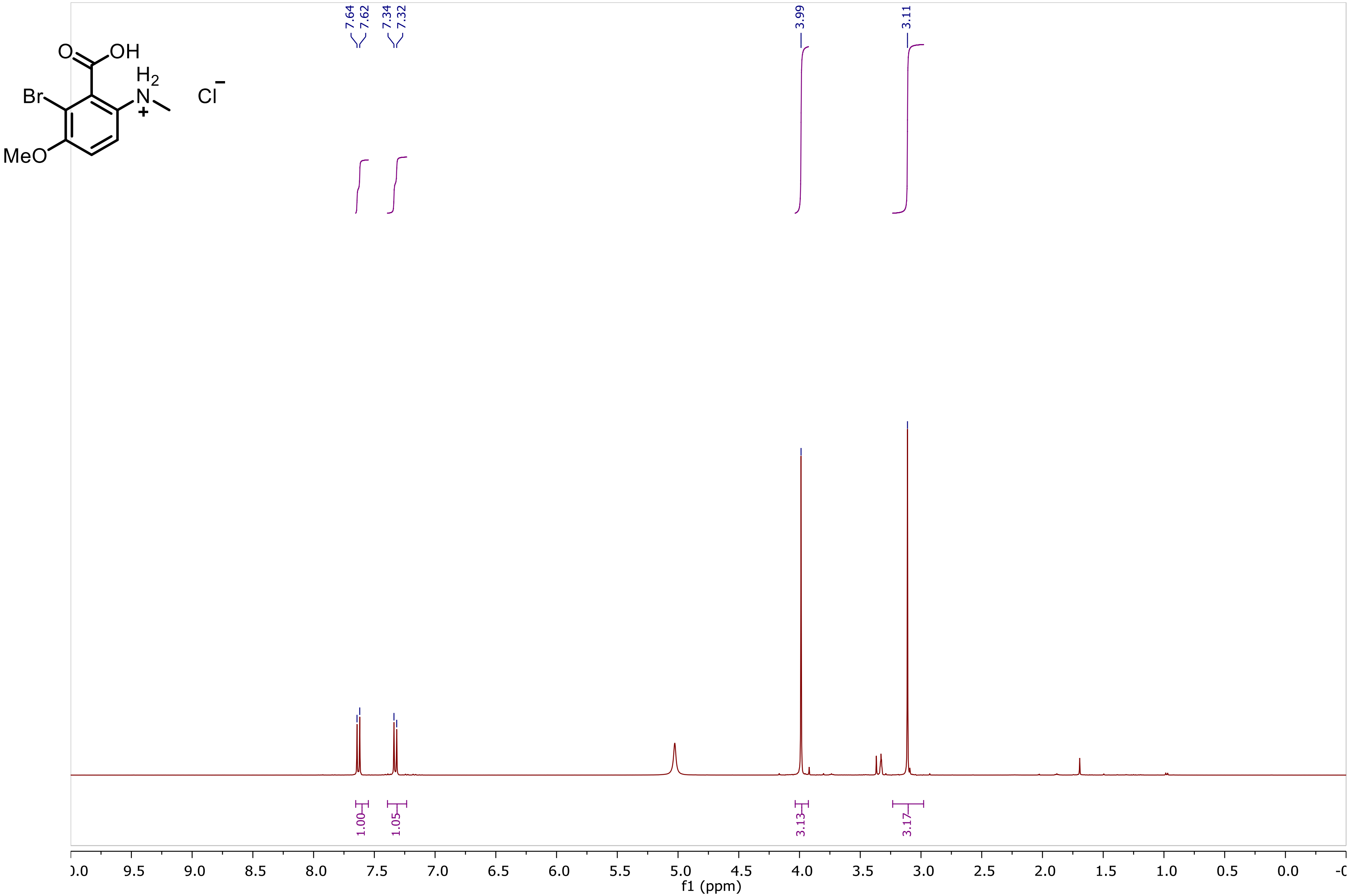

^{13}C NMR spectrum of 2-bromo-3-methoxy-6-(methylamino)benzoic acid hydrochloride **4d** in MeOD-d_4

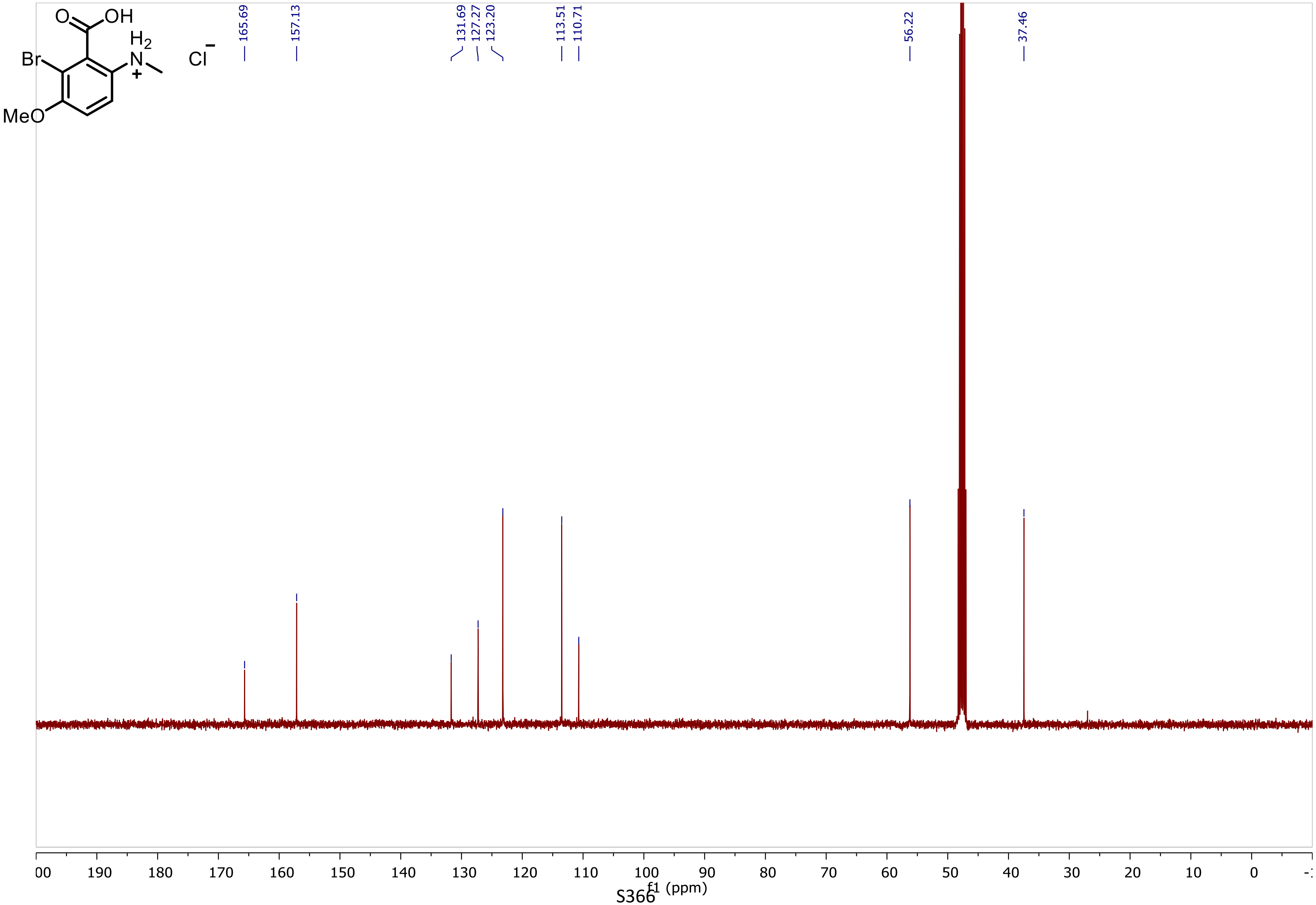

^{1}H NMR spectrum of 3,5-dimethoxy-2-(methylamino)benzoic acid hydrochloride **4b** in MeOD-d_4

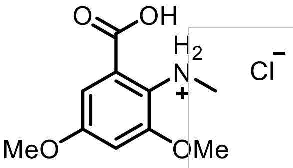

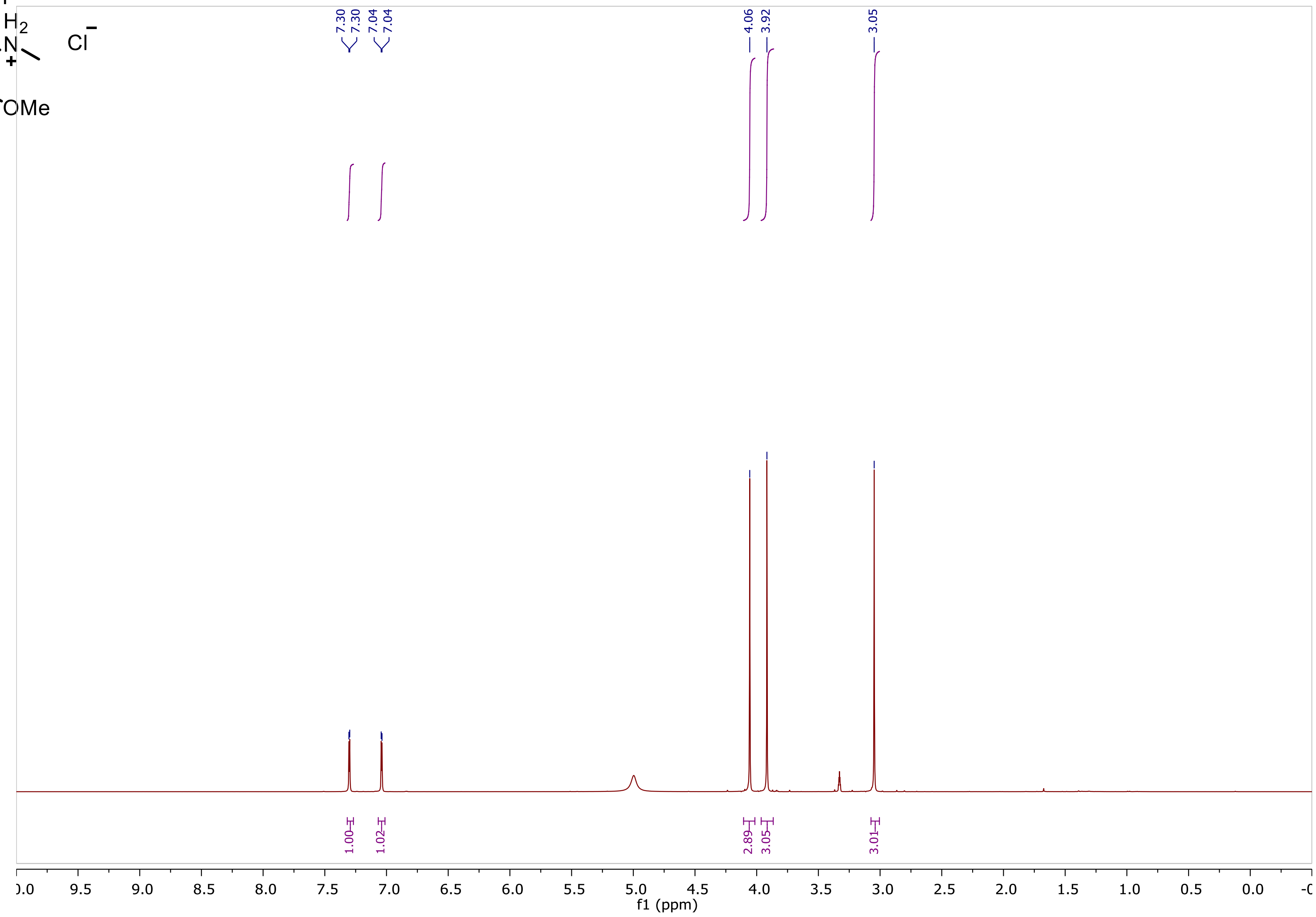

^{13}C NMR spectrum of 3,5-dimethoxy-2-(methylamino)benzoic acid hydrochloride **4b** in MeOD-d_4

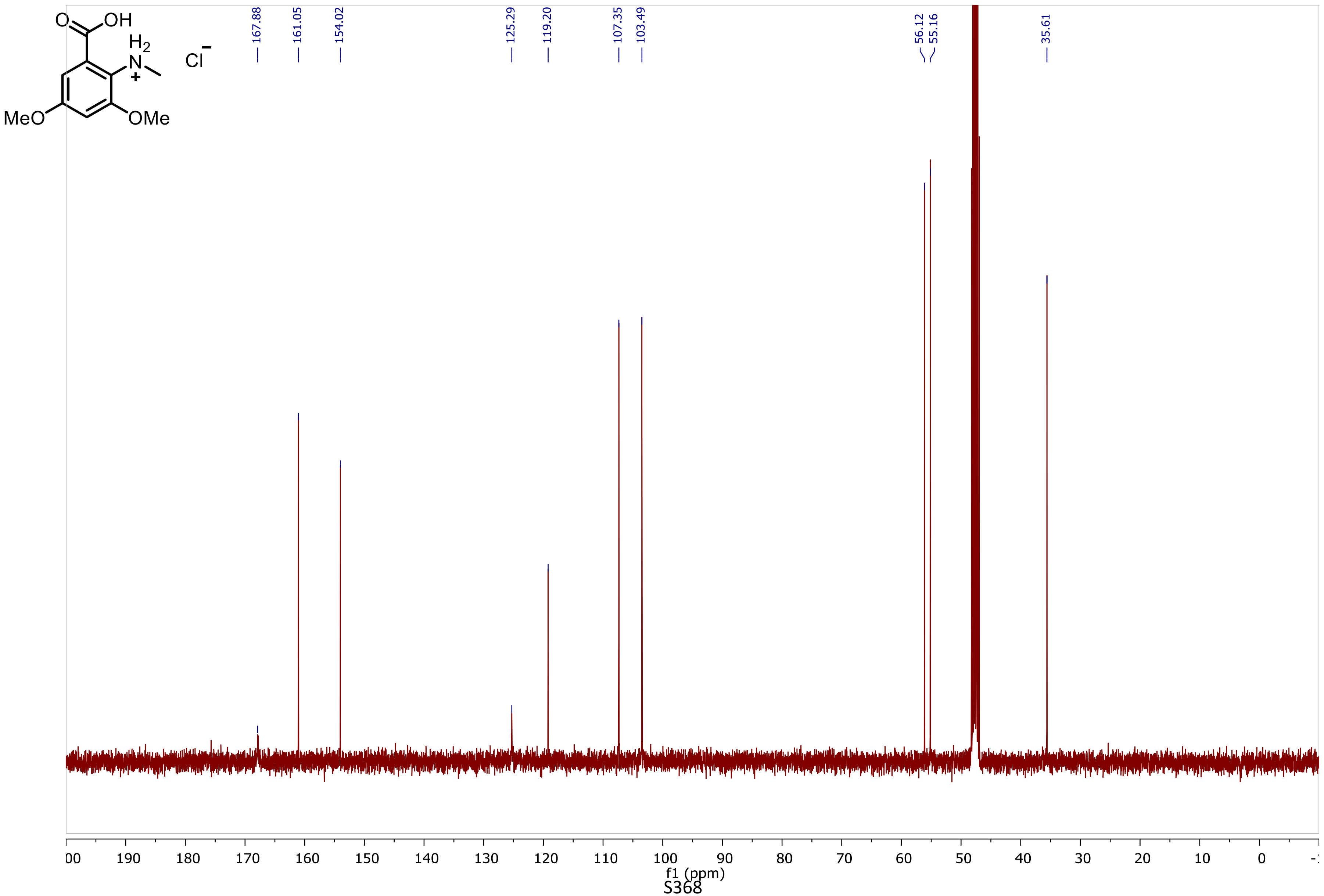

^{1}H NMR spectrum of 6-chloro-3-methoxy-2-(methylamino)benzoic acid hydrochloride **4c** in MeOD-d_4

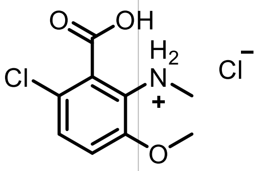

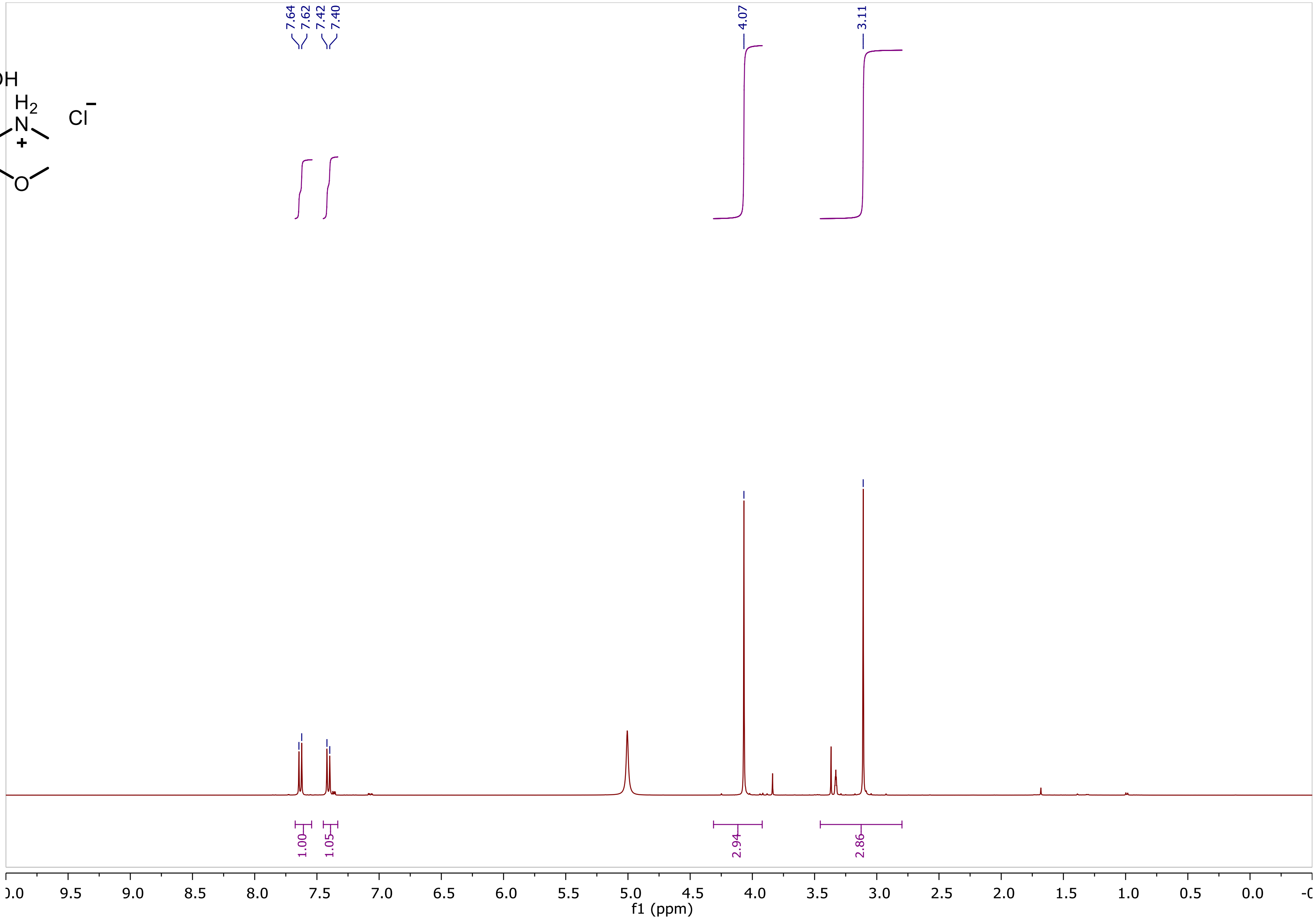

^{13}C NMR spectrum of 6-chloro-3-methoxy-2-(methylamino)benzoic acid hydrochloride **4c** in MeOD-d_4

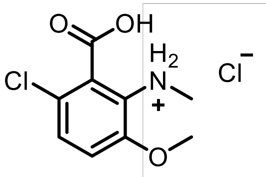

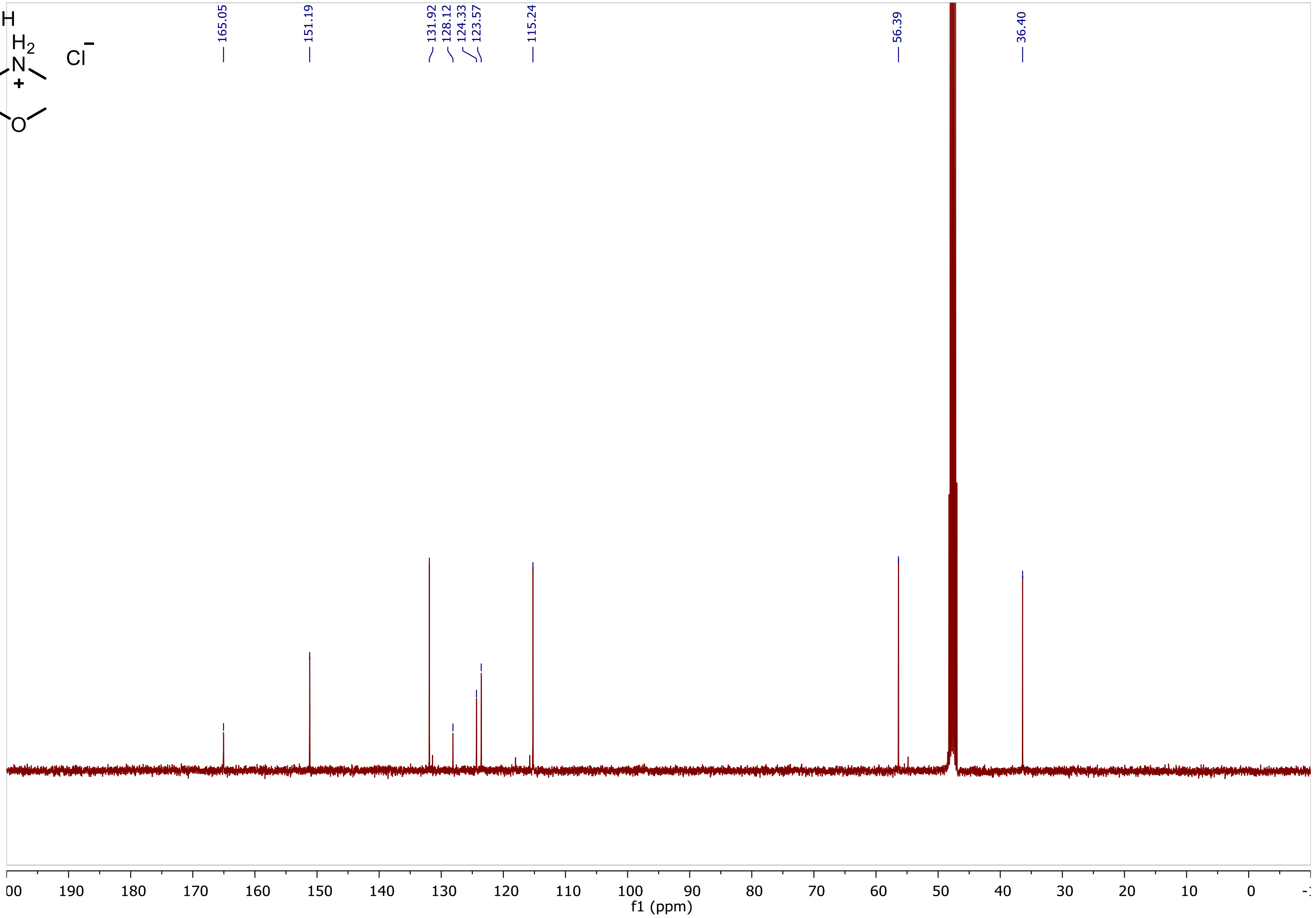

^{1}H NMR spectrum of 3-chloro-2-methyl-6-(methylamino)benzoic acid hydrochloride **4g** in MeOD-d_4

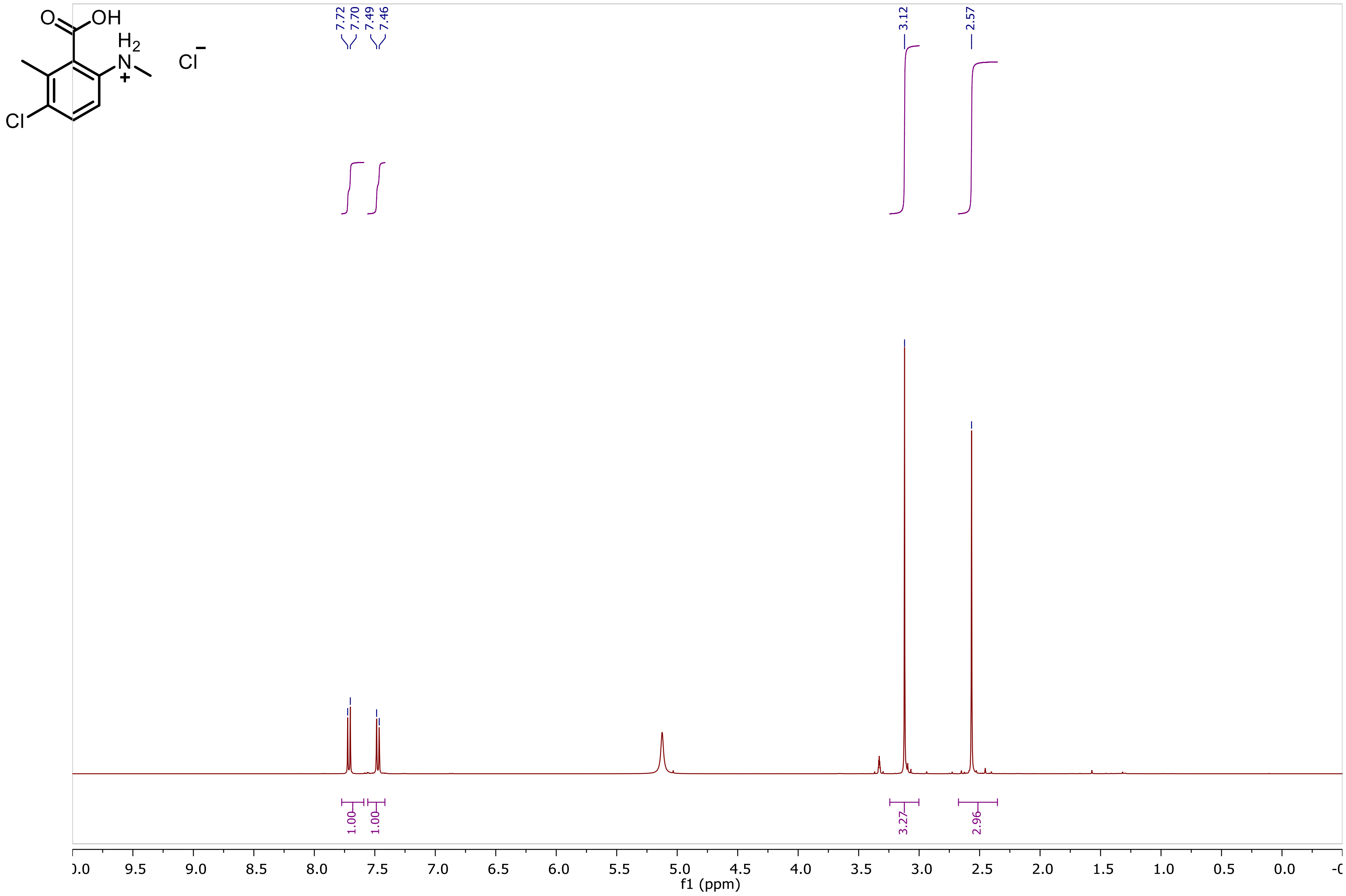

^{13}C NMR spectrum of 3-chloro-2-methyl-6-(methylamino)benzoic acid hydrochloride **4g** in MeOD-d_4

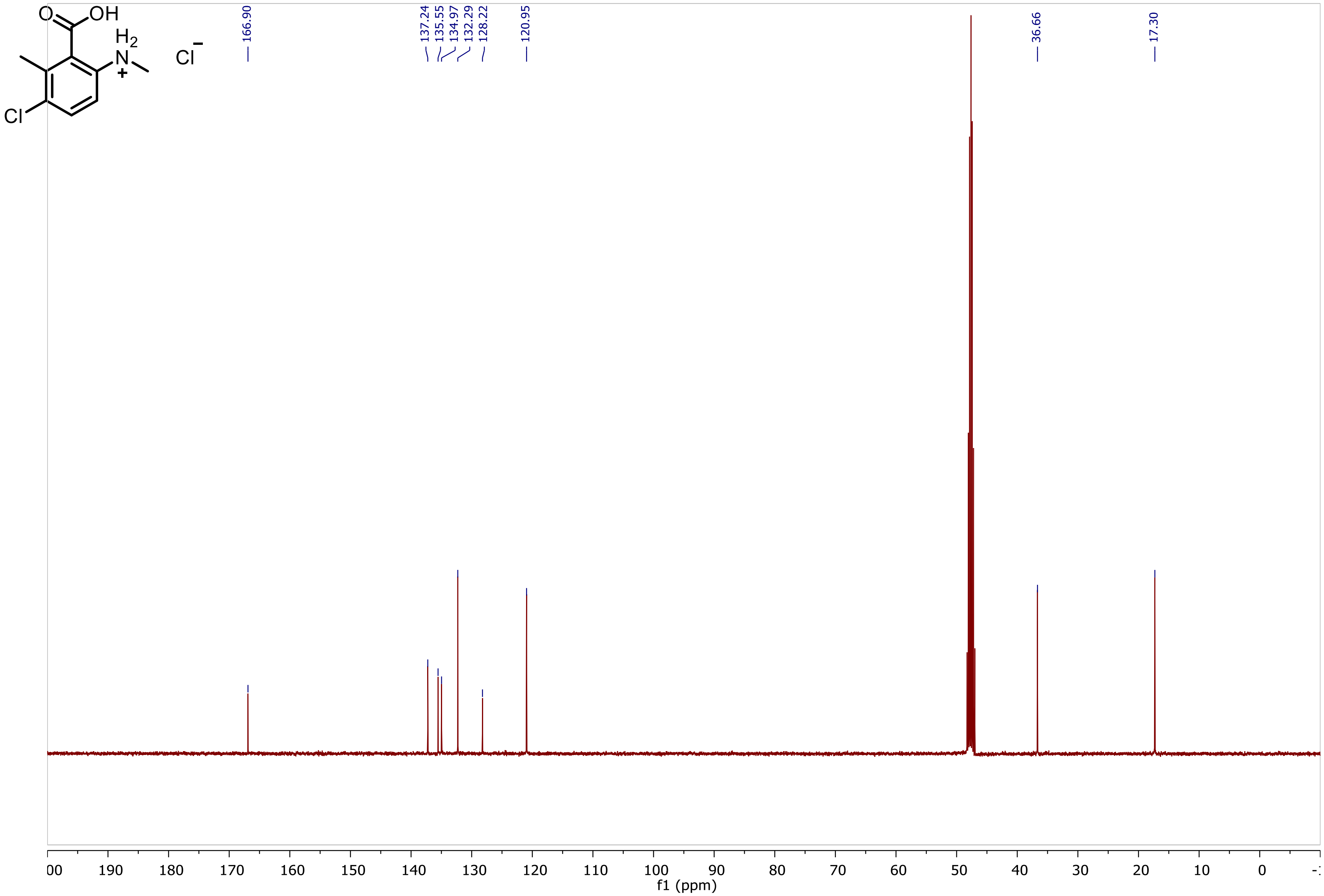

^{1}H NMR spectrum of 4,5-dimethyl-2-(methylamino)benzoic acid hydrochloride **4h** in MeOD-d_4

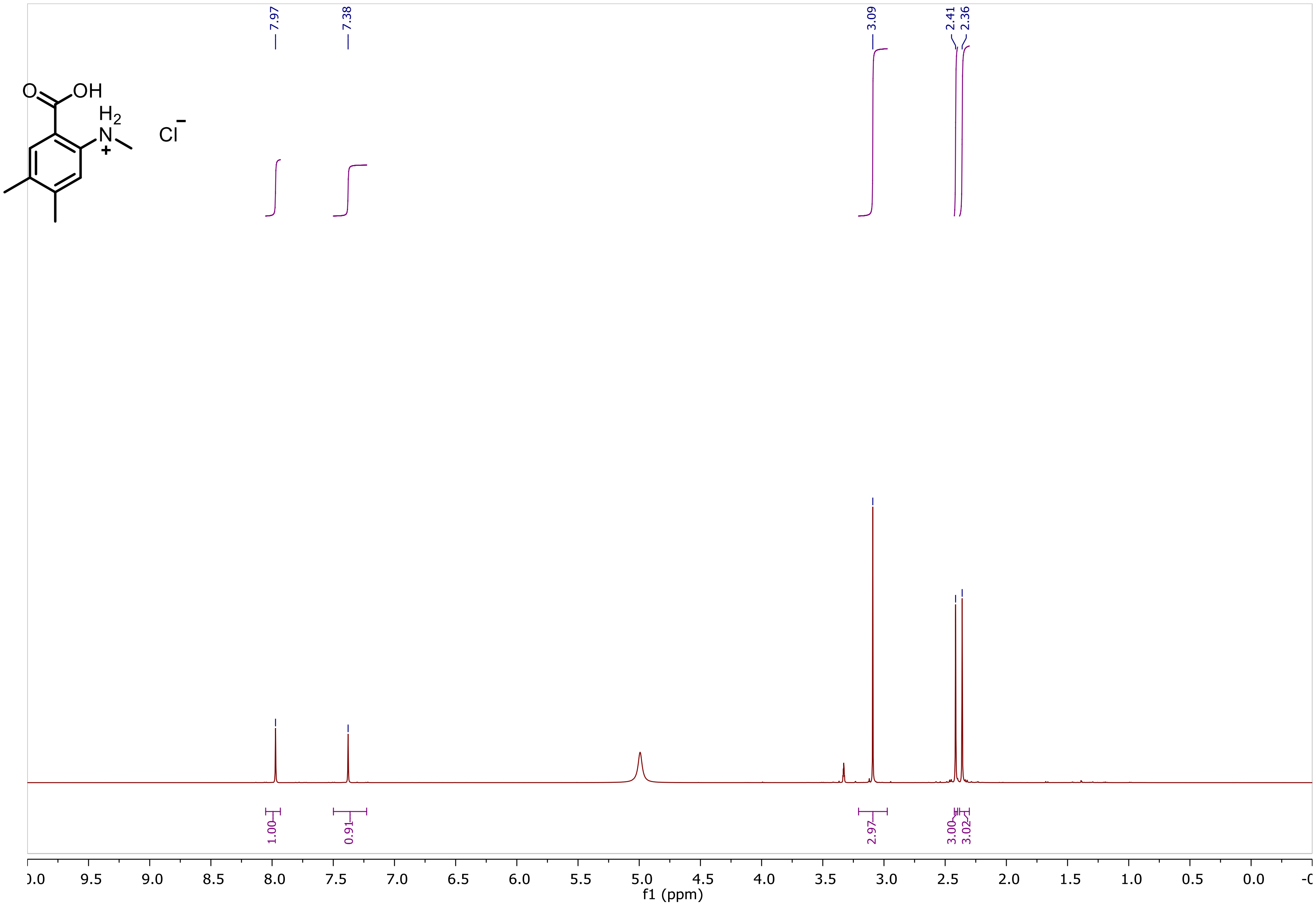

^{13}C NMR spectrum of 4,5-dimethyl-2-(methylamino)benzoic acid hydrochloride **4h** in MeOD-d_4

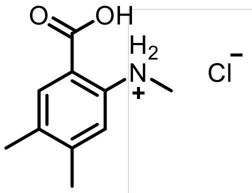

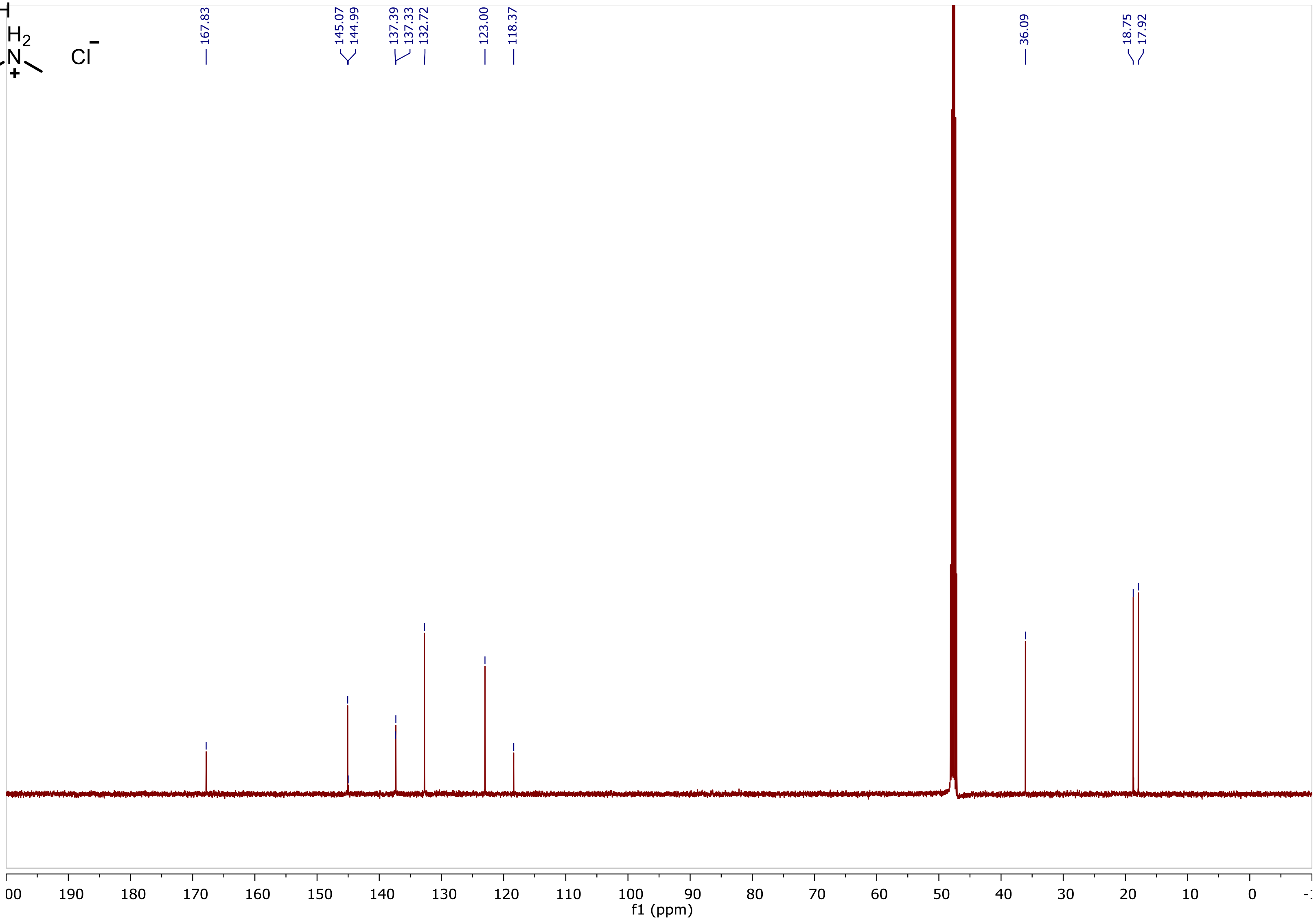

^{1}H NMR spectrum of 3,5-dimethyl-2-(methylamino)benzoic acid hydrochloride **4i** in MeOD-d_4

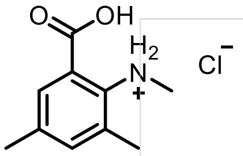

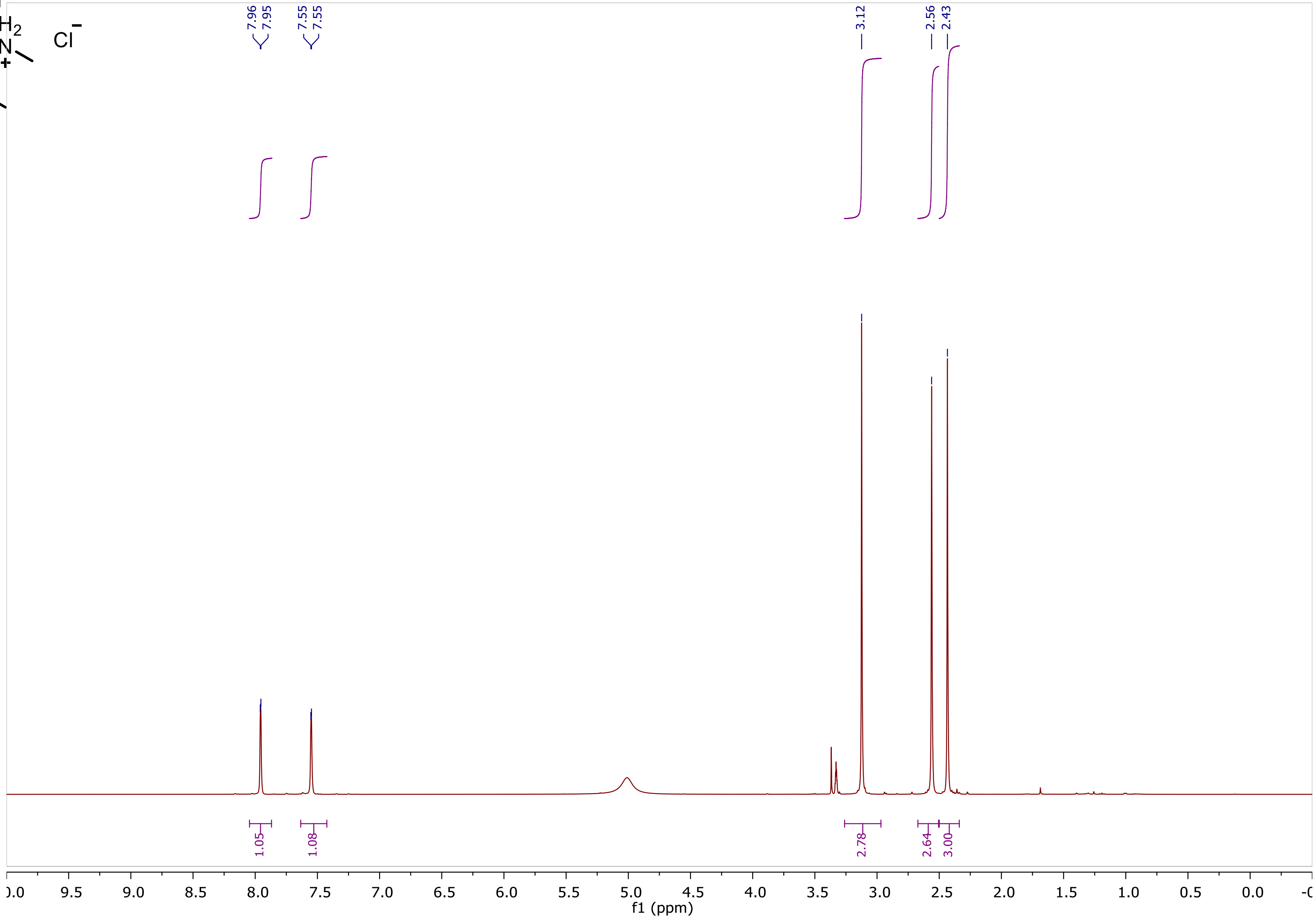

^{13}C NMR spectrum of 3,5-dimethyl-2-(methylamino)benzoic acid hydrochloride **4i** in MeOD-d_4

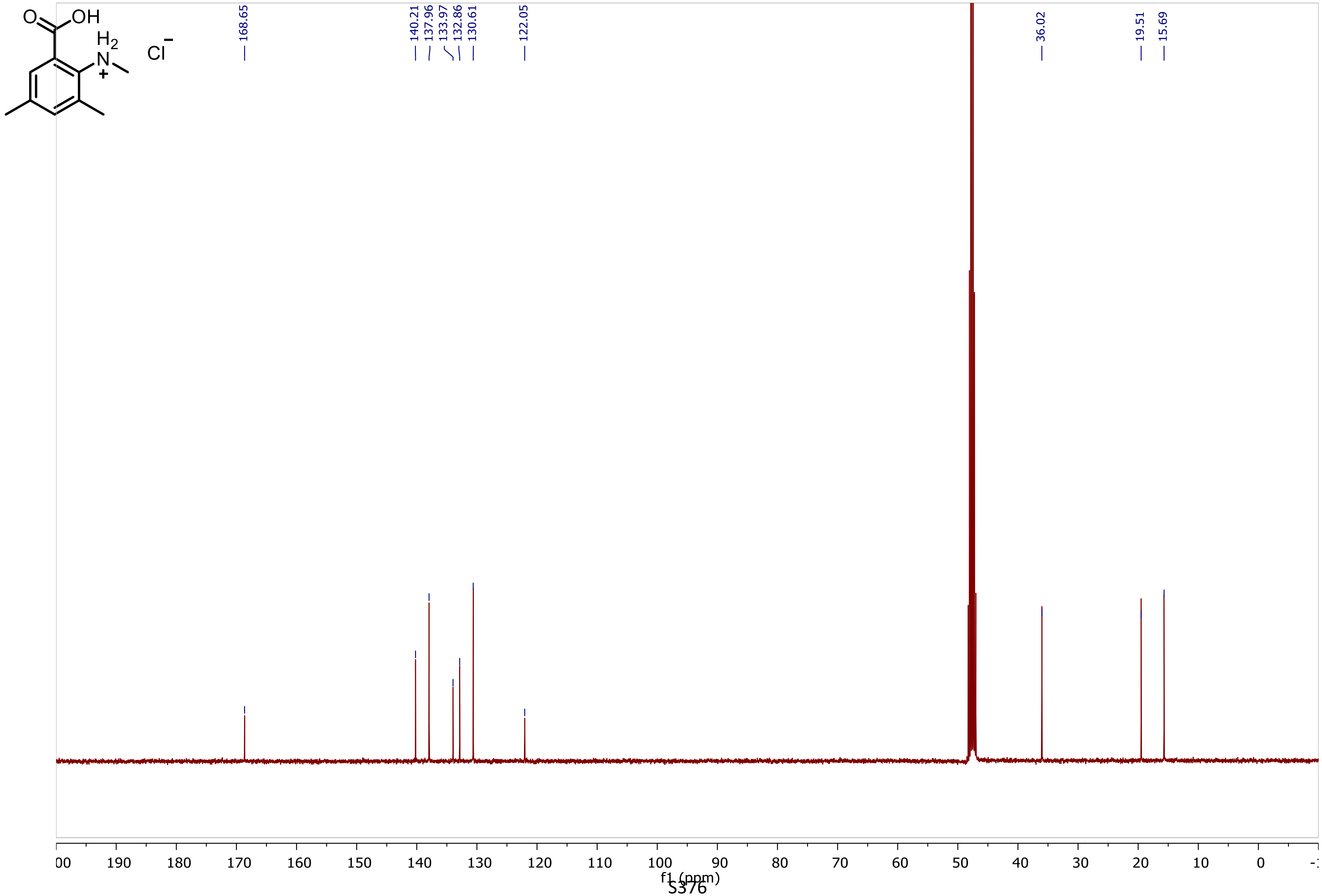

^{1}H NMR spectrum of 2-oxindole **6a** in $CDCl_3$

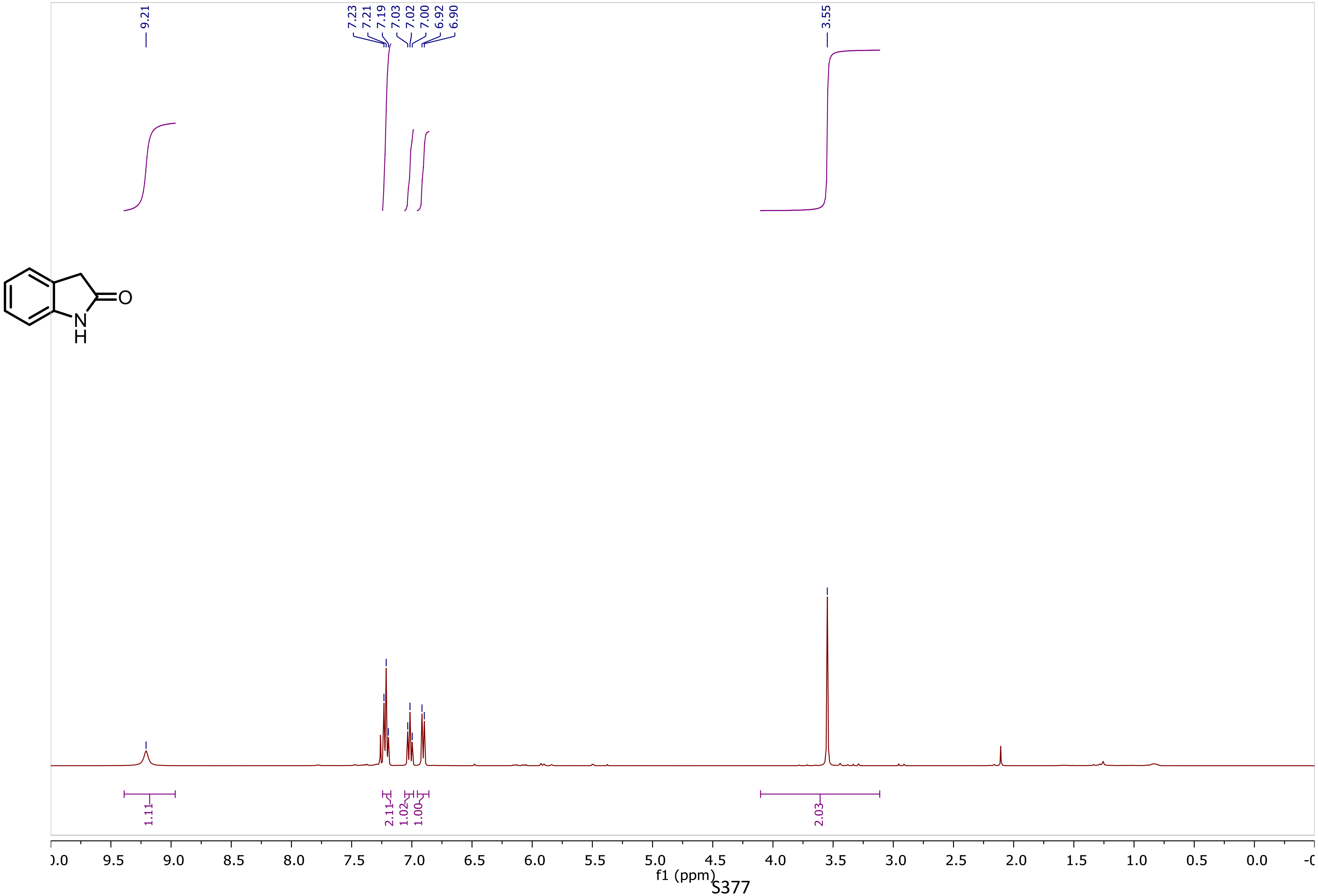

^{13}C NMR spectrum of 2-oxindole **6a** in $CDCl_3$

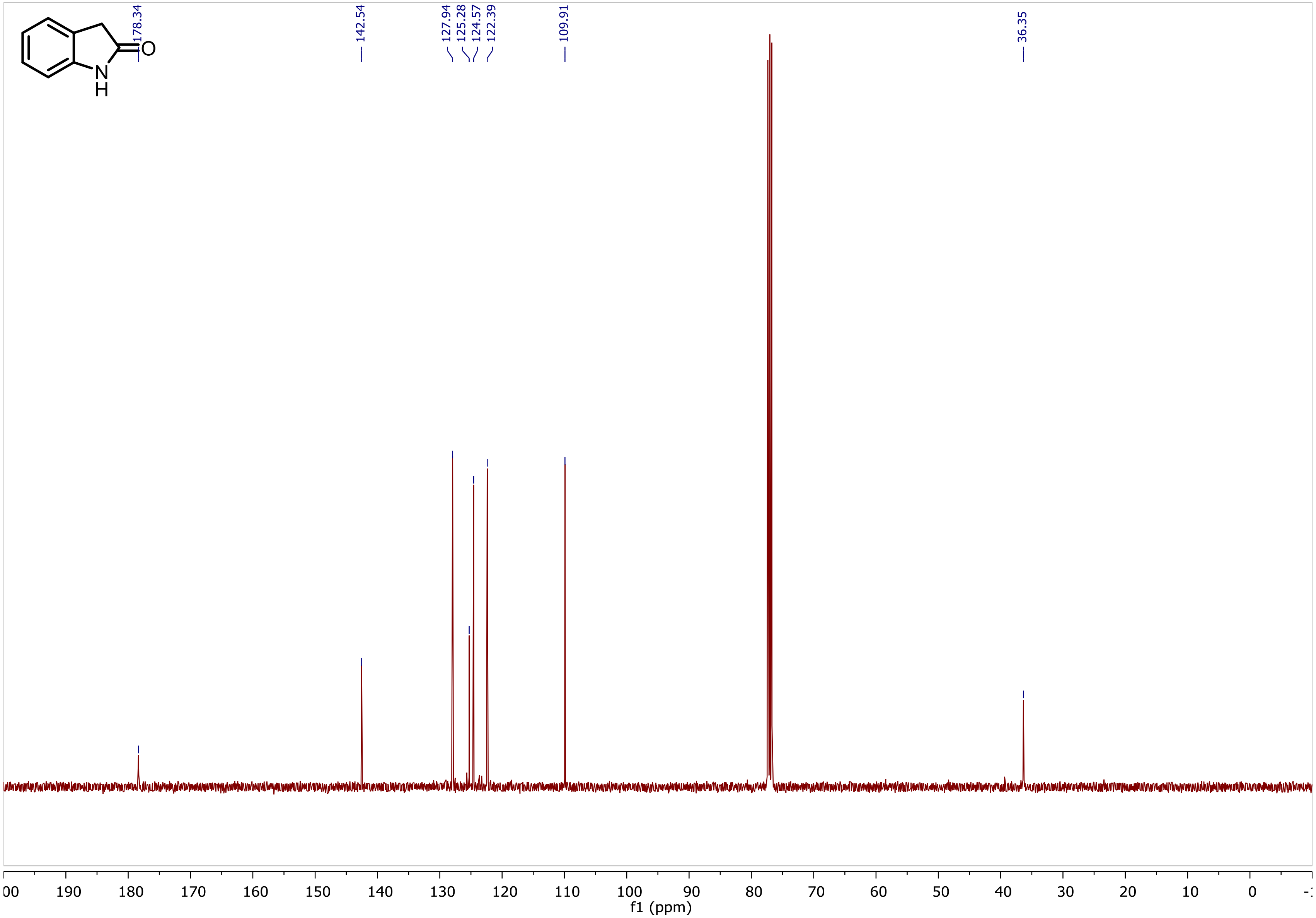

^{1}H NMR spectrum of 3-methyl-2-oxindole **6b** in $CDCl_3$

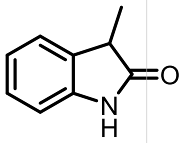

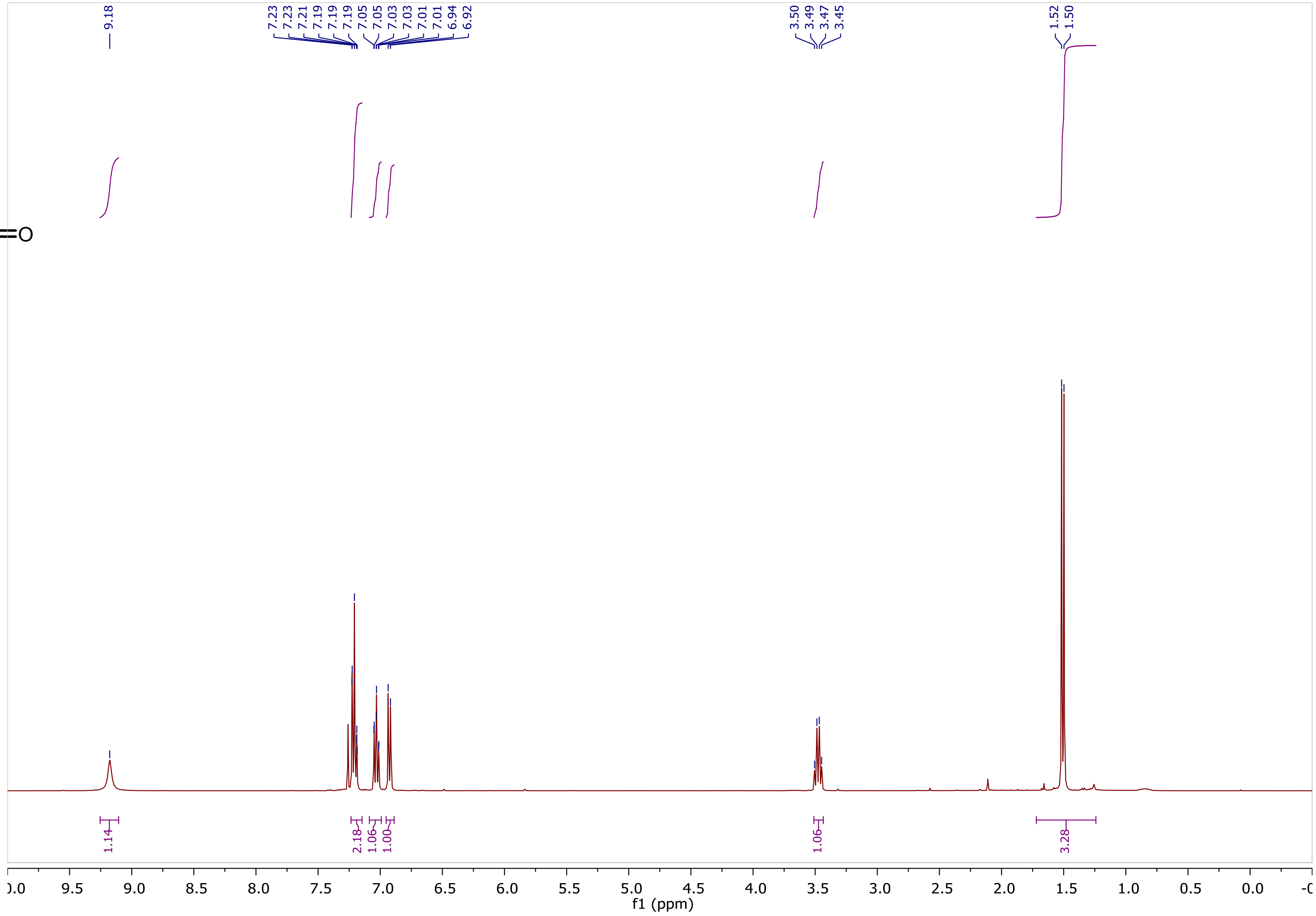

^{13}C NMR spectrum of 3-methyl-2-oxindole **6b** in $CDCl_3$

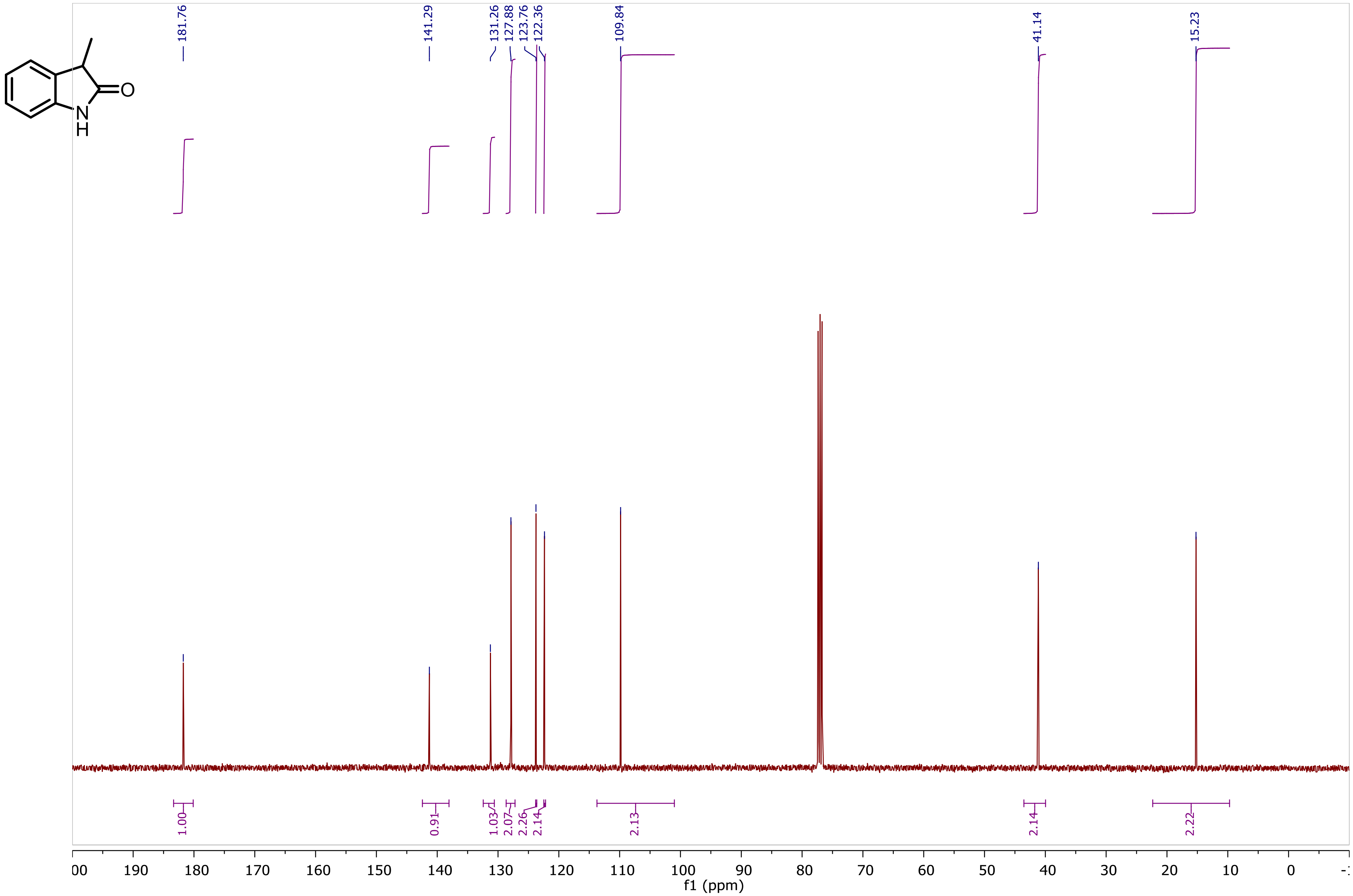

^{1}H NMR spectrum of 3,3-dimethyl-2-oxindole **6c** in $CDCl_3$

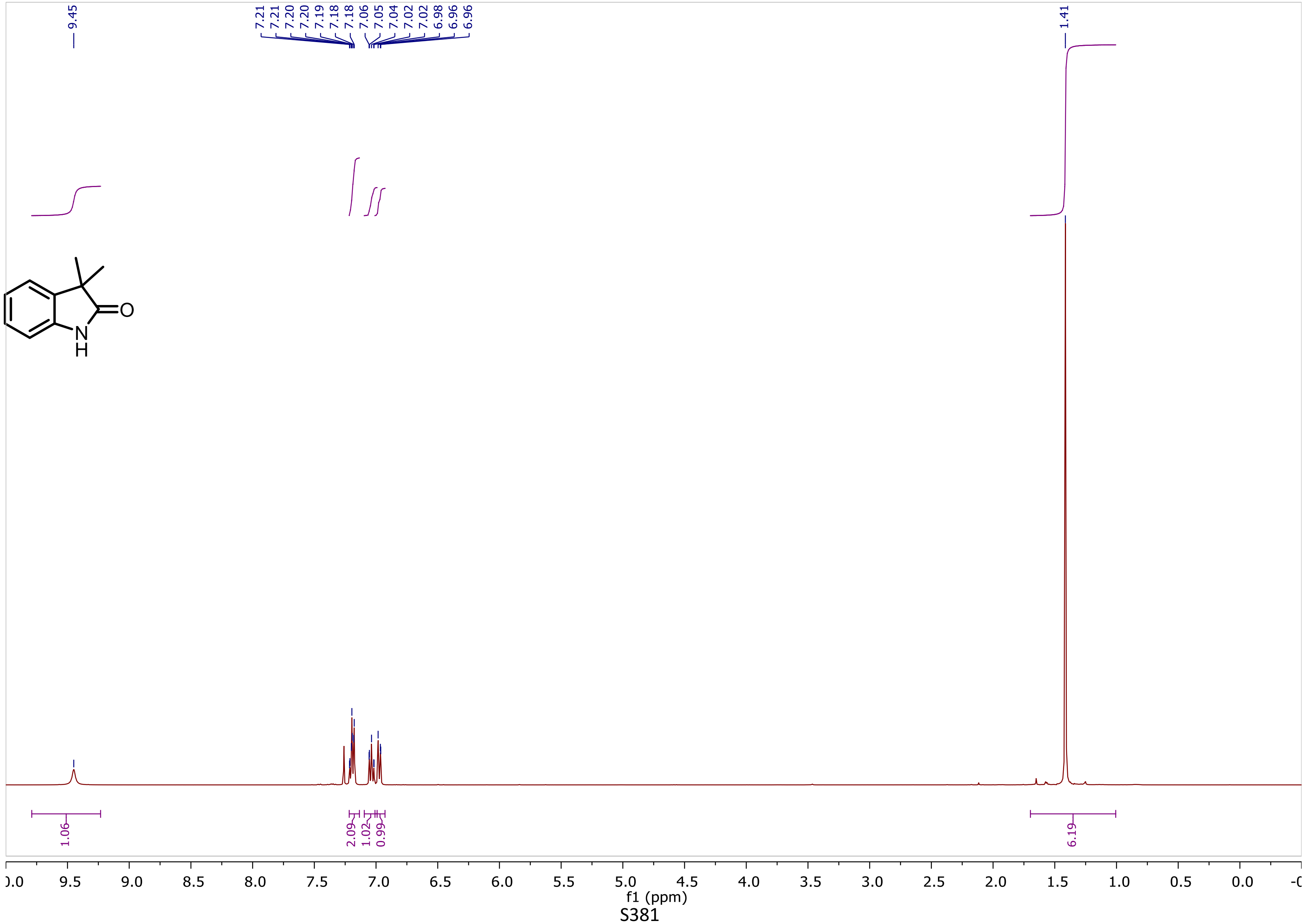

^{13}C NMR spectrum of 3,3-dimethyl-2-oxindole **6c** in $CDCl_3$

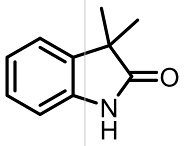

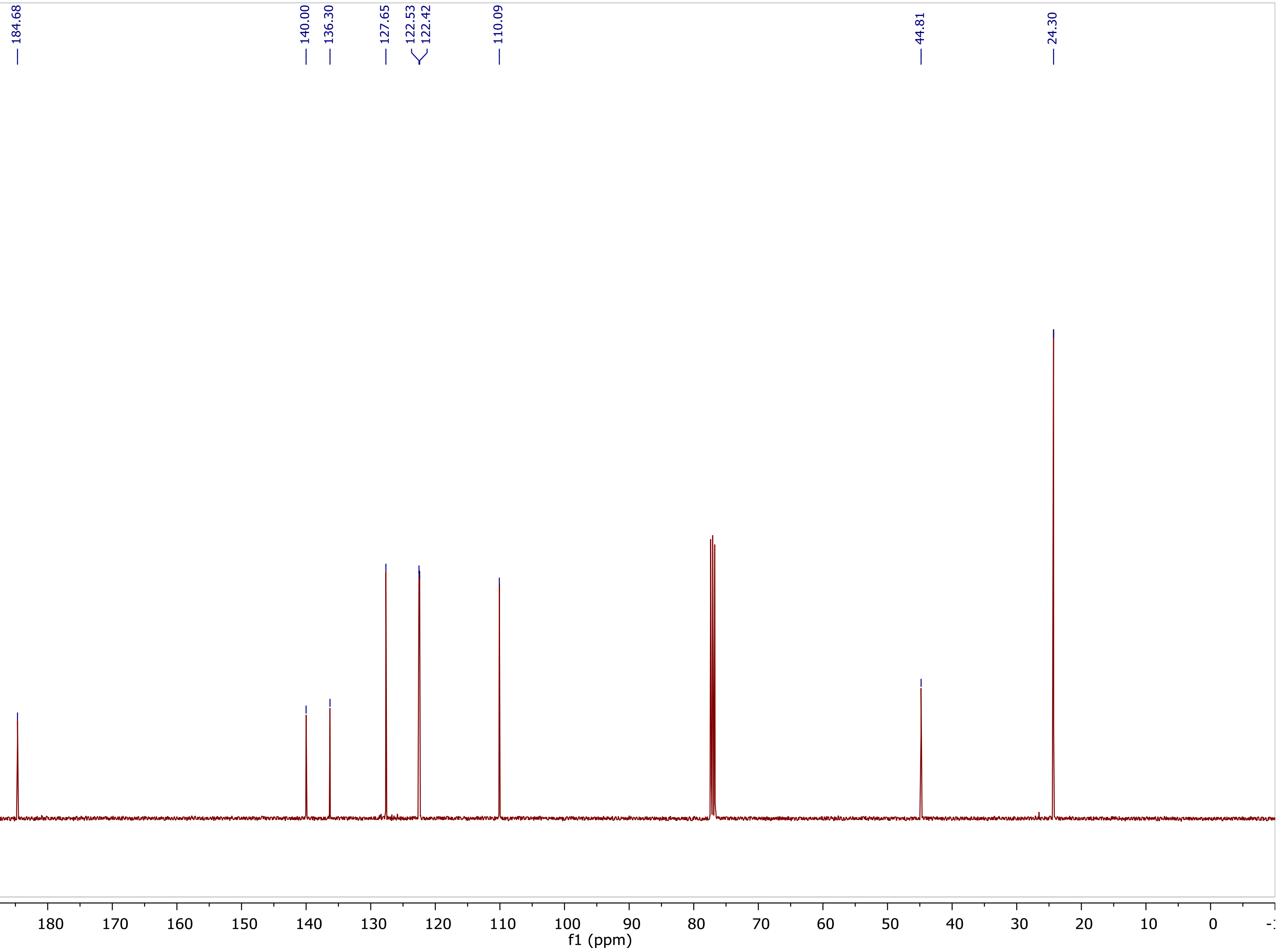

^{1}H NMR spectrum of spiro[cyclopropane-1,3'-indolin]-2'-one **6d** in $CDCl_3$

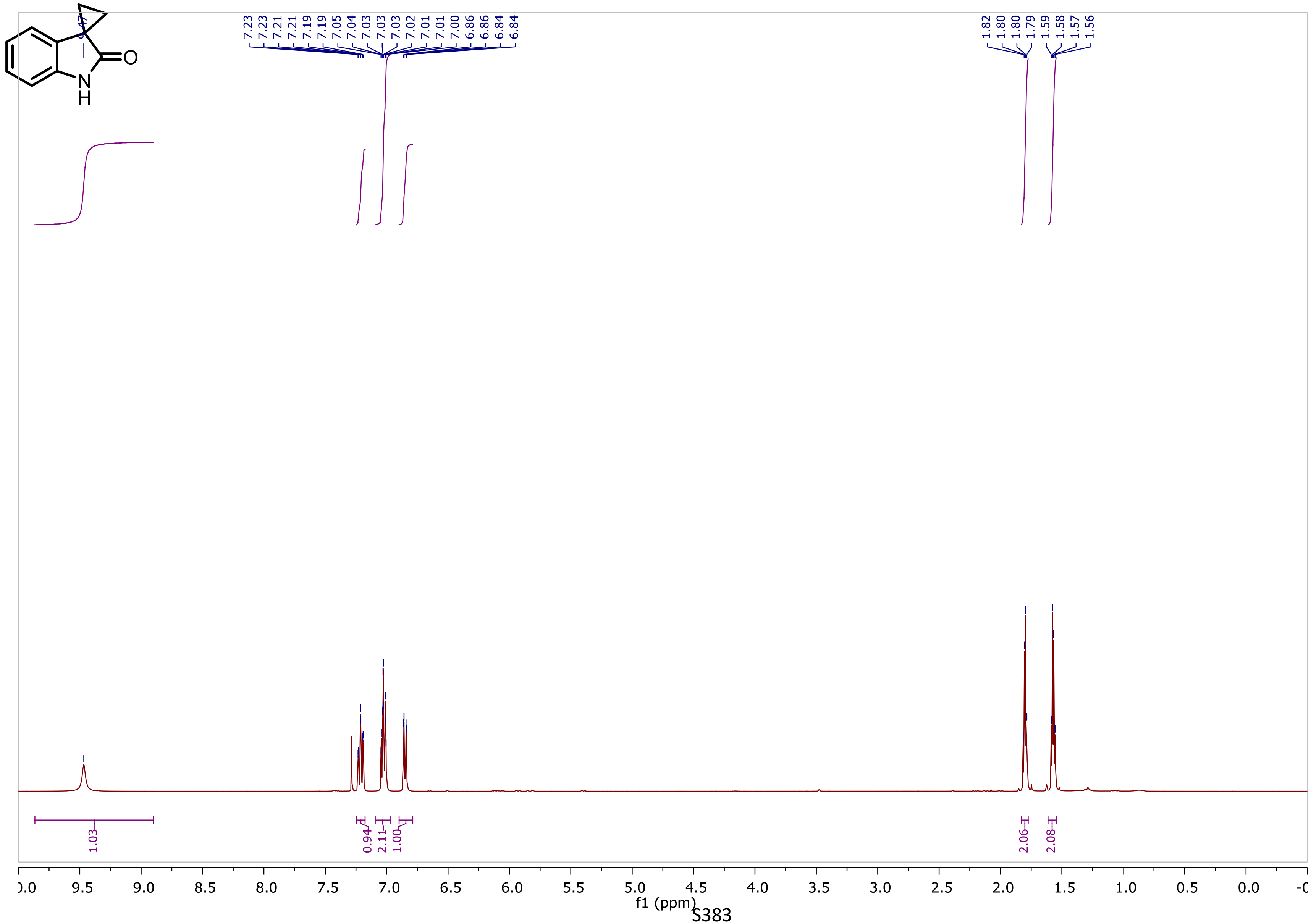

^{13}C NMR spectrum of spiro[cyclopropane-1,3'-indolin]-2'-one **6d** in $CDCl_3$

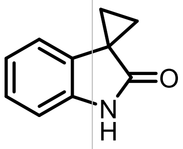

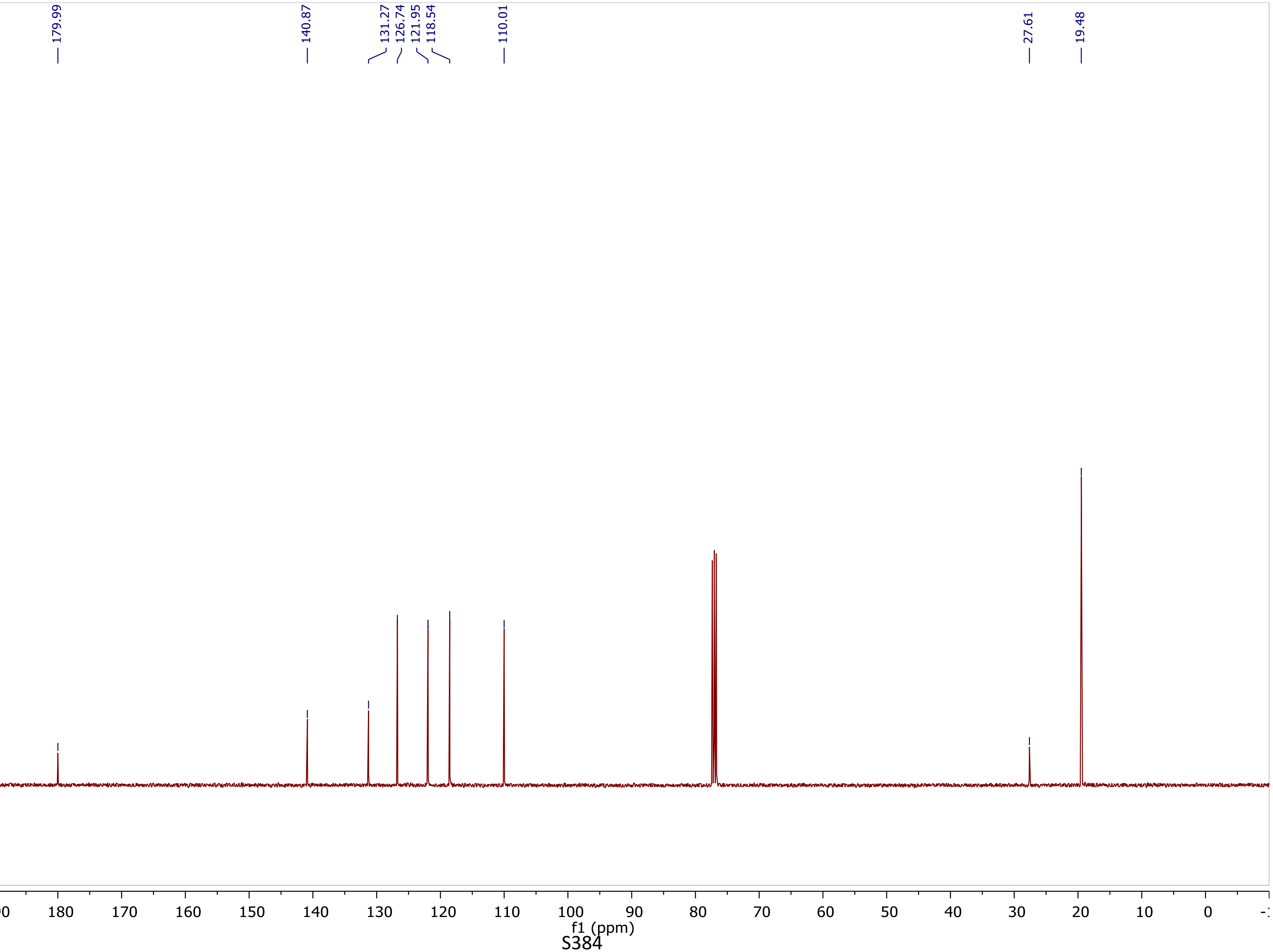

^{1}H NMR spectrum of 4-methyl-2-oxindole **6f** in $CDCl_3$

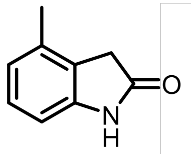

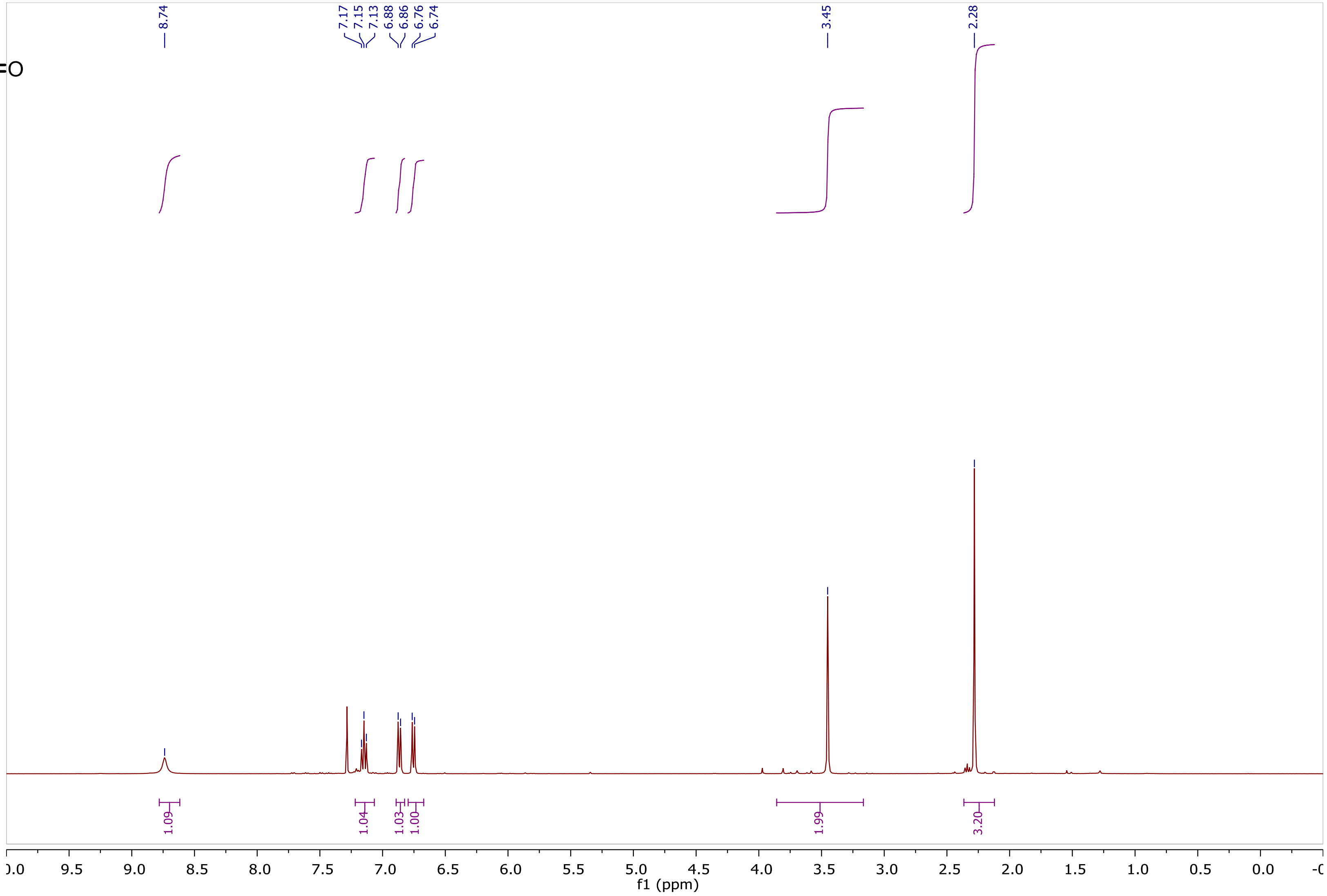

^{13}C NMR spectrum of 4-methyl-2-oxindole **6f** in $CDCl_3$

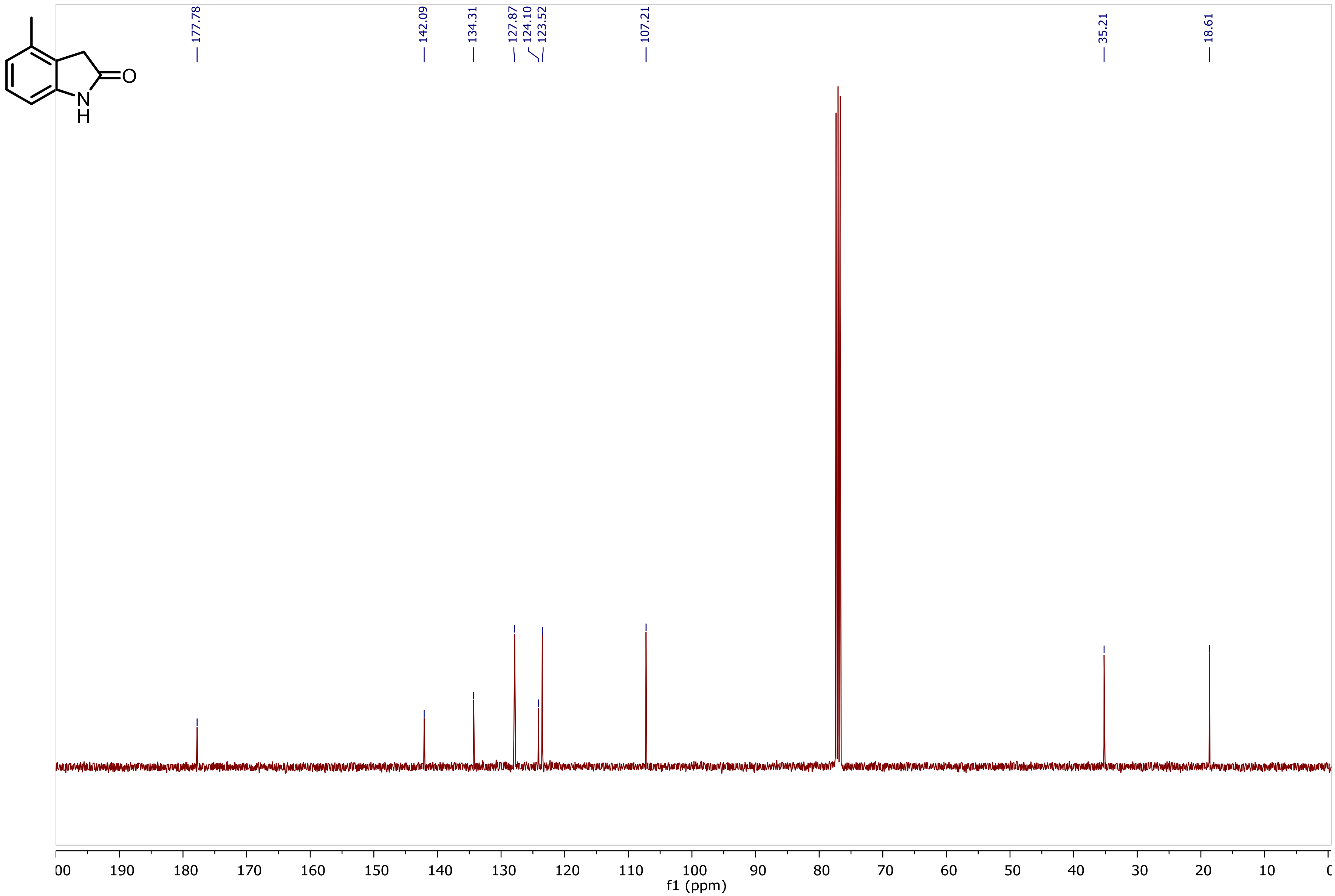

^{1}H NMR spectrum of 4-chloro-2-oxindole **6e** in $CDCl_3$

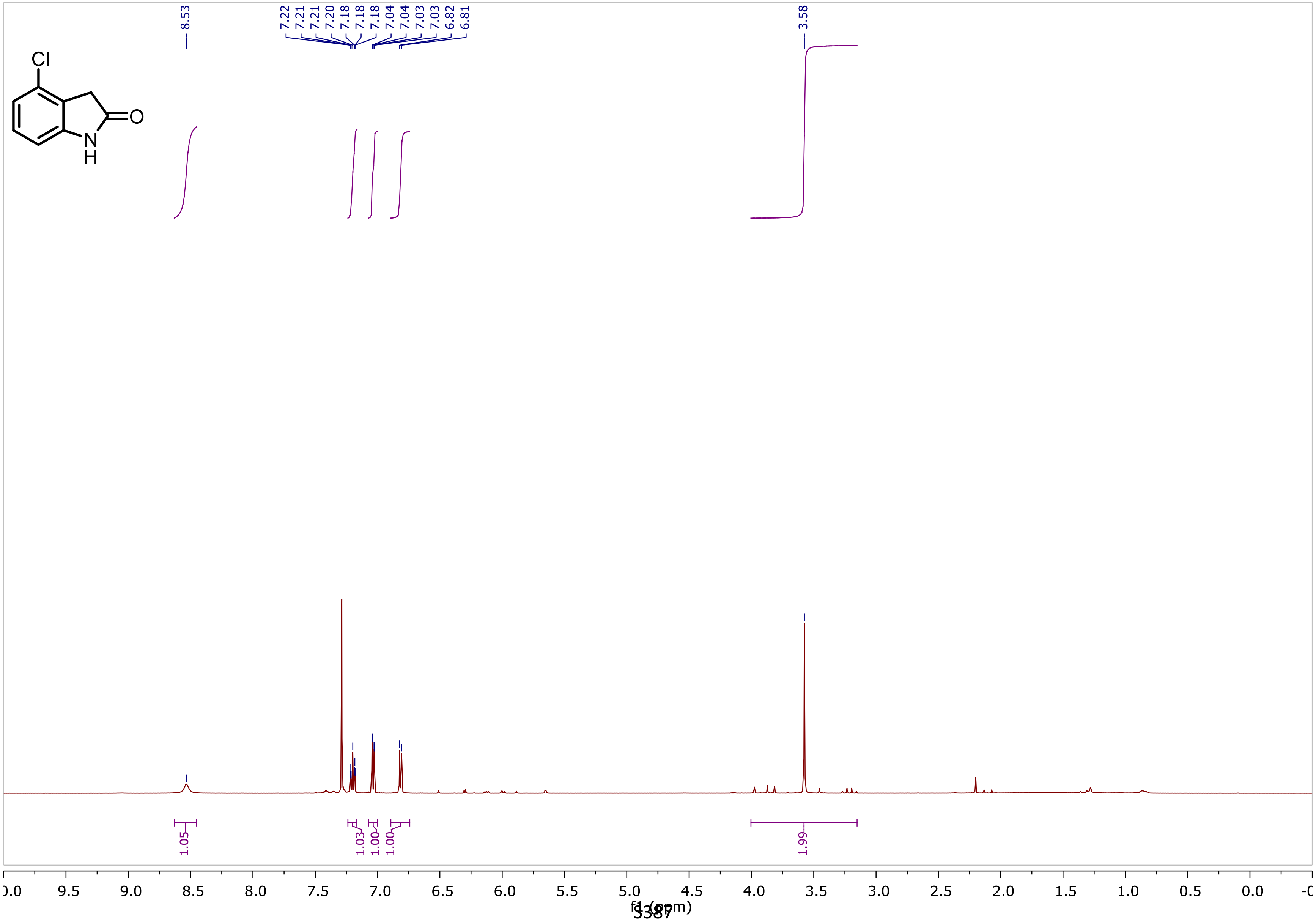

^{13}C NMR spectrum of 4-chloro-2-oxindole **6e** in $CDCl_3$

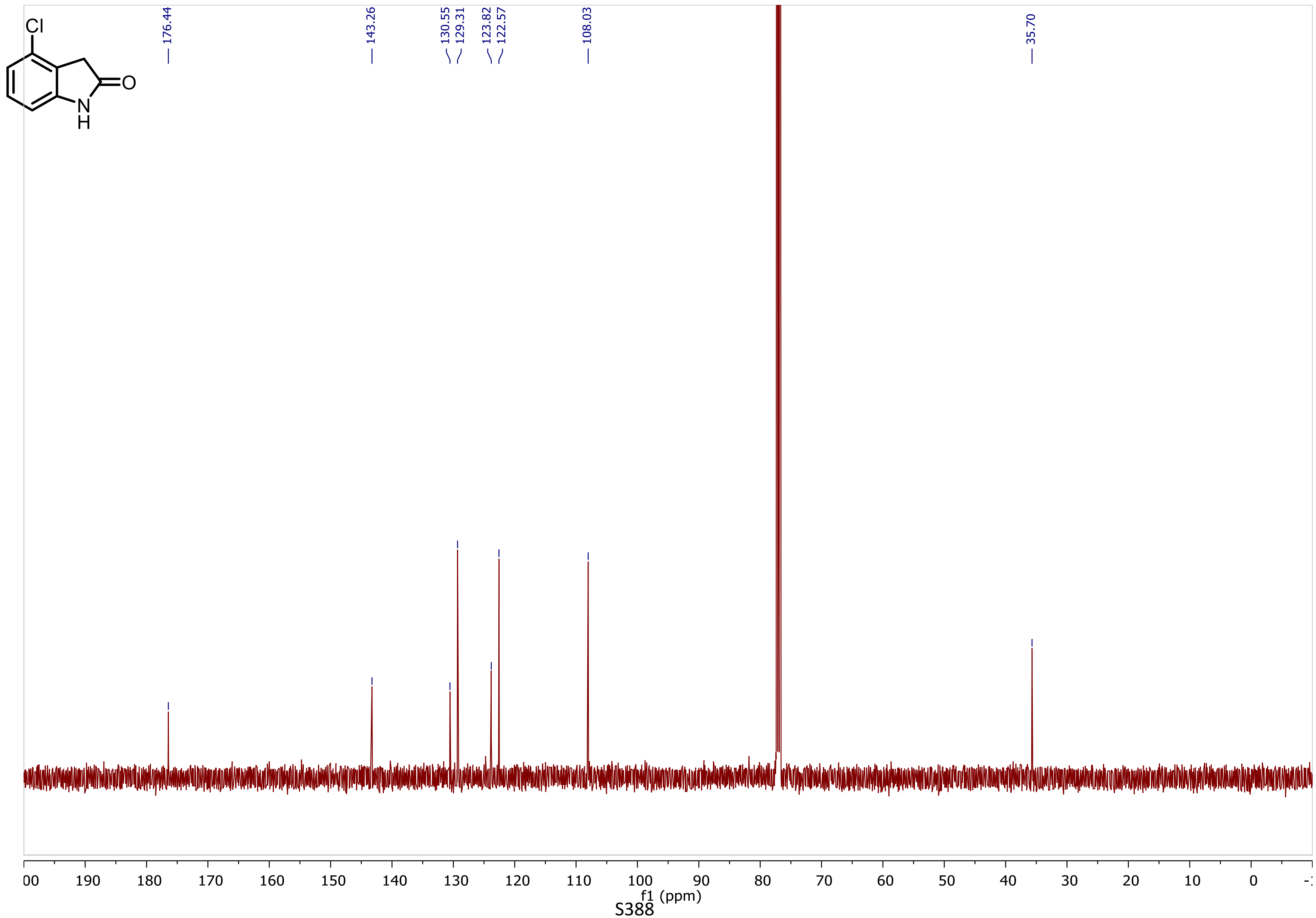

^{1}H NMR spectrum of 4-(trifluoromethyl)-2-oxindole **6g** in MeOD-*d*4

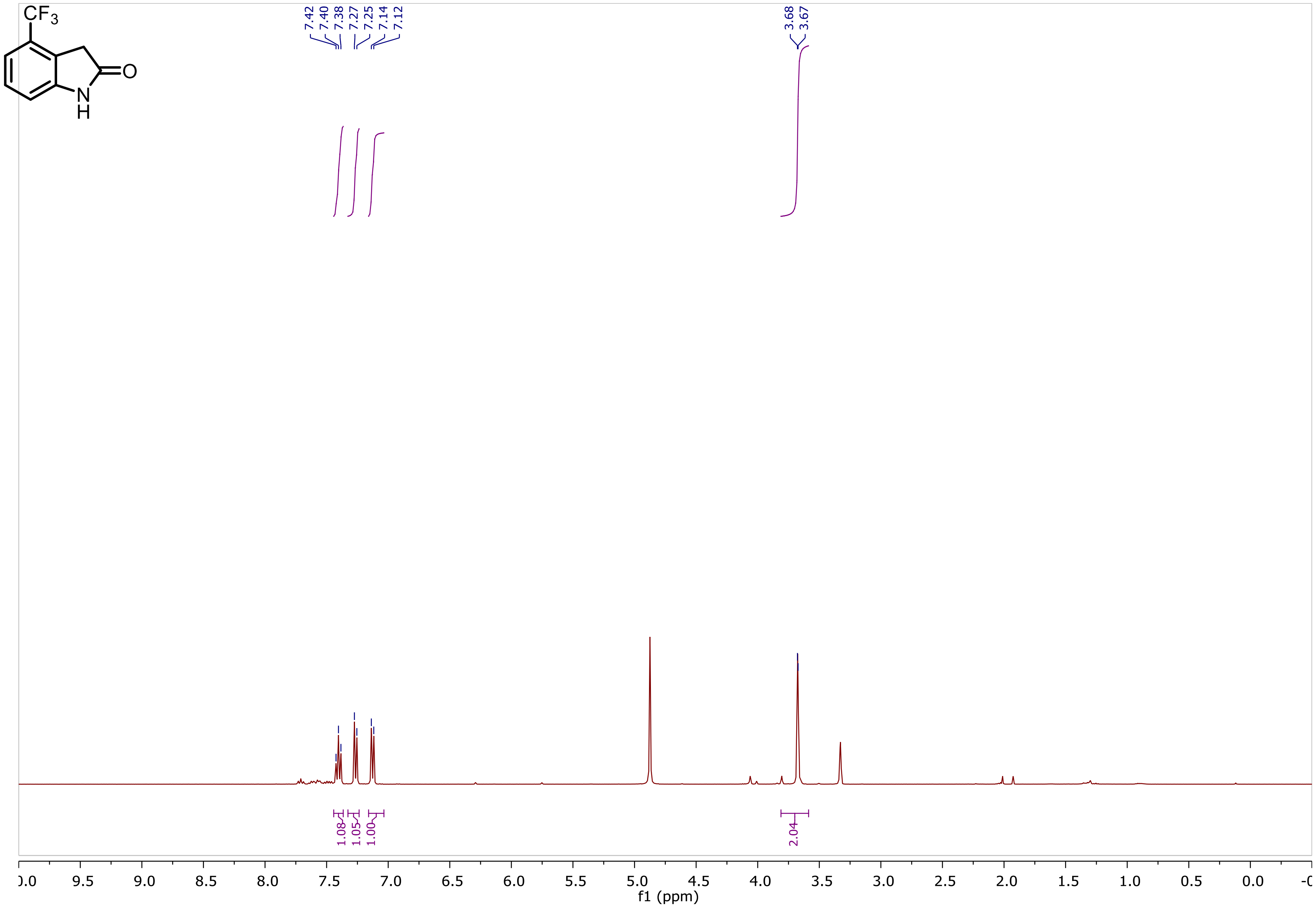

^{13}C NMR spectrum of 4-(trifluoromethyl)-2-oxindole **6g** in MeOD-d_4

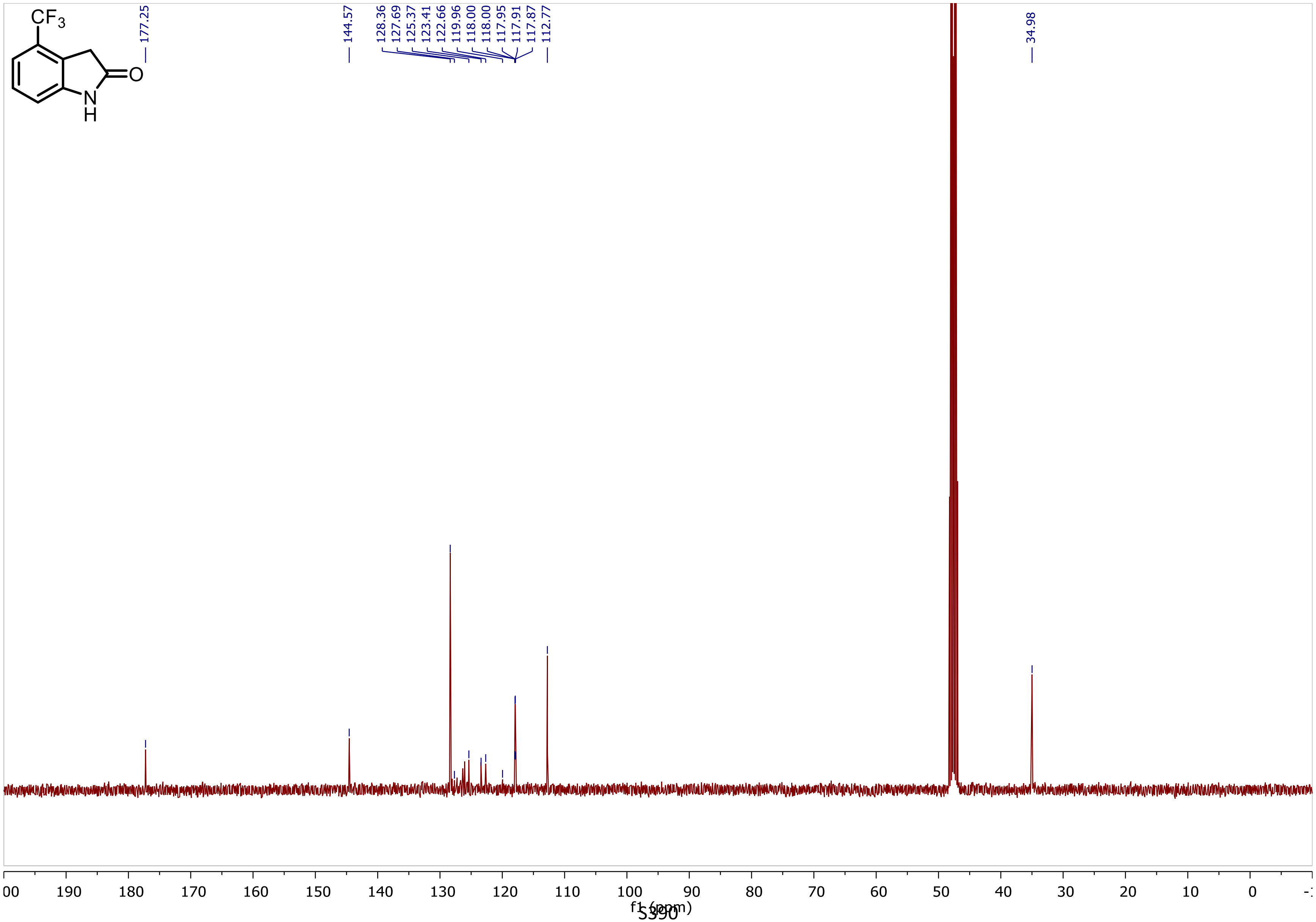

^{19}F NMR spectrum of 4-(trifluoromethyl)-2-oxindole **6g** in MeOD-d_4

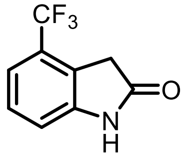

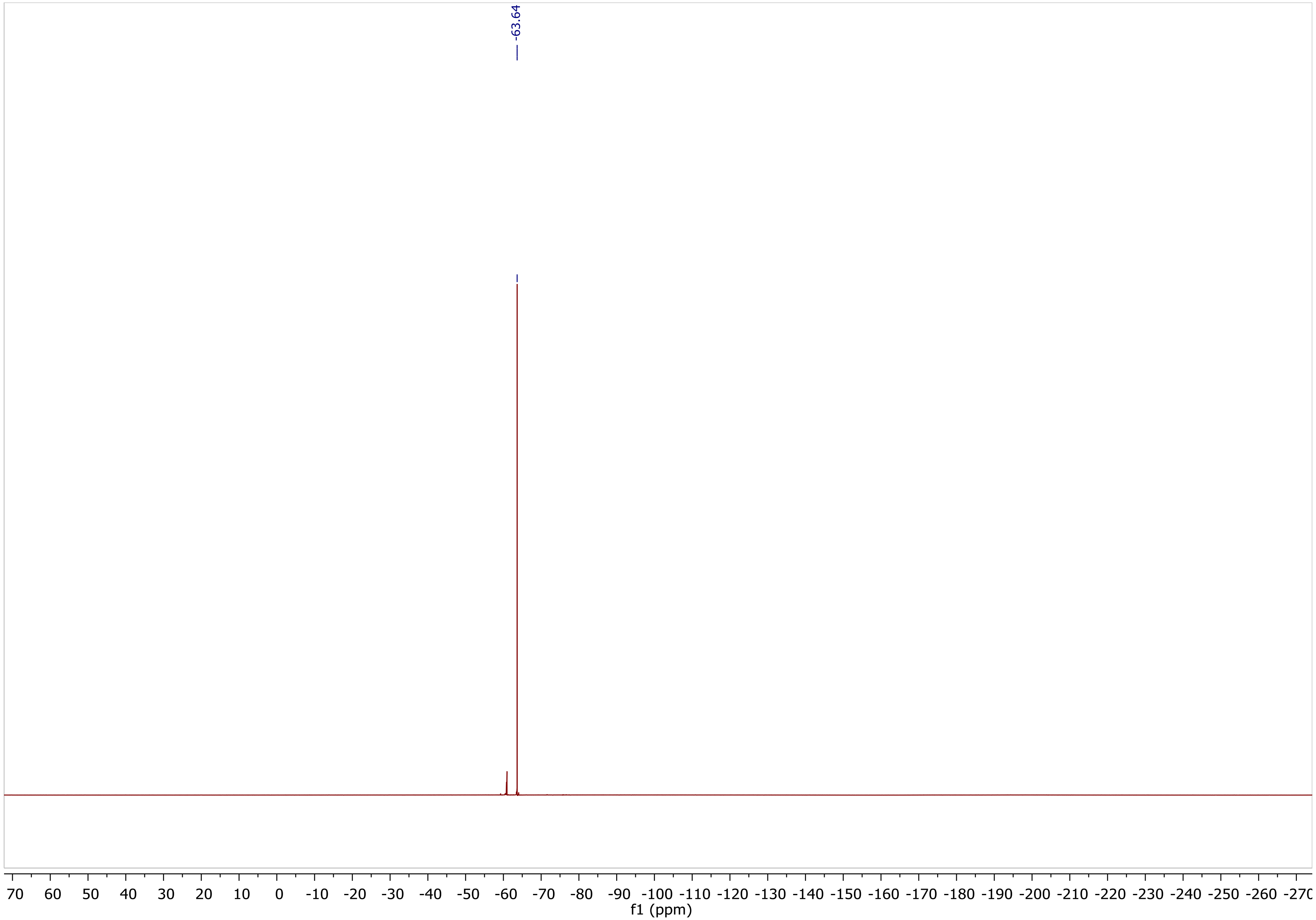

^{1}H NMR spectrum of 5,7-dimethyl-2-oxindole **6h** in $CDCl_3$

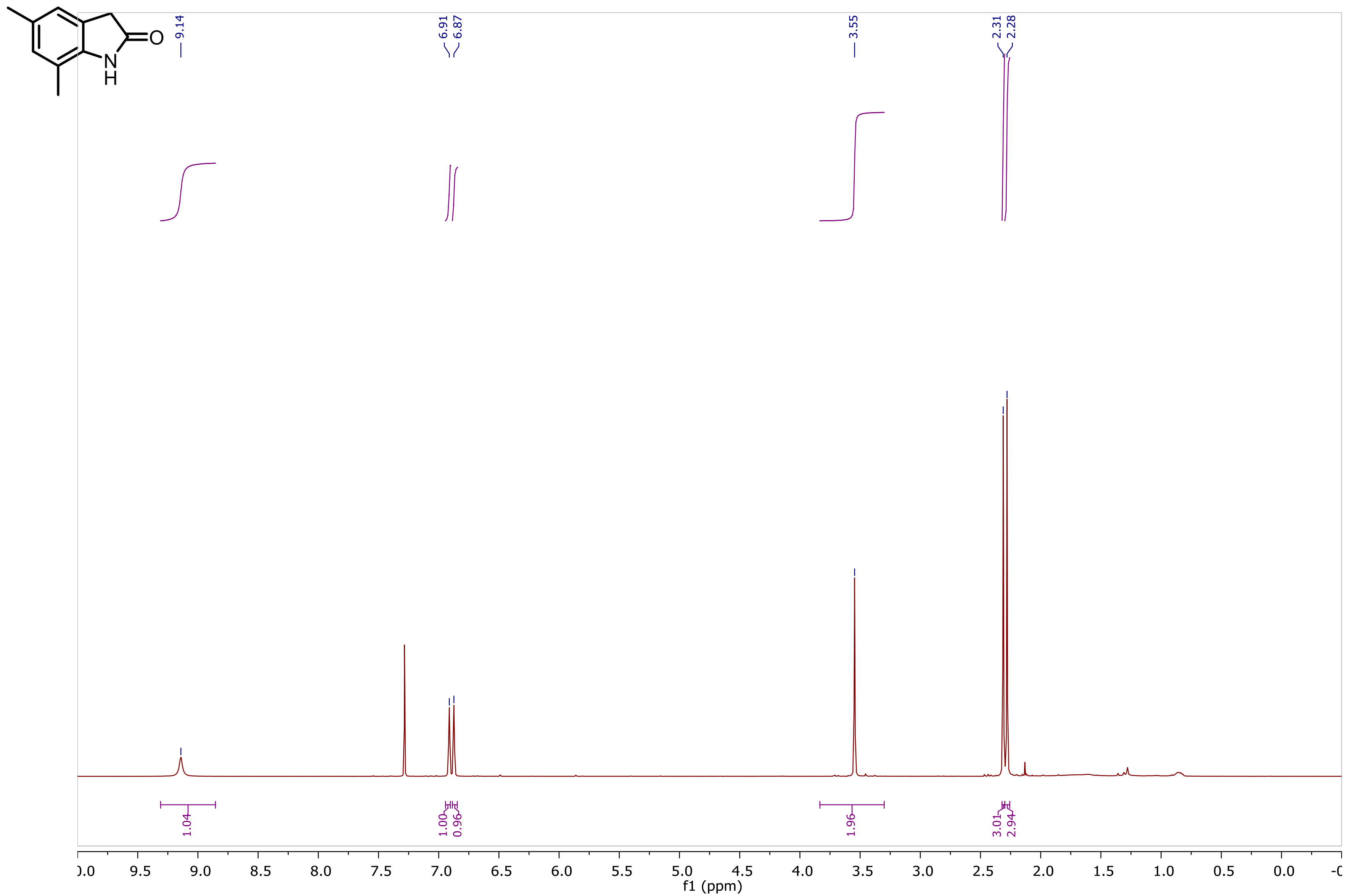

^{13}C NMR spectrum of 5,7-dimethyl-2-oxindole **6h** in $CDCl_3$

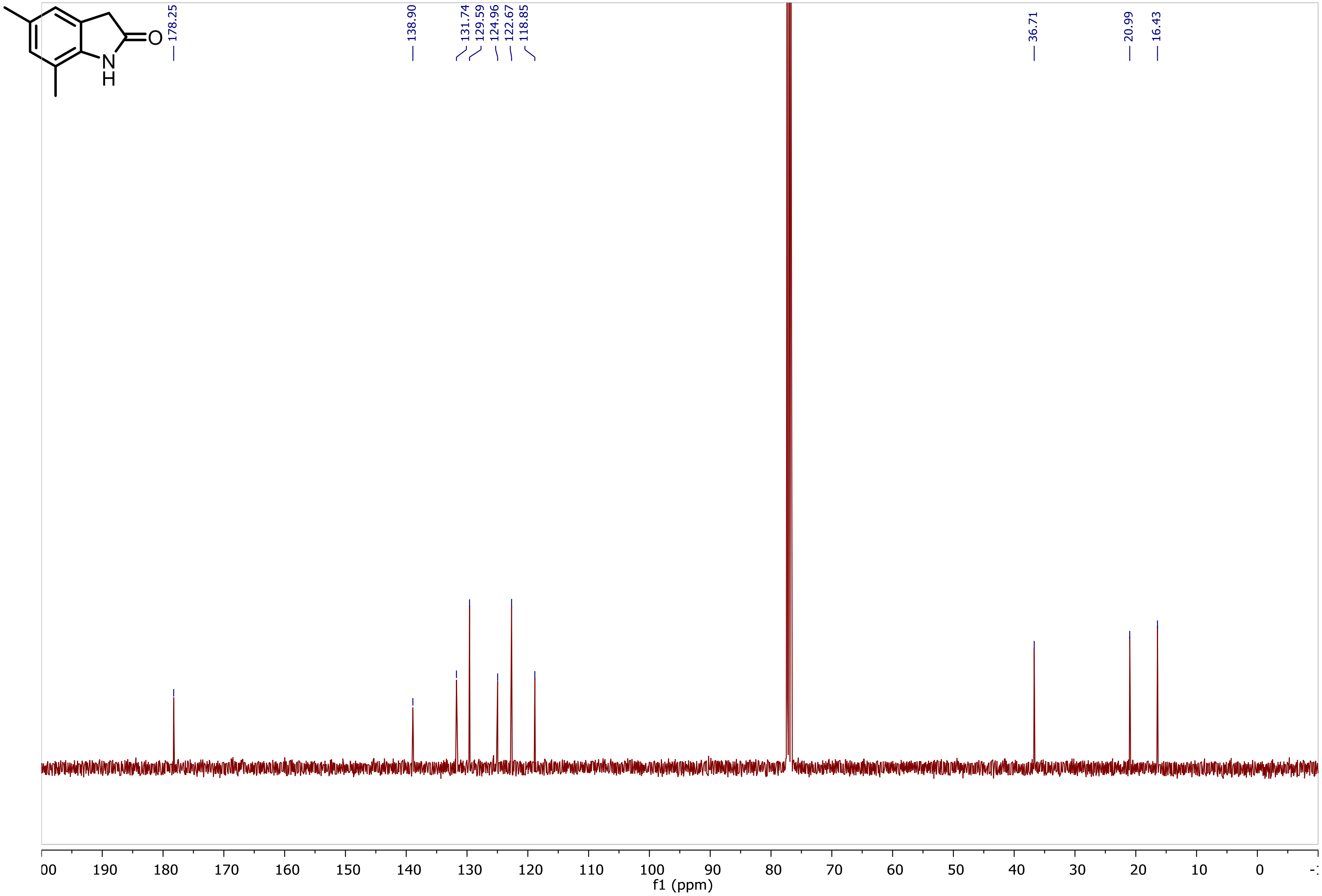

^{1}H NMR spectrum of 5-bromo-2-oxindole **6i** in DMSO-d_6

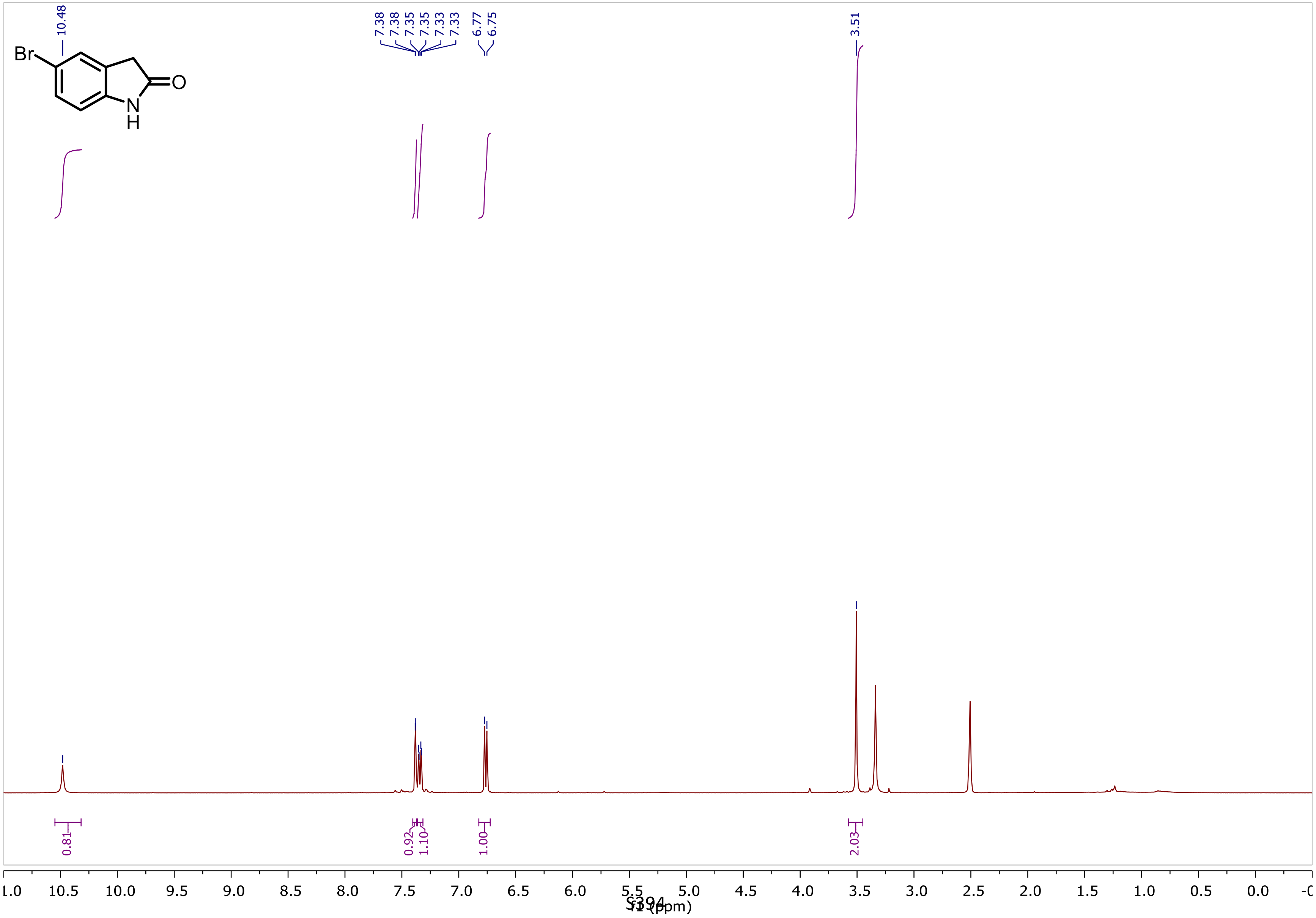

^{13}C NMR spectrum of 5-bromo-2-oxindole **6i** in MeOD-d_4

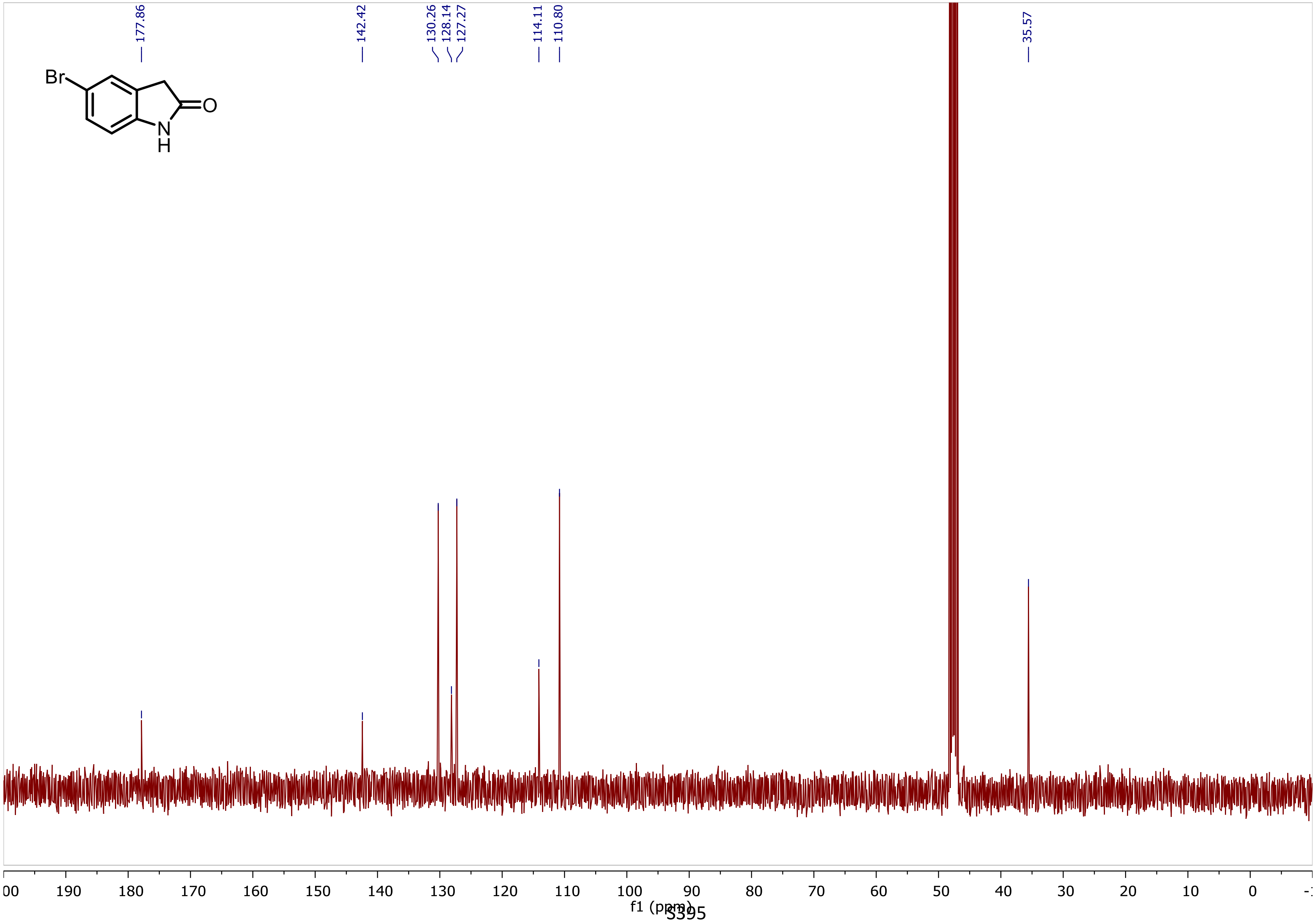

^{1}H NMR spectrum of 7-bromo-2-oxindole ***iso*-6i** in MeOD-d_4

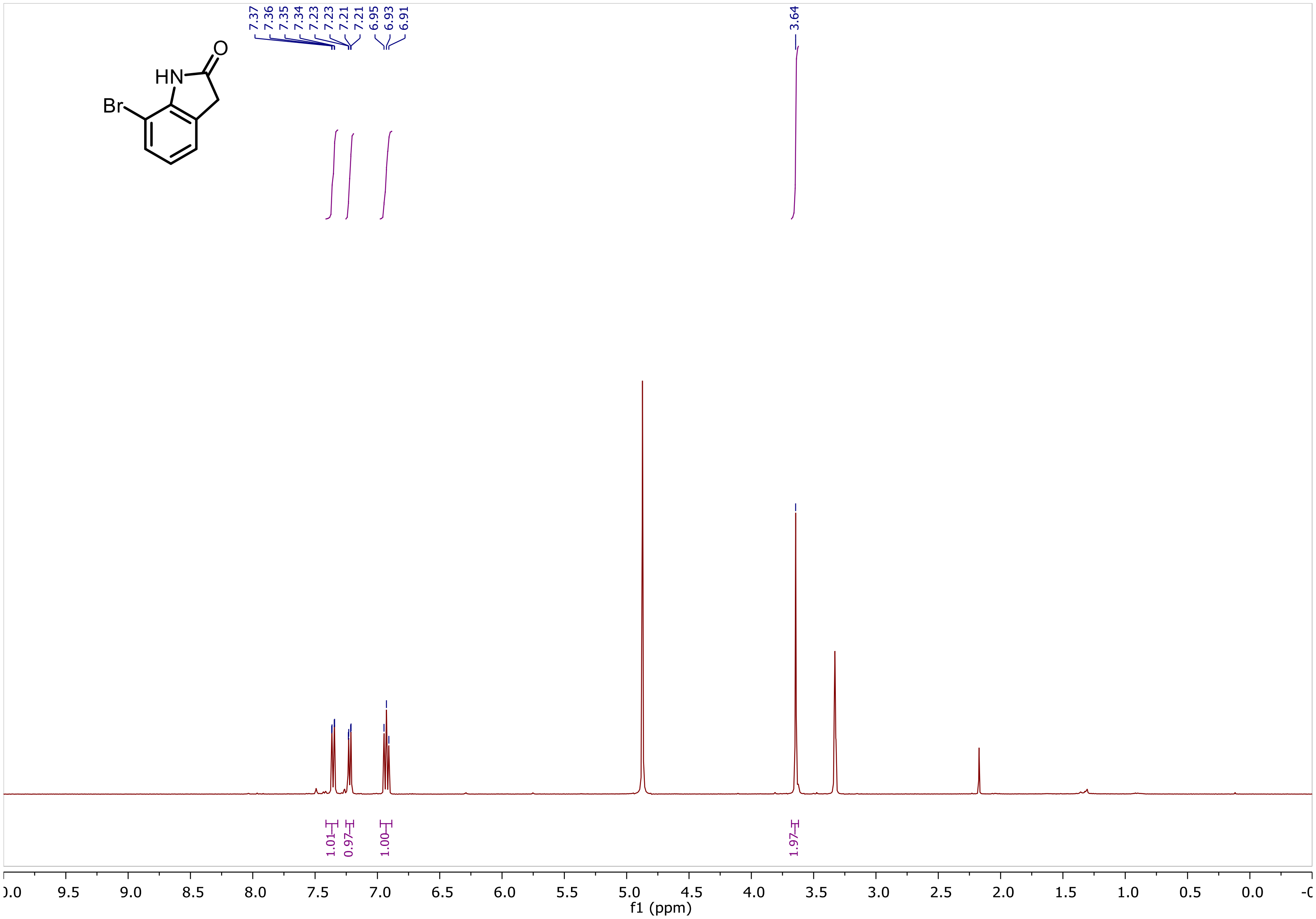

^{13}C NMR spectrum of 7-bromo-2-oxindole ***iso*-6i** in MeOD-d_4

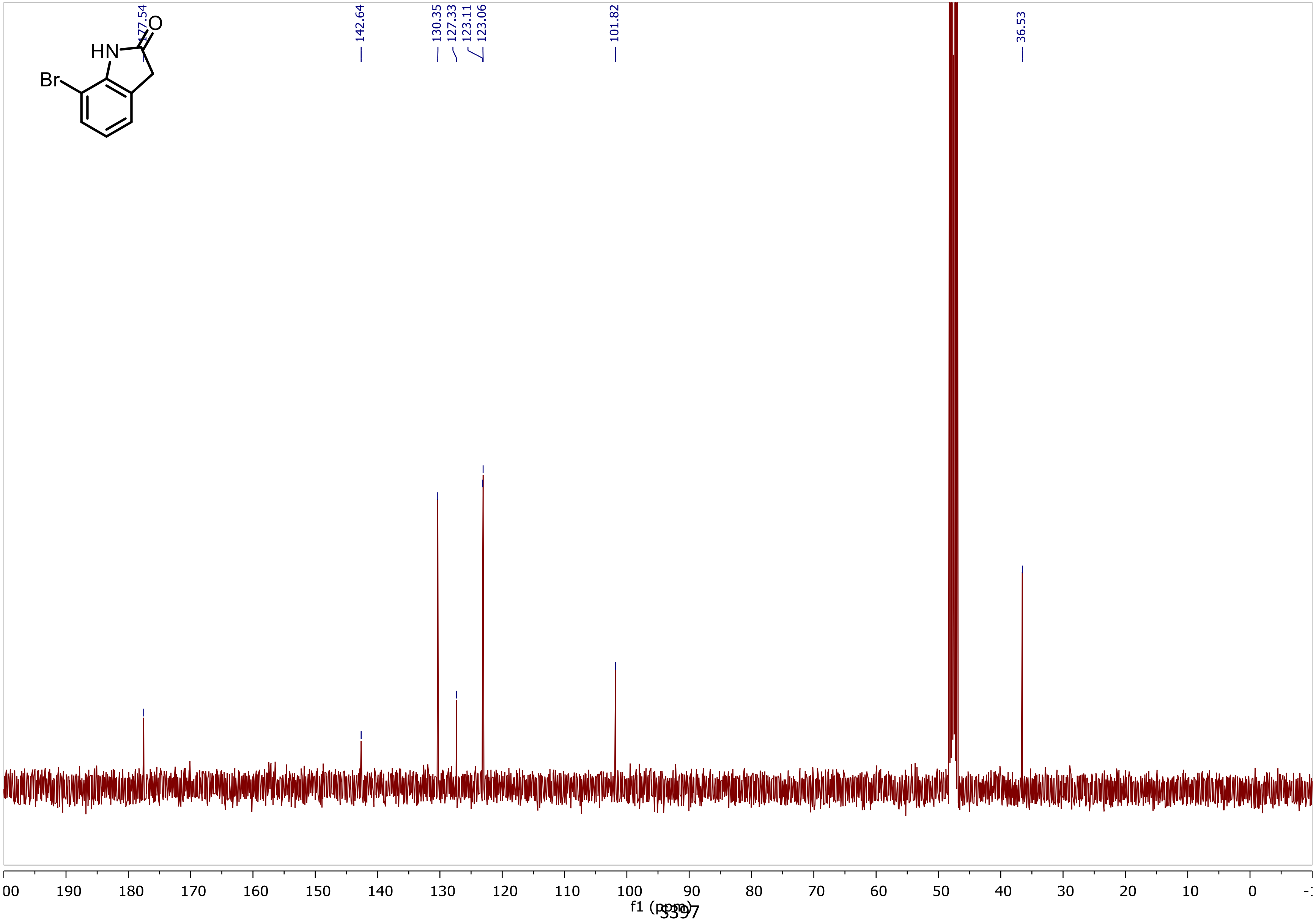

^{1}H NMR spectrum of 6-bromo-3,4-dihydroquinolin-2(1*H*)-one **6j** in $CDCl_3$

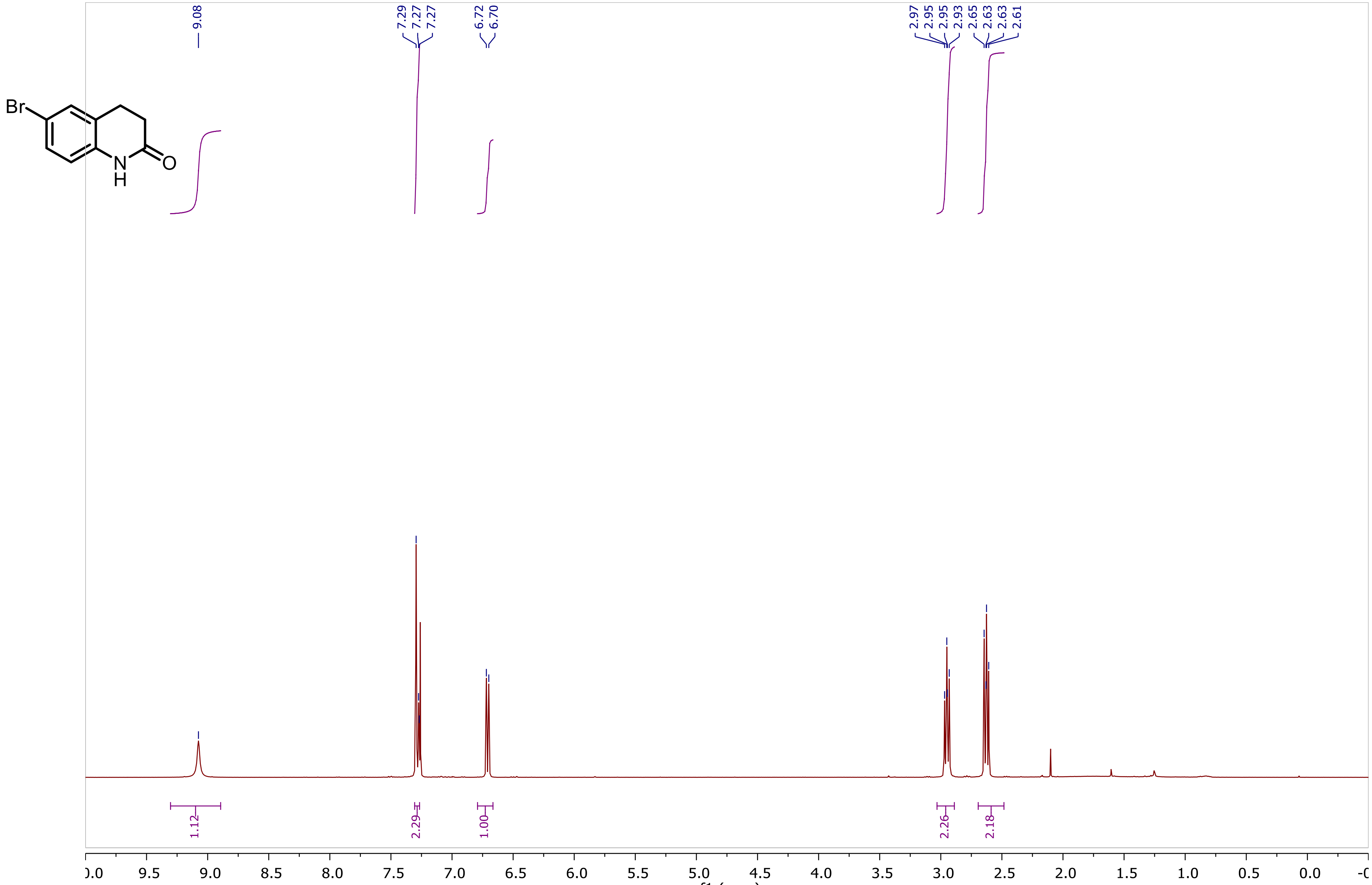

^{13}C NMR spectrum of 6-bromo-3,4-dihydroquinolin-2(1*H*)-one **6j** in $CDCl_3$

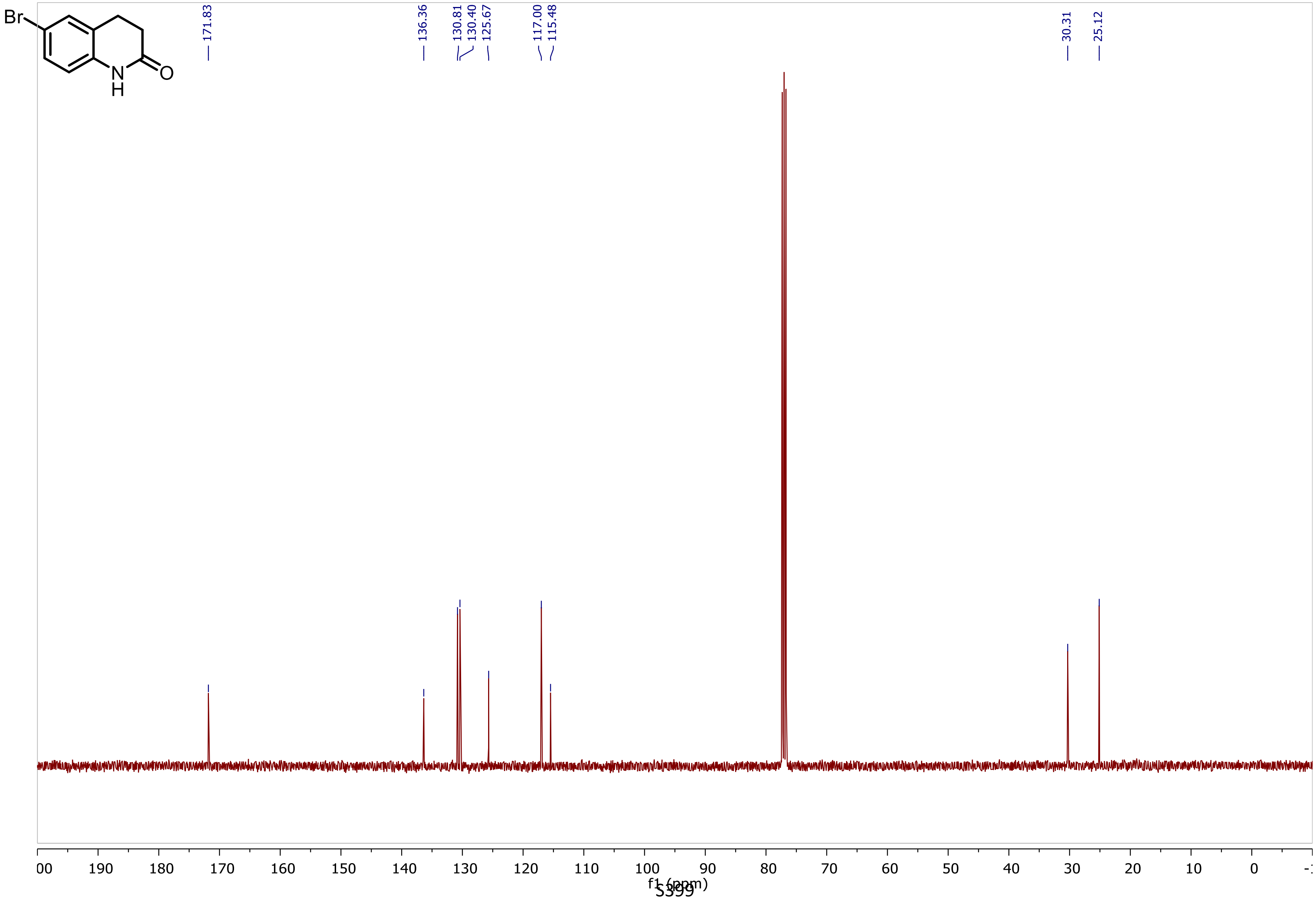

^{1}H NMR spectrum of 8-bromo-3,4-dihydroquinolin-2(1*H*)-one ***iso*-6j** in

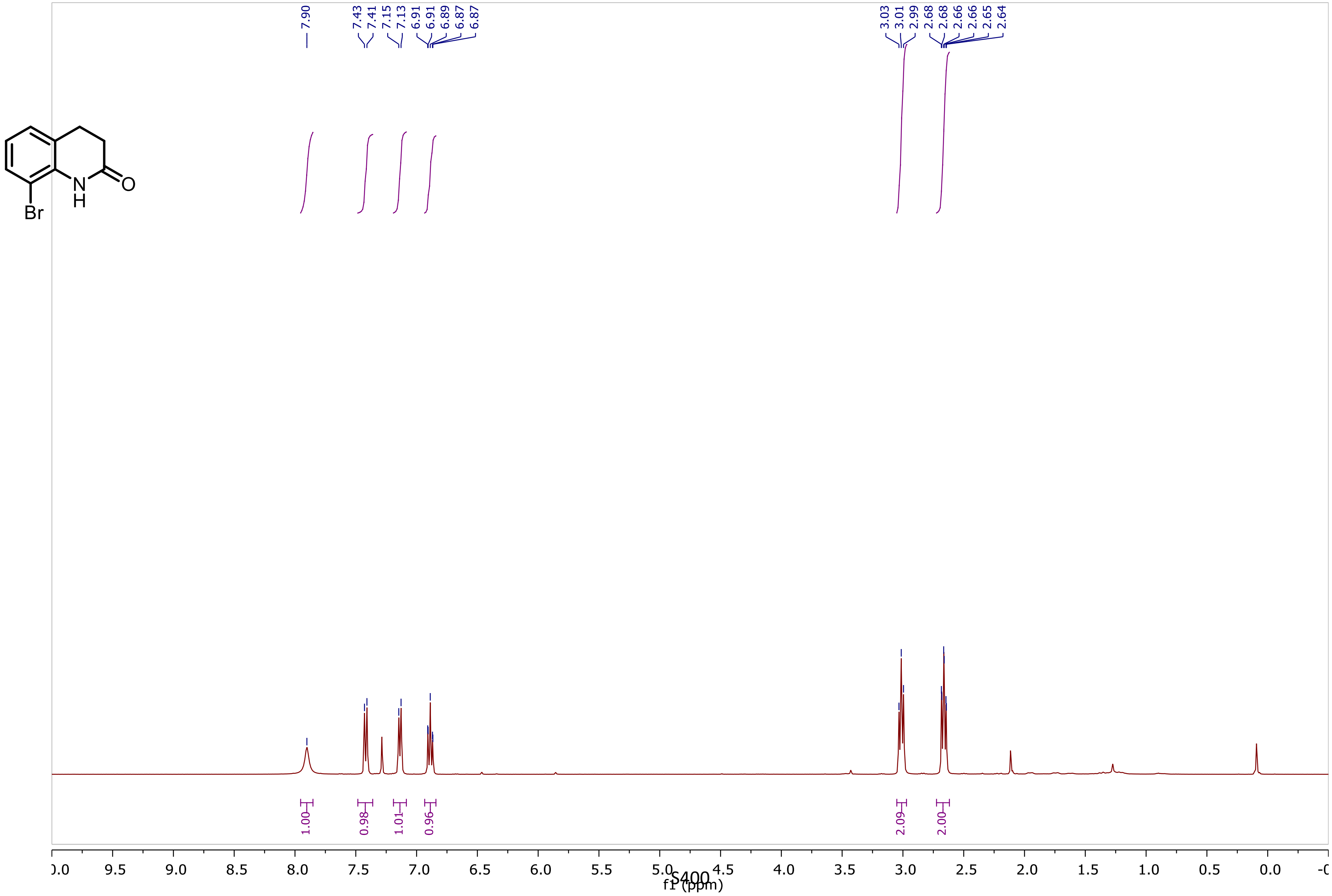

^{13}C NMR spectrum of 8-bromo-3,4-dihydroquinolin-2(1*H*)-one ***iso*-6j** in $CDCl_3$

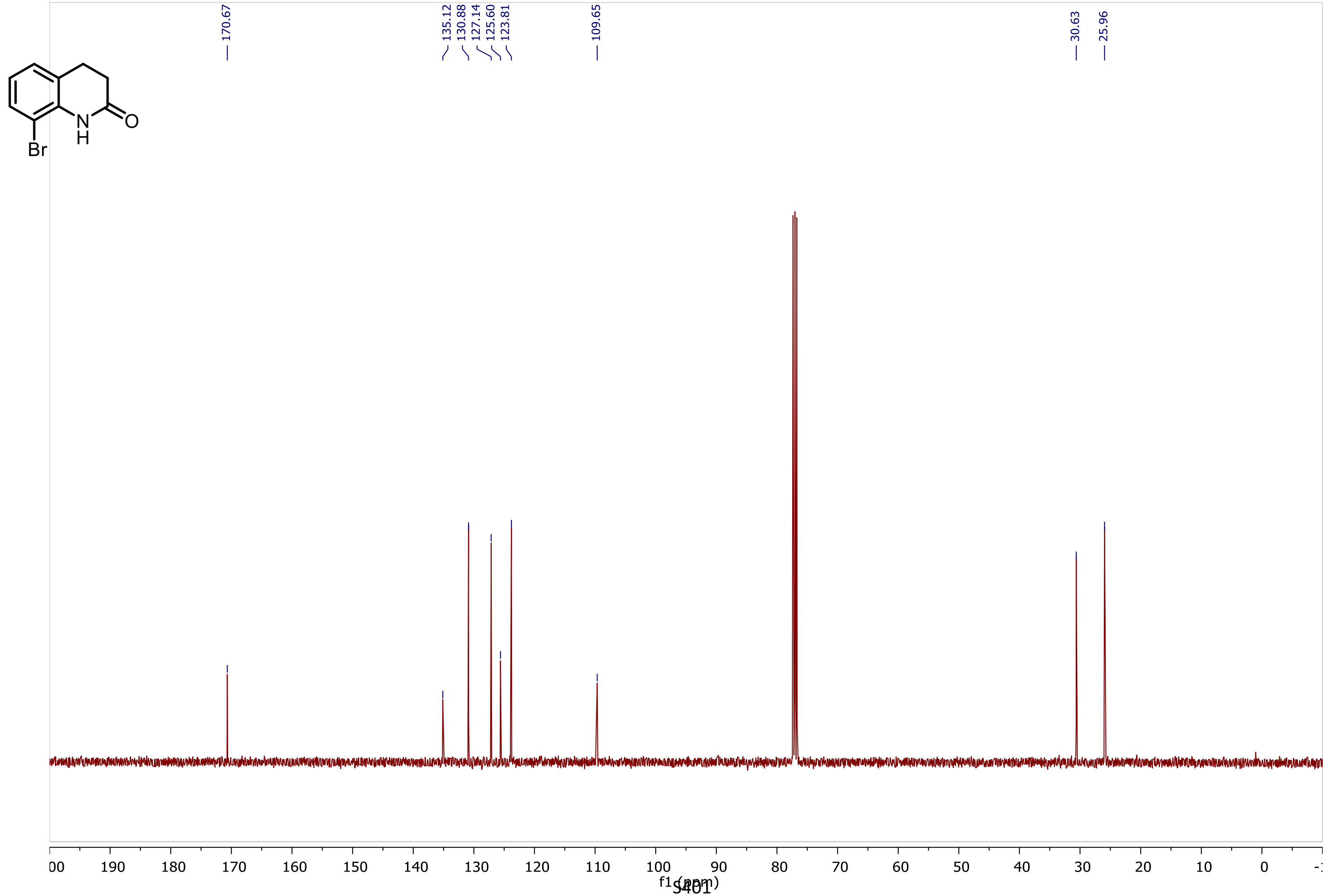

^{1}H NMR spectrum of 6,8-dimethoxy-3,4-dihydroquinolin-2(1*H*)-one **6k** in $CDCl_3$

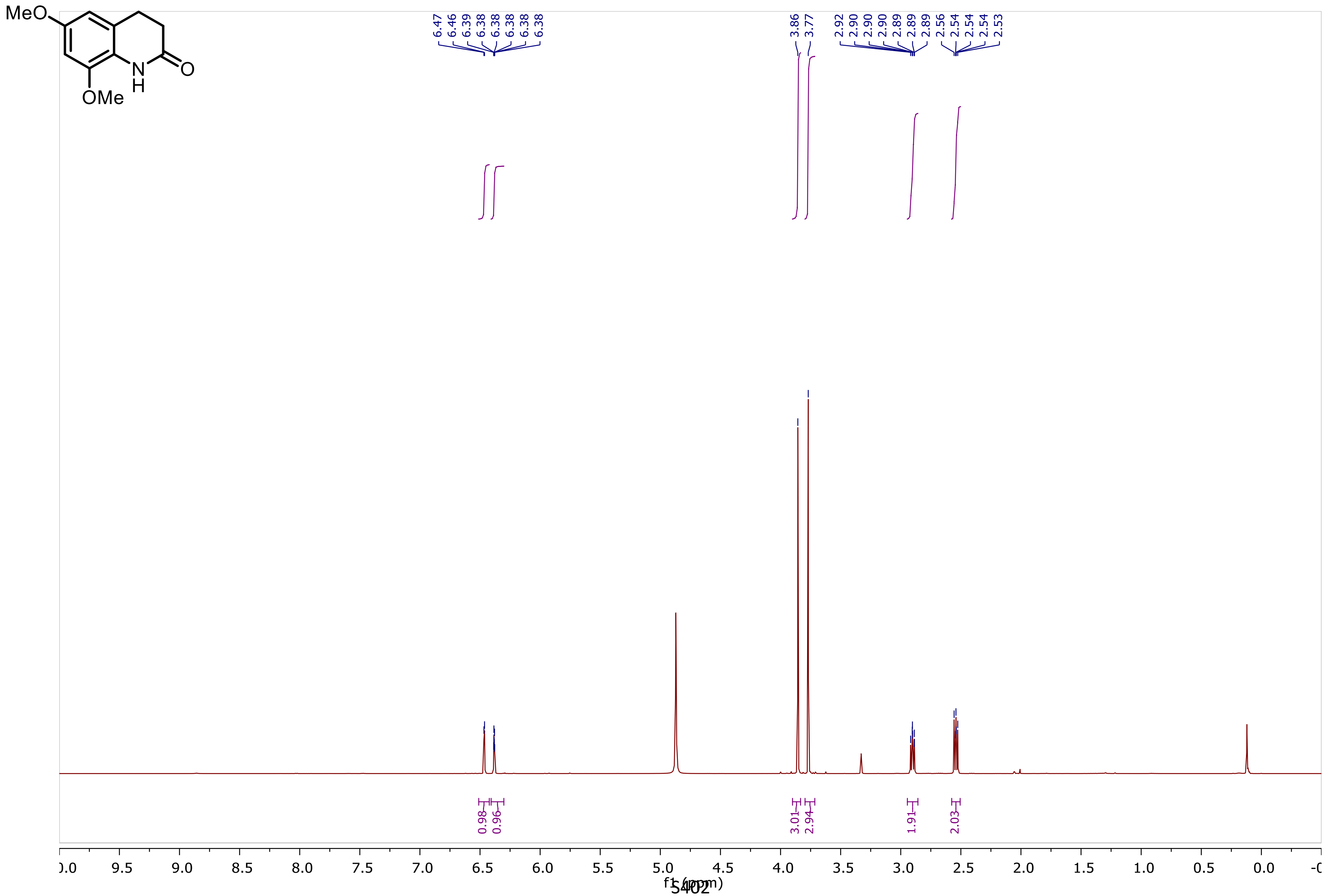

^{13}C NMR spectrum of 6,8-dimethoxy-3,4-dihydroquinolin-2(1*H*)-one **6k** in $CDCl_3$

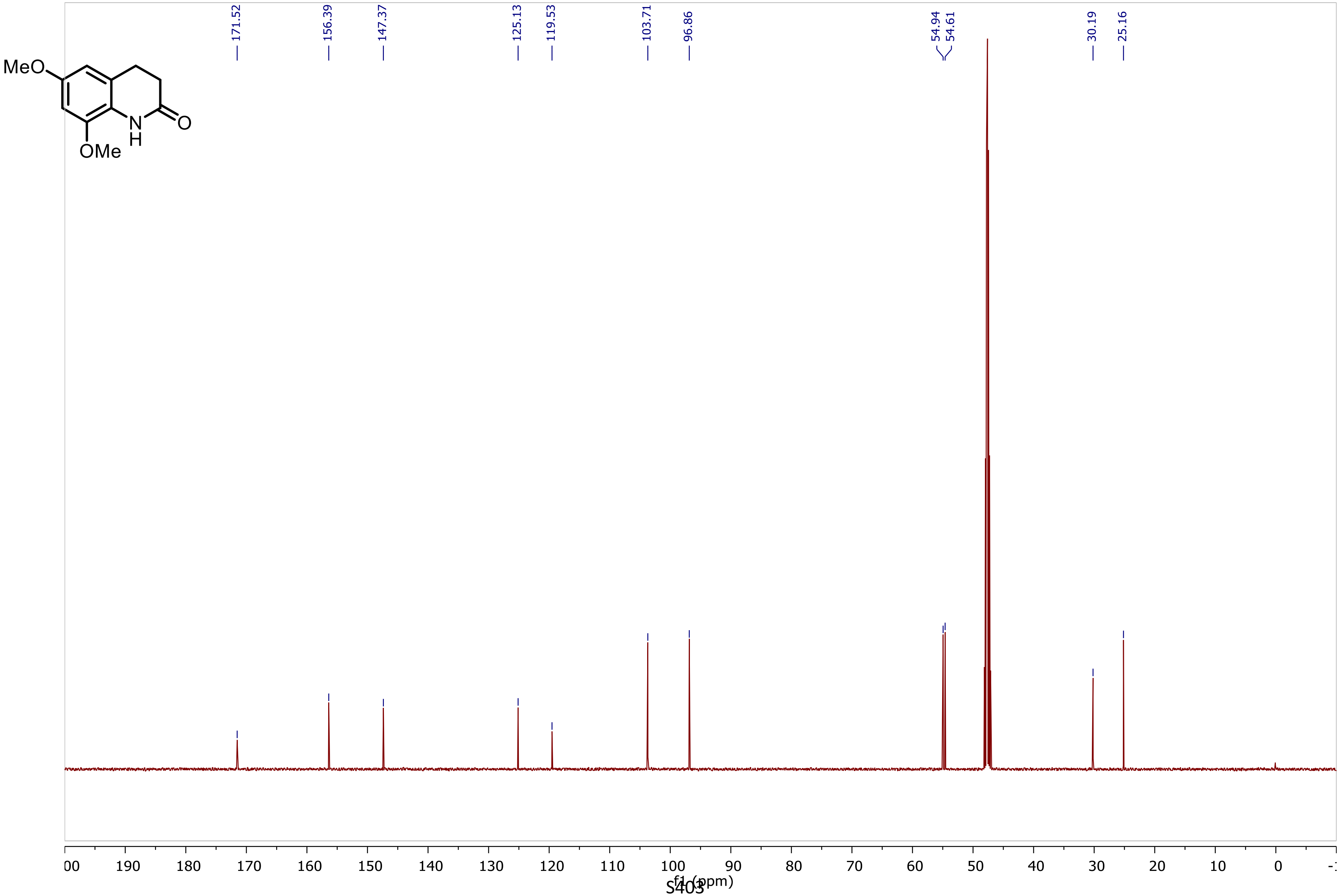

^1H NMR spectrum of 7-methyl-3,4-dihydroquinolin-2(1*H*)-one **6l** in $CDCl_3$

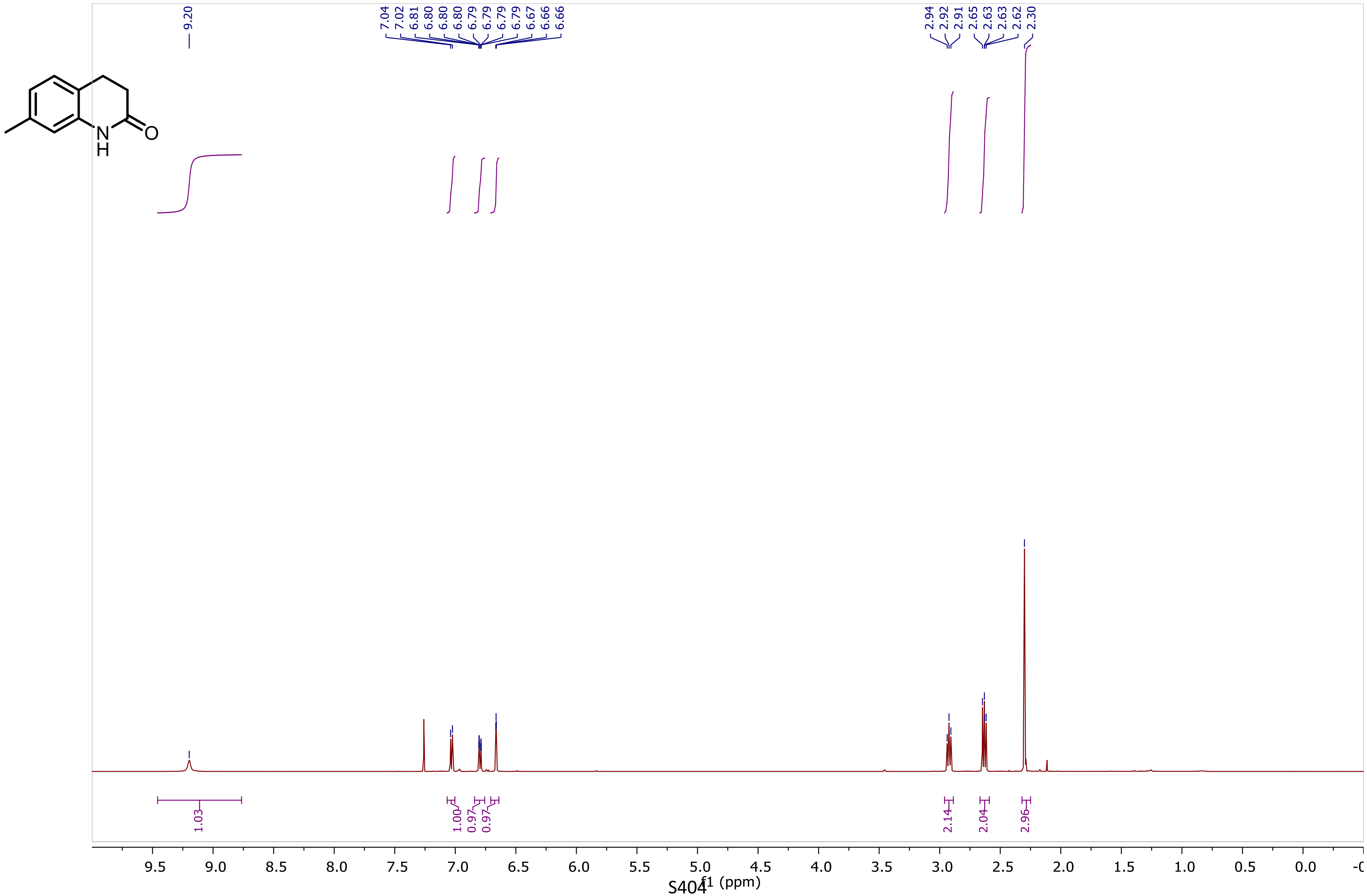

^{13}C NMR spectrum of 7-methyl-3,4-dihydroquinolin-2(1*H*)-one **6l** in $CDCl_3$

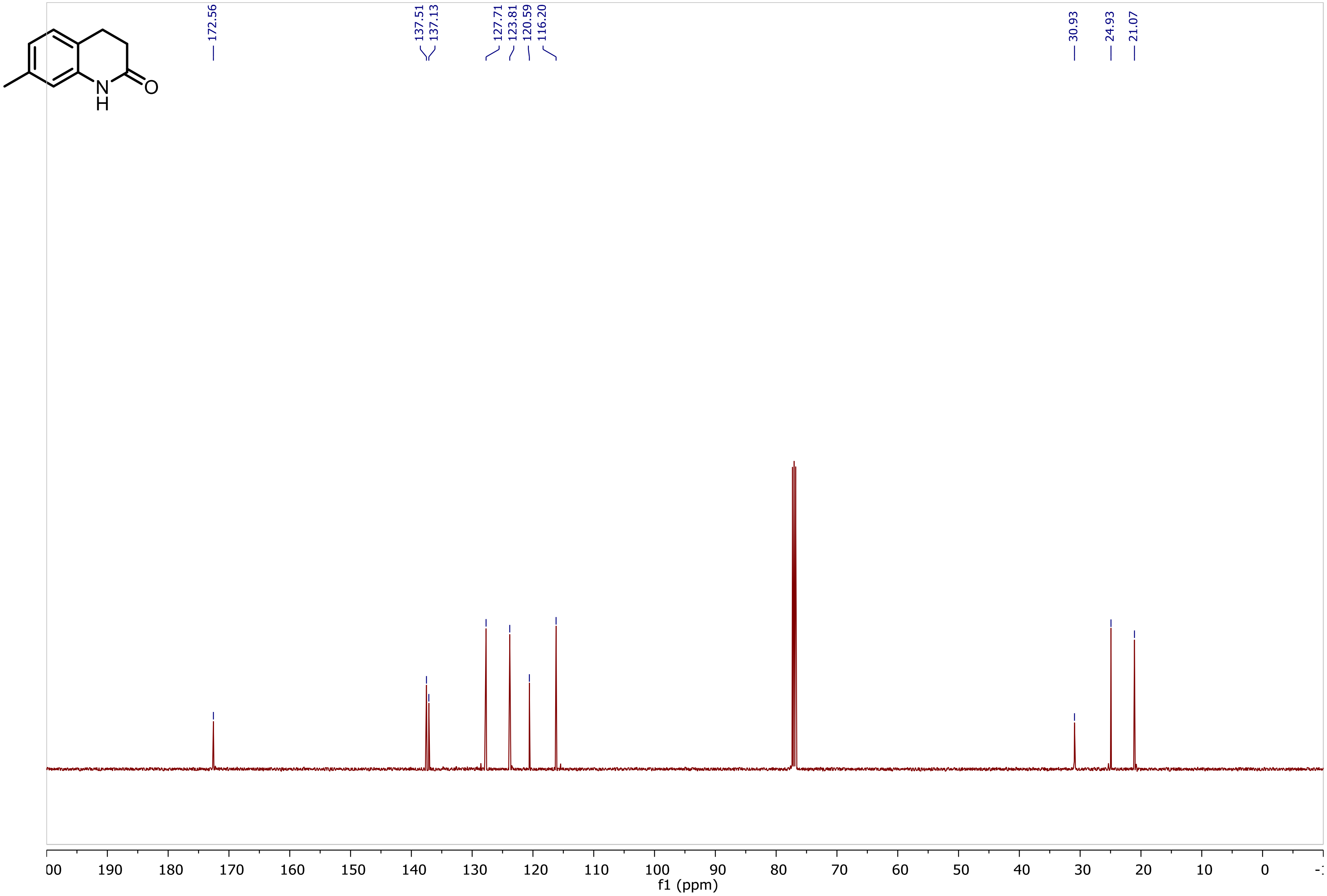

^{1}H NMR spectrum of 3-(3-amino-4-methylphenyl)propanoic acid **6m** in $CDCl_3$

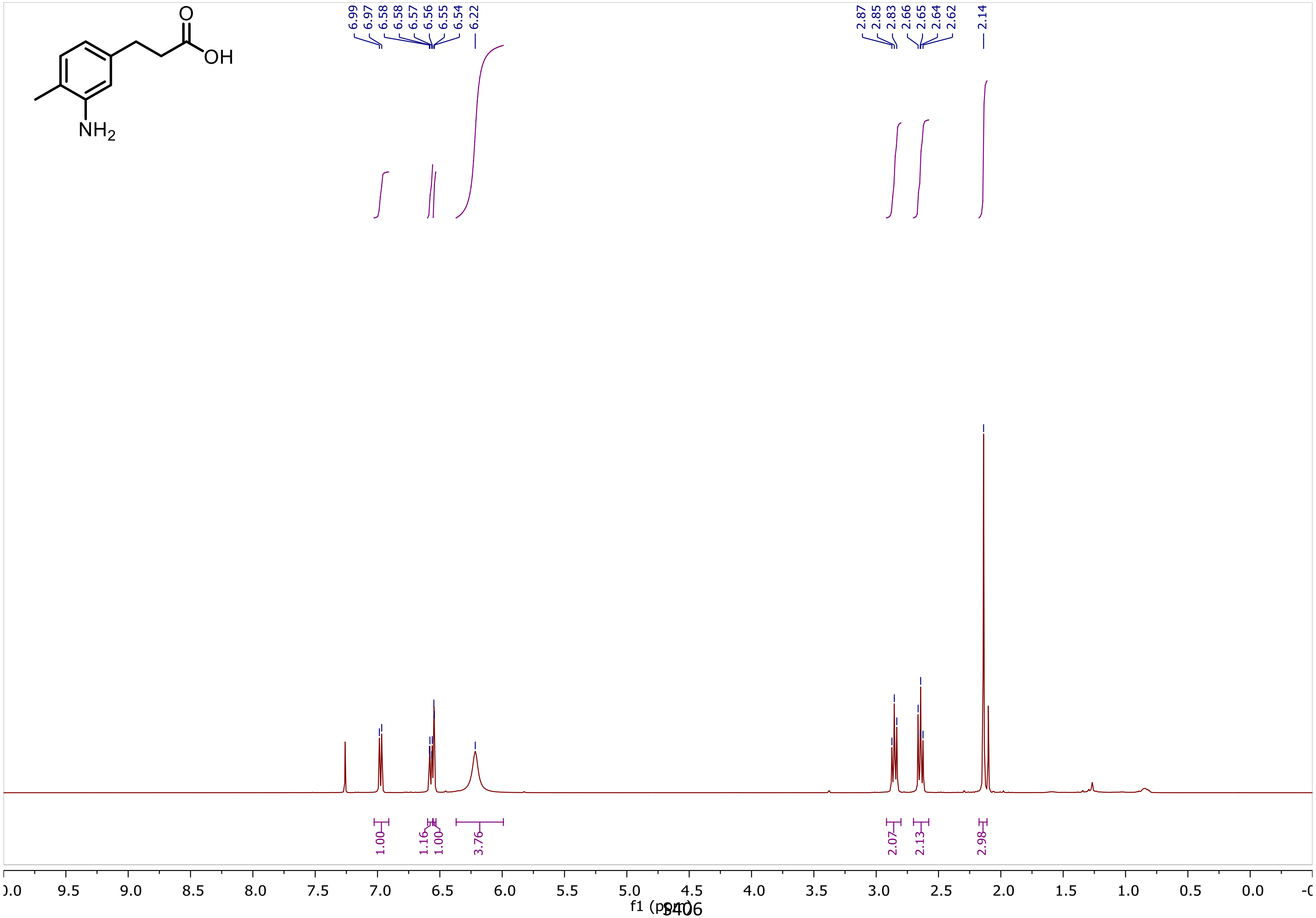

^{13}C NMR spectrum of 3-(3-amino-4-methylphenyl)propanoic acid **6m** in $CDCl_3$

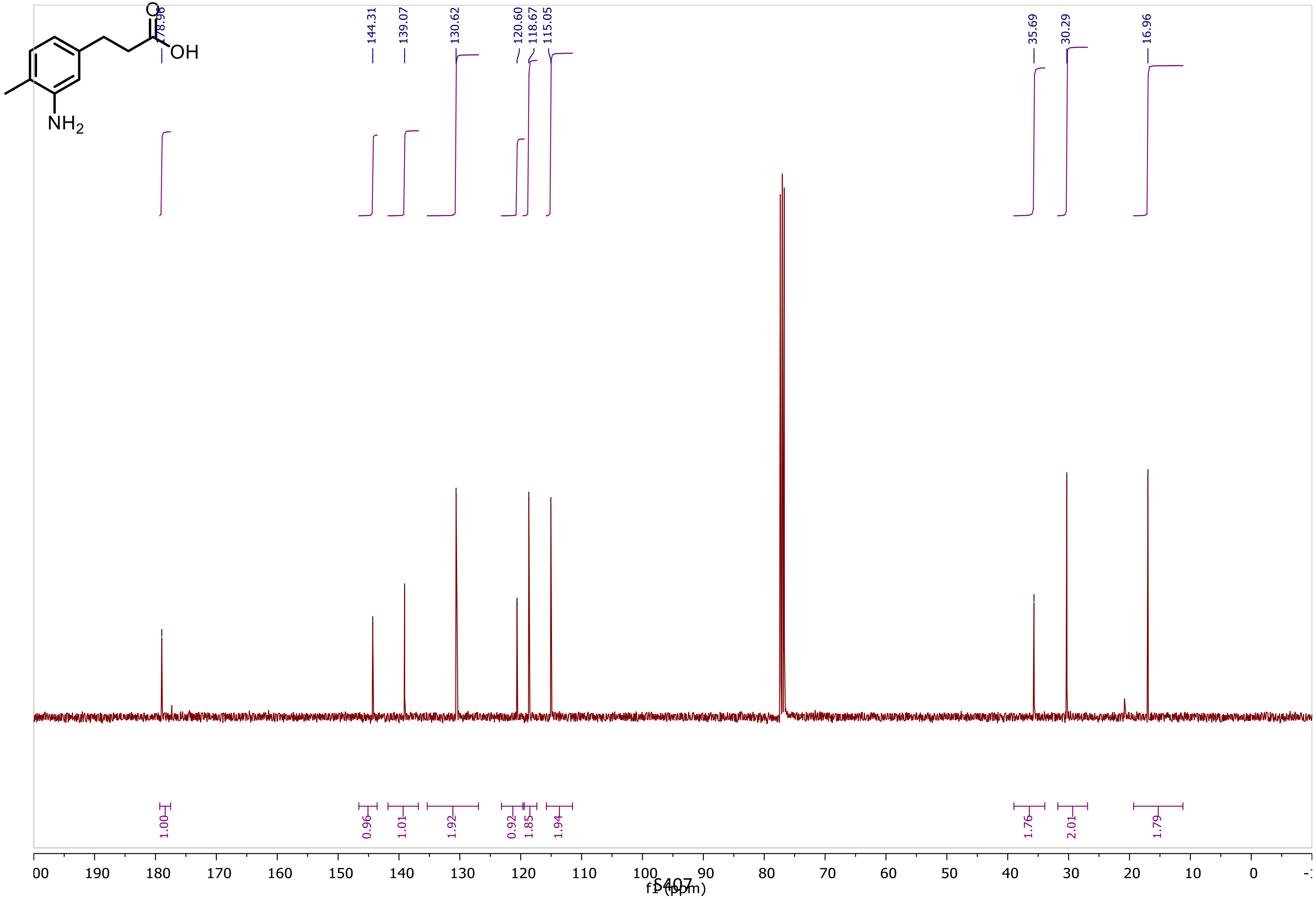

Mechanistic Studies

^{1}H NMR spectrum of (phenyl-2-d)methanol-d **15** in $CDCl_3$

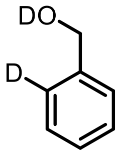

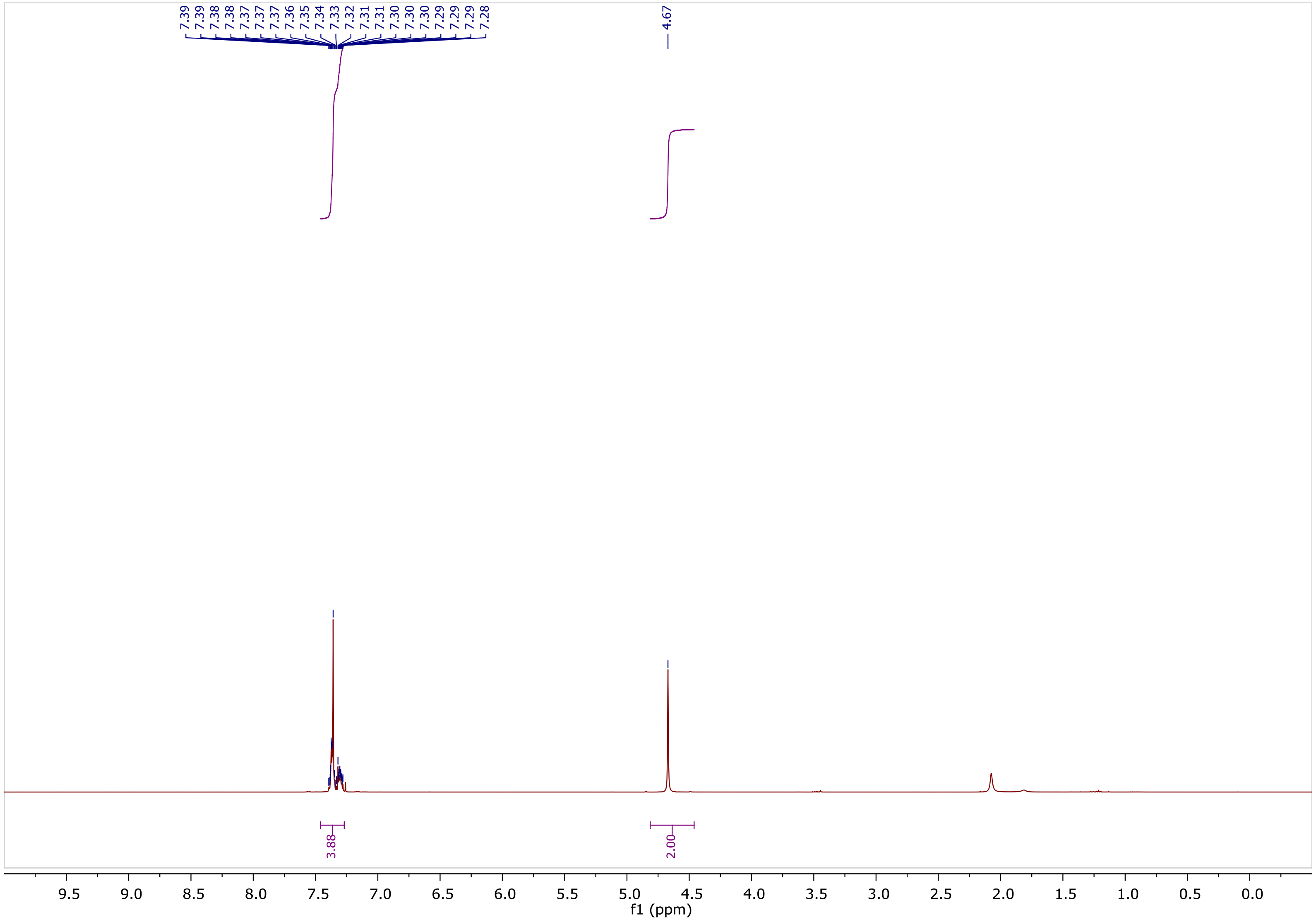

^{13}C NMR spectrum of (phenyl-2-d)methanol-d **15** in $CDCl_3$

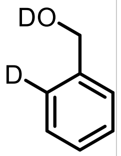

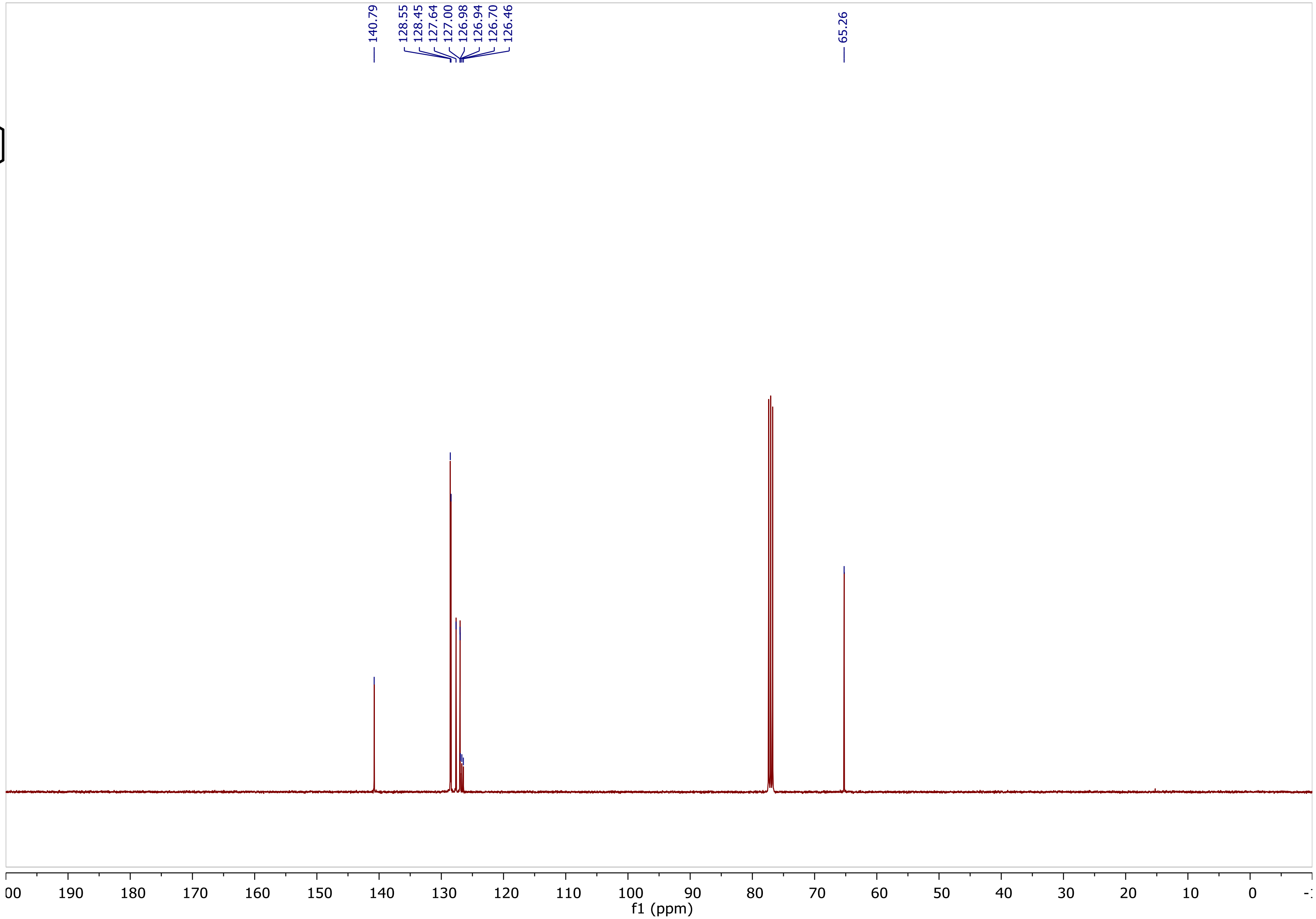

^{1}H NMR spectrum of benzaldehyde-2-*d* **16** in $CDCl_3$

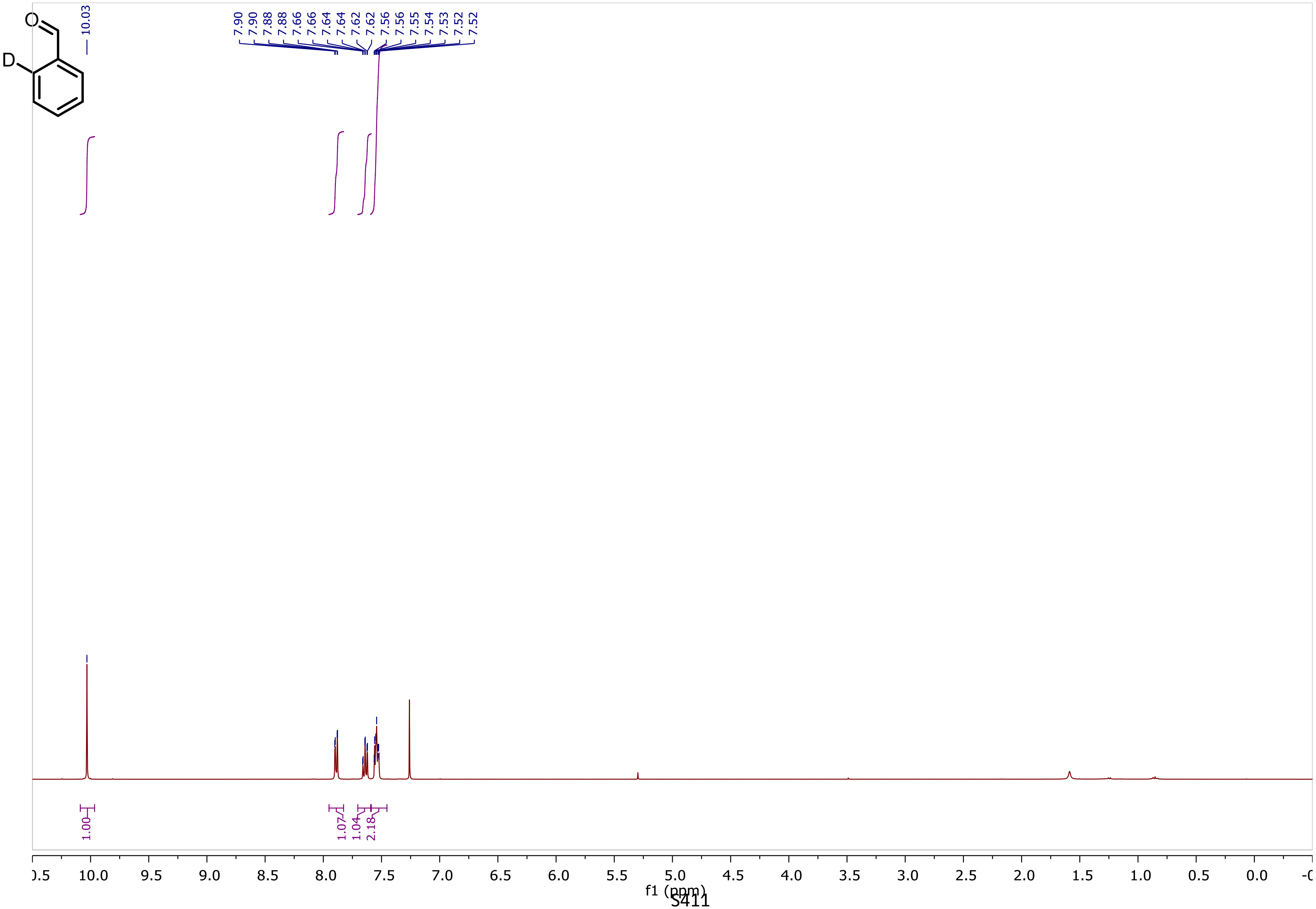

^{13}C NMR spectrum of benzaldehyde-2-*d* **16** in $CDCl_3$

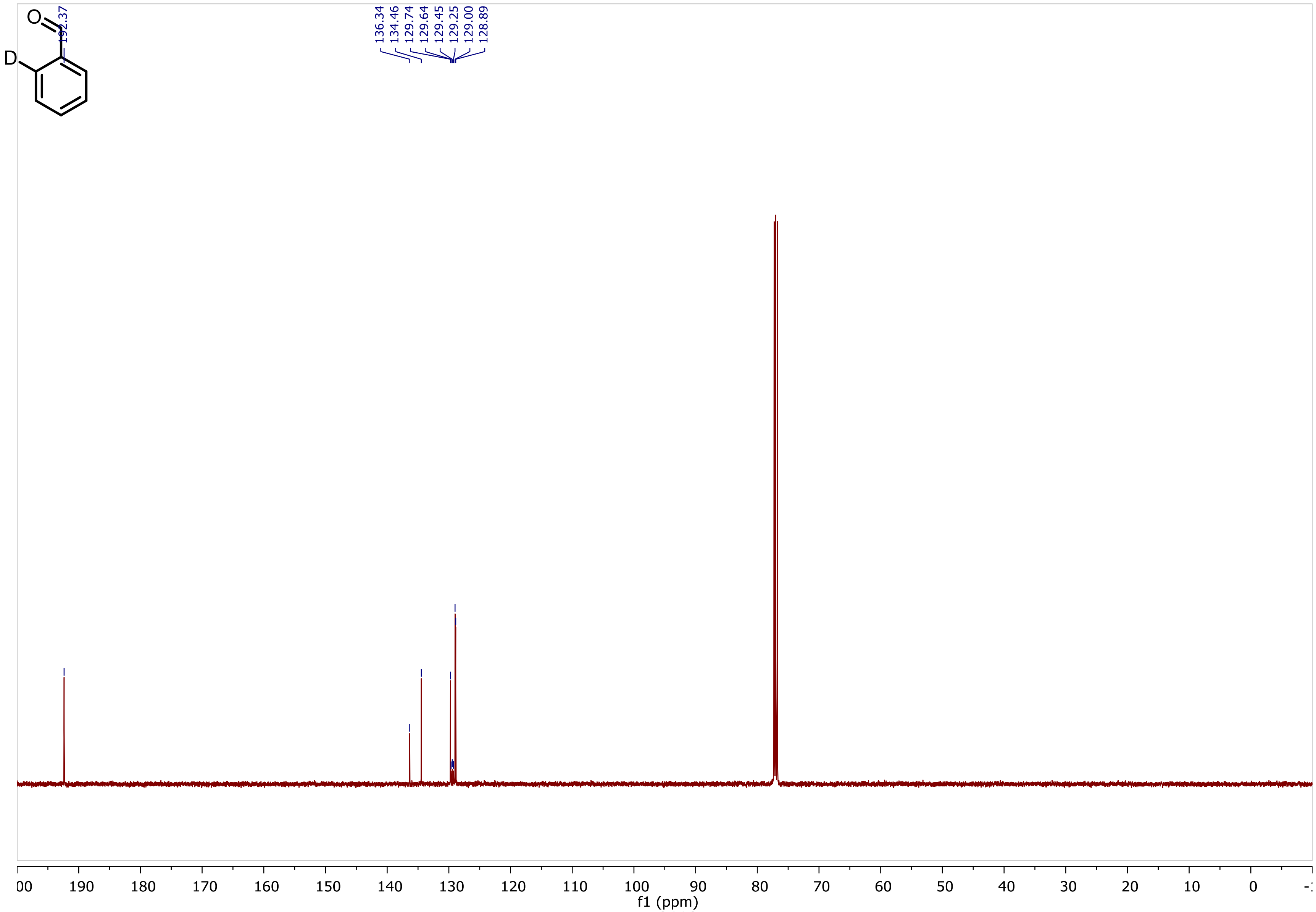

^1H NMR spectrum of *tert*-butyl ((benzoyl-2-*d*)oxy)carbamate ***d*$_1$-1a** in $CDCl_3$

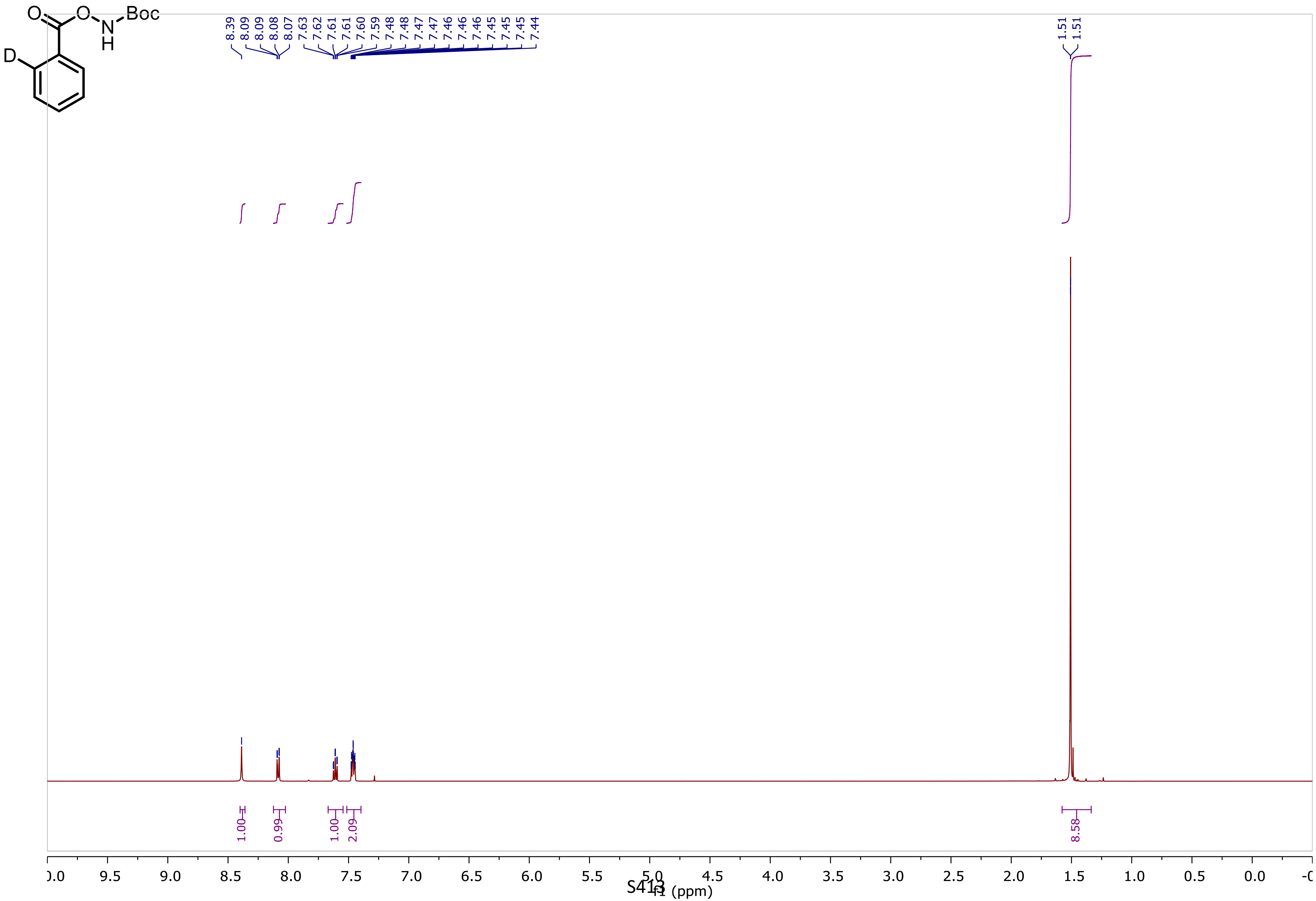

^{13}C NMR spectrum of *tert*-butyl ((benzoyl-2-*d*)oxy)carbamate ***d*$_1$-1a** in $CDCl_3$

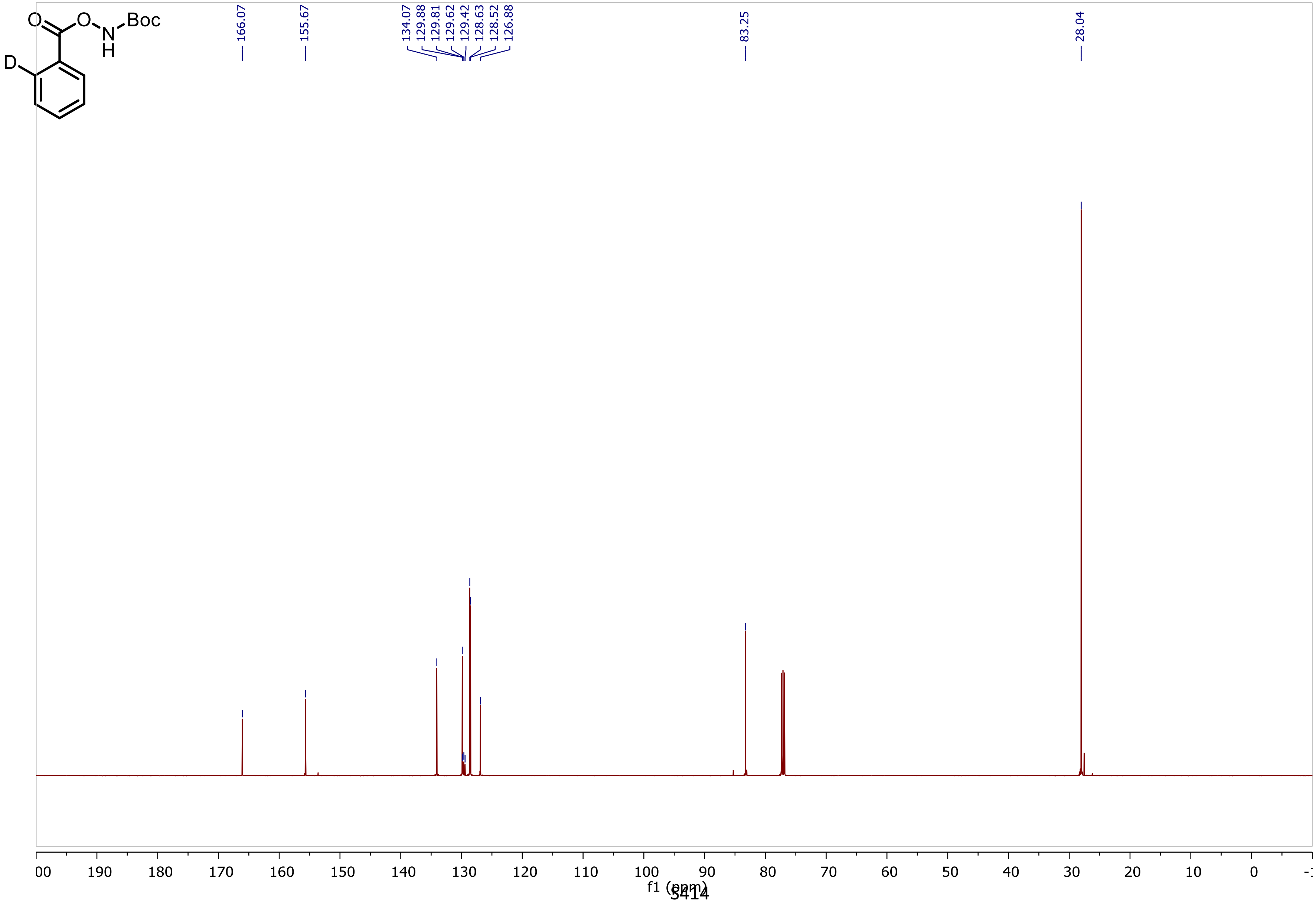

^{1}H NMR spectrum of benzoyl *O*-hydroxylamine **9b** in $CDCl_3$

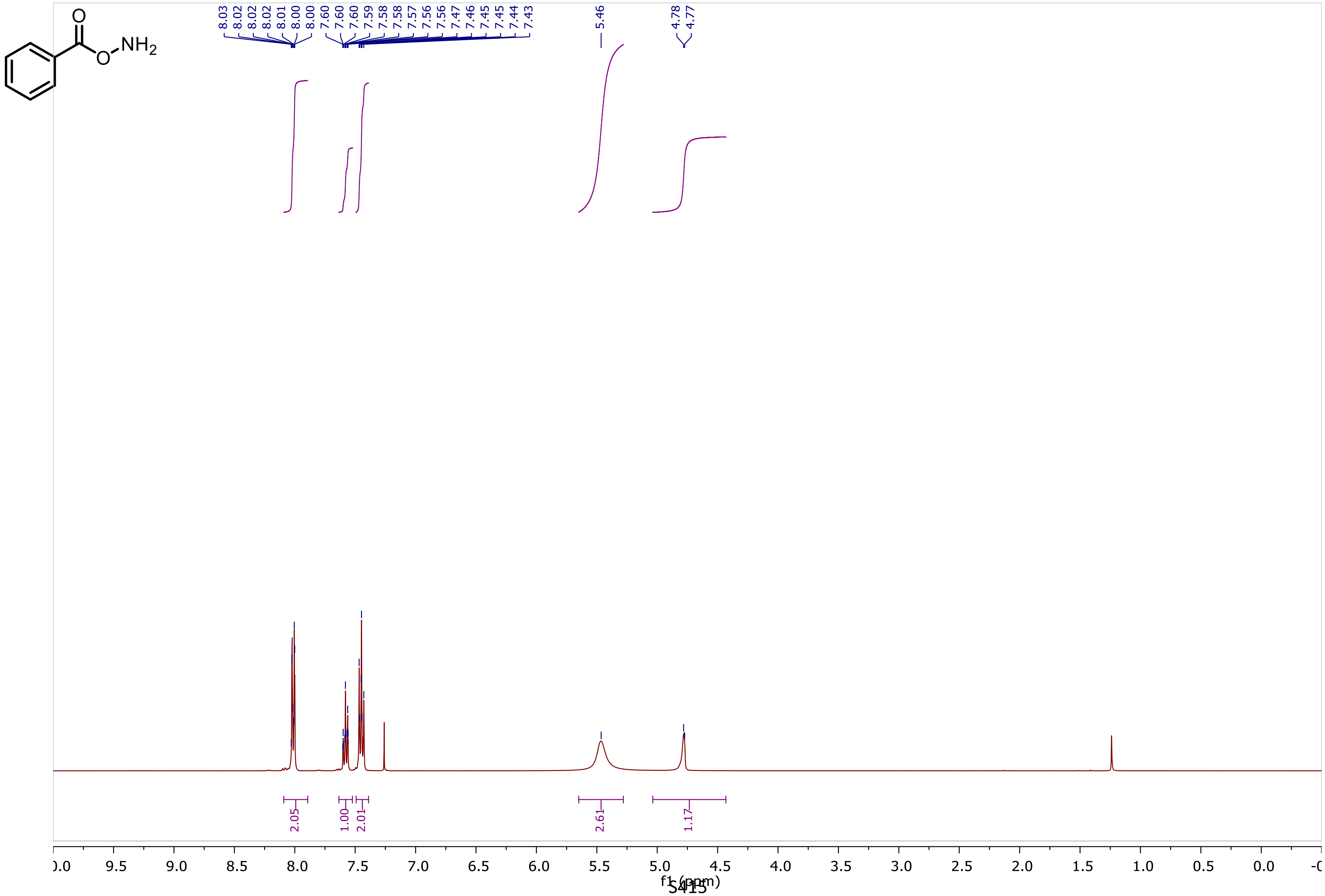

^{13}C NMR spectrum of benzoyl *O*-hydroxylamine **9b** in $CDCl_3$

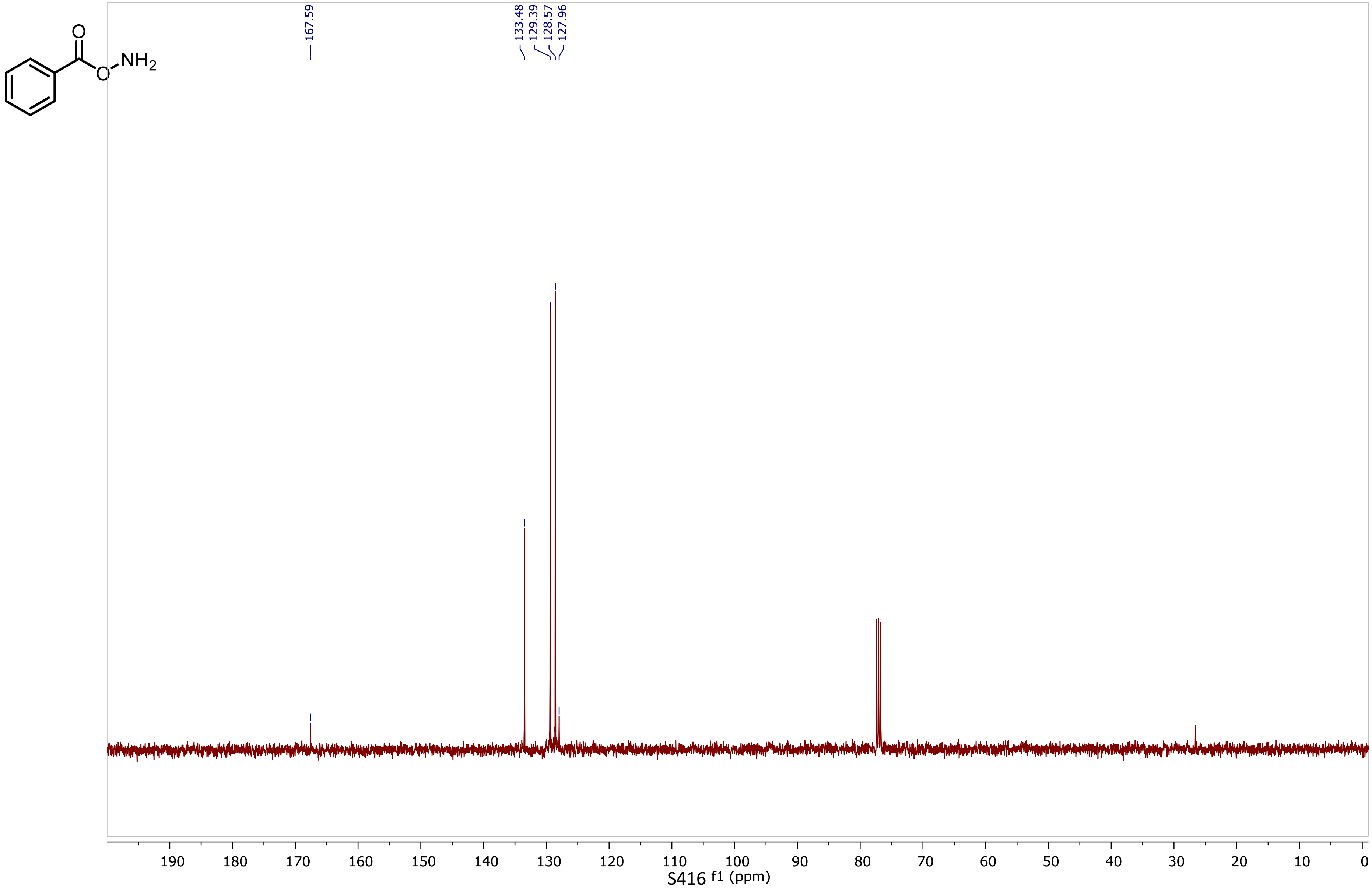

^{1}H NMR spectrum of *tert*-butyl (2-(4-methoxyphenyl)acetoxy)carbamate **1aj** in $CDCl_3$

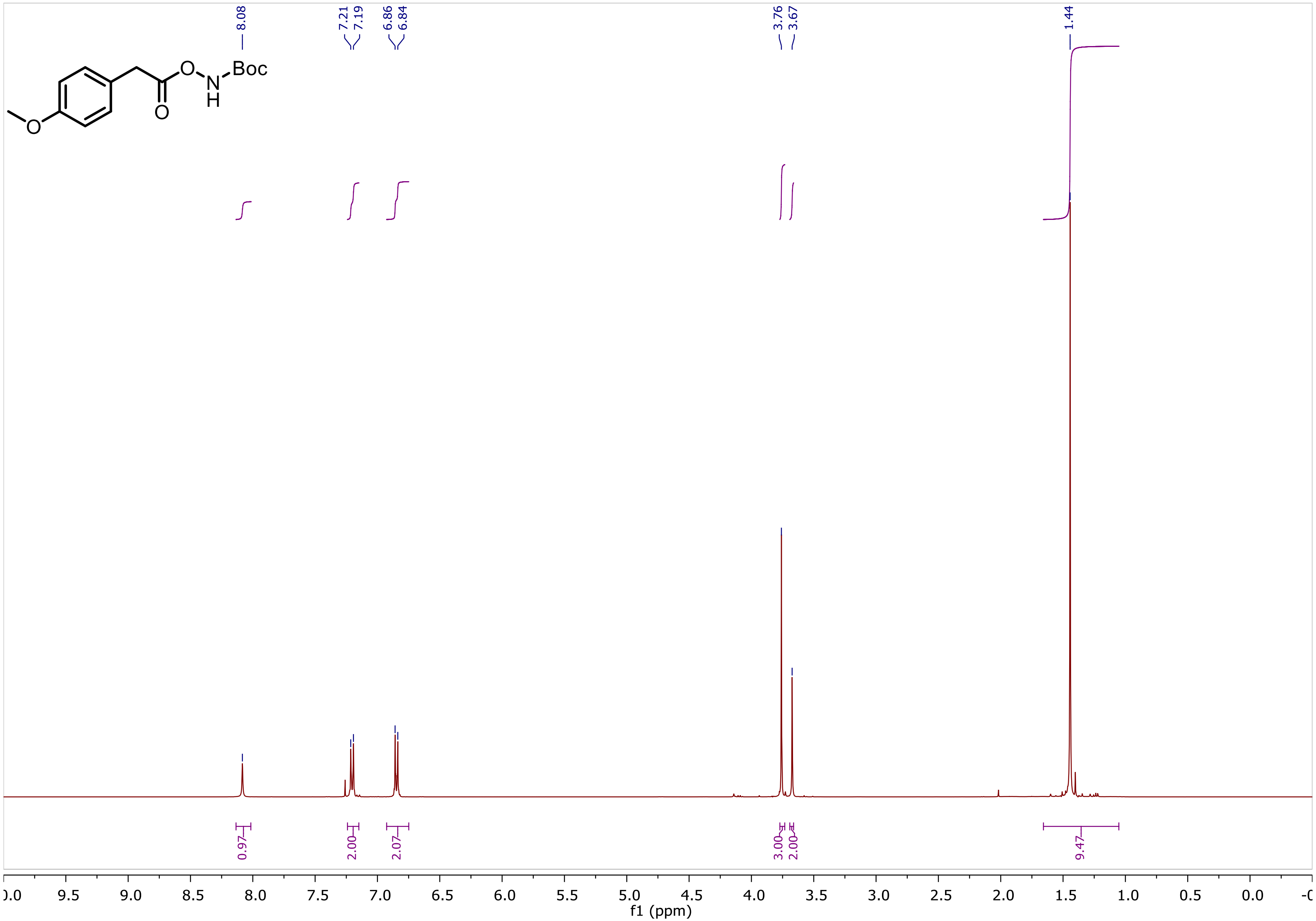

^{13}C NMR spectrum of *tert*-butyl (2-(4-methoxyphenyl)acetoxy)carbamate **1aj** in $CDCl_3$

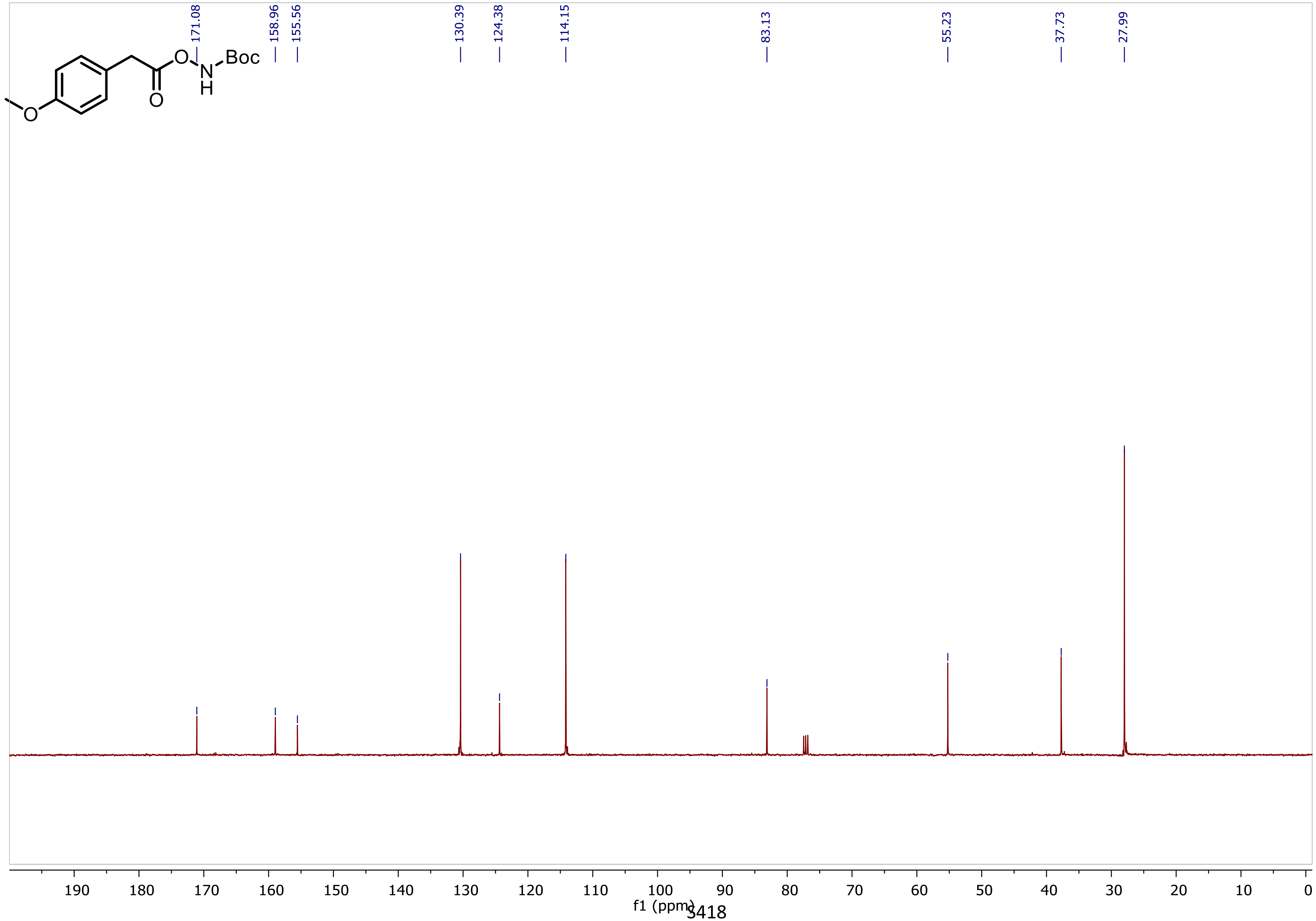

^1H NMR spectrum of *tert*-butyl ((3-(4-methoxyphenyl)propanoyl)oxy)carbamate **1ak** in $CDCl_3$

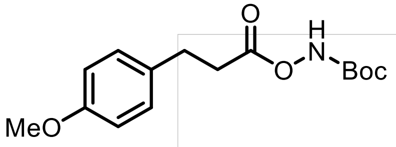

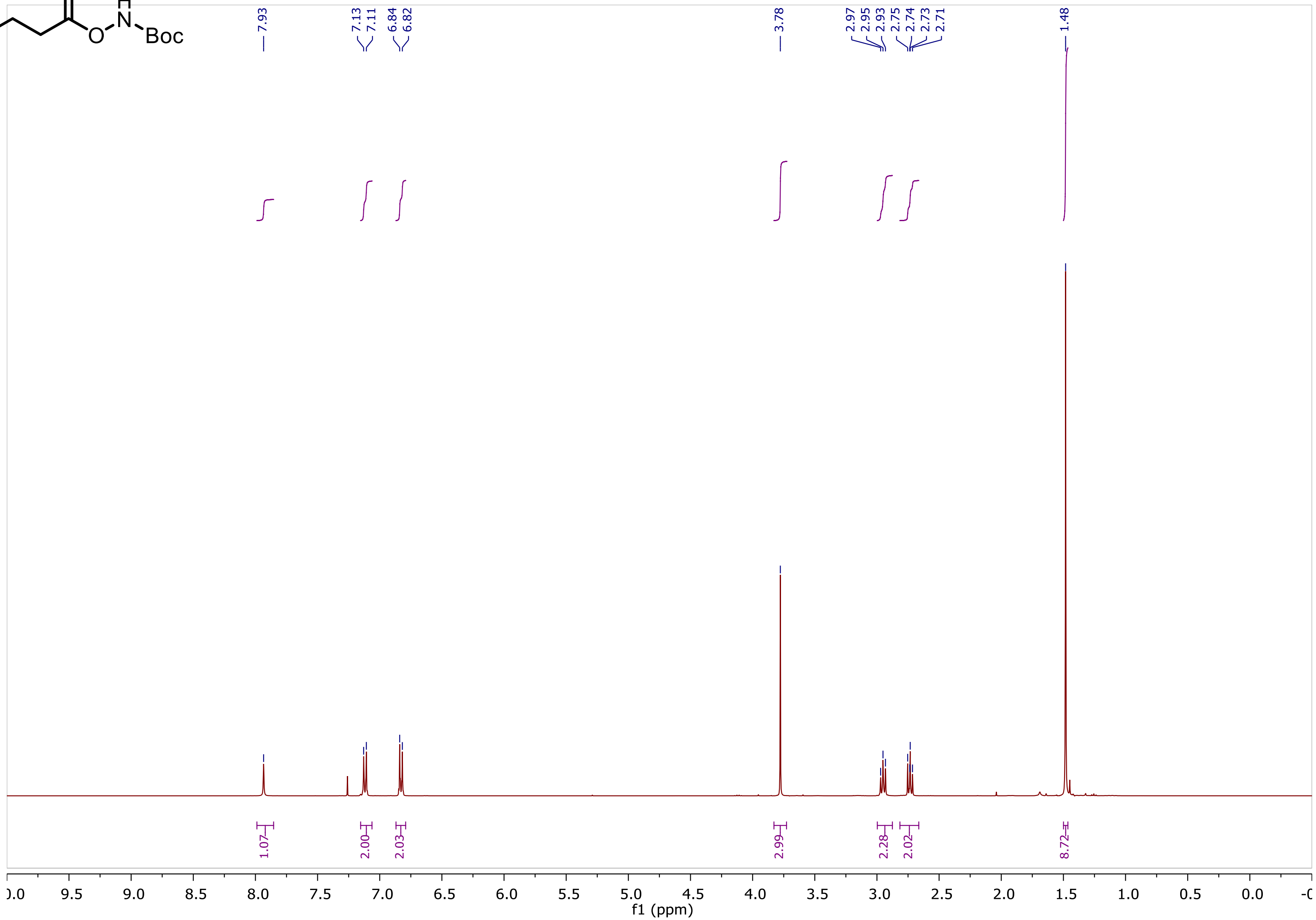

^{13}C NMR spectrum of *tert*-butyl ((3-(4-methoxyphenyl)propanoyl)oxy)carbamate **1ak** in $CDCl_3$

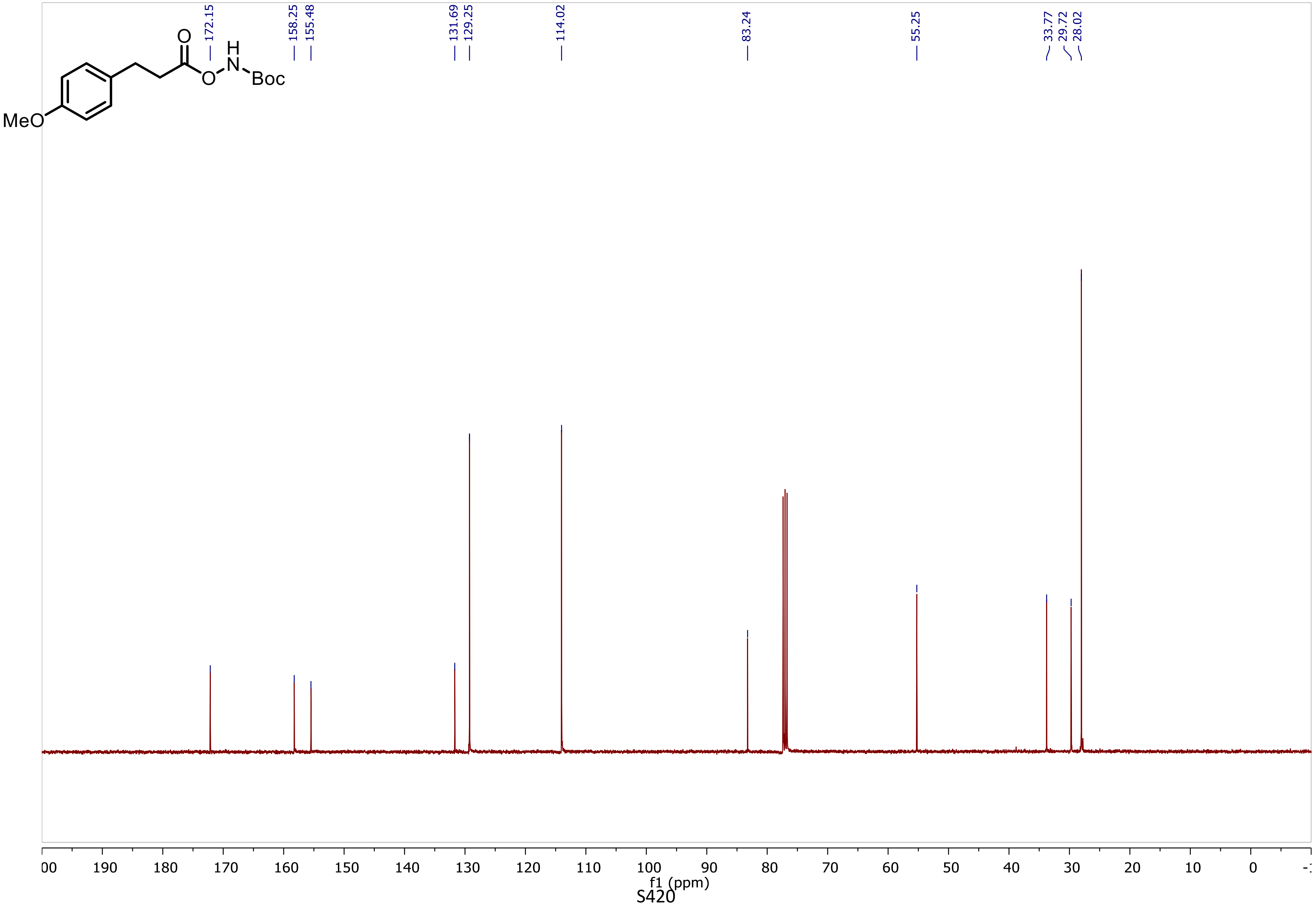

^{1}H NMR spectrum of *tert*-butyl ((4-(4-methoxyphenyl)butanoyl)oxy)carbamate **1al** in $CDCl_3$

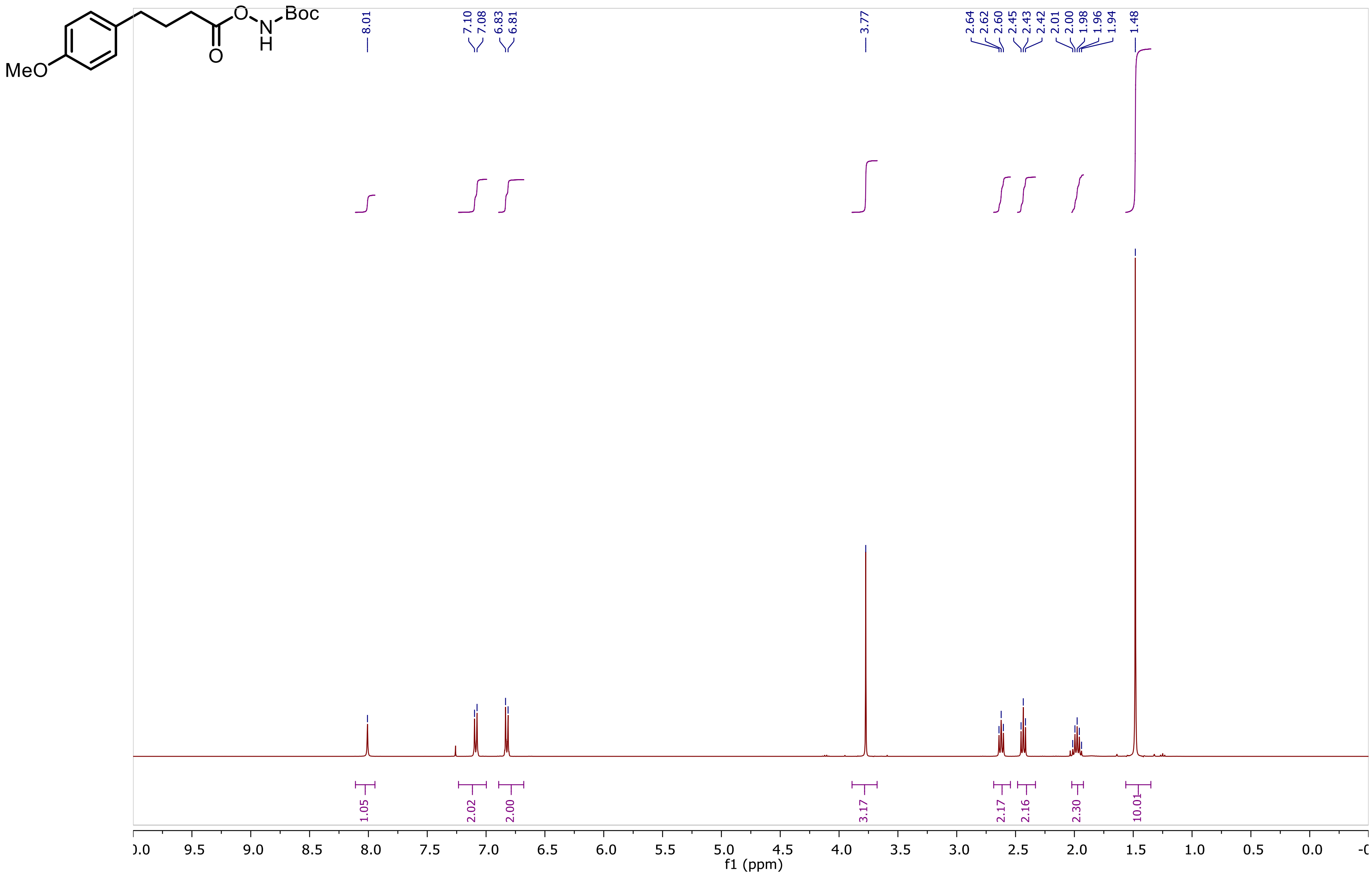

^{13}C NMR spectrum of *tert*-butyl ((4-(4-methoxyphenyl)butanoyl)oxy)carbamate **1al** in $CDCl_3$

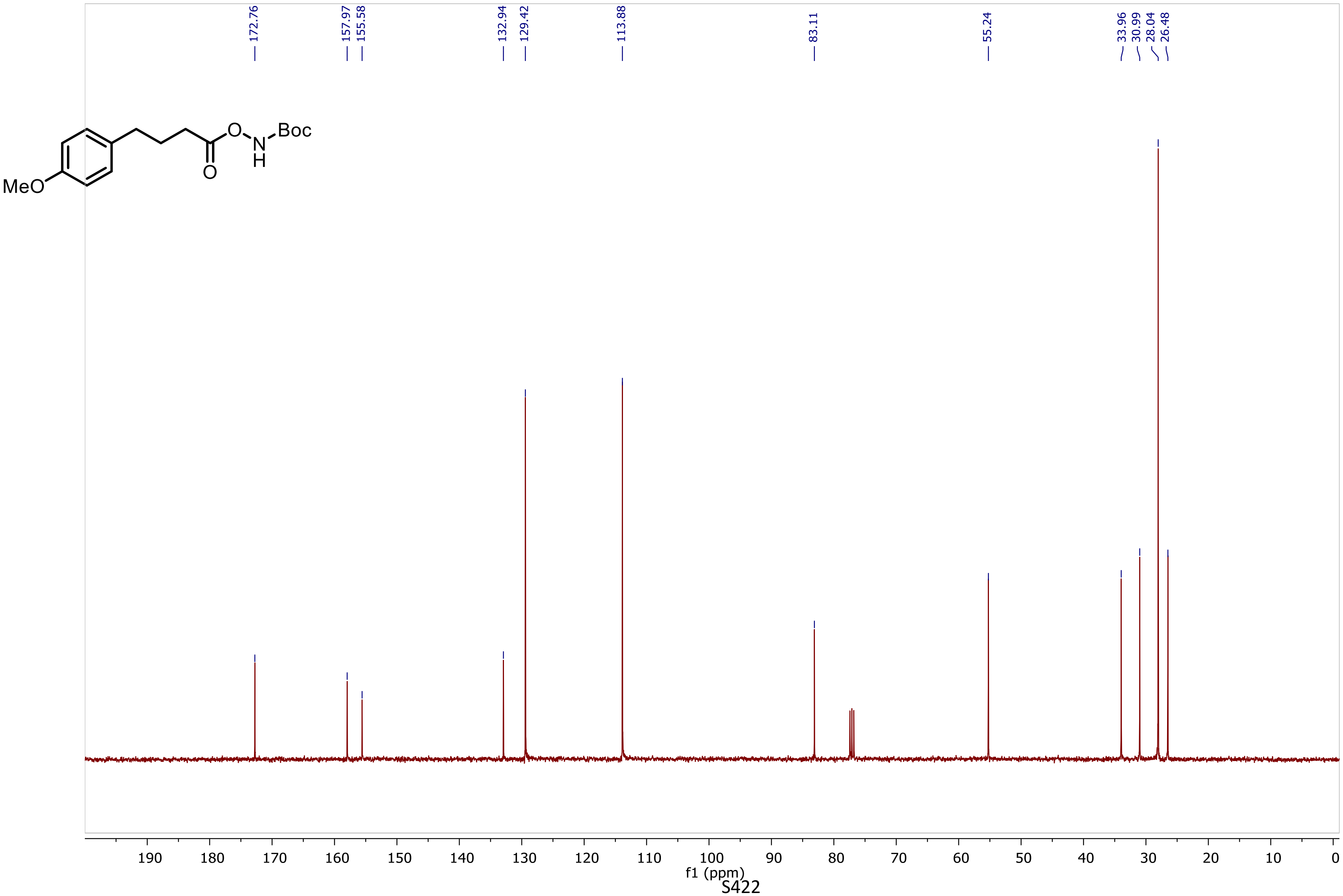

^{1}H NMR spectrum of 2-amino-4-methoxybenzoic acid **2ai** in MeOD-d_4

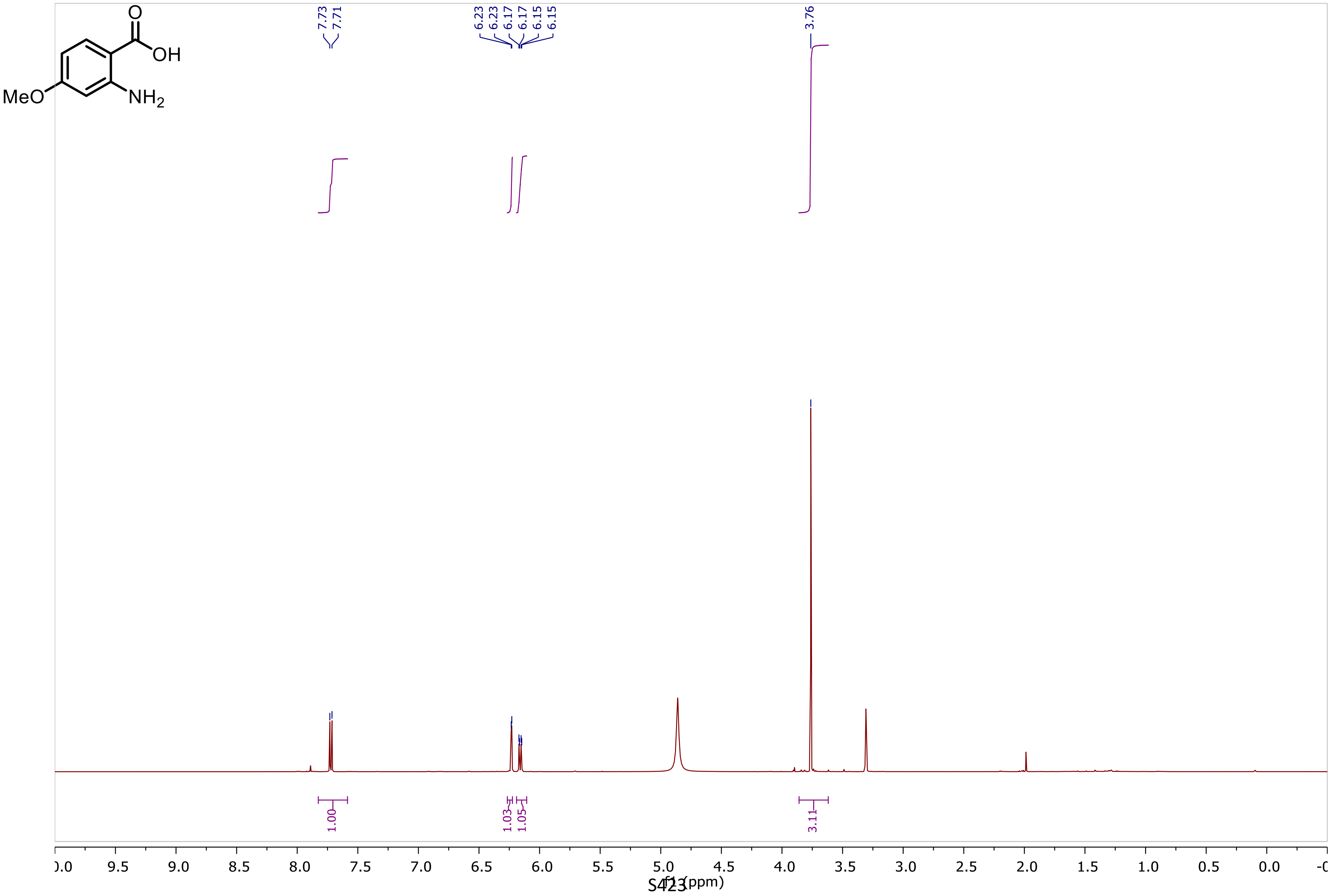

^{13}C NMR spectrum of 2-amino-4-methoxybenzoic acid **2ai** in MeOD-d_4

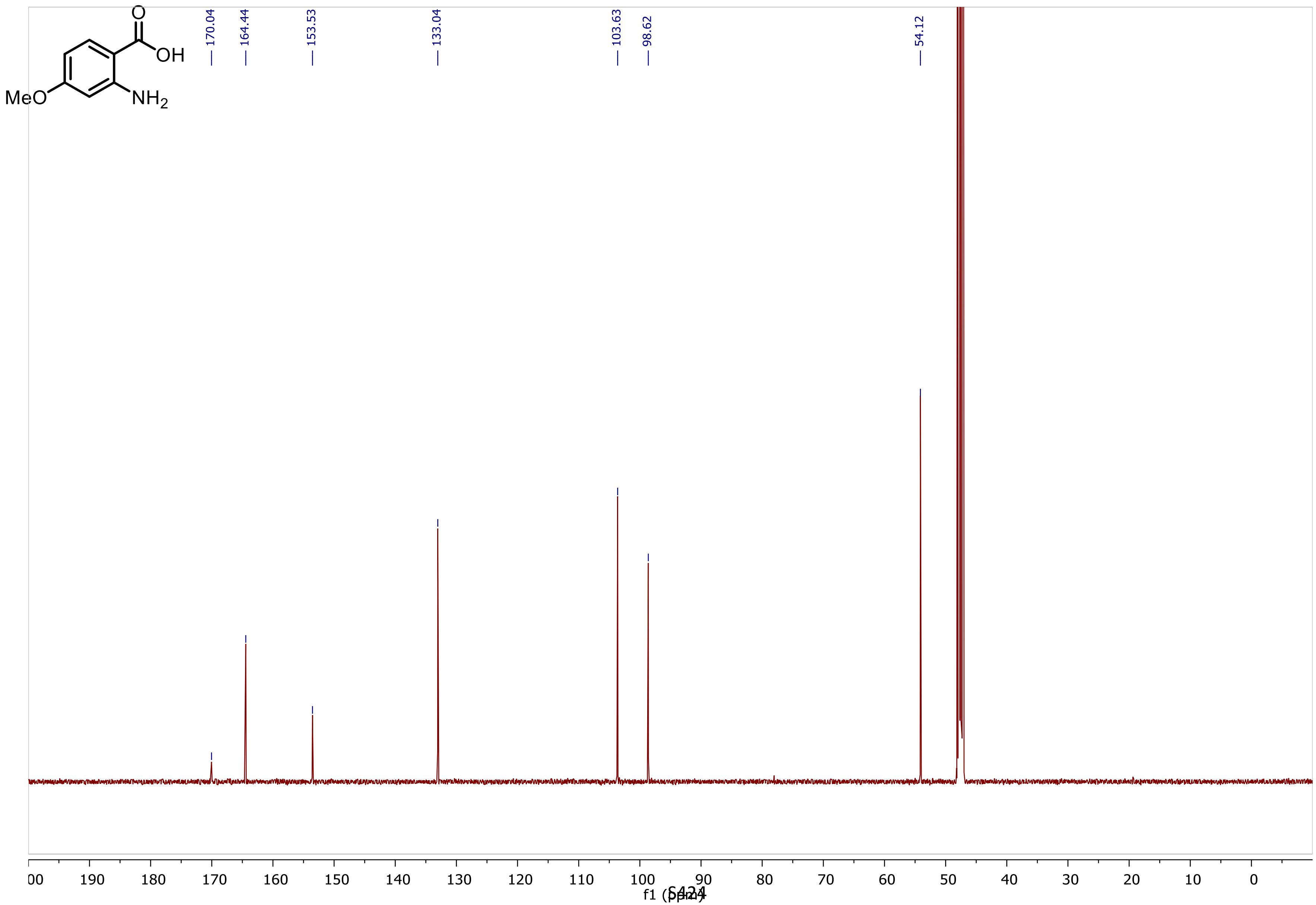

^{1}H NMR spectrum of 3-amino-4-methoxybenzoic acid ***iso*-2ai** in MeOD-d_4

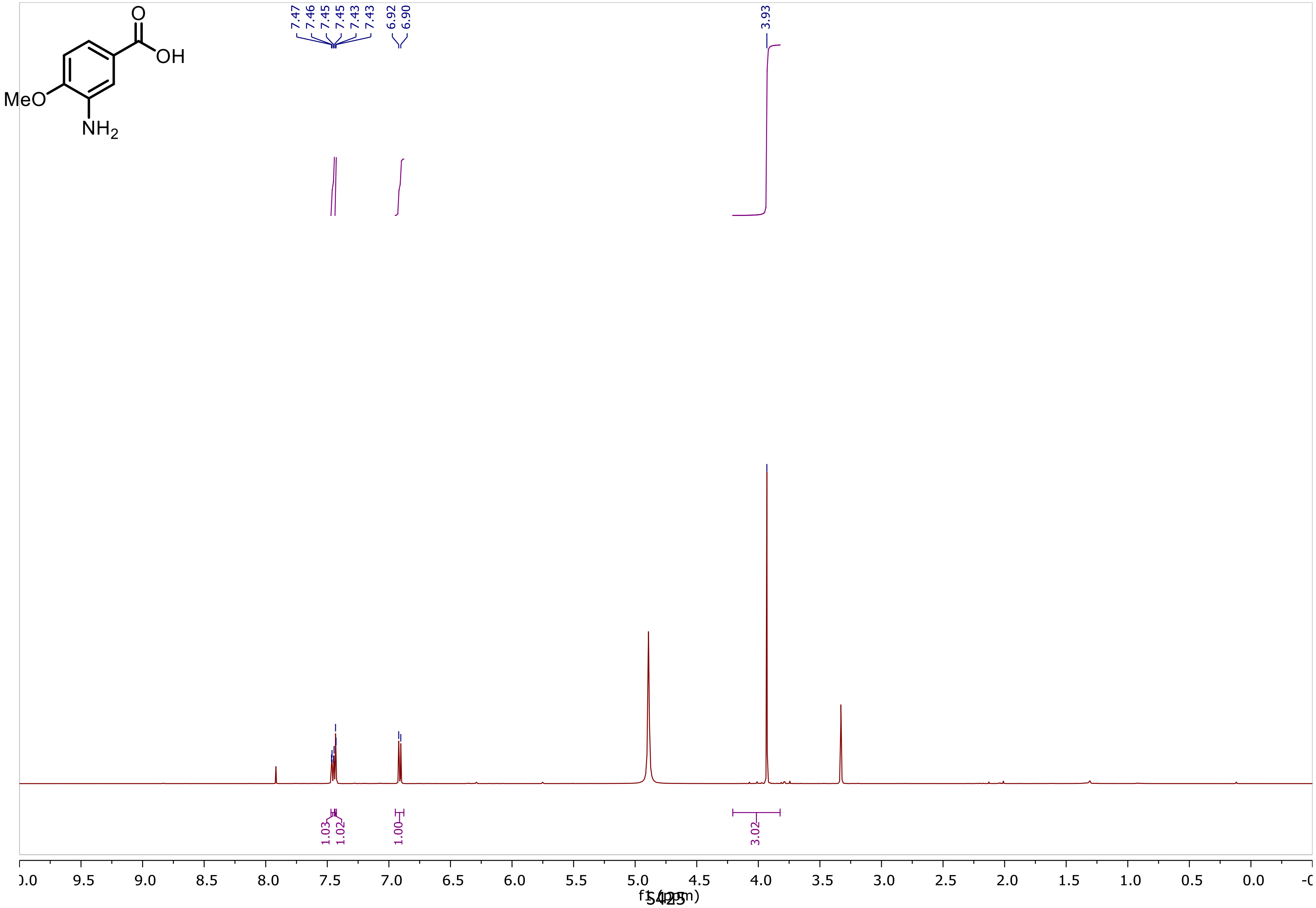

^{13}C NMR spectrum of 3-amino-4-methoxybenzoic acid ***iso*-2ai** in MeOD-d_4

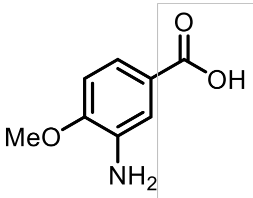

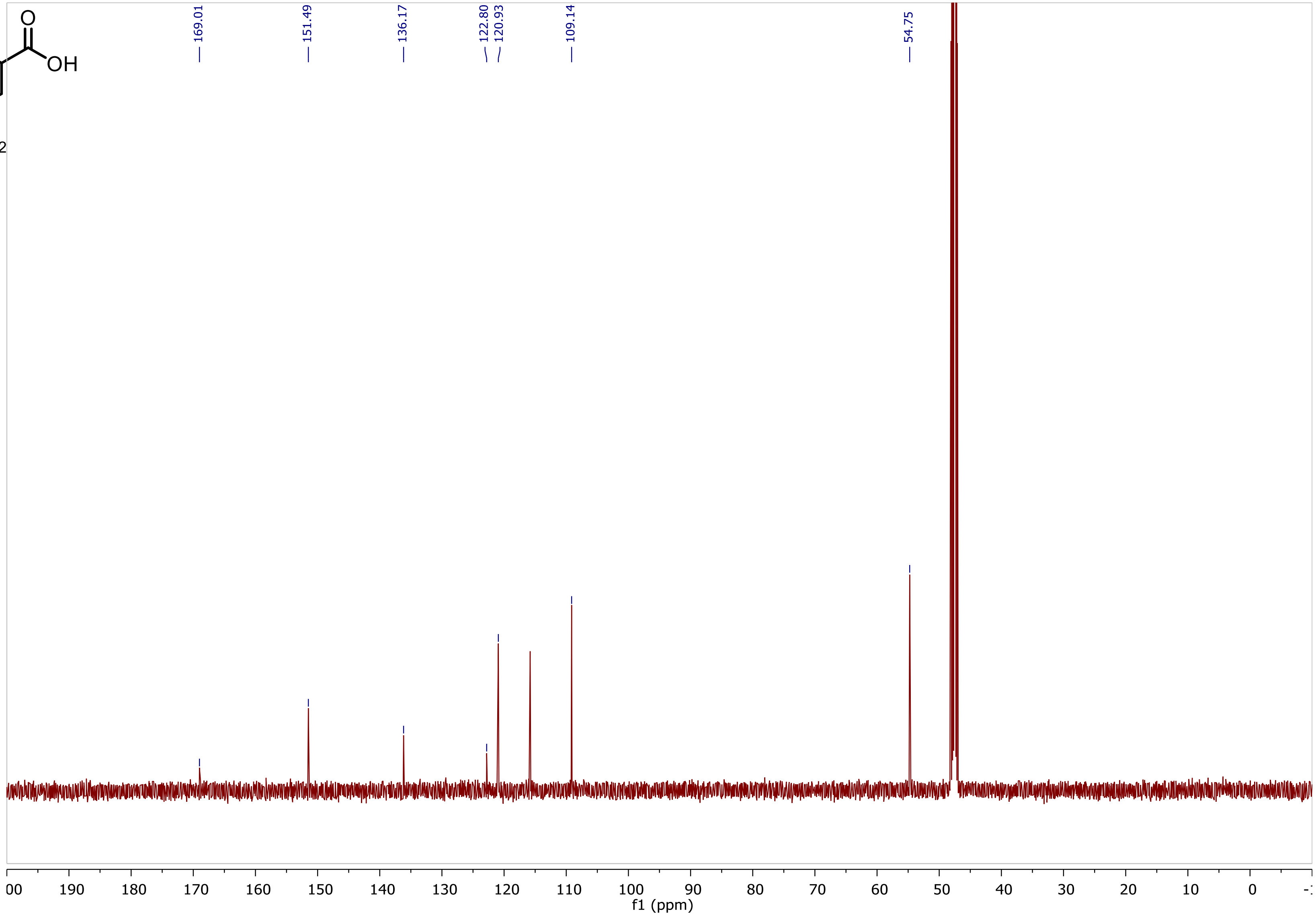

^{1}H NMR spectrum of 7-methoxy-3,4-dihydroquinolin-2(1*H*)-one **2ak** in MeOD-d_4

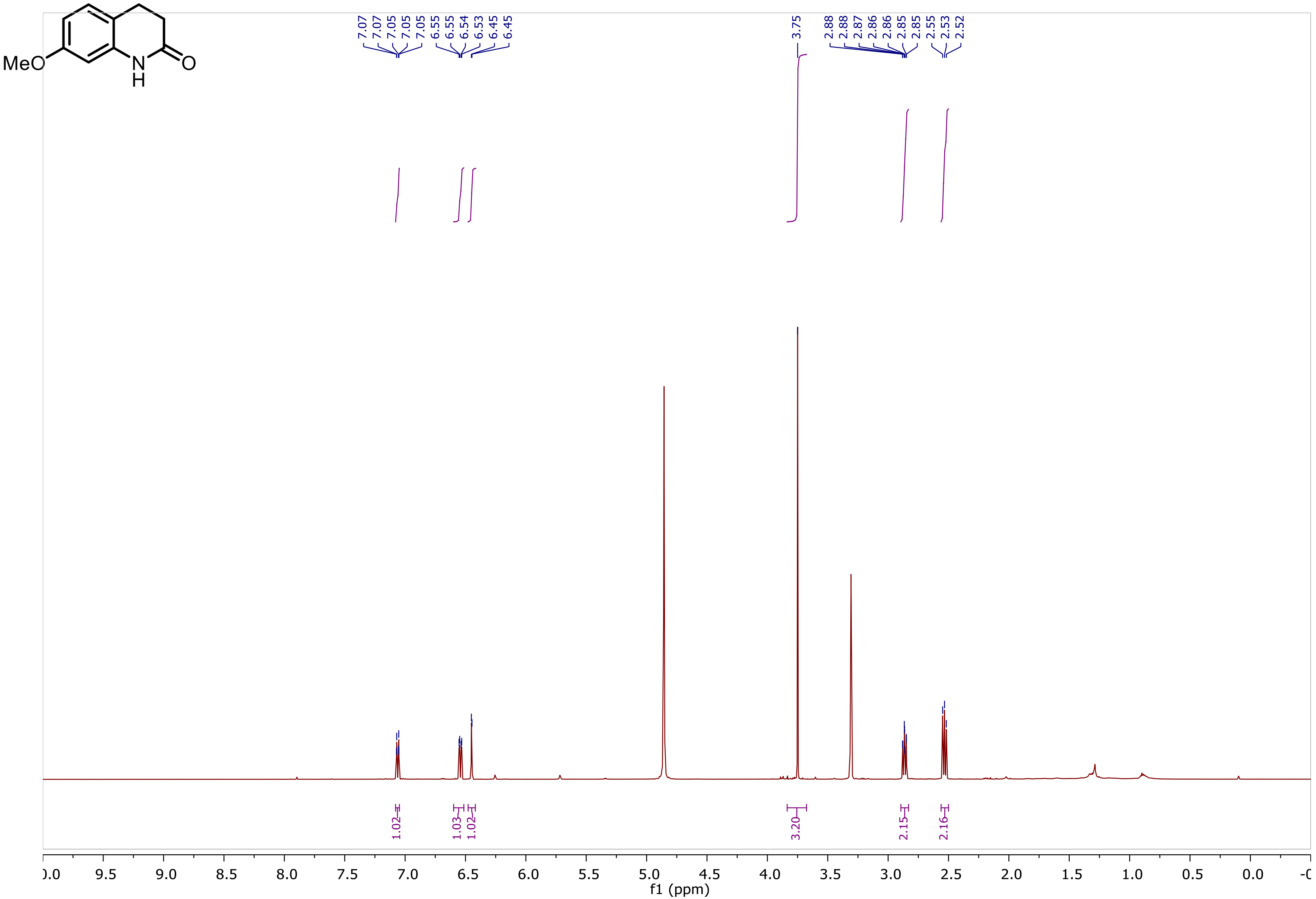

^{13}C NMR spectrum of 7-methoxy-3,4-dihydroquinolin-2(1*H*)-one **2ak** in MeOD-d_4

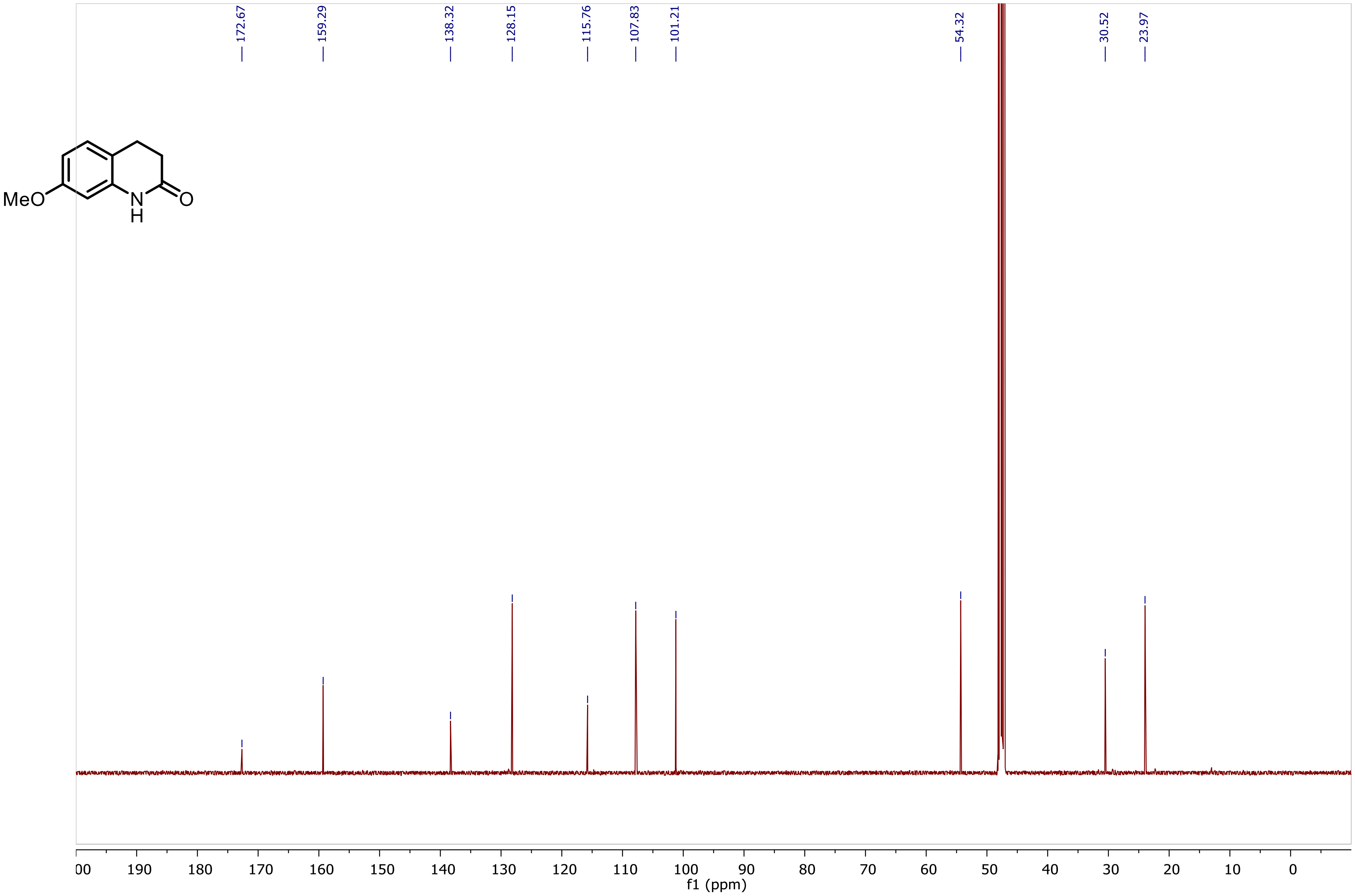

^{1}H NMR spectrum of 3-(3-amino-4-methoxyphenyl)propanoic acid **S2ak** in MeOD-d_4

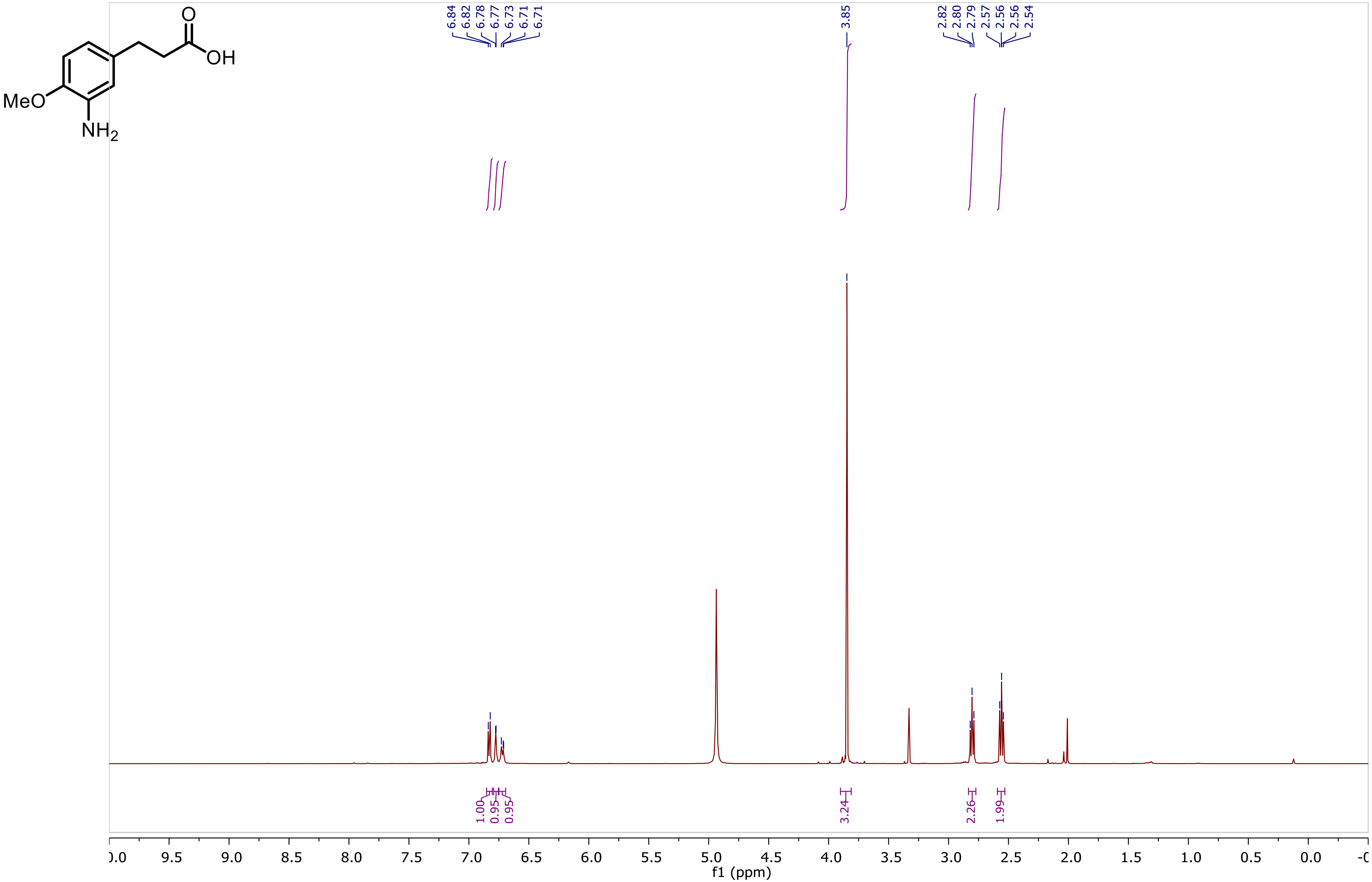

^{13}C NMR spectrum of 3-(3-amino-4-methoxyphenyl)propanoic acid **S2ak** in MeOD-d_4

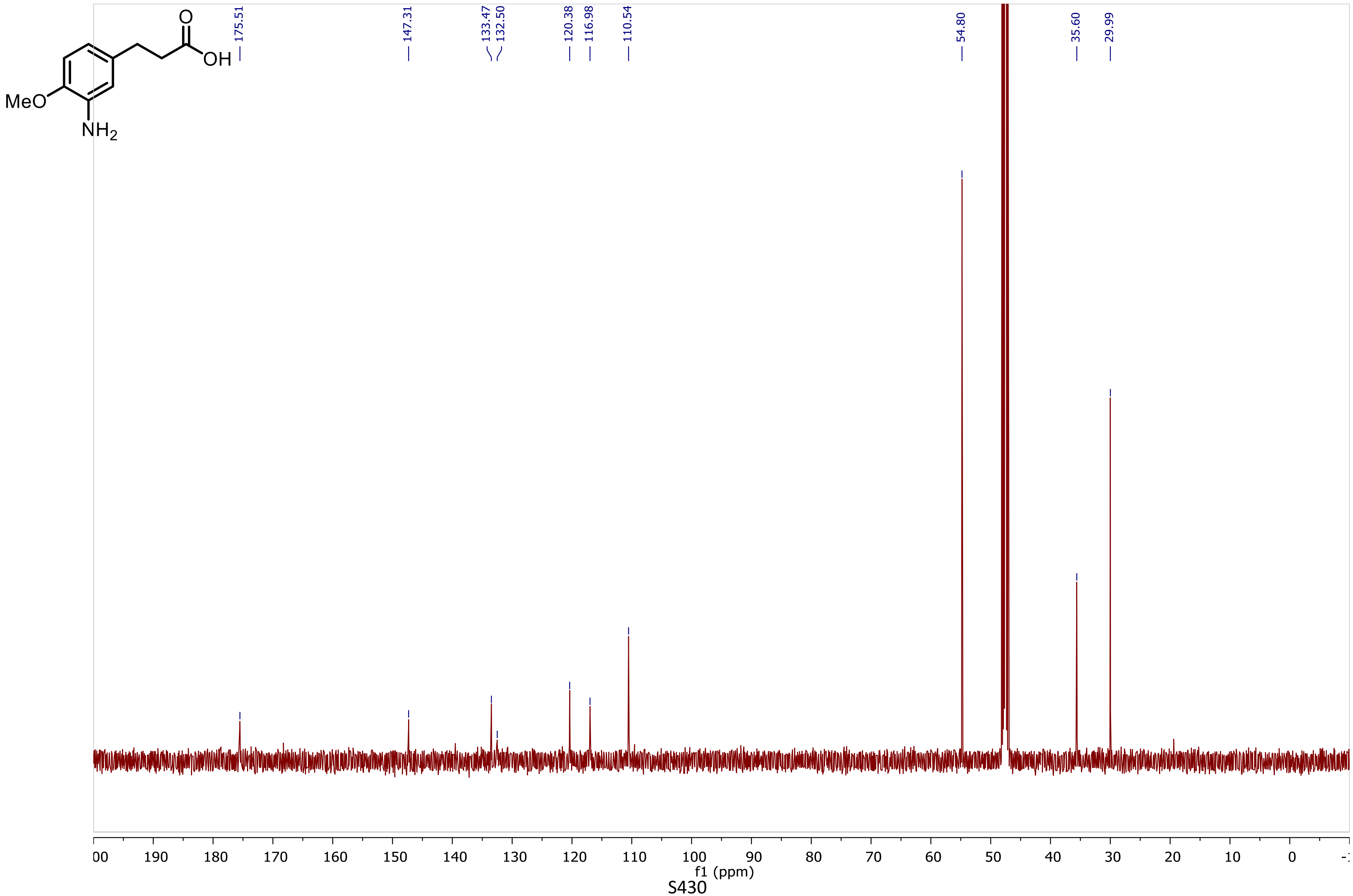

^{1}H NMR spectrum of 4-(3-amino-4-methoxyphenyl)butanoic acid **2al** in DMSO-d_6

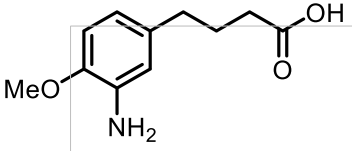

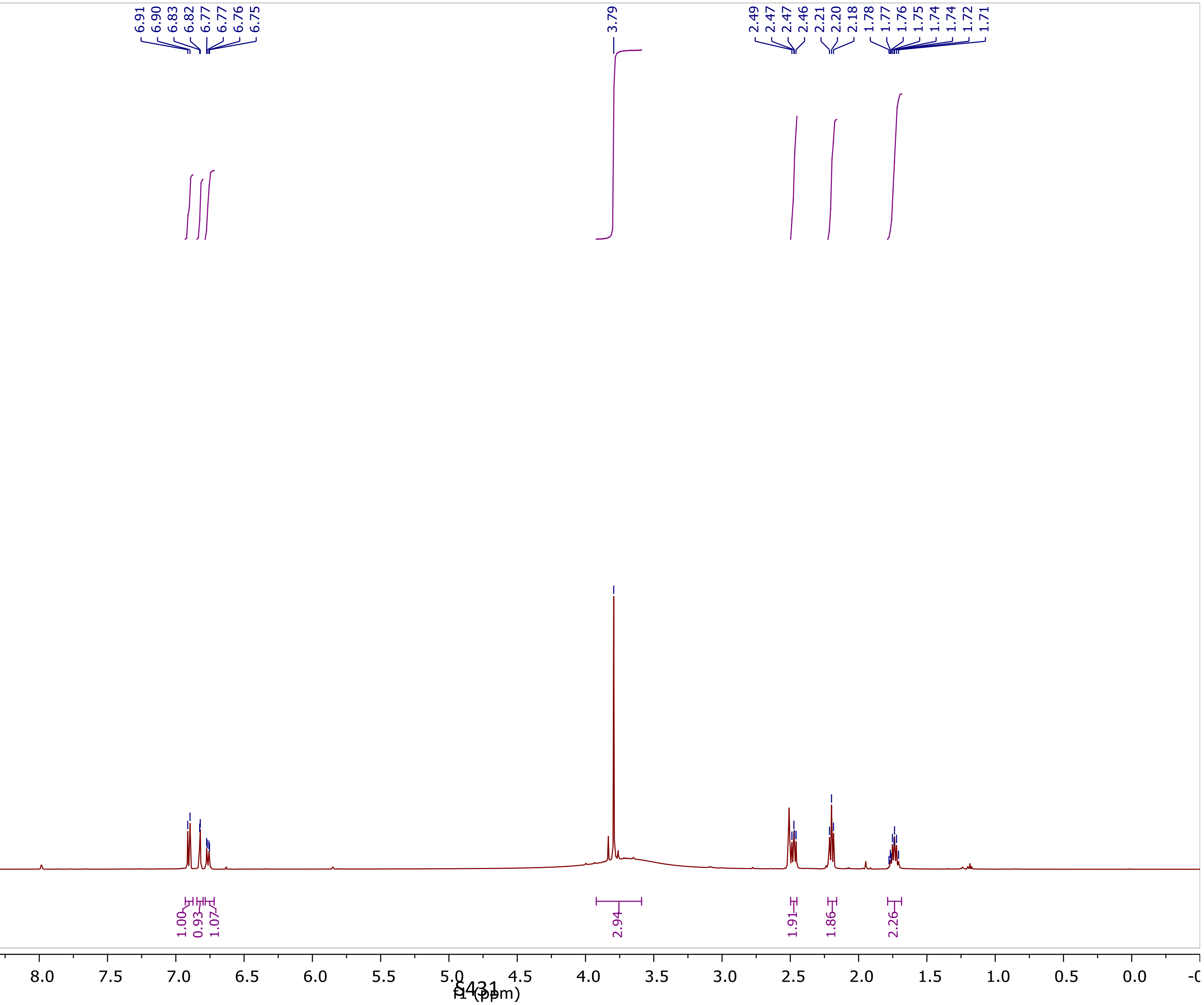

^{13}C NMR spectrum of 4-(3-amino-4-methoxyphenyl)butanoic acid **2al** in DMSO-d_6

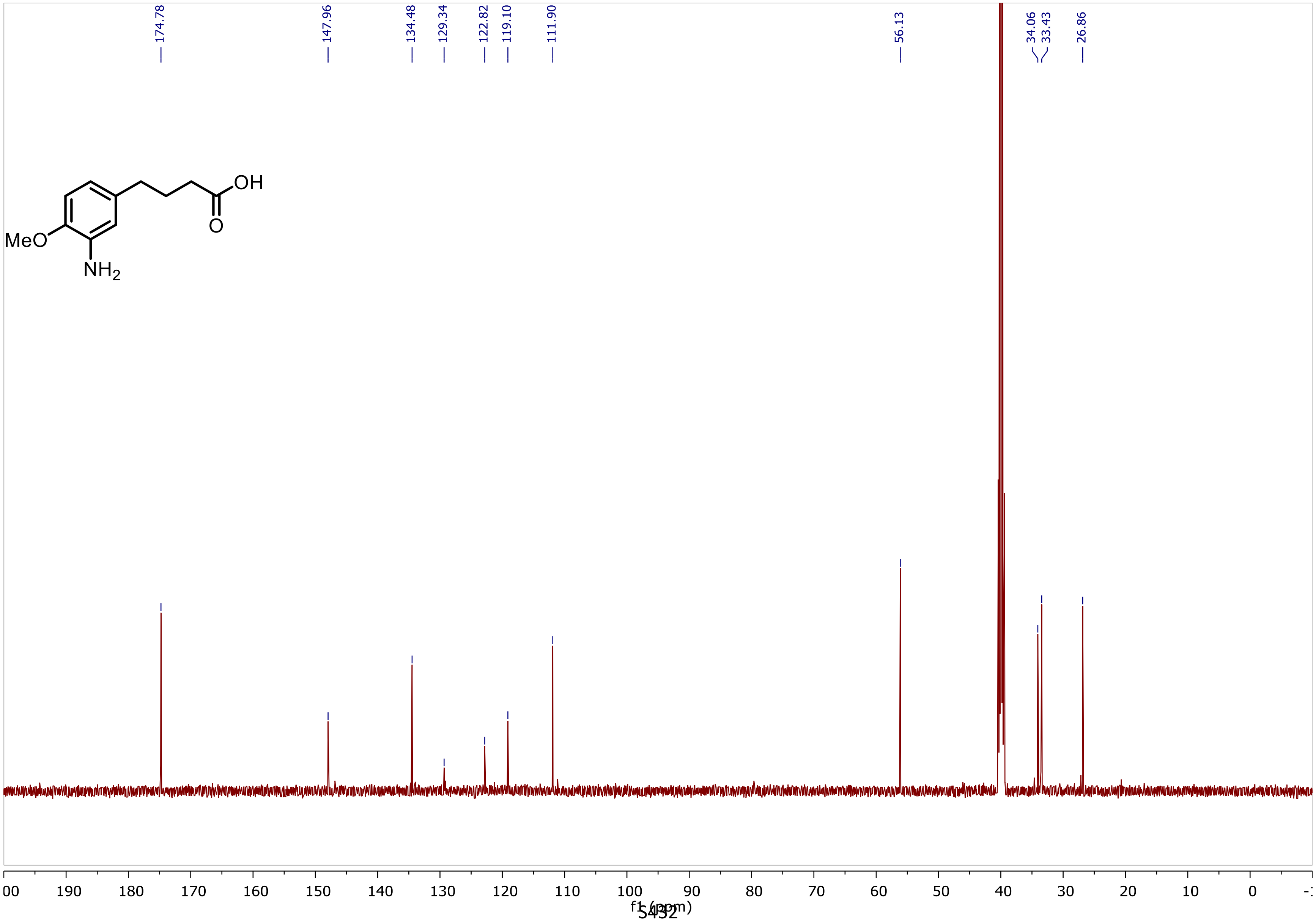

^{1}H NMR spectrum of 3,5-dimethoxy-2-(methylamino)benzoic acid **4b’** in MeOD-d_4

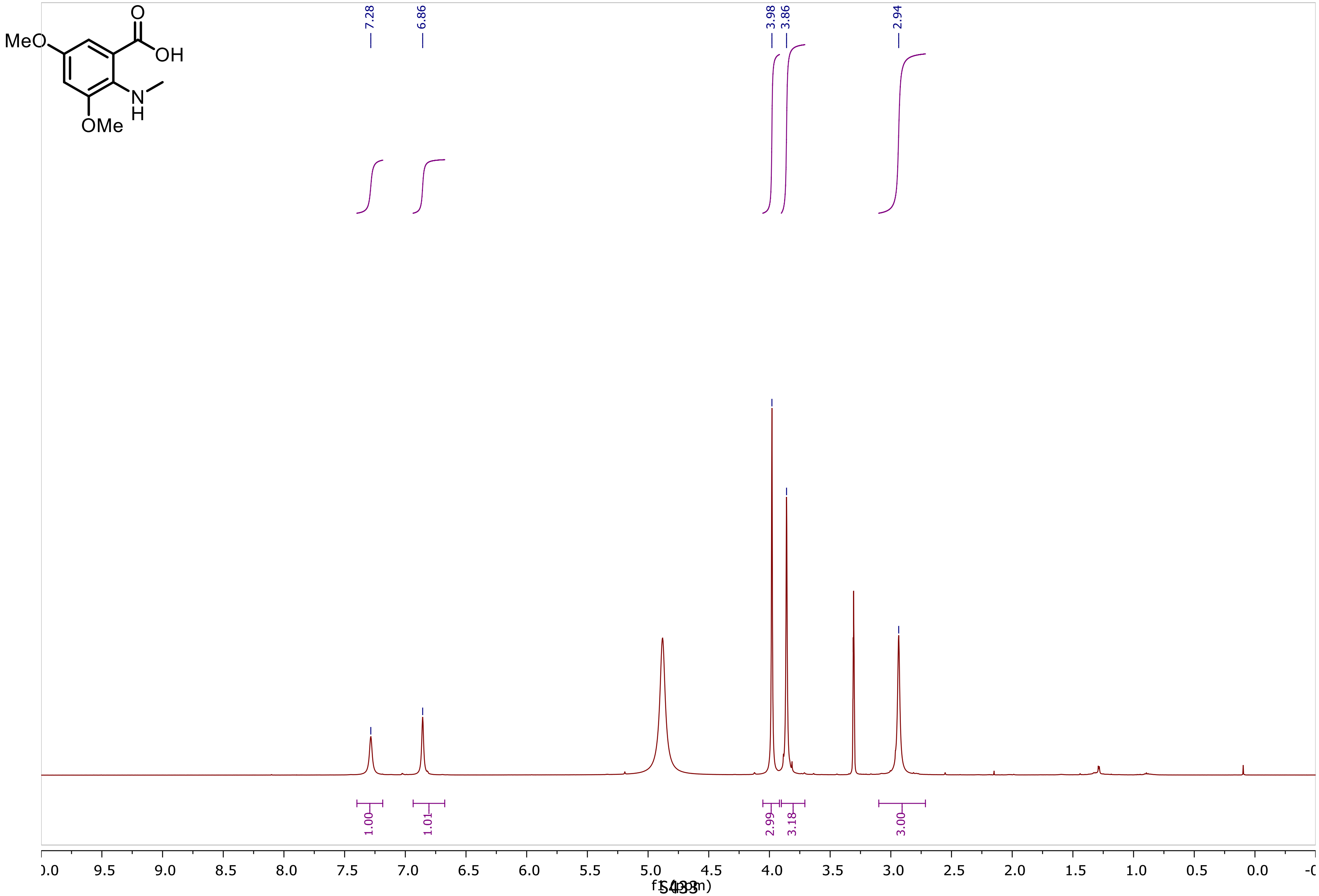

^{13}C NMR spectrum of 3,5-dimethoxy-2-(methylamino)benzoic acid **4b'** in MeOD-d_4

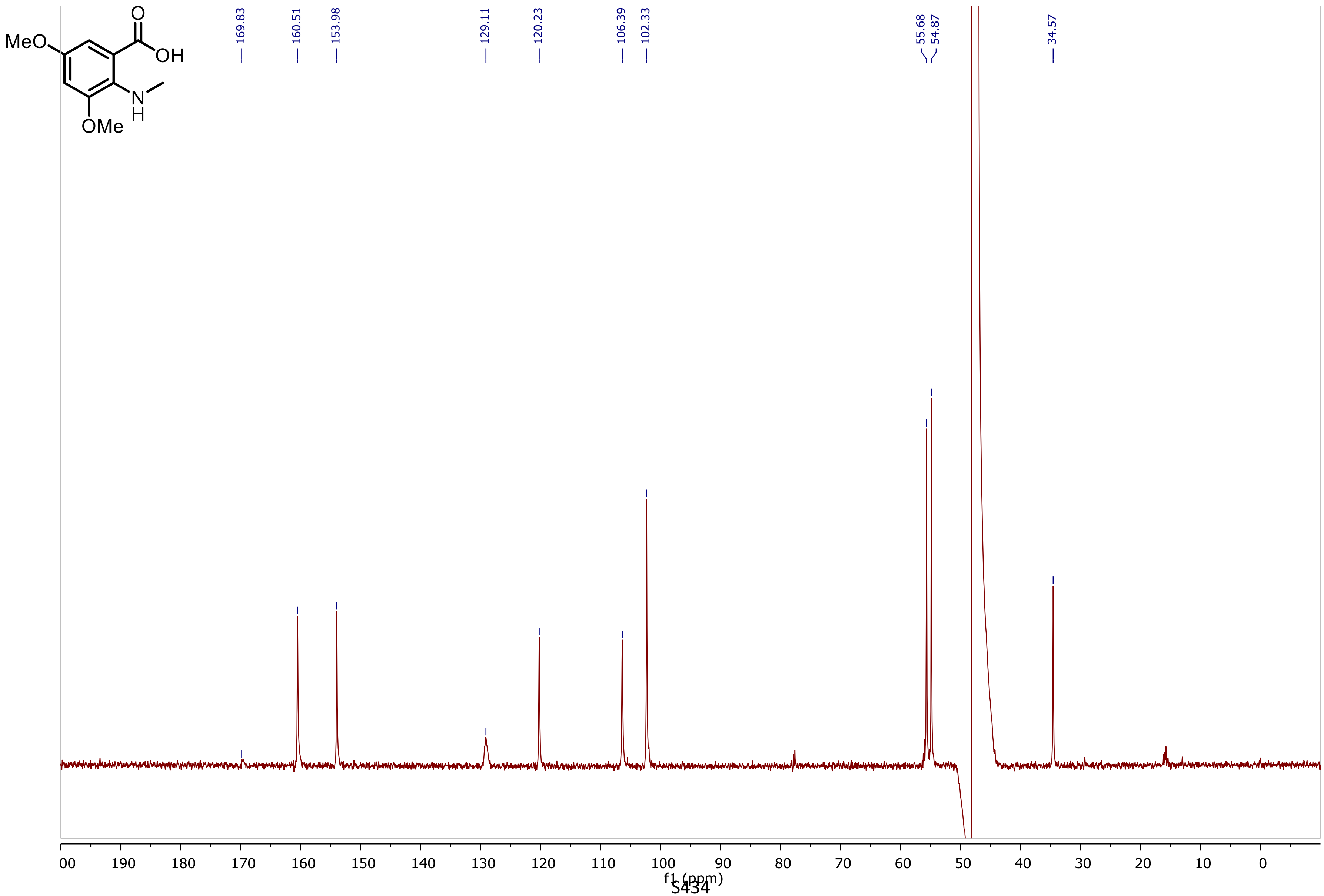

^1H NMR spectrum of 2-amino-3,5-dimethoxybenzoic acid **2ac** in MeOD-d_4

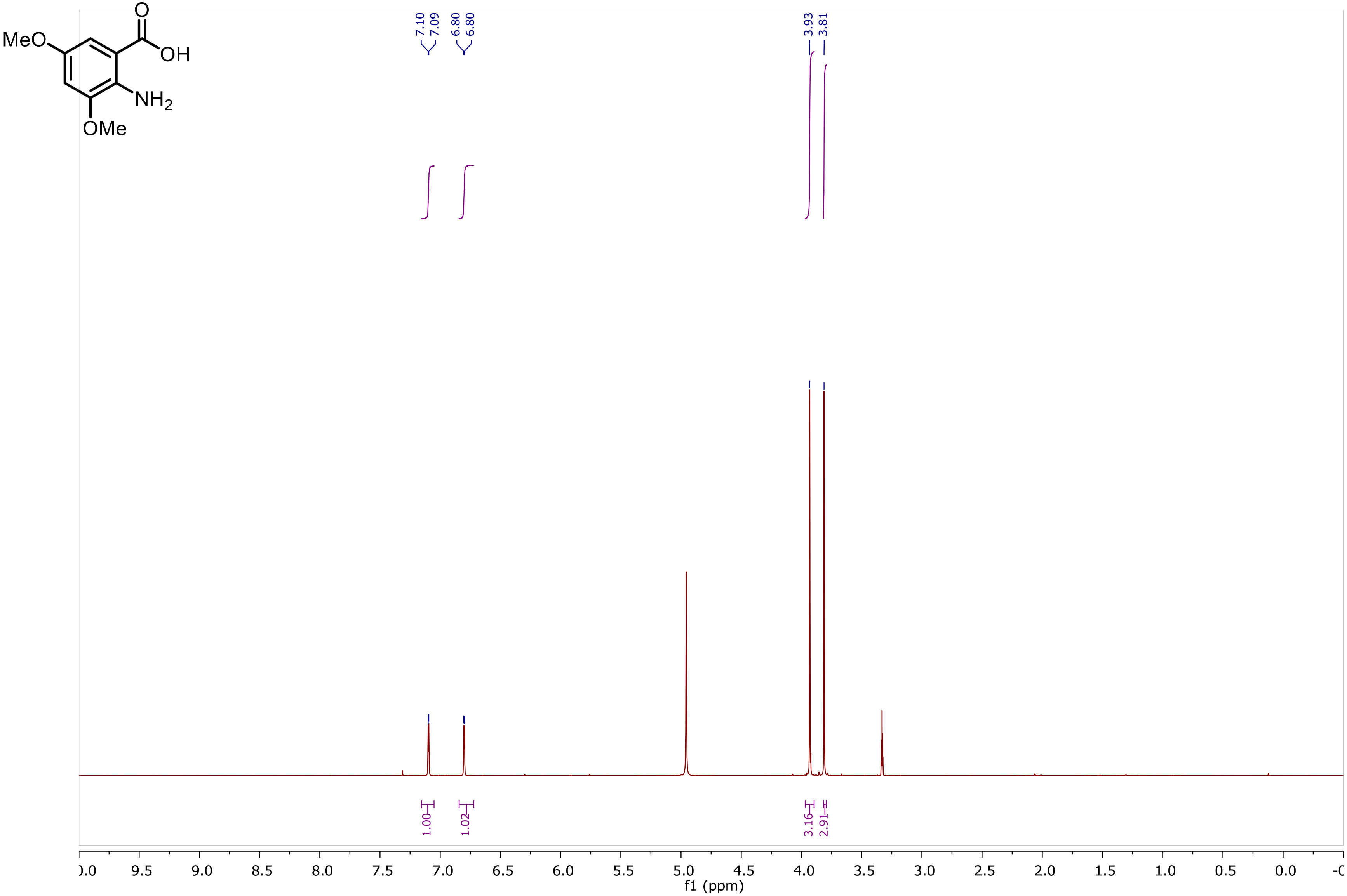

^{13}C NMR spectrum of 2-amino-3,5-dimethoxybenzoic acid **2ac** in MeOD-d_4

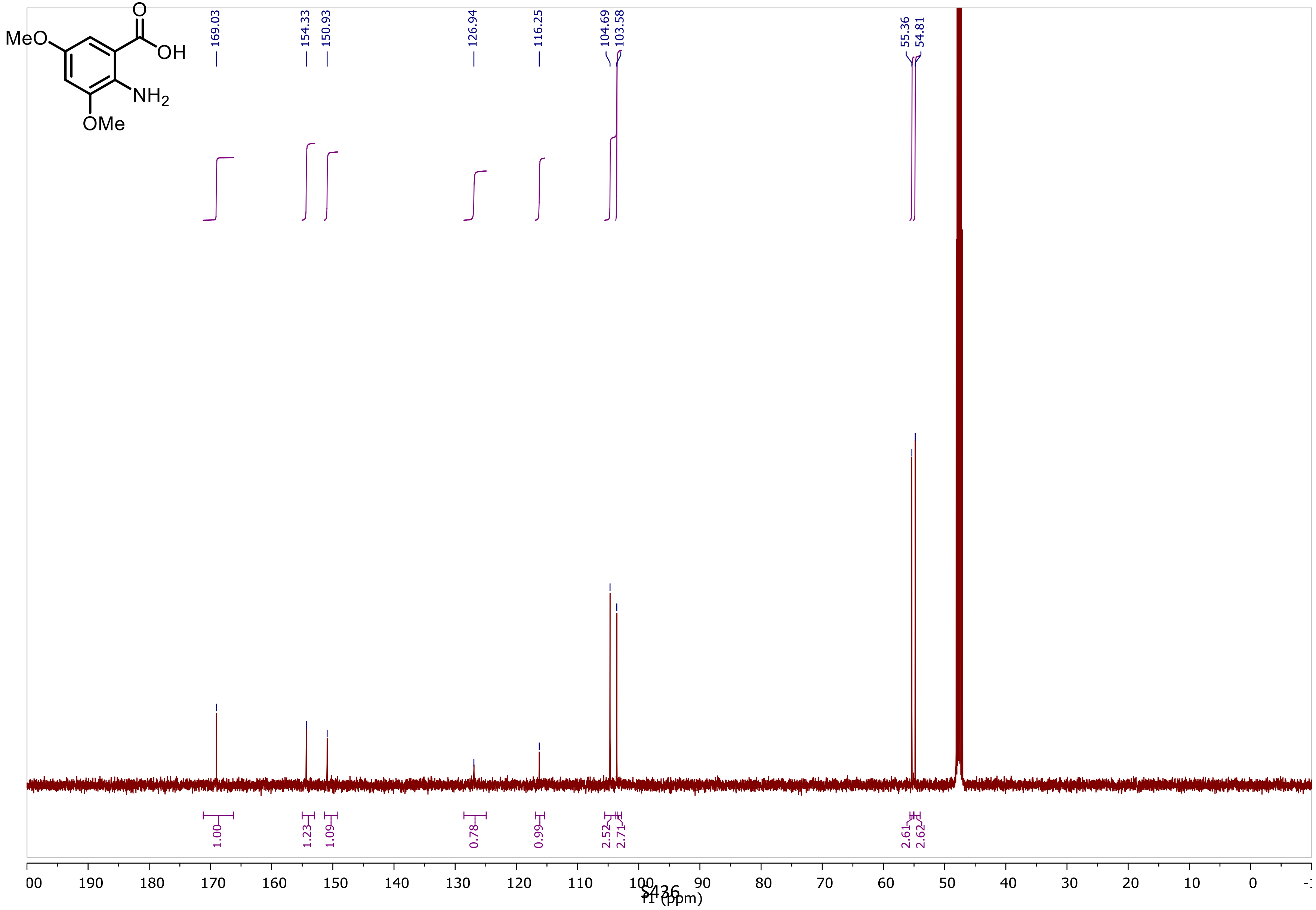

^{1}H NMR spectrum of 4-methyl-2-(methylamino)benzoic acid **4j** in MeOD-d_4

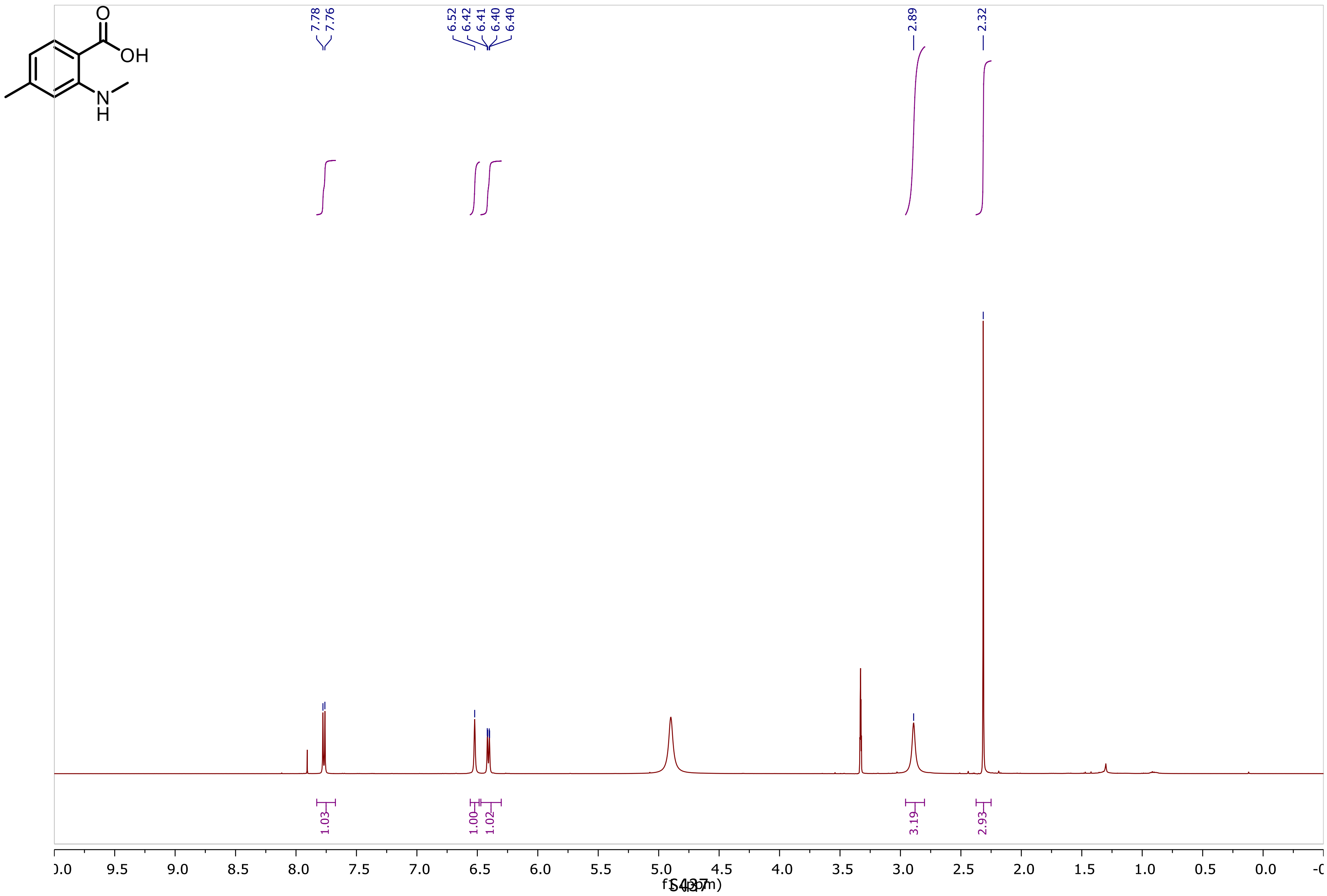

^{13}C NMR spectrum of 4-methyl-2-(methylamino)benzoic acid **4j** in MeOD-d_4

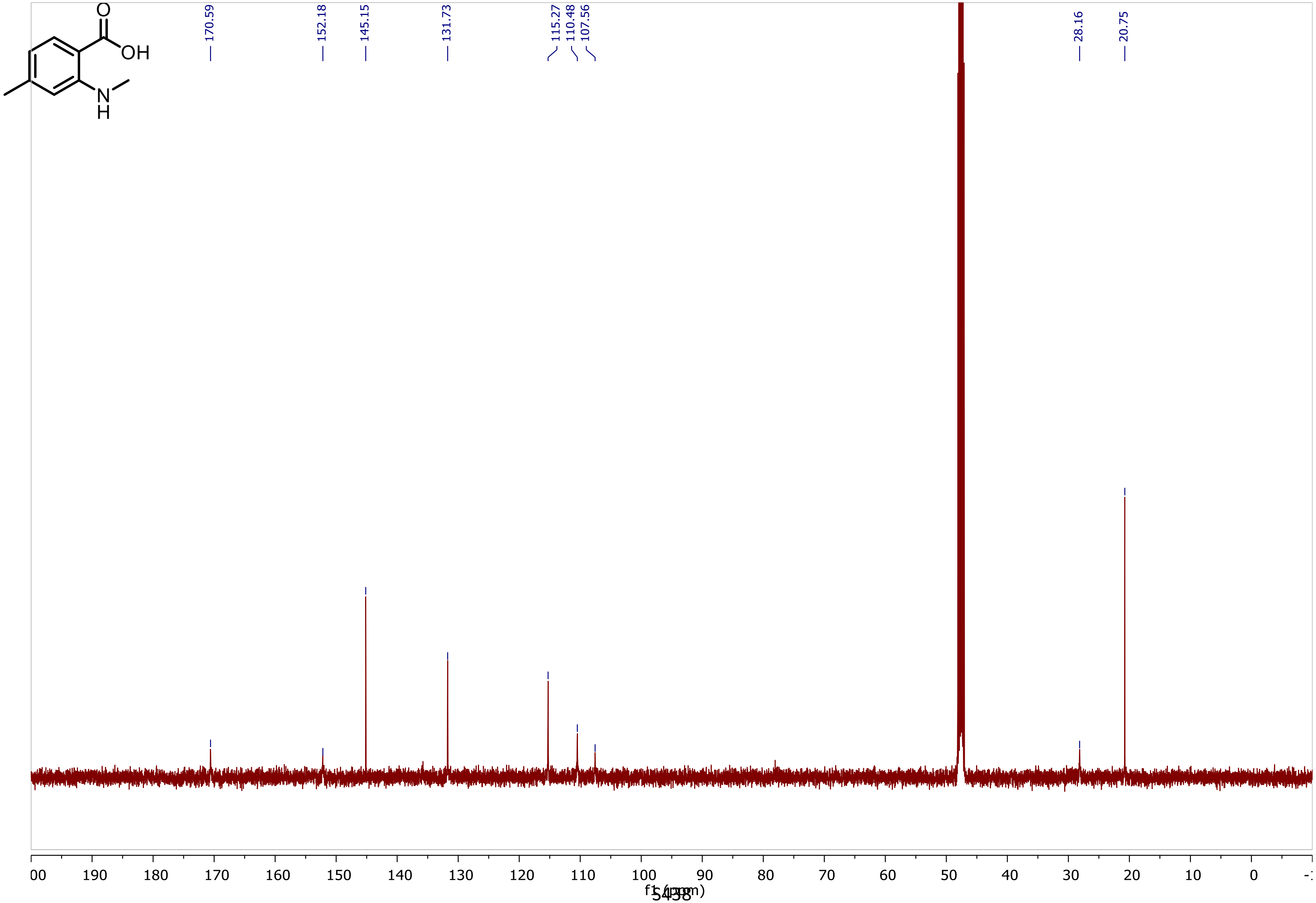

^{1}H NMR spectrum of 2-amino-4-methylbenzoic acid **2b'** in MeOD-d_4

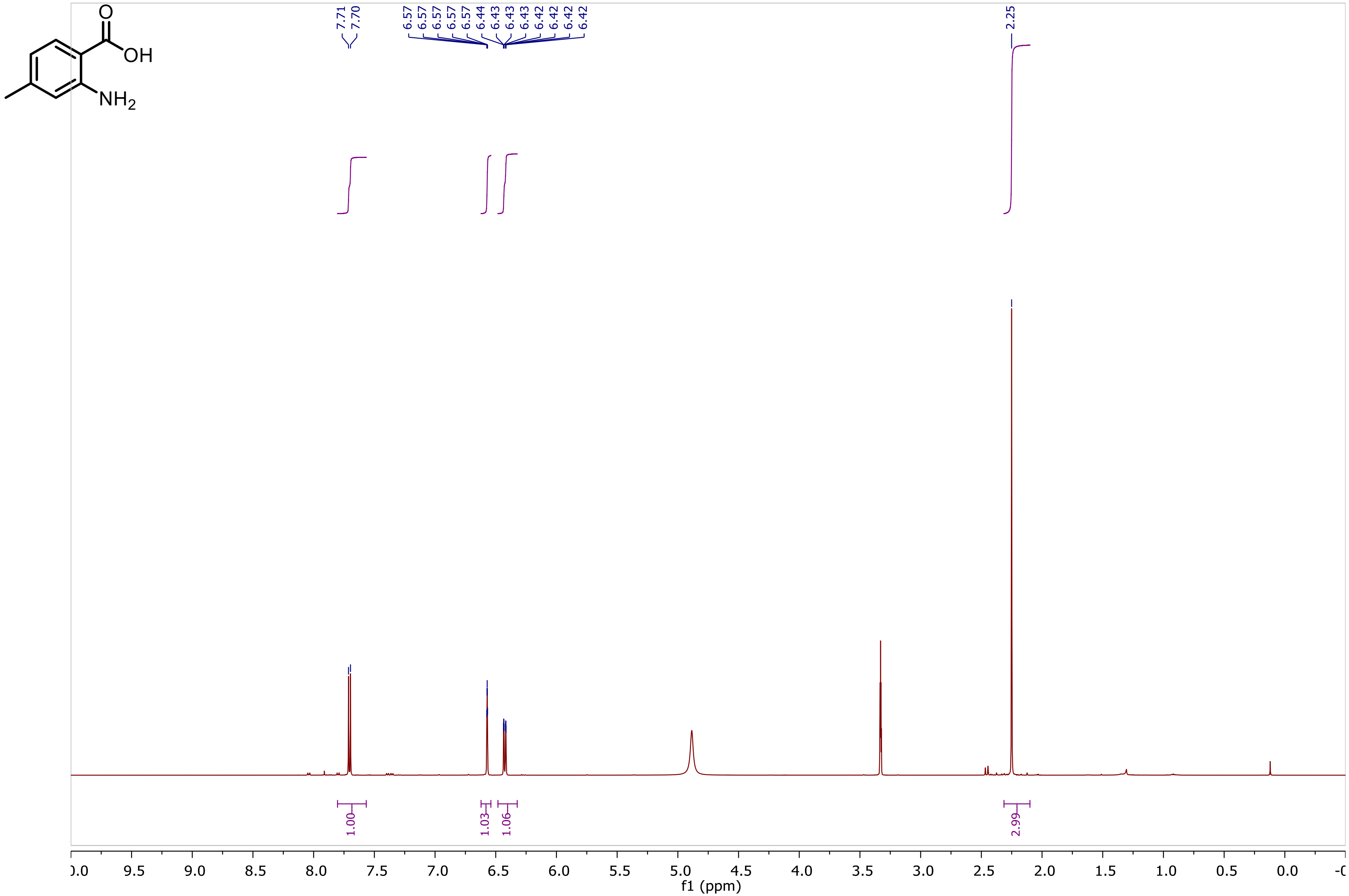

^{13}C NMR spectrum of 2-amino-4-methylbenzoic acid **2b'** in MeOD-d_4

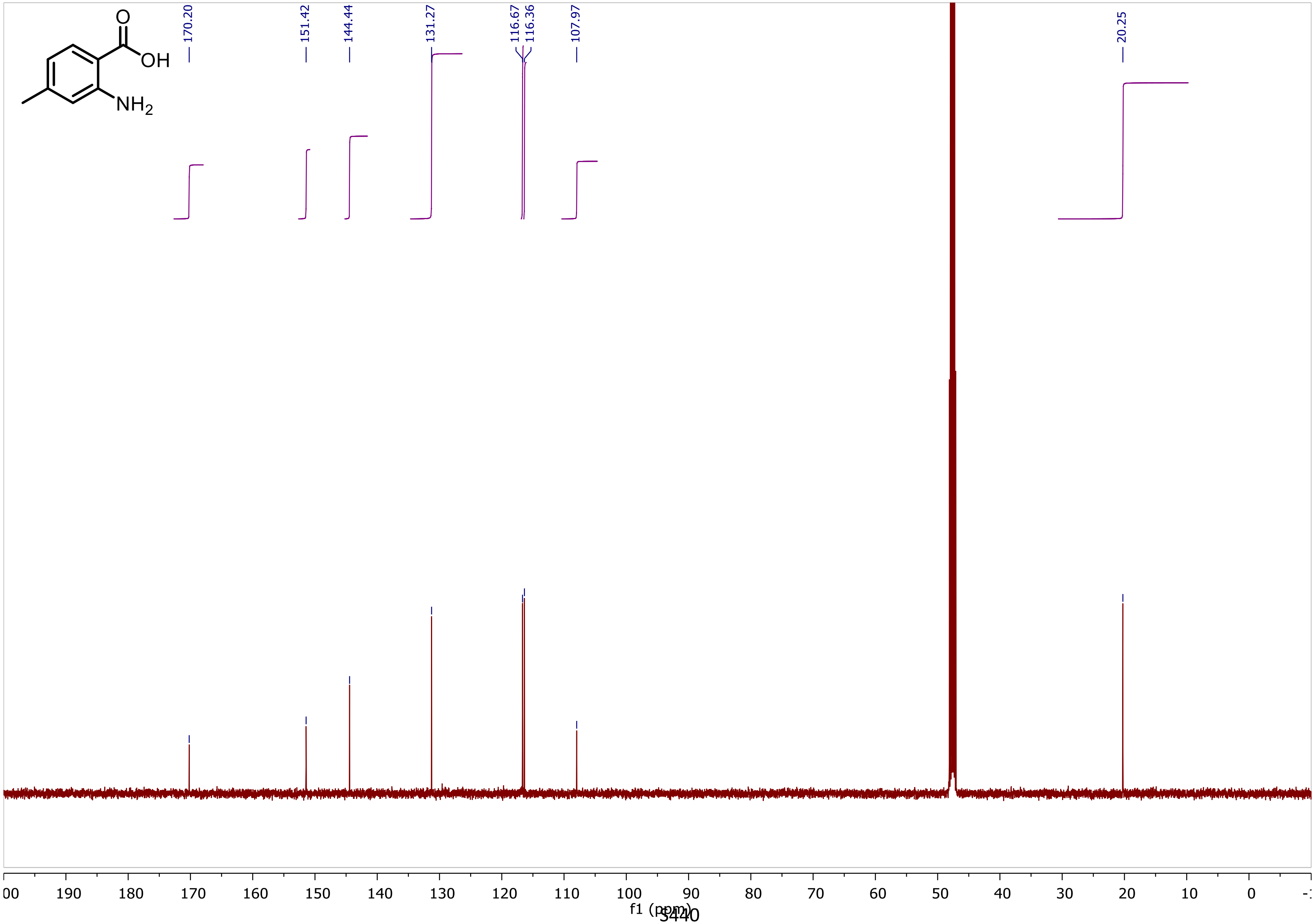

^{1}H NMR spectrum of 2-amino-3,5-dimethylbenzoic acid **2f’** in MeOD-d_4

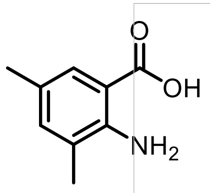

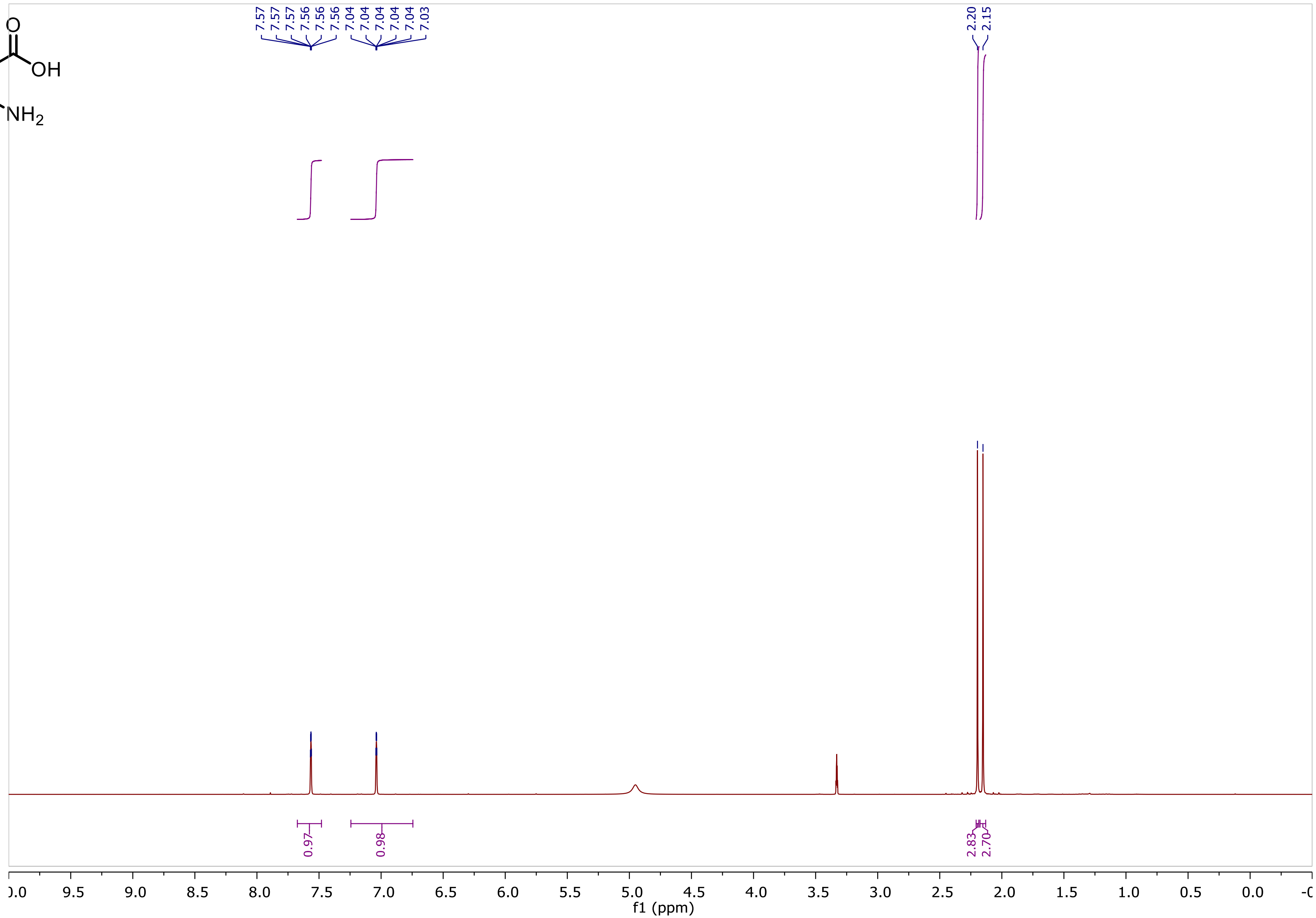

^{13}C NMR spectrum of 2-amino-3,5-dimethylbenzoic acid **2f’** in MeOD-d_4

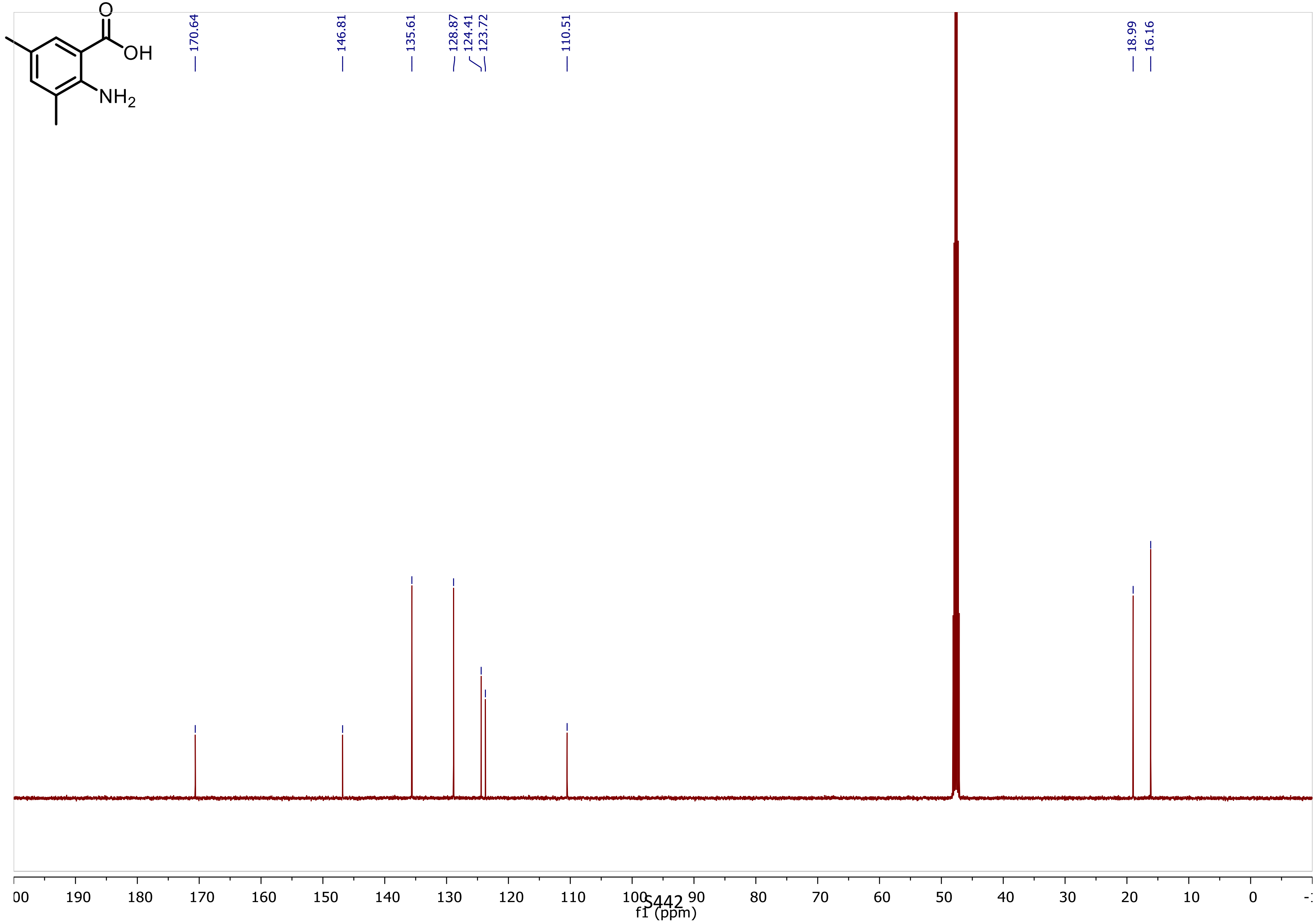

^{1}H NMR spectrum of 3,5-dimethyl-2-(methylamino)benzoic acid **4i'** in MeOD-d_4

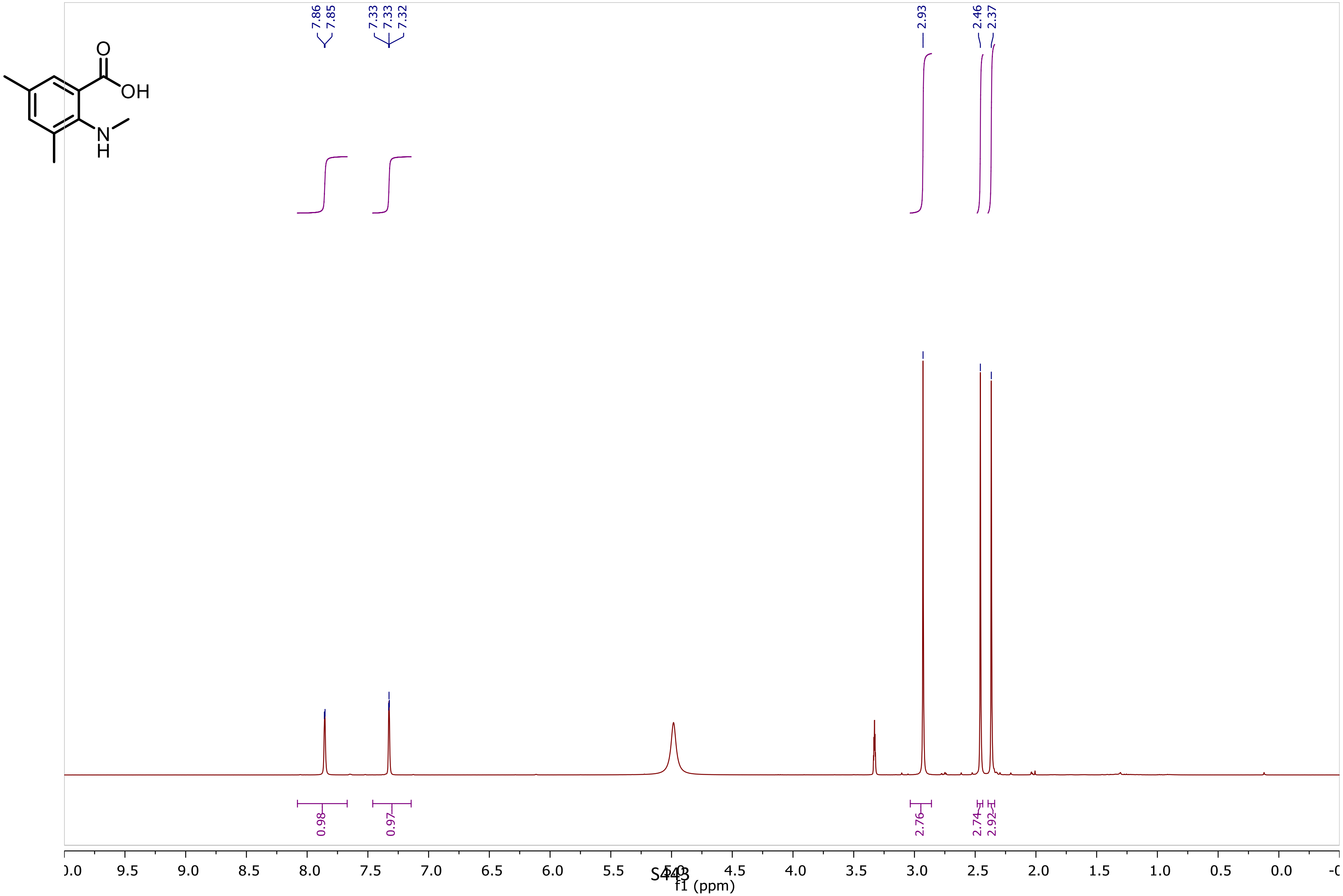

^{13}C NMR spectrum of 3,5-dimethyl-2-(methylamino)benzoic acid **4i'** in MeOD-d_4

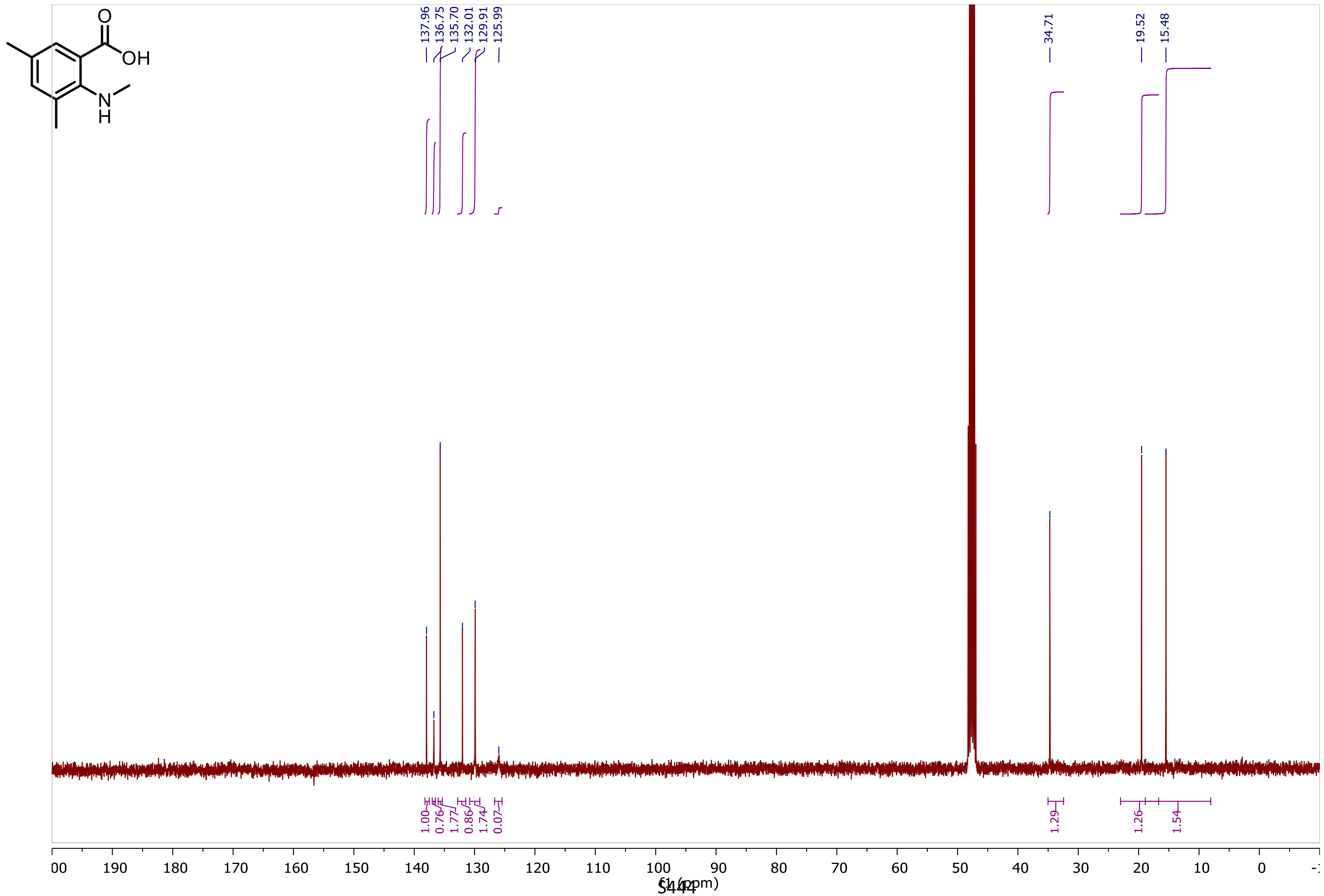

Reaction Utility

^{1}H NMR spectrum of 2-aminobenzoic acid **2a** in DMSO-d_6 (Telescoped procedure)

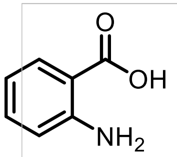

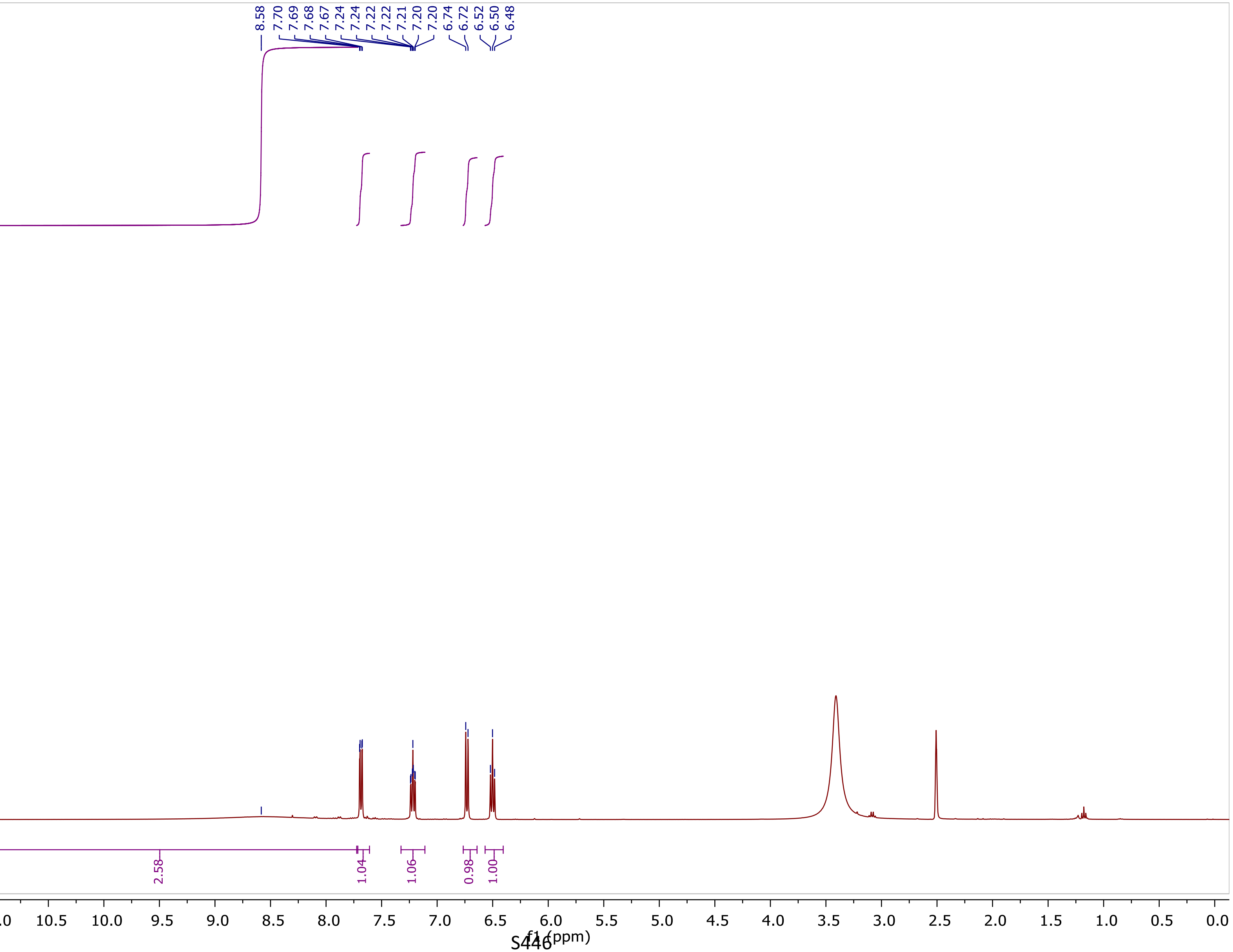

^{13}C NMR spectrum of 2-aminobenzoic acid **2a** in DMSO-d_6 (Telescoped procedure)

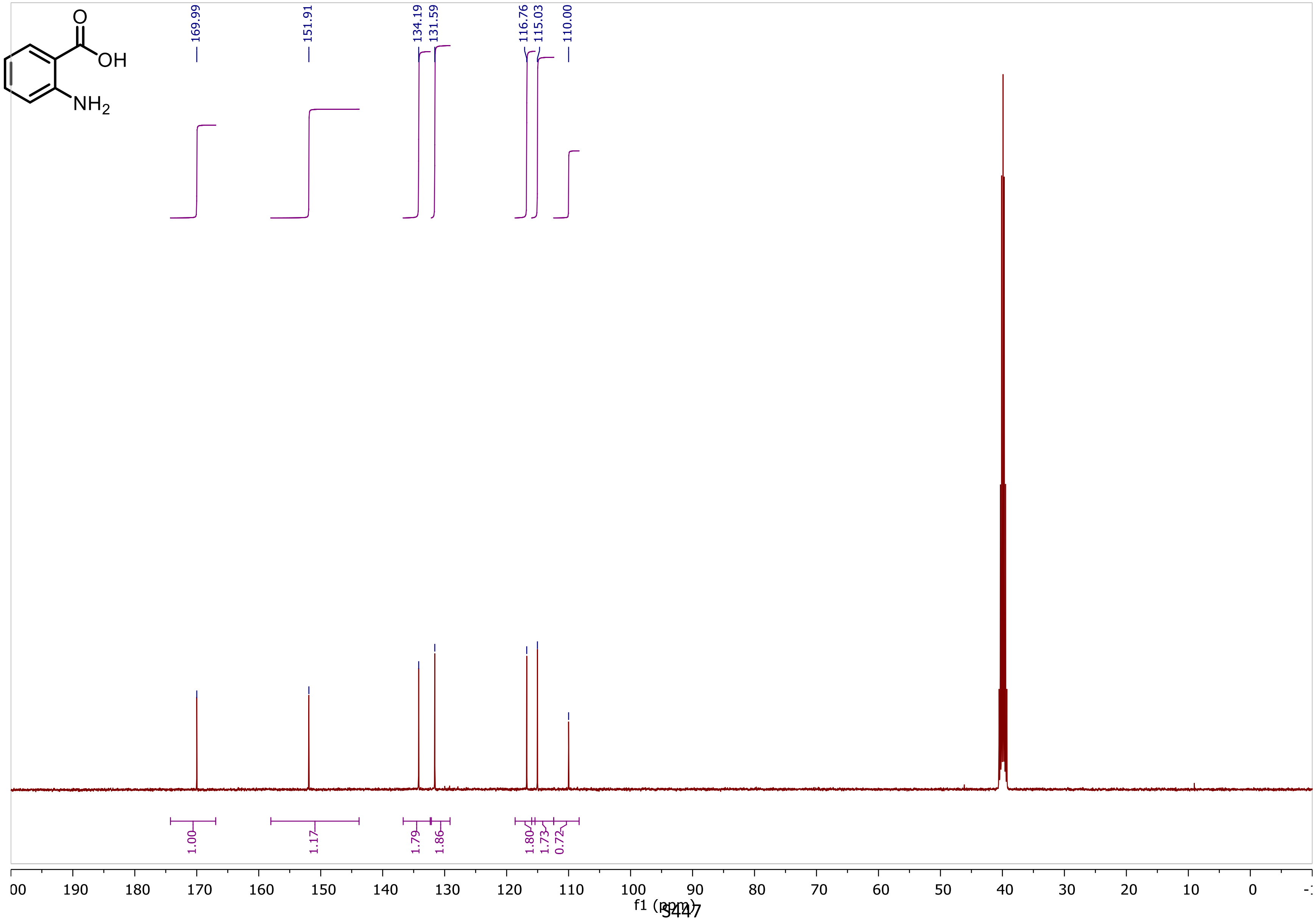

^{1}H NMR spectrum of 2-aminobenzoic acid **2a** in MeOH-d_4 (Scaled up procedure)

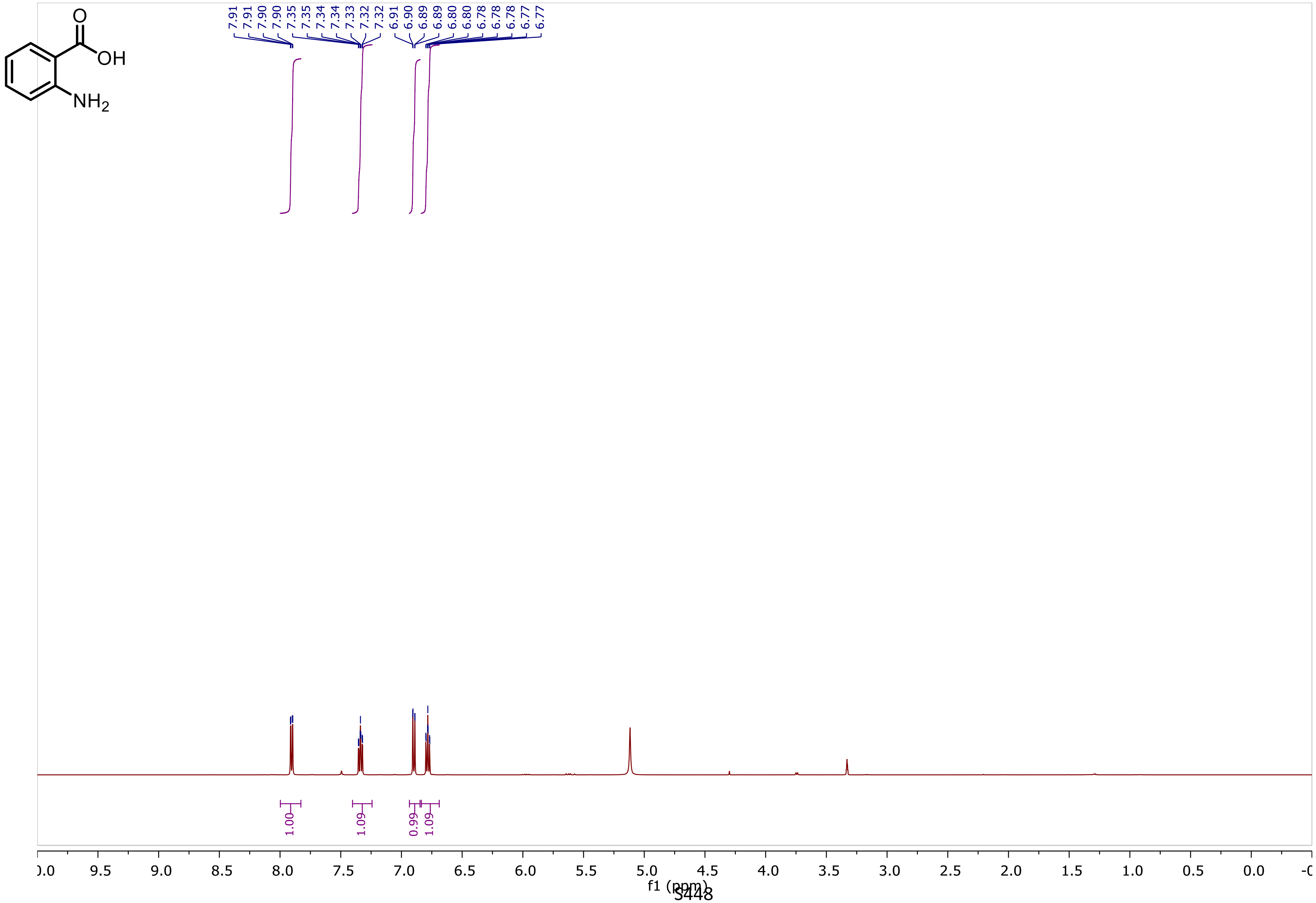

^{13}C NMR spectrum of 2-aminobenzoic acid **2a** in MeOH-d_4 (Scaled up procedure)

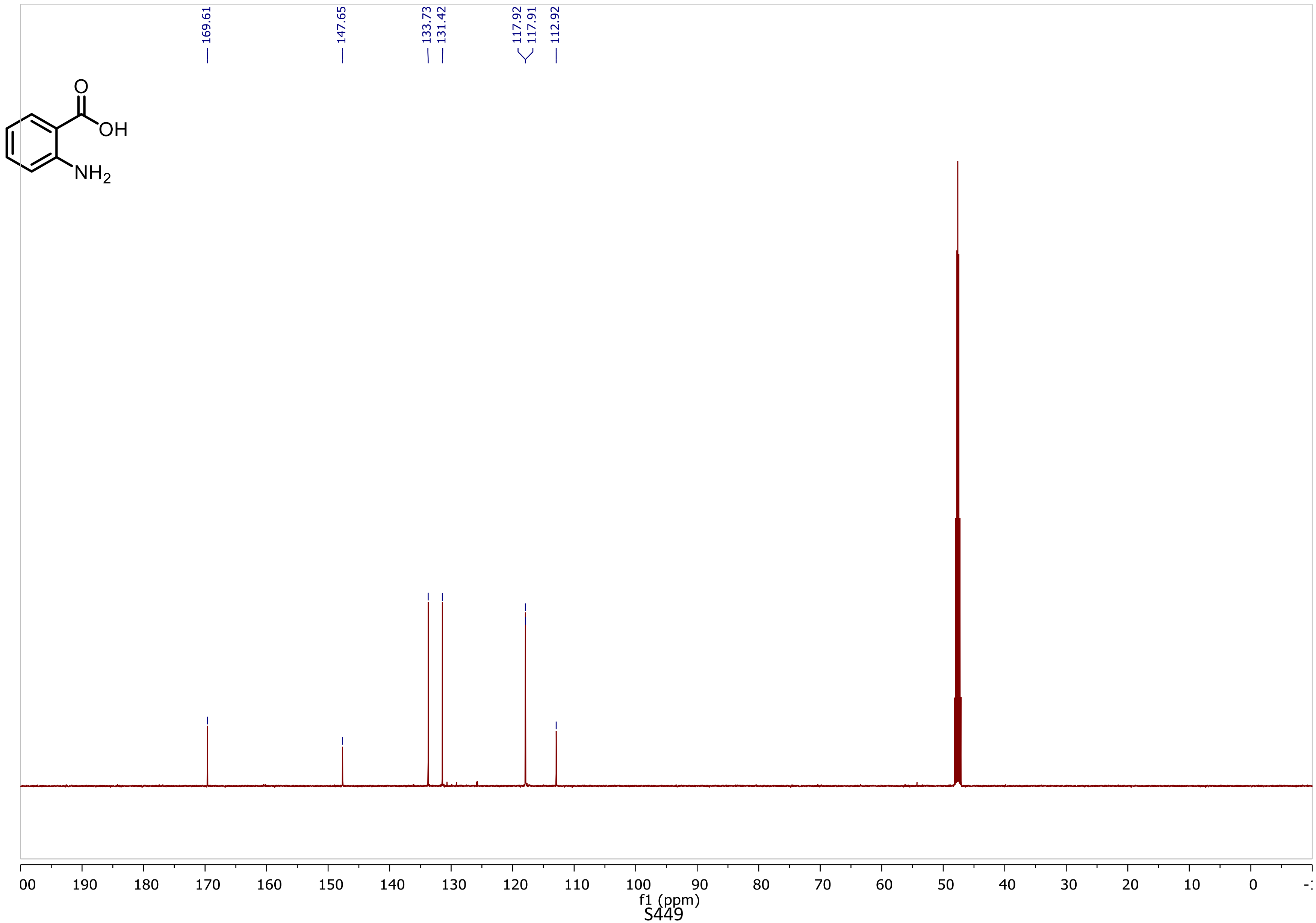

^{1}H NMR spectrum of quinazolin-4(3*H*)-one **7** in $CDCl_3$

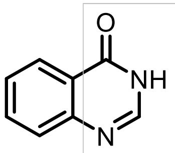

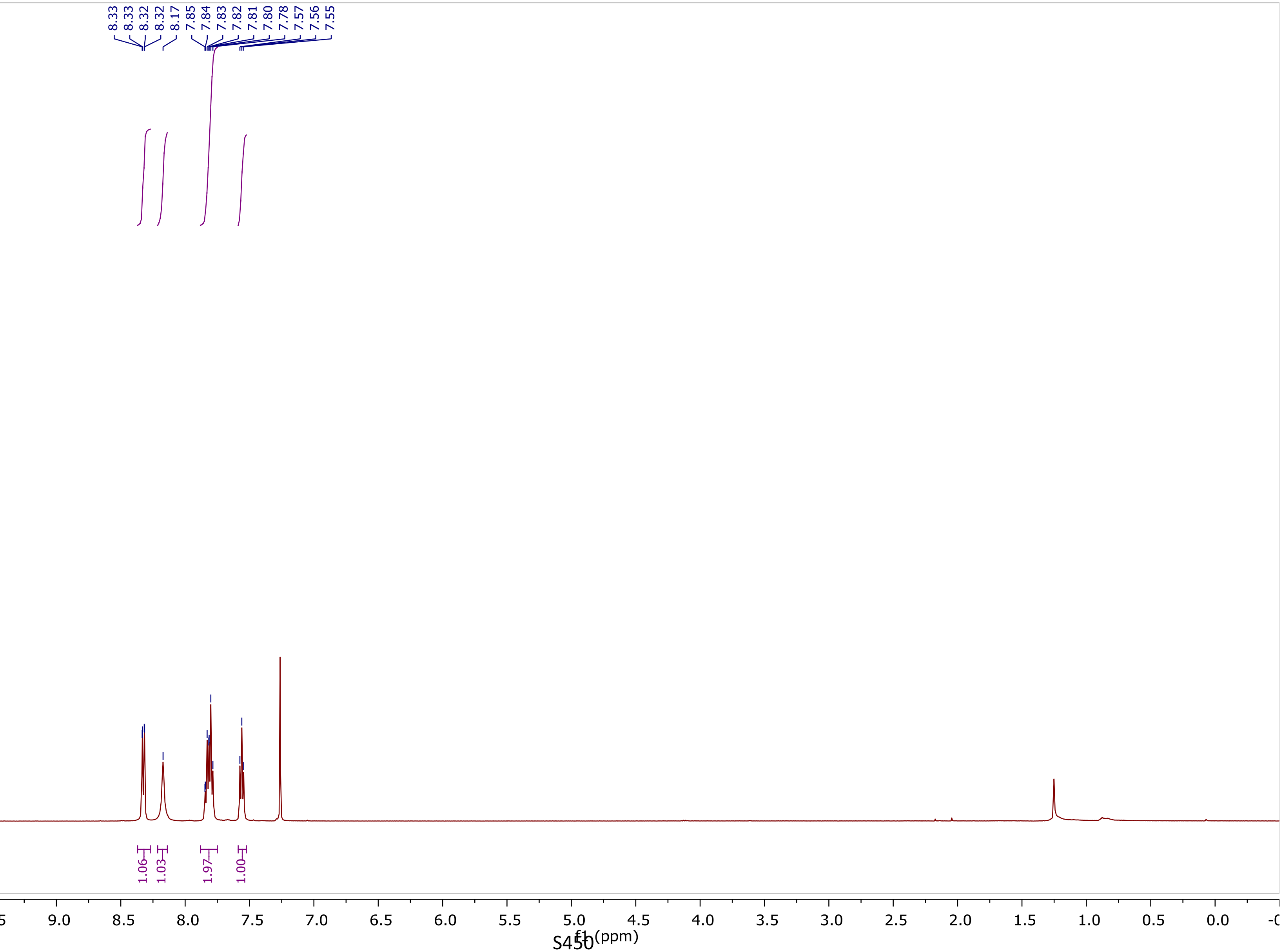

^{13}C NMR spectrum of quinazolin-4(3*H*)-one **7** in $CDCl_3$

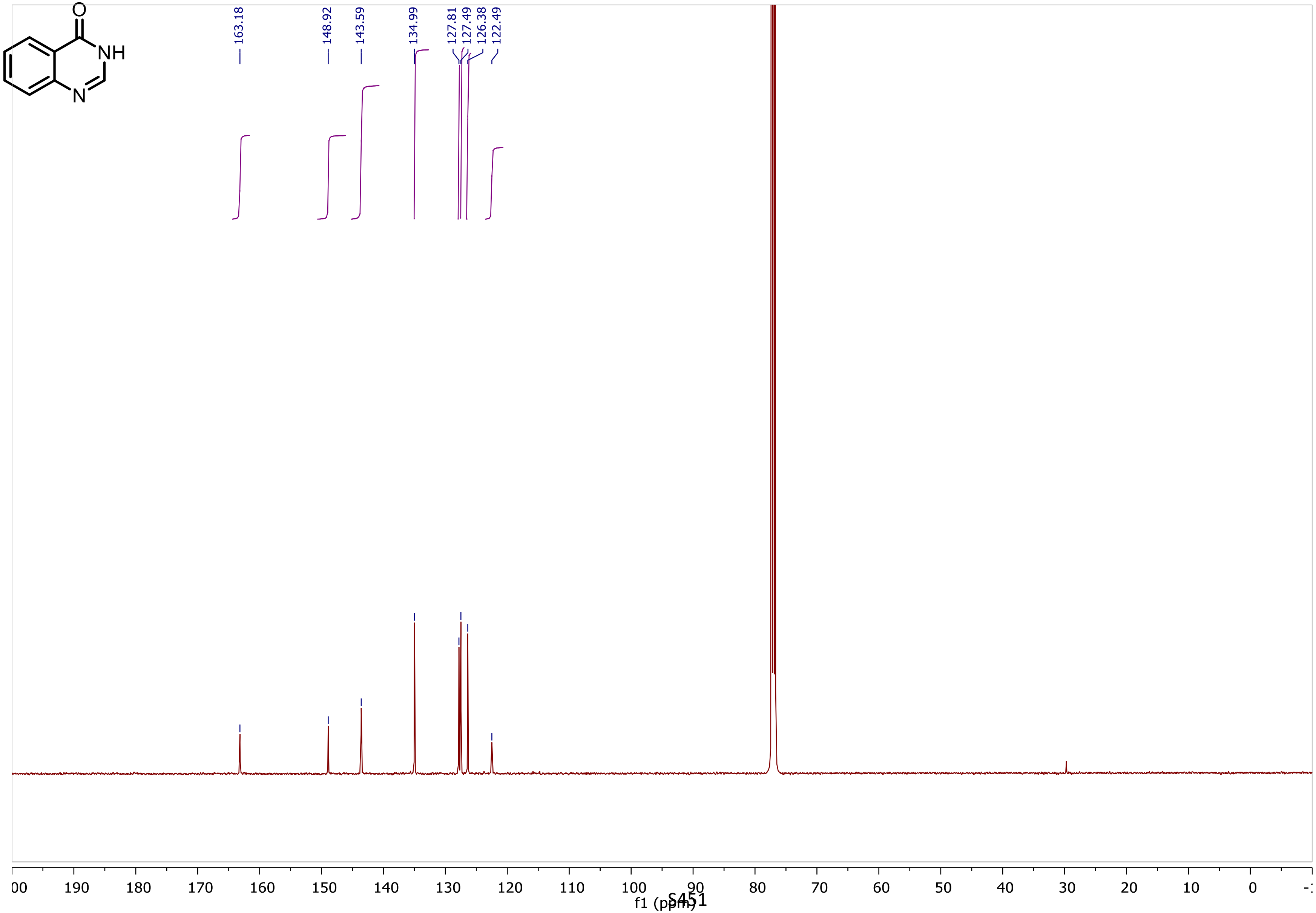

^{1}H NMR spectrum of 2-(*p*-tolyl)-4*H*-benzo[*d*][1,3]oxazin-4-one **8** in $CDCl_3$

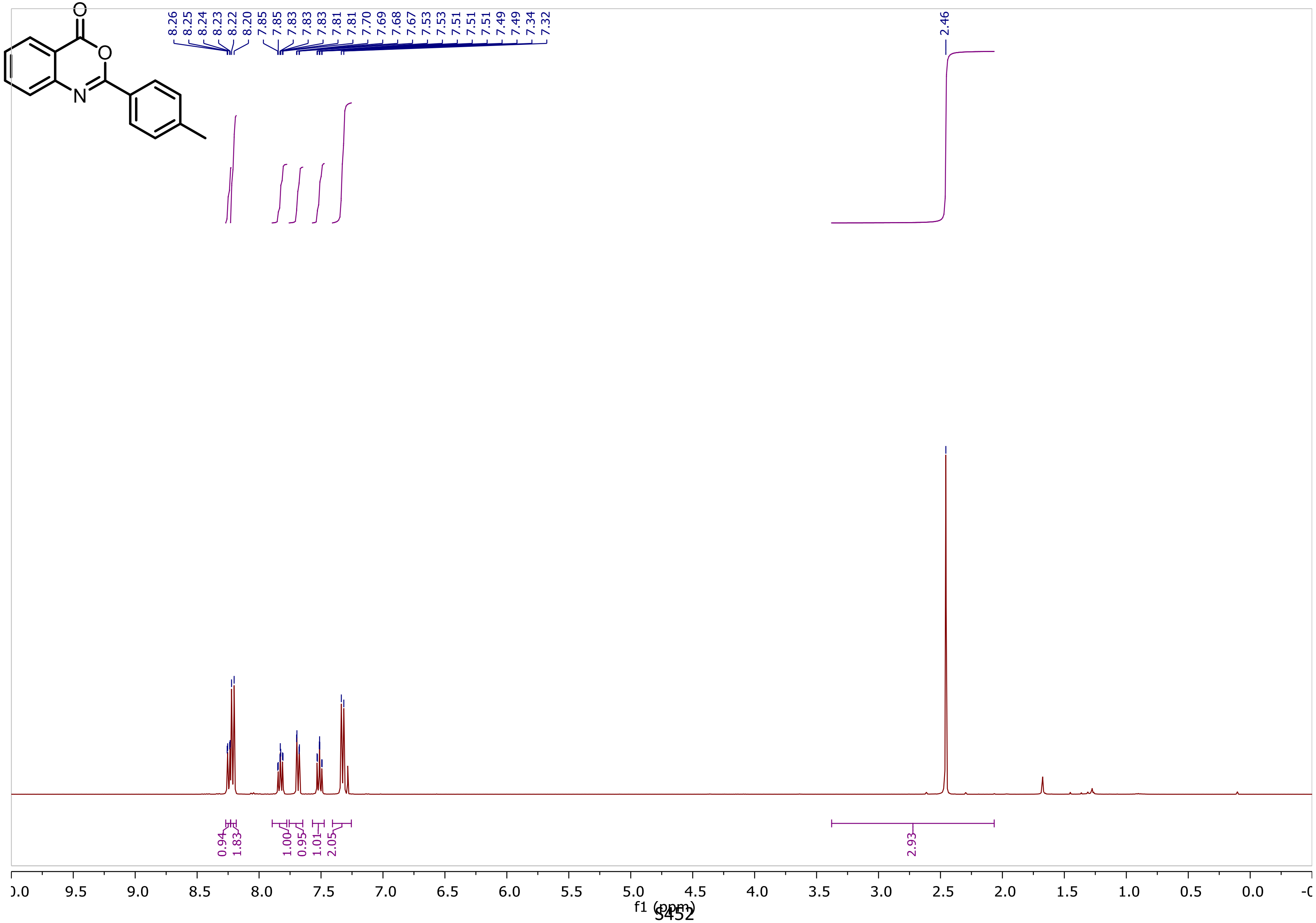

^{13}C NMR spectrum of 2-(*p*-tolyl)-4*H*-benzo[*d*][1,3]oxazin-4-one **8** in $CDCl_3$

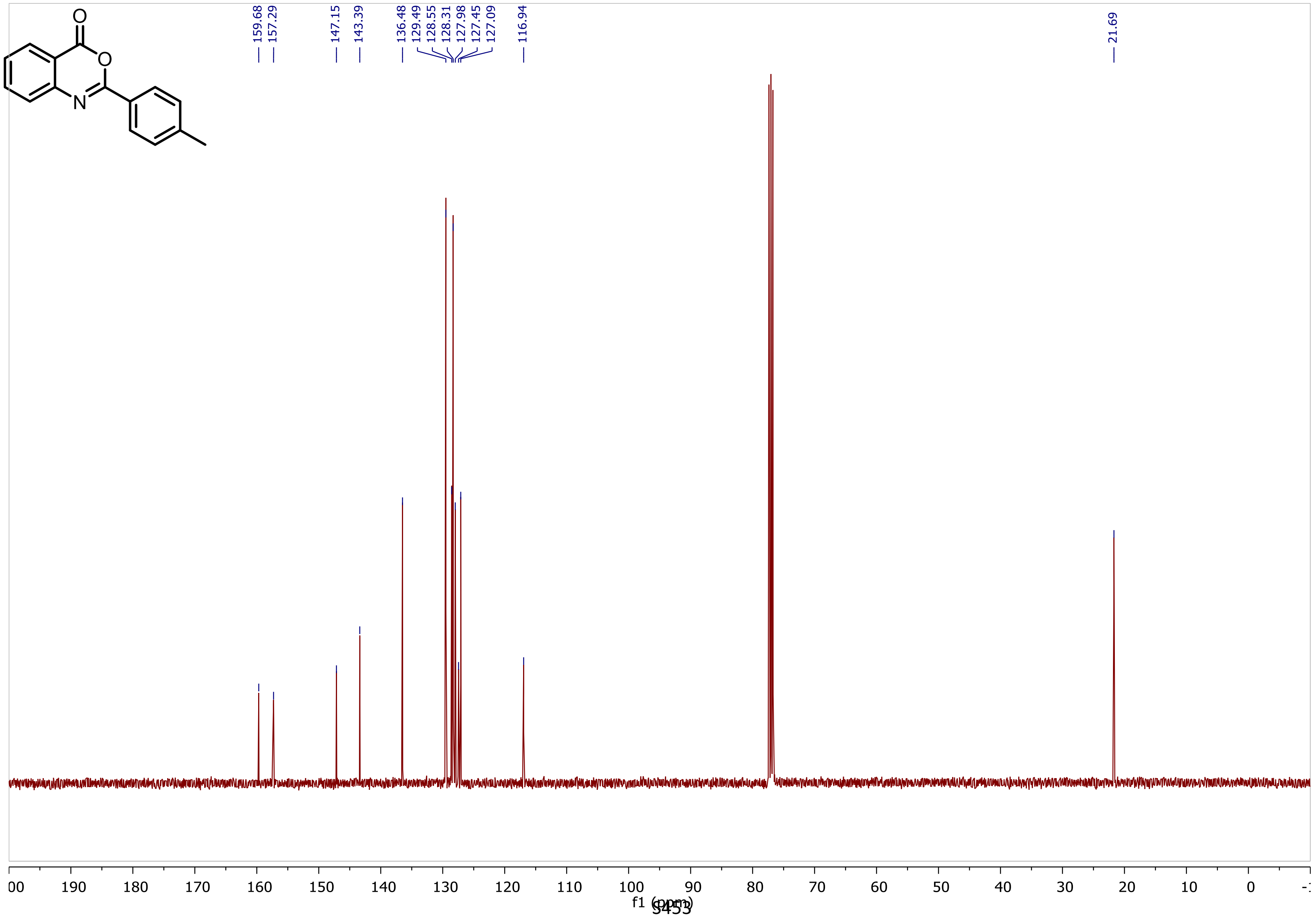

Supplement: SC-014-D3SC03293K-s001 [file SC-014-D3SC03293K-s001.pdf]
